# PERMA.teach: a study on the effectiveness of a standardized positive education training program in Austria

**DOI:** 10.3389/fpsyg.2025.1516572

**Published:** 2025-04-08

**Authors:** Martin Wammerl, Ulrike Lichtinger

**Affiliations:** ^1^Faculty of Psychotherapy Science, Sigmund Freud Private University, Vienna, Austria; ^2^Institute for Positive Psychology and Mental Coaching, Graz, Styria, Austria; ^3^Institute of Social Sciences, International University of Applied Sciences, Regensburg, Germany

**Keywords:** positive education, PERMA model, teacher wellbeing, student wellbeing, education intervention

## Abstract

**Introduction:**

PERMA.teach is the largest Positive Education initiative in German-speaking countries, offering a structured training program to help teachers integrate well-being interventions based on the PERMA model into their classrooms. Embedded in the third phase of Austrian teacher training, the program aims to enhance the well-being and mental health of both teachers and students.

**Methods:**

A representative sample was collected from primary and middle schools across five Austrian federal states. Schools were randomly assigned to either an intervention group, which implemented PERMA-based strategies, or a control group (education as usual). Well-being was assessed using the PERMA-Profiler and the Satisfaction With Life Scale at three time points. The final sample included 155 teachers (65 in the control group) and 1,402 students (657 in the control group). Additionally, interviews with 10 randomly selected teachers and a survey of 120 participants were conducted to explore the program’s implementation.

**Results:**

Teachers in the intervention group reported significantly higher life satisfaction and greater positive emotions compared to those in the control group. They also experienced increased engagement, characterized by higher levels of flow and greater use of their character strengths in the school context. No significant changes were observed in the control group. Qualitative findings emphasized the importance of autonomy in applying Positive Education and highlighted emotional contagion among teachers as a key factor influencing both training and classroom implementation.

**Discussion:**

The results confirm the effectiveness of PERMA.teach in promoting teacher well-being and fostering a positive school environment. The program’s success is driven by the autonomy of its users and the need for a broad range of intervention options to support individualized application. PERMA.teach effectively enhances teacher well-being, fosters a flourishing educational environment, and provides valuable insights into the implementation of Positive Education.

## 1 Introduction

Teachers play a crucial role in passing on cultural values to young people. The development of their worldview is significantly shaped by the attitudes and perspectives of teaching staff, whether they convey pessimism and distrust or foster a consciously positive and optimistic outlook. Research supports the idea that teachers’ attitudes profoundly influence students’ emotional and cognitive development, as well as their overall perceptions of the world (Roffey, 2012). Positive teacher behaviors and attitudes have been shown to enhance student well-being and resilience, creating a more supportive and thriving school environment (Fredrickson, 2003). Conversely, negative emotional climates in classrooms can hinder learning and psychosocial development (Hamre and Pianta, 2006). Many aspiring teachers start with the goal of positively impacting student learning and engagement, but they often feel overwhelmed by the many demands of their role (Yin et al., 2016; Buchanan et al., 2013).

When it comes to the variables that matter most in creating positive learning environments in school, the well-being of teachers is crucial for attracting, retaining, and sustaining educators in the profession as well as for student learning (Turner and Thielking, 2019a). However, the idea of focusing on teachers’ well-being is relatively new, mostly promoted by research in the field of Positive Education (Roffey, 2012). Moreover, most research has focused on the issues that lead to teacher stress and burnout (McCallum et al., 2017; Antoniou et al., 2013). A recent meta-analysis reviews the effectiveness of teacher well-being intervention programs. The findings indicated a significant positive effect, with study quality, intervention type, and duration identified as significant moderators (Li et al., 2024).

Considering the global development of mental health in student populations, as some studies suggest that depression may be on the rise, with strong evidence indicating a significant increase in self-harming and suicidal behaviors (Sachs et al., 2019). This suggests that the need for evidence-based intervention and prevention programs that focus on the well-being of students as well as teachers is more urgent than ever. Recent research in this field suggests that fostering optimism, trust, and hope for the future has a positive impact on how young people perceive the world. In response Positive Education interventions were designed, evaluated, and implemented in the field (White and Murray, 2015; Waters, 2017).

Recent research shows that Positive Education programs may foster higher levels of creativity, leadership skills, and emotional intelligence in students (Leventhal et al., 2015). Additionally, these programs have been shown to enhance academic performance and significantly improve mental health (Seligman et al., 2009; Adler et al., 2016).

The theoretical framework of the PERMA.teach program is based on Geelong’s Positive Education model (Norrish, 2015). Its core is Seligman’s PERMA model (2011), which emphasizes five independent yet interconnected components that foster well-being: Positive Emotions, Engagement, Relationships, Meaning, and Accomplishment. These interconnected components provide a holistic approach to fostering well-being, with mechanisms rooted in well-established psychological theories and supported by recent research. The following sections outline the mechanisms and research findings on the contribution of PERMA factors to teacher well-being and, subsequently, their impact on student well-being. Positive emotions, as described in Fredrickson’s broaden-and-build theory (Fredrickson, 2003, 2013), benefit not only students but also teachers. Research demonstrates that teachers who frequently experience positive emotions exhibit higher resilience and a lower risk of burnout. A recent study highlights that positive emotions significantly contribute to teachers’ well-being and job satisfaction (Dreer-Goethe, 2021). Additionally, gratitude and optimism have been shown to enhance teacher well-being and foster a positive work environment (Emmons and McCullough, 2003).

Engagement among teachers is closely linked to experiences of flow and the application of personal strengths in teaching. Studies indicate that engaged teachers report higher job satisfaction and improved psychological well-being (Siddique et al., 2022). The use of character strengths, such as hope and curiosity, not only supports teachers’ personal motivation but also creates a motivating and enriching learning environment (Janapati and Vijayalakshmi, 2024).

Positive relationships within the school community, including collegial interactions and teacher-student relationships, are essential for teacher well-being. Supportive relationships reduce stress, increase job satisfaction, and foster a sense of belonging. Research highlights that strong relationships with colleagues significantly enhance teacher resilience and reduce workplace stress (Collie et al., 2012). Furthermore, positive teacher-student relationships enhance students’ school engagement (Martin and Collie, 2019).

A sense of meaning in teaching, derived from contributing to students’ development and experiencing purpose in educational tasks, is a key factor in teacher well-being. Teachers who perceive their work as meaningful report higher motivation and emotional engagement. Recent studies show that purpose-driven teaching is associated with increased resilience and reduced emotional exhaustion (Turner and Thielking, 2019b).

Accomplishment, achieved through meeting professional goals and mastering teaching practices, significantly enhances teachers’ self-efficacy and overall well-being. Teachers who experience progress and success in their work report higher confidence and emotional resilience. Recent research confirms that goal-setting and professional achievement are critical for sustaining well-being (Shu, 2022).

The PERMA model integrates and builds upon foundational theories of well-being. Ryff’s Six-Factor Model of Psychological Well-Being (Ryff, 1989) identifies autonomy, personal growth, and environmental mastery, alongside purpose and relationships. While both models emphasize multidimensional well-being, PERMA operationalizes its components more specifically for interventions, particularly in educational settings.

Self-Determination Theory (SDT) (Deci and Ryan, 2000) emphasizes autonomy, competence, and relatedness as psychological needs. PERMA aligns with these elements while expanding to include Positive Emotions and Engagement, providing a broader framework for promoting flourishing.

The Broaden-and-Build Theory (Fredrickson, 2013) underpins the Positive Emotions component of PERMA, emphasizing how positive emotions build enduring psychological resources.

By integrating insights from these theories, PERMA offers a practical, evidence-based framework that addresses both individual and systemic aspects of well-being. Its application in schools emphasizes strengths-based approaches, supportive relationships, and purposeful engagement, making it a robust model for fostering flourishing in educational contexts. The effectiveness of Positive Psychology, or more specifically Positive Education programs, in school contexts has been strongly supported by research conducted in various countries, including the United States, the United Kingdom, Spain, and Australia. Studies by Park and Peterson (2009), Proctor et al. (2011), and Shoshani and Slone (2013) demonstrate that focusing on and strengthening individual strengths in schools can lead to significant improvements in well-being, higher academic performance, and a reduction in problematic behaviors such as unexcused absences and classroom disruptions.

The PERMA.teach project emerged as an intervention program accompanied by research to investigate its effectiveness in German-speaking countries. Grounded in findings from Positive Education and following the Geelong framework model (Lichtinger, 2022), PERMA.teach aims to enhance the well-being of students and teachers and strengthen self-efficacy within school communities through targeted interventions. The initiative includes a standardized training program designed to promote each PERMA factor in the school environment through specific interventions, thereby positively influencing the overall well-being of participants (teachers, students, and school leaders). By applying PERMA.teach, the program emphasizes individual strengths while also fostering stronger social relationships within the school. The standardized training, as described in the preceding chapter, was delivered through professional development sessions, equipping teachers to implement positive psychological interventions in their classrooms. A distinctive feature of PERMA.teach, beyond its scale and the number of participants involved, is its focus on addressing the specific needs and challenges of the educational system in German-speaking countries. The program’s aim is not only to create short-term positive changes but also to have a long-term, sustainable impact on the quality of life and well-being within school communities.

The central research question emerging from the current state of research is: How effective is the PERMA.teach program in enhancing the well-being of teachers and students, as measured by PERMA and life satisfaction, in German-speaking school communities? The central hypotheses derived from this research question propose that participation in the PERMA.teach program will significantly enhance the well-being of both teachers and students. Specifically, it is hypothesized that teachers who complete the program will show notable improvements in the components of the PERMA model—Positive Emotions, Engagement, Relationships, Meaning, and Accomplishment—compared to teachers in control groups. Additionally, students in classrooms led by these trained teachers are expected to report higher levels of well-being, as measured by the same PERMA components, relative to students taught by teachers who did not participate in the program. To this end, a mixed-methods research design was employed, combining and triangulating quantitative and qualitative methods.

## 2 Materials and methods

### 2.1 Design

The research design included both quantitative and qualitative components. The core of the quantitative component was a survey of teachers and students who participated in the PERMA.teach program. Data from these participants were compared with data from control schools that matched the training schools in terms of age and school type but did not undergo the program during the same period. The teachers surveyed were the same ones who taught the students being evaluated in the study. The survey was conducted at three measurement points (pre, post, and follow-up) over a period extending from March 2022 to May 2023, allowing data collection over three semesters. Participating schools were divided into an intervention group, which participated in the PERMA.teach program, and a control group, which continued regular instruction without the interventions of Positive Psychology. Due to the challenge of finding schools willing to serve as control groups, the control group design was only implemented from the second measurement point onward. Knowledge and training in Positive Psychology and Positive Education were collected as control variables and included in the calculations. Consent forms detailing all study content and a data privacy statement were sent to all participating schools and the parents of participating students.

The qualitative part of the study aimed to gain in-depth insights into the effects of the intervention from the perspectives of teachers and developers (experts) through randomized interviews. Of particular interest for the project development was identifying which interventions were perceived as particularly significant for the project’s success and what changes were observed. A follow-up survey collected low-threshold feedback from all participating teachers at the end of the project to obtain a comprehensive assessment of the intervention, particularly regarding the quality of the event format and the use of the content in their own educational everyday life.

### 2.2 Participants

The quantitative sample of teachers comprised a total of 609 datasets from 412 teachers across six federal states. The final dataset was cleaned of missing data and included only datasets from teachers who participated in all three measurement points (intervention group) or two measurement points (control group). This resulted in a final dataset of 155 teachers, with 90 in the intervention group and 65 in the control group. The average age was 44 years with a standard deviation of 11 years. The average service time of the teachers was 14 years. Twenty-one participants were school principals. The student sample consisted of a total of 4,545 datasets from 2,149 students across six federal states. After data cleaning, using the same criteria as for the teacher sample, the final dataset included 1,402 students, forming the basis for the calculations. The gender distribution was approximately equal, with 681 male, 707 female, and 14 diverse students participating in the survey. The distribution between the intervention and control groups was also nearly equal, with 657 students in the control group and 745 in the intervention group. The average age was 11 years, with a standard deviation of 2 years, and the age range was 8 to 15 years. The data in the student sample was collected during the same period.

For the qualitative part, a total of 21 structured interviews were conducted. Fifteen teachers and six PERMA experts (experts who created the intervention and conducted the teacher training workshops) were interviewed online. The sample included teachers from both primary and secondary levels, as well as lecturers at pedagogical colleges, to provide a representative perspective for this educational area. All interviewees had completed all training units to discuss their experiences comprehensively. The follow-up survey, conducted from December 18, 2023, to January 14, 2024, received 120 responses, including 81 teachers and 39 multipliers.

### 2.3 Description of the PERMA-based intervention program

The intervention program was designed as a blended learning module, combining analog and digital components to provide a comprehensive overview of the PERMA model. It focused specifically on its pillars: Positive Emotions, Engagement, Relationships, Meaning, and Accomplishment. The program’s primary aim was to enhance participants’ well-being and resilience through targeted activities and evidence-based methodologies, while also facilitating the transfer of learned concepts into everyday school contexts.

**Figure 1:**
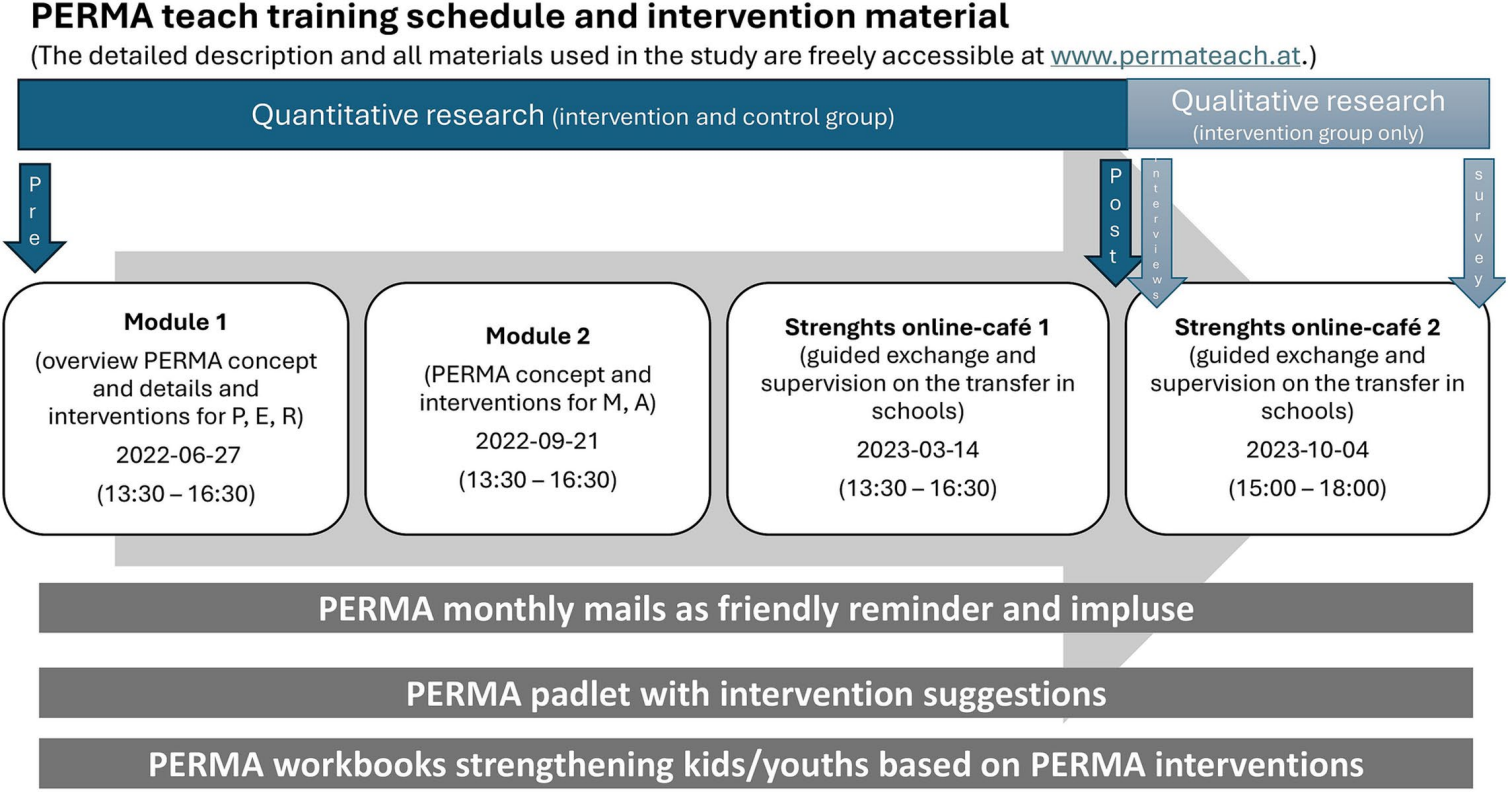
Illustration of the intervention timeline, detailing the activities conducted at each measurement point (pre, post, and follow-up) separately for the intervention and control groups.

The intervention consisted of two modules and two follow-up Strengths Online Cafés, as summarized in Figure 1. Module 1, conducted on June 27, 2022, was delivered in-person and focused on the PERMA pillars of Positive Emotions, Engagement, and Relationships. Module 2, held on September 21, 2022, was conducted via Zoom and addressed the pillars of Meaning and Accomplishment. Both modules ran for 3 h and provided theoretical input, interactive exercises, and practical planning for implementing PERMA-based interventions in schools.

Following these modules, participants attended two 3-h Strengths Online Cafés designed to support the transfer of learned strategies into their professional contexts. The first café, on March 14, 2023, and the second, on October 4, 2023, offered guided exchanges, peer learning opportunities, and supervision on the application of PERMA interventions. These sessions provided a collaborative platform for participants to share their experiences, discuss challenges, and refine their approaches.

Throughout the program, participants were equipped with a variety of materials and resources. A Padlet served as a central repository for intervention suggestions, downloadable exercise guides, video tutorials, and reflective worksheets. Weekly motivational PERMA emails provided friendly reminders and additional exercises to encourage the practical application of the program’s concepts. Participants also had access to workbooks, including *“Jedes Kind stärken”* and *“Jugend stärken,”* as well as a strengths-based treasure hunt app designed to reinforce learning in a playful and interactive way.

The modules and online cafés followed a structured format. Each session began with an introduction to the theoretical foundations of the PERMA model, followed by evidence-based exercises tailored to each pillar. For instance, Positive Emotions were explored through activities like “Three Blessings,” “Gratitude Letter,” and “Savoring,” inspired by Fredrickson (2001). Engagement activities utilized Seligman and Peterson’s character strength framework (2004) and included “Strength Spotting,” “Positive Self-Report,” and practical strength applications. Relationships were developed using Deci and Ryan (2000) and Werner (2008), with activities like “Active Constructive Responding,” “Random Acts of Kindness,” and “Perfect Surprise.”

The Meaning pillar incorporated Frankl’s (2004) and Seligman’s (2011) frameworks, with exercises such as “Good Life Goals” and “One Door Opens,” emphasizing gratitude and identifying opportunities in challenges. Accomplishment was addressed through Seligman’s (2011) research on achievement, with activities focusing on the Pygmalion Effect, tiny habit formation, power posing, and fostering a growth mindset to enable participants to set and sustain meaningful goals.

The program was delivered by a team of experienced educators and trainers with advanced degrees in Positive Psychology and Education, certifications in coaching, and at least 5 years of experience in implementing positive psychology interventions in educational settings. Their expertise ensured a high level of professionalism and relevance, combining scientific rigor with practical applicability.

For replicability, Figure 1 visually summarizes the program’s structure and timeline, detailing the modules, follow-up sessions, and supporting resources. All program materials are freely accessible at www.permateach.at.

### 2.4 Instruments

The first part of the quantitative survey included a sociodemographic questionnaire section. In the second part of the survey, specific questionnaires were used to assess the building blocks of well-being (Seligman, 2018). The PERMA Profiler, originally developed by Butler and Kern (2016) and later adapted into German by Wammerl et al. (2019), was used in this study to measure the PERMA factors of well-being for adults aged 18 to 65. The basic version, which was used for the teacher sample, includes 23 items. Fifteen items measure the PERMA factors, with three items assigned to each factor. Seven items measure negative emotions and self-assessed health status, and one item measures overall subjective well-being for comparison with other standardized questionnaires. For the PERMA.teach project, an adapted version of the German PERMA-Profiler was developed for students, allowing valid measurement of the components of well-being for ages 8 to 15. The psychometric evaluation showed good reliability (Cronbach’s Alpha = 0.81). The items could be psychometrically assigned to their specific factors in a confirmatory factor analysis, and the intercorrelated PERMA factor model showed a comparably good fit to the adult version. A CFA was conducted to test the hypothesized five-factor model of the children’s version of the PERMA Profiler. The factor Engagement was the only factor that had to be excluded from the analyses as it demonstrated low correlations with the remaining factors, which is theoretically inconsistent with the underlying model; consequently, it was also excluded from the subsequent analyses for the children’s dataset. Model fit indices indicated an acceptable fit for the adapted intercorrelated four-factor model: χ^2^(590) = 211.26, *p* < 0.001, CFI = 0.94, RMSEA = 0.08 (90% CI [0.07, 0.10]), and SRMR = 0.04. Standardized factor loadings ranged from 0.45 to 0.86. The final model aligns with theoretical expectations, supporting the scale’s construct validity (Wammerl et al., in preparation).

The second important construct of Positive Psychology measured in this study was life satisfaction (Satisfaction with Life, Diener, 1985). The scale used to measure life satisfaction was the German version of the Satisfaction with Life Scale (SWLS, Janke and Glöckner-Rist, 2012). The five-item version for adults showed good factorial validity and reliability and was used for comparison with other international studies. For the student sample, the German version of the Satisfaction with Life Scale for Children (SWLS-C, Lang and Schmitz, 2020) was used, which also demonstrated sufficient reliability, concurrent validity, and discriminant validity.

For the interviews, three structured versions were developed: one for the teachers, a slightly modified version for the multipliers, and one for the experts. The categories used in the interviews included (1) motivation to participate in the PERMA.teach project, (2) prior knowledge and experiences with Positive Psychology and Positive Education, (3) feedback on the content and quality of the training modules, (4) implementation in teaching and sustainability, and (5) observations of changes in students and personal changes, particularly the impact on professional development. The interviews were conducted online via Zoom, recorded, transcribed, and analyzed using MAXQDA. The follow-up survey consisted of a combination of 18 scaling and three open-ended questions, specifically designed for the project.

## 3 Results

The following results pertain to the calculated mean values. To calculate the differences between the measurement points, a MANOVA with repeated measures was conducted. Following the MANOVA, mixed ANOVAs, repeated measures ANOVAs, and (paired) t-tests were performed to analyze the effects of specific PERMA factors. For *post hoc* testing, Bonferroni correction was used. Descriptive statistics and correlation coefficients for the PERMA-Profiler, its subscales, and the SWLS, separated for all measurement points and for the intervention and control groups, are presented in Appendices 1–5. The term IG refers to the intervention group, while CG denotes the control group. These abbreviations will be used consistently throughout the text to distinguish between the two groups.

### 3.1 Teacher specific results

To account for shared variance within the individual PERMA factors, a MANOVA was conducted. A non-significant multivariate effect of the intervention was observed, Wilks’ Lambda = 0.91, *F*_(5, 103)_ = 1.92, *p* = 0.09, ns., partial η^2^ = 0.09.

**Figure 2:**
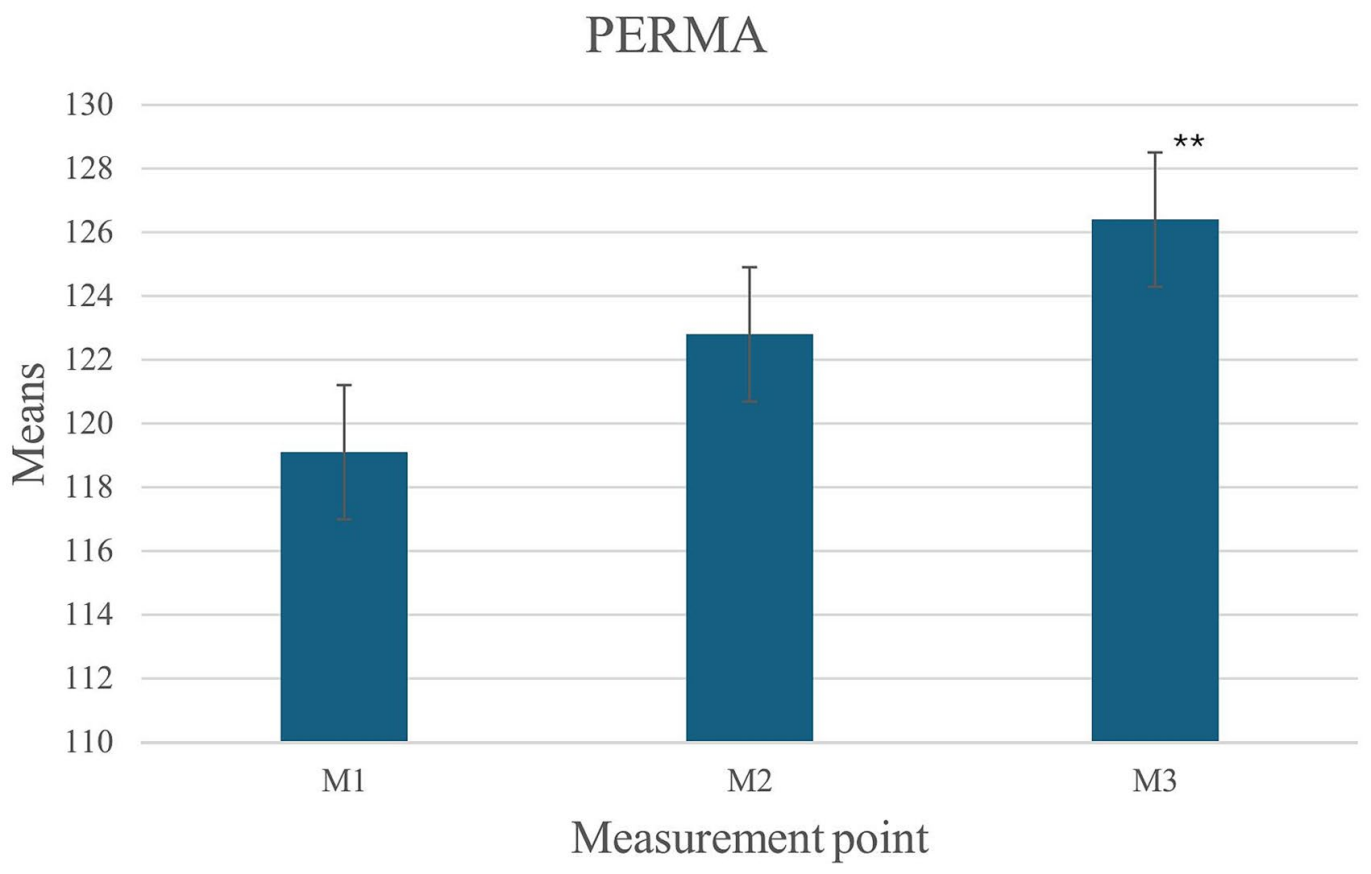
Changes in PERMA across the three measurement points.

Univariate ANOVA revealed that over the course of the project, a significant increase in the overall PERMA score (all PERMA factors combined) was observed. This increase was evident at the third measurement point [*F*_(1,37)_ = 3491,81; *p* < 0.01, *partial η^2^* = 0.25; *Post hoc* pairwise PERMA M1 = 119.1, PERMA M2 = 122.8, PERMA M3 = 126.4, *p* < 0.01, *d* = 0.78] (Figure 2).

In the control group, no significant differences were observed between the two measurement points for overall PERMA and life satisfaction. Both the overall PERMA score and life satisfaction remained unchanged throughout the project [*T*_(64)_ = −0.529, *p* = 0.60.; PERMA M2 = 121.3, PERMA M3 = 122.0, ns.; *T*_(64)_ = 0.84, *p* = 0.60, ns.; SWLS M2 = 28.7, SWLS M3 = 28.4, *p* = 0.41, ns.]. The effect on overall well-being could also be demonstrated at the level of the PERMA components. A significant increase over time in Positive Emotions was found [*F*_(1,37)_ = 18,24, *p* < 0.01, *partial η^2^* = 0.33]. No increase was observed between the first and second measurement points (M1 = 22.7, M2 = 23.8, *p* = 0.18, ns.); however, a significant increase in the experience of Positive Emotions was noted between the first and third measurement points (M3 = 24.8, *p* < 0.01, *d* = 0.69). A similar effect was observed for the Engagement factor [*F*_(1,37)_ = 14,71, *p* < 0.01, partial η^2^ = 0.29], with no change between the first and second measurement points (M1 = 22.0, M2 = 22.5, *p* = 0.92, ns.), while a significant increase was recorded between the first and third measurement points (M3 = 24.3, *p* < 0.01, *d* = 0.69). For the Positive Relationships factor, an increase in mean values was already measurable between the first and second measurement points [*F*_(1,37)_ = 8,0, *partial η^2^* = 0.18; M1 = 24.7, M2 = 26.0, *p* < 0.05, *d* = 0.46], and this effect remained stable until the third measurement point (M3 = 26.0, *p* = 0.10, ns.). The Accomplishment factor approached significance over the three measurements [*F*_(1,37)_ = 5.67, *p* = 0.07, partial η^2^ = 0.13; M1 = 24.1, M3 = 25.1, *d* = 0.39], while no difference was noted between the first and second measurement points (M2 = 24.8, *p* = 0.17, ns.). In contrast, no significant effect was found for the Meaning factor [*F*_(1,37)_ = 2.5, *p* = 0.12; M1 = 25.6, M2 = 25.7, M3 = 26.2, ns.] and the experience of Negative Emotions [*F*(1,37) = 3.33, *p* = 0.1, M1 = 9.47, M2 = 7.92, M3 = 7.73 ns.].

The intervention effect of the PERMA-teach program can be demonstrated by direct comparison with the values from the control group. Teachers who participated in the PERMA-teach program showed significantly more engagement than the control group. In contrast, the engagement of teachers in the control group remained constant between the measurement points [*F*_(1,107)_ = 9.2.; *partial η^2^* = 0.08, CG: M2 = 23.3, M3 = 23.1, *p* = 0.70; IG: M2 = 22.3, M3 = 24.1, *p* < 0.01, *d* = 0.67].

A similar effect was also observed for the overall PERMA score. An intervention effect approached significance [*F*_(1,107)_ = 3.18, *p* = 0.07, partial η^2^ = 0.03]. The intervention group showed an increase in the experience of overall PERMA in their school routines between the second and the third measurement point, whereas no difference was observed for the control group. (CG: *M1* = 121.25, M2 = 121.97, *p* = 0.55; IG: M1 = 121.64, M2 = 125.75, *p* < 0.01, *d* = 0.55).

**Figure 3:**
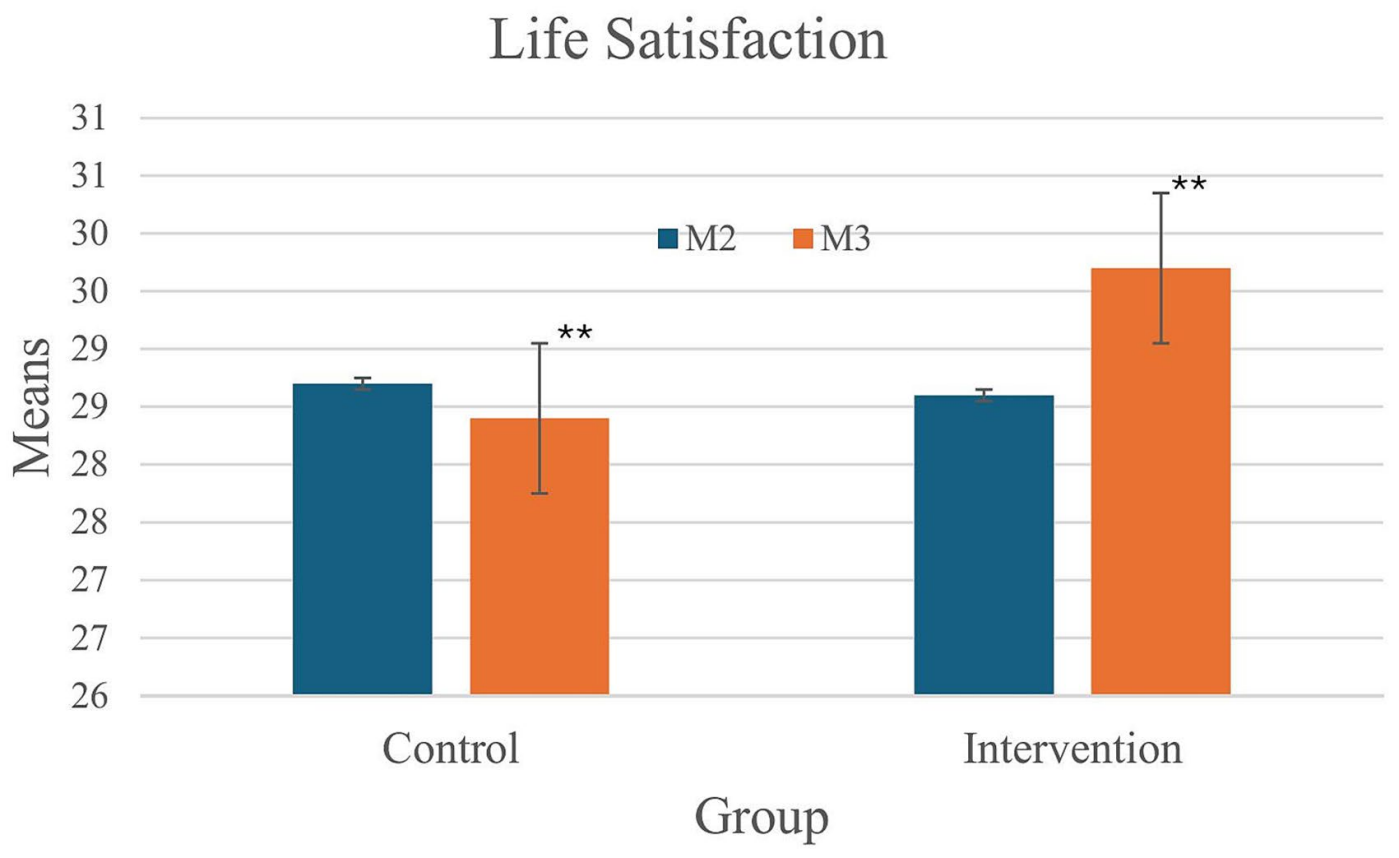
Significant intervention effect on life satisfaction of teachers between measurement 1 and measurement 2 for the intervention group compared with the control group.

A significant interaction effect was also found for Life Satisfaction [*F*_(1,107)_ = 6.88, *p* < 0.01, *partial η^2^* = 0.06]. Participants in the control group showed no differences between the two measurement points (CG: M2 = 28.7, M3 = 28.4, *p* = 0.36), whereas a significant increase over time was observed in the intervention group (IG: M2 = 28.6, M3 = 29.7, *p* < 0.01, *d* = 0.48) (Figure 3).

### 3.2 Student specific results

A MANOVA was also conducted for the student sample to account for shared variance within the individual PERMA factors. A non-significant multivariate effect of the intervention was observed, Wilks’ Lambda = 0.98, *F*_(4, 1,104)_ = 0.87, *p* = 0.48, ns., *partial η^2^* = 0.003.

In the univariate ANOVA, a significant decrease in Negative Emotions was observed for the intervention group [*F*_(1,404)_ = 13.79, *p* < 0.01, *partial η^2^* = 0.03]. The decrease between the first measurement points approached significance (M1 = 10.3, M2 = 9.6, *p* = 0.06, *d* = 0.27); and a significant decrease was noted between the first and third measurement points (M3 = 9.15, *p* < 0.01, *d* = 0.35). This effect was no longer significant when the control group was included in the analysis [*F*_(1,1,093)_ = 1.13, *p* = 0.29, CG: M2 = 9.88, M3 = 9.81; IG: M2 = 9.66, M3 = 9.23, ns.].

In the control group, no significant differences were observed between the two measurement points for overall PERMA and life satisfaction. Both the overall PERMA score and life satisfaction remained unchanged throughout the project [*T*_(644)_ = 1.23, *p* = 0.22.; PERMA M2 = 112.7, PERMA M3 = 111.9, ns.; *T*_(644)_ = 0.84, *p* = 0.55, ns.; SWLS M2 = 19.7, SWLS M3 = 19.6, *p* = 0.41, ns.].

In the student sample, no significant intervention effect on the overall PERMA scores [*F*_(1, 1,093)_ = 0.50, *p* = 0.48, ns.] and Life Satisfaction [*F*_(1, 1,092)_ = 0.75, *p* = 0.39, ns.] could be observed in the intervention group in contrast to the control group.

### 3.3 Qualitative results

**Figure 4:**
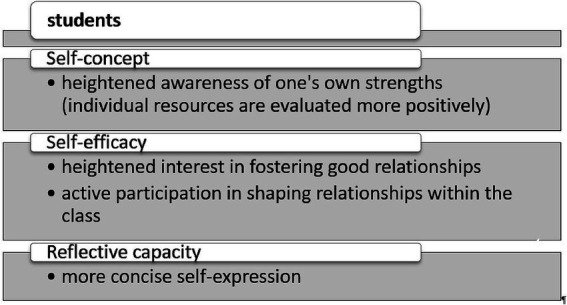
Major behavioral changes observed in students.

**Figure 5:**
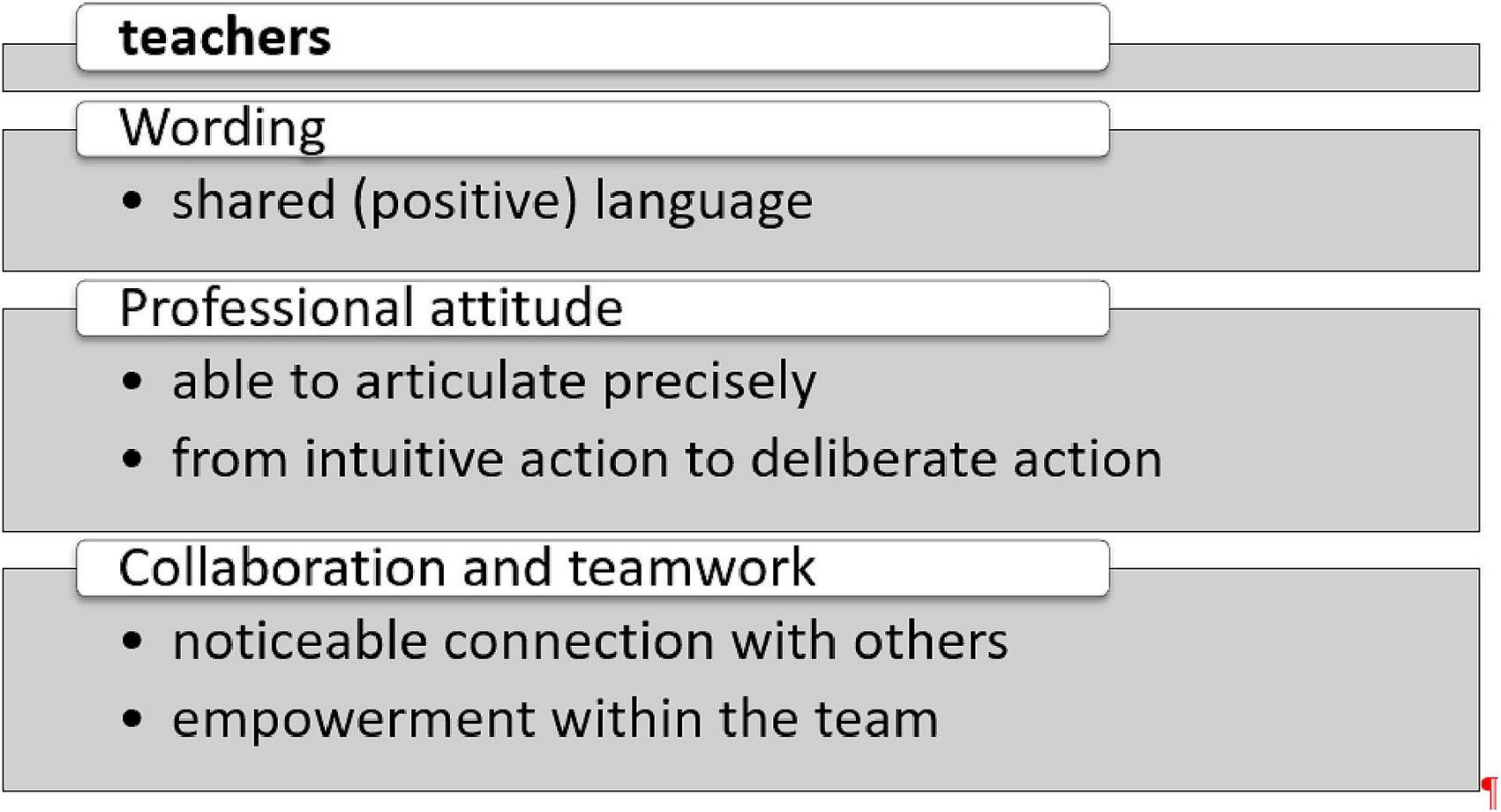
Major behavioral changes observed in teachers.

The interviews confirmed the positive findings obtained from the quantitative assessment. The teachers consistently provided favorable feedback regarding the training components, although no generalizability concerning key elements was evident. Instead, it became clear that different aspects were of central importance to the educators. The teachers accessed the interventions during the training in very diverse ways, ranging from the ‘What Went Well’ exercises to the Mandarin meditation, the wellness oasis in the library, the ALI principle, the encouragement videos, and the strengths book. The interviewees observed significant changes in both their students and themselves, as illustrated in Figures 4, 5.

In the concluding survey, the PERMA.teach program was rated an average of 8.87 on a 10-point scale, indicating that it was perceived as good and helpful. Approximately 50% of respondents strongly agreed with the statement, ‘I implement PERMA.teach in my work,’ while around 40% somewhat agreed.

## 4 Discussion

The PERMA.teach project represents a significant step in applying Positive Psychology principles to enhance well-being in school communities. Drawing on Seligman’s PERMA model (2011), the program aimed to increase well-being among both students and teachers through targeted interventions focused on cultivating Positive Emotions, fostering Engagement, building Positive Relationships, promoting Meaning, and supporting Accomplishment. The results of this study provide evidence for the effectiveness of such interventions, with positive changes observed particularly in Positive Emotions, Engagement, and overall Life Satisfaction for teachers.

### 4.1 Quantitative results

The quantitative findings highlight the specific effect of the PERMA.teach intervention. Across multiple measurement points, a significant increase in overall PERMA scores was observed for teachers in the intervention group, while the control group showed no meaningful change over time. Notably, the largest improvements were recorded between the second and third measurement points, suggesting that the program’s effects intensified as the participants became more engaged with the interventions.

For teachers, the intervention led to substantial improvements in well-being. In particular, the study found significant increases in Positive Emotions and Engagement, with teachers in the intervention group reporting higher levels of joy, optimism, and involvement in their professional activities. This aligns with existing research by Fredrickson (2003, 2013), which emphasizes the importance of positive emotions in building long-term psychological resources and resilience. Additionally, Life Satisfaction saw a marked improvement over the course of the intervention, reinforcing the idea that well-being initiatives in schools can not only prevent burnout but also actively enhance the overall quality of life for educators. However, the factor of Meaning showed no significant changes, raising questions about the effectiveness of the interventions in cultivating a deeper sense of purpose among teachers in the short term.

The non-significant multivariate analysis suggests that the intervention did not have a uniformly strong effect across all PERMA factors simultaneously, which represents a limitation of the study. This outcome highlights that individual effects, while meaningful, may not be sufficient to produce a significant overall multivariate effect across all dependent variables. However, this does not invalidate the observed significant changes in specific factors like Engagement and Positive Emotions, as these reflect targeted improvements within the intervention group compared to the control group. Such findings emphasize the value of examining individual factors, as meaningful improvements in specific domains can still occur even when a global effect is not detected. This limitation underscores the need for further research to explore the nuanced and targeted impacts of the intervention while considering variability in its effects across different domains (Maroof, 2012).

Within the student sample, no significant increases in overall PERMA scores were observed. The multivariate results for the student sample revealed a clearly non-significant overall effect for the PERMA factors, indicating that this transfer is not statistically robust. These results highlight the absence of a measurable multivariate impact.

This suggests that future studies should prioritize developing interventions that more effectively enable the desired transfer of positive outcomes to students. In this study, PERMA.teach was a specific training program designed for teachers. To complement such efforts, the development of a student-specific intervention program aimed at directly enhancing student well-being and sense of meaning within the school environment would be a critical focus for future research.

The exclusion of the Engagement factor in the analysis of the student sample may also reflect the challenge of developing a valid measurement instrument for constructs like PERMA for children across different age groups. The evaluation of Engagement involves the awareness of strengths and flow, which requires metacognitive abilities that are closely tied to neurocognitive development (Weil et al., 2013; Bjorklund, 2022). This highlights the need for further refinement of PERMA measurement instruments, tailored specifically to the developmental stages of children, to ensure validity and reliability across varying age groups.

It is also possible that significant differences exist between age groups and the native language of the students, which could influence their ability to benefit from the intervention. Future studies should explore these factors to better understand their impact and to tailor interventions that address the diverse needs of students from varying developmental stages and linguistic backgrounds.

### 4.2 Qualitative results

The qualitative interviews provided rich insights into the experiences of teachers and school leaders who participated in PERMA.teach. Teachers reported positive changes not only in their professional lives but also in their personal well-being. Many mentioned that the program introduced new tools and strategies—such as gratitude exercises, meditation practices, and wellness spaces—that they found valuable for both themselves and their students.

Interestingly, the qualitative data revealed that different interventions resonated differently with participants, suggesting that the impact of the PERMA.teach program may be highly individualized. Some teachers emphasized the benefits of mindfulness exercises, while others found the focus on strengths-based approaches (such as the strengths book) to be particularly impactful. This indicates that offering a variety of tools and approaches allows participants to select interventions that align with their personal preferences and teaching styles, thereby increasing the overall effectiveness of the program. The qualitative findings also pointed to significant improvements in classroom dynamics, with teachers reporting better student engagement, more positive peer interactions, and a noticeable shift in classroom atmosphere towards a more supportive and positive environment.

Although no significant effects were found in the quantitative analyses for the student sample, qualitative interviews revealed positive transfer effects from trained teachers to students. Students demonstrated improved self-concept (greater awareness of strengths), increased self-efficacy (interest in fostering relationships), and enhanced reflective capacity (more concise self-expression). These findings highlight the intervention’s potential impact through teacher-student interactions. Despite the generally positive feedback, the interviews also highlighted challenges. Several participants mentioned difficulties in sustaining the interventions over time due to the pressure of academic demands and curriculum constraints. This indicates that while short-term interventions can be effective, long-term implementation may require structural changes at the institutional level, such as integrating well-being initiatives into school curricula and providing ongoing support for teachers to maintain their well-being.

### 4.3 Scientific implications and future research

The findings from PERMA.teach offer significant contributions to the field of Positive Education and well-being in schools. First, the study confirms that Positive Education programs can lead to measurable improvements in teacher well-being. The success of PERMA.teach aligns with previous studies (Proctor et al., 2011; Shoshani and Slone, 2013), which suggest that well-being interventions focused on individual strengths and positive emotions can significantly enhance both academic and psychological outcomes in educational settings. The promising results of PERMA.teach open several important areas for future research: While the study showed improvements in well-being over three measurement points, future research should focus on the long-term sustainability of these changes. Do the effects of the PERMA.teach program persist beyond the immediate post-intervention period? This is particularly relevant given the challenges teachers reported in maintaining the interventions amidst other professional demands. Longitudinal studies tracking well-being over multiple years would provide critical insight into whether short-term interventions can produce lasting changes in the school environment.

The lack of significant change in the Meaning component invites further investigation. One possible explanation is that developing a deeper sense of meaning and purpose may require longer-term interventions or more personalized strategies. Previous research has suggested that meaning is often linked to existential or spiritual factors that are not easily addressed through short-term programs (Zhu et al., 2024). Therefore, future interventions might benefit from exploring more targeted methods for fostering meaning, such as mentoring programs or opportunities for students and teachers to engage with causes beyond the immediate school context.

The PERMA.teach program was conducted in German-speaking regions, but how would similar interventions fare in different cultural or educational contexts? Cross-cultural studies that examine the effectiveness of Positive Education programs in diverse settings would help determine the universality of the PERMA model. They would also allow researchers to identify cultural factors that may influence the success of such programs and suggest possible adaptations.

Further research should investigate the underlying mechanisms that drive improvements in well-being through PERMA-based interventions. What specific processes (e.g., changes in mindset, shifts in classroom dynamics, improved teacher-student relationships) contribute to the observed improvements in well-being? Understanding these mechanisms could help refine future interventions to be more targeted and effective.

Future research should consider additional positive psychology constructs, such as emotional intelligence, gratitude, psychological capital, and self-esteem, to provide a more holistic understanding of well-being in educational settings. Emotional intelligence supports interpersonal relationships and stress management (Petrides et al., 2016), gratitude fosters positive classroom climates (Froh and Bono, 2008), psychological capital enhances resilience and engagement (Luthans et al., 2007), and self-esteem promotes academic success and adaptive coping (Orth and Robins, 2014). Including these factors could deepen insights into how interventions promote flourishing in schools.

Finally, research should explore how well-being interventions can be more fully integrated into the broader school system. What structural changes are needed to support the ongoing use of PERMA principles in everyday teaching practice? This could include exploring the role of school leadership in promoting well-being initiatives, as well as policies that allow for greater flexibility in curriculum and assessment to prioritize well-being alongside academic achievement.

In summary, PERMA.teach has demonstrated the potential of Positive Psychology interventions to enhance well-being in schools. While the program has shown considerable success, particularly in promoting positive emotions and engagement among teachers, further research is needed to explore its long-term impact, the cultivation of meaning, and its adaptation to diverse cultural and educational contexts. By addressing these questions, future studies can build on the foundation laid by PERMA.teach to develop more comprehensive and sustainable approaches to well-being in education.

## Data Availability

The datasets presented in this study can be found in online repositories. The names of the repository/repositories and accession number(s) can be found in the article/Supplementary material.
