# Supplementary material for: PERMA.teach: a study on the effectiveness of a standardized positive education training program in Austria
Source: Front Psychol. 2025 Apr 8;16:1516572. doi: 10.3389/fpsyg.2025.1516572 (PMC12053473; doi:10.3389/fpsyg.2025.1516572)
Supplement: Supplementary file 1 [file Supplementary_file_1.pdf]

Appendix:

Appendix1: Descriptive statistics and intercorrelations of the PERMA-Profiler and the SWLS for the intervention group at measurement point 1.

	N	M	*SD*	*O*	*P*	*E*	*R*	*M*	*A*	SWLS
Teacher										
Overall well-being	84	118.23	16.63	-						
P ositive Emotions	84	22.76	4.58	.87**	-					
E ngagement	84	22.39	4.62	.81**	.60**	-				
R elationships	84	24.42	4.36	.82**	.69**	.51**	-			
M eaning	84	24.98	3.87	.83**	.63**	.60**	.60**	-		
A ccomplishment	84	23.67	2.86	.74**	.58**	.53**	.53**	.60**	-	
SWLS	84	28.21	4.04	.69**	.71**	.36**	.36**	.59**	.51**	-
Students										
Overall well-being	699	112.29	19.67	-						
P ositive Emotions	699	24.24	5.06	.78**	-					
E ngagement	699	18.95	5.55	.42**	.12**	-				
R elationships	699	24.06	5.37	.78**	.60**	.12**	-			
M eaning	699	23.89	5.89	.81**	.56**	.16**	.56**	-		
A ccomplishment	699	21.14	5.92	.75**	.50**	.08*	.50**	.55**	-	
SWLS	699	20.24	3.96	.68**	.67**	.12**	.58**	.57**	.48**	-

**sig. $p < .01$, *sig. $p < .05$, two-tailed testing. Note: O= overall well-being score (i.e. average of the PERMA 15 items), P = Positive Emotions, E = Engagement, R = Positive Relationships, M = Meaning, A = Accomplishment, , SWLS = Satisfaction with Life Scale.

Appendix2: Descriptive statistics and intercorrelations of the PERMA-Profiler and the SWLS for the intervention group at measurement point 2.

	N	M	*SD*	*O*	*P*	*E*	*R*	*M*	*A*	SWLS
Teachers										
Overall well-being	68	119.60	17.03	-						
P ositive Emotions	68	23.40	4.31	.88**	-					
E ngagement	68	21.91	4.45	.76**	.58**	-				
R elationships	68	25.27	3.97	.82**	.61**	.46**	-			
M eaning	68	24.90	4.15	.94**	.84**	.62**	.72**	-		
A ccomplishment	68	24.13	3.21	.85**	.68**	.50**	.70**	.80**	-	
SWLS	68	28.34	3.82	.64**	.64**	.36**	.54**	.64**	.55**	-
Students										
Overall well-being	643	113.78	19.62	-						
P ositive Emotions	643	24.00	5.20	.81**	-					
E ngagement	643	20.00	4.93	.43**	.13**	-				
R elationships	643	23.86	5.79	.81**	.65**	.17**	-			
M eaning	643	24.57	5.83	.85**	.67**	.18**	.62**	-		
A ccomplishment	643	21.36	5.07	.72**	.46**	.18**	.45**	.55**	-	
SWLS	643	19.98	4.18	.72**	.73**	.13**	.63**	.67**	.42**	-

**sig. $p < .01$, *sig. $p < .05$, two-tailed testing. Note: O= overall well-being score (i.e. average of the PERMA 15 items), P = Positive Emotions, E = Engagement, R = Positive Relationships, M = Meaning, A = Accomplishment, , SWLS = Satisfaction with Life Scale.

Appendix 3: Descriptive statistics and intercorrelations of the PERMA-Profiler and the SWLS for the control group at measurement point 2.

	N	*M*	*SD*	*O*	*P*	*E*	*R*	*M*	*A*	SWLS
Teachers										
Overall well-being	65	121.25	14.87	-						
P ositive Emotions	65	22.89	3.94	.83**	-					
E ngagement	65	23.26	4.01	.73**	.48**	-				
R elationships	65	25.71	3.89	.84**	.59**	.57**	-			
M eaning	65	25.72	3.43	.81**	.57**	.41**	.67**	-		
A ccomplishment	65	23.66	3.45	.77**	.66**	.37**	.47**	.61**	-	
SWLS	65	28.71	4.33	.68**	.62**	.31**	.52**	.58**	.69**	-
Students										
Overall well-being	657	112.73	20.10	-						
P ositive Emotions	657	23.86	5.47	.83**	-					
E ngagement	657	19.39	5.13	.43**	.16**	-				
R elationships	657	24.06	5.59	.79**	.65**	.16**	-			
M eaning	657	24.45	5.92	.87**	.73**	.19**	.64**	-		
A ccomplishment	657	20.96	5.39	.70**	.45**	.13**	.40**	.55**	-	
SWLS	657	19.73	4.07	.77**	.74**	.19**	.67**	.71**	.48**	-

**sig. $p < .01$, *sig. $p < .05$, two-tailed testing. Note: O= overall well-being score (i.e. average of the PERMA 15 items), P = Positive Emotions, E = Engagement, R = Positive Relationships, M = Meaning, A = Accomplishment, , SWLS = Satisfaction with Life Scale.

Appendix 4: Descriptive statistics and intercorrelations of the PERMA-Profiler and the SWLS for the intervention group at measurement point 3.

	N	M	*SD*	*O*	*P*	*E*	*R*	*M*	*A*	SWLS
Teachers										
Overall well-being	66	124.56	15.49	-						
P ositive Emotions	66	24.52	3.58	.90**	-					
E ngagement	66	23.61	4.66	.82**	.62**	-				
R elationships	66	25.73	3.85	.87**	.78**	.58**	-			
M eaning	66	25.77	3.53	.87**	.76**	.62**	.71**	-		
A ccomplishment	66	24.94	2.65	.77**	.68**	.50**	.64**	.59**	-	
SWLS	66	29.53	3.54	.68**	.70**	.44**	.63**	.67**	.46**	-
Students										
Overall well-being	554	114.98	21.01	-						
P ositive Emotions	554	24.14	5.39	.85**	-					
E ngagement	554	20.07	5.19	.51**	.25**	-				
R elationships	554	24.24	5.46	.81**	.69**	.21**	-			
M eaning	554	24.99	5.75	.88**	.75**	.28**	.68**	-		
A ccomplishment	554	21.54	5.45	.78**	.58**	.25**	.52**	.65**	-	
SWLS	554	20.04	4.14	.72**	.72**	.18**	.67**	.68**	.49**	-

**sig. $p < .01$, *sig. $p < .05$, two-tailed testing. Note: O= overall well-being score (i.e. average of the PERMA 15 items), P = Positive Emotions, E = Engagement, R = Positive Relationships, M = Meaning, A = Accomplishment, , SWLS = Satisfaction with Life Scale.

Appendix 5: Descriptive statistics and intercorrelations of the PERMA-Profiler and the SWLS for the control group at measurement point 3.

	N	M	*SD*	*O*	*P*	*E*	*R*	*M*	*A*	SWLS
Teachers										
Overall well-being	65	121.97	16.17	-.						
P ositive Emotions	65	23.06	4.54	.90**	-					
E ngagement	65	23.09	4.04	.76**	.60**	-				
R elationships	65	25.85	3.89	.77**	.58**	.47**	-			
M eaning	65	25.89	3.40	.86**	.73**	.48**	.64**	-		
A ccomplishment	65	24.08	3.51	.88**	.82**	.57**	.53**	.79**	-	
SWLS	65	28.40	4.24	.76**	.72**	.42**	.63**	.68**	.71**	-
Students										
Overall well-being	657	111.79	21.00	-						
P ositive Emotions	657	23.71	5.40	.84**	-					
E ngagement	657	19.11	5.47	.47**	.20**	-				
R elationships	657	23.61	5.90	.82**	.68**	.20**	-			
M eaning	657	24.17	6.23	.86**	.72**	.20**	.66**	-		
A ccomplishment	657	21.18	5.09	.73**	.51**	.18**	.49**	.58**	-	
SWLS	657	19.60	4.20	.73**	.72**	.14**	.64**	.68**	.50**	-

**sig. $p < .01$, *sig. $p < .05$, two-tailed testing. Note: O= overall well-being score (i.e. average of the PERMA 15 items), P = Positive Emotions, E = Engagement, R = Positive Relationships, M = Meaning, A = Accomplishment, , SWLS = Satisfaction with Life Scale.
