# Supplementary material for: PERMA.teach: a study on the effectiveness of a standardized positive education training program in Austria
Source: Front Psychol. 2025 Apr 8;16:1516572. doi: 10.3389/fpsyg.2025.1516572 (PMC12053473; doi:10.3389/fpsyg.2025.1516572)
Supplement: Supplementary file 2 [file Data_Sheet_1.ZIP › Material PERMA.teach/Material Expertinnen/Interviews Expertinnen inkl. Leitfaden und Codierung.pdf]

Anhang – Übersicht

Anhang A	Interview-Leitfaden Fragebogen	S.46
Anhang B	Transkripte der Interviews	ab S.47
	Interview 1: I. Teufel	S. 47
	Interview 2: D. Andreatta	S. 62
	Interview 3: B. Hellbert	S. 76
Anhang C	Code-System/ Kategorien-Übersicht	S. 91
Anhang D	Tabelle der codierten Segmente	S. 92
Anhang E	EIDESSTATTLICHE ERKLÄRUNG	S. 125

Anhang A): Interview-Leitfaden Fragebogen

1. Was hat dich dazu bewegt am Projekt PERMA.teach teilzunehmen?
2. Welche Veränderungen in Bezug auf die 5 PERMA Faktoren hast du bei den Teilnehmer*innen wahrgenommen?
3. Hast du selbst eigene Veränderungen bei dir feststellen können? Falls ja, welche?
4. Was hat sich im Projektverlauf im Expertinnenteam verändert?
5. Worauf habt ihr bei der Konzeptionierung (Entstehung der Modulinhalte und Praxisangebote) besonderen Wert gelegt? Welche Schwerpunkte und Ziele habt ihr verfolgt?
6. Welche Schwerpunkte und Ziele waren dir dabei besonders wichtig? Wie konntet ihr euch da einigen?
7. Wie würdest du deine Rolle im Team beschreiben? Worauf kommt es dir da besonders an?
8. An welchen bisherigen empiriebasierten Inhalten bzw. pädagogischen Methoden habt ihr euch bei der Konzeptionierung noch orientiert?
9. Wie wurden die individuellen Bedürfnisse der Kinder dabei berücksichtigt?
10. Wie zufrieden auf Skala von 1-10 bist du mit der Qualität der Schulungsbausteine?

    → Präsenzveranstaltung? Online-Fortbildungen? Stärken-Cafés?

    (→Was fehlt deiner Meinung nach, um auf 10 zu kommen?)
11. Wie geht es für dich jetzt weiter?
12. Was möchtest du noch sagen? Was ist dir dazu sonst noch wichtig?

Anhang B): Transkriptionen der Expertinnen-Interviews

1. Interview

mit Ingrid Teufel, am 22.06.23 um 10 Uhr

I (00:01): (Vorstellung…) ja, und das war mir immer wichtig, da immer weiter lernen und ich bin zwar immer der gleichen Schule geblieben, aber ich hab die Schule an meine Bedürfnisse und an die der Kindern angepasst. Und hab mir gedacht, Veränderungen kann man auch von innen leben, also ich bin da immer so ein statischer Mensch. Ich war 46 Jahre verheiratet, 44 Jahre an der gleichen Schule und ja, und dann hab ich einfach, weil ich es als Kind gar nicht einfach gehabt habe, aber mich selber mitm Schopf raus gezogen habe. Da hat mir halt Literatur und Lernen und immer Schnupper-Studieren und Begegnung und Arbeit mit Kindern sehr geholfen, weil das hat mir die Richtung gegeben. Und dadurch bin ich dann immer tiefer, also durch die Bedürfnisse und Bedarfe immer tiefer in das Thema rein gekommen. Weil das für mich und grad für Lehrende die absolute Basis ist und essenziell. Und ein Lehrender, der das nicht lebt, er muss es nicht wissen, er muss es leben. Aber es ist in unserer schnelllebigen Zeit, ist so viel der Fokus darauf verloren gegangen und es wird so viel…ich war jetzt in den letzten Tagen bei drei, vier verschiedenen, oder mehr, Veranstaltungen und da sind, wichtig, lauter Studien vorgestellt worden, aber erst bei der letzten ist von Kindern geredet worden. Und da denke ich mir, es ist ganz wichtig, dass man das beforscht. Aber es hat, es ist mittlerweile ein Gap zur Praxis. Weil´s zu wenig Lehrende gibt und so wenig, und das müssen wir jetzt erreichen und es ist unser Ziel, dass wir ganz viel Lehrende an Universitäten und Pädagogischen Hochschulen einschulen, dass die das in vier Stufen lernen, leben, sie selber, dann einbetten in ihren Berufsalltag und das letzte ist „teach it“, dass sie es den Kindern, den anderen erklären und warum man es macht. Und das war einfach ein Strudel und ein Bedürfnis und ich hab dann einfach auch die Schule umgebaut. In Österreich gibt es ja keine Gesamtschule und das hat mich gestört, weil ich wollte individualisieren, ich hab gehabt Inklusion, immer wichtig, Mehrstufenklasse. Und ich hab gesagt, nein, mit 10 kann ich die Kinder nicht raushauen. Die sind so unterschiedlich, ich schau, dass ich individualisiere und dann hab ich drei besonders bedürftige Burschen gehabt, die hätte ich nicht weiter schicken können. Hochbegabt, aber nicht in Deutsch und Mathe. Also, die wären gescheitert. Und dann hab ich gesagt, nein, das mache ich nicht mehr. Und hab auf Umwegen geschafft, einfach eine gemeinsame Schule, indem ich Schulen angeredet, vier, und wir haben dann von vier Schulen die Lehrer am gleichen Standort gehabt und die Kinder. Das war jetzt die BK Gesamtschule (?).

T (03:18): Okay, in Mathe und Deutsch nicht gut, wo hatten die dann ihre Stärken?

I (03:25): In Biologie, hohes Interesse, riesiges Wissen und sie studieren jetzt. Und das hätten sie nie geschafft, und sie schauen alle auf Lehramt.

T (03:45): Das war auch letztendlich das, was mich an dem Projekt so begeistert hat, weil ich oft erlebt habe, also im Rahmen von PERMA lead oder überhaupt in den letzten Jahren, wo ich Schulungen und Trainings gemacht habe, dass halt doch viele, also später Gewohnheiten zu verändern, das Mindset umtrainieren. Also es geht schon, es geht immer, gleichzeitig es leichter ist, wenn ich das von Anfang an mitnehmen kann. Grad jetzt als Kind, wenn ich da von Anfang an die Möglichkeiten habe. Also das gefällte mir richtig, richtig gut und deswegen sehe ich auch dieses Projekt als so sinnvoll an. Neben diesen Kindern aber grad auch Lehrer, und auch weil wir so einen Mangel haben. Und dabei ist es so ein wichtiger Beruf. Ich find es immer spannend, wie man das bei uns sieht und gleichzeitig wie das in z.B. Asien ist. Da ist ein Lehrer ja „wow" und bei uns eher „oh Gott, du tust dir das an", so mehr oder weniger…

I (04:55): Ja, Ja…Skandinavien ist auch anders

T (04:58): Ich glaub, auch wenn man da diesen Side-Effect, oder was heißt side-effect, ist ja kein Nebeneffekt, es ist beides wichtig. Und gleichzeitig, wenn du sagst, dieses Thema hat dich immer schon interessiert, begleitet und du hast es sinnvoll gefunden. **Was war für dich dann speziell die Motivation bei PERMA.teach mit zu machen?**

I (05:27): Also, das Thema hat mich deswegen interessiert, weil ich mich, weil etwas, also für die Kinder, meine Kinder in der Schule, weil ich gesehen habe, was sie brauchen. Und dadurch bin ich diesen Weg gegangen, das war der einfach logische. Und das PERMA.teach hab ich ja eigentlich entwickelt. Also ich hab Jahrzehnte lang unsere Hefte „be a yes" und so, die sind in der Schule entstanden, in der Arbeit mit den Kindern, aber immer im Austausch mit lernen. Ich hab dann immer geschaut, das Kind braucht das, was könnte helfen. Und dadurch sind die Sachen entstanden und meine Freundin und die Projektleiterin hat ein EU-Projekt gemacht und sie haben Sachen gebraucht. Und dann haben sie mich gefragt, und ich hab gesagt, ja, das hab ich eh am Computer. Und wir haben dann meinen Computer leer geräumt, na, der ist noch immer voll. Und sie hat das dann geordnet, weil ich hab ja einen Haufen gehabt. Und wir haben das dann nach PERMA geordnet und dann bin ich von einer Freundin gefragt worden, warum wir das nicht einreichen, bei Fond „gesundes Österreich". Und das sagt ja wieder viel, nicht das Bildungsministerium, sondern für´s Gesundheitsministerium. Und das habe ich der Eva gesagt und die hat sofort ja gesagt, ja allein, hätte ich das nie geschafft, weil das ist ja ein irrer Aufwand. Und ich bin für´s Praktische und sie hat jetzt diese ganzen formalen Sachen gemacht und ich das Praktische und das Pädagogische. Und das ist unser Kind und es ist nur leider explodiert und Gott sei Dank. Weil wir haben angefangen, wir hätten erst im Juni richtig starten sollen und sind, aber haben im Jänner schon voll losgelegt, weil wir eh schon so viel Sachen gehabt haben. Und wir wollten nur zehn Projektschulen, dann haben wir doch 19 genommen. Wir haben von 14 PHs in Österreich, 11 machen mit. Und bei 11 geben wir alle Sachen weiter, damit die dann Multiplikatoren sind. Also wenn das Projekt aus ist, wollen wir ja, dass es lebt.

T (07:42): Und wenn du jetzt sagst, so für dich persönlich, also was dich persönlich bewegt hat, weil du gesehen hast, was die Kinder brauchen…?

I (07:57): Ja, das war mein Sinn. Weil wenn ich etwas nur mache, weil´s schick ist oder weil´s…ich find, deswegen mag ich PERMA auch so und vertiefe mich immer, komm immer mehr auf den Grund von der Bedeutung vom Sinn. Und der ist ja die beste Burnout-Prävention. Wenn ich weiß, wofür ich es mache, brenn ich nicht so leicht aus. Aber wenn ich sage, ich geh ja in die Schule, weil ich da wenig Stunden hab, na, dann soll ich noch Verwaltung machen, ja da brennt man schnell aus, oder steigt aus innerlich, und gibt den anderen die Schuld.

T (08:37): oder so, ja, das stimmt. Des geht ganz schnell…Ich bin auch Resilienz Trainerin und hab da eine Ausbildung gemacht und ja, der Faktor mit dem Sinn, der ist da richtig, richtig stark. **Aus welchem Grund habt ihr euch dann speziell für PERMA als Muster entschieden?**

I (09:00): Weil ich schon sehr lange mit PERMA gearbeitet habe. Ich hab ja auch das „be a yes“-Heft nach PERMA aufgebaut und dann war halt die Eingebung…ich hab mich früher mit der existenziellen Pädagogik, Frankl, schon damit beschäftigt. Aber das ist soo und wenig greifbar. Und das ist greifbar! Und da kannst du es in den Alltag kriegen und bist nicht überfordert. Und das erinnert dich ja ständig. Da sind wir bei „tiny habit“, das sind einfach nur: hm, hm, hm und es muss so sein, dass es kleine Veränderungen sind. Die kleinen Veränderungen, die 1 % Methode, das werden ja dann immer mehr. Aber wenn man den Lehrern sagt, du musst jetzt deinen Unterricht vollkommen umstellen. Nein, eine Kleinigkeit, positiver Beginn, eh logisch, aber man denkt nicht dran. Aber dann, wenn man das noch dazu aufhängt, am besten an einer schlechten Gewohnheit, dass man die ausradiert, dann…also mir ist so passiert, ich bin eine Zeit lang immer in jedes Geschäft rein gegangen: was brauch ich als nächstes? Man ist unachtsam…also, wenn man durch die Türe geht, dann denkt man schon an den nächsten Schritt. Und ich hab dann gemerkt, man wird dann eigentlich unhöflich. Man ist nicht da, man vergisst so viel. Und unzufrieden und wie´s dann weh getan hat, hab ich gesagt: so, jedes Mal, wenn ich durch eine Türe gehe, lächle ich. Das habe ich durchgezogen, ich mache es jetzt automatisch, es hat unglaublich viel bewirkt, das glaub man gar nicht. Eine Kleinigkeit und wenn Lehrer sich das angewöhnen, jedes Mal vor der Tür einmal A.L.I., durchatmen, lächeln, innehalten und dann rein gehen, ist das ein anderer Beginn, in Ruhe.

T (11:04): und jedem Anfang wohnt ein Zauber inne…

I (11:09): Ja, und darum geht’s mir. Das in den Alltag bringen, einfach, aber nicht trivial. Weil, wir haben ja alles wissenschaftlich belegt.

T (11:19): Ja, das ist definitiv auch eine schöne Sache, dass das eben auch belegbar ist. Du und gleichzeitig, wann ist dir das PERMA-Modell zum ersten Mal untergekommen?

I (11:36): Eigentlich Seligman von Anfang an begleitet. Ich hab jetzt gefunden ein Schulkonzept von mir, das war 2005, da hab ich mitm „happynet“ gearbeitet, auch auf der Basis Positive Psychologie

und die ganzen Sachen. Und dann sehr bald, hab ich das PERMA eingeführt, das war so, also der Seligman hats 2011, also da ist das Buch rausgekommen und 2012 hab ich das in mein Klassenkonzept ergänzt gehabt. Also ich hab, Klassenkonzept war schon mit PERMA.

T(12:12): Ja, cool! Du hast das ja schon sehr früh gelebt. Seitdem das Projekt jetzt aktuell läuft, das ist ja jetzt im zweiten Jahr offiziell, und inwieweit hast du vielleicht schon beobachten können, dass die, die bisher daran teilnehmen, z.B. die Pädagogen usw. **Würdest du sagen, ihr konntet bei den TeilnehmerInnen, weil sie sich ja mehr mit PERMA beschäftigen, damit arbeiten, schon irgendwelche Veränderungen feststellen?**

I (12:54): Also wir haben sehr positive Rückmeldungen und es ist bei Machen schon eine Erleichterung, weil sie waren ja schon am Weg. Aber das ist Manche. Und das ist dann schon eine Bestärkung, dass man das macht. Weil im Alltag, wo geraunzt und gejammert wird, kommt man leicht zu diesem tipping-point und einer Abwärtsspirale. Und dass sie sich da selber wieder raufdrehen können. Also das auf jeden Fall. Ich arbeite auch für die „Schule im Aufbruch". Ja, a bissl viel arbeite ich…und da ist uns absolut bewusst, dass wir grad im mittleren Bereich wirksam sein können. Also man sagt ja bei Unternehmen, ein Drittel ist eh schon weit vorn, und das gibt's, die sind Naturtalente. Oder die gehen in die Richtung. Ein Drittel weiß nicht wohin, und die sind sehr leicht beinflussbar. Und ein Drittel, auf die vergessen wir jetzt mal, weil sonst machen wir uns da fertig. Klingt fürchterlich, aber wenn man realistisch arbeiten will, muss man sich auf die konzentrieren, die man noch beeinflussen kann. Und wenn man dann andere, also bei der Schule im Aufbruch ist das unser Motto, weil dann holen wir einige, also die ins Boot und die restlichen können eventuell mitschwimmen. Weil die sehen, dass das erfolgreich ist und nehmen sich vielleicht ein Beispiel.

T (14:30): Mit gutem Vorbild voran…kann ja auch ansteckend wirken. Okay, du hast vorhin gesagt, da kann man beobachten es geht leichter. **Hast du mir da ein Beispiel dafür? Was geht für die leichter? Welche positiven Veränderungen, seit sich die TeilnehmerInnen damit beschäftigen?**

I (14:56): Also die, die sich zurückmelden, es bringt so viel, sie sehen das jetzt anders, sie gehen anders an die Sache heran, sie denken immer wieder dran. Und das ist natürlich toll. Das man es einfach in ihr Leben bringt. Und ich das Gefühl, das Wichtigste ist, dass sie es wirklich leben. Dann rutsch es eh ins berufliche Leben. Aber wenn wir Ihnen nur die Sachen sagen, sie bekommen natürlich auch Tricks, also positiver Beginn, die Interventionen kennst du eh alle. Alle Positiven, dass man die individualisiert und auf ihr eigene Weise und dann reinholt. Und ich weiß, ich hab letztens ein Seminar gehalten, so ein Wochenend-Seminar, und das war wirklich herausfordernd, da waren Kindergärtnerinnen bis Oberstufenlehrer. Es waren lauter Frauen natürlich. Und da hab ich mir gedacht, ja super, PERMA kennen sie nicht und an einem Nachmittag, äh puh…und dann hab ich sie umgedreht, ich hab ihnen einfach die PERMA-Kurzbeschreibungen der Säulen gegeben. Ich so

einen Spaziergang dann gemacht für Seminare. Die werden dann aufgelegt oder aufgehängt und sie gehen in Gruppen und reden zu den einzelnen Säulen.

T (16:25): so wie das World-Café?

I (16:32): Und dann hab ich sie einfach berichten lassen, worauf sie im Gespräch gekommen sind. Und ich hab natürlich vorbereitet gehabt, zu jeder Säule Impulse, Hintergründe, und bin aber dann auf ihre Aussagen eingegangen und das PERMA hat das Ganze gebündelt, einen Faden darum gesponnen. Weil wir haben das PERMA dann gefüllt und sie haben das Gefühl gehabt, sie leben es schon und haben von den anderen gelernt. Und ich hab auch wieder gelernt. Also, ich denke mir, es ist überall so gut möglich, ich bin so begeistert. Ich werd immer wieder gefragt, für verschiedene Seminare, das letzte Mal war Feedback-Kultur, gelingendes Feedback. Ich habs auf PERMA aufgehängt. Das, sagen die Leute immer, ist nach vier Stunden immer viel zu kurz, und wo kann man mehr und ich hörs gern noch einmal. Und das PERMA gibt so eine Sicherheit, wie Bausteine, und du kannst es bei jedem Thema, kannst du es, kannst du PERMA ineinander verschachteln. Ich kenn eine Architektin, die macht Innenarchitektur nach PERMA.

T (17:52): ui, spannend.

I (17:54): Ja, sie hat eine Feng-Shui-Ausbildung und die hängt sie auf PERMA auf. Und machts genau nach diesen Schritten.

T (18:03): Eine schöne Überlegung und ich find auch, dass die 5 Säulen ja so viel Spielraum geben und lassen, dass jeder für sich da drin was finden kann. Und umso schöner, wenn du sagst, dass eben die, die sich damit beschäftigen, merken, dass es irgendwie leichter geht dadurch. Und manche Dinge schöner werden. Du hast mir vorhin auch schon so eine schöne Kleinigkeit genannt, einfach dir vorzunehmen, bevor ich durch eine Tür gehe zu lächeln. Würdest du sagen, seit ihr, du und Eva, das PERMA.teach gestartet habt, also euer Baby…**was hat sich seitdem bei dir vielleicht auch nochmal verändert? Kam da noch was dazu? Hast du bei dir selbst positive Veränderungen feststellen können? Wenn ja, welche?**

I (19:04): haha, ja immer mehr Arbeit, aber ich bin resilient. Ich sehe den Sinn drinnen und ich bin viel gelassener. Weil, ich hab ja auch Achtsamkeit gemacht für Schulen. Und da sehe ich halt immer mehr, diese Verzahnung drin, also diese Haltung der Achtsamkeit. Vertrauen, Loslassen, Anfängergeist, gehört in die Schule. Also nicht diese Vorurteile und ich sehe immer mehr diese Zusammenhänge und ich glaube, ich kanns auch immer besser auf den Punkt bringen. Also ich bin, ich kanns jetzt immer kürzer rüber bringen und das ist eine positive Veränderung. Weil da muss man erst mal so richtig in der Materie sein, damit man es dann ganz kurz machen kann.

T (20:04): Ja, verstehe, ich glaub auch, dass wenn man da eigen Erfahrungen machen kann und vor allem, wenn man da positive macht, wie du sagst: gelassener werden, entspannter, und voll gut. Du, jetzt aber von den Teilnehmerinnen, von dir, zum Expertinnen Team. Ist ja dann ein bisschen

angewachsen, neben dir und der Eva. **Wie würdest du das beurteilen, wie hat sich da im Verlauf der letzten ein, zwei Jahre die Arbeit mit dem PERMA Projekt, wie hat sich das über die Zeit aufs Team ausgewirkt?**

I (20:50): Es war total spannend, weil es waren Leute, die wir gekannt haben, dass sie die Expertise haben. Und wir haben permanent voneinander gelernt. Also, ich war bei jeder Fortbildung dabei und hab mit den verschiedenen, damit wir dann Multiplikatorinnen haben in den Bundesländern. Und es war wirklich ein schöner Prozess. Und, also es hat, mehr Kraft dem Ganzen noch gegeben. Und das ist so eine Bestätigung und wir sind so unterschiedlich und das find ich toll. Und was draus erwächst, also unglaublich. Und das Schöne ist, es breitet sich immer mehr aus. Das ist, wie wenn du einen Stein ins Wasser wirfst und die Wellen und es geht dann auch dort hin, in die Institution, in die Institution. Und es, ich weiß nimmer, wie ich meinen Tag einteilen soll. Weil so viel aufeinander trifft, das PERMA bedarf. Und es ist so ansteckend und ich hab das Gefühl, dass gerade weil man positiv ist, kommen die Leute, hat man bessere Verbindungen zu anderen und es kommen immer mehr. Und, es entsteht jetzt eine Plattform „Yippie“ zur Positiven Psychologie, wo alle Bereiche drinnen sind, Positive Leadership, Positive Planet, also Umweltschutz, positiver Journalismus. Des wird also ein großes Netzwerk, es ist schon da, aber noch nicht öffentlich, ich kann dich gern einladen, wenn es dich interessiert…

T (22:43): ja, voll gerne, Yippie!

I (22:46): ja, und das ist einfach, es findet so viel zu einander: Positive Planet, positive Schule, Kinder ermutigen, was für die Umwelt tun. Also, es gibt so viel Kraft, Positive Leadership, Lehrer zu besseren Führern…also einfach, dass sie besser die Klasse oder ihr Leben führen. Und das man dann auch nicht nur auf die negativen Nachrichten schaut, dass man da fastet und dass man schaut und sich konzentriert auf die positiven. Und bei positiv Planet ist zum Beispiel eine Freundin dabei, die hat die Mutmacherei, ein geniales Netzwerk. Und da findest du, das ist vorwiegend für positive Umweltentwicklungen gepaart mit positiver Psychologie. Wir lernen ständig voneinander, nur es wird immer mehr. Und jetzt muss ich den Fokus finden, aber es gibt keinen Fokus, weil es gehört alles zam.

T (23:59) ja, das stimmt, mit offenen Augen weitergehen. Auf der einen Seite habt ihr bei der Konzeptionierung einen Fokus gesetzt, PERMA. **Und auf was habt ihr bei der Konzeptionierung als Expertinnen wert gelegt? Was waren da eure Schwerpunkte und Ziele?**

I (24:24): Also mein Schwerpunkt, durch das, dass ich ja nach wie vor viel mit Lehrern arbeite, mit Studierenden und Lehrenden in der Fort- und Ausbildung, kenn ich natürlich die Situation der Lehrenden. Und wie kriegen wir es zu den Kindern, über die Lehrenden? Also war der Fokus einmal die Lehrenden, also dass die Lehrenden, dass ihnen besser geht, dass sie dieses Handwerkszeug lernen. Hauptziel sind natürlich die Kinder und die Veränderung. Aber das geht nur über Lehrende. Und mir war bewusst, es ist, sie haben keine Zeit, es ist überborderdende Bürokratie, es gibt immer

weniger Lehrer. Es gibt Leute im Lehrberuf, die die anderen immer dermaßen anstecken, da sind wir beim mittleren Drittel und das letzte Drittel. Und die sind so frustriert und ansteckend. Junge Lehrer, die mit Begeisterung in eine Schule gehen, sind nach vier Wochen sozialisiert von solchen Typen. Und da so ein Gegengewicht zu schaffen, eine positive Sozialisierung. Und in der Hoffnung, dass sie auch andere anstecken bzw. dass sie nicht ansteckbar sind, von den Negativ-Typen. Also eine Pille, ein Aspro gegen Mieselsüchtige.

T (25:56): Cool, ab wann gibt es das in der Apotheke?

I (26:02): Das ist die Haltung und das müssen wir im Hirn selber erzeugen. Weil jeder Gedanke macht aus dem Gehirn eine Chemiefabrik und das müssen die Leute kapieren. Sie sind verantwortlich für ihre Gedanken und Gefühle und natürlich geht's einem manchmal nicht gut. Und die PoPsy will ja gar nicht, dass man das schön redet, sondern was mache ich, wenns mir grad schlecht geht. Wie komm ich wieder raus, tipping-point, Aufwärtsspirale. Und das sind die Interventionen.

T (26:37): Und gerade bei diesen Interventionen, die ihr da anbietet, oder fangen wir an bei **den Praxisangeboten, die Modulinhalte, die ihr da erstellt habt. Was war euch da besonders wichtig?**

I (26:50): zunehmend die leichte Machbarkeit und die Reduzierung. Weil wir haben natürlich zuerst den Anspruch gehabt, dass alles machen, was wir vorschlagen oder zu jedem eines oder mehrere. Aber das kennen wir ja vom Neujahrsvorsatz, und bestärkt durch die tiny-habits, äh, wird's weniger. Und die Leute stressen sich ja auch, wenn sie merken, jetzt hab ich das nicht gemacht, jetzt hab ich das nicht gemacht. Und das erzeugt ein negatives Gefühl und bringt, ist kontraproduktiv. Aber sonst, super, eines hast du durchgesetzt, wow, und das ist großartig, feier das! Und ich entwickle jetzt mit den teach for Austria, da hab ich jetzt ein neues Konzept entwickelt, ich liebe die, kennst du die?

T (27:51): Ja, ich hab schönerweise vor sieben, acht Jahren, wie ich mich entschieden habe in diese Trainer-Erwachsenenbranche zu gehen, den Reingruber Bernhard kennengelernt. Und der hat damals teach for Austria mit aufgebaut. Der ist aber nicht mehr dabei, der beschäftigt sich jetzt viel mit radical honesty.

I (28:29): was ist das?

T (28:31): Auch eine Herangehensweise in der Kommunikation, wo radical honesty, also Ehrlichkeit und Gefühle ansprechen (…)

I (30:02): Gedankenänderung und da sind wir bei der Achtsamkeit, Anfängergeist, der Frankl hat das so schön formuliert. Ich hab das so aufbereitet, den Sinn nach Frankl. Und der hat eben, es kommt von außen was Negatives und du reagierst negativ. Und das ist seine einzige Entscheidung und das war im Konzentrationslager. Die einzige frei Entscheidung, war seine Reaktion und sein Denken.

T (30:30): die WIDEG-Frage…

I (30:33): Ja, und das bewirkt dann wieder eine Reaktion, und die Quantenphysik, also die Energie folgt der Aufmerksamkeit.

T (30:46): Stimmt, „where focus goes, energy flows"…

I (30:51): So ist es

T (30:53): Du, und gleichzeitig, wie würdest denn du, weil ich vorhin gefragt habe, worauf ihr da im Team bei der Konzeptionierung Wert gelegt habt, **wie habt ihr euch da einigen können, weil jeder hat natürlich seine Punkte, wie war das, als ihr das Projekt aufgestellt habt. Ja, wie konntet ihr euch da einigen?**

I (31:23): Also, es war überhaupt keine Frage, das ist passiert, es war ein Fluss. Also, es hat ja das Konzept gegeben. Jeder hat dann, also wir haben dann geschaut, dass wir das runter brechen auf einzelne machbare Schritte. Also, wie können wir es den Lehrern umhängen, quasi. Dass es nicht zu viel ist, aber doch nicht zu wenig, und das haben wir sehr schnell, also das war eh schon vorgegeben. Das haben wir dann sehr schnell in Schritte gepackt, eben dieses 4-Schritte Modell: learn it, live it, embed it, teach it. Dann hat unser junge Sonja das designed, also jeder hat seine Stärken eingebracht. Die Bianka hat sofort Hand-outs gemacht in ihrer Struktur, die super hilfreich sind. Das ist ihre Stärke. Und so haben wir eben, durch diese Stärken zulassen, wie eben in der Schule auch machen sollte. So sind wir miteinander stärker geworden. Das Wertschätzen der Stärken…

T (32:31): **Und wie hast du speziell in der Anfangsphase/ Konzeptionierung deine eigene Rolle empfunden?**

I (32:40): Als Mitarbeiterin.

T (32:42): als Mitarbeiterin?

I (32:45): Ja, ich muss dazu sagen, ich hab auch diese Schule immer als Kollegin ins Leben gerufen. Und ich wollt nicht Direktorin sein. Ich wollt auch nicht an die PH, ich hab mir die Studenten hergeholt und die Lehrer. Das war mein Kerngeschäft und da war ich auch die Teampartnerin. Und hab aber als Teampartnerin, merk ich jetzt, schon sehr die Richtung vorgegeben, indem ich halt viel Arbeit gemacht habe, Vorarbeit. Und dann mit den Kollegen gelebt. Aber es muss einer da sein, der das auch mal tut.

T (33:21): Stimmt, jemand, der anfängt… Schön, als Mitarbeiterin also. **Worauf ist es denn dir persönlich in dieser Zusammenarbeit besonders angekommen?**

I (33:41): Auf Wertschätzung.

T (33:43): Wie zeigt sich die für dich?

I (33:46): Dass man für alles offen ist. Und…aber eh die Wertschätzung war eh da, es waren eh alle super. Also ich kann gar nichts sagen, es ist einfach mein normaler Arbeitsprozess gewesen, immer

mit Mitarbeitern, also, mit meinen Kollegen. Und dass das in Harmonie abläuft, aber dass auch der Raum sein muss, dass jeder seine Meinung sagt. Und es ist ja nichts negatives, wenn einer eine andere Meinung hat, das kann ja sehr pro…also, in den meisten Fällen sogar ein Fortschritt.

T (34:28): Ja, so kann man das sehen. Definitiv…voll gut. Du, gab´s aber dann auch während diesem Prozess, klar PERMA war von Anfang an klar, **an welchen sonstigen bisherigen Empirie basierten Inhalten und pädagogischen Methoden. An was habt ihr euch da noch orientiert? Gabs da noch was?**

I (35:02): Es war immer, welche Bedarfe, Bedürfnisse hats gegeben. Es ist immer stärker die Achtsamkeit dazu gekommen. Man könnte es auch a bissl mit PERMA health, also des H, Bewegung, also ich hab auch ganzheitlich Lernen lernen gemacht für uns. Wo´s die vier K in Anwendung gibt, also Kommunikation, Kreativität, usw.

T (35:27): die sind super, die 4 Ks, die haben mich persönlich sehr abgeholt, aber darum geht's ja jetzt nicht…

I (35:34): Ja, es gibt von mir auch ganzheitlich lernen im 21. Jahrhundert, das hab ich ganz auf den 4 Ks aufgebaut, als Lehrmethoden, aber mit der Eva natürlich. Weil die hat, ohne Eva gäb´s das nicht. Ja, ähm, wir sind immer mehr zum Reduzieren gekommen. Und was ich schon von Anfang an, Gott sei Dank, konzipiert habe, dass wir nicht mit Rezepten arbeiten, sondern mit Leitfragen. Damit sich jeder selber anleiten kann, weil jeder hat eine eigene Situation, momentan, überhaupt, wo, mit wem,…und das geht nur mit Leitfragen. Und die Leitfragen sind auch flexibel. Dass sie lernen sich selber zu fragen: Ja, Relationship? Was hab ich heute dafür getan? In der Schule hab ich den Kindern das vorgelebt, hab ihnen Methoden gezeigt. Und es war mir immer wichtig, weil, als Frankianerin, dass das nicht bei der Self-Compassion aufhören darf. Also es darf bei dem Projekt nicht sein, wie geht's mir bei Beziehungen, wie hab ich mehr bessere Beziehungen, sondern immer das an…vom ich zum wir. Und da bin ich ganz, ganz stur. Also das ist mir ganz wichtig, dass man da nicht stehen bleibt. Also, wie komm ich zu mehr positiven Emotionen? Wie kann ich auch anderen mehr positive Emotionen verschaffen? Absolut der nächste Schritt. Das war vielleicht das einzige, wo ich mit unserem Team, mit manchen, na, keine Probleme, wo ich nicht ganz überein gestimmt bin oder habe. Die Überbetonung der Self-Compassion. Das war so die Modezeit, ich achte auf mich und ich hab eine Kollegin gehabt, die super war, aber: „wenn´s mir gut geht, geht's den anderen gut." Und dann ist ein 6-jähriger gekommen und hat irgendein Problem. „Nein, das ist deine Sache. Da grenz ich mich doch ab." Und das, und das hat mich vielleicht geprägt, dass ich gesagt habe, ja, erster Schritt ist sich selbst kennen und auf sich achten, ganz wichtig. Aber da darf es nicht aufhören. Auch wie der Frankl sagt, Sinn ist nur, wenn man was für etwas oder für jemand tut, also im Austausch mit jemand oder etwas. Ist der Sinn nur für einen selber, dann ist es kein höherer Sinn. Und das war halt das Einzige, wo ich dann, wie sagt man, ein bisschen reguliert habe.

T (38:23): Für dich, in deinem Tun reguliert hast?

I (28:25): Auch im Team, hab ich das schon gesagt.

T (38:30): Du, jetzt hast du vorhin schon die Leitfäden angesprochen, weil uns auch noch interessiert hätte, im Laufe dieser Konzeptionierung, klar, das ist jetzt ein Modell, dass ganz viele Menschen abholen soll. Und du sagst eh, es wächst unglaublich. Aber nochmal auf den Anfang geschaut, **inwieweit habt ihr diese ganzen individuellen Bedürfnisse, was ja auch grad bei den Kindern da sind, wie hat man das berücksichtigen können, in der Entwicklungsphase? Auf was habt ihr da besonders geachtet, dass das möglich ist?**

I (39:19): Also, das war dieser schwierige Spagat. Wir hatten ja fixe Power-Points, die dann so gültig sind. Und ich, das ist mein Problem gewesen, weil immer wenn ich mit einer Gruppe arbeite, dann bereite ich mich vorher vor, eigentlich neu. Und dann sind neue Aspekte und so. Bei den Pilotschulen wars okay, weil da haben wir gewusst, das ist jetzt eine MS oder Volksschule. Da ist das leicht. Wir haben aber viele Botschafter-Ausbildungen gemacht, wo´s quer durch war. Und da muss man halt natürlich noch mehr reduzieren. Aufblühen tu ich, wenn ich eine konkrete Gruppe habe, weil da kann ich total auf die Bedürfnisse eingehen. Ich habs z.B. gemacht für Lesepaten und da hab ich zur Eva gesagt, nein, ich nehm nicht unsere Folien, weil die duften wir nicht hergeben. Ich konnte mir eigene machen und hab aber das PERMA ganz speziell auf Lesepaten, die keine Ahnung von der Pädagogik haben, die eingeschult. Und da hab ich erst gemerkt, was für ein tolles Modell das ist. Du kannst es wirklich so bedürfnisgerecht machen. Und mein Ziel ist es, dass die Leute lernen, diese Fragen sich selber zu stellen, eigene.

T (40:50): Worauf hast du geachtet, also wenn du sagst bedürfnisgerecht? Also nach Zielgruppe? Wie hast du da differenziert? Lesegruppe?

I (41:02): Also ich hab zielgerecht bei den positiven Interventionen natürlich was zum Lesen gegeben. Wie kann ich das im Leseunterricht einbauen und hab da praktisch gearbeitet und hab das rein gebracht. Weil für das Ministerium bin ich in der Lese Soko. Da hab ich es auch geschafft, an einem ganzen Nachmittag diese Leseförderung, also wir sind vier, und die müssen alle schulen. Und mein Schwerpunkt war: Lesefreude. Und das habe ich über PERMA gemacht, weil das PERMA ist so gut zum Anhalten, sonst ist so beliebig. Und positive Emotionen und da müssen wir den Wortschatz fördern und schauen, usw. Und Beziehung, ja und wie? Und in Büchern…Tandemlesen. Und das, glaube ich, ist ungemein hilfreich, weil es bringts auf den Punkt. Und ich muss sagen, da sind Rückmeldungen, die mir direkt schon peinlich sind, weil sie so gut sind.

T (42:07): Wieso? Mach mal ein Beispiel! Ein bisschen „Lustgruseln"…

I (42:12): Ja, ...beste Fortbildung forever, und so…und bitte mehr und das müssen alle kennenlernen. Und das letzte Mal hab ich´s für Freizeit Pädagogen gehalten. „Na bitte, die ganze Schule muss das hören, die Eltern müssen auch teilnehmen dürfen.

T (42:32): Schön, wenn die Nachfrage da ist und schön, wenn dann auch so ein gutes Feedback währenddessen kommt.

I (42:39): Ja, aber es macht mich auch ein bissl traurig, wenn man sieht, wie man´s braucht.

T (42:46): Wie meinst, es macht dich traurig?

I (42:49): weil es die Leute brauchen. Eigentlich ist es eh ein Alltagswissen, es ist ein Alltagswissen, aber wir haben drauf vergessen.

T (42:59): Ja, du hast vorhin bei dir auch mal den Leidensdruck erwähnt, ganz zu Beginn. Und dass es den vielleicht, bei dem ein oder anderen braucht, dass da dann mal in die Richtung was passiert. Obwohl es schon lange ist…

I (43:20): Ich denk mir, das ist das Gute an Corona. In den Schulen ist endlich gscheit digitalisiert worden. Die Leute schauen…also, alle Studien, es geht eigentlich um die psychosoziale Gesundheit. Und jetzt gibt's so Jugendstudien. Das macht nicht das Ministerium, das machen Stiftungen. Die befragen Jugendliche und Eltern, was sie brauchen. Und überall ist die psychosoziale Gesundheit, also mehr achten auf psychosoziale Gesundheit ist überall, steht überall oben drüber. Und ist das nicht gut, dass Corona das so deutlich gemacht hat.

T (44:09): Ja, würdest du, also das ist jetzt keine Frage, aber wenn wir schon dabei sind, würdest du dann sagen, dass Corona dieses Projekt mitbefeuert hat, unterstützt hat?

I (44:21): Es war ein Corona-Call, weil das Gesundheitsministerium gesehen hat, die Auswirkungen auf Schüler, auf alle Menschen, auf die Psyche und deswegen haben sie das angestoßen. Und das finde ich großartig.

T (44:37): Hm, ja. In allem Negativen findet man, wenn´s so ist auch wieder was Positives.

I (44:46): and one door opens…

T (44:49): Du, und gleichzeitig in allem Gutem kann man, äh andersrum…in allem Negativen was Gutes sehen. Und gleichzeitig würde mich auch noch eine kurze Bewertung deinerseits interessieren. Gut und schlecht soll dabei keine Kategorie sein, um das geht's gar nicht. Wie würdest du, jetzt auch nochmal rückblickend, **auf einer Skala von 1-10**, 10 ist super zufrieden, eh schon wissen, umso weiter unten eher nicht so. **Wie zufrieden bist denn du aktuell mit der Qualität der ganzen Schulungsbausteine? Also z.B., wir haben uns da drei rausgesucht, wenns z.B. um die Präsenzveranstaltungen geht?**

I (45:36): Also mit Präsenz meinst du in den Schulen?

T (45:39): ja, genau…also da im Raustragen…

I (45:44): Also im Prinzip waren die großartig. Es hat einen Ausreißer gegeben. Da war im Vorfeld, da waren die Lehrer überlastet. Und sie haben, da war die Direktorin nicht sehr stark. Sie wollt halt

das Prestige. Und es war Schulanfang, relativ, sie haben schon die dritte SCHILF gehabt und es war der erste schöne Freitag, sie wollten raus. Und es war, ein zwei Lehrerinnen, die total laut am Anfang „Nein" gesagt haben. Und das war dann und dann hat die Bianka, die die vorherige Schulung auch dort gemacht hat. Also, sie hat die vorherige gemacht, aber zum Wohlbefinden von Lehrern. Und dann hat sie das total umgestellt, super, dass sie das gemacht hat, unser Konzept. Und dann ist es aber trotzdem in die falsche Richtung gegangen. Sie wollten viel mehr Übungen machen. Also das war dann so eine spontane Geschichte. Und das war, dass man sich verunsichern hat lassen, und ich weiß, das klingt jetzt sehr blöd, des ist auch mein Problem meiner Teamfähigkeit. Äh, ja…

T (47:14): was meinst du damit?

I (47:16): Ich hätte eigentlich meins durchsetzen müssen. Ich hab aber auch nicht wirklich gewusst, was mach ich jetzt noch, aber ich weiß von meinen anderen SCHILFS, also schulinterne Fortbildungen, sitzen immer wieder Leute drinnen, und das ist bei mir fast ein Sport, die Leute zu knacken. Und die einmal zu kriegen. Und da waren wir eigentlich kontraproduktiv. Und wir haben nachher gesagt, wir hätten nach den ersten Äußerungen sagen sollen, wir verstehen euch, es ist so schön draußen, ihr seid müde. Wer will kann da bleiben, die anderen sollen gehen.

T (47:59): Okay, das wär so das learning daraus, wo ihr dann im Nachhinein anders gemacht hättet?

I (48:07): Ja, also nicht mehr zwingen. Also diese, den Direktoren sagen, sie dürfen nicht überfordern und nicht zwingen, nur aus Prestigegründen.

T (48:18): Mhm ja, das heißt aber, dass neben dieser Erkenntnis, wo ihr euch ja vielleicht auch was mitgenommen habt. Eben grundsätzlich aber die Frage, **wie zufrieden warst du mit den Veranstaltungen, mit dem Aufbau und wie das sonst geplant war? Gerne nochmal anhand der Skala: 1-10? Wo würdest du es ansiedeln?**

I (48:44): Also von den Feedbacks der anderen Veranstaltungen kann man sagen, die waren 9-10, und das war der Ausreißer.

T (48:55): Okay, also top,top…ihr habt ja nicht nur die Präsenzveranstaltungen gehabt, zum einen ging ja dann **online** weiter im Anschluss. **Da dieselbe Frage, wie zufrieden bist du da? Gerne anhand dieser Skala…wie würdest du da die Qualität einschätzen von deiner Seite her?**

I (49:21): Also wir waren immer sehr gut vorbereitet. Also, ich hab ja immer mit anderen gearbeitet und hab die anderen aussuchen lassen, welche Punkte sie machen, damit sie dazu stehen. Ob sie Relationship machen oder so…

T (49:38): das ist ja euer Kriterium, soweit ich weiß, immer im Tandem aufzutreten?

I (49:42): genau, und sie dürfen…ich hab nicht gesagt, was ich mache, sondern ich habs die anderen entscheiden lassen, weil ich das wichtig finde. Und, das habe ich super gefunden. Mich persönlich hat natürlich gestört, dass ich nicht jeden sehen hab können. Ich hab zwar, man spürt ja Resonanz,

das geht schon. Aber so das Gefühl, erreiche ich wirklich jeden? Ich mein, die Rückmeldungen waren großartig, aber da habe ich diesen allgemeinen Anspruch halt. Statt dass man halt, was ich vorhin erzählt habe, dankbar ist, dass man nicht alle erreichen kann. Aber ich will wissen, ob ich, ich glaub schon, dass wir alle erreichen haben können. Weil sind ja freiwillig dort gesessen.

T (50:38): Ja, hoffentlich… haben wir eh schon gesagt, die Freiwilligkeit ist so wichtig, das haben wir vorhin auch schon festgestellt. Auf der anderen Seite, **würdest du da jetzt im Nachhinein was anders machen? Irgendwas verbessern in diesem online Kontext?**

I (50:53): Ja, ich würd noch mehr reduzieren. Ich würde mehr und kürzer machen. Und immer mit Reflexionsphasen dazwischen.

T (51:07): Also mehr, sprich häufigere Treffen, die aber dann zeitlich eher kürzer wären?

I (51:14): genau. Also, dass man dann sagt, pro Säule 1 bis 1,5 Stunden. Dann einen kleinen Arbeitsauftrag. Dann hat man zwei Wochen, drei Wochen, vier Wochen Zeit, dass sie am Ende der einen Stunde wirklich verbindlich, möglichst verbindelich, aufschreiben, was sie ausprobieren, eines. Eines, und das dann reflektieren. Und dann könnte man es ja auch sehr schnell, wenn es die gleiche Gruppe ist, unkompliziert mit Stempel machen. Wie warst du mit dir, mit dem zufrieden? Und dann können sie anonym auf so einer 1-10ner Linie stempeln und man hat einen Überblick.

T (52:00): Okay, also gleich mit visualisieren?

I (52:02): ja.

T (52:04): Okay, auch spannend…Ähm, dann gab´s neben den Online Treffen noch **die Stärken Café Variante. Wie beurteilst du das jetzt so mittlerweile?**

I (52:19): Also meine Kollegen haben das großartig gemacht. Es ist nicht ganz mein Format. Das haben zwei dann gemacht, die eine ist Coach, großartig. Da kann ich lernen davon. Ja ,mittlerweile kann ich es mir vorstellen, mittlerweile ist es auch mein Format, weil ich machs für die teach for Austria jetzt. Weil die sich das wünschen. Also das ist jetzt ein ganz en neues Konzept, wo das eine selbstregulierende Sache ist. Wo die Leute, sie dürfen, aber müssen nicht PERMA kennen, dass sie an mich Fragen stellen. Also über Padlet. Wünsche…ich schau dann, was ich mache über PERMA beantworte, bisschen wie Supervision. Und im Padlet ist auch ein Punkt, wo sie ihre Highlights und learnings rein schreiben. Und das kann ich von den teach for Austria erwarten, das erwarte ich nicht von jedem Lehrer. Aber das würde ich mir wünschen, dass jeder, und da ist ein Padlet großartig, leicht zu bedienen, man sieht es gleich. Also ich würde nicht, man sieht das ja bei der Eva, die Eva hat ein Unmenge an Post und Telefonaten gehabt. Und ich bin dann einmal im Zug neben ihr gesessen und hab immer geglaubt die schreib sehr schnell, und das geht runter. Ich hab gesehen, wie lang sie für eine Mail gebraucht, das Formulieren, also, perfekt. Aber endlose Zeit, also für die Eva war das und sie ist, ich bin lockerer. Ist mir ein Fehler passiert, okay, ist so. Und die Eva, die will das perfekt machen. Und da sind wir wieder: good is good enough. Da bin ich gut drauf…

T (54:11): ja, manchmal reicht Pareto.

I (54:15): Ja und das sind halt die learnings. Dass ich das jetzt anders, also es war im Prinzip genau richtig, wie es war. Aber wenn ich es noch einmal aufsetzen würde, dann würde ich es noch einmal häppchenweise machen, aber aufgrund der Erfahrungen, wie es den Leuten jetzt geht.

T (54:38): Okay, spannend…und gleichzeitig genau das, was du ja gesagt hast, bei euch ist es ja ein Weg und je nachdem, sag ich, wie es da auf diesem Weg weiter geht. Das ist eh auch die Frage, **wie geht's denn für dich da weiter, Ingrid? Wie siehst du deinen Weg noch innerhalb des PERMA.teach Projekts?**

I (55:03): Also die Eva hat jetzt durchgesetzt, dass im IFTE Verein einmal im Jahr nochmal so Kurse gibt. Da mach ich natürlich mit, aber ich hab auch gesagt über Yippie, übern Kindergarten von mir. Und die proben grad, des is die Musik, find ich super

T (55:20): Hab ich da auf deinem T-Shirt auch schon Yippie stehen sehen?

I (55:25): Ja, wir haben zwei Tage bei einem Straßenfest Yippie vorgestellt. Und waren X Leute, die die verschiedenen Facetten von Yippie vorgestellt haben. Ein junges Mädel hat gemacht Accomplishment, Lesen mit Hund, also die ganzen Sachen haben wir vorgestellt. Und da ist jetzt natürlich dieser Weg, dass ich auch über Yippie, dass wir da was anbieten. Eben stell ich mir vor, so halbstündige zu jedem Buchstaben, so Videos, die man sich immer anschauen kann und sich nachher trifft und darüber redet. Weil ich find das einfach wichtig, diesen Weg und das darf man, das möchte ich nicht aufhören. Und die Yippies wollen das auch nicht. Und ich werde immer wieder von Schulen gefragt, teach for Austria ist ein großer Punkt. Schulentwicklung, ich begleite momentan drei Schulen. Ein neues Konzept entwickeln, … also es geht weiter, es fließt, es wird sich verändern. Es ist halt dann, wie passt es wo rein, aber ich bin überzeugt, dass diese Verkürzung oder diese…wir reichen ihnen die Hand zum Anhalten. Ja, man kann sich anhalten, man kanns dem anderen reichen.

T (56:57): aber nehmen muss er es selber…

I (57:00): jeder ist seines Glückes Schmied und aufzwingen kann man es nicht. Ja, und ich denke mir, da gibt es viel Arbeit, viel zu tun. Also, wie ich es jetzt wieder bei diesen ganzen Panik-Veranstaltungen der Wissenschaft, Politik, usw. erleb. Dauernd gibt's irgendwelche Studienpräsentationen und was machen wir, was tun wir,…und in der industriellen Vereinigung war ich gestern, da haben sie eine eigene Innovationsfrau jetzt, wie man Innovation in die Schulen bringt. Und da geht's schon wieder von verkehrt los, von oben runter, des, des, des machen. Statt, ich hab gesagt, nein, wir brauchen wieder Visionen. Wir müssen bei den Leuten Visionen wecken. Wie geht's besser, Prospektion. Und auch gestern, hab ich in der Früh so einen, da sind extra zwei Professoren von der PH Oberösterreich aus Linz nach Wien kommen und wir haben am Bahnhof gearbeitet. Und sie, weil sie so verzweifelt sind, weil so viel Praxislehrer sowas von schlecht sind, so destruktiv. Wie sie das in die Lehre bringen. Zu Studierenden, sie arbeiten auch mit Studierenden. Da haben wir

dann, da hab ich ihnen ein Projekt, ein Konzept, das ich für Teach for Austria für Schüler entwickelt hab, Prospektion für Schüler, dass das Sinn macht. Soweit trau ich mir das über die PERMA Punkte und über die Achtsamkeit-Sinne Punkte, ein großes Zukunftsbild in die Gegenwart, ist dieser Schritt richtig oder nicht. Und ich hab gesagt sie müssen, also müssen, mein Vorschlag war, mit den Studierenden Visionen entwickeln wie sie in fünf, zehn Jahren in der Klasse stehen wollen. Und das nach den PERMA Punkten und nach dem Rad der Achtsamkeit von Daniel Siegel, über alle Sinne, aufschreiben, zeichnen, visualisieren, sprechen, egal, wie es ihnen taugt. Und dass sich immer wieder vorhalten und dann reflektieren, ob der Schritt jetzt in die Zukunft konstruktiv war, den sie jetzt gesetzt haben. Oder ob sie genug auf Beziehung geachtet haben.

T (59:35): Klingt echt gut …(Bedanken für Einblicke, Kennenlernen, …)

(I: Rückmeldung zum Interview zwischen Kristina und Gabi)

2. Interview

mit Doris Andreatta, am 22.06.23 um 13 Uhr

D (Vorstellung) (00:05): Kurz zusammen fassend: 30 Jahre Training und Beratung, die Firma selbstständig zusammen mit meinem Mann. Und mein Ursprung kommt, weshalb ich mich mit Schulen immer noch sehr beschäftige, weil ich selber als Volksschullehrerin tätig war und im Zuge dessen interkulturelles Learning gemacht habe. Und damals vor 30 Jahren die Hälfte der Kinder mit türkischer und die andere Hälfte der Kinder mit österreichischer Muttersprache. Wir haben in der Muttersprache alphabetisiert und ich hab mich schon vielen innovativen Lehr- und Unterrichtsmethoden beschäftigt. Und hab da eben versucht ein Konzept herauszufinden, das alphabetisiert sowohl in der Muttersprache, immer in der Muttersprache, also für die türkischen, die deutschen und österreichischen Kinder immer das gleiche, nur eben in ihrer Muttersprache. Und da hab ich dann schon viel offenen Unterricht gemacht, und muss dann immer so schmunzeln, wenn dann heut das noch immer so neu ist: ah ja, genau…und dann hab ich mich selbstständig gemacht, und hab so ein Stück immer den Zugang zu Lehrpersonen behalten. Im Sinne von Fortbildungen, so auf der Führungsebene, und das dann quer durch den Gemüsegarten. Alle Pädagoginnen, in allen Führungselementen, und in letzter Zeit, seit ich die PoPsy Ausbildung habe, das war eigentlich der Grund, weil ich mir gedacht habe, wie ich das gelesen habe, wie ich das Buch von Martin Seligman gelesen habe, habe ich mir gedacht: Maaa, das mach ich ja und das hat jetzt sogar noch einen wissenschaftlichen Hintergrund. Und dann hab ich begonnen, die Barbara Fredrickson, usw. zu lesen. Und hab immer mehr Bestätigung gekriegt und damit auch so einen guten Background. Das ist nicht etwas, was man sich so intuitiv einfallen lässt, sondern das hat Hand und Fuß.

T (01:29): Wann bist du dann auf PoPsy aufmerksam geworden?

D (02:03): Eigentlich, ich bin…eine meiner Stärken ist auch Liebe zum Lernen und ich mag eigentlich gern, also eigentlich per Zufall, dass ich mir wahrscheinlich im Zuge einer Seminarvorbereitung bin ich eben auf das Buch vom Seligman gestoßen und hab mir das dann gleich bestellt, weil das würde da jetzt gut reinpassen. Und dann hab ich eben diese Sachen gemacht und ja, jetzt lass ich es eben überall einfließen. Und das finde ich, ist einfach ein schönes Instrument. Ich bin ja auch ganz fest tätig im Bereich Qualitätsmanagement, also an Schulen und hab auch da den ganzen Prozess, ich weiß nicht, ob du das weißt, in Österreich hats SQA gegeben, jetzt gibt's QMS und in diesen Begleitungsprozessen die pädagogischen Leitvorstellungen und Entwicklungsbildern da bin ich viel in Fortbildungen und auch dass ich Schulen direkt begleite in diesen Entwicklungsprozessen.

T (03:16): Über das QMS hab ich mich auch ein bisschen eingelesen, da ich ja keinen pädagogischen Hintergrund habe. Aber schön zu hören, da war ja auch spannend, dass die Implementierungsschritte da zu PERMAchange sehr analog gehalten sind. Das macht es vielleicht den Schulen leichter es anzuwenden…

D: (03:48: Infos zu QMS von Doris)… (04:50) und die Schulen, die ich begleite, die sich schon ein Stück mit PoPsy beschäftigen, also noch ganz weit, da versuch ich das auch schon immer einzubinden. Und das schreibt ihr jetzt in den Entwicklungsplan.

T (05:09): Schön, wenn du da schon positive Beispiele erfährst und wenn ja auch schon dieser Wunsch bzw diese Gedanken in Richtung positive Bildung da sind.

D (05:22): genau

T (05:24): Ja, schön, wenn sich das auch da verknüpfen lässt, kombinieren lässt, miteinander Hand in Hand geht. Da merk ich, das Thema zieht sich ja immer schon durch bei dir…**Was war denn für dich so der Beweggrund, Doris, bei diesem PERMA.teach Projekt mitzumachen?**

D (05:52): (..lacht…) Ich hab die Ingrid und die Eva in der Überarbeitungsphase ihrer Bücher kennengelernt. Wir haben gemeinsam ein Modul zur PoPsy gemacht. Und sie sind da in der Mitte, in allen Pausen, sind sie da zusammen gesessen und haben halt die letzten Ausbesserungsarbeiten gemacht. Ich bin dann so hin und hab gefragt, was macht ihr denn da? Und bin halt in meiner Neugierde und sie haben mir das erklärt und dann hab ich mich hingesetzt und hab ihnen ein bisschen erzählt, was halt so ich mache. Und ja genau, dann sind wir da halt so in den Austausch gekommen und haben eigentlich nie den Kontakt verloren von da weg. Und wie dann Eva und Ingrid eben das PERMA.teach aufgesetzt haben, sind sie an mich ran getreten und haben eben gefragt, ob ich auch bei diesem Expertinnen Team dabei sein will. Ja und das hab ich gern und das bin ich noch gern. Wir haben uns grad gestern getroffen.

T (06:49): (…) (07:05) jetzt hast du die zwei kennengelernt und wurdest darauf aufmerksam. **Und was war so dein persönlicher Beweggrund da mitzumachen? Was hat dich speziell gereizt an dem Projekt?**

D (07:15): ähm, weils mein Thema ist, weil ich, ich möchte, das war immer schon eine Vision und jetzt ist es glaub ich, ist es schon ein handfestes Ziel geworden. Ich möchte Beiträge in der Bildungslandschaft liefern und ich möchte einfach für das stehen und jetzt sag ich ganz groß, wenn man in Österreich sagt: okay, ich such jemand, der meine Schule begleitet, dass man dann irgendwann mal auf mich stößt.

T (07:43): sehr schön

D (07:45): In Tirol… also in vielen Schulen, ich hab ja in vielen Bundesländern Schulen das Bildungsmanagement ausgenommen, bin ich tätig gewesen, also man kennt mich an den PHs schon. Und da werd ich dann immer wieder gefragt, meine Idee ist, also ich tret in manchen Sachen jetzt ein Stück weit zurück, ich bin im Jänner 60 geworden und hab so vor, und das ist diese PERMA Sache, egal ob ich es jetzt im Coaching mache oder bei Führungskräften, also ich möchte mich total auf dieses PoPsy in der Anwendung fokussieren. Und wenn Menschen sich in der Sache begleiten

lassen wollen. In der Bildung zusammen, ja, ich möchte da einen Schritt gehen, dann bin ich dir Richtige, sag ich so.

T (08:36): sehr cool, was erhoffst du dir von diesem Projekt?

D (08:43): Dass es fussfest, dass es sowas wie eine Art Selbstverständlichkeit in der größeren Breite von Bildung wird. Und es ist egal, ob das jetzt in der Elementarpädagogik, weil da ist auch was im Entstehen, also beginnend von der Elementarpädagogik bis, und schön wärs, bis in die Universitäten hinauf. Dass einfach Menschen, die Menschen etwas beibringen, etwas lehren, in diese Haltung hineingehen und so eben junge Menschen auf ihrem Bildungsweg begleiten.

T (09:15): Sehr schön, wenn das eben auch als Haltung sieht, was es ja auch ist und in der Form nicht nur eine Methode, ein Konzept, sondern wirklich eine Einstellung, eine Haltung. Voll schön.

D (09:30): ich glaub, dass das sowieso der springende Punkt ist. Es geht nur, wenn sich Menschen auf den Weg machen, selbst reflektieren und ihre Haltung überprüfen. Ich brauch, sag ich jetzt mal, ein grundsätzliche Haltung: sehe ich eher das Glas halb voll oder halb leer? Ich brauch die halb volle Perspektive, ich brauch die Perspektive, wie schau ich auf Fehler? Sag ich, das kannst du gut und das lernst du dazu, das sind Haltungssachen, wie Menschen begegnen, ja. Was sind mir meine wichtigsten Werte und Stärken? Und woran erkennen Menschen, dass sie das leben? Und wenn ich das als Pädagogin im Vordergrund habe, dann kann ich die Interventionen anwenden. Sonst sind Interventionen Hüllen.

T (10:16): Jetzt hast du eh grad schon die Pädagoginnen unter anderem angesprochen, also sprich die Teilnehmerinnen. **Hast du den Eindruck, dass jetzt rückblickend, es ist das zweite Jahr, dass bei den Teilnehmerinnen auch nur aufgrund eben dieses Auseinandersetzens, der Versuch, damit zu arbeiten in Bezug auf die PERMA Faktoren schon Veränderungen stattgefunden haben und falls ja, natürlich welche?**

D (10:51): Also ich glaub, dass es da unterschiedliche Zugänge gibt. Ich denke, dass sich einige von den Pädagoginnen gemeldet haben. Jetzt sagen wir mal so ähnlich wie ich, die eh schon ähnlich arbeiten und jetzt noch ein Rüstzeug haben. Und da ist die Voraussetzung eh da. Es haben sich sicher auch einige gemeldet, weils vielleicht ganz gut klingt. Wir sind es ja, also wir haben uns ja gestern getroffen. Wir haben das jetzt ja so ein bisserl in unserem Team, also wie die Eva und die Ingrid und die Sonja und ich haben uns da ausgedacht, weil wir das letzte Stärken-Café vorbereiten und haben eben gemeint wie, wie kriegen wir sie nur Stückchen mehr in diese Haltung. Weil manche eben sich viel zu sehr auf die Interventionen fokussieren. Und dann immer wieder kommt die Rückmeldung: Ma, wir haben nicht so viel Zeit gehabt und wir haben es nicht umsetzen können und Sie vergessen aber, dass sie durch ihre Haltung ja eh schon ganz viel täten. Und es müsste von der unbewussten Ebene in die bewusste kommen und damit kann die bewusster das PERMA.teach dann auch anwenden. Ja, und eben diesen mehr diesen Fokus in die Selbstreflexion, was kann ich

tun, was, was, was kann ich für meinen Zustand tun, damit die eben positiv vor die Klasse trete? Wie kann ich den Schülerinnen begegnen, damit sie merken, dass man jeder Einzelne, jede Einzelne ganz, ganz wichtig ist?

T (12:26): Also ich glaube, das verkörpert ja auch ein bissl diesen ersten Schritt, dieses selber Leben, der ja da auch in den Stufen dabei ist, bevor man überhaupt mal ins "teach it" geht, was ja dann letztendlich irgendwo, sage ich mal als als letzter Stepp irgendwo ja auch gesehen wird. **Und gleichzeitig gab es dann schon irgendwelche Rückmeldungen von den TeilnehmerInnen. Also abgesehen von denen, die da keine Zeit oder dass da irgendwie irgendwas geäußert wurde?**

D (13:00): Also, das sage ich jetzt wirklich so, dass eine Schule beim Stärken-Café 2, da haben wir so den großen Fokus, wie können wir mit Herausforderungen umgehen und da eben dieses Modell anwenden. Und da hat eine Schule nicht können und ich hab dann diese Schule, ist eine sehr kleine Schule in Tirol zu einem extra Termin gemacht und das ist halt auch im zoom, wenn dann sagen wir mal zehn Leuten im zoom sitzen, kann man mehr individueller austauschen, als wenn 30-40 Leute sitzen und da war auch das Thema. Und wie gesagt, ab jetzt denkt einmal nach, was habt ihr noch die Woche, was ist auch gelungen, auf was seid ihr stolz? wo sagt ihr: Hey, das war eine gute Stunde? Und das haben wir dann gesammelt. Und dann habe ich ihnen gesagt: genau, und das gehört zu E und das gehört zu R. Und wie ich ihnen das dann so wieder so zam gestückelt hab, dass Sie eh in vielen alltäglichen Dingen dann mal reden, dann ist ihnen, glaube ich so ein Knopf aufgegangen und das versuchen wir jetzt auch, dass haben wir gestern dann besprochen, dass uns das in unserem letzten Stärken-Café noch einmal vermitteln wollen, sozusagen nimm dir hin und wieder die Zeit und sag, was ist dir gut gelungen usw. um dann zu zeigen, ja genau das hat alles mit PERMA zu tun.

T (14:21): Accomplishment, auch die Schritte sehen, die Erfolge sehen. Ja du und gleichzeitig jetzt einerseits natürlich, wie du sagst, wie kommt es da natürlich an, interessiert natürlich, klar ich will da was nach außen tragen und auf der anderen Seite... gut, du kennst das Modell schon länger, wie du mir vorhin erzählt hast. Hast du bei dir selber, speziell jetzt auch mit dem, sag ich mal Fokus auch jetzt nochmal gezielter wieder auf PERMA.teach, und da aktiv werden, **und vielleicht sogar bei dir selber auch mal in puncto dieser fünf Faktoren aufgrund der Beschäftigung damit, das Auseinandersetzens, des Verbreitens auch Veränderungen bemerkt?**

D (15:06): Ausschließlich auf PERMA.teach beziehen, ge? Ich denk, dass wir alle in unserem Leben immer wieder Zeiten des Innehaltens brauchen. Und meistens tun wir es halt dann, wenn irgendwas Größeres passiert, nicht so Angenehmes passiert. Dass wir dann wieder auf unsere Ressourcen hinschauen und ich habe sehr ein prägendes Erlebnis gehabt, eben wie in dieser Corona Zeit und wir sind beide, also wir leben beide von der Firma und es war halt dann wirklich so, dass Chuck Bumm alles zusammengebrochen ist und wir einfach keine Einnahmen gehabt und die Perspektive,

ja damals auch alles in den Sternen gestanden ist. Man hat einfach nicht gewusst, wie geht es weiter. Und es war ja damals so, wo wir uns gedacht haben, also mein Mann und ich: Ma, so vier, fünf Jahre vor der Pension und jetzt das noch. Muss da sein? So, Ja. Und? Und ich war, also ich war eine zeitlang wirklich ziemlich unten und es ist mir gar nicht gut gegangen, weil das, das tut einfach nicht gut, wenn du nicht weißt, mit was du deine deine Dinge zahlst. Wenn kein Einkommen kommt, kommt kein Einkommen und ich weiß noch gut den Moment, wo ich innegehalten habe und dann dachte: hey, jetzt stopp! Was sind deine Stärken? Und das Interessante oder damals nicht so lustige war, ich habe sie auswendig nicht herkriegt. Ich hab sie auswendig nicht hergekriegt im Innen. Im einem Negativ-Zustand. In einem Stuck-Zustand hast du ganz schwer. Und da habe ich dann gemerkt, wie gut es ist, dass ich schon ganz viel Erfahrung mit dem gesammelt hat, dass ich mich schon mit dem beschäftigt hab. Und dann habe ich mir diesen Test geholt und dann hab ich mit die drei obersten Stärken angeschaut. Und das heißt noch lange nicht, dass alles leicht gegangen ist, es war viel Arbeit usw. Nur ich bin wieder ins Tun gekommen. Und das erlebe ich immer wieder mal, wenn es so Tage gibt. Oder wenn ich Supervisionen habe und die alle jammern, jammern, jammern und ich dann im Auto sitz und mir denk: Was kann ich jetzt Gutes für dich tun? Und dadurch ist es schon sehr präsent, das Modell für mich.

T (17:21): Weil du grad deine Stärken angesprochen hast, ist jetzt zwar keine Frage, aber ich bin neugierig. Was sind denn deine Stärken?

D (17:30): Ja, also. Also eine Stärke ist, eine Stärke, steht bei mir ganz oben. Das ist immer so, das war bis jetzt, glaube ich, steht ganz oben Weisheit.

T (17:40): Oh, sehr schön. Eine schöne, aber die sind alle schön. Das ist das schöne, das sie alle gut sind. Alles gute Dinge.

D (17:45): Ja, alles schön und eben diese Liebe zum Lernen, und mir dann bewusst macht okay, in der damaligen Situation. So, jetzt recherchierst, wie geht online Beratung, wie gehen, und wie macht man Webinare? Ich habe dann selber gleich Kurse belegt und dank meines Mannes, der technisch sehr begabt ist. Wir haben so, ma das kann ich dir zeigen, wir haben so einen Riesenschirm da. Also wenn ich 20 Leute im Sub-Seminar hab, dann sehe ich sie alle auf einem Schirm.

T (18:17): das ist Gold wert.

D (18:19): Ja, genau. Und du siehst da hinten, wir haben so eine Magnettafel da für uns gekauft. Das ist jetzt alles in unserem Besprechungszimmer, einfach alles fix installiert. So eine Art zoom Station. Und eben das, dass ich im Einzelcoaching, im zoom, da kann ich drauf schreiben. Und ich hab, ich hab so Magnetkarten.

T (18:39): Die sind super, die kenne ich auch. Die sind echt toll.

D(18:41): Die sind cool, ge? Ja, genau. Und dann haben wir, da haben wir da so eine Kamera, mit der kann ich dann mit wandern, wenn ich aufstehe.

T (18:51): das ist schön.

D (18:53): Das meine ich. Und dadurch hab ich, allein wenn ich das jetzt erzähl, merk ich, genau, da hab ich dann wieder Freude entdeckt, auch etwas ganz Neues zu machen. Und dadurch ist dann auch wieder ins Laufen kommen.

T (19:05): Die Liebe zum Lernen. Zu was Neuem entdecken.

D (19:08): Ja, genau. Die Neugier. Die Liebe zum Lernen. Genau das ist es.

T (19:12): Und vor allem, wie du auch sagst, auch da, ja, Corona hat uns da, glaube ich, alle in gewisser Hinsicht gefordert und gleichzeitig auch in dem Kontext, ist aber dann auch echt was weitergegangen und ich sage ja, da ja aus dem ähnlichen Kontext bin, kann ich das sogar wirklich ein bisschen gut nachvollziehen, wie das so gelaufen ist.

D (19:32): Ich glaube auch, in unserem Projekt hat uns das sogar, hat uns gut einer gespielt. Weil die Leute dann mit diesem Medium Zoom vertraut waren, wir hätten das ja gar nicht durchziehen können, in dem man, ja, wir hätten gar nicht so viel live Präsenz-Veranstaltungen machen können. Ja, und so viel Leute erreichen und die Scheu vor dem Medium war durch Corona weg.

T (19:59): Ja, das stimmt. Man hat da vielleicht auch das Positive dann wieder dran sehen können.

D (20:05): Und da hat dann irgendwie niemand... Ah, die machen das jetzt alles so, wir sind ja, wir sind ja nur in die Pilotschulen sind wir einmal im Jahr hin gefahren.

T (20:15): Genau. Also gerade am Anfang ist ja die Präsenz da. Genau. Ja, voll cool. Du, ich sage ja, so kann man sich ja eben aus jedem irgendwie was Positives mitnehmen. Geht schon, wenn man den Fokus dahin legt. Jetzt habe ich dich vorhin zum einen gefragt, inwieweit du vielleicht bei den Teilnehmerinnen draußen schon Veränderungen, einerseits bei dir selbst und jetzt interessiert mich aber natürlich noch, inwieweit sich vielleicht euer ExpertInnen Team in der Zeit, gerade durch die Beschäftigung, die Konzeptionierung. Also da habt ihr ja vermutlich viel Zeit miteinander verbracht, speziell Fokus auf die PERMA Faktoren. **Inwieweit hat sich da vielleicht auch was aufs Team ausgewirkt? Wie würdest du es beschreiben, betrachten?**

D (21:05): Also ich find, wir sind ein Spitzenteam. Also was ich, was ich, was ich, was ich extrem schätze in dem Team, es ist so, wenn jemand eine Idee hat, die wird aufgegriffen, dann wird da nachgedacht, dann wird verfeinert. Also ich hab so ganz frisch den Eindruck, dass wir uns gegenseitig bereichern. Also ich denke an dem, was wir gestern besprochen haben. Jemand hat eine Idee, die andere sagt: uh, da könnte man das mit überlegen? Nur, ich weiß es nicht genau. Ich habe ja nicht alle früher gekannt, viele hab ich ja erst durch das Projekt dann kennengelernt. Ich finde schon, dass das in dem Projekt, das Team wirklich gelebt wird. Auch wenn die Eva was ausgeschickt hat. Da brauchen wir noch Leute, die beim Präsentieren helfen, sind immer gleich welche gewesen. Da habe ich Zeit oder da hab ich nicht Zeit. Aber das war so zu klar. Und es ist ja auch keine Selbstverständlichkeit, dass, ich mein, jetzt im Endfinale. Jetzt im Endstadium haben wir

uns wirklich alle spezialisiert. Ganz am Anfang waren immer mehrere dabei, bei der, auch wie dann die Module und die, die die Powerpoints erstellt worden sind. Jetzt ganz zum Schluss man war mal wirklich, im Endstadium haben die Sonja und ich die Powerpoints gemacht. die Sonja ist immer die, die alle, die das alles so auf diese Ebene noch bringt. Und, und wird und wir haben immer davor mit Eva und Ingrid und wer halt sonst noch aus dem Expertinnen Team Zeit hat, hat Ideen gesammelt und wir haben dann gemerkt: genau, jedem das zu lassen, wo die Stärken sind und. Und bei uns ist halt die Sonja, die, die das unheimlich gut umsetzen kann und in einer PowerPoint und in dieser Einfachheit und auch Klarheit das hinaufbringen kann. Und ich glaube, da sind wir gewachsen, dass wir wirklich erkennen: aha, da liegen Stärken, da sind Ressourcen und, und alle anderen haben dann die PowerPoint verwendet. Also man hat dann nicht angefangen noch was rein zu stückeln oder ja und und und da gab es ein kurzes Briefing und das ist dann so umgesetzt worden. Und da waren wir am Anfang vielleicht schon, da ist dann da, wenn die da präsentiert hat, ist das noch rein gestellt worden und das noch und ich war beim Modul eins und beim Modul zweit, das ist ja ganz oft gehalten worden und und dann hab ich mir gedacht, was haben sie jetzt schon wieder geändert bei der PowerPoint...also das ist sicher etwas, wo wir gewachsen sind…also, so ist es jetzt.

T (23:56): Ja, ich glaub gerade in so einem Entwicklungsprojekt, da entwickelt sich ja im Namen schon was. Und auf der anderen Seite jetzt gerade in dieser Anfangszeit, in der Konzeptionierung, wo ihr eben die Module, die Praxis Angebote, euch da mal ja, eben in dieser Anfangszeit. **Was waren da wirklich so eure ersten Schwerpunkte oder Ziele in dieser Zeit?**

D (24:23): Du, im Grunde genommen wirklich wie kriegen wir, jetzt sag ich mal, in dieser Knappheit, weil wir haben Modul eins, Modul zwei. Modul eins und Modul zwei, haben immer von 14 bis 17 Uhr ungefähr gedauert. So Stärken-Cafés in zwei Stunden und nicht mehr. Und ich denke mir, das war auch ganz am Anfang war ganz viel Brainstorming und Sammeln. Wie kriegen wir in dieser Knappheit des Wesentliche, was wir rüberbringen wollen auf den Punkt.

T (25:00): Und was war dieses Wesentliche speziell für euch?

D (25:04): des war dann, es war dann schon die PERMA- Hand , die, die Eva und die Ingrid entwickelt haben, die ja auch gewachsen ist mit diesem (...). Die haben sich ja auch verändern dürfen. Das ist alles in der Zeit entstanden, die nur bissel präzisiert worden sind. Und das Wesentliche war wirklich zu sagen, zu jedem Buchstaben, also einerseits immer eben was bringen wir aus der ganzen Fülle für einen wesentlichem Input. Was ist der Hintergrund, der wissenschaftliche Hintergrund? Und dann eben immer die Übungen. Und da hat natürlich uns die Ingrid aus ihrem Repertoire und ihrer Fülle enorm viel geholfen und und beigetragen. Das ist ja auch ihr die, ihr Baby, ihr, ihr Ja… Denn wir dürfen ja dein Stück mitwirken. Also ich darf da Stück mitwirken. Aber, aber das ist ja wirklich das, was die Ingrid und die Eva miteinander da konzipiert haben und gemacht haben. Und ich glaub, da ist es auch fest darum gegangen, wie können wir die

mit vielleicht anderen Blickwinkeln unterstützen, dass man das jetzt auf der Schulebene umsetzen kann? Ja.

T (26:17): **Was war so dein persönlicher Schwerpunkt?** Hat´s da was geben, so was du unbedingt da auch unterkriegen wolltest?

D (26:27): Ah, den habe ich verlassen.

T (26:29): Okay…?

D (26:31): Ja. Wir haben ihn immer wieder angespielt. Und wir haben ihn gestern auch wieder angespielt. Also, mein persönliches Thema wäre sicher gewesen, wenn man es auch noch im informellen Managementsystem implementieren kann. Mir wurde aber bewusst, dass viel zu wenig Schulen, die Lehrpersonen über das System Bescheid wissen. Auch wenn es angedacht ist, dass das alles auf der Ebene mit den Lehrerinnen erarbeitet wird, wird es nicht getan. Und ich weiß nur von den Schulen, wo ich Prozesse begleitet habe, dass das wirklich so passiert. Und da haben wir uns dann oder da hab ich mich dann verabschiedet und gesagt ja, okay, das ist jetzt in dem Zuge noch nicht schaffbar. Das wäre vielleicht der nächste Schritt, wo man dann sagen kann, wie kann es sozusagen auf der Qualitätsebene noch mehr verankert werden, ja.

T (27:26): Meine nächste Frage wäre jetzt eigentlich gewesen, wie ihr euch da einigen konntet, weil wenn jeder so a bisserl was hat, was ihm halt sehr wichtig ist. Jetzt hast du gleich gesagt, so, du hast halt...Ja. Oder bzw. wie ist es in den anderen Punkten gelaufen? Sagen wir mal so, weil da hast du für dich entschieden. **Okay, ein bisschen zurückzutreten und gleichzeitig bei den anderen Inhalten, wie ist es da mit der Einigung laufen?**

D (27:56): Unkompliziert, also ich glaub... Also ich habe den Eindruck gehabt, dass so eine Wertschätzung in dem Kernteam, Eva und Ingrid, von uns allen anderen da ist und wir das alle so anerkennen, dass ja das, dass wir ein Stück Beiträge leisten dürfen. Und so habe ich das auch immer wahrgenommen, wenn mehrere, wenn wir uns mit mehreren getroffen haben, da war immer unter uns allen ein sehr wertschätzender Austausch und ich glaube, dadurch ist es auch gelungen, dass wir es so gut auf den Boden gebracht haben.

T (28:34): Wie würdest du dann so trotzdem deine Rolle im Team beschreiben?

D (28:43): Hmmm. Meine Mama hat mal zu mir gesagt, ich muss das jetzt sagen. Ganz ehrlich, ich war ganz, ganz stolz und hat mich wahnsinnig gefreut, wie sie mich gefragt haben, ob ich mit im Expertinnenteam dabei bin. Und ich habe das dann meiner Mama erzählt. Meine Mama wird jetzt nächste Woche 86 Jahre und meine Mama hat dann zu mir gesagt Doris, jetzt, jetzt bist alt, jetzt bist dann schon bei die Expertinnen.

T (29:15): Also das heißt die, die die Weisheit…

D (29:18): Ja, ich habe es einfach schön gefunden, dass man dadurch, dass sie jetzt vielleicht einerseits aus dem Schuldienst heraußen bin und doch immer wieder mit Schulen zu tun habe, dass man einfach da so meine Wahrnehmung auch mit einbeziehen will. Und ja, mein Gesamtwissen über Kommunikation, Konflikte sind ja die Themen, die ich hab. Jeder hat gewusst, im Kernteam, vor allem Eva und Ingrid, wenn irgendwo was ist, darf man mich anrufen, dann bin ich so ihr Coach a bissi...

T (29:54): Okay, so a bissl in Richtung Konfliktmanager, so, so...

D (29:57): Ja was nicht jetzt irgendwie groß auftreten ist, eher so einfach in der Fülle, wie gehe ich mit der Fülle wieder um, weil das war schon immer wieder ein großes Thema. Wie kommen wir mit dem Zeitmanagement zusammen? Weil ja alle, ja, niemand von uns hat das hauptberuflich gemacht und wie können wir da gut mit dem umgehen? Und ich denke, ich hab vielleicht mit dem einen oder anderen Telefonat so bissi Entlastung bringen können, meine Expertise als Coach also, ja.

T (30:34): Cool, auch wieder so eine persönliche vielleicht Stärke, die da jeder so einbringen kann. Du, und gleichzeitig innerhalb so dieser Teamarbeit. Du hast vorhin die Wertschätzung erwähnt, **was ist dir noch besonders wichtig in der Zusammenarbeit im Team gewesen?**

D (30:52): Das voneinander, miteinander lernen, also dass so alle Menschen ihr Wissen, ihr Know how eingebracht haben und dass wir aneinander oder miteinander dann so ein tolles Produkt geschaffen haben und miteinander auch gewachsen sind. Und ich glaube, es ist wirklich aus dem entstanden, weil aus mehreren Ebenen Wissen verknüpft worden ist.

T (31:21): Ja, grad weil du sagst, aus mehreren Ebenen ist Wissen verknüpft worden. **An welchen schon empirisch basierten Inhalten, sprich oder anderen pädagogischen Methoden habt ihr euch während der Konzeptionierung noch orientiert?** Neben speziell jetzt PERMA natürlich…

D (31:43): Na ja, also ich meine, der Hauptfokus war schon, und alle die, also alle Elemente, alle Wissenschaftsbereiche, die da noch einfließen, also Broaden & Build und, und diese Dinge auch mit Gefühlen, so ein Stück. Ich hab so ein Stück das noch eingebracht, also von Robert Diltz, diese Neurologischen Ebenen, also diese Wichtigkeit von Arbeit mit Werten, das haben wir bei den Herausforderungen so als ein Tool noch gebracht. Das war vielleicht des, also ich denke schon, also der Großteil, ist einfach aus all den Bereichen, die halt da zufließen gekommen. Eh ein Stück Resilienz, ja, das hat auch was mit der positiven Haltung zu tun.

T (32:37): Okay, also auch NLP? Also wenn du Diltz, die logischen Ebenen... also ist ja auch ein Konglomerat aus allem. Und die Resilienz, voll fein... da auch speziell ein Modell oder so grundsätzlich, weil gibt es ja da auch, und kommt immer aufs Gleiche raus... gab es da auch so eine spezielle Herangehensweise für dich, die dir da oder die euch da auch wichtig war?

D (33:02): Wie meinst du?

T (33:04): Im Bereich Resilienz? Weil da gibt es ja fünf Schlüssel, sieben Tore und wie sie alle heißen…

D (33:09): Ja, aber das ist, also ich sag jetzt ja mal grundsätzlich, dass es eben in diese Haltung der Positiven Psychologie im Grunde genommen vernetzt es sehr, ganz viel mit den Ansätzen der Resilienz Forschung oder der Salutogenese auch, sodass wir eben auch gesagt haben, immer wieder darauf hingewiesen haben, es ist nicht etwas, was so ganz alleine irgendwo steht, sondern dass da viele Elemente mit hereingebracht werden und einfließen. So ja. Oder jemanden, den Flow Effekt, den haben wir auch erwähnt, beispielsweise der Csikszentmihalyi, also genau so Sachen.

T (33:47): Okay, sehr spannend, du und gleichzeitig habt ihr da ein wunderbares Konzept, das jetzt bereits draußen ist. Inwieweit waren dann schon, sage ich jetzt klar, mal über die Pädagogik gesprochen, aber in einer Frage würd es mich doch eher interessieren. Letztendlich soll es ja bei den Kindern ankommen und ich habe ein bisschen reingeschaut, also was da PERMA.teach anbietet. Also ich hätte es ja am liebsten gerne für meine PERMA-lead Trainings, diese Übungen und was da mit den Kids gemacht wird. Also, super cool. Und auf der einen Seite, **wie habt ihr da versucht, jedes Kind hat individuelle Bedürfnisse. Wie habt ihr versucht die abzudecken? Was war euch da besonders wichtig?**

D (34:36): Naja, die Auswahl. Und na ja, wir haben ja im Grunde genommen die nur unter Anführungszeichen, die Ebene der Pädagoginnen, das ist ja unser Zugang, in dem man halt einfach wir haben es ja so aufbereitet Modul eins Modul zwei das immer Übungen zu jedem Buchstaben für die eigene Ebene war und dann die Übungen für die Schülerinnen Ebene. Und natürlich auch einfach in unserem Vortrag den Hinweis, dass es deshalb auch so eine Fülle an Übungen, weil eben die eine Übung bei dem Schüler bei der Schülerin greift und vielleicht für Begeisterung sorgt und eine andere Übung beim anderen Schüler und das denke ich, das war halt einfach von uns unsere Haltung. Und immer wieder der Hinweis, dass ja, so sind ja die Bücher gedacht, die soll ja nicht mit der ganzen Klasse durchpauken, sondern es sind ja Angebote. Es wird Kinder geben, die werden Seiten davon lieben und Übungen davon lieben und es wird Kinder geben, die werden Dinge auslassen und, das, dieses Modell dazu gedacht ist, dass man eben ganz speziell, ganz individuell arbeiten kann und es wird sich jedes Kind irgendwas finden.

T (35:47): Also sprich, eigentlich so ein bisschen die Vielzahl?

D (35:51): Ja, dadurch haben wir schon bewusst so eine Vielzahl an Übungen und wenn wir jetzt ins Stärken Café drei gehen, dann machen wir zu jedem nur mal so die Quintessenz und wirklich so, denken wir uns halt nochmal so Übungen aus, die so jeden Tag gehen, so Alltagsübungen, wo du nichts vorbereiten muss, die du einfach so, zack, zack, Tag beenden mit Danke, was war heut wunderschön oder so, so schnelle Dinge, wo man sie noch einmal darauf hinweist. Und wenn man halt etwas intensiver und länger damit arbeiten will, dann kann man sich ja eben die ganzen Übungen, die längeren Übungen mit denen auseinandersetzen.

T (36:32): Ja, also quasi von einfach schnell, Tiny Habits, bis große, große Bandbreite, sehr cool. Du und gleichzeitig, jetzt eben wenn wir bei diesen Inhalten letztendlich sind. Uns interessiert natürlich auch noch, wie du, ah passt eh super aus Qualitätsmanagement Sicht. **Ja tatsächlich, wie beurteilst du die Qualität von den entstandenen Schulungsbausteine? Wir haben speziell drei uns da rausgesucht, wo mich deine Meinung sehr interessieren würde, Doris. Zum einen die Präsenzveranstaltungen, eine Skala von 1 bis 10, du kennst das zehn wär Top. Wie ist deine qualitative Einschätzung, wenn es um die Präsenzveranstaltungen geht?**

D (37:22): Also, die Qualität, die war top. Ja, was man überdenken könnte, ist: reichts? weil wir haben nur eine Präsenzveranstaltung? Und sie hätten ja sich ja per Coaching begleiten lassen können und es hat sich keine einzige das Schule gegönnt.

T (37:47): okay…

D (37:48): Ja. Und das ist. Ja so ein bissi ein Wehmutstropfen, weil ich mir denke: ma schade, da hätten wir jetzt wirklich diese genau individuelle Begleitung bekommen, wo man sich einmal hingesetzt hätte, eben im Sinne von Qualität. Was macht ihr schon und wo könnt ihr jetzt einen kleinen nächsten Schritt machen? Wie könnte das für die Gesamtsumme ausschauen? Wie könnte es für die einzelnen Klassen ausschauen? Ja, aber das. Nicht jetzt an dem, was wir zur Verfügung gehabt haben, sage ich, war die Qualität bei den Präsenzveranstaltungen also gut, da, wo ich aufgetreten bin mit der Ingrid, das war gut, das war sehr gut.

T (38:32): Ist gut? Sehr gut ist eine 10?

D (38:35): Also gut ist, gut ist... Und deshalb habe ich es jetzt noch mal gesagt. Für mich ist es wirklich. Ich sag ja, im Sinne dieses Zeitbudgets haben wir nicht mehr machen können, das ist zur Verfügung standen. Deshalb habe ich jetzt, sage ich jetzt so dieses Gut, wenn ich sage okay, ich könnte Präsenzveranstaltung machen, wo ich anderthalb Tage Zeit habe und das im Stück. ich merk das halt immer, wenn ich solche Dinge mache, wo halt die Menschen, wo man einen halben Tag beginnt und dann schlaft man drüber und am nächsten Tag kann man das verfestigen und vertiefen. Und da kommen dann noch andere Fragen. Ja, da trauen sich die Leute dann mehr Fragen zu stellen oder mehr nur in ihrem Kontext Überlegungen anzustreben und zu sagen, genau wie könnte ich bei dem Kind oder was könnte in der Klasse...ja, deshalb war es eben...

T (39:24): Also?

D (39:25): Von dem, was wir zur Verfügung gehabt haben, find ich, war es sehr gut.

T (39:28): Okay, also ich sage mal, inhaltlich qualitativ top. Eher dann die Quantität, dass man da vielleicht noch ein bisschen mehr Möglichkeiten... **Okay verstehe, du und gleichzeitig wie schaut es in dem Bereich da bei den Online Veranstaltungen aus?**

D (39:42): Also ich finde ja, dass die sehr gut waren, weil sie, also ich finde, die waren extrem gut durchstrukturiert. Wir haben ein irrsinnig gutes Zeitmanagement gehabt, weil wir zu zweit moderiert
