# Supplementary material for: PERMA.teach: a study on the effectiveness of a standardized positive education training program in Austria
Source: Front Psychol. 2025 Apr 8;16:1516572. doi: 10.3389/fpsyg.2025.1516572 (PMC12053473; doi:10.3389/fpsyg.2025.1516572)
Supplement: Supplementary file 2 [file Data_Sheet_1.ZIP › Material PERMA.teach/Material LPs/Interviews LPs Teil 3.pdf]

4. Interviewtranskription von [redacted] (19.06.2023 um 19:00 Uhr)

00:00
B: Hallo, hallo, sie sehen mich auch und hören mich, ja? Okay, erstmal danke für ihre Zeit. Kurze Vorstellung zu mir, mein Name ist Kristina Baum und ich studiere an der IU-Kindheitspädagogik. Arbeite selbst als Vertretungslehrerin und schreib gerade meine Bachelorarbeit. Ich bin deshalb in die Forschung ein bisschen reingerutscht.

00:27
B: Wir haben mit der Uli ein paar Fragen zusammen überlegt. Das sind insgesamt acht Fragen und für jede Frage haben wir demnach so ungefähr sieben Minuten. Ich zeichne hier alles mit dem Handy auf. Dann werde ich dann nachher alles verschriftlichen. Gibt es irgendwelche Fragen von Ihrer Seite?

00:40
K: Nein.

00:46
B: Dann starte ich mal gleich mit der ersten. Die erste Frage ist: Was hat sie dazu bewegt an dem PERMA-Projekt teilzunehmen?

00:47
K: Ich habe ursprünglich, also ich komme aus der Gesundheitsförderung aus der schulischen Gesundheitsförderung und habe an der Pädagogischen Hochschule das Gesundheitsförderungszentrum aufgebaut. Und habe im Rahmen dieses Aufbaus dann auch die Glücksausbildung beim Ernst-Fritz Schubert-Institut gemacht.

Und bin da dieses PERMA Konzept gestoßen. Und habe dann auch beim FPÖ arbeiten dürfen als Projektberaterin und bin im Zuge dieser Projektberatung auf dieses PERMA.teach gestoßen und haben gedacht, dass es genau etwas, was ich auch noch brauche in meiner Ausbildung und was mich interessiert hier noch genauer hinzuschauen. Und ja deswegen habe ich mich dann dafür gemeldet.

1:52
B: Was würden Sie dann in kurz sagen sind genau ihre Beweggründe? Der Hintergrund? Also wegen dem Schulsystem oder wegen ihrem Job?

K: Vordergründig für eine Persönlichkeitsentwicklung also für mich selbst, aber natürlich in weiterer Folge dann für Schulentwicklung bzw. auch um vor Herausforderungen in der heutigen Zeit, gerade auf der psychosozialen Ebene, ein Tool zu haben, womit man in Schulen gut arbeiten kann.

02:25
B: Welche Herausforderungen sehen Sie in der Schule da genau, also aktuell?

K: Zurzeit ist es sicher noch einmal verstärkt worden durch Corona aber, dass die psychosoziale Gesundheit unserer Kinder und Jugendlichen schon sehr an… - wie soll ich sagen? Ja, sehr leidet. Ganz einfach auch unter diesen ganzen Bedingungen, Herausforderungen mit denen die Kinder konfrontiert sind. Sei es, dass es eine sehr schnelllebige Kindheit ist, aber auch laute Umgebung.

03:08
Also sie sind ständig mit mit Medien konfrontiert. Sie sind ständig mit Musik konfrontiert, also es ist überall sehr laut. Also sie haben diese Medien, mit denen sie konfrontiert sind. Sie sind dadurch auch sehr konfrontiert mit Themen die eigentlichen Kinder noch nicht betreffen sollten. Sie kriegen viel mehr mit vom Krieg. Sie kriegen viel mehr mit von der Problematik: Klimaschutz beziehungsweise Klimakrise, Energiekrise.

03:43
Ich glaube nicht, dass man früher als Kind so betroffen war und das ist heutzutage viel viel Ärger und auf der anderen Seite aber natürlich auch sehr gestresste Erwachsene. Größtenteils die auch mit den Herausforderungen oftmals nicht umgehen können. Und daher glaube ich, dass die Schule hier oder auch die Lehrkörper, der Lehrkörper, die Aufgabe hat hier so ein bisschen auch Tools in der Hand zu haben und Kinder ein bisschen begleiten zu können oder besser begleiten zu können.

04:19
B: Haben sie dann eine Veränderung wahrgenommen, also seitdem sie in dem pädagogischen Bereich sind oder würden sie sagen, dass es jetzt sehr spontan kam durch Social Media?

K: Spontan nicht, das ist eine Entwicklung. Also ich bin seit 30 Jahren im Schuldienst tätig und da ist eine gravierende Veränderung. Also ich finde die Nullerjahre 2000 bis 2010, die waren sehr angenehm. Da habe ich mir gedacht das geht alles so in die richtige Richtung, damit ich das Gefühl gehabt wir schaffen es die Jugendlichen und Kinder zu stärken. Seit 2008/2009 ist halt vieles passiert. Und das so ein schleichend war. Auch natürlich die Generation der Eltern, die eine ganz andere Einstellung auch hat zum Eltern sein und dann natürlich die ganzen Herausforderungen unserer Gesellschaft.

05:25
B: Kommen wir zur zweiten Frage, welche Veränderungen haben sie in dem Entwicklungsprojekt bei den Teilnehmenden festgestellt? Also gezielt bei den Lehrern, die sie ausgebildet haben?

05:42
K: Was habe ich da festgestellt?

05:50
K: Das ist auf jeden Fall einmal eine tiefergehende Auseinandersetzung mit ihrer Lebensgeschichte, mit ihrer Biografie war. Und das natürlich dadurch auch so die Punkte herausgearbeitet werden konnten oder Sie sich auch bewusster wurden, wo denn so ihre Schwachstellen sind, wo sie angetriggert werden können. Wo Sie einfach auf sich gut aufpassen müssen. Aber natürlich auch der Blick auf ihr zukünftiges Arbeitsfeld, wo sie dann auch mit Hilfe dieser Methode mit Hilfe dieses Konzepts, mit Hilfe dieser Haltung auch entgegenwirken können und Kinder gut begleiten und unterstützen können.

06:50
B: Also haben sie festgestellt, dass die Teilnehmenden beruflich, aber auch privat eine Veränderung durchgemacht haben? Gibt es da vielleicht konkrete Beispiele, die sie von den Teilnehmern erfahren haben?

7:10

K: Ja, auf alle Fälle! Vor allem einmal so der Blick auf das Positive und auch so diese Wahrnehmung. Es braucht drei positive Blickpunkte, drei positive Meldungen, drei positive Feedbacks gegenüber den einem Negativen. Und wie mächtig eigentlich so negative Meldungen, negative Rückmeldungen, negative Blickpunkte wirken auf uns. Und sich das einmal so bewusst wahrnehmen.

07:50
K: Da haben einige Teilnehmer/ Teilnehmerinnen schon darüber sehr nachgedacht. Auch was macht das mit einem Kind, weil wir sind sehr Fehlorientiert und wir sind sehr schnell mit dem. Das ist falsch, aber auch dann zu überlegen: „Was kann ich Ihnen dann positives rückmelden und positive Meldungen geben? Und das natürlich dann auch so in Bezug auf was macht das mit mir selbst?"

08:19
K: Was ist das bei mir für ein Gefühl? Was macht das dann erst mit einem Kind? Das war so ein konkretes, aber auch natürlich gerade im Bereich der Stärken, dass man sich selbst auch einmal zu seinem Stärken bewusstwird. Wir behaupten zwar immer, wir können uns schnell sagen, was unsere Stärken sind, aber wenn man dann so einmal genauer hinschaut und sagen, was versteckt denn hinter diesen Stärken?

08:50
K: Wobei helfen sie mir? Dann ist es manchmal doch schwieriger.

B: Haben sie auch in irgendeiner Form negative Veränderungen wahrnehmen können oder wo vielleicht die Teilnehmer/ Teilnehmerinnen eher zu sehr ins Grübeln gekommen sind oder irgendeine andere Form?

K: Das ist immer die Frage, ob man Grübeln als negativ sieht. Es sind viele ins Grübeln gekommen, ja.

09:21
K: Ich habe selbst in einem Rahmen in der Lehrerfortbildung oder Lehrerausbildung eine kleine Forschung gemacht. Wir haben ein ganzes Ausbildungsmodul dazu gehabt zu diesem Thema. Und wir haben dann ein Jahr, nachdem sie an der Pädagogischen Hochschule abgeschlossen haben, die Lehrer Lehrerinnen noch einmal gefragt, was sie denn so mitgenommen haben und worauf sie jetzt nach einem Jahr noch immer schauen.

09:54
K: Und das traurige war oder in sehr vielen Fällen, dass es im Alltag ganz schnell wieder weg ist. Also wenn man nicht wirklich dranbleibt und sich wirkliche Ankerpunkte setzt, dass ich immer wieder konfrontiert werde mit diesem Thema. Dann ist alte positive Veränderung auch relativ schnell wieder weg. Man kann sie zwar dann wieder schneller hervorholen.

10:20
K: Wir haben sie dann wieder begleitet und es war dann wieder relativ schnell da. Aber so im Alltag das dann zu leben ist dann doch schwieriger als man glaubt. Und es nutzt nicht einmal ein Semester, wo wir uns wirklich intensiv damit beschäftigt haben, wo man sagt wir haben doch längere Zeit Phasen dazwischen gehabt.

10:55
K: Da hat es gut funktioniert, aber dann im Alltag, wenn niemand da ist. Der sagt du. Übrigens schaue mal auf dich und schaue mal auf gewisse Punkte. Es ist relativ schnell wieder weg.

B: Was glauben Sie persönlich, wie oft bräuchte man so eine Art Erinnerung? Er was Tägliches, wöchentliches oder wie könnte man das so gestalten, dass die Veränderung wirklich lange Zeit dabei ist?

11:21
K: Ich glaube, dass das wirklich… Da müsste es gerade in der Ausbildung. Müsste das etwas geben, dass die gesamte Ausbildung sie begleitet. Und wirklich dran bleibt an dem Thema und nicht immer unterbrochen wird durch andere Lehrpersonen und doch wieder ein bisschen einen anderen Blickwinkel auf die Sache. Also sie haben schon Coaching die ganze Zeit, aber wenn wir jetzt nicht jemand drinnen ist der genau nach diesen Modellen nach diesem Verhaltensmuster arbeitet, dann ist es wieder weg. Ja und ich glaube, dass es dann auch sehr viel. Überzeugend braucht.

12:12
B: Also so eine Art Buddy System auch dann unter den Kolleginnen, dass man sich gegenseitig ermutigt?

K: Ja oder eben mal eine Schulleitung, wo man sagt, das ist in der Schule so implementiert, dass sie das immer wieder anstößt und immer wieder vorliegt. Ja, muss ja nicht einmal sagen, du machst das. Das ich etwas als Schulleiterin umsetze und womit ich die anderen wieder so auch daran erinnert für sich was zu tun. Die Inspiration innerhalb des Kollegiums in der Arbeitswelt.

12:52
B: Ist ein guter Punkt. Kommen wir zur dritten Frage, das bezieht sich jetzt auf Sie, also welche Veränderungen sie bei sich und im Experten Team feststellen konnten?

13:06
K: Ja, viel tiefer gehende Auseinandersetzung. Einmal überhaupt mit diesem Konzept. Also ich kannte es und ich habe schon einmal erarbeitet, aber in dieser Form der genauen Auseinandersetzung war es natürlich noch einmal viel tiefer gehend. Und auf der anderen Seite aber sehr bereichernd auch durch die Rückmeldungen der Teilnehmer, der teilnehmenden Personen, auch so wieder neue Ideen aufzugreifen und noch mehr hineinzuwachsen in diese Haltungsebene.

13:45
B: Haben sie da auch Feedback von ihren Kolleginnen bekommen, also von den anderen ExpertInnen oder haben Sie sich da nicht so ausgetauscht?

K: Nein, diesbezüglich eigentlich nicht. Und dadurch, dass wir auch also mit vielen ExpertInnen, bin ich jetzt nicht so zusammen, dass wir sagen könnten sie erleben mich im Alltag. Also über die Ausbildung und über die die Seminare da schon, aber dann würde ich jetzt nicht sagen, dass ich da wesentlich was verändert hat von mir. Sondern das ist wirklich so mein privates mein inneres, wie ich jetzt in der Früh aufstehe, wie ich rausgehe, wie mich in einer Schule hineingehe. Also eher da.

14:47
B: Wie würden sie sagen hat sie das beruflich verändert?

K: Ja, auf alle Fälle. Ich schaue viel mehr darauf, dass für diese Dinge Zeit ist, dass der Rahmen gesetzt wird, dass ich auch darauf

achte. Ebenso auf meine Sprache, auf gewisse Gestiken, die ich mir den Kindern mache, oder…

15:16
K: Ja also da gibt es viele Dinge, wo ich mir denke. Da bin ich jetzt viel sensibler geworden, noch einmal durch diese so intensive Auseinandersetzung im Expertenkreis.

B: Gibt es denn auch - jetzt wieder die gleiche Frage - auch negative Aspekte, die sie wahrgenommen haben?

15:41
K: Nein. Nein. Alles positiv! Also ich denke mir auch diese auseinandersetzung mit mir, auch wenn etwas schief geht. Das hat nichts mit rosaroter Brille zu tun, sondern es ist wirklich so, dass ich sage: „Ich, ich bin in der Lage auch meine negativen Gefühle wahrzunehmen, anzunehmen und damit umzugehen.“ - Und ich glaube das ist wieder ein positiver Aspekt.

16:07
K. Ja, aber das heißt jetzt nicht, dass mir immer nur gut geht und ich immer nur glücklich bin. Aber es ist ganz einfach eine andere Ebene der Auseinandersetzung und der Selbstpflege auf dahinter.

B: Konnten sie auch zwischenmenschliche Veränderungen im Familien Freundeskreis feststellen?

K: Ja, dass ist vielleicht etwas Negatives, wenn man das so sieht. Dass ich manchmal meiner Familie oder meiner Partner - nicht auf den Nerv gehe – aber schon manchmal. So nicht schon wieder, aber ich sag: „Wofür bist du denn heute dankbar oder so?“ Ja oder, wenn wir so spazieren gehen oder es ist ein Geburtstag und ich sage: „Was sind denn so deine Wünsche, deine Ziele für die nächste Zeit?“ Und so. Also immer wieder, so dass ich es doch in so meinen Alltag auch Einbau, wo dann halt schon.

17:13
K: Ich mein ist auch meinen Berufen geschuldet. Aber es ist dann eher immer so auf einer witzigen Ebene und auf einer angenehmen. Also jetzt nicht, dass wir deswegen streiten.

B: Also nehmen Sie das Wissen auch mit in ihre zwischenmenschlichen Beziehungen?

K: Ja.

17:20
B: Sehr schön. Zur vierten Frage, dass die Frage: Welchen Schwerpunkt und welche Ziele wurden bei der Konzeptionierung verfolgt?

17:51
K: Was wurde da verfolgt bei der Konzeptionierung? Also bei der Konzeptionierung? Auf jeden Fall, dass man dieses Konzept auf eine Haltungsebene bringt. Das ist ein Rahmenmodell. Es ist für eine Haltung, die man einfach aufbauen sollte und dass es nicht um reine Wissensvermittlung geht, sondern, dass es wirklich darum geht, dieses Modell in sein Leben zu integrieren und in weiterer Folge dann eben auch in seinem Leben, seiner Arbeitswelt zu leben und es dann noch weitergeben zu können.

18:34
K: Eben an vor allem Schüler, Schülerinnen, Kinder, Jugendliche beziehungsweise auch in vielerlei Studierende. Aber grundsätzlich war immer so dieser Hintergedanke und dieses Ziel der Konzept. Wie bringen wir eine Haltung hinüber? Und nicht reine Wissensfakten. Und darum war es uns so wichtig eben sehr viele Übungen einzubauen. Ein breites Spektrum an Ideen aufzuzeigen, aber eben auch die Ideen der Teilnehmer hereinzuholen und zu schauen, was bringen die mit? Was ist denn schon vorhanden? Wo lässt sich das einordnen?

19:18
B: Also das es wirklich eine Art Lebenshaltung, eine Einstellung wird, die wortwörtlichen in Fleisch und Blut übergeht?

K: Ja, genau.

B: Konnten sie sich denn bezüglich der Schwerpunkte und Ziele im Team gut einigen?

19:50
K: Ja, ja. Also das habe ich sehr angenehm empfunden. Ich habe mit mehreren verschiedenen ExpertInnen zusammengearbeitet und das war immer sehr einfach sich da zu einigen. Aber es wurden auch diese individuellen Bedürfnisse wahrgenommen und wo man seine Stärken hat. So wie, das, was wir denn Teilnehmern mitgeben wollen, wurde auch im Expertinnnenteam sehr wohl gelebt und hier umgesetzt!

20:28
B: Sehr schön! So lässt es sich gut arbeiten. Schönes Arbeitsklima.

K: Ja, ja genau.

B: Dann jetzt die fünfte Frage ist, wie würden sie ihre Rolle im Team beschreiben?

20:56
K: Ich bin an sich eine, eine - Ich kam in den Genuss damit arbeiten zu dürfen. Ja, ich bin jetzt keine tragende Rolle. Also ich bin eher so die die eben mitwirken darf und für mich eben sehr viel selbstlernen durfte. Aber die Konzeptionierung da war ich nur am Rande beteiligt. Ich war hauptsächlich eben in den in den Seminaren als Referentin dabei.

21:22
K: Aber die die Kopfarbeit und genaue Konzeptionierung und Erstellung das haben alle andere gemacht, weil da war nein Zeit Ausmaß, das ich dem Projekt zur Verfügung stellen konnte, auch nicht ausreichend genug, aber das war von Anfang klar.

B: Wie sah ihr Zeitausmaß ungefähr aus während dem Projekt?

21:49
B: Also wie viel Zeit haben sie in ihrer Rolle aufgenommen?

K: Ich habe - Jetzt nageln sie mich gerade fest. Wie viele Fortbildungen ich mit begleitet habe? Es waren einige. Aber es war so jeden Monat maximal ein, zwei Fortbildungen mit Vorbesprechung und einer anderen Nachbesprechung. Ja, aber ich war jetzt nicht die, die eben bei den genauen Konzeptionsschritten überall dabei war.

22:26
B: Was war Ihnen bei ihrer Rolle dann wichtig? Also waren sie zufrieden mit der Rolle oder hätten Sie gerne was geändert?

K: Nein es war ich war total dankbar dabei sein zu dürfen und um dieses Konzept eben oder diese dieses Modell weitergeben zu dürfen und dann mitzuarbeiten, dass es in die Welt hinauskommt, dieses verbreitern und dadurch, dass ich auch an der Pädagogischen Hochschule ursprünglich tätig war damals war das für mich so okay.

22:57
K: Ich bin der Motor an einer Pädagogischen Hochschule, der das dort weiterträgt und durfte dann im engeren Kreis da noch bei der Ausbildung dabei oder bei der Fortbildung dabei sein. Ja? Aber ich möchte auch für meine, ich werde Schulleiterin und da möchte ich es als Schulentwicklungsprogramm nehmen und diese Schule auf diesen auf dieser Haltung aufbauen.

23:21
K: Dafür konnte ich ganz viel für mich mitnehmen, aber es ist jetzt nicht meine Priorität zu arbeiten.

B: Ja, interessant.

23:35
B: Dann ist die sechste Frage. An welchen pädagogischen Methoden und Maßnahmen haben sie sich während der Konzeptionierung orientiert?

23:46
K: Das ist eben die die ein bisschen eine Schwierigkeit, weil ich eben nicht konzeptioniert habe, und die Methoden habe ich größtenteils übernommen. Ich habe schon meine eigenen Ideen auch hineingebracht, sprich dass wir sehr viel geschaut haben, dass sehr viel Austausch dabei ist. Aber eben auch, dass er zum Beispiel Biographiearbeit gemacht haben, was für mich im Zusammenhang mit diesem Thema ganz ganz wichtig ist.

24:18
K: Das durfte ich hineinbringen. Wir haben sehr viel in Richtung Bewegung auch hineinbringen können oder ich durfte viel durch Bewegung hineinbringen, weil das auch so einer meiner Schwerpunkte ist. Aber grundsätzlich war ich jetzt bei der Konzeptionierung eben nicht so dran beteiligt und habe auch viele Methoden übernommen. Wie das aufgebaut worden ist.

24:47
B: Wie haben Sie bei den pädagogischen Methoden darauf geachtet, dass die individuellen Bedürfnisse der Kinder immer beachtet werden?

25:01
K: Insofern, dass die Teilnahme eigentlich oder immer freiwillig war. Ja das heißt, dass ich ein Angebot gesetzt habe und für wen es nicht gepasst hat, oder wer gesagt hat: Nein er darf da nicht oder will da nicht mitmachen, der musste auch nicht mitmachen. Ja? Also das ist bei vielen und das ist auch bei der bei der Fortbildung so gewesen, dass man sagt man…

25:38
K: Die Teilnehmer dürfen selbst entscheiden.

B: Wurde bei den Konzepten dann darauf geachtet, dass sage ich mal auch Kinder mit körperlicher oder geistiger Behinderung oder Kinder mit Migrationshintergrund, die kein Deutsch sprechen können, auch an den pädagogischen Maßnahmen teilnehmen können?

K: Jetzt muss ich sie kurz unterbrechen. Was verstehen sie unter Expertin? Sehen Sie mich als Expertin für die Fortbildung und Weiterbildung oder meinen Sie das, was ich in der Klasse umsetze?

26:07
K: Das sind für mich zwei Paar Schuhe. Ich bin nämlich Referentin auch weil beim PERMA.teach. Ich mache es aber auch in meiner Klasse, da sehe ich mich aber nicht als Expertin in dem Sinne. Ja ich glaube Sie vermischen da jetzt zwei Dinge.

B: Es ist bezogen auf die Teilnehmenden, die dann also die Maßnahmen erfahren haben, ja.

26:35
K: Okay, aber das sind ja nicht Kinder gewesen. Das waren bei mir Erwachsene, die das übernommen haben. Aber es ist ja eh für beide gültig. Es dürfen auch Erwachsene sagen, das mache ich nicht, da kling ich mich heraus und genauso dürfen, dass die Kinder machen. Ja also, wenn wir so eben diese warme Dusche angeboten haben oder so, dann darf auch jemand sagen: Nein das passt für mich jetzt nicht.

27:09
K: Aber drüber die vorige Frage, wo sehe ich mich in dem Expertenteam? Das war jetzt auch bezogen auf die Referenten. Auf die, die das Konzept, also diese Fortbildung Weiterbildung konzeptioniert haben. Ja, das bezieht sich nicht auf das, was ich in der Klasse mache.

B: Ahja, da ist der Unterschied. Dann ist die nächste Frage: Auf einer Skala von 1 bis 10.

27:35
B: Wie zufrieden sind Sie mit der Qualität der Schulungsbausteine?

K: Von eins bis zehn? 9. Also ich glaube das es gut aufgebaut ist - eventuell bräuchte es die eine oder andere Einheit mehr. Also ich glaube dann wäre es 10. Also der Zeitfaktor da könnte man vielleicht dran schrauben.

28:05
K: Es ist wieder die Frage, wie viele Teilnehmer TeilnehmerInnen würden es dann machen, wenn es wieder länger dauert. Aber das ist der einzige Faktor. Sonst glaube ich waren die Bausteine sehr gut gewählt und sehr gut aufgebaut. Dass all das rüber kam und auch vom Zeitabschnitt waren sie so aufgesetzt, dass wirklich Zeit dazwischen blieb, dass man sich damit auseinandersetzen konnte.

28:33
B: Welcher Baustein hat ihnen besonders gut gefallen?

28:41
K: Das stärken-café, heute. Also ich glaube, dass das Stärken Café ein ganz wesentlicher Baustein ist um das Ganze noch einmal reflektieren zu können, um noch einmal drauf zu schauen, wo stehe ich als Teilnehmerin.

Wo stehe ich? Welche Säule oder welchen Bereich habe ich gut im Fokus? Welcher Bereich ist noch gar nicht da?

29:06
K: Also ich denke mal diese Stärken Kaffee. Und das wäre vielleicht auch ein Punkt, wo man sagt, da noch die Menschen vielleicht doch noch ein zwei Stärken Cafés weiter zu begleiten.

B: Also das die länger fortgesetzt werden?

K: Ja genau, aber das ist in meine Kostenfrage. Das ist in meiner Zeitfrage. Wer will das machen?

29:30
K: Aber ich glaube, dass das Stärken Café so ein ganz wesentlicher Baustein ist, weil er mir am Anfang wie ich das erzählt bekommen habe und ausprobierte. Ja braucht man das noch? Ja man brauchts. Also das ist jetzt für mich einer der wesentlichsten Elemente.

B: Und die Präsenzveranstaltung - wir haben sie die genommen?

29:58
K: Welche?

B: Die Präsenzveranstaltung allgemein.

K: Ich hatte nie eine Präsenzveranstaltung.

B: Ah, oh. Es wurde mir ebenso mitgeteilt, dass da auch Präsenzveranstaltungen waren, aber die waren da nicht verpflichtend?

30:19
K: Das ist jetzt wieder die Frage: als was bin ich hier?

B: Sie sind als Expertin hier. Wurde mir so mitgeteilt.

K: Ja. Genau, ich habe an einer Schule habe ich eine Präsenzveranstaltung gehalten. Da waren wir in einer Pilotschule. Das ist immer angenehmer als online, aber ist dadurch, dass das also bei den anderen Fortbildungsveranstaltungen waren immer alle also aus ganz Österreich und teilweise aus Deutschland.

30:54
K: Da ist natürlich das Onlineformat leichter zu händeln. Ja das natürlich immer eine Präsenzveranstaltung angenehmer ist noch bereichernder ist, weil man einfach diese emotionale und persönliche Ebene viel mehr noch mitbekommt und spürt. Ist logisch.

31:25
B: Und dann die letzte Frage ist, wie es für sie in Zukunft weitergeht.

K: Also beruflich ist es so, dass ich jetzt eben meine Schule übernehmen darf an dieses PERMA.teach als Schulentwicklungskonzept als als Haltungsgrundlage für diese Schule einsetzen werde und umsetzen werde. Und dass ich hier ganz viel von diesen Elementen einbauen möchte in den schulalltag, sowohl bei meinen Kollegen Kolleginnen als auch eben bei den Schülerinnen Schülerinnen.

31:59

K: Das wir eben auch versuchen, da den Elementarbereich mitzunehmen. Das ist eben die eine Schiene und auf der anderen Seite darf ich sehr wohl noch bei den nächsten Ausbildungen Weiterbildungen im Bereich PERMA.teach wieder als Referentin tätig sein.

32:27
B: Also sie bleiben im PERMA.teach Bereich?

K: Ja. Genau.

B: Und nochmal zurück zu ihrer Schule, zu der Schulleitung. Wie genau planen Sie da PERMA.teach in den Alltag umzusetzen?

K: Also es soll unser… Diese fünf Bereiche von PERMA.teach sind unsere fünf Eckpunkte, sage ich immer. Wo ich sage innerhalb dieser Eckpunkte dürfen sich alle Lehrer Lehrerinnen bewegen.

33:00
K: Aber das sind so diese Eckpunkte, die ich als Rahmen sozusagen vorgebe und wir uns jetzt aber in der Schulentwicklung eben gemeinsam überlegen. Was bedeuten diese Rahmen? Was bedeutet es positive Emotionen in den Schulalltag zu bringen? Da geht es dann um Rituale. Da geht es um Schulgestaltung. Da geht es auch darum.

33:24
K: Wie empfangen wir Eltern. Wir haben einen Kindergarten auch im Haus. Wie gehen wir mit dieser Unterschiedlichkeit um? Wir haben auch Schwerstbehinderte in unserem Haus. Das heißt wie schaut eine Inklusion aus? Wie können wir da trotzdem oder gerade deswegen positive Emotionen immer wieder hervorholen? Wie können wir die Stärken stärken?

33:48
K: Was bedeutet das bei Kindern? Ich habe auch vor als Schulleitung da auch so eine Art Post zu machen. So angelehnt an die PERMA Post. Nur heißt es dann eben Gabi Post, dass ich so jede Woche auch die Kinder einen Brief schicke, was das bedeutet, worauf wir achten, diese Woche. Ihnen ein Spiel, eine Übung ein Buch mitschicken, was sie dann in der Klasse bearbeiten können, auch im Umgang eben im Kollegium.

34:21
K: Was bedeutet das? Was möchte ich für Sachen setzen, für Punkte setzen? Wie eben so kleine Feedbackzettel immer wieder hergeben, um auch einen Raum schaffen, wo wir eben gemeinsam feiern können, wo wir gemeinsam Erfolge besprechen können oder eben feiern können. Wie schaut meine Feedback Kultur aus? Also auf allen Ebenen und auch eben in Bezug auf was heißt eine Hausordnung in Bezug auf diese fünf Säulen?

34:54
B: Wie groß ist die Schule, die sie leiten werden? Also wie viele Lehrkräfte gibt es da?

K: Es sind 17 Volksschulklassen und 4 F.Klassen plus 9 kindergartengruppen und drei Kleinkindgruppen, wobei wir eine kollegiale Führung sind. Das heißt es gibt eine Kindergartenleitung und mich als Schulleitung.

B: Also eine relativ große Schule?

K: Für Wien ist es eine mittelgroße Schule

35:30
B: Sehen Sie da Herausforderung, dass auf so viele Lehrkräfte, sag ich mal, das weiterzugeben?

K: Ja, das ist eine große Herausforderung, denn ich habe noch nicht alle Lehrer. Die Lehrer, die ich jetzt habe, die konnte ich in Vorstellungsgesprächen schon darüber informieren oder sie schon da so ein bisschen prüfen und die lassen sich alle bewusst darauf ein.

36:02
K: Die Lehrkräfte, die ich jetzt bekommen, kann sein, dass ich die erst am 1. September kennenlernen und dann ist es schwierig. Die wissen nicht, worauf sie sich einlassen und bekommen das dann so ein bisschen vorgesetzt. Und da ist es dann die Schwierigkeit. Wie hole ich die ins Boot?

36:21
K: Die Leute, die ich jetzt schon kenne, die mit mir Vorstellungsgespräch hatten, die habe ich schon ein Stück im Boot. Die anderen halt noch gar nicht. Auf der anderen Seite auch wie kommuniziere ich? Wie lebe ich es für Eltern sichtbar? Also das die einfachste Ebene ist die Kinderebene.

36:45
K: Also ich glaube, dass es da einfach ist, weil das ist mein Handwerkszeug. Das kann ich. Die Kolleginnenebene könnte bei manchen eine Herausforderung sein. Aber ganz spannend finde ich, wie bringe ich es zu den Eltern hinüber.

37:08
B: Spannend ist ja auch zu sehen, wie bringen die Kinder als Multiplikatoren es zu den Eltern, oder?

K: Genau, das dann auch noch einmal. Aber trotzdem müssen die Eltern ins Boot geholt werden und müssen wissen was da passiert. Ja also nur von den Kindern erzählen lassen könnte auch wieder zu gewissen Widerstand führen. Aber das machen wir!

B: So ist das mit Veränderungen. Immer was Neues.

37:40
B: Gibt es dann sonst noch, was sie sagen, möchten zu dem Projekt oder allgemein?

K: Das ist eines der wichtigsten Projekte war, die ich bis jetzt miterleben durfte. Und das wirklich ganz viel tolle Arbeit innerhalb dieses Teams geleistet worden ist und ich eben dankbar sein darf, dass ich dabei sein durfte.

38:07
K: Das habe ich der Eva auch immer wieder gesagt. Eine ganz große Ehre war dabei sein zu dürfen.

B: Das merkt man bei Ihnen auch, dass sie da total hinter stehen.

K: Vielen Dank!

38:26
B: Das waren alle meine Fragen. Mehr Fragen habe ich nicht.

K: Doch keine sieben Minuten pro Frage. Ich bin immer sehr kurz und knackig in meinem Antworten.

B: Das ist auch prima, war ja alles dabei. Vielen Dank! Danke und viel Glück mit der Leitung wird bestimmt sehr spannend.

K: Danke, ja sicher eine spannende Geschichte. Alles Gute noch für Sie. Tschüss!

5. Interviewtranskription von Sonja Käferböck (20.06.2023 um 17:00 Uhr)

00:00
B: Fangen wir mit der ersten Frage an. Was hat dich dazu bewegt am PERMA-Projekt teilzunehmen?

K: Seitdem ich auf die positive Psychologie geschlossen bin, das war vor ca. 6,5 Jahren, habe ich mich gefragt, warum diese Inhalte, die mir damals so geholfen haben, nicht unterrichtet werden. Warum ich durch Zufall in meinem Auslandssemester auf diese Inhalte komme und warum das nicht im Schulsystem ist und das hat mich dann auch damals bewegt meine Masterarbeit in dem Bereich zu schreiben, wo ich mich gefragt habe, wie kann man positive Psychologie in Englischunterricht einbinden, weil ich eben als Lehre genau da Potential gesehen habe.

01:10
K: Und ja dadurch bin ich irgendwie in diese Community reingerutscht und habe da Gott sei Dank die Eva Jambor kennengelernt, die mich dann kontaktiert hat als das Projekt im Anlaufen waren. Für mich ist PERMA.teach ein Weg um diese Inhalte, von denen ich sehr überzeugt bin aus der positiven Psychologie an Menschen zu bringen.

01:33
K: Menschen, die vor allem auch als Multiplikatoren und Multiplikatorinnen eintreten können. Also ich sehe da einfach extremes Potenzial. Und ja für mich ist eben PERMA.teach eine dieser Wege wie ich das Wissen, von dem ich so überzeugt bin, weitergeben kann. Und besonders denke ich da an Kinder und Jugendlich.

01:58
K: Ich arbeite ja auch selbst in einer Handlungsakademie, also mit 14 bis 20-Jährigen und ich sehe den Bedarf so sehr dort und ich würde mir wünschen, dass dieses Projekt noch vertieft wird, weil der Bedarf so da ist und ich deswegen sehr dankbar war Teil dieses Projekts zu sein.

02:23
B: Du sagtest, du bist vor sechs Jahren mit positiver Psychologie in Kontakt gekommen. Wie bist du da reingerutscht?

K: Das war wirklich durch Zufall. Also ich war ich habe ein Auslandssemester gemacht an der University of Melbourn in Australien und das war am Ende meines Studiums. Ich konnte nämlich Fächer wählen, die mich interessiert haben. Also ich war nicht so eingeschränkt und ich bin einfach den Katalog durchgegangen von dem, was ich machen konnte, und habe halt so geschaut was mich anspricht.

02:53
K: Ich habe insgesamt drei Kurse gewählt und durch einen Zufall - ich hatte davor noch nie von der positiven Psychologie wirklich was gehört, obwohl ich Psychologie auch als Teils meines Lehramtsstudiums studiert habe. Auf jeden Fall bin ich in zwei positiven Psychologie Seminaren gesessen und dann hatten wir eben diese Kurse und ich war von Beginn an so fasziniert, dass Glück und

Wohlbefinden und Potenzialentfaltung und Persönlichkeitsentwicklung nicht nur eine Frage des Zufalls ist, sondern, dass da eine Wissenschaft dahinter ist.

03:21
K: Dass es diese Übungen gibt und diese Konzepte gibt und diese Methoden gibt, die erforscht sind und die erforscht werden und dass man mit denen das Wohlbefinden von anderen Stärken kann.

03:44
K: Ich habe zu dem Zeitpunkt auch dann auch gleich alles bei mir selbst im angewandt und war da selbst gerade selbst bisschen in einer Selbstfindungskrise und eben in einer Quaterlifecrisis. Das hat mir so viel Kraft gegeben. Also diese Übungen und dadurch, dass ich eben da selbst gemerkt habe, was das mit mir selbst tut. Hat sich eben das entwickelt, dass ich das weitergeben wollte, aber grundsätzlich bin ich in Melbourne auf der University zufällig mit den Ideen in Kontakt kommen.

04:16
B: Man merkt richtig dein Feuer was da brennt, wenn du redest. Magst du eben noch kurz sagen, wo du jetzt heute stehst? Also deine Berufung, was du machst. Du sagtest das Lehramt studiert hast.

K: Ja also ich bin hauptberuflich Lehrerin in Wien. Das mache ich hauptberuflich.

04:45
K. Daneben gebe ich immer wieder Workshops, Webinare, Kurse und auch manchmal Kooperationen mit anderen Menschen in dem Bereich, wo ich Beiträge machen kann bei anderen Größen zum Thema positive Psychologie, Persönlichkeitsentwicklung, auch viel Kommunikation, aber da auch viel im Bereich positiver Psychologie. Und ich habe eben diesen Blog und dau gibt es eine Webseite. Und auf Instagram und Facebook, wo ich eben zu diesen Themen regelmäßig poste. Also hauptberuflich Lehrerin und nebenberuflich, wenn man so sagen will, mache ich Kurse, Workshops, Webinare zu Themen Persönlichkeitsentwicklung und positive Psychologie.

05:33

B: Welche Veränderungen hast du in während dem Entwicklungsprojekt bei den Teilnehmerinnen festgestellt?

K: Wir hatten ja meistens die drei Termine und der letzte Termin ist das Stärken Café, wo die Teilnehmenden selbst auch Ideen teilen konnten oder Wege teilen konnten, wie sie PERMA eingebaut haben in ihr eigenes Leben und vor allem auch in die Arbeit mit den Kindern und Jugendlichen, mit denen sie zusammenarbeiten. Und eins, was immer wieder genannt wurde, ist einfach dieser veränderte Blickwinkel, diese veränderte Haltung.

06:20
K: Und das ist es ja auch, worum es uns eigentlich geht bei PERMA.teach. Das ist jetzt nicht unbedingt diese Methoden sind. Man muss jetzt nicht irgendwelche Methoden und Übungen unbedingt

einbauen. Es kann auch helfen. Aber es ist vor allem diese veränderte Haltung dieser Stärken, diese Stärkenorientierung und wenn man anders in der Klasse steht.

06:44
K: Und vor allem auch das veränderte Mindset. Wenn man trotzdem diese Übungen auch macht und sich halt bewusst auf fragt, was es heute gut gelaufen, wie geht's mir gerade? Wofür bin ich dankbar, dass ich da über die Zeit hinweg wirklich unser Gehirn eigentlich umstrukturiert und sich mehr öffnet für diese positiven Erfahrungen und das haben viele Teilnehmenden berichtet, dass jeder das auch merken, dass sie jetzt das auch verstärkter wahrnehmen, wenn positives passiert.

07:13

K: Das sie vielleicht vorher nicht wahrgenommen hätten. Also dieser Ausgleich der Negativitätsverzerrung, die alle Menschen besitzen, dass sich diese Negativitätsverzerrung in den Teilnehmenden verbessert hat oder weniger stark geworden ist. Das war eine Sache, die ich beobachtet habe.

07:40
K: Und vielleicht eine zweite Sache, die auch sehr viele Kollegen und Kolleginnen in den Seminaren gesagt haben, dass die, die eh schon viele dieser Methoden gemacht haben vorher. Natürlich haben Sie das, weil das ist eh irgendwie schon so drinnen. Das macht man auch intuitiv, aber sie haben jetzt den wissenschaftlichen Background dazu und sind jetzt noch motivierter das weiterzumachen.

08:03
K: Weil sie haben vielleicht vorher eben die Stärkenorientierung gemacht oder haben irgendwelche Dankbarkeitstagebücher schreiben lassen. Aber jetzt sucht das Seminar haben sie erfahren, das macht auch wirklich wissenschaftlich sind. Also das ist nicht nur was ich gut anfühlt, sondern das hat wissenschaftlich messbare Ergebnisse. Und dass sie sich einfach sicherer werden in ihrer bereits bestehenden Praxis von PERMA, auch wenn sie es damals nicht PERMA genannt hatten.

03:81
B: Sehr spannend. Ja macht Sinn. Ich schreib auch selber Dankbarkeitstagebücher und mit dem Wissen, dass es wirklich was in deinem Gehirn verändert, da ist man viel motivierter auch am Ball zu bleiben. Hast du denn auch von negativen Erfahrungen gehört oder Herausforderungen - irgendwas, was die Teilnehmenden geäußert haben?

08:53
K: Also jetzt im Sinne von der Umsetzung von PERMA oder in den Seminaren? Für mich? Also Herausforderung für mich oder Herausforderung für die Teilnehmenden?

B: Für die Teilnehmenden. Also in den Veränderungen, dass sich das Handeln von ihnen negativ verändert hat oder ob besondere Herausforderungen durch PERMA entstanden sind.

09:17
K: Was immer wieder kommt ist der Zeitdruck oder die mangelnde Zeit, dass sie so viel mehr umsetzen wollen würden, aber die Zeit hat nicht finden. Das ist ein großer Punkt. Auch ein Punkt, der jetzt nicht so oft vorgekommen ist, aber wenn ich mich noch erinnern kann, wo sie gesagt haben, dass einfach ein bisschen Gegenwind kommt aus dem Kollegium, wo halt manche Leute das nicht verstehen, wenn man eine ganz andere Einstellung zum Lehrberuf hat, dass da Gegenwind kommt.

09:52
K: Ich glaube das sind so die zwei größten Hürden. Also die fehlende Zeit das umzusetzen und der Gegenwind aus dem Kollegen.

10:15

B: Die dritte Frage ist ein bisschen ähnlich wie die davor. Welche Veränderungen hast du bei dir selbst und in dem Team der Expertin wahrnehmen können?

10:32
K: Beim Präsentieren, dass man sich natürlich sicherer wird. Also wenn man dann das siebte Webinar macht, ist man natürlich irgendwie noch

gesettelter und sicherer als jetzt beim ersten Seminar. Also so allein vom Präsentieren.

10:59
K: Ich finde auch, dass es ein gegenseitiger Austausch und eine Bereicherung ist, weil in jedem Kurs was - besonders in den Stärken Cafés - kommen ja auch Ideen von den Teilnehmenden. Es kommen immer wieder Ideen von den Teilnehmenden, wo sie Ideen einbringen. Man wird also bereichert, indem man sieht, wie vielfältig die Anwendung von PERMA eigentlich stattfindet oder was man alles machen könnte.

11:28
K: Also eine Erweiterung der Ideen, die man hat bezüglich der Umsetzung von PERMA. Das hat sich verändert.

11:44
K: Was mich auch begeistert hat ist, dass so viele Leute sich da angemeldet haben. Damit hat niemand gerechnet, dass da so viele interessierte Menschen waren. Am Anfang waren drei Reihen geplant und dann waren die schon alle überfüllt und im Endeffekt gab es sechs Reihen. Gestern beim Abschluss waren auch wieder 50 Leute oder so drinnen.

12:19
K: Und die sagen alle, wie geht's jetzt weiter und können wir da noch irgendwas bekommen? Also das einfach, das hat sich verändert dieses Bewusstsein, dass die Community eigentlich so groß ist und der Andrang, der der Hunger nach solchen Materialien und nach so einem Konzepten so groß ist. Diese Erkenntnis auch, dass man mit bestehenden Konzepten oft ansteht und dass es eben so viel diese positive Methoden der positiven Psychologie gibt.

12:51
K: Also das war auch noch eine Sache, dass mir einfach nicht bewusst war, wie froh Leute sind, wenn man da sowas anbietet in der Richtung. Besonders Leute im Lehrbereich, weil ja eben alle haben wenig Zeit, aber es waren trotzdem so viele Leute da, die sich Zeit genommen haben und sich ausgetauscht haben. Das finde ich total schön. Auch länderübergreifend eigentlich.

13:14
B: Das ist wirklich schön zu sehen, dass so großes Interesse im Bereich positiver Psychologie gerade im pädagogischen Bereich ist. Du hast die Expertinnen am Anfang kennengelernt und jetzt auch noch zum Ende gesehen, hast du wahrgenommen, dass sie auch PERMA mehr und mehr in ihren Alltag oder ihr berufliches Leben eingebracht haben?

13:41
K: Das kann nicht schwer beurteilen. Also mit den Experten haben wir halt vor allem zum Beginn viel zusammengearbeitet in der Erstellung der Module, in der Erstellung der Unterlagen. Oder man hat sich ausgetauscht mit Leuten. Wie andere Expertinnen das jetzt konkret einsetzen, das kann ich jetzt eigentlich nicht beurteilen.

14:14
K: Ich weiß nur zum Beispiel, dass die Gabi ihre eigene Schule startet.

14:22
K: Da wird sie PERMA Kur irgendwie einbauen in der Schule. Da hat sich das insofern sicher verstärkt der Einsatz. Ich kann das aber von

anderen echt schwer einschätzen. Die sind ja Expertinnen, weil sie in dem Bereich sowie extrem viel gemacht haben und ob sich das jetzt verstärkt hat oder nicht, da weiß ich nicht.

14:48

B: Ok. Die vierte Frage, welche Schwerpunkte und Ziele wurden bei der Konzeptionierung verfolgt?

15:15
K: Vielleicht kann man das anhand der vier Phasen gut erklären, die wir bei den Modulen immer verfolgt haben, was die fünf Elemente betrifft. Wir haben ein vier Phasenmodell entwickelt. Wir haben es auch teilweise übernommen von der (Chilon?) Grammar school, die haben es ein ähnliches Modell, und zwar heißen die lear it, live it, embed it und teach it.

15:47
K: Und die Idee davon war, dass wir für jedes Element, zum Beispiel nehmen wir positive Beziehungen. Das in learn it Bereich man sozusagen einen theoretischen Background bekommt, was positive Beziehungen ermöglicht. Welche Praktiken führen dazu, dass positive Beziehungsgestaltung möglich ist. Das war learn it. Life it ist wie man Ideen für sich selbst im eigenen Leben umsetzen kann.

16:22
K: Embed it: Wie kann ich das einbauen implizit in den Klassenraum? Und teach it: wie kann ich es auch konkret weitergeben? Zum Beispiel wie kann ich gewaltfrei Kommunikation oder ich Botschaften mit Kindern und Jugendlichen machen? Also das war eigentlich unsere Grundidee, dass die Teilnehmenden sowohl einen theoretischen Background bekommen als auch konkrete Methoden, die sie sofort nehmen können und einbauen können in den Unterricht.

16:49
K: Das war auf jeden Fall mal eine Sache, dass wir all diese Bereiche abdecken. Learn it, life it, emded it und teach it. Und was uns dann auch noch ganz am Herzen gelegen ist, ist eben diese Idee der Haltung zu übermitteln. Also das die Teilnehmenden begreifen, dass perma eben hauptsächlich ein eine Art und Weise ist, mit Menschen und mit Kindern und Jugendlichen zu interagieren.

17:24
K: Und das ist weniger eine zusätzliche Baustelle sein muss, die man irgendwie abarbeiten muss, sondern einfach mehr eine Art und Weise im Unterricht zu stehen. Was waren unsere Ziele?

17:52
K: Ja, einen Werkzeugkoffer, einer Ideenkoffer mitzugeben an Tools aus der positiven Psychologie. Die Lehrer und Lehrerinnen und generell alle Teilnehmenden darin zu bestärken in den positiven Praktiken, die sie jetzt schon anwenden, eben mit dem theoretischen Background. Auch vielleicht eine Art Community zu gründen, dass man sich mit anderen vernetzen kann, sich austauschen kann.

18:20
K: Nämlich über Österreich und auch Grenzen übergreifend!

18:30
B: Gab es bei der Konzeptionierung denn auch Probleme, Unstimmigkeiten oder wart ihr euch da meistens immer einig, was ihr verfolgen wollt und wie ihr das machen möchtet?

18:44
K: Ja, also natürlich hat jeder zu bestimmten Elementen von perma zum Beispiel verschiedene Assoziationen oder verschiedene Dinge, die einem wichtig sind. Zum Beispiel ich weiß, dass bei Engagement am Anfang der Konzeptionierung kam die Frage auf, ob wir bei dem Flowaspekt eingehen oder mehr auf die Stärken.

19:00
K: Also ich glaube, wenn es Probleme gab, dann einfach, dass wir einen gewissen Rahmen hatten, nämlich im Endeffekt zweimal zwei bis zweimal drei Stunden, wo wir die ganze Theorie oder das ganze also das alles rein geben mussten und wir am liebsten das Dreifache genommen hätten und dann müssen wir irgendwie kürzen.

19:38
K: Also einfach die Auswahl war sehr schwierig, was ist jetzt wirklich wichtig? Was wollen wir jetzt wirklich einbauen und wo müssen wir halt irgendwie aussparen, weil sich einfach zeitlich

nicht ausgeht? Vielleicht dieses, wenn man es nennen will, so dieses Spannungsfeld zwischen Quantität und Qualität des Inputs und zeitliche Begrenztheit.

20:01
K: Und dazu entscheiden welche Elemente, welche Inhalte kommen jetzt wirklich in die Module rein? Was ist wirklich extrem wichtig und was können wir auch sparen? Das finde ich war die größte Herausforderung.

B: Also gerne noch mehr Zeit?

K: Ja, also man könnte ja eine zehnmodulige Fortbildungsreihe machen und richtig auf verschiedene Sachen eingehen. Aber dann melden sich halt niemand an.

20:30
K: Also man muss einerseits die Zeit der Teilnehmenden respektieren und andererseits eben auch was hochqualitativ ist unterbringen. Das Spannungsfeld war ein bisschen schwierig.

20:44

B: Wie würdest du deine Rolle im Team beschreiben?

K: Meine Rolle im Team. Ich finde wir waren ein total bereicherndes Team und ich durfte die Rolle einerseits - Also welche Funktionen ich übernommen habe?

B: Ja.

K: Also ich durfte einerseits wirklich beim bei der Konzeptionalisierung dabei sein, wo wir entschieden haben, was kommen jetzt in die Module rein. Und innerhalb der ausgewählt haben.

21:58
K: Ich habe das Design auch erstellt von den Folien, die dann immer wieder umdesignt wurden und so weiter. Aber grundsätzlich so dieses Anfangsdesign kam auf jeden Fall von mir.

21:39
K: Also ich durfte designen, ich durfte konzeptionalisieren, ich durfte Fortbildungen durchführen. Also als Leiterin der Fortbildungen. Ideen sammeln. In allen Phasen sowohl in der Ausführung der Workshops als

auch in der in dem Design der Workshops, in der Design Fortbildungen. Ich durfte bei der permapost mitarbeiten und habe da sehr viele der Emails verfasst. Also eigentlich in allen Bereichen konnte ich mit dabei sein.

22:14
B: Was war dir bei der Rolle besonders wichtig? Also welche Schwerpunkte hast du da für dich persönlich gesetzt?

K: Bei den Aufgaben die ich gemacht habe.

22:27
K: Ja, wahrscheinlich auch, dass die Inhalte auf jeden Fall Qualität haben, dass sie praktisch umsetzbar sind, dass sie durchdacht sind. Mir ist auch Struktur sehr wichtig, dass ganz klar ein roter Faden da ist und das eine mit dem anderen logisch zusammenpasst. Also da weiß ich, dass wir dann oft die Inhalte ein wenig verschoben haben.

22:52
K: Weil mir nicht klar war, warum ist das jetzt da drin ist. Und ich finde das gehört dort. Für mich ist so dieses Gesamtkonzept sehr wichtig. Das alles in sich stimmig ist. Also ich war zum Beispiel auch diejenige die dieses vier Phasen -

23:07
K: Die diese vier Phasen-idee eingebracht hat, weil es einfach mir hilft, deshalb, wenn ich eine Struktur dieses dann. Learn it, life it und so weiter. Ja und Design ist mir auch wichtig, dass es halt optisch nach irgendwas ausschaut. Was war mir im Endeffekt wichtig? Im Endeffekt ist mir wichtig, dass Module kreiert werden also, dass das was dann im Endeffekt rausgeht, Leute anspricht und Leuten hilft und sie dort abholt, wo sie sind und ihnen aha-momente mitgebt, ihnen Inspiration mitgebt, ihnen neues Wissen und neue skills mitgibt. Ja, also im Endeffekt alles, was wir machen zielt darauf ab, dass die Teilnehmenden aus den Fortbildungen mit mehr Inspiration und Wissen und Motivation und Techniken ausgehen, als sie eingegangen sind und ich glaube, das haben wir ganz gut gemacht.

24:11
B: Sehr cool!

24:16

B: Dann ist die Frage, an welchen pädagogischen Methoden und Maßnahmen habt ihr euch während der Konzeptionierung orientiert? Kannst du ja schon ein paar genannt, aber vielleicht noch ein paar weitere.

21:39
K: Eben auf jeden Fall dieses Leute abholen, wo sie sind.

25:00
K: Ich verstehe die Frage jetzt nicht so ganz. Ist damit die Unterrichtsphilosophy gemeint? Kannst du ein Beispiel nennen.

B: Ihr habt doch auch Methoden und Maßnahmen für die Teilnehmenden erstellt, die sie dann im Unterricht verwenden können, oder?

K: Methoden und Maßnahmen also so okay so Übungen, die sie dann machen können. Ja das war uns sehr wichtig. Bei jedem Element eine Art Methodenkiste erstellt mit lauter Ideen, wie man jetzt dieses Element eine hohe gut konkret im eigenen Leben umsetzen kann, aber auch im Leben von den Kindern und Jugendlichen, mit denen man arbeitet.

25:36
K: Also da waren immer im langen Listen von Ideen und da gab es dann auch die Möglichkeit sich eben auszutauschen mit den Teilnehmenden, wo sie auch gegenseitig Methoden miteinander teilen konnten, inspirieren konnten. Also das war unser großes Anliegen, dass sie wirklich aus dem Seminar rausgehen und ganz konkrete Ideen haben. Okay, was möchte ich jetzt umsetzen. Und wir haben sie dann auch zum Beispiel immer nach einem Element gefragt oder nach einem Modul gefragt.

26:07
K: Was ist denn jetzt eine Sache, die du in der nächsten Woche umsetzen möchtest? Damit das eben greifbar ist, das nicht nur so ist, man lässt sich berieseln und man hört im nette Dinge, sondern man setzt dann auch gleich Sachen um und von dem her gab es da auch verschiedenste Kanäle. sowohl eben in den Modulen gab.

26:27
K: In den Modulen hab es diese Inputs und diese Methoden und Übungen. Es gab die permapost mit ganz konkreten Methoden und Übungen zum sofort einsetzen. Es gab den Austausch im StärkenCafé, wo sie gegenseitig Methoden und Übungen erarbeitet haben. Was gab es noch? Es gab das Padlet, dass die Ingrid erstellt hat. Ein Padlet, wo alle Buchstaben und noch mehr links waren und lauter Übungen waren.

26:56
K: Es gibt diese tollen Jugendstärken und jedes Kind stärken Bücher, die dann ausgegeben wurden, wo auch ganz mit - Also es gab so viele Übungen, die sie eben alle mitnehmen konnten und eigentlich alles eben alles kostenlos. Viele haben rückgemeldet, dass sie sehr dankbar waren, darüber, dass sie so viel Materials eben auch bekommen haben.

27:22
B: Würdest du sagen, dass Material ist auch so aufgebaut, dass die individuellen Bedürfnisse jeweils alle abgedeckt werden?

K: Das würde ich sagen, weil es eben so viele verschiedene sind, dass ich einfach jede und jeder die Sachen rausnehmen kann, die zu dem eigenen Schülerinnengruppe passen und zu dem Alter von den jeweiligen Schülerinnen und Schüler. Oder mit den Kindern und Jugendlichen mit denen man arbeitet.

28:47
K: Also ich glaube, weil es eben auch so viel ist und weil so viele verschiedene Methoden und Übungen es gibt. Da wirklich für jeden und jeder was dabei ist und die Lehrkräfte hat dann selbst diese Individualisierung und Differenzierung vornehmen können in ihrem Unterricht.

B: Sodass man selber als Lehrkraft dann die Übungen umändern kann, so dass es passt, meinst du?

28:08
K: Man muss sie oft gar nicht selber umändern, aber man muss halt

auswählen, welche passen. Also wir haben Angebote für verschiedene Altersstufen bzw. manche Übungen, die passen halt besser für ältere und manche, die passen besser für jüngere. Das heißt sie müssen es gar nicht so adoptieren, obwohl das natürlich auch immer geht, aber einfach

aus dem Pool, den wir ihnen zur Verfügung gestellt haben, die auswählen, die zu ihrer Gruppe passen.

28:31
B: Gibt es auch Übungen speziell für Kinder mit besonderen Bedürfnissen sage ich mal körperlich oder geistige Behinderung oder Kinder, die Migrationshintergrund haben und deren Deutsch nicht so stark ist?

K: Also ich glaube das ganz viele Übungen wirklich total inklusiv sind, weil man die machen kann mit geistigen und körperlichen Einschränkungen oder egal von welcher Herkunft und welche Muttersprache.

29:08
K: Ja, also mir fehlen jetzt gerade ganz konkret – finde ich schwierig, was zu nennen, aber so viele Übungen. Allein dieses, was ist gut gelaufen. Ich glaube das kann ebenso viele Arten und Weisen darlegen, dass man das auch mit körperlichen oder geistigen Einschränkungen machen kann. Ja also ich glaube da geht auch wieder darum einfach nur die Richtigen auszuwählen.

29:37
K: Manche Herausfordernissen, die passt dann nicht so. Aber es gibt so viele ganz einfach Übungen, die ich eben auch schon im Kindergarten machen kann. Aber auch mit 18-Jährigen machen kann. Also ich glaube da ist für alle was dabei.

B: Auf einer Skala von eins zu bis zehn wie zufrieden warst du mit der Qualität der Schulungsbausteine?

30:01
K: Ich würde sagen acht.

B: Okay dann die nächste Frage, was hat dir gefehlt für eine 10? K: Was mir gefehlt hat für eine 10? Ja, noch mehr Zeit.

30:24
K: Noch mehr Zeit für konkrete Übungen, die man irgendwie gemeinsam machen kann. Es ist mir – Mir waren es manchmal vielleicht zu viele Listen von Ideen und ich probiere dann gerne Dinge halt gleich aus oder dass man mehr in die Praxis geht. Das heißt man könnte - Wenn man jetzt einfach alle Ressourcen zur Verfügung hat und einfach vielleicht noch paar Stunden dranhängen kann und theoretisch, dass das dann für alle das trotzdem alle kommen würden.

30:51
K: Dann könnte man mit mehr Zeit und mehr dann mehr Inhalten, die man füllen kann. Hätte ich es eine 10 bekommen. Also ist hauptsächlich so dass Sachen halt rausgenommen wurden, die ich halt

gerne noch, wenn ich jetzt nur für mich machen würde, würde ich sie noch reingeben. Aber es war super.

31:12
K: Ich glaube, wir haben mit den Ressourcen, die wir hatten, haben wir wirklich, was Großartiges geschaffen und wenn man noch ein bisschen mehr Zeit hat, noch ein bisschen mehr Ressourcen. Vielleicht könnte man es noch auf 10 von 10 bringen.

B: Welcher Schulungsbaustein hat dir besonders gut gefallen? Welcher schulungsbaustein hat dir besonders gut gefallen?

31:46
K: Ich würde sagen der Erste, weil da so viele Themen für mich drinnen sind, die ich so wichtig finde. Zum Beispiel am Schluss das selbst Mitgefühl, aber auch diese Stärkenorientierung, die positiven Emotionen. Ich glaube den ersten habe ich, also von der Seite der Vortragenden, habe ich den ersten, das Modul 1, am liebsten gemacht.

32:11
B: Wie hast du für dich das Stärken-café wahrgenommen?

K: Gemischt, also einerseits sehr inspirierend. Also so von den Inhalten, die gekommen sind. Sehr inspirierend, was die Kolleginnen und Kolleginnen schon umsetzen und welche tollen Ideen sie haben und das fand ich sehr inspirierend. Ich fand es einfach so von der präsentierenden Seite anstrengen, weil man muss immer so aufmerksam zuhören und dann halt irgendwie noch Fragen und darauf reagieren.

32:49
K: Und wenn dann 12 Gruppen präsentieren, dann bin ich am Schluss - dann kann ich gar nicht mehr authentisch mich freuen mit denen, sondern es hat mich ausgelaugt, deswegen fand ich das Stärkencafe gemischt. Es hat mich ausgelaugt als Vortragende, aber es war total schön zu sehen, was die Leute schon umsetzen, also deswegen war es gemischt für mich.

33:15
B: Und wie - Hattest du für dich selbst auch Präsenzveranstaltungen oder war alles online bei dir?

K: Ich hatte auch Präsenzveranstaltungen.

B: Wie hast du die wahrgenommen?

K: Sehr positiv, also da hatte - wie viel hatte ich dann so? Circa vier oder so? Vier bis fünf waren präsent. Und das habe ich schon sehr schön auch gefunden, dass - also wie dieser Austausch ein bisschen anders funktioniert, wo Leute sich eher trauen, was zu fragen oder was zu sagen.

33:44
K: Also das ist halt immer so. Wenn man persönlich ist, dann ist die Hemmschwelle eine Frage zu stellen oder einen Kommentar zu machen.

Also live ist das niedriger, als wenn was online macht. Das fällt auf jeden Fall auf.

34:05
K: Ich finde schon, dass das halt sehr viel hat, wenn ich Leuten in die Augen schauen kann und mit ihnen halt irgendwie mehr reden kann als online. Wenn ich online bin, dann sehe ich in Wahrheit eigentlich keine Geschichte, sondern ich sehe nur Slides, in die ich rede.

34:23
K: Und das gefällt mir dann schon einfach persönlich noch besser. Diese Connection, die man mit Leuten kriegen kann, aber vom logistischen her ist einfach online viel besser. Ist halt einfacher. Also wenn ich mich daheim hinsetze und auch drehe, muss nirgendwo hinfahren und so weiter. Also es hat beides vor Nachteil.

34:49

K: Ich glaube wir hätten niemals so viele Leute erreichen können, also einem Bruchteil hätten wir erreichen können, hätten wir es Präsenz gemacht. Deswegen finde ich super, dass wir es online angeboten haben und dass wir eben die Schulen, mit denen wir auch ganz eng zusammengearbeitet haben mit ein paar mit dem Pilotschulen. Da waren wir eh dann zumindest einmal vor Ort und auch bei teach for Austria war ich vor Ort. Das war total nett.

35:14
K: Hat beides seine Vor- und Nachteile. Und so wie es war, hat es gut gepasst.

B: Und wie geht's jetzt für dich weiter? Wie sieht es für dich in der Zukunft aus?

K: Ja, es ist tatsächlich das ist sehr intensiv weitergeht, weil ich werde ab nächstes Schuljahr bei der KPH Wien Krems. Ich habe da zehn Stunden, werde ich eingestellt, um auch um PERMA.teach eben auf institutionalisierte Art und Weise weiterzuführen.

35:52
K: Also ich weiß jetzt schon, dass ich eben über die KPH Wien Krems nächstes Jahr drei - wieder zu diesen Seminaren geben werde. Ich werde auch die Seminare geben gemeinsam auch mit anderen ExpertInnen. Bei der PH Kärnten und PH (Oberstreich?) ! Und aber auf jeden Fall dieselbe Modulreihe, werden wir nächstes Jahr in andere Institution auf jeden Fall durchführen.

36:26
K: Und ich werde auch im Sommer - gebe ich so ein zwei-Tage train the Trainer Event für drei bis zehn Frauen aus Tschechien, die ein ähnliches Konzept in Tschechien verwirklichen wollen und da bin ich eben zwei Tage in Brünn mit denen und gebe halt das Konzept weiter. Also es tut sich viel und ich glaube, dass da noch viel Großes raus werden kann.

36:59

B: Es wächst und wächst.
K: Ja, das Bedürfnis ist auch da.

B: Dann die letzte Frage: Ob du noch irgendwas mitteilen möchtest? Noch irgendwas zu dem Projekt oder aus seinem privaten Leben zu dem Projekt sagen möchtest du, gibt da irgendwas?

K: Dass ist eine Bereicherung für mich war. Dass ich mich auch persönlich sehr weiterentwickeln konnte, dass ich total dankbar bin Teil dieses Projekt sein zu dürfen und dass mir da auch so viel Vertrauen geschenkt wurde von der Seite der Projektleiterinnen.

37:28
K: Dass ich so viel machen durfte. Das finde ich voll. Dankbarkeit und Reichtum und ich bin froh, dass ich dabei sein könnte!

B: Sehr schön. Das ist ein runder Abschluss würde ich sagen. Das waren alle meine Fragen.

6. Interviewtranskription von Elke Poterpin (22.06.2023 um 10:00 Uhr)

00:00
B: Wie sind Sie auf PERMA teach aufmerksam geworden?

P: Vielleicht muss ich ganz kurz zu meiner Person sagen: Ich bin gelernte Volksschullehrerin. Vielleicht ist das auch interessant. Ein bisschen. Habe von 1988 bis 1991 (Peter Claas?) gemacht. Und war dann 22 Jahre lang oder über 20 Jahre in einer Wiener Ganztagsschule. Und beschränkte Form und habe in meinen ersten Dienstjahren Psychologie studiert.

00:30
P: Und in dieser Zeit. Das war dann in den ja in den 90er Jahren war von positive Psychologie noch kein Rede und als ich dann aber vor zehn Jahren an die Pädagogischen Hochschule Wien gewechselt bin und dann auch mich forschungsmäßig und wieder eingearbeitet und lesen habe. Bin ich dann eben auf die positive Psychologie gestoßen und auf das PERMA Modell. Ich habe dann sehr schnell bei der Auswahl für die Inhalte meiner Vorlesungen und so weiter -

00:56
P: War mir klar, dass als Grundlage für Lehrpersonen so ein theoretisches Konzept eigentlich - wir haben nicht viel Zeit in diesen Vorlesungen. Da muss man eine Auswahl treffen und da habe ich mich dafür entschieden hier einen ganzen Block sozusagen diesen positiven psychologische Theorie - , die zu vermitteln.

01:20
P: So als Grundlage, die ich dann immer wieder auch in der Lehrerinnen Ausbildung aufgreifen. Das heißt das habe ich, mache ich schon seit zehn Jahren und bin bekannt mit der Ingrid Teufel, die in dem PERMA teach Modell tätig ist.

01:37
P: Und sie wusste von meiner Tätigkeit. Sie wusste von meinen Inhalten und als sie dann diese Ausbildungen gerade gemacht hat so positive Psychologie und sich gemeinsam mit der Eva das Ziel gesetzt hat: Wir wollen hier dieses PERMA.teach ins Land tragen. Dann ist sie auf mich zugekommen und hat mir davon berichtet und mich gefragt, ob ich da einfach gerne noch mitarbeiten möchte und ich habe natürlich unterstützt.

02:03
B: Und was genau war ihre Motivation an der Teilnahme von PERMA.teach? Ihre persönliche Motivation?

P: Ich erreiche natürlich jetzt in meinen Vorlesungen, wo alle Studierende der PH Wiener sozusagen durch meine Hände gehen, erreiche ich schon eine ganz große beachtliche Zahl. Wir haben im Jahr um die 400 Studierende. Und aber natürlich diese Chance zu sehen, dass hier österreichweit und vielleicht sogar darüber hinaus,

02:37
P: dieser Inhalt sozusagen die Breite findet, um letztlich mit der großen Vision dieses Wohlbefinden im schulischen Handlungsfeld immer

mehr ankommen zu lassen. Also das kling dann immer so banal. Positive Visionen, Klassenklima, Beziehungen. Und was man machen soll, das wissen wir ja eigentlich. Wenn man aber in die Schulpraxis schaut – ich

betreue auch Praxisgruppen. Dann erlebt man eben im Alltag genau nicht das. Und man liest in Studien von Belastungen von Schülerinnen und Schülern, aber auch von Lehrpersonen. Also von allen Beteiligten im Bildungssystemen. Und nachdem die psychosoziale Gesundheit ein Schwerpunkthema von mir ist und mir die Wohlbefindeskonzepte auch zugrunde liegen.

03:29
P: Da haben wir eine Schnittstelle! Und auch meine Dissertation in diesem Bereich gelegen hat Emotionsregulation und so weiter. Ja, es ist einfach eine Herzensangelegenheit, weil ich einfach ganz viel Sinn darin sehe und das Gefühl habe, wenn das noch mehr gelebt wird, kann Schritt für Schritt das Schulsystem, aber vor allem die Gesellschaft insgesamt dadurch bereichert werden.

04:04

B: Welche Veränderungen haben sie durch das Entwicklungsprojekt bei den TeilnehmerInnen festgestellt?

P: Meinen Sie die Teilnehmenden der Projektgruppe?

B: Nicht die ExpertInnen. Sie gehören ja zu den ExpertInnen, sondern die Teilnehmenden.

P: Aha, wenn wir Fortbildungsveranstaltungen gehalten haben. Ja, da muss ich jetzt ehrlicherweise sagen, da war ich ja natürlich nur punktuell vor Ort.

04:33
P: Also ich habe während dieser Module von mir. Da sehe ich jetzt Bilder vor mir und Szenen vor mir sehr viel Offenheit, sehr viel Neugier, sehr viel positive Rückmeldung der Lehrpersonen erfahren und erlebt im Sinne von auch zum Teil Transfer Erkenntnisse. A eigentlich mache ich schon längere Zeit dieses und jenes und eigentlich passt das dazu, das war mir nur nicht bewusst, dass das irgendwo da so eine passen könnte. Bis hin zu Ambitionen von ganzen Standorten, die versuchen in irgendeiner Art und Weise etwas zu etablieren.

05:07
P: Also ich habe das dann bei den Stärkencafes am Schluss auch letztlich die Schulstandorte präsentieren durften, konnten. Was sie sozusagen für Ziele gesetzt haben, wie sie was umgesetzt haben. Da habe ich dann schon gemerkt, dass da etwas angekommen ist, dass Kolleginnen und Kollegen, kann aber nicht abschätzen, ob das die Breite dann in einem Kollegium ist oder ob das einzelne engagierte Leute sind.

05:36
P: Da muss ich sagen da fehlt mir der Einblick. Da müsste man

speziell die Personen ansprechen und nachfragen, was bei ihnen selbst sozusagen das ausgelöst hat oder obs nachhaltig war. Mein Wunsch wäre natürlich und das ist auch die Projekteidee an sich: nachhaltig dranzubleiben, immer wieder die Themen in Erinnerung zu rufen. Ich kenne das noch aus meiner Lehrerinnen Zeit. Man kommt doch sehr motiviert und begeistert aus irgendwelchen Fortbildungen.

06:01
P: Und nimmt sich einiges vor und dann verschlingt einen wieder so der Alltag. Und dann geht’s ein bisschen in Vergessenheit, daher glaube ich es notwendig da auch immer wieder Impulse zu setzen. Was das Projekt

auch macht, über Newsletter und über diverse Vielschichtigkeit. Man versucht ja genau das auch bestmöglich zu erreichen.

06:33
P: Jetzt habe ich die Frage wieder vergessen. Wie war die nochmal?

B: Wie sie Veränderungen bei den Teilnehmenden wahrgenommen haben.

P: Wie gesagt ich traue mich da kein großes Urteil abgeben, was da bei den einzelnen Individuen sich sozusagen an Entwicklung vollzogen hat. Aber wie gesagt die Rückmeldungen vor Ort. Während und nach so einer Fortbildung oder nach so einem Modul war immer sehr positiv. Ich glaube alleine schon, dass die Inhalte in einem Bereich gehen, der sonst sehr selten angesprochen wird.

06:57
P: Es geht oft um fachliches. Es geht oft um didaktische. Es geht oft um methodisches und hier geht's mal um persönliches und emotionales auch und letztlich auch um das Wohlbefinden der Lehrpersonen selbst und nicht nur das der Kinder. Lehrerinnen und Lehrer kümmern sich ja sehr stark um andere und vergessen manchmal ein bisschen Selbstfürsorge auf sich selbst und das vielleicht, das ist jetzt interpretativ, natürlich ja, also wir müssen da -

07:22
P: Aber ich glaube es wurde ja eine schriftliche Erhebung durchgeführt bei den TeilnehmerInnen. Ich nehme an diese Frage kann man da besser ablesen. Das wären jetzt Spekulationen.

07:31
P: Ich vermute und ich hoffe – ein subjektiver Wunsch ist es natürlich meinerseits. Es ist mit Vorsicht zu genießen diese Aussage, dass bei dem einen oder anderen, oder der einen oder anderen einen Impuls geweckt hat, der leben möchte und der, wie so ein kleines Pflänzchen gedeiht und wächst und im besten Fall im Multiplikatorinnen System einen Dominoeffekt weitergeht (an Kolleginnen, die was erleben und beobachten?) Das habe ich so bei diesen Veranstaltungen als die Rückmeldungen kamen, was sie denn umgesetzt haben, ein bisschen so den Eindruck gehabt, dass da dann immer wieder Kollegenschaft etwas sieht und fragt, was machst du da, und diese sehr niederschwellige Schiene bei Themen, wo sie Haltung finden. In erster Linie geht es ja um eine Haltung (...?).

08:27
P: Möglicherweise auch, wie es herum geht. Im positiven Sinne, dass man infiziert.

B: Also, dass die Lehrkräfte selber zu Multiplikatorinnen werden, um die Haltung mit ihrem eigenen Kollegium zu teilen?

P: Genau bewusst und unbewusst. Ich glaube es könnte dann solche die sagen: Ja das finde ich so spannend, da gehe ich noch weiter in eine Weiterbildung, Fortbildung, Weiterbildung und möchte das Schwerpunkt setzen. Manche sagen ich nehme das jetzt einfach als Jahresschwerpunkt für mein für meine Klasse oder überhaupt für meine ja - oder und anderen erleben es vielleicht eher unbewusst oder -

09:09
P: Oder ja und setzen einzelne kleine Übungen um, kleine Rituale, erste Schritte und möglicherweise über den Austausch kommen dann andere, die geben selber als gar nicht in der Fortbildung vielleicht direkt dabei waren. Ja auch in diesem Bereich in unsere PERMA.teach Gruppe und ich hoffe und ich denke auch es werden immer mehr.

B: Haben Sie vielleicht auch von einzelnen Lehrkräften erfahren, ob sich bei den persönlich im privaten Leben was verändert hat durch die Teilnahme beim PERMA.teach Projekt?

09:47
P: Auch da muss ich wieder sagen, dass dadurch, dass ich jetzt mit den unterschiedlichsten Kolleginnen und Kolleginnen und Kollegen Wohlfortbildung gehalten haben, manche war ja auch online. Und manchmal hat man dann nicht keine zweite Begegnung eigentlich so dass so eine Entwicklung - Das wäre jetzt unseriös. Hier eine Entwicklung zu skizzieren also das kann ich leider nicht beantworten.

10:12

B: Dann können Sie aber vielleicht was zu der Veränderung innerhalb von dem Expertenteam und bei sich selbst durch das Entwicklungsprojekt sagen.

10:29:
P: Fang ich da mal bei mir selbst an. Also ich habe gespürt, das war jetzt glaube ich eine der schönsten Dinge überhaupt, dass wenn man in so einem Kreis arbeiten darf, wo sämtliche Teilnehmerinnen und Teilnehmer genau von dieser Idee überzeugt sind, sonst wären sie vermutlich nicht dabei, dann hat arbeiten in so einem Team eine unglaubliche Atmosphäre und es war jedes Mal wunderschön.

10:59
P: Ich habe immer wieder im Kalender gesehen und wir haben wieder einen Arbeitsmeeting egal ob digital oder persönlich. Da war immer Freude. Ich würde es gar nicht arbeiten nennen sondern es war Austausch mit Gleichgesinnten, die alle dasselbe Ziel anstreben oder ein ähnliches oder in die Richtung gehen, weil sich eigentlich alle diese fünf Säulen in diesem tun sozusagen auch wieder gespielt haben und fühlbar waren, erlebbar waren.

11:28
P: Und das hat es insofern erstens einmal natürlich meinen Arbeitsalltag wahnsinnig bereichert. Einerseits erfreut es meine positiven Emotionen. Mein Wohlbefinden hat sich durch die Mitarbeit verstärkt. Den Sinn drin zu sehen, erfolgreich zu sein, wenn man spürt, es kommt was an. Sozial eingebunden zu sein in diese Expertinnengruppe. Auch das Engagement der anderen zu sehen und immer wieder zu sehen, was andere in der Gruppe weitergebracht und welche kreative Idee sie hatten oder wo was umgesetzt haben. Das ist so eine Aufwärtsspirale, die sich da dann auch in einem selbst stärker integriert hat.

12:10
P: Eine Wechselwirkung, die einfach auch mein Wohlbefinden bestärkt hat so mit dem Bedürfnis, es muss in irgendeiner Form weitergehen, weil das würde mir fehlen. Also diese Art der Zusammenarbeit. Also es ist einfach schön zu sehen, dass tatsächlich diese Gedanken, diese Einstellungen, diese Haltungen, tatsächlich für einen selbst und sein eigenes psychosoziales System einfach wirken in positiver Art und Weise.

12:38

P: Ja eigener Erfahrung, die das für mich auch noch inhaltlich untermauert hat (...?) und in der Gruppe, es ist immer schwierig für andere Aussagen zu treffen, aber allein anhand, wenn man es jetzt vielleicht qualitativ betrachten würde. Die E-Mail-Texte, die ausgetauscht werden. Die Art, wie häufig kommt es da zu einem Danke, zu einer Wertschätzung, zu einem – würde man alleine die zur Kommunikation jetzt hier analysieren, würde man schon merken, dass wir hier auf einem - ja es auch gelebt wird, also von all diesen ExpertInnen, die da hier in diesem Raum waren.

13:22
P: Ich habe den Eindruck sehr verinnerlicht schon ja also gar nicht so sehr: Ah wenn man die positiven Emotionen, Dankbarkeit ausdrückt, dann ist das ein beforschtes, sinnvolles Tool, sondern ich habe das Gefühl das ist in dem Personen, die hier tätig waren, schon so inhärent, schon so in der Persönlichkeit, in die Haltung integriert, das dieses Tun schon automatisiert abläuft mit eben diesem Hintergedanken des PERMAs.

13:46
B: In welchen Lebensbereichen würden sie sagen, haben sie besonders eine Veränderung wahrgenommen?

P: Bei mir selbst? Ja? Lebensbereiche, sie meinen jetzt Familie, Freizeit, Freunde, Beruf und so weiter.

14:14
P: Ich würde sagen, nachdem ich schon sehr lange dieses Thema in mir trage. Ich würde sagen seit 2008 ungefähr war das so ein Schlüsselereignis, wo ich begonnen habe in diese Richtung zu denken, zu tun. Das ist jetzt doch schon 15 Jahre und daher war schon vorher viele etabliert und verändert. Vielleicht eben wie gesagt das berufliche tun in beruflichen Bereich vielleicht noch am stärksten.

14:42
P: Weil es mir noch einmal bestätigt hat, dass ich glaube, in einer guten Auswahl zu sein, bei meinen Themen, aber auch was die Studierenden betrifft. Und vielleicht auch, weil ich natürlich auch durch diese Arbeit viel Material für mich und für meine Studierenden dazugewonnen habe. Es ist ja auch viel entwickelt worden in diesem Bereich und das ist einfach auch wunderschön zu sehen, wenn viele an etwas arbeiten, was dabei in einem Jahr oder in kurzer Zeit eigentlich oder mittelfristig zustande kommen kann.

15:19
P: Diese wunderschönen Grafiken auch, die dann da auch entstanden sind. Also ich kann natürlich im beruflichen Bereich zu PERMA noch PERMA.teach sozusagen dazugeben. Auch in Lehrerinnen Fortbildungen habe ich natürlich schon, also in allgemeinen Fortbildungen, gar nicht aus der PERMA.teach Reihe, also zu einem anderen Thema, konnte man es immer wieder einfügen. Also ja ich würde sagen im beruflichen Bereich hat es mich am meisten inspiriert, was aber natürlich naheliegt, weil es ja auch um teach geht. Aber auch privat insofern, denn mein jüngerer Sohn lernstudiert für Sekundarstufe und ist natürlich dann auch, wenn man das jetzt privat nennen will, teilberuflich sozusagen. Mit ihm spreche ich auch über solche Themen, da er ja auch in der Haltung, in der Mutterhaltung, großgeworden ist. Und auch ganz spannende privaten Bereich zusammen zu reflektieren, wenn jemand dann auch in diesem Beruf geht und es als Kind erlebt.

16:16

P: Und wie geht's mir jetzt damit? Und ja, für sich einfach auf die Erkenntnis, dass es für ihn eine ganznormale Haltung ist, die eigentlich selbstverständlich ist und erstaunt manchmal, dass er von Mentorinnen und Mentoren genau diese Rückmeldung bekommt. Wie wertschätzend er mit den Schülerinnen umgeht, wie positiv, und so weiter.

16:47
P: Das wird mir erst jetzt während des Sprechend bewusst, dass das einen schönen Outcome (...?) Noch bewusster, noch ein Stück mehr bewusster zu leben und auch weiter zu tragen. Privat, wie beruflich.

B: Schön, dass es auch so familiär gelebt wird.

P: Ja auf jeden Fall. Da gibt es auch ein Gespräch mit meiner Tochter, die die Wirtschaft studiert hat und ganz in einem anderen Bereich tätig ist und wo sie aber auch sehr kritisch natürlich gewisse wirtschaftliche Strukturen betrachtet und sieht. Soziale und emotionale Aspekte vermisst und wieder versuchen diesen Transfer herzustellen.

17:27
P: Was würde denn das auch in der Wirtschaft bedeuten? In Human Ressource. Oder wie gehen eigentlich Firmen mit ihren Mitarbeitern um? Wie gehen Kollegen untereinander um? Wie ist man denn da eingebettet ins Team? Wie stark sehe ich den Sinn in meiner Tätigkeit? Ja und und... also Ja, also das ist so gesehen auch für

mich wahnsinnig spannend! Ich habe drei Kinder, jetzt muss ich den Dritten auch noch erwähnen. Mein ältester Sohn ist im Gesundheitsbereich. Der ist Physiotherapeut und Medizintechniker. Und auch das ist natürlich ein Bereich, wo PERMA genauso, wie eigentlich ja in jedem Lebensbereich, wenn man so will, unglaublich wichtig ist.

18:09
P: Nachdem wir in der Schule diese wunderbare Gelegenheit haben Schülerinnen und Schüler zu begleiten, ein Stück weit ihnen solche Themen hinzugeben, habe ich halt die große Vision, die große Hoffnung, ich bin da extrem optimistisch, dass diese Kinder und Jugendlichen, das dann in all diese unterschiedlichsten auch Arbeitsbereiche und Lebensbereiche und privaten Bereiche immer mehr hineintragen können.

18:37
P: Also ich denke das jetzt ganz groß. Ich traue mich da jetzt mal ganz groß zu denken. Wir tragen da zu einer wirklichen Weiterentwicklung der Gesellschaft bei. Regional, hoffentlich dann irgendwann auch mal international, aber da tun ja Gott sei Dank andere Gruppierungen in anderen Ecken der Welt ja auch einiges. Australien, Amerika. Es gibt ganz engagierte Gruppierungen. Also wir müssen ja nicht von hier aus die gesamte Welt bedienen.

19:03
P: Aber ich denke mir so Stück für Stück führt das, denke ich, insgesamt zu einer besseren Welt, was auch immer man darunter sich vorstellen mag.

B: So wird die Vielfalt von PERMA auch nochmal sehr bewusst. Also es ist wirklich ein allen Bereichen, wo Menschen agieren, benutzt wird.

P: Und vielleicht, weil auch immer wieder dieser Kritikpunkt aufkommt, der auch okay ist und auch notwendig ist. Den ich dann immer wieder versuche zu diskutieren und auch zu relativieren. Da kommt dann oft der

Begriff der Kuschelpädagogik. Es geht ja nicht darum, dass die da sich nur wohl fühlen.

19:41
P: Die sollen ja auf die harte Realität, auf die Wirklichkeit vorbereitet werden. Eben diese Realität in der Wirtschaft mit all den Bedingungen. Ich sag dann immer mal, drehen wir den Spieß um. Und ja, wie wäre es denn, wenn sich dort was verändert. Und vor allem, was wäre denn, wenn Menschen dort hineingehen mit einer anderen Haltung und ja vielleicht kann man dann auch dort ansetzen.

20:08
P: Also Menschen so auszubilden, dass wir sie so erziehen oder in Haltungen bringen, dass sie möglichst alles, was schwierig ist in dieser Welt auch aushalten. Im Sinne der Selbstoptimierung halte ich für den falschen Zugang. Ich glaube auch, dass selbst mit PERMA.teach und PERMA erleben Kinder und Jugendliche ja trotz allem in der Schule immer wieder auch Stress und Herausforderungen und

Frustration. Nicht immer gelingt alles gleich und es geht ja tatsächlich auch in unserem Konzept, in unserem PERMA-Denken auch den Kindern sozusagen alle Hindernisse aus dem Weg zu räumen.

20:55
P: Ja, lernen ist anstrengend und bedeutet intensives Engagement und Energie, die man da hineingibt und bedeutet eben auch ja auch schwierige Emotionen manchmal. Manchmal bin ich traurig, wenn wir was nicht gleich gelungen ist manchmal bin ich irritiert, weil mein Sitznachbar schon viel weiter ist als ich, oder oder. Das passiert ja trotzallem.

21:18
P: Und die Frage ist aber, wie gehe ich dann mit Stressfaktoren um und da denke ich ist PERMA eine ganz großartige Antwort oder eine begleitende Schiene. Das mit Kuschelpädagogik gleichzusetzen, halte ich für absolut nicht zulässig.

B: Dankeschön. Bei der vierten Frage geht es darum, welche Schwerpunkte und Ziele Sie bei der Konzeptionierung verfolgt haben im Expertinnen Team.

22:11
P: Die theoretische Grundlage liegt auf der Hand. Das PERMA Modell an sich, das schon die grundlegende Struktur vorgibt. Als ich dazugestoßen bin, war schon die Idee der Hand da. Ich glaube, das war von den Urgründerinnnen schon ein kreativer Ansatz. Was sehr schön ist, wenn wir – Hand, Herz, Hirn, diese Kombi, dieser ganzheitliche Blick auch sehen und gleich auch einen Anker für die anderen bieten kann auch als Merkhilfe. Also das war sozusagen schon gegeben. Diese Struktur war klar und dann kam so diese Überlegungen hinzu: Welches didaktische Konzept soll uns eigentlich hier durchleiten, durch diesen Prozess, so dass es auch sehr transparent und nachvollziehbar für Kolleginnen und Kollegen wird?

22:48
P: Kolleginnen und Kollegen, die hier aus dem Bereich sind, aus dem pädagogischen Bereich, und dann natürlich auch Ihre Erwartungen Ansprüche zurecht haben und eben auch um den Inhalten noch effizienter sozusagen zu transportieren und da kam dann dieses vierstufige didaktische Konzept. Learn it, life it, enbed it, teach it.

23:13
P: Dieses didaktische Konzept war dann nicht gleich von Anfang an, also wir haben da mal begonnen und ein bisschen noch zu ungleich erschienen und dann haben wir das noch drübergelegt und ich glaube das hat dann sozusagen zu den inhaltlichen Strukturen, oder über die didaktische Struktur gelegt, wie man möchte und das war eigentlich der Hauptfokus. Alles andere waren dann Überlegungen, die stark natürlich in die Richtung gingen.

23:53
P: Wie viel theoretisches hintergrundwissen brauchen die Teilnehmerinnen und Teilnehmer, um schließlich den Draht praktischen Transfer gut nachvollziehen zu können, sozusagen. Also waren schon

wichtig, dass Sie gewisse Grundlage halten, aber das haben wir sehr (...?). Wir haben das natürlich auch verwiesen auf Studien auf Begründungen oder aus meiner Sicht auch auf psychologische Hintergründe wie (...?) oder selektive Wahrnehmung. Solche Erkenntnisse der Grundlagenforschung sozusagen, aber wie gesagt das würde immer nur dann Kurz erklärt, um sozusagen die Bedeutung des Transfers klarzumachen und verständlicher zu machen.

24:46

B: Und wie, haben sie ja schon ein bisschen dazu gesagt, aber wie war allgemein ihre Rolle im Team?

P: Meine Rolle im Team. Ja also ich würde mal sagen, ich denke, dass ich in meiner Rolle als Hochschullehrende der Pädagogischen Hochschule Wien mit Erfahrung eben vermitteln von diesen Inhalten an Studierende dazu geholt wurde und ich würde mal meinen, dass meine psychologische Ausbildung in Kombination mit meiner pädagogischen Ausbildung wahrscheinlich jetzt der Hauptfokus war.

25:22
P: Ich habe mich auch drin gesehen genau auf diese theoretischen Inhalte hinzuweisen, dass ich das die vielleicht Sinn machen können einzubringen. (...?) da war einiges schon vorhanden. Auch so an kleinen YouTubes und kleinen Erklärungstools.

25:51
P: Ja und das war dann sozusagen - Ich habe meine Rolle dann so gesehen, dass ist eben aus der psychologischen Perspektive ein bisschen noch zu fundieren. Im Austausch auch - Und andererseits eben im Austausch mit manchmal zweien, dreien oder auch größeren Teams. Wir haben uns verschiedene Aufgabengebiete dann eben manchmal den kleineren Runden oder größeren Runden getroffen und über den Austausch gemeinsam -

26:21
P: Zu hinterfragen, wo wir da gerade stehen und was es noch braucht. Sozusagen auch als critical friend manchmal, wenn jemand schon etwas vorentworfen hat noch mal den Blick darauf zu machen oder eigene Ideen von den anderen feedbacken zu lassen, wie sie das sehen, also es war ein sehr gemeinschaftliches hinein tun!

26:46
P: Ist meine Rolle klar geworden?

B: Sie können ja nochmal sagen, was ihnen besonders wichtig war oder besondere Schwerpunkte, die sie selbst für sich hatte in ihrer Rolle.

P: In meiner Rolle, meine Schwerpunkte. Erstens mal gut zuzuhören den anderen, was sie aus ihren Erfahrungsbereichen da bereits mitgenommen haben, jeder kommt ja von einer anderen Vorerfahrung mit anderem Vorwissen.

21:17
P: Sehr spannend fand mich auch von meiner Kollegin, die da diese

Sekundarstufenperspektive dazu gebracht hat. Ich selbst komm ja aus der Primastufe. Da habe ich aber auch ein Seminar zu gemacht zum Thema Lernen und Lernen und habe da PERMA eingebracht. Ich fand das Lernen von den anderen ganz spannend. Das würde mir noch zu der ersten oder zweiten Frage einfallen. Das war auch ein Mehrwert, den ich herausgezogen habe. Es kam auch immer von anderen dazu. Das war wunderschön und bereichernd. Also das Zuhören und dann vielleicht manchmal, ich glaube ich setze mich so ein, dass ich jemand bin, der Dinge ganz gut auf den Punkt bringen kann. Manchmal auch sprachlich dann formulieren kann und vielleicht manchmal gehört es dann noch (...?) einmal zusammengefasst habe. Meinst du das so?

28:09
P: Aber ich glaube ich bin noch jemand, dass ich mir auch immer wichtig, dass wir im Prozess gut vorankommen. Also, dass wenn ich das Gefühl habe, es driftes jetzt ab oder es zieht sich ein bisschen oder das ist jetzt zu detailliert oder so. Vielleicht auch wieder hin und wieder mal das zu beobachten, wie reicht es jetzt noch, was dazu gehören könnte oder noch bereichern sein könnte oder vielleicht auch einmal die Idee zu haben wieder zurückzufinden.

28:38
P: Auch ist mir wichtig im Prozess dann zu besprechen, wie ist der nächste Schritt. Was haben wir heute vereinbart oder was hat uns weitergebracht? Weiß jeder von uns seinen nächsten Schritt? Solche Arbeitsschritte effizienter machen. Das klingt jetzt wieder so ökonomisch, aber ich mein wir haben alle unsere Arbeitszeit und unsere Tätigkeiten und deshalb war es mir ein Anliegen, dass man nicht so vor sich hin plaudert, auch wenn das auch mal schön ist.

28:58
P: In diesem Kontext, aber ich bin jetzt auch nicht die Einzige, die darauf geachtet hat. Das haben immer alle sozusagen. Sehr auf Augenhöhe, aber es gibt natürlich auch immer solche in einem Team und das ist so wertvoll. Die unterschiedlichen Stärken mitbringen. Und sind die einen halt, die da total kreativ beseelt sind und dann passiert halt manchmal, dass man total in seiner Kreativität nicht gespannt kriegt und umgekehrt natürlich auch.

29:28
P: Der Blick auf den Gesamtprozess, das würde ich so sehen. Das Lernen und Zuhören, ja und da oder dort diversen Stellen, wo ich das Gefühl habe, da fällt mir jetzt auch was sinnvolle ein, um meine Gedanken einzubringen.

B: Schön, das hat es auf den Punkt gebracht.

30:01

B: Dann die nächste Frage. An welchen pädagogischen Methoden und Maßnahmen haben sie sich während der Konzeptionierung orientiert?

P: Pädagogische Methoden und Maßnahmen? Müssen wir mal Methoden und Maßnahmen definieren. Ist das für Sie was Unterschiedliches? Müssen wir diese zwei Begriffe definieren. Sind Methoden und Maßnahmen für Sie etwas Unterschiedliches?

B: Gute Frage! Jetzt dürfen sie frei entscheiden.

30:52
P: Das sind gar nicht so einfach Begriffe, die wir da verwenden. Bleiben wir bei den Maßnahmen. Vielleicht ein allgemeinerer Begriff als Methoden, denn bei Methoden müssten wir in der pädagogischen Literatur hineinschauen. Nachdem ich jetzt nicht Erziehenschafterin bin, würde ich lieber allgemeine Maßnahmen nennen. Ich habe zwar als Lehrperson meinen Methoden Pool, aber das ist jetzt anwendungsorientiert.

31:09
P: Pädagogische Maßnahmen. Mir persönlich ist immer erfahrungsbezogenes und handlungsorientiertes Lernen auch ganz wichtig. Nicht nur mir, da waren wir uns in der Gruppe auch einig. Das wir unbedingt bei jedem Themenbereich etwas Erfahrbares, Erlebbares den Teilnehmerinnen und Teilnehmern mitgeben. Sprich ganz konkret eine, zum Inhalt passende, Übung, etwas wo eben Selbstreflexion oder Selbstwahrnehmung zunächst - was spüre ich überhaupt, wie bin ich heute da? Kann ich das überhaupt benennen? Habe ich den Wortschatz überhaupt für meine Emotionen? Also um bei der ersten Frage zu bleiben.

31:56
P: Wenn mir das bewusst wird, dass es eigentlich ganz schön schwierig ist festzustellen, wie fühle ich mich gerade? Welchen Mix nehme ich da in mir wahr und auch zu verstehen, dass da manchmal die Grundlage dafür auch Achtsamkeit ist. Achtsam mal mich von diesen vielen äußeren Reizen abzuschotten, in welcher Art und Weise auch immer und Taschenlampe nach innen zu richten. Und in mich hineinzuspüren.

32:17
P: Wie fühle ich mich gerade? Ist das erstens einmal nicht nur eine Sache - , wo ich ganz oft schon von den Studierenden gehört habe, noch nie in meinem Leben habe ich mich damit eigentlich so richtig beschäftigt und das finde daher ist schon allein diese Säule so zentral, weil ich denke das ist, was uns selbst allen am nächsten ist, unsere eigenen Emotionen und wie wir damit umgehen im Leben

32:43
P: Die beeinflussen unser ganzes Tun. Auch unser Lehrerinnen Handeln, wenn wir im Beruf bleiben, aber auch jegliches andere private Handeln. Unser Verhalten, aber mittelfristig langfristig auch unsere Gesundheit. Unsere psychische wie körperliche, weil das ja auch alles eine Einheit ist. Das heißt, Emotionen sind für mich Kern und Zelle und Urquell sozusagen in jeglichen Erlebenshandelns und Tuns in diesem Leben. Und daher finde ich gehört diese Thematik noch viel stärker auf den Lehrpersonen bewusst gemacht.

33:18
P: Das ist für mich ein Teil ganz wichtiger Personalisierung auch. Sie haben selbst was davon und sie tragen das wieder an ihre Schülerinnen und Schüler heran. Und so ein Thema kann man natürlich nicht nur theoretisch kognitiv vermitteln. Das muss erfahrbar und erlebbar werden.

33:50

P: Da muss ich selber was spüren, aha interessant. Und parallel dazu, wie gesagt, im Ausgang ist uns das hoffentlich gelungen da eine Balance gut hinzukriegen. Dann im nächsten Schritt nach dieser Selbstwahrnehmung auch in den Austausch zu gehen. Aber andere haben das anders erlebt oder ähnlich erlebt. Oder wie geht's dir? Oder wie geht’s mir? Das erlebe ich immer als sehr bereichernd. Also von anderen zu hören und wahrzunehmen, was deren Erfahrungen sind, wird es auch wieder mit meiner Selbstreflexion was macht.

34:13
P: Und dann natürlich zu dieser erlebbaren Säule, dann schon natürlich auch dieses Fachwissen, was ich vorher schon erwähnt habe, auch kognitive Inhalte dazuzufügen also sozusagen die Menschen in ihrer Gesamtheit anzusprechen auf allen möglichen Kanälen im besten Fall. Ja was ich wahrgenommen habe und wo ich überhaupt davon bin und das muss ich jetzt vielleicht nach Corona Zeiten wirklich erwähnen. Wir haben wirklich einiges auch online gemacht, was extrem praktisch ist, wenn wir (...?) Vorträge voranbringen wollen. Sonst würden wir viele nicht erreichen, die die Möglichkeit nicht hätten Präsenz zu erscheinen.

34:50
P: Wenn wir aber ein Schulstandorten Module abgehalten haben zum Beispiel und das ganze Präsenz abgelaufen ist, hat man halt schon gespiegelt, dass man dann noch einmal anders in diese Erfahrungsqualität auch eingehen kann, wo ich auch eingestehen muss, dass digital mehr möglich ist als ich ursprünglich dachte. Selbst in solchen Wahrnehmungsübungen, das ist auch eine erstaunliche Erkenntnis meinerseits.

B: Wie zufrieden sind sie auf einer Skala von 1 bis 10 mit den Schulungsbausteinen?

35:25
P: Ganz insgesamt? Oh, was sag ich jetzt als Teil des Entwicklerinnenteams. Also ich würde jetzt mal sagen acht bis neun ja tatsächlich, weil ich glaube wir wirklich hier viele Expertinnen und Experten auf hoher Qualität miteinander gearbeitet haben. Ich glaube auch in relativ kurzer Zeit unglaublich viel hervorgekommen ist und wir haben auch jedes Mal nach den Unterhaltungen von diesen Bausteinen eine Reflektion betrieben.

36:45
P: Und warum sage ich jetzt nicht 10? Weil natürlich immer etwas Spielraum da ist, um etwas zu optimieren. An einem Schräubchen da oder dort zu drehen. Und dann ist die Frage: Gibt es überhaupt Perfektion? Ich glaube Perfektion gibt es gar nicht. Wir dürfen stolz sein auf das, was wir geschafft haben im ersten Anlauf, müssen uns aber bewusst sein, dass wir in weiteren Schritten noch adoptieren, verbessern, hinzufügen, weglassen, wollen, können, sollen. Ich glaube das ist notwendig immer einen Veränderungsprozess zu vollziehen.

36:45
P: Dort und da noch zu polieren, um noch ein bisschen mehr zum glänzen zu bringen, aber wie gesagt, ich denke Perfektion ist sowieso eine Illusion. Wir nähern uns dem an, was unsere Vision ist. Es ist uns schon recht gut gelungen für den ersten Schritt. Es können ja noch weitere Fortsetzungsschritte folgen.

B: Wie zufrieden sind sie zum Beispiel mit dem Stärken-café?

P: Was ich da verbessern würde?
B: Oder was sie gut fanden. Sie dürfen beides sagen.

37:23
P: Ich war nicht bei jedem Stärkencafé dabei. (...?) Jedes Stärkencafe war einem anderen Schwerpunkt gewidmet. Was wahrscheinlich auch den Prozess geschuldet war, ich war aktiv dabei - Ich war bei diesen Cafés nur als Zuhörerin/ Zuschauerin dabei, weil es mich einfach interessiert hat.

37:51
P: Ich wollte sehen, was hat sich getan und was kam da zurück. Da habe ich eine andere Meinung gehabt. Weil als Zuschauerin habe ich das Gefühl gehabt, da war der der Zeitrahmen nicht ganz stimmig. Es war – Manche haben sehr weit ausgeholt, es war dann weit über die Zeit hinaus, so wie ich das in Erinnerung habe. Also da müsste man noch ein bisschen an dem Format nach justieren, wie das präsentiert werden kann wieder, wie der Austausch dazu stattfinden kann. Und sowas zum Beispiel könnte ich mir in Präsenz auch schön vorstellen, wenn da auch sehr wertschätzend, wenn man auch mal anderen Kolleginnen begegnen könnte. Da kann auch informeller Austausch stattfinden. Ich stell mir das so vor mit Hochtischen mit dahinter irgendeine Pinnwand oder einfach ein bisschen Fotos –

Videocall ist für eine Minute kurz stehen geblieben

39:00
B: Sie waren gerade kurz eine Sekunde weg. Jetzt geht's wieder. Sie hatten darüber - Also der letzte Punkt war, dass sie darüber erzählt hatten, wie sie sich das bildlich sogar vorstellen.

P: Genau, das habe ich mir vorgestellt wie Messestände, so Informationsstände, wo jeder Standort einen Hochtisch hat mit vielleicht einer Pinnwand oder irgendeinem Laptop, Video oder Ahnung, wie auch immer Fotos zeigt und wo man vielleicht eher informell durchgehen kann, wo vielleicht jede Gruppe zwar ein anteasert, worum es in ihrem Schwerpunkt oder Programm geht.

keine
so
bisschen

39:33
P: Wo dann aber eben wenigstens nicht so einer nach dem anderen alles präsentiert, sondern wo man mehr informell miteinander in den Austausch geht. So könnte ich mir das vorstellen, das ist auch digital möglich, dann würde ich noch ein wenig am Konzept ein

Schräubchen drehen. Obwohl ich glaube es war ganz gut so für den ersten Versuch. Ich glaube schon, dass da einige von profitiert haben. Wie gesagt, aber ich glaube, dass es eine Spur lang war. Ich vermute mal, dass sich dann viele auch vom Rumsitzen, dann irgendwo ausklinken. Das sind Spekulation, aber ich könnte mir vorstellen, dass man die Aufmerksamkeit noch ein bisschen optimieren kann und besser halten kann.

40:14
P: Das ist das eine. (...?) Ich glaube, das war das letzte Stärkencafe, wo wir versucht haben, dann noch einmal einen Schritt weiter zu gehen in der Expertise sozusagen und da haben wir dann versucht auch über Studien ein bisschen mehr noch inhaltlich, auch tiefer hineinzugehen. Es ist immer sehr schwierig, wenn man auch in der Rolle des Vortragenden ist. Da vergeht dann die Zeit immer so schnell, weil man von etwas begeistert ist. Da müsste man dann wieder die Empfängergruppe/ die Zielgruppe fragen, wie sie das wahrgenommen haben.

Ich finde das ganz interessant und lese dann auch eure Berichte, wenn ich die bekomme, weil das natürlich genau diese Weiterentwicklung, von der wir gesprochen haben, für uns sein könnte.

41:07
P: Ein Satz der mir noch einfällt. Gerade das Element des Stärkencafes ist, dass die Kolleginnen und Kollegen unbedingt viel Austausch brauchen. Also dieses Bedürfnis mit anderen in Kommunikation/ Interaktion zu treten. Also dem muss man genug Raum geben. Man hat manchmal als Vortragender oder Leiter so das Gefühl, man muss möglichst viel Input liefern muss. Es wird zum Teil schon auch erwartet. Weil die Teilnehmenden schon darauf warten etwas zu bekommen. Aber wie gesagt in einer guten Balance, dass sie auch selbst zu Wort kommen, dass sie sich selbst gut - wie so Interventionen. Dass sie da auch mal eine Rückmeldung bekommen. Das ist ja auch wieder eine Form der Wertschätzung.

41:45
P: Alleine schon das Präsentieren oder anderen davon zu erzählen macht ja auch wieder bewusst, was schon geleistet wurde. Es zielt auch gleich wieder auf diesen Effekt, den wir ja insgesamt erzielen wollten. Stolz auf das zu sein, was man zusammengebracht hat. Also das hat auch gleich wieder so eine spürbare Wirkung. Das war mir jetzt noch wichtig, dass es genug Raum für Austausch gibt.

42:22
B: Die Bedeutung des Austausches wurde auch von den anderen Experten. Ich hatte ja schon zwei interviewt. Wurde auch immer wieder hervorgerufen. Da besteht absolute Einigkeit.

P: Das belegen ja auch Studien. Diese soziale Eingebundenheit, der soziale Austausch ist sehr wichtig. Man spürt es halt immer wieder aufs Neue.

42:45

B: Dann noch einmal zu ihnen. Wie geht's für Sie jetzt weiter?

Ü: Meinen Sie im Zuge des PERMA.teach Projektes? Oder allgemein? B: Allgemein.

42:56
P: Ja, ich bleibe diesen Weg treu. Vielleicht ganz konkret darf ich Ihnen erzählen, oder Dir erzählen. Bei der PERMA Kultur sind wir immer bei Du.

43:13
P: Ich habe - Es kam der Aufruf von der Eva. Sie hätte gern ein Lied oder einen Song, der PERMA ausdrückt, vermittelt und musikalisch erfahrbar, erlebbar, spürbar macht. Und nachdem ich auch gerne Musik mache, immer wieder gerne mit meinen Schulkindern musiziert hab, habe ich mich irgendwann hingesetzt und einen PERMA Song geschrieben. Und dann nur eine erste, ganz einfache Aufnahme weitergeschickt und die waren dann gleich so euphorisch, dass diese noch so stümperhafte Aufnahme gleich verbreitet wurde. (...?) Aber es ist jetzt so weit gekommen, dass die Eva gebeten hat, sie wünscht sich, dass der Song aufgenommen wird, dass es ein Video gibt. Das soll auch auf die Homepage und das soll dann auch ein Teil der Tools sozusagen sein, die sich auch Lehrerinnen, Lehrer oder Kindergartenpädagoginnen, Kindergartenpädagogen abholen können. Vielleicht auch singen,

vielleicht als Inspiration nehmen, selber was anderes Kreatives dazu zu machen und das freut mich riesig, weil das natürlich auch einmal eine wunderschöne Abwechslung zu meinen sonstigen Hochschulischen Aufgaben ist.

44:19
P: Wie gesagt ein Lied mit Kindern, Jugendlichen. Eva wünscht sich, dass auch Erwachsene dabei sind, damit man diese Punkte der Vielfalt sieht. Das es für alle ein Thema ist (...?)

44:43
P: Ja, das ist konkret der nächste Schritt. Wir haben erst jetzt im Juni angefangen zu denken, das heißt das braucht jetzt noch bis Oktober, bis wir das realisieren können. Jetzt sind ja erstmal Ferien. Wir brauchen ja die Kinder und die Chorleiter und so weiter. Ja, das ist das nächste konkrete Ziel. Und in all meinen anderen Tätigkeiten bin ich auch dran - Wie soll ich sagen? Zu verfeinern. Ich habe zum Beispiel in einem Seminar, in einem Vertiefungsseminar, eine Internetreihe für meine Studierenden vor zwei drei Jahren entworfen, wo sie eben auch, nicht nur zu PERMA.teach, aber auch zu anderen Themenbereichen, die relevant sind, wenn man beginnt Lehrerinnen und Lehrer zu werden, recherchieren soll und so weiter.

45:16
P: Die sind auch sehr praxisnah. Die dienen zur Verknüpfung mit der Praxis. Und da werde ich auf jeden Fall jetzt im Sommer die neuen Instrumente und Tools und Folien und Broschüren, die es gibt, zufügen. Ich habe auch bemerkt, dass die, die eine handy-app zu Stärken-stärken, jetzt nicht nur funktionieren. Also das alles werde ich jetzt im nächsten Schritt auf den neuesten Stand bringen,

sozusagen. Das ist eine ganz konkrete Maßnahme damit auch alles up to date ist. Und ansonsten habe ich auch noch vor in meinem - ich habe eine Gruppe bei mir von Hochschullehrer-Personen. Wir nennen uns (Pückgruppe?) also da geht es um personenbezogene, überfachliche Kompetenzen. Es sind Kollegen und Kolleginnen, die aus unterschiedlichen Bereichen in der Methodik, der Didaktik oder wo auch immer Persönlichkeitsarbeit einbauen.

46:10
P: Auch im Coaching. Und dort werde ich bei der nächsten Besprechung oder bei einer der nächsten Besprechungen - Es sind manchmal aktuelle Themen die vorrangig sind. Im Moment haben wir zum Beispiel eine (...?) zu organisieren, das ist sehr kurzfristig. Aber sobald wieder ein Slot frei ist, möchte ich PERMA.Teach auch in diese Gruppe wieder hineintragen und versuchen zu schauen, dass auch hochschulintern sozusagen noch mehr Vernetzung, Austausch und Absprache zu dem Thema auch noch stattfindet.

46:37
B: Also du bleibst auch weiterhin PERMA.teach treu?

P: Auf jeden Fall. Wenn Eva wieder ausschreibt, oder wieder Gelder kommt, was auch immer nötig ist, bin ich natürlich immer offen und freue mich, wenn ich angesprochen werde.

B: Schön! Das waren alle meine Fragen. Du hast alles gut abgedeckt. Danke!
