# Supplementary material for: PERMA.teach: a study on the effectiveness of a standardized positive education training program in Austria
Source: Front Psychol. 2025 Apr 8;16:1516572. doi: 10.3389/fpsyg.2025.1516572 (PMC12053473; doi:10.3389/fpsyg.2025.1516572)
Supplement: Supplementary file 2 [file Data_Sheet_1.ZIP › Material PERMA.teach/Material LPs/Interviewleitfaden_PERMA.teach.pdf]

Leitfaden Experteninterviews

Begrüßung und Bedanken, Kurze Vorstellung
Darstellung der Thematik, Ablauf des Interviews
Einverständniserklärung zur Video und Audio-Aufnahme und Weiterverarbeitung anonymisiert
Aufnahme starten

	**Einstiegsfragen**
**Tätigkeits-feld des Ex-perten**	**1.** Wie lange sind Sie bereits als Lehrkraft tätig?<br>**2.** Seit wann arbeiten Sie an der aktuellen Schule?<br>**3.** Welche Klassen unterrichten Sie?<br>**4.** Was würden Sie sagen, ist für Sie die Hauptmotivation Lehrkraft zu sein? Bzw. Warum sind Sie Lehrperson geworden?
**Deduktive Kategorien**	**Interviewfragen**
**1.**<br><br>**Motivation zur Teilnahme**<br><br>**Vorwissen zu Positiver Bil-dung**	**5.** Hatten Sie vor den Fortbildungen bereits Vorwissen zur Positiven Psychologie und/oder der Po-sitiven Bildung?<br>a. Wenn ja, welches?<br>b. Was hat Sie von der Positiven Bildung überzeugt?<br><br>**6.** Mit dem Wissen aus der Fortbildung: Stellen Sie sich bitte vor, Sie müssten einem Außenste-henden den Unterschied zwischen Regelunterricht und Positiver Bildung erklären. Wie würden Sie das kurz und knapp formulieren?<br><br>**7.** Wie haben Sie von PERMA.teach erfahren? Warum haben sie an dieser Fortbildung teilgenom-men?<br><br>**8.** Was waren die ersten Gedanken, die Ihnen zu diesem Projekt eingefallen sind?<br><br>**9.** Was hat Sie motiviert, an der Fortbildung teilzunehmen?<br><br>**10.** Was war die Erwartung zu Beginn des Fortbildungskonzepts?<br>a. Gibt es etwas, was Sie besonders angesprochen hat, worauf Sie sich gefreut haben?<br>b. Gibt es etwas, bei dem Sie zu Beginn skeptisch waren?<br><br>**11.** Wie würden Sie Ihre aktuelle Motivation, PERMA.teach in der Schule umzusetzen, einschätzen?<br><br>**12.** Würden Sie diese Fortbildung anderen empfehlen?<br>a. Was ist für Sie das entscheidende Argument, das für die Fortbildung spricht?<br>b. Gibt es Argumente, die aus Ihrer Sicht gegen diese Fortbildung sprechen?
**2.**<br>**Feedback zu Inhalten und**	**13.** Wie bewerten Sie die Schulungsbausteine im Modul 1 und 2?<br>a. Wie fanden Sie die Schulung? Fanden die bei Ihnen in Präsenz oder virtuell statt?

**Qualität der Schulungs-bausteine**	b. Wie ging es Ihnen mit der Vernetzung mit den anderen TN?<br>c. Wie bewerten Sie die fachlichen Impulse?<br>d. Wie bewerten Sie das schriftliche Handout?<br>e. Wie bewerten Sie die weiterführenden Inspirationsquellen (z. B. Apps und Padlets)?<br>f. Bitte geben Sie ein Feedback zu den Rahmenbedingungen (Einladung, Erinnerung, Terminabgleichung etc.).<br>g. Gibt es etwas, was Sie sich bei den beiden Modulen noch gewünscht hätten?<br>h. Oder gibt es etwas, was aus Ihrer Sicht weggelassen werden könnte?<br><br>**14.** Was konnten Sie sich aus dem Stärken-Café mitnehmen? (z. B. PERMA.Highlights)<br><br>**15.** Wie bewerten Sie das Buch „Jedes Kind stärken“/“Jugend stärken“?<br><br>**16.** Wie bewerten Sie die PERMA.Post?<br>a. Haben Sie diese erhalten?<br>b. Was konnten Sie aus der PERMA.Post mitnehmen?<br>c. Konnten Sie das darin enthaltene Material in Ihren Alltag integrieren?<br><br>**17.** Wie hilfreich bewerten Sie das Padlet?<br><br>**18.** Welches Material aus dem gesamten Repertoire war für Sie persönlich besonders hilfreich?<br><br>**19.** Gab es ein Material, aus dem Sie persönlich weniger Nutzen ziehen konnten?<br>a. Wenn ja, welches Material/Übung war es und warum?<br><br>**20.** Was hätten Sie sich noch gewünscht? Material, Input etc.<br><br>**21.** Gibt es noch etwas, was Sie zum Fortbildungskonzept sagen möchten?
**3. Informatio-nen zur Umsetzung im Unterricht**	**22.** In welchen Klassen haben Sie PERMA.teach angewendet?<br><br>**23.** Gibt es Klassen, in denen Sie PERMA.teach häufiger/weniger häufig angewendet haben?<br>a. Wenn ja, woran meinen Sie, lag es?<br><br>**24.** In welchen Unterrichtseinheiten haben Sie PERMA.teach angewandt?<br>a. Als Haltung, z. B. bei der Begrüßung/Einstieg?<br>b. Im Fachunterricht, z. B. Deutsch: Textanalyse nach Stärken?<br>c. Gibt es Rituale, Projekte, die Sie mit Ihrer Klasse durchgeführt haben?<br>d. Wenn nein, gibt es etwas, was Sie (noch) dafür benötigen?<br>e. Wenn ja, was?<br><br>**25.** Welche Maßnahmen aus dem angebotenen Repertoire wurden angewandt?<br>a. Welche Erfahrungen haben Sie mit der Methode in der Klasse gemacht?<br>b. Gibt es bestimmte Übungen, die Ihnen näherliegen?<br>c. Können Sie begründen, warum Ihnen gerade diese Übungen näherliegen?<br>d. Gibt es Übungen, die Sie seltener oder gar nicht verwenden?

	**26.** Welche Maßnahmen wurden über die angebotenen Übungen hinaus angewandt?<br>a. Welche Erfahrungen haben Sie mit der Methode in der Klasse gemacht?<br>b. Gibt es bestimmte Übungen, die Ihnen näherliegen?<br>c. Können Sie begründen, warum Ihnen gerade diese Übungen näherliegen?
**4.**<br>**Feedback zu Veränderungen bei den SuS**	**27.** Welche positiven Veränderungen haben Sie bei Ihren Schülern wahrgenommen?<br>a. P<br>b. E<br>c. R<br>d. M<br>e. A<br><br>**28.** Was ist für Sie die „schönste“ Veränderung, die Sie auf PERMA.teach zurückführen können? (Wo ging Ihnen das Herz auf?)<br>**29.** Woran meinen Sie liegt das?
5.<br>**Veränderung bei der Lehrperson**<br><br>**6.**<br>**Einfluss der Pos. Entwicklung auf die Professionsentwicklung**	**30.** Was ist für Sie die schönste Veränderung, die Sie an sich selbst wahrgenommen haben?<br>a. Im Umgang mit den Schülern?<br>b. Im Umgang mit den Kolleg:innen?<br>c. Im beruflichen Alltag?<br>d. Gibt es darüber hinaus noch Veränderungen (pos. wie neg.)?<br><br>**31.** Gibt es Veränderungen, die Sie den PERMA-Faktoren zuordnen können?<br>a. P<br>b. E<br>c. R<br>d. M<br>e. A<br><br>**32.** Hat PERMA.teach einen Einfluss auf Sie als Lehrkraft?<br>a. Können Sie ein typisches Beispiel aus dem Alltag darstellen, das diese Veränderung verdeutlicht?<br>b. Wie geht es Ihnen mit dieser Veränderung?
	**33.** Woran würde ein Außenstehender erkennen, dass sie die Positive Bildung in Ihrem Unterricht anwenden?<br><br>**34.** Woran würde ein Außenstehender erkennen, dass Sie die Positive Bildung „leben“? (- Außenwirkung/Wechselwirkung)<br><br>**35.** Wen würden Sie gerne mitnehmen „auf dieser positiven Bildungsreise“? Umsetzung von PERMA als Haltung
**7.**<br>**Haltung und Unterstützung durch die Schulleitung**	**36.** Wie erleben Sie die Haltung der Schulleitung gegenüber PERMA.teach?<br>**37.** Wie erleben Sie die Unterstützung durch die Schulleitung?<br>**38.** Wann und wie können Sie sich mit Kolleg:innen über PERMA.teach austauschen?

	**39.** Wie könnte PERMA.teach an Ihrer Schule noch weiterverbreitet werden? Was benötigen Sie dazu?
**8. Verstetigung von PERMA.teach**	**40.** Welche Veränderung können Sie an Ihrer Schule feststellen?<br>a. Gibt es Rituale, Projekte, Leitbild, Visionen etc.?<br>b. Gibt es Rituale, Projekte etc., die Sie gerne in Ihrer Schule etablieren wollen würden?<br>c. Was könnte helfen, diese auf den Weg zu bringen?
**9. Sonstiges**	**41.** Bitte formulieren Sie mir möglichst in wenigen Sätzen Ihren Hauptnutzen von PERMA.teach.<br><br>**42.** Wofür sind Sie dankbar? Nennen Sie drei Gefühle, die Sie mit PERMA.teach in Verbindung bringen.<br><br>**43.** Gibt es etwas, was Sie noch ergänzen möchten?
