# Supplementary material for: PERMA.teach: a study on the effectiveness of a standardized positive education training program in Austria
Source: Front Psychol. 2025 Apr 8;16:1516572. doi: 10.3389/fpsyg.2025.1516572 (PMC12053473; doi:10.3389/fpsyg.2025.1516572)
Supplement: Supplementary file 2 [file Data_Sheet_1.ZIP › Material PERMA.teach/Material LPs/Interviews LPs Teil 2.pdf]

gehen darf, für das bin ich irrsinnig dankbar. ähm wie viele in der arbeit, erleben wir viel, kriegen viel von den kindern zurück, für das bin ich wahnsinnig dankbar. fhm, ja, für die familie bin ich dankbar, die einfach für einen da ist, und wo man den rückhalt kriegt, für meinen freundeskreis bin ich dankbar (LACHT), und ähm für die natur an sich bin ich dankbar, weil ich freue mich über jeden schmetterling, jede raupe, jede * ja jede kleinigkeit oder wenn die knospen wachsen im frühling, dann freue ich mich drüber. ja, also über die ganze natur bin ich dankbar wie wir es haben. ähm wie wir leben dürfen, bin ich dankbar.

I: sehr schön.

B: ja, ich habe ganz viel sachen. (LACHT) genau. für jedes lachen bin ich dankbar, was ich haben darf. genau. und für den glauben, den ich habe, für den bin ich auch dankbar, das ich einfach da auch gefestigt bin. einfach weiß, das ist ein guter fels an den ich mich halten kann. für das bin ich auch dankbar.

I: sehr schön. dann * vielen dank für die schöne zeit.

B: danke.

I: und jetzt wünsche ich ihnen einen schönen sonnigen, also bei uns scheint die sonne, ich hoffe, bei ihnen auch.

B: hier ist es wunerschön. ich werde jetzt mit meinem kleinen nach draußen gehen. (LACHT)

I: das klingt nach einem sehr guten plan. dann eine schöne zeit.

B: ja ebenfalls, danke. ich hoffe ich habe ein wenig helfen können.

I: auf jeden fall, tschüss.

B: ciao.

Anhang E: Interview 4

01.06.2023 14 Uhr

I: muss ich dann noch bestätigen.

B: ich versuche es jetzt nur noch ein bisschen * weil sie brauchen auch kein so vollbild von mir.

I: * das ist auch * im nachhinein wird das nicht verarbeitet. also, es geht wirklich nur um, um den text dann letzten endes.

B: ja, ja, ja. * so, jetzt hoffe ich, dass das so passt.

I: absolut.

B: so, passt.

I: (sollen wir ku*?)

B: hier im büro. (LACHT) und meine, meine pinwand drauf. ja. (LACHT)

I: das kann man alles gar nicht so genau erkennen. (LACHT)

B: ja, gut.

I: okay, dann sage ich ihnen ganz kurz nur was zu mir, damit sie auch wissen, mit wem sie es zu tun haben.

B: mhm. (zustimmend)

I: ähm, ich komme aus deutschland. ich unterrichte in deutschland an einer fachakademie für sozialpädagogik. wir bilden also erzieher und erzieherinnen aus. bin selber auch grundständig erzieherin und habe kindheitspädagogik studiert und mache jetzt eben berufsbegleitend meinen master. und so bin ich auch zu der studie gekommen. wie gerade schon gesagt, ist ja zum einen für dieses perma.teach zu schauen, ähm, wie es in der praxis wirkt. und ich habe noch einen zusätzlichen schwerpunkt, da geht es tatsächlich auf ähm, auf lehrerprofession letzten endes. also, welche auswirkungen perma.teach oder generell die positive bildung, auf die lehrerpersönlichkeit und deren entwicklung haben kann.

B: mhm. (zustimmend)

I: genau, das ist so ein bisschen die beiden richtungen, die ähm, das interview verfolgt.

B: okay. ähm, da habe ich eine kurze frage zu ihrem background oder halt allgemein eigentlich zur ausbildung in deutschland. ist es bei ihnen schon äh, länger standard, dass äh, die zusatzqualifikationen dann berufsbegleitend gemacht werden? * so wie sie jetzt gesagt haben, sie machen den master berufsbegleitend?

I: * nein. also, ähm, sie meinen jetzt, um, um dort als lehrkraft tätig zu sein?

B: ja, zum beispiel.

I: mhm. (zustimmend) also, die, die au* ausbildung zur erzieherin ähm, ist tatsächlich so, dass die meisten ähm, eine ausbildung und ein studium fachspezifisch haben. also jetzt bei mir, ist es eben kindheitspädagogik. oder die musiker, haben dann ein, ein musisches studium. und die wenigsten haben wirklich lehramt studiert. das sind bei uns die, die ähm, deutsch oder englisch unterrichten, die haben ein reguläres lehramtsstudium. und die anderen haben halt dann ein, ein fachspezifisches studium ähm, auf bachelor- oder masterebene.

B: mhm. okay, okay. ja, gut, interessant. (LACHT)

I: (LACHT) ja. also, da gibt es tatsächlich doch die einen oder anderen unterschiede zwischen deutschland und österreich. (LACHT)

B: ja, die (rema?). ja.

I: (LACHT) okay, dann ähm, würde ich zu anfang sie einfach bitten, dass sie mir ein bisschen was über sich als, als lehrkraft erzählen, wer sie so sind, dass ich mal einen eindruck davon bekomme, mit wem ich denn spreche. (LACHT)

B: ja, ja, ja gut. also, ich bin jetzt am ende meiner dienstlaufbahn und ähm, ich bin ausgebildete volksschullehrerin, so heißt das bei uns. ähm, damals auf der pädagogischen hochschule, früher hat es pädak geheißen, zu meiner zeit, habe ich meinen schwerpunkt in vorschulerziehung und ähm, eine zusatzausbildung auch für katholischen religionsunterricht gemacht und sonst ein ganz normales äh, gesamtstudium einfach für äh, volksschul* äh* lehramt. ähm, und dann ähm, habe ich eigentlich durchgehend unterrichtet. ich habe zwar vier kinder bekommen, aber ich bin eigentlich immer wieder relativ schnell eingestiegen. ähm, * eigentlich immer, wenn bei uns die karenzzeiten, äh, sind zwei jahre, und äh, da bin ich meistens knapp davor oder knapp danach, je nachdem, wie sich das mit dem schuljahr ausgegangen ist, entweder ziemlich, ziemlich gleich voll oder als es dann mehrere kinder waren, halt noch ein, zwei jahre mit reduzierten stunden äh, lehrverpflichtung und ansonsten dann gleich wieder voll. und seit ähm, acht jahren bin ich jetzt hier in klösterle die schulleiterin. also, ich war vorher im nachbardorf. wir leben in einer sehr ländlichen region * äh, mit kleinstschulen. das heißt, die schülerzahlen bewegen sich irgendwo zwischen 20 und 35, 40 schülern. äh, wir haben im moment äh, ähm, 32 kinder an der schule. äh, das bedeutet dann, ha* wir haben dann immer zwei klassen mit jeweils jahrgangsmischungen, erste, zweite und vorschulstufe integriert und dritte, vierte schulstufe. äh, wie gesagt, die gegend ist ländlich und touristisch geprägt. ähm, * wir sind in klösterle eine umweltzeichen-schule. also, das ist ein güte* gütesiegel, dass wir alle vier jahre wieder äh, äh, erneuern müssen, wo wir uns einer überprüfung unterziehen müssen. und äh, da werden ei* eigentlich sämtliche lebens- und äh, pädagogischen bereiche ähm, abgeprüft oder, oder halt nach bestimmten standards, ähm, wird man *. äh, * ähm, kontrolliert ist fa* ist falsch gesagt. aber halt, man muss das einfach erfüllen, diese bestimmten kriterien. ja, und ansonsten an unserer schule, ähm,

wir sind vier kolleginnen zurzeit, aber nur zwei mit voller lehrverpflichtung und zwei mit halber lehrverpflichtung. das heißt also, insgesamt würde unsere schülerzahl drei stellen ergeben. heuer haben wir auch zusätzlich eine äh, pädagogische assistentin, die ähm, uns äh, in einem bisschen schwierigeren klassensetting unterstützt. ja, und ansonsten steht es uns zur verfügung, eine sprachheillehrerin, die an die schule kommt und eine äh, pädagogische beraterin, die in regelmäßigen abständen sich mit uns austauscht. und wo wir immer unsere ähm, * problematischeren äh, fälle einfach besprechen können und sie uns tipps gibt beziehungsweise sie vernetzt. ja. * so, das ist der hintergrund, jetzt einmal im allgemeinen.

I: mhm. okay, schon mal ganz spannend, vielen dank. ähm, wie sind sie denn auf die, auf das fortbildungskonzept perma.teach aufmerksam geworden?

B: * auch durch das umweltzeichen. weil die umweltzeichen *. ähm, also gibt es einen punkt soziales, äh, integration, ähm, * äh, vernetzung. und ähm, ja, da hat das alles gut zusammengepasst. wir äh, gestartet haben wir eigentlich im bildungsnetzwerk inklusion. und aus dem, da waren wir ähm, angeleitet von der *. Oder angestupst, wurde das ganze von der äh, schulqualitätsmanagerin damaligen, j. s. äh, und die hat so ein bildungsnetzwerk äh, begonnen. und da waren wir halt einige schulen, aller ermöglichen größen aus dem, aus unserem bezirk, politischen oder, oder schulbezirk. und ähm, und da hatten wir dann zum index inklusion eineinhalb, zwei jahre gearbeitet, mit input äh, aus südtirol. weil südtirol ja da eine vorbildliche region ist. und äh, und aus dem heraus ist über die pädagogische fe* hochschule feldkirch, mit der uli lichtinger, dann ähm, der kontakt äh, erwachsen. und dann hat sich das eben in richtung perma.teach. durch das flori* flourishing ist es äh, dann ähm, weitergegangen, äh, auf die äh, ingrid teufel und eva, hm, jambor.

I: jambor.

B: ja. und dann, und dann waren wir da in diesem perma.teach äh, ähm, kreis drinnen. ja, und das hat, das passt sehr gut zu unserem konzept. also äh, zum beispiel ähm, selbstreflexion, teilhabe und diese ganzen dinge, äh, umgang miteinander, das ist im umweltzeichen einfach auch drinnen. das ist einfach auch ein bereich, der gute umgang miteinander. und ich habe das für sinnvoll gehalten, dass wir uns da einklinken.

I: mhm, das heißt, sie haben auch schon ein bisschen vorwissen zur positiven bildung gehabt, bevor sie zum, bevor sie an dem fortbildungskonzept teilgenommen haben?

B: ja, ein klein wenig. wir hatten *. unter uns schulleitern hatten wir mal einen ganzen ähm, einen ganzen tag, einen ganzen pädagogischen tag, wo wir auch vom ähm, strolz bertram, bertram stolz, zur positiven psychologie, äh, einen ganzen tag input hatten. ja, das war jetzt zwar unabhängig mit dem perma.teach, aber da wurde ich schon, äh, in diese richtung ein bisschen vorsensibilisiert.

I: mhm. was hat sie da am meisten angesprochen, so aus der positiven psychologie und bildung?

B: * mh, * dass man (LACHT) in der sch* ich denke, in der schule, ist man ab und zu gefährdet, dass man so in einen jammer-kreislauf nach unten, ähm, versinkt und gar nicht mehr wahrnimmt, was eigentlich alles passiert und was man eigentlich alles macht und bewegt. und äh, und dieses, jeden tag ein bisschen reflektieren und sich jeden tag einfach ein kleines neues ziel setzen, ähm, so habe ich das halt empfunden, äh, das ha* * habe ich gefunden, dass das guttut und dass man da ein bisschen dagegen ankämpfen kann, dass man nicht immer nur sich gegenseitig vorjammert: „und der hat, und der war lästig und das ist schon wieder vorgefallen“ und so weiter.

I: mhm.

B: vor allem auch, wir sind einfach auch stark konfrontiert damit, dass * die eltern so unzufrieden sind. mit den kindern äh, wären, haben wir immer das gefühl, dass die arbeit eigentlich relativ ähm, gut funktioniert. und dass man, dass man das * dass man ein angenehmes lernklima schafft und einen ort wo, wo, wo sich alle wohlfühlen, alle daran beteiligten. äh, dass das eigentlich nicht immer gleich gut gelingt, aber im großen und ganzen schon. und äh, und, und dann kommen oft so sachen von außen, die irgendwie transportiert und aufgebauscht werden. und da finde ich eben, dass man dazu neigt, dass man dann das positive einfach nicht mehr sieht. und die viele arbeit, die einem, äh, aufgehalst wird, das ist ja dann noch, noch mal, das kommt noch dazu, oder, von, von den institution* von der institution einfach.

I: mhm. haben bei ihnen alle kolleginnen ähm, mitgenommen äh, mitgemacht an dem fortbildungskonzept?

B: also, ursprünglich haben alle gemacht gesta* ähm, mitgemacht gest* weil wir uns das einfach als gemeinsames ziel für das umwelt zei* für die umweltzeichen-prüfung ge* äh, gesetzt haben. und dann äh, das war letztes jahr im frühjahr, da waren alle dabei bis zur nachmittagsbetreuerin. und dann hatten wir aber einen stärkeren personalwechsel. da ist die klassenführende lehrerin weggefallen, da ist äh *. die eine kollegin konnte sich sowieso nicht daran beteiligen bei der erst-fortbildung, weil sie, weil sie, ähm, dort an einer anderen schule unterrichtet hat. und wenn der grundinput weg ist, dann wird es schon ein bisschen schwieriger. sie hat sich zwar immer äh, redlich bemüht, das auch mitzutragen, aber so, so der gesamtimpuls, das musste schon von mir ausgehen. dann hatten wir eben den klassenlehrerinnen-wechsel. die andere kollegin war zwar dabei, ist aber nur an einigen wenigen tagen an der schule und unterrichtet vor allem mathematik und sport. äh, und ja, im sport finde ich, geht es ganz gut mit dem perma.teach. aber ja, wenn man nur, * wenn man nicht so, so, so ganz jeden tag damit konfrontiert ist, dann ist es auch ein bisschen schwieriger zum umsetzen, finde ich. ja, und dann äh, dann die nachmittagsbetreuung hat auch gewechselt. und die neue klassenführende lehrerin, die kämpft noch so mit dem system, dass sie *. sie hat es zwar probiert, aber sie hat gesagt: „bah, also mit diesen vielen schulstufen ich, äh, mir ist das einfach alles zu viel“, oder? wobei ich denke, dass das ganze eine haltungsgeschichte auch ist. und äh, und wenn die

haltung in der schule äh, von den kolleginnen passt, dann äh, passiert wahrscheinlich einiges, ohne dass man es wirklich explizit als perma* äh, ähm, aktion wahrnimmt.

I: mhm. * wie würden sie sagen, wird das perma jetzt bei ihnen in der schule umgesetzt?

B: *4Ü* ähm, grundsätzlich einmal durch die, durch die einstellung der lehrpersonen, dass man das wahr* das positive versucht, äh, wahrzunehmen und, und, und hochzuhalten. äh, dann ähm, natürlich arbeite ich mit den größeren auch immer wieder in perma.heft. und dann aber durch die übungen im perma.heft, ähm, fallen einem dann auch, wenn man so ein bisschen aufmerksam im internet auch unterwegs ist, (RÄUSPERT) gibt's ja genügend foren. jetzt gerade auf instagram, gibt es auch sehr viele solche, so, so kolleg* ei* ein, das insta-kollegium, wo auch ähm, sachen dabei sind, die einfach auch in diese richtung gehen. wo man selbstreflexion macht, wo man äh, wertschätzung, warme dusche und wertschätzung vermittelt und so. und, und da habe ich jetzt schon *. immer wieder fallen mir so dinge zu, die ich mir dann ausdrücke, drucke. und ich habe mir so eine perma.schachtel gemacht, wo ich das einfach alles sammle und äh, und dann immer wieder einfach in den unterricht einbaue.

I: mhm. * wollen sie mir vielleicht ein oder zwei beispiele ein bisschen näher erzählen, dass ich mir gut vorstellen kann, wie sie es jetzt einfach umsetzen?

B: mhm, mhm. (zustimmend) also im herbst, als, als, als das auch für mich noch relativ neu war *. wir hatten ja immer die stärken.cafés. und an den fortbildungen haben übrigens die meisten kolleginnen, wenn, wenn sie die möglichkeit hatten, teilgenommen. also, da, das da, das tragen sie schon mit, aber sie tun sich mit der umsetzung äh, nicht immer so leicht, im alltag. ja, äh, und dann habe ich eben äh, gestartet, zum beispiel, also mit dem perma.heft. aber zum beispiel ist dann in dem beiheft eine übung drinnen, die heißt 4-ecken-spiel. und da mussten die kinder * eine frage, was weiß ich: „wo verbringst du am liebsten deine freizeit? am spielplatz, zu hause in meinem zimmer, im schwimmbad, im wald?“ Und dann mussten sie sich zuordnen. und ähm, das war ganz spannend, weil sie auf einmal gesehen haben: „ah, du stehst immer gleich wie ich.“ darf ich so sprechen? * verstehen sie, wenn ich, #wenn ich

I: ja.#

B: mundart rede? äh, * und beim nächsten mal waren vielleicht nur zwei kinder in einer ecke und acht in einer anderen ecke. und das war für die kinder, aber auch für mich total spannend. also, ich habe vor allem den prozess beobachtet, wie sie das äh, sich gegenseitig: „aha, ma, da sind wir immer gleich.“ oder: „bah, diesmal st* äh, äh, äh, stehst du ganzwoanders.“ und so, also das war total spannend. dann äh, haben wir zum beispiel so ein danke-plakat gestaltet, wo wir jetzt nach wie vorher äh, nicht so regelmäßig, aber immer wieder, wenn es mir halt hineinpasst *. äh, gestartet haben wir mit so übungen: „ich bin dankbar für ...“ aber ich möchte es * oder ich habe es auch erweitert äh, dass man auch an jemanden anderen einmal ein danke weitergibt. und, also das finde

ich jetzt auch so eine übung, die, die sich äh, ganz gut ähm, durchs, durchs schuljahr ziehen lässt und die einem einfach auch ein bisschen schuld *. das habe ich auch von der positiven psychologie ein bisschen auch mitgenommen, dass man einfach ähm, bewusst wahrnimmt, was einem gutes geschieht. * und heute habe ich, äh, am donnerstag habe ich immer stationen-tag. und da hatten wir stationen übers ganze schulhaus verteilt und auch ins freie. und wenn die kinder äh, * mehrere stationen erledigt hatten, durften sie sich da mal dazwischen eine kleine belohnung nehmen. und als schluss-belohnung, habe ich so karten ausgedruckt. die kann ich jetzt gerade schnell holen, weil ich sie griffbereit habe.

I: mhm. (zustimmend)

B: (HOLT KARTEN) *13Ü* so *3Ü* ah ja. *3Ü* sind eigentlich vom zaubereinmaleins und sind eigentlich schon * uralt ehrlich gesagt. aber ich bin vor kurzem beim surfen halt drauf gestoßen. und da waren so, so mini-auszeichnungen, nennt sich das. ich weiß nicht, wahrscheinlich halte ich es jetzt verkehrt herum. aber sie können es eh nicht lesen, oder, weil es jetzt spiegelverkehrt ist. zum beispiel mini-auszeichnung: „du bist sehr rücksichtsvoll und hilfsbereit.“ oder ähm, ei* eines war: „ähm, selten jemanden gesehen, der sich so viel mühe gibt wie du.“ oder: * „wir sind froh, dich in unsere klasse zu haben.“ oder: „mini-auszeichnung für die weltschönste schrift.“ oder: „ich gratuliere dir, du hast dein ziel schon fast erreicht.“ und wir hatten gestern auch zufälligerweise *. oder: „weiter so, du bist auf einem guten weg und ich bin stolz auf dich.“ und gestern hatten wir eben ref* schüler, eltern, äh, lehrer, reflexionsgespräche. und da haben wir natürlich auch feedback gegeben, mit den kindern dabei. und * heute sind zwei kinder zu mir gekommen und haben gesagt: „wah, jetzt habe ich mir diese und diese karte ausgesucht. ich glaube, das passt super für mich, oder?“ mit dem background, was ich gestern zu ihnen gesagt habe und was sie selber *. den elternsprechtag gestalten wir nämlich auch so, mit so einem, mit * diesmal haben wir die reflexionsscheibe, diese mandalascheibe genommen. ich weiß nicht, wahrscheinlich kennen sie die eh, oder?

I: mhm. (zustimmend)

B: okay. * buh, wenn ich ihnen so viel zeige, dann sind wir nicht durch, in einer stunde. (LACHT) ähm, * ja, da liegt das eine, das andere nicht. ja. *10Ü* haben wir so eine, so eine mandalascheibe gehabt. da geht es hier um äh, persönliche wahrnehmung. also, das kind hat sich selber ähm, äh, zugeordnet, ohne dass wir irgendwas dazu gesagt haben, einfach mal still. und da, auf der einen seite ging es um die ganzen äh, schulischen fächer und auf der anderen seite ging es um so ähm, und so selbstwahrnehmung wie ähm, mein fleiß, meine friedfertigkeit, äh, meine verlässlichkeit und so. auch ein, ein, ein ganzes, äh, äh, ein ganzer bunter bogen. und äh, und da haben sie sich sehr streng auch beurteilt. und wir sind gestern mit dieser scheibe *. also, die kinder haben es vor ein paar tagen gemacht. und äh, ich habe dann das gespräch so gestartet, indem ich, äh, indem meine kolleginnen und wir, äh, das noch mal angeschaut haben, und z* zu dem kind und zur mama gesagt

haben: „ah, da beurst* beurteilst du dich aber streng oder hier hätten wir das* dir zugestimmt. oder wieso glaubst du, dass du hier und hier äh, in diesem bereich bist?“ und das nehmen wir so immer als aufhänger. wir haben noch ein zweites. das ist so ein, auch so was, so ein ähnlicher bogen, * wo sie einfach äh, sich ähm, was überlegen, was sie sich vornehmen, was sie der lehrperson sagen möchten, was sie, was sie gern in der schule machen, welche tugenden und so weiter. also, es geht *. das ist auch was altes. das kommt vom tiroler bildungsserver zum beispiel. ähm, ja, ich habe halt im laufe der jahre schon sehr viel so material zusammengesammelt. und deswegen, wenn es es sich ergibt und ich daran denke, dann bauen wir eben solche sachen ein. und heute, das war echt total schön, dass sie erstens ähm, * aus diesem strauß von, von positiven ähm, äh, lobkärtchen, sich selber was, was aussuchen können, konnten. manchmal mache ich auch, dass ich ihnen das zu-ordne. aber ich habe gemerkt, dass sie auch wirklich, äh, gut sind in dem, dass sie sich selber, ähm, ein bisschen einschätzen und, und sich selber, was passendes suchen. und sie machen das auch sehr gerne. ja.

I: mhm. * sehr schön. aber würden sie sagen, dass sich jetzt durch die fortbildung, das repertoire bei ihnen noch mal erweitert hat oder verändert hat?

B: ähm, ja, schon. ich bin aufmerksamer geworden. ich habe schon vorher auch solche dinge gehabt und gemacht, aber ich bin halt noch ein bisschen aufmerksamer geworden und mache das, setze das noch mehr ein, ja.

I: mhm. * und wenn sie so auf ihre kinder schauen, wenn * die verändern sich ja auch jede,

B: mhm. (zustimmend)

I: jedes jahr haben die kinder auch so eigene themen. aber würden sie sagen, so rückblickend, ähm, gibt es auch eine veränderung bei den kindern, mit der jetzigen herangehensweise, mit mehr auf stärken schauen, * zum beispiel?

B: ja. (LACHT) wenn ich jetzt ja sage, dann, (LACHT) dann ist morgen sicher gleich wieder ganz was schlimmes. aber, (LACHT) bei uns ist es jetzt so: ich habe die dritte, vierte. also, ich steige nicht mit auf, sondern ich habe jeden, jedes jahr die halbe anzahl der schüler zirka neu und die halbe anzahl bleibt, oder? das heißt, die, mit denen ich letztes jahr gearbeitet habe, die sind zum teil schon weg und einen teil habe ich neu dazu bekommen. ähm, mit den eigenen stärken wahrnehmen, das ist so, so eine sache. aber der umgang miteinander, habe ich schon das gefühl, dass *. also, an den eigenen stärken wahrnehmen, möcht* möchte ich eigentlich noch ein bisschen mehr arbeiten. der umgang miteinander und das positive ein bisschen sehen, das allgemein positive, das finde ich, ist schon besser geworden. denn vor kurzem haben sie, habe ich am morgen mit einer kollegin, die nicht jeden tag da ist, kurz noch gesprochen und wir waren nicht ganz pünktlich im klassenzimmer. die kinder haben schon alles herausgerichtet und dann war es mucksmäuschenstill. und ich habe so in die klasse hineingeschaut ums eck und habe gesagt: „oh, sind die kinder überhaupt da, oder?“

und dann haben wir aber noch weiter gesprochen und sie sind die ganze zeit still und ruhig geblieben. und dann bin ich hereingekommen und habe gesagt: „oh, ich glaube, ihr wollt, ihr wollt die, die, die weltbravste klasse werden.“ und dann haben sie gesagt: „ja, das wollen wir (LACHT) unbedingt.“ und äh, also jetzt nicht nur für mich, sondern auch miteinander. und dann haben wir, haben wir nachgefragt, ähm, äh, warum oder, oder wie das bei ihnen jetzt so funktioniert hat? und dann haben sie gesagt, ja, äh, als die kleinen, also dritte schulstufe, heuer dazugestoßen sind, äh, haben sie gemerkt, äh, die großen geben einfach den ton an. und die großen haben gesagt: „ja, wir mussten letztes jahr auch ähm, äh, das lernen, mit den mh, großen miteinander gut umzugehen. und, und so haben wir das jetzt heuer auch weitergegeben.“ ich weiß nicht, ob das jetzt gerade eine gute antwort ist auf ihre frage. aber das ist mir jetzt halt gerade in, in letzter zeit so eindrücklich passiert. also ich finde schon, dass sie, dass ein bisschen was äh, vorangegangen ist. und etwas, was nicht selbstverständlich ist. * weil sie von sich aus ähm, einfach freundlich sein wollten, brav sein wollten, etwas gut machen wollten.

I: * mhm.

B: und nicht nur, nicht leistung, jetzt in dem sinne, nichts, was mit noten äh, äh, belohnt wird oder beurteilt wird.

I: mhm. haben sie sonst irgendwie veränderungen bei den, bei den kindern wahrgenommen?

B: 4Ü also, ich lege auch sehr viel wert auf teamarbeit. das wird natürlich, finde ich, durch perma.teach auch ein bisschen äh, äh, ähm, positiv be* beeinflusst. und da finde ich auch, dass, dass sie schon auf einem guten weg sind. ähm, sie arbeiten nicht mit jedem gleich, äh, gerne zusammen, aber sie arbeiten sehr gerne im team. und das finde ich auch etwas, das eigentlich durch perma.teach schon auch noch einmal, noch einmal forciert worden ist, dass sie auch gut miteinander umgehen und sich gegenseitig bereichern.

I: mhm.

B: also sich* sie von ihren gegenseitigen stärken auch profitieren, * glaube ich, haben sie ein bisschen besser gecheckt, als vorher.

I: mhm. das heißt, durch ähm, durch das stärkten bearbeiten und gemeinsam erkennen, ähm, würden sie sagen, hat sich die, die bereitschaft, im team zusammenzuarbeiten, noch mal gesteigert?

B: ja. ja. ja. ich glaube, sie haben den mehrwert erkannt.

I: mhm. * sehr schön. was ja für später unglaublich wichtig ist, weil man mit* immer #irgendwann

B: ja.#

I: mit anderen zusammenarbeiten

B: ja.

I: und nicht immer nur mit der besten freundin, mit dem besten freund.

B: ja. #ja.

I: sondern# immer auch mal mit, #mit

B: ja.#

I: menschen.

B: manchmal, manchmal teile ich auch zu. und dann sage ich: „okay, das ist jetzt nicht mein lieblingspartner, aber das muss jetzt einfach funktionieren.“ und dann sind sie zwar nicht überglücklich, aber sie wissen, dass sie das akzeptieren müssen. und ein anderes mal dürfen sie sich das ja wieder frei, also frei wählen. und das klappt ganz gut. und äh, und äh, wir haben in österreich ja äh, bald einmal einen neuen lehrplan. jetzt muss ich gerade schauen, hoffentlich ist *. doch, der akku von meinem handy passt noch. äh, ähm, im herbst einen neuen lehrplan. und da ist ja in der präambel, im, im, im grund, äh, äh, äh, sta* also in dem, was allgemeingültig ist, sind ja diese vier äh, kernkompetenzen oder, oder die neuen skills für das 21. jahrhundert drin. und da ist ja teamarbeit explizit erwähnt, dass das äh, äh, verstärkt, ähm, äh, geübt oder, oder dass man dafür sensibilisiert werden soll. ja, und das versuchen wir einfach, äh, wirklich gut umzusetzen.

I: ja.

B: wir haben uns das auch für unseren schulentwicklungsplan so gewählt.

I: mhm. * sie haben ja vorher auch gesagt, dass es auch ganz wichtig ist, dass ähm, das kollegium letzten endes das auch mitträgt und dass es ähm, dass es kolleginnen gibt, denen es mh, mh, schwerer fällt, jetzt im alltag.

B: * mhm. mhm. (zustimmend) äh, es fällt ihnen schwerer, im alltag, so ganz gezielte perma-übungen einzubauen.

I: mhm.

B: weil ich natürlich, ich hab deutsch, ich habe religion, ich habe musik. äh, das sind lauter, lauter fächer, wo, wo ich mir nicht so schwer tue, immer wieder d* da was herauszuziehen und kurz einzubauen, oder? äh, die eine kollegin mit mathematik, die hat es sehr gut drauf, positiv zu verstärken. * aber sie hat immer das gefühl, sie macht zu wenig perma. weil sie nicht jetzt das heft herauszieht und eine übung bearbeitet. im sport, denke ich, äh, würde es auch sehr leicht gelingen, äh, da immer wieder äh, was einzubauen. aber ja, wenn einem oft die zeit fehlt, da was, äh, äh, ähm, sich einzulesen, äh, ähm, dann, dann, ja, ihr* ich habe halt bei mir festgestellt, je mehr ich am anfang investiert habe, umso mehr fallen mir die sachen dann zu ein. jetzt muss ich gerade einen kurzen moment äh *

I: natürlich.

B: (UNTERBRECHUNG SPRICHT MIT ANDERER PERSON) herein? *19Ü*. so, entschuldigung.

I: kein problem. (LACHT) * was glauben sie denn, könnten denn die kolleginnen jetzt, oder was brauchen denn die kolleginnen, um da mehr sicherheit zu gewinnen oder perma für sich, noch mal anders einbringen zu können?

B: es ist so: die meisten kolleginnen ähm, fühlen sich sehr, sehr stark belastet bis überlastet

I: mhm.

B: vom system. und weil wir mit der niederorganisa* organisation natürlich schon auch einen großen anteil mehr vorbereitungsarbeit haben. weil wir bereiten ja immer mehrere schulstufen vor, oder? ähm, * nicht nur zwei. die eine kollegin hat drei, mit den vorschülern drinnen. und wir haben noch zusätzlich jetzt drei nicht-deutschsprachige kinder bekommen, die quer eingestiegen sind, wo a* die alle auf einem unterschiedlichen niveau sind. das muss immer berücksichtigt werden. also, die haben komplett anderen lehrstoff. ähm, * und das ist natürlich schon sehr zehrend. und wie ich ihnen schon gesagt habe, am anfang muss man investieren. und im moment habe ich das gefühl, dass sie einfach * nicht mehr können und sich* und, und wenn sie nur schon hören, sie sollen irgendetwas machen, dann, dann ähm, ist so ein berg von ihnen, vor ihnen und sie kommen gar nicht so weit, dass sie den mehrwert erkennen. deswegen ist es bei mir mo* momentan so, dass ich auf diese karte setze, dass ich durch das beispiel, * dass äh, die kollegen geben, die es halt machen, beziehungsweise wenn sie merken, äh, das wirkt sich gut auf die klasse oder auf das einzelne kind aus, dass sie dadurch motiviert werden, auch solche dinge einzusetzen. also, mit fortbildungen und zusätzlich, du sollst das oder das lernen, geht gar nichts. eine kollegin habe ich, die ist so wie ich auf instagram unterwegs. ich weiß gar nicht, ob das, äh, ob das überhaupt, (LACHT) ob das peinlich ist, wenn ich, wenn ich das sage. (LACHT) weil da ist natürlich auch viel mist, oder? aber, aber es ist, ich finde, es sind auch impulse drauf. und ähm, und ich schicke ihr immer wieder so ideen für, für so kurze soziale spiele für den sport oder so. und, und, und da gebe ich halt immer da so kleine stupser. * aber ich weiß, dass, wenn ich gerade jetzt am ende vom schuljahr, wenn ich sagen würde, jetzt machen wir noch das oder das, dann, dann, das wäre kontraproduktiv. da wollen sie gar nichts davon wissen. * aber zum beispiel war es überhaupt kein thema, dass wir, dass wir diese sachen gemeinsam machen, oder? * das erkennen sie schon und da kennen sie auch den mehrwert.

I: mhm. * genau das ist ja der hintergedanke von perma.teach, letzten endes. also, dass es nicht um die großen sachen geht, sondern dass man für sich ja auch den, die positiven effekte sieht und dann halt lieber auf ein, zwei kleine sachen setzt, als dass man noch mehr druck hat.

B: mhm. (zustimmend)

I: weil das ist ja so eine kernerkenntnis letzten endes, druck und dann was machen müssen und dann aber motiviert sein, das zu machen, das #schließt einander

B: ja, ja.#

I: einfach aus.

B: ja. ja. und ich denke mir, ähm, äh, ja, wenn, wenn ich, wenn ich jetzt da *. ja, druck erzeugt einfach gegendruck, oder? und, und, und wenn ich jetzt da ähm, * einfach so äh, äh, sagen würde, ihr müsst das und das, also es langt einfach schon das, was wir von der bildungsdirektion aus machen müssen, * oder? und wenn es nur kleine dinge sind wie diese, wir haben jetzt verpflichtend so eine äh, schülerbefragung. dass jeder lehrperson einfach im schuljahr, eine schülerbefragung machen muss, wo die kla* wo man die klasse befragt, egal, zu irgendeinem thema halt, oder? und das auch, können wir ja mit dem smartbot total leicht machen, ist überhaupt kein problem. aber rein das schon, dass man das machen muss, obwohl es für mich jetzt überhaupt (LACHT) kein ding ist, aber rein, dass man das schon machen muss, das ist schon wieder so was, dass, dass viele lehrerinnen dann einfach wieder: „ah, jetzt kriegen wir schon wieder was vorgeschrieben, was wir machen müssen, oder?“

I: * ja. wenn es zu viel ist, dann reichen kleinigkeiten, dass es #einfach

B: ja, ja.#

I: noch, noch mehr #ist. mhm.

B: ja. ja. ja.#

I: was würden sie sagen, wenn wir jetzt mal so auf die, auf die lehrer und auf die profession letzten endes schauen? und wir entwickeln uns ja stetig weiter. also ich meine, das lässt sich ja gar nicht vermeiden und ist ja auch ein ganz positiver prozess. welchen mehrwert könnte denn die positive bildung auch auf die lehrkräfte haben?

B: * na ja, schon letztendlich, dass wir auch im, im kollegium einfach positiver sind. ähm, ich denke, was bei uns relativ gut funktioniert, ist, dass wir uns äh, im großen und ganzen, auch nicht immer zu 100 prozent, aber doch, dass wir uns bewusst sind, dass wir ein gutes team sind, wo wir auf * dass wir uns aufeinander verlassen können, dass wir uns unterstützen und äh, dass wir auch versuchen, jeder seine, seine stärken gut einzubringen. dass wird die team auch gut äh, nutzen,

I: mhm.

B: dass wir uns da gegenseitig leben lassen. der eine übernimmt das was er gut kann, der andere übernimmt das. und dass man sich so gegenseitig einfach stärkt.

B: * mhm.

I: und dass man eben nicht in dieses äh, dieses so nach unten gezogen werden äh, äh, hineinfällt, wobei es wirklich äh, phasenweise schwerarbeit ist.

I: mhm. aber würden sie sagen, ähm, die positive bildung kann dann zumindestens ein kleiner gegenpol darstellen?

B: ja schon, natürlich. ja. weil man einfach das positive bewusster wahrnimmt.

I: * mhm.

B: ich habe jetzt zum beispiel ein kleines ritual. das hat eigentlich nicht unbedingt mit der, mit perma.teach was zu tun. aber, aber äh, ja, bei der * als wir diese, diesen tag mit dem bertram strolz hatten, mit der positiven psychologie, ist mir das gleich eingefallen, dass ich das immer mache. und zwar gibt es diese yogitees. ich weiß nicht, ob sie die #kennen.

I: mhm. (zustimmend)#

B: die haben ja so ein kleines sprüchlein immer dran. und ich liebe es, wenn ich in früh, mir einen tee aufbrühe und wenn ich da so ein sprüchlein habe. ähm, * und entweder denke: „ach ja, das passt eh schon“. oder ich habe es als motivation für den, als motto für den, für den tag. also, es ist so ein bisschen banal, gell? aber (LACHT) das sind so, so kleinigkeiten, die mir persönlich halt einfach äh, guttun.

I: mhm. und da sind wir ja wieder ganz, ganz, ganz klar beim perma letzten endes. weil man ja für sich selber auch herausfinden muss, was tut mir gut? und wenn es in der früh #einfach

B: mhm.#

I: so eine kleine botschaft ist, die, auf die ich mich auch freuen kann

B: mhm.

I: und die mich entweder bestärkt oder noch mal motiviert, dann ist es ja, dann macht das was positives mit mir. und wenn es mir gut geht, strahle ich das ja auch noch mal ganz anders aus. und das wirkt ja dann auch wieder auf die kinder oder #auf

B: ja.#

I: die kolleginnen.

B: ja, ja. wir haben jetzt zum beispiel eine kollegin, der geht es zurzeit nicht so gut, weil sie einen vorfall hatte im unterricht. und äh, ich merke, dass einfach sie * ähm, * ja, da braucht es gar nicht viel, dass sie einfach ihre motivation verliert. sie hat mir jetzt zum beispiel ähm, heute etwas mitgebracht, auf das ich schon ganz lange gewartet habe. und es ist eine kleinigkeit und es ist ihr zufällig untergekommen. sie hat an mich gedacht und hat mir das gebracht. es ist aber etwas für die schüler. und äh, und äh, dann ähm, habe ich ge* gesagt: „was kriegst du?“ also, wie viel hat es gekostet? und dann hat sie gesagt. „drei euro. normalerweise hätte ich es dir äh, geschenkt, aber ähm, momentan reicht es mir so.“ äh, ja. und dann habe ich gesagt: „kein problem, ich hätte es ja sowieso

dir äh, äh, zahlen wollen.“ und ich finde, man hat auch das recht, wenn man, wenn man das gefühl hat, dass grenzen überschritten worden sind und dass man immer, dass man ausgenutzt wird und das, was man positives weiter* äh, gibt, äh, nicht wertgeschätzt wird. und die, und sie ist momentan einfach au* auf einem, in einer phase, wo, wo, wo sie jetzt das einfach nicht mehr tun kann. und äh, ich verstehe das und versuche halt wieder, sie äh, äh, auf schiene, also halt, sie irgendwie wieder so äh, zu, zu unterstützen und, und ihr wertschätzung entgegenzubringen, dass ich von meiner seite aus eben, äh, beitrage, was, was ich beitragen kann, als, als schulleiterin und auch als klassenlehrerin. aber es sind nicht immer dinge, die ich beeinflussen kann.

I: mhm, das stimmt. ja. * mhm. ähm, sie haben auch gesagt, dass sie bei den stärken.cafés waren. wie, wie ging es ihnen mit den stärken.cafés?

B: * ja, das ist schwierig. äh, also es war vom, vom zeitlichen her meistens ähm, recht lang, im verhältnis jetzt für uns. aber * äh, die ideen *. es ist halt dann schon immer etwas dabei, was, das man gut gebrauchen kann, oder? also, der austausch ist schon wertvoll. es ist, ähm, wie soll ich sagen, eine kollegin habe ich dabei, die sagt: „mah, bitte, lass mich mit dem in ruhe, das dauert mir viel zu lang, und es ist viel zu viel äh, getüdel dabei, oder?“ äh, aber * letztendlich, irgendwas ist immer dabei, was man, was man gut nutzen kann, was einen selber anspricht. ähm, ja, und wer, wer soll das *? es kann ja niemand vorher wissen, wem was gefällt und guttut. das heißt, es liegt ins, in der natur von diesem, von diesem konzept, dass da halt einiges dabei ist, wo man sich denkt: „oh, wuh, zeit, oder?“ * aber an und für sich ist austausch, finde ich, schon, * schon nicht schlecht. und wir sind jetzt halt *. früher ging man auf einkehrtage oder auf klausurtage oder so. ähm, ja, das ist jetzt halt mit corona ganz abgekommen. es ist zu teuer, die personalsituation ist nicht mehr so, dass man sich gegenseitig so leicht freispielen kann. ja, sonst finde ich das eigentlich, dass das schon sehr, sehr wichtig war,

I: mhm.

B: damit, damit man neue impulse bekommt.

I: mhm.

B: weil, im grunde genommen ist das nichts anderes, oder, als ein #austausch.

I: ja.# * genau. und das ist ja das schöne, dass man sich nicht noch mal was neu anlesen muss und irgendwie suchen muss, sondern dass man auch einfach eine, eine möglichkeit hat. und dann erzählt jemand was. „ich habe es so und so gemacht.“ und das muss ja nicht der impuls sein, den ich dann erst in der nächsten unterrichtsstunde umsetze. aber ich habe wieder eine neue idee. oder es ist vielleicht auch mal eine idee, die habe ich #schon

B: mhm.#

I: vor langer zeit mal gemacht,

B: mhm.

I: aber sie wird mir wieder in, in erinnerung gerufen.

B: mhm. also, was, was, was, was ich halt gemerkt habe, was, was, ähm, ganz schwierig ist, einmal zumindest bei meinem kollegium, äh, wir hatten da ja so ein, ein, stärken.café, glaube ich war das oder ein, ich weiß * oder eine, ein perma.modul, ich weiß es nicht mehr genau. auf jeden fall hatte man * fünf minuten ungefähr, drei bis fünf minuten, wo man die eigenen ähm, sachen äh, äh, präsentieren konnte, mit powerpoint, mit video, wie auch immer, oder? und äh, manche haben das halt sehr überzogen.

I: mhm.

B: und das ist jetzt zum beispiel gar nicht gut angekommen bei meinen kolleginnen, oder? ich finde, da, das, das verstehe ich auch, oder? da, da wird dann das ganze schon ein bisschen ausgereizt, die geduld. * so, so nett das alles ist, aber ja, * wenn es die vorgabe (wohl?) gibt und sich die meisten dran halten. (LACHT) also, solche dinge sind dann, wenn es eh schon ein bisschen infrage gestellt wird, oder, äh, sind dann solche dinge einfach schwierig.

I: ja. wie sieht es sonst allgemein mit rückmeldungen zu dem fortbildungskonzept aus?

B: * ja. ähm, ich habe es eigentlich immer ganz angenehm empfunden. ähm, diese online geschichten finde ich sowieso *. wir sind in einer totalen randlage. ich habe sonst immer einen ziemlichen wegezeitverlust. also, das ist für mich äh, ganz gut, äh, von der äußer* also von der organisationsform her.

I: mhm.

B: und, und, und die inputs, ähm, * die perma.post und, und diese kleinen videoimpulse von dem kleinen knietsche, die habe ich auch ganz, ganz äh, angenehm gefunden und genossen. nur waren sie mir ein bisschen zu häufig.

I: mhm.

B: und jetzt ist dafür gar nichts mehr. also, ich hätte es vorgezogen, wenn jetzt zum beispiel nur zweimal im monat was gekommen wäre und das dann aber durchgehend gewesen wäre. weil das ist eine zeit lang massiv gehäuft kommen und dann war fertig. und ich hatte oft gar nicht die zeit. jetzt zum beispiel, letztes jahr um die zeit, war das gerade. und jetzt bin ich so mit, mit organisation, weil ich bin ja die schulleiterin auch und muss voll unterrichten, bin so mit organisation befasst, dass ich oft nicht zeit habe, noch äh, zusätzlich zu meinem, zu meiner unterrichtsvorbereitung, mich noch in videos hineinzuschauen. und vor allem sind es ganz oft dinge, wo ich mir ja denke, da muss man noch ein bisschen weiter bohren, ein bisschen tiefer gehen. und, und das war dann, war dann schade. das ist ein bisschen verpufft. aber ich habe es im hinterkopf. und wenn mir mal die ideen

total ausgehen würden, für, in meiner perma.kiste, dann werde ich wieder den kleinen knietsche aktivieren.

I: mhm. gibt es irgendwas, was sie sich dafür noch gewünscht hätten oder was sie noch gebraucht hätten?

B: 5Ü nein. mh, * also, * bin ich jetzt nicht so ideenreich, das mir einfällt, was, was es sonst noch * an möglichkeiten gäbe. ja, 8Ü ja, nein, das ist schwer zu sagen. vielleicht wäre es noch gut gewesen, äh, äh, sich einmal noch mit den, mit, mit, mit den initiatorinnen, so in einem kurzen äh, äh, gespräch auszutauschen. dieses, dieses video, das hat mir auch ganz gut gefallen, was wir mit den schülern gedreht haben. das war auch ganz nett, ähm, für mich im hintergrund zu, äh, äh, beobachten auch, wie die, wie die frau äh, äh, äh, ivy (?) oder so, wei* weiß nicht mehr genau, irgendwas mit ypsilon war das, äh, wie sie aus den kindern, wie sie die, die interviews äh, geführt hat. das habe ich auch total spannend gefunden. und wir haben dann mit den schülern auch einige davon angeschaut und unser eigenes natürlich auch. also, das war auch ganz eine nette idee. und ähm, ja, vielleicht so einen direkten austausch noch äh, * mit tipps. * aber ja, das ist halt auch immer wieder zeit. also, ich war schon froh, dass wir sonst nicht uns wahnsinnig unterlagen anlesen mussten, zum beispiel. das habe ich sehr positiv empfunden.

I: mhm. * was ja auch wieder zeit ist, die man einfach dann nicht hat, und es noch was #anderes ist,

B: ja, ja.#

I: wenn man sich einfach nur, #nur

B: genau.#

I: impulse anschaut oder nur eine kleinigkeit zum lesen hat und nicht so viel *.

B: mhm. (zustimmend) ja, ja.

I: würden sie die fortbildung weiterempfehlen?

B: * ja, doch. also wenn jemand, mh, wenn jemand diese haltung hat, dann ist es sicher gewinnbringend. wenn jemand ganz anders eingestellt ist, dann weiß ich nicht, ob man ihn zu seinem glück, äh, zwingen kann. also ich glaube, es brau* es braucht schon eine grundbereitschaft überhaupt, äh, genauso wie eine, in eine integrationsklasse einzusteigen oder so als, als unterrichtender. ich meine, wir, wenn wir einfach ein integrationskind bekommen, dann, dann, dann ist das einfach so. (RUFT ANDERER PERSON) sabine? * äh, kollegin hat gerade was gebraucht, aber ist schon wieder weg. äh, dann, dann ist das einfach so, oder? aber ich weiß das von meinem mann, der ist an einer größeren äh, schule und, und sekundarstufe. und äh, da macht es keinen sinn, äh, wenn eine lehrperson nicht in ein, in das integrationsteam äh, äh, s* möchte, also, we* wenn der mit dem nichts anfangen kann, ihn dann s* in so eine klasse rein äh, zwangszuzuteilen. weil, ja, das wird

wahrscheinlich nichts werden. ich glaube, dass das die wenigsten *. ja, es kann sicher gelingen, dass der bekehrt wird, aber, ja, * schwierig. (LACHT)

I: also, gewisse grundhaltung schon so #in die

B: ja.#

I: richtung ist positiv. auch diese bereitschaft, auf das positive zu schauen und nicht nur das #negative

B: ja, ja.#

I: mit leistungen und benoten #zu

B: ja.#

I: schauen, die #ist aus ihrer

B: ja, ja.#

I: sicht schon notwendig?

B: schon notwendig. außer, es gibt so ein initialerlebnis. wo diejenige person halt * grade, äh, sozusagen vom saulus zum paulus wird und durch, durch ein spezielles erlebnis. aber so ein erlebnis, finde ich, kann man nicht äh, zwangsweise herbeiführen. sondern, entweder gibt es das, dann ist es gut, und dann wird vielleicht jemand, der, der da gar nichts damit anfangen konnte, ähm, auf einmal da für sich einen wert erkennen. und ja, und ansonsten halt nicht. (LACHT)

I: ja, auf jeden fall. was würden sie sagen, ist so das, das, der schönste moment oder das, was sie sich jetzt so, ja, am meisten mit herausgenommen haben aus dem, aus dem fortbildungskonzept?

B: * moment kann ich eigentlich nicht sagen. das ist eher ein gefühl, dass, wenn so, so, so ge* gelungene momente sind, wie zum beispiel, wenn wir am, am, * am ende einer woche irgendwie reflektieren und die kinder nehmen eine reflexionskarte und äh, es kann auf einmal jemand etwas dazu sagen, der sonst * überhaupt sich nicht wahrgenommen hat und nur gesagt hat: „das, oh, das coolste für mich in diesem, in dieser woche, sind immer die großen pausen“ oder so, wenn dem auf einmal was einfällt. das finde ich total schön. oder wie ich ihnen gesagt habe, als sich die ganze klasse einig war und so einen, einen schönen, warmen moment gestalten, äh, gestaltet hat, für uns lehrerinnen natürlich in erster linie. aber natürlich auch für sich selber haben sie das ja dann auch gespürt. also, das sind schon tolle sachen.

I: mhm.

B: * oder, wenn eben die kinderaugen so äh, wirklich äh, leuchten und sagen: „bah, frau t, wah, schau mal, das, das kärtchen, das, wie findest du das? das habe ich jetzt für mich gefunden.“ also, das finde ich schon auch schön. also, sind eigentlich so warme momente einfach.

I: ja. was ja dann nicht nur als, als schöner oder warmer moment bei den kindern ist, sondern tatsächlich auch mit, mit uns lehrkräften was macht.

B: ja. ja. * ja.

I: * okay. * gibt es noch veränderungen oder ideen, die sie bei sich in der schule etablieren wollen, die so aus dem ganzen noch entstanden sind, die noch keinen raum hatten oder keinen platz hatten, die in der zukunft vielleicht noch angedacht sind?

B: * nein. eigentlich äh, das, das, das gesamtpaket möchte ich einfach nicht aus den augen verlieren und einfach noch in diese, in die richtung selbstreflexion eben und die stärken wahrnehmen. weil, bis jetzt haben wir vor allem am umgang miteinander und klima und so gearbeitet. und dass uns das einfach guttut, äh, wenn wir gut miteinander umgehen, dass es dann allen besser geht, oder? und äh, ja, aber * schon langfristig ist dann schon mein ziel, jetzt, mit den jetzigen drittklässern, denke ich mir, die haben ja jetzt schon die volle dosis bekommen heuer, so in diese richtung. dass wir dann schon noch mehr eben in die richtung gehen, * wo sind meine stärken und wo kann ich noch äh, mh, äh, mich, also meine, meine, das, was mir gut gelingt, einfach wirklich gut wahrnehmen und dann noch für die gemeinschaft gut einbringen? also ja, schon eigentlich schon in richtung stärken noch mehr arbeiten.

I: mhm. * okay, dann, vielen dank.

B: ja, bitte.

I: ähm, dann würde ich so zum abschluss noch *. * vielleicht finden sie noch so ein, zwei oder drei sachen, wo sie sagen, dafür bin ich jetzt dankbar, das 4Ü hat mich weitergebracht, war für mich wichtig?

B: *8Ü* na ja, einfach zum *. schon wahrnehmen, dass das, dass das ganze immer so in spiralen läuft und wie es so self-fulfilling prophecy, dass, äh, äh, wenn man negativ denkt, dass es einen dann auch ins negative hineinzieht. äh, ähm, also dieses, dieses denken kenne ich ja schon ganz lang, aber dass das nicht eine blase ist, sondern dass das wirklich, dass das wirklich sich bewährt. * aber das es halt ein dauerndes daran arbeiten ist.

I: mhm.

B: also, das ist sicher eine erkenntnis. und ich sage immer: „der teufel schläft nicht.“ ähm, gerade wenn man das gefühl hat, (LACHT) es läuft gut ist, ist die gefahr oft doppelt da, dass es dann

I: mhm.

B: grade, hm, dass man gerade eine hineinbekommt. allerdings habe ich schon auch die erfahrung gemacht, dass dann nicht alles weggespült, sondern dass dann, ähm, dass man einfach ausdauernd

sein muss. steter tropfen höhlt den stein. und dass dann schnell wieder, äh, dass man wieder auf etwas aufbauen kann und nicht wieder bei null anfangen muss.

I: ja.

B: ja.

I: das stimmt. gut, gibt es von ihrer seite noch irgendwas, wo sie sagen, das hat jetzt noch keinen platz gehabt, das würden sie zu dem ganzen noch einfach gerne loswerden oder fragen?

B: nein, nein. ähm, ja, doch, etwas gibt es schon. dieses entrepreneurship, das auch im, im äh, lehrplan verankert ist, im neuen. ja, das ähm, macht mich schon ein, schon ein bisschen stutzig. mit dem kann ich noch nicht ganz gut umgehen. weil ich, mir ist nicht klar, was dahintersteckt. ähm, * sollen unsere kinder jetzt wirklich nur aufs wirtschaftsleben (LACHT) vorbereitet werden und, und, und da möglichst ähm, effiziente leistungsträger werden? also da, da, da weiß ich noch nicht ganz genau, was ich damit anfangen soll, wie ich das einordnen soll.

I: mhm.

B: weil das geht ja dann für mich wieder so in ein, in ein, äh, in so ein, äh, äh, preis-leistungs-verhältnis-denken. so, so möglichst, wie ziehe ich möglichst viel aus irgendwas heraus und in dem fall aus unseren kindern heraus, oder? und das, da, aber vielleicht *. wie gesagt, ich kann's noch nicht einordnen. vielleicht denke ich da in eine komplett falsche richtung.

I: ja.

B: aber es ist ein-, zweimal gefallen, das äh, sind diese schlagworte gefallen. und dann beim lesen von dem, von den, äh, grund* äh, äh, allgemeinen äh, neuerungen oder halt allgemeinem vorwort und, und vom neuen lehrplan, bin ich wieder darauf gestoßen. und da habe ich mir (gedacht?): „aha, in welche richtung laufen wir jetzt, oder?“ * weil mir (LACHT) ist, wenn wir in die richtung äh, äh, laufen, uns soll es allen gut gehen und auch äh, rücksichtnahme und nicht, und nicht einfach schonungslose ausbeutung.

I: * wäre auch nachhaltiger.

B: genau. und genau aus, äh, dafür habe ich ja auch das perma.teach für mich im auge oder für uns im auge.

I: mhm. okay.

B: und das passt so mit, mi* für mich zusammen. aber wie gesagt, vielleicht äh, habe ich das einfach komplett falsch verstanden. ja, da wird es noch irgendwann eine aufklärung geben. (LACHT)

I: bestimmt. muss es ja letzten endes auch. also auf jeden fall. * gut, wie geht es jetzt weiter? ähm, sie werden von mir oder eben vom forschungsteam auf jeden fall ähm, eine, eine, diese, diese

hauptererkenntnisse, also einen kurzen bericht bekommen, was einfach aus der studie so herausgekommen ist. und ähm, wenn sie fragen, im nachgang zu unserem interview haben, dann können sie mir jederzeit schreiben. wenn es so allgemeine zur studie sind, ähm, dann eben zu den ansprechpartnern vom, vom, vom fortbildungskonzept, genau. haben sie sich bei ihnen noch irgendwelche fragen ergeben?

B: * jetzt im moment nicht, wüsste ich nicht.

I: okay, dann ihnen herzlichen dank für das interview.

B: bitte.

I: es hat mir sehr viel spaß, spaß und freude bereitet, ihnen da zuzuhören.

B: danke.

I: und wünsche ihnen jetzt einen schönen, hoffentlich sonnigen, nachmittag, den sie auch ein bisschen genießen können und ein bisschen kraft tanken können.

B: ja, dankeschön. ihnen wünsche ich viel erfolg für ihre arbeit und möglichst viele offene interviewpartner, damit sie auch ihre forschungen entsprechend betreiben können.

I: genau. vielen, vielen dank.

B: bitte.

I: danke. tschüs.

B: wiedersehen. tschüss.

Anhang F: Interview 5

01.06.2023 16.30 Uhr

I: und wie gesagt, es geht dann * geht dann hauptsächlich darum, die erkenntnisse letzten endes, ähm, dass die dann eben weiterverarbeitet werden dürfen. und natürlich alles anonymisiert.

B: soll ich jetzt auf verstanden drücken, oder?

I: genau, nur dann darf es auch wirklich aufgenommen werden.

B: okay. gut.

I: sehr gut. dann vielen dank. dann würde ich sagen, bin ich ganz gespannt, wer sie so sind als person und was sie so als lehrkraft ausmacht. also einfach mal so ein bisschen von ihnen erzählen.

B: hm (zustimmend), okay. also ich bin 44 jahre alt. ich habe zwei kinder, 21 und 16. ich bin seit 24 jahren lehrerin, mit begeisterung. ich * ich erlebe das wunder kind immer wieder gerne. und für mich sind einfach kinder ein unglaubliches wunder. und ich begleite sie gerne. äh, ich habe auch immer bei den fortbildungen mich immer stark fokussiert auf den * auf den sozialen bereich und habe eben in diesem bereich, der jetzt perma heißt, schon viele vorgänge, sage ich jetzt einmal, besucht und gemacht. und mache nebenbei seit einem jahr * also ich bin jetzt im dritten semester des studiums zur psychomotorik. ich bin auch im masterlehrgang. also vielleicht melde ich mich dann bei ihnen, wenn ich dann etwas für meine masterarbeit brauche (LACHT). dann drehen wir es dann um. wo es auch darum geht, also mein masterarbeitsthema heißt dann auch stressbewältigung, äh, von 6 bis 14 jahren und wie man eben die kinder unterstützen kann. genau, äh, natürlich auch über den psychomotorischen bereich. ist ja logisch. sonst wäre es am Thema vorbei. genau, also ähm, ich habe eine vierte klasse gerade eben aktuell. und habe schon die neuen kinder gestern kennengelernt von meinem nächsten rad. wir haben ja dieses vierjahresrhythmussystem, genau. und ja, bin an der schule seit sieben jahren, glaube ich, leitervertretung zusätzlich. und, ähm ja, war jahrelang vertrauenslehrerin. habe aber letztes jahr gebeten darum, dass man mich einmal nicht wählt, einmal mich aussetzen lässt, weil das sehr intensiv ist an der extraarbeit. ja. genug (LACHT)?

I: auf jeden fall schon mal sehr spannend. heißt, sie haben sich mit der thematik, egal unter welchem deckmantel sie sind * äh, sie ist, ähm, positive stärken et cetera schon, schon länger beschäftigt.

B: hm (zustimmend). es hieß ursprünglich ja so ganz zu beginn transaktionsanalyse. dann nachher war es eigenständig werden, dann danach kam faust los. danach kam die gewaltfreie kommunikation mit marshall rosenberg und die resili* resilienzforschung. also so habe ich das ein bisschen dokumentiert. und ich habe in allen bereichen die ausbildungen gemacht, die auf der ph angeboten wurden. genau.

I: wow. war auch schon ganz schön vielfältig. also alles eine, eine sehr ähnliche richtung. aber es ist auch schön, wenn sich dann diese unterschiedlichen, ähm, der unterschiedliche blickwinkel aber immer wieder irgendwo kreuzt und man dann auch immer wieder auf ähnliche erkenntnisse letzten endes kommt.

B: das stimmt. also, es * man merkt immer wieder, wo sich dann wieder überlappt. aber ich finde es einfach wichtig, dass man immer wieder den fokus drauf hinlegt, weil ich halt davon ausgehe, ja, ich kann in dem deutsch, mathe, sachunterricht sowieso immer beibringen. natürlich gibt es ganz viel. ich möchte das jetzt nicht runtermachen. also nicht, dass es dann irgendwo bei dem interview da falsch rüberkommt, wenn sie das aufnehmen (LACHEND), ja? dass es nicht wichtig ist, sich da auch immer wieder weiter fortzubilden. aber wenn man das kind nicht auf seiner seite hat, wenn man eben, äh ja, auf der sozialen ebene die vertrauensbasis nicht geschaffen hat, dann hat das sowieso gar keinen sinn überhaupt anzufangen, einen buchstaben zu lernen. daher ist mein fokus immer, von allen möglichen seiten auf das kind zuzugehen, jetzt eben gerade aktuell eben mit der psycho-motorik, äh, über die bewegung.

I: hm (zustimmend).

B: äh, perma parallel läuft bei uns seit einem Jahr und läuft über diese stärken, äh, Punkte, die ich zwar in vielen bereichen schon kenne, aber immer wieder neue ideen kommen. und jede neue idee ist eine idee mehr. genau.

I: ja, ganz wichtig. und bei uns im elementarbereich heißt es von der beziehung zur erziehung. aber letzten endes lässt sich das genauso auch auf die * auf den bildungsbereich weiter ausdehnen. also ohne beziehung lässt sich letzten endes auch nicht, ähm, gemeinsam lernen oder überhaupt aufs lernen vorbereiten.

B: genau. und inklusion hat uns so schöne gedanken gegeben. und ich stehe auch total dahinter, aber hat uns einfach neue voraussetzungen und neue hürden gebracht, die, äh, die wir da alleine leisten müssen. und das ist nicht einfach. und ich denke mir gerade da, noch mehr dinge im rucksack zu haben ist sicherlich immer hilfreich, ja?

I: ja, das stimmt. ein voller methodenkoffer oder rucksack, wo man dann einfach wirklich situations-orientiert, äh, rausnehmen kann oder bedürfnisorientiert, ähm, rausnehmen kann, was man gerade braucht. * wie sind sie denn auf perma.teach aufmerksam geworden?

B: äh, da muss ich ganz ehrlich gestehen, unsere tutorin hat uns für die pilotschule angemeldet (LACHT). nicht darauf aufmerksam geworden, ich habe das da das erste mal gehört, wie sie es vorgestellt hat mit der hand, vor eineinhalb jahren. kann das stimmen, vor eineinhalb jahren? ist das jetzt her? oh ja, weil wir hatten zuerst dieses pilot ein, äh, diese (?) phase. und losgegangen ist es ja dann so richtig, äh, im juni. mai, juni genau stimmt, eineinhalb jahre.

I: und was war so ihr erster eindruck?

B: ähm, also * hm (nachdenkend). (LACHT ETWAS) der allererste eindruck war insofern, wir waren ein bisschen mit corona, äh, dinge ein bisschen überbelastet. und wie sie gesagt hat, sie hat uns für ein neues pilotprojekt angemeldet, war das so okay, gut. und dann, ähm, war schon eigentlich die erste fortbildung schon, wo halt die, die fünf finger erklärt wurden, mit den, mit den worten und was halt dazugehört. dann war es okay gut, ist grundsätzlich bekannt. eine, eine entspannung dabei. und dieses, dieses säulenprogramm kennen wir ja eben in anderen varianten halt auch, sind halt jetzt finger. das hat ein bisschen entspannung reingebracht. es war dann mit der perma post, ähm, ein bisschen ambivalent, muss ich gestehen. weil es war viel. es war wirklich viel. wenn man sich diese ganzen videos anschaut, hat man immer abwechselnd toller input und nicht wieder was. und deshalb, es war, es war wahnsinnig viel auch, ja? also ich meine, es waren * ein paar videos waren großartig. nicht alle haben mir persönlich gefallen. aber ich muss sagen, das ist auch okay. weil ich finde, man soll sich herausnehmen, was für einen passt. und, ähm, und manchmal war es einfach wirklich viel im alltag. also die post kam sehr, sehr regelmäßig.

I: hm (zustimmend).

B: ja.

I: also da wäre ein etwas längerer abstand und dafür aber insgesamt länger was davon haben, vielleicht die bessere variante.

B: ja, ich denke, wir * also, mir * meiner persönlichkeit käme es mehr entgegen, zum beispiel einmal im, im, im quartal, aber dafür dann mehrere videos und ich kann die durchzappen, wenn ich möchte. ich hatte halt schon immer den drang, oder wir hatten auch den auftrag es kommt, wir schauen es an. und wenn das dann regelmäßig kommt, wir sind ständig, gefühlt, stimmt ja dann wahrscheinlich nicht, ständig damit beschäftigt gewesen, das alles anzuschauen, damit wir am ball bleiben. und so könnte ich mir dann einfach im quartal * ich habe dann die zeit, sage ich gut, heute habe ich hier nachmittags zeit, jetzt schaue ich mir da fünf videos an, zum beispiel. schreibe mir das raus oder kopiere mir die links, die mir gut gefallen und bin ein bisschen autonomer in der, in der bearbeitungsphase, vorbereitungsphase. genau die war bei uns sehr dicht.

I: hm (zustimmend) okay. auch nachvollziehbar. und insgesamt, was * wie würden sie die, die fortbildung, das ganze gesamtpaket mit modulen und, ähm, stärken-café und allem * wie bewerten sie es?

B: also mir hat die, die erste powerpoint, die erklärt, worum es geht, sehr gut gefallen, weil einfach dieses durchführen und wissen, was kommt auf mich zu, das finde ich, es liegt mir auch wieder sehr am herzen, dass ich mich auskenne. das kommt, das heißt, (LACHT) das sind so quasi die etappen, wie sie eigenständig damit arbeiten können und sollen. was mir auch gut gefallen hat war der

zusammenschluss dann mit mehreren schulen, der austausch. weil halt dann wieder ganz viele persönliche ideen dazugekommen sind. ich finde zwar, dass manche schulen das sehr lang gehalten haben. da hätte man vielleicht auch ein bisschen ein zeit-limit vielleicht vorgeben sollen, damit man da nicht dann, ich glaube, das waren vier stunden, fünf stunden, es war wirklich lang, bis dann alles durch war. ähm, aber, aber es waren ganz großartige dinge dabei. auch dinge, die ganz leicht zum umsetzen sind, dazwischen, wo man sich halt dann was neues suchen möchte, weil die kinder auch dann immer neue dinge haben. die ich mir dann abfotografiert habe schon während dem vortrag oder halt aufgeschrieben habe. das war schon sehr hilfreich, ja? das hat mir gut gefallen.

I: hm (zustimmend). gibt es was, was sie sich vielleicht noch zusätzlich gewünscht hätten oder jetzt fernab von der perma gesagt haben, ähm, was noch eine anregung wäre?

B: äh, eine anregung von meiner seite wäre das, es wurde sehr viel von den beiden damen * und ich muss das gestehen, ich weiß die namen nicht einmal, die beiden etwas älteren damen, die das auch initiiert haben und ganz entzückend und toll sind. wissen sie wen ich meine?

I: die, die äh, ingrid teufel und

B: # ja.

I: eva # jambor.

B: ja, danke. äh, sie wollen es zeigen. sie wollen zeigen, was für eine unglaubliche arbeit darin steckt. ähm und das verstehe ich einerseits. es war nur bei jedem stärken.café ein extrem großer punkt der eigendarstellung, wo ich mir schon gerne gewünscht hätte, da steht schon auf der powerpoint, wie der punkt input. ich nehme mir raus, was ich brauche. ich denke mir, ich verstehe, dass sie das zeigen wollen. umgekehrt verstehe ich aber auch mich und alle anderen kolleginnen um mich herum, die so viel bekommen an, äh, an inputs und an aufgaben die ganze zeit. und wir wollen uns dann einfach relativ rasch heraussuchen dürfen, was, was können wir nehmen, wollen wir nehmen. wir müssen das dann eh für uns umsetzen. genau, das war mir teilweise einfach zu lang. da hätte ich mir gewünscht, dass da einfach der* ein, ein impuls steht und ich kann das dann, ob in powerpoint-form oder wie auch immer. und aus dem nehmen wir dann unsere sachen raus, die wir halt brauchen können. genau.

I: hm (zustimmend). gibt es noch andere rückmeldungen an, an material, oder *

B: es gab extrem viel material. ich finde, das buch gefällt mir sehr, sehr gut. die kinder lieben es auch, also darin zu arbeiten. wir, äh, haben tatsächlich fast jede seite bearbeitet und umgesetzt. äh, dazu noch ergänzungen, die ich eben aus diesen vorträgen der anderen schulung, äh, mir herausgeholt habe. weil wir haben ja mit dem angefangen, da war meine vierte in der vierten. und wir sind so ein bisschen perfektionistisch. wir wollten halt dieses ganze Programm in dem jahr umbringen * also unterbringen. daher haben wir jede woche eine perma-übung gehabt und haben das dann

genau getaktet, damit wir das alles schaffen und die kinder einen guten überblick haben. und * also sie haben besonders gern gehabt dieses sich gegenseitig interviewen. das war für sie das größte. und da ist mir auch wieder aufgefallen, wie wichtig den kindern es ist, dass sie da frei durch die klasse gehen, einfach miteinander reden. und aber dieses geziel * also frei, aber doch gefühlt, dass es viel mehr stellenwert braucht. das hat definitiv viel weniger gehabt. das macht man in der ersten klasse finde ich ganz, ganz viel, um sich zu kennen * also kennenzulernen. in der zweiten auch noch, damit man das da vielleicht ein bisschen dokumentieren kann. in der grundstufe zwei ist es bei mir sicherlich viel mir untergegangen. und das war schön zu sehen, wie gerne die kinder das noch immer machen und dann von sich gegenseitig ein profil erstellen und das andere kind vorstellen. noch einmal, obwohl wir schon vier jahre zusammen waren. also das war eigentlich total schön. und da waren so ein paar aha-erlebnisse, die * auch wie schwierig es manche kinder finden, aus diesem ganzen wörter-pool sich ihre wörter herauszusuchen, was alles sie beschreibt. sie so, mehr als fünf, oder? (LACHT ETWAS) dass war dann oft so. also die kinder sind gewohnt, minimalistisch zu arbeiten. das geht sich in der zeit sonst gar nicht das aus. und dass da eigentlich so viel mehr steht. du bist das alles, ja? und wie cool eigentlich. und da darfst du das wort nehmen und das und das und das. also da haben wir schon ein paar schöne ergebnisse gehabt. und da war in dem buch dann sehr, sehr viele inputs drinnen. ich war weniger auf diese, ähm, wie haben sie es vorher genannt, die sagen, das war nicht padlet, aber irgendwo stand das vorher drinnen, diese * ich habe diese befragung auch gleich gemacht von perma.teach davor. ähm, aber es hieß nicht padlet. oder war * ich komme darauf auf diese eine seite, das muss ich gleich dazu sagen. ich war bei den videos eben, die abgespeichert waren, habe geschaut, was passt. und habe dann eins vorgespielt. und dann, äh, eben viel mit dem buch gearbeitet. und mit den, äh, inputs, die ich bekommen habe von den stärken-cafés und von diesen treffen. deswegen kann ich von dieser internetseite ehrlicherweise nicht so viel sagen.

I: das ist auch völlig in ordnung. also das ist ja das schöne, dass man * jeder sich das raussucht, ähm, was er brauchen kann. und für, für manche ist es schön, da in padlet, oder ich glaube, es ist tatsächlich padlet, die plattform, die man wirklich einfach überall durchscrollen kann und ganz, ganz viel findet und jeder was einstellen kann. ähm, aber für manche ist es das einfach nicht. und für manche ist es dann einfach der persönliche austausch auch nochmal ganz, ganz wertvoll. und so darf es ja auch sein. so unterschiedlich wir ja auf die kinder eingehen und schauen, was die für bedürfnisse haben, so geht es uns ja auch, dass jeder einfach auch was anderes anspricht und, und einen anderen input auch für sich gut annehmen kann.

B: hm (zustimmend). ich glaube auch, das ist ein bisschen der punkt. weil wir hatten parallel dazu, und dafür kann wieder natürlich perma nichts, dauernd den auftrag, auf diese plattform gehen zu müssen. die ist ja * ich weiß nicht, ob sie die kennen, die ist monströs groß. und wir mussten uns dann da zurechtfinden und dann einen, einen feedback-bogen, weil wir hatten auch den auftrag, war

nicht so spürbar, aber auch extrem schön. also ich habe * ich mache gern feedback in der klasse und mit dem, äh, ding. aber wir waren dauernd auf so plattformen drauf. und diese plattform ist wirklich riesig. und dazu noch mit dem neuen lehrplan, dass ich * dann ist die total untergegangen. weil grundsätzlich merke ich das, wenn ich aus einem vollen pool einfach raussuchen kann, dann schöpfen kann, habe ich ja auch vorher schon erwähnt. Aber das ist mit den anderen plattformen, die jetzt parallel in dem jahr so extrem gelaufen sind, sicherlich bei mir zumindest dann untergegangen. also ich möchte schon dann * und das sage ich deswegen, weil ich eigentlich solche plattformen gerne mag. nicht, dass es dann als rückmeldung so kommt, dass das für mich, ja, nichts ist.

I: ja, während die plattform ja bestehen bleibt. also man hat ja auch jetzt die möglichkeit, zu einem späteren zeitpunkt auch nochmal drauf zu schauen.

B: hm (zustimmend).

I: bei ihnen an der schule haben ja mehrere oder dann alle teilgenommen. als pilotschule haben ja alle dann teilgenommen.

B: hm (zustimmend).

I: wie, wie war der austausch unter * also innerhalb des kollegiums?

B: äh, in den * also ich kann nur von mir in meinem, äh, jahrgang arbeiten wir zusammen, so alle vierten klassen. da war es sehr intensiv. wir haben auch die gesamte perma, ähm, vorbereitung gemeinsam gemacht. und wir wollten eben alle das so durchkriegen. deswegen haben wir uns da alle ausgetauscht. wo wir dann auseinandergegangen sind war, wer was macht von dem, äh, persönlichen austausch. weil da sind * wir sind * wir sind unterschiedlich angesprungen auf gewisse dinge. also da * das war auch schön. weil man muss nicht alle alles gleich machen. äh, sonst kann ich sagen, im lehrerkollegium war definitiv meiner ansicht nach, aber vielleicht ist das jetzt auch ein bisschen gefärbt und unfair, oder ich weiß es nicht, aber, ähm, das feedback oftmals positiv, äh, vom nachmittag. wir haben ja die freizeitbetreuerinnen auch dazu geholt. da war, zumindest was ich mitbekommen habe von den kleinen pulk (?), aber ich kann wirklich nicht für alle reden. wir haben da 30 leute und ich habe jetzt nicht zu allem immer kontakt, ja? äh, war das ein bisschen, ähm, distanzierter, weil sie auch von der primär (?) so viel * also gerade in dem jahr, dieses pädagogische buch dazu bekommen haben und das wieder alles so parallel war. und das war einfach nicht so einfach. ich meine, da schreibe ich eh schon das nach der auflage von dort. und das war nicht immer so einfach zu kombinieren. genau, also ja. somit war das auch ein bisschen getrennt. es kommt aufs team an. zum beispiel mein team, also meine freizeitbetreuerin, wir machen damit meinen kindern sehr viel zu dritt. auch schon am vormittag. und sie hat es dann fertig gemacht. das heißt, ich kann bei meinem team sagen, wir haben einfach am vormittag angefangen und oft den nachmittag weiter gemacht und fertig gemacht. war sicherlich aber jetzt nicht generell usus.

I: hm (zustimmend). was aber schön ist, wenn auch die kinder erleben, dass es da einen * eine * einen roten faden gibt, dass das einfach auch weitergeführt wird. dass das jetzt nicht nur sehr isoliert betrachtet ist, sondern einfach, ja, weitergeht.

B: hm (zustimmend).

I: okay. das heißt, sie haben ja vorher gesagt, dass ihre, ihre, ähm * dass die schulleitung letzten endes sie angemeldet hat. wie war dann so, so von, von der schulleitung her die unterstützung oder die begleitung? gab es da irgendwie *

B: ja, es war auf jeden fall so, dass es jede * bei jeder konferenz thema war. also jede konferenz war ein punkt perma. sie hat * war ja auch immer dabei. Also sie war bei jeder fortbildung dabei, bei jedem stärken-café dabei. somit war das auch immer up to date und auch immer auf das letzte bezogen, was wir gehabt haben. sie hat auch oft schon vorab die videos alle schon durchgecheckt und uns dann auch gesagt, wenn zum beispiel wieder mal viel war in einem moment, wo wir zeugnisse geschrieben haben oder so weiter. die sind besonders gut. die sind aufgefallen, hat dann schon quasi ausgefiltert manchmal. das war teilweise sehr hilfreich. und ähm ja, sie hat unterstützt auch, dass wir * wir mussten jetzt ein interview vorbereiten mit einem kind und so weiter, damit da auch überall immer zeit und platz und so weiter war und auch eine lehrerin da war, die vielleicht mal ablöst, damit man mit einem kind wohin gehen kann. das hat auch gut funktioniert.

I: okay. das heißt, da haben sie auch, soweit es möglich ist, einen rahmen bekommen, in dem man das auch umsetzen * gut umsetzen kann.

B: genau, diese extras, sage ich jetzt mal, die dann einfach nur manche kinder betroffen haben. ja.

I: ja, okay. was ja ganz, ganz wichtig ist. ja. gut, dann gibt's noch was, was sie zur fortbildung * irgendwas wo sie jetzt noch sagen, das braucht noch eine rückmeldung.

B: nein. also was ich, äh, jetzt nicht ganz sicher bin, ich glaube, wir haben eh am schluss bekommen, dass das * also diese internetseite, ich glaube eben, dass das padlet offen bleibt, für uns in zukunft bleibt, dass wir weiterhin diese bücher bestellen können, glaube ich. aber ich muss sagen, äh, wir haben den abschluss noch nicht gemacht. und da gibt's auch so glaub ich noch irgendwas bin ich jetzt falsch informiert, dass man einen abschluss, äh, treff mit * oder? oder nicht?

I: da weiß ich es * da weiß ich es # leider nicht.

B: oder höchstens # bei der konferenz. oder es ist bei der konferenz der letzte punkt, wo man dann sagt, so wie geht es im nächsten jahr weiter? wir bestellen das weiter und wir haben es dort. also irgendwo ist noch bei uns ein Punkt, wo perma quasi als piloting abgeschlossen wird, und wir besprechen, wie es dann einfließt, äh, im nächsten jahr. weil wie wir zu den ganzen sachen halt dann kommen.

I: ja, genau. ähm ja. dann, ähm ja, wäre es noch so ein bisschen so die, die umsetzung. sie haben ja immer schon wieder ein bisschen was einfließen lassen, wie sie es umgesetzt haben, was ihnen wichtig ist. aber da wäre ich einfach noch neugierig, wo sie sagen, das war für die kinder besonders wertvoll, oder es war für sie, ähm, einfach auch sehr wertvoll.

B: hm (zustimmend). also ich sage, das sind die größten sachen, die mir jetzt aufgefallen sind, die mich dann auch überrascht haben, waren die geschichten mit dem interview. und auch das, äh, das sich selbst beschreiben mit * also sich selbst sehr umfangreich beschreiben mit adjektiven. wir machen das seit der ersten klasse eh gern, aber sich dann noch mehr zeit nehmen und noch mehr und noch mehr worte zu finden, das war für mich die größte überraschung. wir machen sehr viel mit komplimente geben. das haben wir auch teilweise in anderen varianten gemacht. also ich hatte für komplimente boxen, natürlich in einem couvert. aber da gab es jetzt viel mehr möglichkeiten, noch über, ähm, perma. das, das mit der dusche hat ihnen gefallen. und, äh, wir haben das noch ein bisschen abgeändert mit dem du bist super spiel vor weihnachten. und ähm, also quasi das gleiche nochmal. sie haben * ist im prinzip was wie mit eben ich gebe meinen mitschülerinnen, mitschülern ein kompliment weiter und mit auf den weg. und jetzt am schluss noch mal so mit dem erinnerungs- und komplimente-buch, damit sie es dann mitnehmen können in die nächste schule. ähm ja, die stärken haben die gemacht. und die haben wir auch in mehreren varianten aufgebaut, dass die kinder so ein stärken-buch sich selber gestalten, wo sie, äh, anfangen, eben mit diesem blumenteil, was dann * das in dem buch eh drinnen war. und dann haben wir das erweitert, weil ich eben gemerkt habe, dass es den kindern total schwer fällt, mehr also aufzuzählen als die fünf. und, äh, sich so richtig mal einzulassen auf das ganze. ist also ein büchlein, das haben wir dann gebunden. äh und dann geht es eben, was kann ich gut und was mache ich gerne. was macht mir freude und ähm * also sehr viel positives, aber auch eben, wo sage ich nein. also wir haben das dann dazu noch hineingepackt. wir hatten dann eben ja auch den workshop, ähm, wenn, äh, ich, ich darf nein sagen. mein körper gehört mir, wo es eben geht um grenzen abstecken. das haben wir dann auch noch dazu hineingepackt, genau. dazu haben wir noch die gewaltfreie kommunikation, die wir dann auch immer noch ein * das ist ja bei uns ein qualitätsmanagement-punkt, der ja schon seit jahren läuft, wo wir die sonnenstrahlen haben. sie haben ja noch dazu gepackt, ich will leuchten, wie die sonne. ich will das ausstrahlen. ich möchte nicht verurteilen, aber ich möchte ansprechen. und das konnte man wunderbar mit diesen perma-punkten da halt einfach immer kombinieren. und äh, die kinder haben extrem viel gemalt. wir haben das jetzt auch in einer musik, die noch ein bisschen, ähm, da musiziert mit so liedern, wie du wirst gesehen. und ähm ja, das auch noch vorgetragen. wir haben viel mit kontrafaktur gearbeitet, wo wir lieder mit unseren eigenen texten umgemodelt haben, wie let it shine. und was wir alles brauchen, damit wir halt strahlen können. ja, das sind so die vielen punkte, die wir * wo wir perma halt gelebt haben. und diese dinge. also im * es ist schwierig. ich kann jetzt nicht nur perma nennen * weil wie gesagt, wir haben klar perma, wir haben resilienz, wir haben die

bilder aus der transaktionsanalyse hängen bei uns an der wand. zum beispiel das du darfst wachsen, wenn sie das kennen. mit den ganzen bereichen und dem eigenständig denken. und * also da ist so viel drin. das ist alles wie so eins.

I: ja.

B: genau.

I: da greift ganz viel ineinander.

B: richtig. und wir haben jetzt auch mit dem jahr angefangenen, das auch ein teil meiner Praxisarbeit mit dem sich freispielen. das kam dann wieder zur psychomotorik dazu. und das hat den kindern dann auch so gut gefallen, dass gleich frühdienst genutzt wurde für große fünf stationen, wo sie sich in, äh, allen varianten freispielen durften. sandspiele, bewegungsspiele, eine kuschelecke, halt dann die tut das und die ganzen sachen. das alles, was sie halt brauchen, dass sie sich wohlfühlen. zusammen oder manchmal alleine, genau.

I: in allen schulen ist ja großes thema dass * dass es wahnsinnig viel lerndruck oder stoffdruck ist, dass man manchmal den eindruck hat, man kann gar nicht noch irgendwas dazu machen. ähm, wie ist es ihnen gelungen, das so gut einzubinden? weil das, was sie so erzählen, das ist ja doch recht viel, was sie von den unterschiedlichen aspekten gut mit einbauen. wie ist ihnen das gelungen?

B: ja. also ähm, ich habe im frühdienst angefangen. ich habe jeden tag frühdienst. um 07:15 uhr hat das ganze programm begonnen. ähm, es ist mir gelungen, weil eine vierte klasse, wir sind ein sehr eingespieltes team, äh, super mitmacht. wir hatten extrem viel zu lernen. ähm, wir hatten die ganzen icm (?) testungen plus die schularbeiten, plus noch andere extras mit dem wiener lesetest und so weiter. es war wirklich viel. ähm, wir haben versucht einfach, dass wir perma und resilienz in alles hineinpacken, was nicht deutsch, mathe, sachunterricht ist. also ich muss * das muss ich wirklich ein bisschen * es war in zeichnen, das war in der musik, da haben wir uns wirklich alle sorgen von der seele gesungen. und deswegen habe ich so viele lieder umgetextet mit ihnen, damit die kinder einfach das überall einbauen können. manchmal wollten sie ein lustiges lied dichten oder texten, und manchmal wollten sie zum beispiel wie bei corona, ähm, den frust singen. also wir haben es einfach in alle anderen fächer hineingepackt. also beim werken, wir haben extrem viel in dem jahr auch, äh, mit freiem werken, ohne jetzt klassische werkstücke gemacht. das war für mich auch neu. das habe ich noch nie so offen werken gemacht, wo wir einfach alles hingelegt haben. so, wir bauen jetzt mal was. was bauen wir denn? wir bauen eine stadt. wir bauen ein * also alles mögliche haben wir gebaut mit, mit allem. (LACHEND) eine höhle. also ja. und ich finde, das war dann möglich, da hinein das alles, äh, zu verpacken, ja? also sicherlich war* perma eine fixe stunde auch. Also dieses perma, tatsächlich arbeiten am buch, war eine fixe unterrichtsstunde. die haben wir uns einfach rausgenommen vom sachunterricht. also das war jeden montag in der zweiten stunde fix. und dann war das ein teil von der freiarbeit, genau.

I: sehr schön. ähm, warum würden sie sagen, braucht es die positive bildung oder dieser positive blick, ähm, auch für die lehrkräfte?

B: ja, * weil *

I: was * macht das mit denen?

B: erstens einmal weil, ähm, die anforderungen werden extrem hoch, höher, am höchsten. also es wird immer mehr auf uns, äh, auferlegt in meinen augen. und, äh, ich finde auch, dass die umstände, wie wir die kinder bekommen, teilweise sehr auseinanderdriften. äh, ich habe, wie gesagt * ich meine, ich habe angefangen vor 24 jahren in einer, äh, integrationsklasse. und wir waren zu hundertprozent zu zweit. also da, da hatte ich autistische kinder zum beispiel drinnen. ein autistisches kind zählt als hundertprozentiges inklusionskind, egal in welcher stufe. ähm, und ich bin alleine. also wenn ich glück habe, bekomme ich für drei stunden die woche eine, eine unterstützung. und das war es. also ich bin grundsätzlich alleine mit 25 kindern. teilweise auch schon aufgehoben. bei dem letzten rad waren wir bei 26 kindern, weil die klassen voll waren. ähm, plus diese unglaublichen anforderungen, was wir alles dokumentieren sollen und müssen, äh, in wirklich unterschiedlichsten bereichen. und wenn wir da nicht selber bei uns selber und bei den kindern diesen positiven blick bewahren, dann neigt man vielleicht dazu, manchmal unterzugehen in dem ganzen. also ich finde es extrem wichtig. auch, dass sie sich zeit nehmen, das kind anzuschauen, jedes einzelne. ich denke mal, was, was mir hilft, wenn ich mir denke gut, ich weiß nicht, ich nenne jetzt einfach mal der maxi, der ist heute wieder unglaublich laut und dann schaue ich ihm einfach in die augen. und ich schaue mal und versuche herauszufinden, wie geht es ihm. was ist da gerade bei ihm los? das macht ja kein kind absichtlich. aber ich muss mir dafür zeit nehmen. und, äh, ich finde, dass diese ganzen bereiche mich einfach immer wieder daran erinnern, egal was wir für einen stress haben, die nehme ich mir einfach. die autonomie habe ich noch. und alleine das macht es ja schon so wertvoll.

I: hm (zustimmend).

B: * und auf mich selber schauen. wobei ich ehrlich * äh, ehrlicherweise zugeben muss, sorry, ich fange zu stottern an, dass ich auch das oft vergesse. also ich, ich müsste perma manchmal bei mir anwenden, ja (LACHT).

I: ja, das ist jetzt ganz, ganz wichtig. nur wenn, wenn wir es für uns auch immer mal wieder schauen, ähm, dann können wir es letzten endes auch authentisch bei den kindern anwenden. das ist ja bei allem. egal, ob es partizipation oder resilienz ist. oder wir sind ja doch auch einfach in der vorbildfunktion. und die kinder spüren ja sehr genau, wenn, wenn wir da einfach auch nicht echt sind. und das im alltag auch noch, ähm, bewusst zu leben und auf sich zu schauen. das ist gar nicht so einfach.

B: ja, absolut. vor allem, wenn man auch, äh, leicht perfektionistisch veranlagt ist (LACHT). dann wird es schwieriger.

I: das stimmt. gibt es denn irgendwas, was ihnen persönlich besonders gut geholfen hat? also einfach jetzt nur für sich, wo sie sagen, da habe ich mir jetzt ganz viel mitgenommen für, für meine lehrerpersönlichkeit.

B: ähm, ich finde, dass ich * äh ja, dass ich * dass die kinder nicht zu groß sind für manche dinge. dass man nicht vergessen das darf, dass auch die * die kinder sind auf der einen seite wirklich groß. also ich denke manchmal auch, wenn ich da jetzt in die klasse schau, vielleicht bildet man sich das auch ein und es stimmt nicht. aber ich finde, sie sind größer als vor 14 jahren oder 24 jahren. viele zumindest. und dass man das nicht * äh, und sie sind auch manchmal in der entwicklung schon so fortgeschritten. wenn ich denke, ich hatte jetzt in den letzten monaten immer wieder kinder, die haben schon die menstruationsblutung zum beispiel gehabt in der vierten klasse. und man neigt vielleicht manchmal dazu, die dann noch größer zu machen, sind sie aber nicht. und dass ihnen das noch alles gut tut. und dass sie das brauchen, auch in einer vierten klasse noch. das glaube ich, das war das, das schönste gemeinschaftsgefühl, was wir in dem Jahr jetzt dadurch noch bekommen haben, wieder zurück zu steigen und zu sagen, ah, und das tut uns aber noch immer gut und sie mögen das. und vor allem gerade im zweiten halbjahr nach ostern, wo die kinder mit einem bein schon in der nächsten schule stehen und eigentlich schon wieder weiter sein sollen. aber eigentlich wollen sie gerne nochmal ein stück zurückgehen und diese ambivalenz die ganze zeit. da hat es eigentlich besonders gut getan. und jetzt gehen wir nochmal zurück und setzen uns zusammen und jetzt kümmern wir uns um uns. und ich habe auch wirklich dann noch ein paar übungen zu dem, zu dem ‚ich‘ alleine gemacht. weil eigentlich machen wir in der grundstufe zwei in erster linie das ‚wir‘. und die grundstufe eins ist ich. aber sie haben das ich nochmal so gebraucht. und das fand ich eigentlich sehr spannend. ja, und, und schön, dass wir das nochmal damit, mit diesem buch, muss man ehrlicherweise sagen, da so herausgekitzelt haben, genau.

I: sehr schön. das heißt auch für einen selber, nochmal, nochmal andere prioritäten setzen und vielleicht nochmal viel sensibler drauf schauen,

B: ja.

I: was brauchen die kinder. also ja, wir haben den lehrplan, den muss man erfüllen. das hilft halt leider nichts, auch mit vergleichsarbeiten, die halt einfach vorgegeben sind. aber womöglich einfach auch nochmal auf sich und auf die kinder schauen. und zu schauen, was brauchen wir eigentlich gerade, um das andere, was ja erwartet wird, auch leisten zu können.

B: hm (zustimmend).

I: haben sie da auch eine veränderung bei den kindern festgestellt? also so im vergleich zu vorherigen jahrgängen.

B: ich finde es total schwierig in diesem rad, weil durch corona, das ist ins lernen * diese vielen monate, ähm, das so außergewöhnlich anderer ablauf war, dass ich jetzt * ja, das war alles ganz anders. die kinder waren da, dann waren sie * in der ersten klasse hatten wir corona. das heißt, äh, ich habe sie gerade ein halbes Jahr gekannt, wir sind gerade angefangen, klassengemeinschaft zu werden und dann waren sie quasi nach ostern weg. also bis auf die betreuungskinder. dann habe ich das über ms teams gelöst mit den kindern. da waren sie wirklich, manche haben geweint, wenn sie mich gesehen haben, weil sie gesagt haben * also auf der straße, ich wohne quasi hinter der schule in der siedlung, da, wo die kinder auch wohnen. beim spazieren gehen, mich gesehen haben, ich darf nicht hin, aber sie ist da. und das war total schwierig. sie wissen ja auch nicht alle, wo ich wohne. aber ich war trotzdem nicht greifbar. und das hat alles so schwierig gemacht. ja und dann nachher wieder zueinander finden im sinne von nicht, dass sie nicht sofort sich so gefreut haben und die, die sind in einer riesensiedlung. somit kennen sich die kinder schon ganz lange und davor schon fast alle. ähm, sondern dass sie es wieder durchhalten. weil ein schulvormittag ist lang, das braucht training. und dann haben wir wieder von vorne gestartet, weil die kinder müde waren. wie am anfang in der ersten klasse, in den ersten wochen. dann nochmal von vorne damit zu starten, dann diese langzeitschäden, dieses angst haben davor, wann kommt der nächste lockdown. das hat in den kindern viel ausgelöst. dieses dauernd eine maske tragen und die begleiterscheinungen dadurch. äh, alles abgesagt. wie viel, äh * ganz viele ausflüge, die wir nicht machen durften. und danach versucht haben, so komprimiert in einen bereich zu legen, ja? also das ist alles so anders gewesen. echt schwierig. ja, ich finde schön, dass wir es geschafft haben, trotz alldem so eine tolle klassengemeinschaft zu sein und so viel zu erleben. äh u * ja, trotz corona.

I: ja. wo sie ja vollkommen recht haben. es braucht danach * hat danach eigentlich nochmal eine ganz andere * ein ganz anderes einarbeiten wieder in die klassengemeinschaft und in die struktur gebraucht, als, als man vielleicht normalerweise gebraucht hätte. weil man halt wirklich wieder neu anfängt in manchen punkten.

B: ja. weil die betreuungskinder waren ja immer da. das waren sechs, sieben kinder, die waren immer da und die anderen nicht. also das hat ja was bewegt, hat das gleichgewicht ein bisschen gestört. und das musste man wieder alles (LACHT) wieder dann nachher neu, ja, mischen, bearbeiten, stärken.

I: (LACHT ETWAS) was würden sie denn sagen, sind so die chancen, aber auch die grenzen, die perma.teach einfach * oder perma, positive bildung wie auch immer, einfach hat?

B: ähm, perma hilft sicherlich extrem, äh, schöne momente zu gestalten, den kindern vor augen zu halten, äh, was, was bin ich, was kann ich, was bin ich * selbstwirksamkeit ich meine, das kommt das das wort, das alles umfasst, ja? und äh, auch einen, einen grundstolz und selbstbewusstsein zu, äh, bilden, zu zeichnen, zu besprechen. und das ist für die kinder wirklich gar nicht so leicht. äh

und einfach den unterricht auch immer wieder in diese richtung zu fokussieren. einfach, weil´s dazugehört. und wenn man sich dann zeit nehmen muss unter anführungszeichen, weil es einfach ein teil vom unterrichtsprinzip ist in der schule. äh und wir dann nicht darauf vergessen im alltag, wenn wir dann doch im sachunterricht die wien-wochen (?) haben, oder ich weiß nicht. der, der schmetterling und wie er sich da verändert. also dann gibt's einfach den fixpunkt. und das ist toll. was * wo die grenzen sind, ist ähm, es ist trotzdem, ähm, unser schulsystem. das wird dadurch nicht verändert. sie haben trotzdem die schwierigkeiten der, ähm, permanenten Leistungsbeurteilung zu bewältigen. das kann ja man nicht wegnehmen. wir können sie stärken, ja? aber, äh, sie müssen das trotzdem stemmen. und sie werden beurteilt, permanent. ja, quasi vom ersten schritt in die klasse hinein. und, äh, das ist auch meine größte kritik an dem schulsystem, aber ich habe eh nichts zu melden. ich kann nur versuchen, soweit ich eben das in meiner macht liegt, im alltagsunterricht, äh, entgegenzuwirken. tatsächlich aber bleibt es dabei. wir geben die noten, wir machen schularbeiten, machen lernzielkontrollen, wir schicken die kms weiter. und die kinder bekommen dann eine stufe, wo sie stehen. und das ist halt schon schwer zu hinterfragen in meinen augen. und ja, und da sind absolut die grenzen.

I: hm (zustimmend). * ja. da kann perma.teach nur einen, einen, einen kleinen gegenpol schaffen, indem man eben nicht, ähm, nur dieses bewerten und noten und druck hat an der schule, sondern dass man auch trotzdem, ähm, es schafft, momente zu, zu ermöglichen, in denen die kinder wachsen dürfen und in denen sie sich entfalten können. und nicht nur so, wie, wie ziehen, das muss jetzt alles funktionieren.

B: ja.

I: weil letzten endes sollte ja schule ein ort sein, an dem ich wachsen kann. und nicht nur schule ein ort, wo, wo ich vom ersten schritt an eigentlich bewertet werde. was natürlich unser schulsystem, also sowohl das deutsche als auch das österreichische, da sind sie leider nicht, nicht sehr unterschiedlich, ähm, einfach immer auf leistungen aus ist. und ich sehe das selber bei den, bei den korrekturen. okay, ich nehme keinen rotstift mehr, wir korrigieren alle eigentlich in grün oder in lila oder völlig wurscht, aber halt einfach nur nicht rot. aber trotzdem ertappe ich mich auch immer wieder dabei, dass, wenn du korrigierst, dass du halt immer hauptsächlich logischerweise hinschreibst, was hat denn eigentlich gefehlt, was bräuchte es denn, ähm, damit es besser wird. dann habe ich irgendwann angefangen, okay, und dann schreib es wenigstens auch dazu, wenn etwas wirklich gut ist. aber das ist ja auch wieder so, wo man bei mir * wo ich dann bei mir merke, das ist ein enormer mehraufwand, weil alleine das alles schon zu kommentieren, dass die bei uns, wenn sie das durchlesen, auch eine Vorstellung haben okay, in die richtung kann ich mich noch entwickeln. also da ist so. das wäre jetzt noch eine möglichkeit. und ich versuche immer, anreize zu bieten. also nicht nur das ist falsch, sondern einfach so impulsfragen nochmal zu stellen, dass sie eine idee haben, uns selber wieder die nächsten schritte gehen können. aber in erster linie ist es natürlich defizitorientiert.

B: ja.

I: und braucht aber auch zeit. also ich finde auch da, ähm, wenn's du wertig rückmeldest, das kostet auch viel zeit. und wenn man sich dann immer wieder bewusst macht okay, nicht nur wertig negatives rückmelden, sondern auch bewusst rückmelden, was ist denn gut gelungen, das kostet nochmal mehr zeit.

B: das stimmt. und wir hatten ja einen schritt nach vorne und haben ihn dann wieder zurück gemacht, weil ich denke an die alternativen beurteilung * beurteilungsformen zum beispiel die kdn (?), äh, das hat schon gut funktioniert. das konnten wir jetzt echt in den zwei jahren schön gestalten. und ich zum beispiel, bei mir war das so, ich hab das ja * ich hab kdl (?) gehabt, jahrelang, und habe dann * also zwei waren hintereinander, muss man sagen, ja. habe den eltern gesagt, äh, ich bin sehr stark dafür. ich möchte das machen. und in dieser zeit werde ich nicht über eine note sprechen, weil wir sind alternativ unterwegs. ähm und da konnten wir das wirklich umsetzen, dieses ich zeige das, ich habe * das kann ich, das mache ich, das * so weit bin ich. wenn die kinder das dann präsentiert haben, herrlich. und dann kommen sie jetzt mit dem, äh, eigenständig, selbstständig erreicht und so weiter, was ist das? also ein riesenschritt zurück. weil dann kann ich gleich noten geben. erstens schaut das scheußlich aus. ich weiß nicht, ob sie das schon gesehen haben, die zeugnisse, wie die jetzt ausschauen bei uns. also früher stand dann dort, ähm, nach kommentierte leistungsbeurteilung, ähm, zum beispiel so quer drüber, wenn die kinder in der grundstufe eins das eben * äh, dieses, dieses beurteilungssystem hatten. und dann war das in diese gespräche gelegt. also kinder, eltern, lehrer, wo wir das einfach genau da * die kinder haben gezeigt und ich hab dann an einem kompetenzraster gezeigt, ähm, diese kompetenz hat das kind schon erreicht. also da sind wir schon auf einem guten weg. und da war wirklich der blick auf das positive. also dass wenn wirklich ein, ein großes defizit war, dann habe ich das extern wo noch aufgeschrieben und gesagt zu den eltern in einem direkten gespräch, das braucht's noch. aber die kinder haben das gezeigt, was sie können. und das war cool. ähm, jetzt, wenn wir das jetzt machen, so kdl (?) gibt's nicht mehr. es gibt das pensen Buch (?) äh, wo wir halt so ein, ein rasterbuch machen, was eigentlich eh schon jetzt wieder obsolet ist, weil wir so oder so die kompetenzraster austeilen müssen. aber * und dann steht * muss man dazu zu diesem rasterzeugnis dokumentieren, ist das kind jetzt in diesem fach eigenständig, selbstständig, hat es das erreicht oder nicht erreicht. slso quasi noten. und das steht dann auch so schwierig dort. das ist ein riesengroßer satz. wir haben das das erste mal * wir haben das ja nicht gewusst, hat uns ja kein mensch gesagt, wir machen alternativ. wir geben das ein. und sage, warum können wir das nicht eintippen, so quer nach, ähm, alternativen dingen. dann kommt das und fragen wir nach. dann nach * bei dem zeugnis schreiben schon. also das muss man sich mal vorstellen, ne? statt dem. äh und dann kam das. und das war so schwierig. da stand dann in jeder zeile bei der note, zum beispiel sachunterricht und da stand so ein satz, den eh keiner verstanden hat. letztendlich war der inhalt, ihr kind hat in dem bereich sachunterricht, äh, die lernziele selbstständig erreicht.

oder erreicht. oder * also im prinzip benotet. urscheußlich, wirklich hässlich. und die eltern sind gestanden, was heißt das? weil die konnten jetzt, den unterschied zwischen eigenständig und selbstständig hat keiner gecheckt, ja? also das war alles ein drama. dann haben wir gesagt, na gut, solange sie das nicht ändern, dann gebe ich gleich noten ab der ersten klasse. weil ich, ob ich jetzt das hinschreibe oder das ist egal, ja? und die eltern, die, die übersetzen das. sie wollen wissen, was das heißt.

I: ja klar.

B: auch fürchterlich. also wie gesagt, ich finde, das ist alles so eine tolle initiative, die es da gibt. ich unterstütze das, und ich finde das großartig. es fehlt einfach, das parallel das schulsystem mitzieht. weil das wäre dann einfach die idealform. aber ich hab das gefühl, da versucht * der zieht dorthin, und die anderen versuchen, ein bisschen dagegen zu ziehen. und es ist einfach ein * und so fühlt man sich auch ganz oft. also ich fühle mich genau da in der mitte zerrissen zwischen ich versuche, ihnen das zu geben und da mitzuhalten mit den kindern, damit ich ihnen die bestmögliche, äh, schule weiter wieder ermöglichen kann. ja, der auch wichtig ist.

I: ja. das ist es ja, dass man auch immer nur für den eigenen bereich irgendwie schauen kann, was kann man machen, was, was, was * wo sind die möglichkeiten. aber es geht ja auch weiter. und, ähm, es bringt ja nichts, wenn man jetzt in der einen * in dem einen abschnitt nochmal völlig neue sachen machen könnte, und dann müssen sie aber über die sommerferien sich wieder ein ganz neues system gewöhnen, das nicht kompatibel ist. also das ist * das müsste von grund auf dann einfach schritt für schritt weiterentwickelt # werden.

B: genau. #

I: und das wäre ja schön. ich meine, der grundgedanke ist ja auch die positive bildung, äh, nicht nur in den pilotschulen zu haben, sondern als, als haltung ins system reinzu * ins bildungssystem reinzubringen, womit ja viel einhergeht. also ich finde, perma ist ein, ein riesen methodenkoffer. aber es ist auch eine haltung. also bin ich bereit, ähm, mehr auf die stärken zu schauen, bin mir aber auch den grenzen bewusst. ähm, oder sehe ich nur die brille mit leistungen und sehe alles nur in noten und vergesse den menschen dahinter so ein bisschen. und ich glaube, dass es die, die grundhaltung überall braucht, dass man bitte die kinder sieht. weil das sind menschen. und die menschen brauchen auch die * da muss man auch drauf schauen. nicht nur auf leistungen, weil wir sind halt nun mal keine maschinen, gott sei dank. gut. 4Ü dann hab ich schon ganz, ganz, ganz viel. jetzt schaue ich, muss mal so bisschen auf meinen spickzettel schauen, was mir auf jeden fall noch wichtig ist (LACHEND). was ich auf jeden fall noch sagen oder möch * oder wissen möchte, ähm, wenn sie jetzt einem außenstehenden erklären sollten, warum sie bei sich auf die positive bildung schauen. was sind so ihre argumente?

B: hm (nachdenkend). ähm, es ist mir wichtig, das kind als persönlichkeit zu sehen. als, als wunder, das ich begleiten darf. und ich will das kind, äh, unterstützen. ich möchte nicht den weg fix vorgeben müssen. und das geht nur, wenn ich das kind als etwas ganzes betrachten kann und dort abhole wo es steht. ich weiß, das klingt immer schon so abgedroschen. das ist dann * weil diesen satz, glaube ich, äh, der läuft durch die bildungsministerien die ganze zeit. aber es ist das einzig wichtige. ich schau mal, wo ist das kind, was braucht es jetzt gerade von mir. und dann immer wieder dranbleiben. und, äh, das schönste geschenk ist das vertrauen von einem, einem kind. und natürlich muss ich grenzen setzen. natürlich muss ich auch erziehungsarbeit leisten. und das sehe ich auch als positiven effekt, ehrlicherweise, dass ich da ein bisschen mitgestalten darf und, ähm, helfen darf, auch den blick zu erweitern. wir sind zum beispiel die regenbogenklasse aus vollster überzeugung. also für mich ist alles, wofür der regenbogen steht, äh, werte wichtig. und da dabei sein zu dürfen, äh, den kindern diesen blickwinkel da aufzuzeigen und dass die lernen, hinzuschauen, zu hinterfragen. ähm ja, also das ist glaube ich das schönste, was man einfach da erreichen kann. und, und wenn man diese basis geschaffen hat, dann kann man zu lernen anfangen, stück für stück. und dann kann man hinterher die bausteine aufbauen. und dann läuft es eigentlich. also natürlich gibt es immer mal wieder mehr arbeit, aber ich glaube, es zahlt sich so viel aus, da hinein zu investieren, weil es nachhaltig ist. und ich bekomme was zurück. das ist nicht eine einbahnstraße. weil sie kommen gerne. sie strahlen mich an. sie erzählen mir gerne, äh ja. sie, sie schreiben mir auch komplimente. sie wollen es auch anwenden. ja, ich erhalte eine postkarte zum urlaub. sie sind traurig, wenn sie gehen müssen, weil sie einfach die zeit genossen haben. und das sind schöne komplimente. und wenn sie dann sagen, ich fühle mich wohl bei der lehrerin. oder es kommt dann jemand beispielsweise mit dem informationskurs. wo gehst du hin, wenn du hilfe brauchst? und sie sagen dann, zu y.. dann denke ich mir so, ja, was gibt es schöneres? dann habe ich meinen job erledigt. einmal in (LACHT). wenn ich *

I: ja.

B: also ja, sind so meine hauptargumente. und natürlich muss man jetzt auch schauen, dass in sachen integration, wenn man sich das gehirn anschaut, dass jetzt auch gar nicht anders möglich ist. also schon allein rein wissenschaftlich gesehen kann ein kind, nicht nur ein kind, auch wir erwachsene, wir können nur wirklich lernen, wenn wir uns wohlfühlen, wenn die sogenannten schranken unten sind, ja? und, äh, wir uns in so einer gefühlswelt ausbalanciert fühlen. weil sonst kommen wir gar nicht dazu, kreativ zu sein und so aufzunehmen und etwas zu verstehen. und wenn wir halt uns selbst auch zutrauen.

I: ja.

B: genau.

I: schafft letzten endes die basis, um, um, um darauf dann aufzubauen.

B: genau.

I: okay. gibt es denn irgendwas, was sie von ihrer seite gerne noch ergänzen möchten oder sagen möchten, was irgendwie gar keinen platz oder raum hatte?

B: boah, wir haben alles so schön besprochen. ich habe alle meine sachen, die ich mir aufgeschrieben habe, habe ich abgehakt nebenbei (LACHT). ähm nein. ich finde schön, dass, dass wir wieder als, als team, auch als großes lehrerinnen-team, ähm, gemeinsam an dem strang mitziehen. ich sage jetzt ein bisschen provokant mussten, ja, weil es ein bisschen aufgesetzt war. weil es einfach uns auch alle immer näher bringt. ähm genau und mein wunsch bleibt der gleiche. ich möchte gerne, dass die bildungsdirektion, dass die leute in den, in den, äh, in den ministerien sich da auch hinsetzen und sich das einmal tatsächlich anschauen. und ja, vielleicht auch eine perma fortbildung machen. oder eine, eine von den anderen halt. können sie sich gerne aussuchen, welche (LACHT). sind überall, aussortieren. ich glaube, das wäre hilfreich. ich wundere mich sicher bei dem, ich habe ja den * ich habe jetzt den neuen lehrplan, das seminar machen müssen. und, äh, zuerst über mooc und dann eben mit diesem austausch. und da stand halt drin. und einen habe ich kennengelernt, persönlich, der dann zufällig bei uns in der schule einen, einen, ähm, nachmittag gehalten. und die hat gesagt, das waren 150 pädagogen und pädagoginnen am berg, das alles zu dokumentieren. und das sind so viele schlaue köpfe. und das ist alles * und wenn ich ihnen dann zuhöre, was sie sich gedacht haben, dann denke ich mir so, schön, ja. das ist ja schön. und dann, was soll dann daraus gemacht werden aus dieser idee, aus dem ganzen? weil zum beispiel diese 13 überfachlichen kom * ähm, kompetenzen, die sie vielleicht schon gehört haben, die wir jetzt bekommen haben im neuen lehrplan, wunderbar, wir haben eine dazu, ja? aber was wird denn in der realität draus gemerkt? das muss im kompetenzraster drinstehen. das müssen wir dort und dort dokumentieren. dass wir aber eigentlich das in das integrierte erleben. dass es gar nicht so einfach ist, das wieder herauszufiltern. und das ist zum beispiel der punkt, der halt überall hineinfällt. ähm und dann wieder einen * eine kompetenz daraus zu machen, die wir bewerten können. das ist genau der punkt, wo wir dann wieder zerrissen sind. also so viele gute ideen, so viele gute möglichkeiten. und dann wird es durch dieses angst haben, dass wir uns halt nicht beweisen können, dass wir was gemacht haben, in die endlosigkeit dokumentiert und quasi am ziel vorbeigeführt. schade. # und

I: ja. #

B: wir versuchen natürlich alle, weil wir idealisten sind und idealistinnen, äh, trotzdem in unser autonomie den kindern das * den, den kernpunkt zu vermitteln, kommen aber um das andere nicht drum rum. hilft nichts, ja? weil wenn ikm dann ausschreibt * und auch das testformat, muss ich sagen, an sich ist toll, was sie für beispiele gebracht haben. kein thema, ja? äh, wie es aufgezogen wird, wie das verpackt wird, darüber können wir sehr wohl streiten, ja? und da, da sind die punkte, wo ich finde, gehört endlich mal auch ein anderer blick und dieser mut zu sagen, ja, es gibt lehrer und

lehrerinnen, die erfüllen den auftrag nicht, aber die gibt's auch jetzt trotz den ganzen kompetenzrastern. den mut zu haben, aber ein großer teil erfüllt es. die brauchen das nicht. und wir können vertrauen haben in die pädagoginnen und die kinder, dass die da schon zu den lernzielen kommen. dass wir uns an den rahmen halten. deswegen # können

I: ja. #

B: umgesetzt haben.

I: und nicht wieder in zu enge raster reinpacken, wo man halt einfach nicht individuell und frei gestalten kann, was ja viel zielführender ist, wie dieses es müssen alle in genau den kasten irgendwie reinpassen. und wer da nicht reinpasst, der * da wird es dann schwierig. also das ist natürlich, ja.

B: weil dann bleibt es perma, ein gegenpol zu dem anderen, damit das ausgeglichen wird. aber beim anderen wäre das ineinander also quasi verschränkt. weil sie automatisch mit mehr stärken fühlen oder selbstbewusstsein die dinge, die sie halt auf ihrem * in ihrem tempo erlernen und gestalten dürfen, ohne kompetenzraster im hinterkopf die ganze zeit, ja dann automatisch so vollbringen ja? sie, sie machen das dann, ja? ohne druck.

I: ja. da können wir leider in den oberen etagen nichts ändern. wir können nur schauen, dass wir es in den schulen mit den schülern, ähm, so gestalten und so ausloten, um das beste einfach rauszuholen. und nicht das beste im sinne von die besten noten, sondern das menschliche beste. die, die bestmöglichen rahmenbedingungen auch für die kinder, aber auch für die lehrkräfte zu schaffen. weil, wie sie ja vorher schon gesagt haben, also, es geht ja selber auch an die substanz. und ähm, ich muss mich in der schule auch wohlfühlen. und nicht nur die kinder, sondern ich muss da ja auch gerne hingehen. und ich möchte weiterhin spaß daran haben, mit den kindern neue themen zu erarbeiten und zu entdecken. und mir nicht denken, oh gott, ich muss schon wieder dahin, und ich muss schon wieder.

B: ja. wobei das, das gefühl habe ich nicht. ich, ich bin nur manchmal die krise, wenn ich einen neuen zettel zum ausfüllen bekomme. und ich so, was ist jetzt das (LACHT)? wo mache ich wieder einen haken? also da halt, ja. das ist für mich belastend. und die arbeit mit den kindern ist das, was ich, was ich wirklich gerne mache. also nicht in der klasse steht, sondern das in ruhe lässt (LACHT). die glücklichste von allen.

I: sehr schön. ja, auf das geht ja, kommt ja drauf an. nur die anderen sachen brauchen leider gottes meistens sehr viel platz und nerven.

B: genau.

I: okay. dann würde ich sie zum abschluss so ganz im sinne von perma, äh, noch bitten, dass sie mir einfach irgendwie so zwei oder drei, ähm, gedanken nennen, für die sie dankbar sind. oder situationen oder momente.

B: hm (zustimmend). aber auch * also ich nehme an, nur schulisch, oder? jetzt meinen sie im # schulischen bereich.

I: ja, also irgendwo # in diesem * im bereich, was jetzt mit der positiven bildung zu tun hat. das ist ja nicht, nicht immer nur isoliert auf schule. manchmal wirkt es ja auch noch woanders hin.

B: ja. also ich bin * ich bin dankbar für meine autonomie, die mir privat bleibt, nach wie vor in dem ganzen, äh, dokumentationsirrsinn. die mir die möglichkeit gibt, eine beziehung zu den kindern aufzubauen. das ist einmal das, wofür ich am meisten dankbar bin. äh, dass es mir auch gelingt. und ich weiß, dass die kinder, äh ja, mich mögen, vertrauen haben, gerne kommen und dass wir dieses band haben. ich bin dankbar für das kollegium drum herum, das mit mir gemeinsam am strang zieht. und wo wir gemeinsam uns stärken und, äh, füreinander da sind. uns, äh, unterstützen und, ähm, im sinne der resilienzforschung auch dann da sind, wenn einmal die kraft ausgeht. genau, also ich bin aber auch dankbar für die eltern, muss ich auch dazu sagen. weil man die ja gerne, gerne vergisst. manchmal sind die eltern auch so ein bisschen das feindbild, äh, weil man sich hier fast ein bisschen fürchtet manchmal davor, wenn wieder eine nachricht kommt. Was kann man wollen, dass es stets wertschätzend gegangen. das heißt, dass sie mitgezogen sind, dass sie das auch mit mir gemeinsam den weg gegangen sind und sich das * auf das eingelassen haben und wir dann auch daraus eine stärke ziehen konnten. und ich hatte gestern erst elternabend von den neuen kindern, habe das auch vorgestellt wieder und habe gemerkt, dass die eltern das wohlwollend aufnehmen und dabei sein wollen. und dass wieder so dieses dreieck dann wunderbar wieder fahren können. also somit ein schönes rundherum, ein schönes wirken.

I: sehr gut. dann ihnen vielen, vielen dank. sie bekommen von mir oder eben vom forschungs-team dann auf jeden fall so ein, so ein ergebnis, was aus der ganzen studie eben auch so als erkenntnis rausgekommen ist.

B: hm (zustimmend).

I: wenn sie im anschluss an unser interview nochmal fragen haben oder so an mich oder so, dann gerne an mich wenden. ansonsten eben an das forschungs-team. genau, das ist so das ganz wichtige. gibt's denn jetzt schon irgendwelche fragen oder irgendwas, was sie noch wissen wollen?

B: * nein (LACHT).

I: okay, dann vielen dank. und jetzt hoffentlich noch ein bisschen, ähm, schöne zeit draußen. ein bisschen sonne tanken.

B: danke schön. auch alles liebe.

I: danke. und ihnen auch viel erfolg bei der masterarbeit.

B: ja, danke. ebenfalls (LACHT).

I: danke, tschüss!

Anhang G: Kodierleitfaden

Kategorie	Definition	Ankerbeispiele	Kodierre-geln
**OK 1: Fortbildungs-konzept PERMA.teach**	PERMA.teach ist ein Fortbil-dungskonzept, welches die Positive Psychologie auf den Kontext Schule überträgt.		
**UK 1.1 Zugang zur Fortbildung**	Wie sind die Lehrkräfte über die Fortbildung informiert worden und wer hat die Lehr-kräfte angemeldet.	Oder angestupst, wurde das ganze von der äh, schulqualitätsmanagerin damaligen, j. s. äh, und die hat so ein bildungsnetzwerk äh, begonnen. und da waren wir halt einige schulen, aller er-möglichen größen aus dem, aus unserem bezirk, politischen oder, oder schulbezirk. und ähm, und da hatten wir dann zum index inklusion einein-halb, zwei jahre gearbeitet, mit input äh, aus süd-tirol. weil südtirol ja da eine vorbildliche region ist. und äh, und aus dem heraus ist über die päda-gogische fe* hochschule feldkirch, mit der uli lichtinger, dann ähm, der kontakt äh, erwachsen. und dann hat sich das eben in richtung perma.teach. durch das flori* flourishing ist es äh, dann ähm, weitergegangen, äh, auf die äh, ingrid teufel und eva, hm, jambor (Interview 4, Zeile 78-85)	
**UK 1.2 Teilnahme**	Geschlossene oder teilweise Teilnahme des Kollegiums an der Fortbildung sowie deren Veranstaltungen.	ursprünglich haben alle gemacht gesta* ähm, mitgemacht gest* weil wir uns das einfach als ge-meinsames ziel für das umwelt zei* für die um-weltzeichen-prüfung ge* äh, gesetzt haben (Interview 4, Zeile 118-119)	
**UK 1.3 Teilnahme-gründe**	Darstellung der Gründe für die Teilnahme an der Fortbil-dung.	ich denke, in der schule, ist man ab und zu ge-fährdet, dass man so in einen jammer-kreislauf nach unten, ähm, versinkt und gar nicht mehr wahrnimmt, was eigentlich alles passiert und was man eigentlich alles macht und bewegt. und äh, und dieses, jeden tag ein bisschen reflektieren und sich jeden tag einfach ein kleines neues ziel setzen, ähm, so habe ich das halt empfunden, äh, das ha* * habe ich gefunden, dass das guttut und dass man da ein bisschen dagegen ankämpfen kann, dass man nicht immer nur sich gegenseitig vorjammert: „und der hat, und der war lästig und das ist schon wieder vorgefallen“ und so weiter (Interview 4, Zeile 99-105)	
**UK 1.4 Erster Eindruck**	Nach der Erstinformation über die Inhalte der Fortbildung, entsteht ein erster Eindruck darüber, ob die Fortbildung zu der Person oder der Schule passt.	dieses säulenprogramm kennen wir ja eben in an-deren varianten halt auch, sind halt jetzt finger. das hat ein bisschen entspannung reingebracht. (Interview 5, Zeile 71-72)	

**UK 1.5 Erwartungen der Lehrkräfte**	Die Lehrkräfte haben eine Erwartungshaltung an die Inhalte der Fortbildung sowie deren Umsetzungsmöglichkeiten an der jeweiligen Schule.	für meine unruhigen jungen, die gerade im sozialen bereich *, sie sind zwar recht, ja, sie sind auf einen, auf der einen seite recht sozial, sie helfen sich gegenseitig, sie sind freundlich. aber sie sind auch recht rüpelhaft, oft und einfach unvorsichtig im umgang miteinander und unbedacht miteinander.<br>(Interview 1, Zeile 89-92)	
**OK 2: Kenntnisse der Positive Bildung**	Vor der Teilnahme an der Fortbildung besteht ein unterschiedlicher Wissensstand über die Positive Bildung.		
**UK 2.1 Vorwissen**	Bereits vorhandenes Wissen über die Positive Bildung oder Assoziationen der Positiven Bildung.	ich glaube, positive bildung ist alles was man oft mal außer, also was im schulischen bereich eh ist, aber was man zusätzlich oftmal macht. also, wenn man mit klassen zum beispiel weihnachten oder theater einstudiert und das vor die eltern aufführt, oder wenn man mit den kindern gewisse sportliche aktivitäten macht, ob man jetzt eislaufen, schwimmen, wenn man solche sachen macht, das prägt einfach eine klasse. und das fügt das ganze sozialgefüge verbessert es einfach auch. oder wenn man eine lesenacht macht.<br>(Interview 3, Zeile 148-153)<br><br>wenn man eben, äh ja, auf der sozialen ebene die vertrauensbasis nicht geschaffen hat, dann hat das sowieso gar keinen sinn überhaupt anzufangen, einen buchstaben zu lernen.<br>(Interview 5, Zeile 40-42)	
**UK 2.2 Erkenntnisse der bisherigen Arbeit**	Die bisherige Arbeitsweise und verwendeten Methoden werden mit der Positiven Bildung in Verbindung gesetzt und daraus resultiert eine Einordnung<br><br>- Verknüpfung der Positiven Bildung mit anderen Konzepten	früher war das sqa, und da war unser thema tugenden und umgang *, wertschätzender Umgang miteinander, und das ist jetzt praktisch für uns die fortführung, weil das geht ja genau in dieselbe richtung. und, so haben wir jetzt für das, was wir machen müssen, von schulansicht her, einfach das fortgeführt. es hat einen anderen namen, es nennt sich anders, a *, aber vom inhalt her hat sich nicht sehr viel verändert. es ist nur noch genauer und noch mehr unterstützend durch das buch, das wir da bekommen jetzt für die kinder, durch die *, durch den fragebogen, durch die perma.post, wo immer sehr viele sachen drinnen waren. durch den knietzsche, der *, der bei den kindern voll eingeschlagen hat, schon. und auch *, auch bei den lehrpersonen, weil dieser macht es so toll und bringt es ist auch wertiger * wertiger ist das ganze geworden.<br>(Interview 2, Zeile 63-71)	
**OK 3: Feedback zum Fortbildungskonzept PERMA.teach**	Feedback über die bereits absolvierten Bestandteile der Fortbildung sowie Anregungen für zukünftige Veranstaltungen.		

**UK 3.1 Fortbildungs-aufbau**	Grundsätzlicher Aufbau des Fortbildungskonzeptes mit den unterschiedlichen Veranstaltungen, welche online und in Präsenz stattfanden.	wir sind in einer totalen randlage. ich habe sonst immer einen ziemlichen wegezeitverlust. also, das ist für mich äh, ganz gut, äh, von der äußer* also von der organisationsform her (Interview 4, Zeile 453-455)<br><br>aber auf der anderen seite wieder, als ich das erzählt habe, auch bei kolleg*innen, dass wir da jetzt die *, das online haben von allen volksschulen, von *, von ganz österreich, die da mitmachen, vom burgenland bis vorarlberg, das wäre sonst nie möglich, und das ist *, hat wieder in einen anderen wert (Interview 2, Zeile 401-404)	
**UK 3.2 Fortbildungsinhalte**	Umfang sowie Auswahl der vermittelten Fortbildungsinhalte.	dieses irgendwann einmal geht es zu weit, man kann nicht alles durch eine rosarote brille sehen. und wenn das kind mit schimpfwörtern um sich wirft, dann kann ich nicht sagen, es hat eine stärke, es ist verbal kreativ." und da finde ich auch, da gibt es wirklich irgendwo einen punkt, wo wir lehrer auch mal sagen müssen, das ist eine grenze, die erreicht ist und da will ich auch keine stärken mehr finden. da will ich unterstützung von außen, weil ich überfordert bin und weil die situation untragbar ist in der klasse. und da will ich keine stärken mehr suchen. also, ich finde, dieses stärken suchen, kann man auch übertreiben. also, es muss einfach grenzen geben. (Interview 1, Zeile 214-220)	
**UK 3.3 Schulungsbausteine und Stärken.Café**	Das Fortbildungskonzept besteht aus zwei Schulungsbausteinen sowie Stärken.Cafés, in denen den Lehrkräften die PERMA.Faktoren vermittelt und Ideen ausgetauscht werden.	„letztendlich, irgendwas ist immer dabei, was man, was man gut nutzen kann, was einen selber anspricht“ (Interview 4, Zeile 417-421)	
**OK 4 Feedback zu den Fortbildungsmaterialien**	Im Rahmen der Fortbildung stehen den Lehrkräften verschiedene Materialien zur Verfügung, sodass ein Repertoire unterschiedlicher Materialien vorhanden ist, aus denen sich die Lehrkräfte individuell die Materialien herausnehmen und mit den Schüler:innen bearbeiten können.		
**UK 4.1 Stärkenbuch**	Die Stärkenbücher stehen den Schüler:innen und Lehrkräften in unterschiedlicher Ausführung zur Verfügung und ist ein Unterrichtsmaterial, welches die Positive Bildung umfasst.	also sicherlich war* perma eine fixe stunde auch. Also dieses perma, tatsächlich arbeiten am buch, war eine fixe unterrichtsstunde. die haben wir uns einfach rausgenommen vom sachunterricht. also das war jeden montag in der zweiten stunde fix. (Interview 5, Zeile 303-305)	
