# Supplementary material for: PERMA.teach: a study on the effectiveness of a standardized positive education training program in Austria
Source: Front Psychol. 2025 Apr 8;16:1516572. doi: 10.3389/fpsyg.2025.1516572 (PMC12053473; doi:10.3389/fpsyg.2025.1516572)
Supplement: Supplementary file 2 [file Data_Sheet_1.ZIP › Material PERMA.teach/Material LPs/Interviews LPs Teil 1.pdf]

Anhang B: Interview 1

09.05.023 14 Uhr

I: aufzeichnung. genau, sie müssten auf der anderen seite zustimmen, dass das alles seine richtigkeit hat. * okay, ich würde es zusätzlich am handy noch laufen lassen, falls aus irgendwelchen gründen eine technik spinnt, dass eben die andere auch noch aufnehmen kann. ist das für sie in ordnung?

B: ja.

I: okay, gut. dann bin ich erstmal ein bisschen neugierig, wer sie so als person sind. vielleicht wollen sie mir einfach so zwei, drei sätze von der frau j als lehrkraft erzählen.

B: als lehrkraft, okay. also, ich unterrichte in einer ganz kleinen schule, wir sind nur zweiklassig. wir haben teilungsstunden, naja, unterschiedlich viele, je nachdem wie viele kinder. ich habe heuer erste, zweite klasse. In der ersten vier kinder, in der zweiten acht. die erste klasse, das sind vier total geschickte kinder, unkompliziert und einfach zu händeln. und die zweite klasse ist eigentlich das gegenteil davon. in der zweiten klasse ist dann generell *, es ist nur ein mädchen und sieben buben und die sind alle total aufgeweckt und quirlig. * es sind drei sehr schwache kinder drinnen und * ja, sie sind sehr, sehr unruhig. und vor allem haben wir einen drinnen, der uns wirklich total kopfzerbrechen bereitet. also, das ist ein ganz schwieriges kind, der ist vom sozialverhalten her wirklich ganz extrem. wir sind jetzt auch gerade dabei, dass wir alle möglichen, ja, alles in die wege leiten, dass wir einfach unterstützung bekommen. es ist jetzt so, dass wir die woche erfahren haben, wir bekommen für nächstes jahr zumindest vier sbf stunden für ihn. wobei bei uns das große problem ist *, also, wir hoffen eigentlich auf eine schulassistenz, aber es ist einfach dieser personalmangel das große problem. was hinzukommt, ist, dass für die teilungsstunden, die wir übrig haben, meine chefin und kollegin und ich haben wir heuer eine quereinsteigerin bekommen, die insgesamt, glaube ich, zwölf unterrichtsstunden hat. bei mir, * es gibt insgesamt zwei, * drei, vier, ich habe fünf teilungsstunden. eins, zwei, drei, vier, ich glaube, ich habe fünf teilungsstunden. die restliche zeit habe ich die kinder immer beisammen. und die kollegin, also die kollegin, die die teilungsstunden jetzt bei uns hat, ist seit, ich glaube, sechs wochen im krankenstand. das heißt, wir haben eigentlich überhaupt keine teilungsstunden mehr, außer die religionsstunden. wo ich, wenn meine kinder religion haben, bei der anderen klasse bin und umgekehrt, meine kollegin oder halt chefin bei mir ist, wenn ihre kinder religion haben. und das ist einfach total schwierig. es ist, ja, also einfach nicht mehr lustig. das ist die schulische situation, aber gott sei dank geht es privat bei mir einfach so gut, dass ich wenigstens zu hause dann kraft tanken kann und wenigstens zu hause keine sorgen habe. ich denke mir immer, wenn jemand daheim dann vielleicht auch noch so kleine kinder hat, die eventuell auch probleme in der schule haben oder so, dann, ja, keine ahnung, wie man das dann schafft. also, ich würde das nicht schaffen, wenn ich zu hause auch noch irgendwelche fabels hätte, muss ich echt

sagen. ich habe drei kinder, die schon langsam jetzt erwachsen werden, die sehr unkompliziert sind, gott sei dank. aber wenn sie nicht so wären, keine Ahnung. also, es war noch nie so, dass ich eigentlich in meiner lehrersituation wirklich überlegt habe, welche alternativen es gäbe für mich. das war wirklich noch nie. und ich muss auch sagen, ich habe das jetzt auch im, im kollegium, im bekanntenkreis schon mitbekommen und auch tatsächlich von einer lehrerin in einer nachbarschule, die 15 jahre jetzt im dienst ist und kündigt und sagt, sie schafft es nicht mehr. mir war meiner, mit meiner chefin, wir sprechen zurzeit viel darüber. also dieser personalmangel, der einfach so extrem ist, der wird bestimmt nicht nur jetzt aus der, also von der seite her das problem sein, dass es so wenig lehrer gibt, sondern auch, dass die lehrer, die es gibt, entweder in das burnout gehen oder krank werden oder kündigen. es werden viele ganz einfach mit der situation nicht mehr fertig, wie es jetzt ist. und bei uns ist jetzt das problem, dass die quereinsteigerin, die bei uns die stunden genommen hat, jetzt eben so lange krank ist und uns diese woche mitgeteilt hat, nächstes jahr wird sie nicht mehr bei uns unterrichten. sie wird das wieder lassen, weil es ihr auch zu viel ist. quereinsteiger, glaube ich, stellen sich auch alle ganz anders vor als es tatsächlich ist. und sie macht das nächstes jahr auch nicht mehr. das heißt, momentan wissen wir gar nicht, ob wir für nächses jahr jemanden haben für teilungsstunden und die sbf stunden. also, ich bin gespannt, was auf uns zukommt.

I: ja, das klingt tatsächlich nach einer spannenden und herausfordernden zeit richtung, richtung zukunft, richtung nächstes schuljahr. umso schöner ist es ja, dass sie für sich einfach noch diesen, den punkt haben, dass sie sagen, ich arbeite wahnsinnig gerne und ich habe aber auch meine anderen kraftquellen. die wir ja alle einfach brauchen und die gerade in der bildung ganz viel auch die, die energie eben reinbringen müssen. genau. was war denn damals so ihre motivation, so diese hauptmotivation, lehrkraft zu werden, oder, ja?

B: na, weil es einfach ein wunderschöner beruf ist und das denke ich nach wie vor. also das lehrersein an sich, die arbeit mit den kindern, die schönen erlebnisse, die wir haben. also, das, ich kann mir gar nicht vorstellen, dass ein anderer beruf mich so erfüllen könnte. aber ja, das rundherum ist es einfach, das *. sogar mein mann, der nicht lehrer ist, hat heute in der früh haben wir darüber diskutiert und hat gesagt: "ja", von ihm selber ist es gekommen: "ja, es ist ja schade, dass du deinen beruf eigentlich so an sich gar nicht mer genießen kannst, wegen dem ganzen rundherum." und das, also das war es eigentlich, warum ich lehrerin geworden bin. die arbeit mit den kindern, einfach auch zu sehen, wie sie sich entwickeln, wie man ihnen dabei hilft, wie sie persönlichkeiten werden. wie sie entdecken, welche stärken sie haben, was in ihnen steckt. wie man erlebt, welche erfolgserlebnisse sie haben. also das alles ist einfach für mich total bereichernd und das war der grund, warum ich lehrerin geworden bin.

I: sehr schön. da spricht so eine vollblut lehrerin aus ihnen oder lehrkraft aus ihnen.

B: auf alle fälle.

I: das ist *, aber ich finde, das ist in unserem beruf oder im pädagogischen bereich so wichtig. also in dem moment, wenn man begeistert ist, dann fällt es einem leichter. ich merke das bei meinen studenten oder meinen studierenden, die jetzt dann jetzt gerade im letzten ausbildungsjahr sind und ab september eine kindergartengruppe zum beispiel übernehmen. wenn die mich schon begrüßen und das leuchten in den augen haben, wenn sie mit den kindern arbeiten können. das ist immer so das, wo man merkt, okay, da steckt eine berufung dahinter und das ist mal ganz was anderes, weil da viel mehr engagement und herzblut einfach auch in so eine arbeit gesteckt wird. was ja letzten endes dann im schulischen sinne genauso ist. dass, wenn ich herzblut reinstecke und einfach wahnsinnig gerne auch mit den kindern arbeite, dann hat das einfach nochmal eine ganz andere tiefe auch, das stimmt.

B: hmhm (zustimmend).

I: gut. wie sind sie denn eigentlich zu dem,zu der, zu dem fortbildungskonzept gekommen oder zu der fortbildung? oder was war ihre motivation, da teilzunehmen?

B: also, meine chefin und kollegin ist immer sehr motiviert, etwas neues aufzugreifen, überall mitzumachen, das ist mir manchmal auch zu viel. und da war es wieder sie. also, sie hat davon gehört und hat gesagt: "okay, da könnten wir mitmachen, das hört sich interessant an." und es war aber für mich auch gleich so, dass ich gesagt habe: "okay, also, das finde ich auch interessant. kann ich mir vorstellen, dass das gut passt." und ja, also ich bin dann schon gleich neugierig worden und habe mir gedacht: okay, das, glaube ich, macht sinn und bringt sicher was für den unterricht. oder vor allem für meine unruhigen jungen, die gerade im sozialen bereich *, sie sind zwar recht, ja, sie sind auf einen, auf der einen seite recht sozial, sie helfen sich gegenseitig, sie sind freundlich. aber sie sind auch recht rüpelhaft, oft und einfach unvorsichtig im umgang miteinander und unbedacht miteinander. und da habe ich mir gedacht, wäre so was, glaube ich, auch nicht schlecht.

I: jetzt muss ich nochmal nachfragen, welche altersklassen oder welche, welche jahr, ja, alter haben sie jetzt nochmal?

B: sechs bis acht.

I: sechs bis acht. okay. * ja. * das heißt, was hat sie besonders angesprochen an diesem fortbildungskonzept? was hat sie sofort begeistert, dass sie sagen, ja, das probieren wir aus?

B: die kinder stärken und dass sie durch ihre, durch das bewusstwerden der eigenen stärken, auch die stärken der anderen kinder sehen. und wenn jemand gestärkt ist in sich, in seiner persönlichkeit, hat er es ja auch nicht so not, dass er andere klein macht, wenn er, wenn er sich selber nicht klein fühlt. das war für mich auch so ein hintergedanke. also wenn, wenn die kinder gestärkt sind, sind sie glaube ich, auch ja, verständnisvoller, großzügiger und sozialer.

I: ja, das stimmt. haben sie schon über die grundsätzlich, über die positive bildung oder psychologie vorher schon so, so vorwissen oder so eine vorahnung gehabt?

B: ja, ja, doch.

I: okay. * und wenn man jetzt sagen würde, der unterschied zwischen regelunterricht und jetzt diesem unterricht mit dem perma.teach, was würden sie jetzt sagen, ist so der größte unterschied, der jetzt so auffällt? für sie jetzt einfach erstmal.

B: es ist für uns nicht so ein großer unterschied. wir haben viele dinge, die dort in dem perma vorkommen, ganz viele dinge vorher sowieso schon gemacht. also wirklich ganz viele dinge waren ohne, ohne dass man es bewusst macht, ohnehin im unterricht eingebunden. insofern hat sich eigentlich nur verändert, dass ich es bewusster mache und diese dinge, die ich im Unterricht mache, einfach * mit perma verbinde.

I: können sie ein beispiel bringen, was sie einfach auch vor der fortbildung schon so in den alltag integriert haben?

B: ein beispiel? ja, zum beispiel so glücksmomente aus der woche, so einen wochenrückblick machen. und auch mit dem positiven abschließen, dass man am ende der stunde oder am ende der woche einfach das positive hervorhebt. gerade wenn man vielleicht auch mal nicht so eine gute woche gehabt hat, finde ich, dass die kinder einfach mit, mit einem anderen gesicht, mit einer anderen haltung oder mit einer anderen motivation auch aus der klasse oder aus der schule gehen, wenn man über das positive in der woche redet. * würde ich zum beispiel. und dann auch, * ja, also, das mit den stärken, solche dinge haben wir schon auch vorher gemacht, aber jetzt halt einfach bewusster. oder ein bisschen gezielter.

I: #verständlich.#

B: wobei ich trotzdem noch mehr erhofft habe, ich hoffe, ich greife jetzt nicht vor. weil ich eben gewisse dinge bewusster *. also zum beispiel so im sachunterricht, die eigenen stärken oder die stärken der anderen kinder, das macht man ja immer wieder. und dieses jahr habe ich es einfach bewusster gemacht. auch mit sprechblasen, habe mir da was gebastelt, am computer zum ausschneiden. welche stärken gibt es und auch natürlich wortschatzerweiterung. das ist eh recht aufwendig, finde ich, bis man überhaupt soweit ist, dass man stärken bestimmen oder, oder finden kann, weil der wortschatz nicht vorhanden ist. und das war dann irgendwie schon sehr ernüchternd, weil ich doch sehr viel zeit investiert habe. und dann waren wir endlich soweit, dass die kinder stärken suchen konnten. dann haben sie erstens einmal doch wieder ganz viele herausgesucht und nicht nur ein paar. dann kann man auch nicht durchgehen und sagen, du, pass mal auf, das ist eigentlich keine stärke von dir. (LACHEN) weil wir haben es eh vorher genau besprochen, was das bedeutet. und ich habe dann auch eigentlich mir erhofft, dass, dass die mehr freude haben mit ihren

stärken und stolz sind darauf und das vielleicht auch ab und zu betrachten. aber es war eigentlich fertig gemacht, heft zu, vergessen.

I: okay.

B: und man muss dann halt auch immer wieder an der gleichen sache arbeiten und das immer wieder heraus holen und hervor holen, sonst ist es einfach auch wieder vergessen. und das finde ich ein bisschen schade, dass es so, so doch zeitintensiv ist und ja, viel trotzdem einfach nicht so fruchtet, wie man sich erhofft.

I: haben sie eine vermutung, woran das liegen könnte? dass es einfach so dieses arbeitsauftrag erledigt, heft zu?

B: * naja, weil, weil man es einfach immer wieder machen müsste und immer wiederholen und, und dran bleiben, nur das, die zeit hat man einfach nicht. dass man, dass ich jetzt, wenn ich im sachunterricht oder auch in deutsch im sprechen, wenn ich das jetzt mal als schwerpunkt mache, das passt. aber ich müsste halt jede woche wiederholen und weitermachen und dranbleiben und irgendwann schafft man es nicht. da denkt man sich, nächste woche mach ich es und dann ist aber wieder ein anderes thema. und man muss da schauen, dass man fertig wird und da kriegt man einen stress und da hat man einen zeitdruck, und dann ja, verläuft es sich wieder. es ist *, ja, und wenn man nicht dranbleibt, vergessen es die kinder auch.

I: ja, das stimmt allerdings. gerade bei neuen methoden und neuen herangehensweisen braucht es am anfang einfach doch tatsächlich vieles an, an zeit, dass es quasi schon, schon so ein selbstläufer, ist, dass die kinder das von sich aus schon auch mit aufgreifen. und das ist am anfang tatsächlich aufwendig und vor allem, wenn sie sagen, dass es gerade personell jetzt auch nicht super eingedeckt ist, dann haben halt gewisse andere themen, also letzten endes der unterrichtsstoff, auch vorrang, nehme ich an.

B: hmhm. (zustimmend)

I: hätten sie sich von dem, von der unterstützung vom fortbildungskonzept mehr gewünscht? wenn sie sagen, sie haben zwar sachen übernommen, aber die wrwartung wäre nochmal eine andere gewesen?

B: nein, also, ich finde, es waren viele inputs, viele tolle ideen, es war ein super austausch. nein, von dem her, ich finde, es war sehr verständlich alles, sehr gut aufgebaut. nein, das ist hat mir gut gefallen.

I: gibt es was, was ihnen von dem, von dem fortbildungskonzept, also sie haben ja zwei module gehabt und das stärken.café und auch die perma.post zum beispiel, was, wo sie sagen, das hat ihnen besonders viel geholfen? oder da konnten sie sachen auch anwenden oder prägt noch?

B: * nein, man nimmt sich eigentlich aus allem immer ein bisschen was mit. nein, nicht jetzt direkt ein konkretes beispiel.

I: okay. und die materialien, also, die, die hefte so kinderstärken, haben sie das auch mit in die arbeit mit reingenommen?

B: ja, vereinzelt. also ich nehme nur einzelne seiten. ich habe auch bewusst das heft nicht, weil das für mich einfach wirklich für mich persönlich wieder zusätzlicher druck ist. wenn ich irgendwelche unterlagen habe, will ich die auch verwenden und dann stresst mir das, wenn ich das gefühl habe, ich komme da nicht durch, oder es bleibt so viel unausgefüllt in dem heft. also, das ist mir einfach lieber und das gefällt mir auch ganz gut an dem konzept, dass ich die einzelnen seiten aus dem internet holen kann und dann macht man das halt und bringt es ganz unkompliziert einfach in seinen unterricht ein. und so vorgefertigte hefte oder, oder bücher geben einem das gefühl, man muss das durchbringen, man muss alles machen.

I: das stimmt. und so soll es ja letzten endes sein, dass man sich das raussucht, was einem selbst eben letzten endes entspricht. also da wären wir wieder ganz beim beim perma, letzten endes, dass man sich selber drinnen findet und auch so die eigenen stärken, bei sich entdeckt und auch die auch nutzen kann.

B: #genau.#

I: deswegen wäre auch so ein bisschen das thema. haben sie bei sich irgendwelche bereiche erkannt, wo sie sagen, die liegen ihnen eher? bei diesem ganzen repertoire, ist ja der unterschied zwischen eher kognitive sachen oder eher, eher praktischeren sachen? haben sie da irgendwie so eine tendenz, wo sie sagen jetzt, das nutzt sich häufiger?

B: * nein.

I: * völlig in ordnung. das war jetzt einfach nur so eine überlegung, weil es von mir tatsächlich so eine hypothese war, dass man ja selber auch so ein bisschen präferenzen letzten endes hat. dass man zum beispiel, sie haben ganz am anfang gesagt, dass ihnen so diese stärken stärken ganz wichtig ist, dann könnte es ja sein, dass so, dass letzten endes so ein bisschen so ein fokus ist, dass man eher so auf die stärken schaut. deswegen war so diese frage, ob sie bei sich auch eine neigung erkannt haben, weil das von mir so eine hypothese war.

B: naja, vielleicht, * doch, es könnte sein, dass ich durch dieses perma mehr über meine eigenen stärken nachgedacht habe. bewusster, ja, doch, also meine, ja, vielleicht schon. wobei gerade das, mit den stärken, es stimmt schon. es ist für mich ein thema, aber was auch wieder, also beim letzten café, muss ich ehrlich sagen, war sehr ernüchternd für mich und ich glaube auch für andere, die dabei waren. weil ich finde, da ist es so, also das war dann schon so, das war einfach zu viel. also das ist darum gegangen, man soll sich jetzt überlegen, ein kind in der klasse, dass *, an die

schwächen eines kindes denken und dann überlegen, was kann ich aus diesen schwächen für stärken, also in diesen schwächen für stärken finden. und dann hat sich eine gemeldet und hat gesagt, naja, sie hat ein kind, das ist unmöglich und ich glaube, das ist so in der art, wie das kind bei mir in der klasse ist. und hat ein bisschen aufgezählt, also wirklich sozialverhalten total schwierig. ist zu den kindern gemein, also beschimpft, mit schimpfwörtern, mit hässlichen und ist auch nicht nur verbal, sondern auch körperlich, dass es die kinder angreift. ist respektlos, befolgt keine anweisungen und so ungefähr ist mein kind in der klasse auch. und dann hat eben die vortragende, ich weiß nicht mehr, wie sie hieß: "ja, welche stärken kann man da finden?" und einige sehr bemühte haben dann auch stärken aufgeschrieben, wie zum beispiel mit den schimpfwörter, ja, ist verbal geschickt oder solche sachen. und dann hat sich aber schon irgendeine gemeldet, hat gesagt: " wisst ihr was, leute? dieses irgendwann einmal geht es zu weit, man kann nicht alles durch eine rosarote brille sehen. und wenn das kind mit schimpfwörtern um sich wirft, dann kann ich nicht sagen, es hat eine stärke, es ist verbal kreativ." und da finde ich auch, da gibt es wirklich irgendwo einen punkt, wo wir lehrer auch mal sagen müssen, das ist eine grenze, die erreicht ist und da will ich auch keine stärken mehr finden. da will ich unterstützung von außen, weil ich überfordert bin und weil die situation untragbar ist in der klasse. und da will ich keine stärken mehr suchen. also, ich finde, dieses stärken suchen, kann man auch übertreiben. also, es muss einfach grenzen geben. *

I: ja.

B: und das ist dadurch gut herausgekommen, dass es auch nicht immer passt.

I: * ja, tatsächlich. also, man muss ja immer schauen, was habe ich für eine situation und letzten endes ist ja perma auch nur eine erweiterung des methodenkoffers. also, so würde ich es jetzt einfach sehen, dass man schon gezielt in diese, in die positive richtung nochmal den blick weitet und auch nochmal die methodenkoffer füllt. aber es es gibt nicht jede situation her, dass man daraus einfach schöpfen kann. nur, der blickwinkel sollte halt mehr richtung positives gehen. aber da (HUSTEN), entschuldigung, da gehe ich ganz mit ihnen, dass es mit sicherheit auch einfach grenzen gibt, in denen das ganz gezielt auch nicht hergenommen werden kann, genau. bei den fachlichen impulsen, die sie da mitbekommen haben, wie fanden sie die so? die so in den modulen?

B: gut, also wirklich toll.

I: sehr gut. gab es da irgendeinen schwerpunkt, der ihnen besonders lag, wo sie sagen, da habe ich viel mitgenommen? #oder, der ihnen jetzt *.#

B: auch der ideenaustausch, also, ja, der ideenaustausch war eigentlich recht interessant.

I: also auch mit den kolleginnen letzten endes?

B: erfahrungsaustausch, aber jetzt, nicht jetzt auf erfahrungen mit kindern oder, oder wie die umsetzung gelungen ist, sondern auch methoden, welche methoden man verwendet hat und doch, wie

die umsetzung auch gelungen ist. doch, das war, glaube ich, für einige recht interessant, ja, also das war gut.

I: kann ich mir kann ich mir auch gut vorstellen, vor allem, wenn man dann wirklich die möglichkeit hat, situationsorientiert einfach nochmal darüber sich auszutauschen. weil doch jeder auch die, die ideen, die man ja mitbekommt, auch nochmal anders ausnutzt und ja dann auch nochmal neue ideen entstehen. das ist ja das schöne, #genau# wenn man sich austauscht, dass man was hört. das muss ich nicht eins zu eins übernehmen, sondern letzten endes habe ich auch dann die möglichkeit, mir zu überlegen, okay und einen aspekt davon, den kann ich mir wunderbar hernehmen, weil der ist in meiner klasse in dem setting genau richtig. aber das alles drum herum vielleicht eben nicht. und ich glaube, je mehr man auch austauschen kann und auch so ein bisschen hören kann, wie machen es denn eigentlich die kollegen und kolleginnen? wie gehen die eigentlich mit solchen situationen um? ist vielleicht auch nochmal ganz was anderes, wie wenn man es nur vorgetragen bekommt oder eben nur schriftlich, was ja eine anregung sein kann. gerade so mit der perma.post ist ja auch nochmal so eine schöne, so ein schöner denkanstoß. aber wenn man dann nochmal hören kann, wie es die kollegen oder kolleginnen machen, hilft auch nochmal. waren sie bei sich die, oder wie war denn der austausch mit ihren kolleginnen oder mit ihrer chefin? haben sie da auch sich austauschen können über perma?

B: ja. also, wir haben die, die cafés und also alle veranstaltungen eigentlich gemeinsam gemacht und haben uns auch nachher immer ausgetauscht. aber ja, wir sind sowieso immer total im gespräch, also egal, generell, ja.

I: sehr schön. da höre ich jetzt so auch raus, dass sie dann letzten endes auch die unterstützung von ihrer chefin bekommen haben. gerade wenn sie sagen, dass das ihre Idee war, dass sie das gehört hat und gesagt hat: "ach, das wäre was."

B: ja.

I: das mal hernehmen, dass man auch ausprobieren könnte.

B: nein, wir sind auch beide begeistert. also es ist schon eine tolle sache auf alle fälle. und wir versuchen beide verschiedene dinge in der umsetzung, erzählen uns dann, wie es, wie es uns gegangen ist, wie es uns gefallen hat, wie es bei den kindern ankam. also, * das passt gut, der austausch.

I: sehr schön.

B: und dann tun wir uns ja auch gegenseitig motivieren, was neues auszuprobieren oder auch ja, verschiedene unterlagen oder irgendwelche dashcards oder dinge, die man dann selber bastelt für, für *, um das umzusetzen. das tauschen wir dann natürlich auch zusammen.

I: ja, sehr schön. das ist tatsächlich eigentlich ideal, wenn man nicht alleine auf so eine fortbildung geht, sondern immer mit jemanden, mit dem man zusammenarbeitet. weil man einfach die eigenen kinder oder die eigene klasse nochmal ganz anders kennt und dann wirklich auch da nochmal schauen kann, okay, was haben wir jetzt alles an input und wie können wir das jetzt für uns einfach anwenden. und wenn man da einfach wirklich direkt auch einen austauschpartner hat und nicht auf einem stärken.café oder so warten muss. das stimmt schon, dass das bereichert, kann ich mir vorstellen, dass das schon nochmal bereichert. * muss nur nebenbei ein bisschen auf meine zettel spricken, (LACHEN) was ich so ein eckpunkt noch an fragen habe. genau, material hatten wir schon, fortbildungskonzept. gibt es irgendwas, was sie zu diesem ganzen unterschiedlichen materialien oder von dem fortbildungskonzept, wo sie so rückmeldung geben wollen würden? positiv, negativ, anregungen, wünsche?

B: nein, es ist wirklich klasse zum verwenden. es ist übersichtlich, es ist von der optik her, finde ich, ansprechend, auch für die kinder. es ist leicht verständlich, es ist super erklärt und es gibt total viel, was man sich holen kann. also, nein, super, toll. auf alle fälle klasse.

I: sehr schön, das freut mich. dann würde ich tatsächlich nochmal so ein bisschen mehr richtung umsetzung gehen. sie haben ja schon gesagt, dass sie das eine oder andere auch schon anwenden. ob sie mir vielleicht einfach nochmal, vorhin haben sie schon eine situation erzählt. aber ob sie jetzt so aus den letzten wochen, mir vielleicht einfach nochmal eine situation schildern wollen, wo sie sagen, ja, das habe ich jetzt durch perma.teach vielleicht wieder in erinnerung geholt? oder das ist sowas, was, was sie jetzt unter perma.teach oder unter der haltung letzten endes im alltag, im unterricht sehen würden?

B: Ü3 Ja, ganz viele, ganz viele kleinigkeiten einfach. * verschiedene übungen, so bewegungsübungen, auflockerungsübungen aus dem perma. dann über ziele sprechen, ziele formulieren, kleine ziele. dann auch über erreichte ziele sprechen und über den verlauf, wie das ziel erreicht wurde, dass man das auch immer wieder mit den kindern gemacht hat. dann auch in meiner art mit den kindern zu sprechen, bewusst darauf zu achten, es ist dir noch nicht gelungen. oder auch über die fehler. also, wir haben zum beispiel umgestellt im, im mathebuch zum beispiel, wenn die kinder fehler haben, dann machen wir einen punkt daneben. das habe ich immer schon gemacht und unten habe ich ein v früher hingeschrieben. dann wussten die kinder, auf der seite ist was zu verbessern. und wenn das verbessert war, wurde das v durchgestrichen. und jetzt ist aber kein v mehr da auf der seite, sondern unten eine lupe und da wissen die kinder, da müssen sie jetzt ganz genau schauen. ich habe das auch vorzeigt mit der lupe und wenn das verbessert wurde, wird aus der lupe eine sonne.

I: ach schön. sehr schön. kann ich noch ganz kurz bei dem beispiel bleiben. wie, wie haben sie die reaktion von den kindern erlebt, also von diesem, von diesem wechsel? haben sie da irgendwie bei den kindern auch was *.

B: nein, das war nur für mich. das war eigentlich nur für mich ein wechsel, weil in der ersten zweiten, das am anfang mit dem verbessern so noch nicht war. also das habe ich eigentlich von früher. letztes jahr habe ich eine dritte, vierte gehabt, das habe ich früher immer so gemacht. das v hat die, haben die kinder in diesem jahr eigentlich, glaube ich, noch nicht erlebt. das habe ich gleich einfach mal so gemacht, gleich von anfang an eigentlich. * was haben wir noch? ja, eben das mit den, mit den stärken *. ah ja, was ich noch gemacht habe, genau, und zwar, wie wir da uns mit stärken beschäftigt haben, habe ich so komplimente-kärtchen aus dem internet mir gesucht und laminiert. ganz nette bilder und da waren komplimente dabei. und dann haben wir einfach auch besprochen, was ist ein kompliment und was bewirkt ein kompliment. wie fühlst du dich, wenn du ein kompliment bekommst, wie fühlst du dich, wenn du jemanden ein kompliment gibst und der sich dann freut und so. und dann durften die kinder *, zuerst einmal haben wir uns die komplimente angeschaut. ist ja auch, ist immer ein prozess, bis das ganze auch verstanden wird und so. aber dann durften die kinder komplimente verteilen, so im kreis. und wie das dann halbwegs geklärt war, wie das funktioniert, was das bedeutet, habe ich dann ein paar wochen lang die komplimente in einer schachtel bei mir im tisch gehabt. und die kinder durften immer in der früh einem anderen kind ein kompliment auf den tisch legen. aber jedes kind durfte nur ein kompliment am tisch haben. also, wenn jetzt ein anderes, als wenn ein kind kein kompliment dort hatte, hat auch irgendein kind noch eins nehmen dürfen und hinlegen dürfen. und ich habe dann auch geschaut, natürlich, dass bei jedem kind ein kompliment am tisch liegt. und so hat einfach jeden Tag in der früh ein kind etwas nettes gelesen, gleich zu beginn des tages und das war eigentlich ganz, eigentlich ganz nett. nur, ich habe es jetzt wieder aufgehört, weil ich einfach finde, wenn solche dinge zu lange passieren, dann haben, dann verlieren sie an wert.

I: ja.

B: und ja, das haben wir halt auch eine zeit gemacht, jetzt ist wieder schluss damit. jetzt haben wir uns in letzter zeit mit glücksmomenten beschäftigt und am ende der woche *. also die kinder hatten am anfang der woche auf dem hausübungs, also hausaufgabenheft, wo sie hausaufgaben eben aufschreiben, eine blase, wo oben steht glücksmoment, mein glücksmoment. und da haben sie die ganze woche darüber nachdenken können und am ende der woche haben wir dann über den glücksmoment der woche berichtet. war eigentlich auch recht nett. * was haben wir denn noch? *Ü3* ja, sonst fällt mir jetzt momentan nicht mehr ein.

I: das macht gar nichts. das sind ganz, ganz schöne, schöne momente und sie haben am anfang gesagt, es sind nur lauter kleine sachen. aber das ist ja das schöne, dass es manchmal gar nicht so viel braucht, dass man so, so schöne kleine momente auch schafft und dass die, die vielen kleinen

momente, letzten endes das ja nochmal ausmachen. und nicht das eine große und dann passiert nichts, sondern immer wieder wechselnde kleine momente. haben sich dann bei ihnen auch irgendwelche rituale oder so etabliert oder einfach so, so phasenweise, was, worauf sie sich fokussieren?

B: nein, es sind eher phasen. weil es einfach tatsächlich so ist, dass ich finde, je, je länger man gewisse dinge *, oder nicht je länger, aber bei gewissen dingen muss man irgendwann einmal feststellen, das ist jetzt, das machen die kinder nicht mehr bewusst. es ist ihnen nicht mehr so wichtig, es verliert an Wert, das wird schon langweilig und dann braucht es einfach wieder ein bisschen einen abstand, eine pause.

I: ja. was ja auch wieder ein absolutes feingespür auch braucht für die kinder, um zu merken, wann, wann braucht es einfach jetzt vielleicht stärken, vielleicht glücksmomente. und wann ist jetzt tatsächlich der moment erreicht, wo jetzt auch wieder vielleicht auch was neues geschaut werden muss.

B: genau. also eine sache habe ich auch noch gehabt und zwar zum damit *, also kinder, die nicht so schön schreiben, damit sie daran erinnert werden, dass sie besonders schön schreiben sollen, habe ich so schön verzierte muscheln hingelegt auf den tisch. aber das war dann nach einer zeit auch wieder vergessen. also das sind so dinge, die man halt einfach ja, ab und zu wieder wechseln muss. oder auch, was ich auch gemacht habe, so am ende der woche den wochenrückblick. diese woche, da waren so satzanfänge. diese woche ist mir gut gelungen, diese woche hat mir besonders gut gefallen. also das war einfach einmal eine phase, da haben wir das mit den kärtchen gemacht im sitzkreis am ende der woche. und jetzt haben wir halt ähnlich mit dem glücksmoment, dass man halt auf verschiedene arten immer wieder auch solche, solche dinge macht. ich glaube, wenn man jede woche jetzt nur die kärtchen nimmt, ich glaube, beschäftigen sie die kinder dann irgendwann nicht mehr so bewusst damit.

I: ja, das stimmt.

B: und sie hören vielleicht auch nicht mehr so zu.

I: ja. haben sie bei den kindern eine veränderung gemerkt? ich meine, manche kinder haben oder einige kinder haben das ja dann quasi von anfang an von ihnen so mitbekommen. aber so aus der erfahrung heraus, gibt es irgendwas, wo sie merkten, dass die kinder anders reagieren, wo ja so ihr an ursprungsmotivation her ist, da das auszuprobieren?

B: * ja, ich weiß nicht, ob das jetzt deswegen ist, oder, ja. also, es, ich merke schon, wenn sie irgendwo ein erfolgserlebnis haben, dass dann sagen, zum beispiel *. oder heute hat zum beispiel ein kind den anderen recht viel geholfen und das hat auch bei den stärken dabei gehabt ich bin hilfsbereit, ich kann anderen gut helfen. und das kind hat dann heute gesagt: "ich habe heute so gut geholfen, weil ich weiß, dass ich, weil ich weiß, dass das eine stärke von mir ist." also doch, es bleibt schon was hängen.

I: sehr schön. * aber gerade, weil sie am anfang gesagt haben, dass so der gedanke war bei den, bei den, ja, vielleicht unruhigeren kindern oder so, dass sie da nochmal methoden bekommen, um da einfach auch nochmal was machen zu können. haben sie den eindruck, dass sie da nochmal neue herangehensweisen haben?

B: vielleicht nicht unbedingt neue. eher die, in der art, die dinge, die wir schon gemacht haben, aber es geht halt um das dranbleiben. man muss immer wieder, immer von neuen, das ist halt die herausforderung. dass man das trotzdem immer wieder reinbringt, auch wenn man einen stoffdruck hat.

I: also letzten endes dieses bewusstsein schaffen und halt diese, diese erinnerung daran, was man ja eigentlich alles schon mal gemacht hat oder was man schon an ideen, an guten ideen hatte, dass man die doch immer wieder, ja, wieder dazuholt.

B: genau.

I: ja, das verstehe ich. also gerade wenn man dann in so einem tunnelblick ist, wenn besonders viele leistungsnachweise oder stoffdruck da ist, dann fällt leider gottes oft sachen raus, die man eigentlich so als schöne momente hat. wo ich zumindestens bei mir als lehrkraft sagen kann, das sind doch die momente, in denen man ja eigentlich selber auch aufblüht. weil das natürlich nochmal ganz anders ist, solche schönen momente mit den kindern auch zu teilen, als im leistungsdruck zu sein. aber der dann wahrscheinlich vorrang hat. * genau. ja, haben sie reaktionen von eltern oder rückmeldungen von eltern bekommen?

B: * nein, eigentlich diesbezüglich nicht. was mich immer wieder wundert, ist, dass die eltern, also das, dass es eigentlich nicht sehr viele beschwerden gibt über dieses eine kind. weil ich doch glaube, dass sie, dass die anderen kinder sehr tolerant sind und sehr verständnisvoll und zu hause gar nicht so schimpfen, wie, wie sie vielleicht * schimpfen könnten, wenn man nicht zu viel daran arbeiten würde. dass sie verständnis haben, dass sie versuchen, das positive zu sehen, oder wenn was positives mit dem kind passiert, dass man es hervorhebt. das vielleicht, dass das zuhause weniger beschwerden gibt von den kindern, glaube ich, als man eigentlich meinen würde.

I: * sehr schön. fällt ihnen das im alltag dann auch auf, also im umgang in der gruppendynamik?

B: nein, es ist schon unheimlich anstrengend. aber ja, sie reagieren verständnisvoll, wenn ich sie darauf hinweise. wenn ich sie an gewisse dinge erinnere, über die wir geredet haben, dann geht es wieder. aber es ist halt mühsam.

I: ja, völlig verständlich, ja. sind ihnen sonst irgendwelche veränderungen bei den kindern aufgefallen? vielleicht auch so, ja, oder die reaktionen auf ihre, auf ihre glücksmomente, auf die stärken? wie haben denn die kinder so am anfang reagiert? weil Schule ist ja am anfang ganz oft mit was anderem versehen und manche kennen das ja gar nicht, dass sie sich gezielt mit ihren stärken und ressourcen auseinandersetzen. gab es da irgendwelche reaktionen?

B: nein, also eigentlich jetzt nicht nicht erwähnenswertes, muss ich ehrlich sagen.

I: okay. was ja durchaus was positives sein kann, weil sie das ja dann vielleicht einfach auch schon davor mitbekommen haben, dass man eben nicht nur auf das negative schaut, sondern dass es ganz, ganz wichtig ist, auch auf die eigenen positiven aspekte zu schauen, von daher ist das *

B: ja, also das habe ich am anfang auch gesagt, dass wir einfach durch diese perma fortbildungen, durch die stärken.cafés dann auch bewusst geworden ist und auch meiner chefin. wir haben beide gesagt, es gibt eh viele von diesen dingen, die wir ohnehin vorher schon gemacht haben und mit denen wir aber gewisse art und weise ohnehin schon im Unterricht gearbeitet haben. also das war jetzt nicht lauter neues. und ja, ich denke, dass wir uns auch vorher schon mühe gegeben haben, das positive hervorzuheben und die kinder zu stärken und ein positives klima zu schaffen.

I: also eher schon so eine grundhaltung entspricht, die ja dann auch nochmal eine ganz andere außenwirkung hat. also wenn man vom grundsatz her ja schon dieses positive bild vom, vom kind einfach auch lebt und auf die wertschätzung und empathie einfach auch achtet, dann strahlt man ja noch ganz was anderes aus, wie wenn das jetzt einfach nur ein methodenkoffer ist. das stimmt schon, dann nehmen es die kinder auch nochmal ganz anders an.

B: genau. und was für uns dann eigentlich auch ein positives erlebnis war, war eigentlich die bestärkung oder auch die bestätigung, dass wir es ganz gut gemacht haben wahrscheinlich, bis jetzt. wenn, wenn wir es richtig gemacht haben, also wenn wir, wenn wir es richtig umgesetzt haben. aber die ideen, die methoden waren schon vorher da, einige und das hat uns natürlich bestätigt oder ja, gestärkt.

I: sehr schön. das ist ja auch eine schöne erkenntnis, weil oft macht man sachen und macht es einfach aus einem, aus einem gefühl heraus. oder was man einfach schon sich selber angeeignet hat und dann im rahmen von so einer, von einem fortbildungskonzept, das ja manchmal noch eine ganz andere qualität hat, nochmal zu hören, dass das, was man davor einfach gemacht hat, offensichtlich auch der richtige weg ist #genau# oder ein richtiger weg ist.

B: also, das war schon ein schönes, schönes erlebnis oder schönes gefühl, zu sehen, hey, wir sind eigentlich eh auf einem richtigen weg. da passier ja eh schon einiges bei uns.

I: ja, sehr schön. * nochmal, so ein bisschen auf sie als lehrperson oder als lehrkraft. was ist denn die schönste veränderung oder der schönste moment, den sie jetzt irgendwie so mit, mit dem, mit der positiven bildung oder dem perma.teach oder was die davor schon gemacht haben, jetzt so verbinden? wo sie sagen, ja, da ist mir so richtig das herz aufgegangen?

B: also, das schönste ist tatsächlich, dass ich ganz bewusst * genieße, wenn die kinder, wenn man, wenn man so eine stunde gemeinsam machen oder so eine einheit gemeinsam machen, wo die kinder * ihre stärke, eine stärke oder ein erfolgserlebnis bewusst einfach verbalisieren. darüber

nachdenken und ihnen dann auffällt, ah ja, es war die wochen, was ganz klasse ist, oder ich habe die woche ganz, ganz toll hingebracht. das finde ich das schönste, wenn man dabei ist und sieht, wie den kindern das bewusst wird * und, und weiß, also, hätte man diese stunde oder diese einheit jetzt so in der form nicht gemacht, dann hätten die kinder gar nicht darüber nachgedacht und diese situation nicht erlebt. also, das, das finde ich am schönsten, wenn man einfach das mitkriegt, dass ein kind stolz auf sich ist oder freude hat mit sich selbst aus einem bestimmten grund. weil etwas gut gelungen ist oder etwas besonders schön war.

I: ja, das ist echt *

B: das sind einfach momente, die ich, finde ich, also die für mich eigentlich die, die schönsten sind, ja. weil man selber dann auch rausgeht und ja, sich gut fühlt.

I: ja, das stimmt. ich habe mit meinen studierenden habe ich mal auch sowas in die richtung gemacht. ich habe es damals unter warme dusche verbucht. gut, ich habe jetzt junge erwachsene, das ist nochmal ein unterschied. aber die sollten sich gegenseitig eine situation erzählen, in der sie einfach nur agiert haben. jetzt gar nichts besonderes, sondern wo sie aktiv waren. und der gesprächspartner sollte so wirklich mit dem ressourcen-ohr zuhören und einfach mal hören, was höre ich eigentlich an stärken raus und dann sollten sie das rückmelden. und es war so spannend, weil letzten endes auch diese erkenntnis war, mensch, also, erstens habe ich wahnsinnig viele stärken rausgehört, egal was das für eine situation war, ob das jetzt an der kasse war oder irgendwo, es sind immer irgendwelche wirklich guten stärken mit dabei gewesen. das war die erste erkenntnis. die zweite erkenntnis war tatsächlich bei demjenigen, der die stärken zurückbekommen hat. da waren welche dabei, da waren die sich so jetzt gar nicht bewusst, aber haben wir dann noch ein bisschen gesprochen darüber. also, man hat auch wieder neue stärken erkannt. und die dritte erkenntnis, die ich wirklich am schönsten fand, war wirklich dieses, ja, eigentlich hätte ich mir manches davon vorher auch gedacht, aber ich spreche es halt nicht aus. also, wenn mir was auffällt, was jemand quasi nicht so ideal reagiert, dann melde ich das eher mal zurück und sage: "ey du, vielleicht solltest du da nochmal darüber nachdenken" oder: "ich hätte es vielleicht anders gemacht". aber dass ich nach so einem, so, so vermeintlich kleinen situationen rückmelde: "hey, klasse, wie du da jetzt reagiert hättest, wäre mir jetzt so nicht eingefallen" oder: "wow, wie geduldig du in der situation warst". und das war tatsächlich die erkenntnis, worüber ich mich am meisten gefreut habe, wo sie dann gesagt haben, ja, eigentlich müssten wir uns alle wieder an die nase packen und in alltagsgesprächen nicht nur negatives rückmelden oder uns einfach nur unseren teil denken, sondern ganz bewusst auch mal aussprechen, wenn uns solche kleinen, tollen momente einfach auch auffallen.

B: hmhm. (zustimmend)

I: was ja dann auch wieder in richtung kollegium geht. das ist mir dann so gekommen, ja, und wann machen wir das im kollegium? also das ist, da ist man ja auch oft viel im austausch mit kollegen,

über, über alles mögliche, über bestimmte situationen und man ist oft lösungsorientiert und oft konstruktiv. aber dass man wirklich so rückmeldet: "hey mensch, das, was ich jetzt mitbekommen habe, das find ich richtig klasse" und das dann noch beschreibt, was man eben gut findet, das findet häufig gar nicht so statt.

B: hmhm. (zustimmend)

I: hat sich da bei ihnen im Kollegium auch nochmal was getan? oder sie sind hauptsächlich mit ihrer chefin im austausch, oder?

B: genau, wir sind ja eigentlich nur zu zweit. wir sind eigentlich nur zu zweit und nein, da ist sowieso, also, das machen wir sowieso, doch.

I: sehr schön. und das ist so wichtig, weil letzten endes, wenn wir selber erfahren, wie sich das anfühlt, wenn ich auch positives rückgemeldet bekommen habe, dann fällt es mir nochmal viel leichter. und behalte es ganz anders im hinterkopf, dass das auch, dass ich diese momente auch für die kinder dann schaffen möchte. naja, es ist sehr schön, wenn sie sich da gegenseitig auch darin stärken können.

B: hmhm. (zustimmend)

I: ja, mir geht es auch tatsächlich so wie vorher, ganz am anfang schon gesagt, so ein bisschen um die professionsentwicklung. also um die eigene persönlichkeit, was die positive bildung mit der eigenen persönlichkeit so zu tun hat. vielleicht können sie mich da so ein bisschen mitnehmen, was das mit ihnen als, als person so gemacht hat, sich mit diesen positiven bildungen und so auseinanderzusetzen, oder ob es was mit ihnen gemacht hat?

B: nein, also ganz ehrlich, ich kann keine veränderung feststellen. es ist nicht so, dass ich jetzt merke, durch perma bin ich im unterricht anders oder fühle ich mich irgendwie * mehr gestärkt oder so, nein. nein. aber ich denke, es ist vielleicht auch deswegen, weil bei uns wirklich vorher in der schule schon viel in die richtung stattgefunden hat. ja, bei uns ist einfach der umgang sehr positiv, sehr wertschätzend, sehr unterstützend. ja.

I: das ist auch tatsächlich das, was ich bei ihnen jetzt die ganze zeit schon rausgehört habe. dass ist, dass es zwar gut war, sich nochmal daran zu erinnern und bestätigt zu bekommen, dass es so, dass es gut ist, wie man agiert, aber dass es letzten endes eine grundhaltung war, die einfach davor schon vorhanden ist. dann würde ich die frage nochmal anders formulieren, vielleicht ein bisschen passender. warum meinen sie, ist denn diese haltung, die jetzt, jetzt hier in dem rahmen mit perma.teach oder positiver bildung betitelt wird, aber warum, meinen sie, ist das so wichtig, dass man diese haltung hat im pädagogischen kontext, in der schule?

B: na, weil meiner meinung nach das voraussetzung ist, dass man überhaupt * in diesem beruf tätig sein kann. * weil, bevor man den kindern irgendwas beibringen kann, muss man schauen, dass es

ihnen gut geht und dass sie sich wohlfühlen, dass das klima passt. weil sonst das lernen meiner meinung nach gar nicht stattfinden kann. und weil ich auch glaube, dass man sich in dem beruf sonst ja auch gar nicht wohlfühlt. wenn, wenn das klassenklima nicht passt, wenn man sich selbst nicht wohlfühlt, wenn sich die kinder nicht wohlfühlen. also für mich ist das voraussetzung, dass, dass man auf diese art und weise eine angenehme atmosphäre schafft und, und dann kann man auch gut lernen.

I: ja. sehr schön gesagt. ja *

B: und ich glaube, dann ist man auch offen für verschiedene methoden, für neues. man muss ja auch immer wieder was neues ausprobieren und darf nicht in einem trott stecken bleiben irgendwo. und ich denke, wenn man, wenn man diese grundeinstellung nicht hat, dann, * ja, glaube ich, bleibt man irgendwo stecken oder verliert auch vielleicht so den, den weitblick, * gerade in der schweren personalsituation momentan. *

I: ja, auf jeden fall.

B: für einen selber, also gerade wenn man jetzt so schwierige situationen hat mit kranker kollegin und keine teilungsstunden und dem ganzen rundherum, was man alles ausfüllen und erledigen muss. wenn man da immer nur das negative sieht und selber nicht diese perma haltung einnimmt und, und einfach auch die positiven dinge herausholt aus, aus allem, was man machen muss oder kann oder soll, dann, glaube ich, wird es auch einfach schwierig. dann wird einem irgendwann auch die motivation fehlen.

I: * ja, von der eigenen begeisterung für etwas zu der begeisterung von den, von den kindern. und wenn, wenn dann eine begeisterung da ist, ist natürlich das lernen auch nochmal ganz anders, ja. gut. * gibt es denn irgendwas, was, was für sie noch wichtig ist, was so noch gar nicht irgendwie platz gefunden hat? was sie gerne noch sagen wollen würden?

B: * nein. das einzige, das habe ich vorhin schon gesagt, dass, also, dass ich denke, irgendwo gibt es dann auch grenzen. man kann nicht immer nur die stärken sehen. aber nein, ich finde es eine tolle sache.

I: sehr schön. dann ist so meine letzte frage, die das so ein bisschen nochmal abschließt und im perma gedanken auch nochmal bleibt. wofür sie letzten endes dankbar sind. ob sie so drei punkte ungefähr sagen können, wo sie sagen, ja, das ist jetzt so, muss nicht rein perma sein, aber so in dem zeitraum, in dem sie jetzt da teilgenommen haben. das sind drei sachen, für die sie dankbar sind.

B: dankbar *, achja, genau, das haben wir auch gemacht, so ein danke-plakat haben wir aufgehängt in der aula. da durften die kinder mit post-its auch einfach irgendwas draufschreiben, wofür sie dankbar waren. das war auch ganz nett eigentlich. also, ich bin dankbar dafür, dass, dass man einfach

wieder neue inputs bekommen hat, die auch dann beim probieren recht schön waren oder zu schönen erlebnissen geführt haben. auch für den austausch und für die netten kollegen, die man auch kennengelernt hat. und, * ja, schon dafür, dass, dass mit meiner chefin schrägstrich kollegin, die zusammenarbeit so gut funktioniert. und vielleicht auch durch verschiedene perma dinge, * noch, noch besser funktioniert oder manche dinge einfach, * ja, noch einfacher oder vielleicht auch zu wort gekommen sind oder zur sprache gekommen sind eben. ja, dass manche dinge durch perma vielleicht zur sprache gekommen sind, genau.

I: sehr schön. dann auf jeden fall vielen, vielen dank. es sind ganz viele sachen, ganz viele schöne sachen, die ich jetzt irgendwie so noch mitbekommen habe, die im rahmen von perma einfach nochmal da waren. schön zu hören, mit welcher begeisterung sie letzten endes auch davon erzählt haben und von diesen vielen, vielen kleinen momenten. also man merkt ihnen wirklich an, so, dass dieses, diese haltung, dieses wertschätzende, dieses mit den kindern und für die kinder, dass ihnen das ganz, ganz wichtig ist. und dafür auf jeden fall vielen, vielen dank. wie geht es weiter? ich, beziehungsweise entweder von mir oder von der, vom forschungsteam, gibt es auf jeden fall so eine kleine rückmeldung, was so die erkenntnis von dem forschungsvorhaben war oder ist. und wenn sie nochmal irgendwelche fragen oder anliegen haben sollten oder auch an mich, dann können sie sich gerne jederzeit melden. und haben sie noch irgendwelche fragen?

B: nein.

I: sehr schön. ich bedanke mich auch nochmal. auf jeden fall ist es mein erstes interview, ich war super nervös am anfang. und habe wir gedacht, um gottes willen, jetzt habe ich da meinen leitfaden mit zig fragen. ich weiß noch gar nicht so genau, wie und was und wo, aber sie haben es mir so leicht gemacht, weil sie einfach so schön erzählt haben. und von daher, ihnen vielen dank und ich wünsche ihnen auf jeden fall eine schöne zeit. und hoffentlich auch im nächsten jahr wieder eine engagierte kollegin, die mit ähnlichem herzblut dabei ist, sodass man auch wieder viele schöne momente einfach genießen kann und nicht nur quasi dieses kerngeschäft unterricht hat, sondern einfach auch nochmal ein bisschen schöne momente mit den kindern auch schaffen kann. auch weiterhin.

B: dankeschön. danke, ihnen auch viel erfolg.

I: danke.

B: für das studium.

I: danke, tschüss.

B: tschüss.

Anhang C: Interview 2

22.05.2023 14 Uhr

I: so (LÄCHELT). sehr gut. genau, du kannst jederzeit zwischenfragen stellen, wenn irgendwas ist.

B: hm (bejahend).

I: du meldest dich einfach. ich muss ein kleines bisschen aufs hochdeutsch hinweisen.

B: ja.

I: weil ich danach ein programm habe, dass im idealfall möglichst viel versucht, ähm, zu verschriftlichen, und dialekt ist zwar wahnsinnig schön, und ich liebe den persönlich sehr, aber das programm tut sich dann umso schwerer (SCHMUNZELT).

B: ich werde es versuchen. passt. ja.

I: sehr gut. vielen dank. genau. dann bin ich erst mal so ein bisschen neugierig, wer du so bist als lehrperson. wie lange du schon da arbeitest, wie lange du schon lehrer bist.

B: ja, ich bin seit, glaube ich, jetzt, 27 jahre lehrer, bin jetzt an dieser schule in bad häring seit elf jahren, und dort *, damals ist die leiterstelle ausgeschrieben gewesen und habe ich mich beworben und hab die bekommen, und war bis letztes jahr schulleiter und lehrperson. und wir sind jetzt eine klasse mehr geworden. wir sind jetzt achtklassig, und ab acht klassen, bin ich freigestellt, das heißt, ich habe keine eigene klasse, heuer das erste mal, was *. vorerst habe ich gesagt, ich nehme nur eine schule, bei der ich auch unterrichten kann. und jetzt heuer bin ich aber froh, keine klasse zu haben, weil das letzte jahr war sehr zehrend und fordernd mit corona noch und die vierte klasse mit schularbeiten, deutsch, mathematik. Und privat dann auch noch *, hat es eine sache gegeben. jetzt war ich heuer wirklich froh, mich nur auf eine sache konzentrieren zu können, schule leiten und nicht auf die Kinder dann auch noch. man ist ja immer so hin- und hergerissen, man glaubt, für die klasse zu wenig zu tun oder für die direktion zu wenig zu tun. und ja, ist eine schwierige situation. heuer ist es so, dass ich auch unterrichte, aber nur, also unterstützend, speziell mit den schülern in der ersten klasse. lesetraining oderzehnerübertritt, zehnerunterschreitung. dann, wenn ein kind *, wir haben spezielle kinder, die sich schwerer tun in anderen schulstufen, und mit denen arbeite ich einmal bei mir. und das ist sehr angenehm für mich, weil das sind ein oder zwei kinder, kann ich mir wirklich zeit nehmen, das, was ein klassenlehrer sonst nie *, was eben nie möglich ist.

I: ja, absolut verständlich. ja, schon mal vielen dank für deinen ersten eindruck oder von *, von dir einfach, was immer ganz spannend ist, wer ist *, wer sitzt einem denn eigentlich so gegenüber? soll ich ein bisschen was von mir erzählen, dass du auch weißt, wer *, wer da sitzt (LACHT)?

B: ja.

I: also, ich bin gelernte erzieherin und bin jetzt das siebte jahr an der fachakademie und unterrichte eben die zukünftigen, erzieher und erzieherinnen, und eben in diesem ganzen *, bei uns nennt sich das praxismethodenlehre. also, alles, was so richtung praxis geht, praktika und die ganzen methodischen vermittlungen, wie die *, die später einfach mit den kindern arbeiten. und in deutschland ist es ja eine breitbandausbildung, also die zukünftigen, erzieher und erzieherinnen, die sind ja von null bis 27 dann, haben die klientel. genau, und da bin ich eben auch noch klassenlehrerin, deswegen verstehe ich das sehr gut, wenn man viele unterschiedliche baustellen hat. man hat halt als *, als klassenlehrerin, wenn man die hauptverantwortung hat, relativ viel zu tun, und das ist nochmal was anderes, wenn man, in anführungszeichen, nur unterrichtet oder nur einzelne einfach auch nochmal begleitet. was ja eigentlich viel *, manchmal viel schöner ist, wenn man sich die zeit nehmen kann und nicht diesen druck hat, den man ja häufig mit *, mit der ganzen klasse auch hat. deswegen kann ich das gut nachvollziehen, was du gesagt hast.

B: und wo in deutschland unterrichtest du?

I: in traunstein, also nahe salzburg.

B: ah. okay. ja.

I: nahe der österreichischen grenze. wir haben auch ganz oft studierende. bei uns nennen sich die *, die auszubildenden studierende, die dann in salzburg, sprich in österreich, auch das praktikum machen.

B: ah. okay. ja, und du hast aber gar keinen bayerischen einschlag.

I: nee, ich bin tats *

B: oder

I: nee, überhaupt nicht. ich bin hochdeutsch erzogen worden. also, meine eltern waren der meinung, war irgendwie noch so der rest generation, der der meinung ist, dialekt gehört sich nicht, obwohl ich eigentlich auch *. ich bin münchner kind, also eigentlich schon *, schon mit sehr viel dialekt aufgewachsen, aber, mei, erziehung halt (LACHT).

B: ja, genau. aha. ja.

I: okay, gut. wie bist du auf die *, auf das perma.teach aufmerksam geworden?

B: durch die schulqualitätsmanagerin von unserer bildungsregion, die hat gesagt, andreas, ich hab da etwas, das würde ganz gut zu dir und deiner schule passen. möchtest du das nicht machen? dann hat sie mir das geschickt. ich habe mir das durchgelesen und war dann gleich feuer und flamme, weil wir vorher *, jetzt müssen wir *, Quali *, jetzt fall *, fall *, fällt mir gerade die abkürzung nicht ein. wir müssen *, müssen ja so einen plan erarbeiten für die schule, schulentwicklungsmäßig, und da ein Thema erarbeiten. früher war das sqa, und da war unser thema tugenden und umgang

*, wertschätzender Umgang miteinander, und das ist jetzt praktisch für uns die fortführung, weil das geht ja genau in dieselbe richtung. und, so haben wir jetzt für das, was wir machen müssen, von schulansicht her, einfach das fortgeführt. es hat einen anderen namen, es nennt sich anders, a *, aber vom inhalt her hat sich nicht sehr viel verändert. es ist nur noch genauer und noch mehr unterstützend durch das buch, das wir da bekommen jetzt für die kinder, durch die *, durch den fragebogen, durch die perma.post, wo immer sehr viele sachen drinnen waren. durch den knietzsche, der *, der bei den kindern voll eingeschlagen hat, schon. und auch *, auch bei den lehrpersonen, weil dieser macht es so toll und bringt es ist auch wertiger * wertiger ist das ganze geworden.

I: sehr schön. das heißt, so hauptsächlich hat dich angesprochen, dass es was ist, was ihr davor einfach schon so ein bisschen in die richtung schon hattet?

B: ja.

I: also, war das so der hauptpunkt.

B: das war ein punkt und für uns *, unseren erziehungsplan, dass wir das gleich dafür hernehmen können.

I: hm (zustimmend).

B: das war auch schon *, wir haben ein schulleitbild erstellt. das war schon vor, ja, acht jahren, und wo das erste mal so etwas gemacht werden musste von den schulen, und da habe ich auch das verbunden. ich wollte sowieso ein schulleitbild machen, und dann war das gleich das, was ich dem bundesministerium abgeben habe können. ich bin sehr dafür, ressourcen schonend zu arbeiten und etwas, was wir sowieso wollen, das dann gleich dafür herzunehmen. wir haben da auch *, wir waren zum beispiel leseschule und haben das auch dafür hergenommen. also, wir haben nicht speziell etwas erarbeitet, um so einen erziehungsplan und schulentwicklungsplan zu erstellen, sondern haben etwas, woran wir sowieso arbeiten wollten, dafür hergenommen. Und das war als perma.teach genauso mit den untersuchungen, die gemacht werden, weil wir müssen ja das auch evaluieren, und das ist praktisch so jetzt schon passiert, und da müssen wir nicht extra.

I: * sehr gut.

B: zwei *, zwei fliegen, mit einem schlag so in die richtung.

I: ja, was ja eigentlich perfekt ist, weil gerade in der jetzigen zeit so viel mehraufwand ist häufig einfach gar nicht mehr zu stemmen. und wenn man eh schon in die richtung geht, wenn man dann einfach mehrere sachen gut kombinieren kann, um *, um möglichst viel für sich und für die eigene schule rauszuholen, dann ist es natürlich ein großer vorteil (NICKT).

B: genau. so sehe ich das auch, und das wird von meinen kollegen und kolleginnen sehr stark honoriert, dass ich das so mache, weil *, weil ja schon die arbeit wird immer mehr und das, was von

uns verlangt wird, immer mehr. nicht nur arbeit mit kindern, sondern das rundherum, und wenn dann da jemand schaut, dass nicht noch mehr ist, dann ist es natürlich sehr positiv.

I: wer hat denn aus deinem team *, oder wie groß ist denn dein team?

B: wir *, wir haben acht klassen, es sind acht lehrer *, klassenlehrer, und derzeit ist nur eine mit den reststunden, weil wir eigentlich eine halbe lehrverpflichtung zu wenig haben. nächstes jahr bekommen wir dann noch eine *. also, da wird die, die jetzt zur hälfte *, hälfte unterrichtet, bekommt eine ganze lehrverpflichtung, und eine halbe kommt noch dazu. also, da sind wir dann *, nächstes jahr sind wir dann zehn lehrpersonen, und zusätzlich sind noch zwei religionslehrerinnen, und ja, und ich. Ja.

I: und wer von denen *

B: ja, passt.

I: wer von denen hat an der fortbildung noch teilgenommen oder warst das hauptsächlich du?

B: na, wir ma *, machen *, nehmen alle daran teil. wir haben als gesamte schule *, bei der präsentation zum beispiel haben *, haben wir uns alle in der *, in der schule versammelt vor dem computer und mit der dokumentenkamera hat das sehr gut funktioniert, dass wir *, dass man uns alle gesehen hat. und bei den stärken.cafés waren wir auch zum teil alle miteinander. das letzte stärken.café, da konnten wir leider nicht teilnehmen, weil ich den termin übersehen habe und wir zum gleichen datum einen elternsprechtag angesetzt haben, aber wir haben diesen donnerstag einen nach *, noch ein stärken.café, speziell für uns, für unsere schule. ja. und *, und wir schauen eigentlich auch beim stärken.café ist es so, dass da manche kolleg*innen in der schule sind. die sind da so zwei zu dritt oder zu viert vor einem gerät und die anderen von zu hause aus. aber wir *, wir genießen es im kollegium sehr stark und haben auch schon von unserem ersten schulen *, schulleitbild an haben wir gemerkt, dass wir gerne zusammenarbeiten. also, nicht nur schulisch, sondern außerschulisch oder über das unterrichten hinausgehend. dass wir sehr gerne und gut zusammenarbeiten, und deshalb ist das sehr befruchtend für uns und für das ganze kollegium.

I: das kann ich mir wirklich richtig gut vorstellen, weil man ja dann nicht nur den input hat und den dann irgendwie für sich verarbeitet oder mit nur ein oder zwei kolleg*innen, sondern in der tegel ist es ja so, dass sich danach ganz viele gespräche im team auch noch ergeben, die dann nochmal sehr bereichernd sind, weil ja jeder so mit eigenen ideen daherkommt und man dann gemeinsam gleich schauen kann, wie man es eigentlich für die eigene schule hernehmen kann.

B: ja. das haben wir auch *. wir haben letzte woche konferenz gehabt, und es ist immer ein punkt von mir, perma.teach, was läuft gerade, was steht an, was ist in planung, und es ist *. ich weiß es selber vom letzten jahr, in der vierten klasse sind einfach so viele sachen zu tun, dass man das gefühl hat, es *, das kann sich nicht ausgehen, wenn man da auch etwas macht. ich hab dann den

kolleg*innen auch gesagt, sie müssen einfach in die tagesplanung das einplanen, zehn minuten. das muss gar nicht länger sein, dann hat man auch zeit, weil, wenn man wartet, wann hat man zeit, dann werden *, wird man nie zeit finden. und auch kollegen haben dann so beispiele erzählt, was sie gerade machen, und dann haben die anderen, ah, das ist toll, dürfen wir das auch machen? ja, sowieso. und das ist einfach dann so ein gegenseitiger austausch, und *, und wir *, wir merken einfach immer, es braucht nicht, was ganz großes sein, es sind oft kleine sachen, worauf man schauen sollte und die man einbauen *, leicht einbauen kann, und die da *, die da sehr viel bringen.

I: kannst du mir von den *, von den beispielen irgendwie ein, zwei benennen, kurz beschreiben, dass ich mir vorstellen kann, wie ihr es umsetzt?

B: ja. ein kollege hat erzählt bei der letzten konferenz, er hat in der schule in seiner klasse die frage gestellt, ob sich die kinder vorstellen können, wer sein lieblingsschüler ist oder -schülerin. und dann war ganz betretenes schweigen bei den kindern, und sie haben hin und her *. und dann wurde ein *, ein name genannt von einer schülerin, die die klassenbeste ist, und dann haben sie über *, über leistung gesprochen haben. sie glauben, dass nur wer gut *, wer gute leistung bringt, eben dann ein *, ein guter mensch, ein guter schüler ist, oder lieblingsschüler. dann sind sie jetzt auf verschiedene sachen draufgekommen, und dann hat er gesagt, ich zeige euch jetzt, wer meine lieblingsschülerin oder mein lieblingsschüler ist. ihr dürft aber nicht verraten, weil sonst ist es für die anderen nichts neues. und dann hat er so eine box gehabt, und da sind die kinder hingegangen und haben den deckel aufgemacht, und drinnen war ein spiegel. das war dann der lieblingsschüler oder die lieblingsschülerin.

I: (NICKT).

B: ja, das *, das hat uns sehr gut gefallen, oder eine andere kollegin hat, so einen abreißzettel gemacht mit, ich weiß nicht mehr, wie sie es genannt hat, so komplimente zum abreißen oder so, und da *, da steht dann drauf, jemanden ein kompliment machen oder fünf personen grüßen oder der mama zu hause im haushalt helfen und die kinder, wenn die nach hause gehen, nicht immer, aber die dürfen sich dann so eine abreißen. und versuchen, das dann umzusetzen und erzählen dann am nächsten tag, wie es ihnen dabei gegangen ist. oder grüßen ist generell, wahrscheinlich nicht nur bei uns an der schule, ein *, ein *, ein thema, ein immer wiederkehrendes. ein thema, bei dem wir schon *, schon langsam müde werden und zermürbt werden, weil einfach der erfolg nicht so groß ist. wir weisen die kinder immer wieder drauf hin, machen spezielle tage oder *, oder wochen des grüßens, wo man schaut, dass man möglichst viele personen grüßt, unterschiedliche. und da haben die kolleg*innen dann auch den kindern zum teil den auf *, den auftrag gegeben, beim nach hause gehen, am schulweg, möglichst viele leute grüßen, und dann am nächsten tag erzählen, wie viele leute es waren und wie die reaktion der leute war und sie aufmerksam machen, das zu machen.

I: sehr schön.

B: das wären jetzt so ein paar beispiele gewesen. ja.

I: was ist denn dein schönster moment in *, in bezug auf perma.teach und mit den schülerinnen und schülern?

B: letztes jahr in der vierten klasse, kann ich mich noch ganz gut erinnern, wie wir *, wie ich ihnen das erste mal dann das buch präsentiert habe und sie darin gearbeitet haben. erst *, es war eine ganz tolle stimmung in der klasse. eine sehr positive stimmung, und die kinder waren total ruhig und haben eifrig gearbeitet, und ich habe einfach gemerkt, speziell die kinder, die eher unsicher sind, bei denen die schulische leistung jetzt nicht so ist, wie sie sich es erhoffen oder wünschen, dass sie das sehr genießen, da auf die positiven sachen zu schauen. oder, wenn wir dann als klasse etwas gemacht haben und so den verschiedenen kindern komplimente aufgeschrieben haben, und sie haben das dann gelesen. also, das waren ganz berührende momente, und als ich ihnen dann gesagt habe auf die frage, was passiert mit dem buch dann? ich habe ihnen dann gesagt, am ende der vierten klasse dürft ihr das mitnehmen, das ist euer buch. und immer, wenn es euch irgendwann schlecht geht, sollt ihr da hinein blättern und schauen und durchlesen, und das war *. also, man hat so gemerkt, bei allen natürlich, aber bei einigen kindern war das so *, so *, das ist so *. sie haben das wie einen schatz empfunden, das buch. und das war *, war sehr schön. ich selber habe auch so hoch *, ein paar up and downs. ich hab versucht, immer zum beispiel, wenn ich durch die tür durchgehe, dass ich immer lächle. das war irgendwo bei so einer perma.post *, war das drinnen. und *, und *, und das ist total toll. aber dann vergesse ich immer wieder drauf. oder irgendeiner hat einmal erzählt, er hat so *, sich einen punkt gesucht, dass er immer zehn liegestütze macht. und immer nach dem klo gehen hat er das gemacht. fas habe ich dann auch probiert, und *, und das halte ich dann schon einen monat durch, aber dann flaut es wieder ab. aber das sind so *, so sachen. immer wieder fällt mir das dann ein, dann mache ich die ein paarmal, und *. oder das, * wie heißt das, am abend, wenn man das stärken-tagebuch *. nein. nicht. wenn man so ein tagebuch *. wenn man sich fünf sachen aufschreibt, die an dem tag gut funktioniert haben *, gut funktioniert sind, dass man sich das positive herholt. das habe ich auch öfter gemacht, und jetzt schreibe ich es eigentlich nicht mehr hin. aber, wenn ich mich ins bett lege, dann denke ich dran, was ist gut gewesen an dem tag, und vorm einschlafen denke ich mir, ist das ganz etwas tolles. also, ich hab selber auch etwas mitgenommen, nicht nur den kindern das weiterzugeben, sondern für einen selber ist das auch wirklich sehr, sehr wertvoll.

I: ja, auf jeden fall. jetzt hast du schon zwei ganz spannende sachen angesprochen. zum einen so ein bisschen, wie es dir damit ging, wo ich dann gerne noch weiter einsteigen möchte. Und aber davor nochmal, wie es *, wie deine klassen oder wie du es auch von den anderen klassen mitbekommen hast. eben, ihr seid ja von anfang an schon so ein bisschen in die richtung gegangen, das heißt, es war jetzt kein kompletter cut und es ist etwas neues entstanden. aber wie würdest du sagen, haben die kinder darauf reagiert und jetzt auch im prozess, wie gehen sie mit perma um?

B: sehr gut. ich denke mir, das ist *, wird von allen, ich sag jetzt mal, von allen, es wird vielleicht ein paar ausnahmen geben, auch von allen kindern sehr positiv aufgenommen. wenn es heißt, wir machen perma.teach oder etwas zu perma.teach, dann ist das positiv bis jetzt. und die kinder wissen, sie erfahren dann etwas neues oder *, oder wir wiederholen etwas. das ist ein *, ein mehrwert, der auch von den kindern so gesehen wird. nicht schulische leistungen, und wird abgeprüft und gibt noten, sondern das ist, was darüber *, was darüber hinausgeht. und von den kindern wird das auch so *, so empfunden und so angenommen. und, na *, natürlich sind unsere erwartungen dann immer noch viel größer von den lehrpersonen, dass wir denken, ja, wenn wir das jetzt ein paarmal durchmachen mit ihnen, dann muss das so verinnerlicht sein, dass sie das von sich aus machen, und das ist leider halt nicht so. ja. nichtsdestotrotz ist einfach wichtig, immer dranzubleiben, immer wieder das zu machen, weil, wenn es von *, von 50 kindern, wenn einer sein verhalten ändert, und an denke ich mir, ist *, ist schon etwas gewonnen. m *, man will natürlich als erziehungsperson immer möglichst alle *, dass alle ihr verhalten ändern, aber das geht leider nicht. ja.

I: ja, sehr schön gesagt.

B: hm (zustimmend).

I: du hast ja doch schon ein paar Jahre berufserfahrung. würdest du sagen, dass es grundsätzlich so in der gruppendynamik und in der klassendynamik durch *, durch das viele, auf stärken achten und positive rückmeldung, dass es da auch eine veränderung gibt?

B: es *, auf alle fälle gibt es eine veränderung, auch in den wortmeldungen der kinder. wir *, wir haben morgen leichtathletik-grandprix in der nachbargemeinde, zum *, zum beispiel eben, und ich habe heute supplieren müssen. dann habe ich gesagt, ja, und da ist dann da der leichtathletik-grandprix, und da schauen wir dann, w *, wer der schnellste oder der beste ist. und im wettlaufen, weitspringen und weitwurf. und dann ist gleich von *, von drei oder vier kindern gekommen, dabei sein ist alles, und es kommt auf den spaß an und nicht aufs gewinnen. und ich denke mir, das *, das sagt dann schon einiges aus, dass von ihnen gleich von selber so etwas kommt und nicht, dass es von der lehrperson sein muss. ja.

I: hm (zustimmend).

B: und, ja, es *, es hat sich *. ich denke mal, es sind einfach immer mehr kinder, bei denen zu hause solche themen nicht angesprochen werden. ja, und wo es nur um leistung geht oder um pflichterfüllung oder solche sachen. aber es geht nicht um die personen selber, und wo sind ihre ängste oder ihre stärken? sodass persönliche werte *, werte sicher in vielen familien, auch am land und der schule. es wird nicht angesprochen oder ist kein thema zu hause. deshalb ist das halt auch eine sache, die zumindest dann in der schule gemacht wird. mein *, mein wunsch wäre es, auch *. wir können nicht aus, wir *, wir *, wir müssen den kindern noten geben, wir müssen die leistung beurteilen. das wäre mal mein erster ansatz, das komplett abzuschaffen. leider hören mir die *, die wichtigen

leute da nicht zu (LACHT). und *, und mein wunsch wäre, es in der schule das noch mehr umzusetzen. und das wird sicher ein prozess in nächster zeit, da einfach die fehlerkultur *, dass die fehlerkultur eine andere wird und auf die stärken mehr geschaut wird und nicht auf das, was nicht funktioniert. wir müssen da noten geben, da kommen wir nicht aus, aber doch, wie man das vorbereitet, wie man zur note kommt, kann *, gibt es ja auch verschiedene wege.

I: ja. was glaubst du, braucht es, um da eine *, eine andere schulkultur letzten endes zu *, zu etablieren?

B: ich denke mir * (TELEFON KLINGELT). moment, jetzt muss ich ganz kurz hingehen, weil das ist von der nachmittagsbetreuung.

I: natürlich.

B: (TELEFONIERT) *36Ü* entschuldigung. wir haben ein problem mit einem kind, das nicht mehr in die schule hereinwill, und *, und wir haben einen betreuer für die nachmittagsbetreuer *, nachmittagskinder, für 16 kinder, und er kann jetzt nicht eine zurücklassen, aber er *, er schaut jetzt, ob er noch jemanden anderen findet, sonst ruft er mich nochmal an. ja. gut. wo waren wir jetzt vorher?

I: was du glaubst, dass es braucht, mit der *, dass sich die schulkultur ändern kann.

B: ja, ja. ich denke mal, der erste oder ein schritt wäre gemacht mit dem neuen lehrplan. im neuen lehrplan stehen so viele tolle sachen drinnen, von wegen, stärken fördern, dann unterschiedlich zu, also, differ *, zu differenzieren und nicht allgemeine leistungen abzuprüfen und nicht alle das gleiche abzuprüfen, und da würde es eigentlich drinnen stehen. aber auf der anderen seite müssen wir dann, ich weiß nicht, ob dir ikm plus etwas sagt? das ist jetzt so ein standardisierter test, der an allen schulen, alle dritten und vierten klassen der volksschulen in österreich, wird der gemacht. so ähnlich, wie früher die *, die standards waren, die *, die gemacht wurden. und da müssen die kinder heuer in mathematik etwas machen und deutsch lesen. und da müssen alle zur gleichen zeit das gleiche machen, ob sie das im unterricht schon durchgenommen haben oder nicht, weil das ja eh nach lehrbuch verschieden gehandhabt wird. und das widerspricht komplett den vorgaben, die im neuen lehrplan drinnen stehen. Und da sind noch mehrere so standardisierte tests. denke ich mir, ja, das sollte mal komplett verändert werden. ja, und *, und die noten wären dann das nächste. ich hab jahrelang drei klassen mit einem pensenbuch beurteilt, also, die ersten drei jahre. In der vierten wurde es mir nicht erlaubt, aber die ersten drei jahre, und das hat wunderbar funktioniert. es ist viel mehr aufwand für die lehrperson, aber für die kinder als rückmeldung und für die eltern als rückmeldung ist das viel besser, und dann ist es auch nicht so leicht zu vergleichen. der hat einen dreier, der hat einen zweier, der hat eine eins. also, da müsste es *, sollte viel mehr darauf geschaut werden, kann der oder diejenige das, oder kann er das nicht und nicht wie gut? weil es geht nur darum, kann ich die rechnung lösen, und wenn ich ein bisschen länger brauche, ja, wo ist das problem? ja, also, da müsste von, *also, natürlich ist es für mich jetzt leicht zu sagen, von oben her das Umdenken

kommen, weil es für mich dann leichter wäre. aber ich kann zum teil nicht komplett jetzt was anderes machen, weil ich habe ja gelobt, dass ich das so umsetze das von oben. ich schaue halt dann im rahmen des möglichen, dass wir das vielleicht anders handhaben oder bei den bei *, den standardisierten tests, die wir jetzt machen müssen, wissen meine Lehrpersonen, dass ich komplett nicht hinter dem ganzen stehe und *, und wenn bei ihnen kinder nicht so gut abschneiden, sie aber wissen, dass die sonst eine gute arbeit leisten, dann ist das überhaupt kein problem, weil für mich zählt ja viel mehr, dass sie ja ein ganzes jahr oder länger schon mit dem kind arbeiten, als wie einen test da, 60 minuten an einem bestimmten tag.

I: ja, weil's ja einfach nur eine momentaufnahme ist.

B: ja, genau.

I: die kinder wissen dann irgendwann auch, bei uns sind's vergleichsarbeiten, aber im prinzip das gleiche. die spüren dann schon auch, dass da ein gewisser druck dahinter steht und druck und leistung erbringen. das ist ein bisschen schwierig.

B: das ist schwierig, und wenn man dann von schulen weiß, wo die lehrpersonen in den kindern helfen, bei den standardisierten tests, dass sie die aufgaben lösen, oder, äh, äh, beim korrigieren das irgendwie noch ausbessern und dann besser korrigieren. und nur, um besser *, als schule besser dazustehen, denke ich mir, wo ist der sinn? die sinnhaftigkeit hinter den tests? ich würde die komplett weglassen.

I: ja (NICKT). das heißt, du versuchst *, versuchst so ein bisschen die *, die möglichkeiten auszureizen, um einfach das positive in der schule auch zu lassen, so wie ich es jetzt verstanden habe, und nicht nur dieses *,diese negativverzerrung durch noten und diese *, dieses, ja, generell dieses negative, ähm, so überhand zu *, dass es überhandnimmt.

B: genau. ja.

I: das war schon so ein bisschen das, was *, was es bei den kindern ausmacht. hast du eine veränderung in deinem kollegium gemerkt?

B: * ich denke mal, speziell bei den jungen kolleg*innen ist jetzt hoffentlich das angekommen bei ihnen, dass schule auch so etwas sein darf, wo es nicht um lehrstoff geht, wo es nicht um Noten geht oder *, oder beurteilung, sondern um menschenbildung. und ich denk mal, jedem im lehrberuf hoffentlich ist klar, wie wichtig das ist, aber nur die klarheit, das zu wissen, und dann wirklich auch sich trauen, das umzusetzen, sind zwei paar schuhe, und ich hoffe schon, dadurch, dass wir jetzt bei den konferenzen, bei den perma.cafés *, dass sie da mitbekommen, wie wichtig das ist und dass das genauso schule und unterricht ist. und das, denke ich mir, hat sich schon ein bisschen geändert, und ich denke, es ändert sich immer etwas in der einstellung jeden einzelnen, wenn wir darüber

sprechen und wenn dann beispiele genannt werden und wenn sachen umgesetzt *. versucht *, wenn sie versuchen, das umzusetzen. ich denke mal, das macht mit uns allen etwas.

I: ja (NICKT). auch diese kleinen erfolge dann gemeinsam feiern zu können. ich habe was ausprobiert, und das ist *, das ist mir gut gelungen, und das ist *. damit habe ich die kinder erreicht, und das sind ja dann auch nicht nur die kleinen erfolge für die *, für die schülerinnen und schüler, sondern letzten endes dann auch die *, die kleinen erfolge für die lehrkräfte, und dass man sich das auch viel auch erzählt, weil das ist ja immer dieses gutes tun und #drüber reden.

B: ja, genau.#

I: das hilft einem ja auch selber und stärkt eigentlich das persönliche selbstbild auch nochmal.

B: ja, auf alle fälle.

I: ich habe da bei meinen Studierenden auch eine schöne erfahrung gemacht. ich habe mit denen *, die sollten sich gegenseitig eine aktion erzählen, eine, wo sie aktiv geworden sind, zu zweit. und der zweite sollte immer zuhören, welche ressourcen, welche stärken er eigentlich raushört, und dann haben sie getauscht, beziehungsweise dazwischen halt auch dieses rückgemeldet. danach haben wir reflektiert, weil bei uns geht es auch viel darum, auch dieses, wie erfahre ich das selber, und wie kann ich das dann wiederum auch den kindern und jugendlichen, mit denen sie später arbeiten, auch vermitteln? und da hatten wir zwei ganz tolle erkenntnisse. die eine war, ich habe so viele stärken erkannt, und normalerweise hätte ich die so gar nicht rückgemeldet, weil warum? also, das ist ja irgendwie klar, dass jeder stärken hat und dass man die jetzt irgendwie *, dass man bewusst auch, wenn man *, wenn jemand einem was erzählt, auch mal rückmeldet, hey, das finde ich klasse, wie du es gemacht hast oder wie geduldig du da an der kasse warst. es ging ja um keine großen sachen, sondern um so alltagssituationen.

B: hm (zustimmend).

I: und die zweite war letzten endes dieses, dass wir einfach alle sehr, sehr viele stärken haben und dass wir so oft einfach drauf schauen, gott, was habe ich gerade nicht gut gemacht, und das fand ich auch so spannend, weil es war eine übung. bei uns geht es richtung prüfungen, das heißt, die sind alle recht nervös. die sind ganz froh, wenn sie wieder so situationen haben, in denen ihnen bewusst wird, dass sie eigentlich sehr viel können und sehr viel leisten. und haben dann auch im nachhinein viel mehr drauf geachtet, sich gegenseitig immer mal wieder zu erzählen, wenn sie irgendwas mitbekommen haben, was sie gerade klasse finden. also, hat auch eine gewisse nachhaltigkeit.

B: ja. ja, das ist auf alle fälle ganz wichtig, auf das positive hinzuschauen und sich gegenseitig zu stärken und man ist schon oft verleitet, auf das *, auf die andere seite zu schauen. mir *, mir fällt jetzt da gerade ein, meine tochter, wenn die von der schule gekommen ist und hatte in mathematik

einen zweier und dann, ja, super, was hat denn gefehlt auf den einser? die denkt halt, es ist schlimm, so eine meldung, weil ich hätte selber nie einen *, einen zweier zusammengebracht in mathematik, und das ist einfach gewaltig. und man schaut aber dann doch auf das, was *, wo der fehler war.

I: (NICKT),

B: und *, und das ist schon in unserer Gesellschaft, denkt man, so tief und fest verwurzelt, dass wir fast nur oder *, oder vermehrt auf diese sachen schauen.

I: ja (NICKT).

B: ja.

I: deswegen ist es ja so wichtig, dass man da irgendwie so einen gegenpol auch noch schafft, weil manchmal braucht man das ja. Ich meine, das ist ja leider gottes so, dass wir schauen müssen, wo *, wo gibt es denn noch Entwicklungsbedarf und wo *, wo kann ich weiter stärken? aber weniger richtung, das ist falsch, sondern was sind die stärken und an dem arbeiten wir und wie können wir *, wie können wir bei dem anderen einfach auch noch unterstützen.

B: ja. und *, und deswegen merkt man auch, wenn man in diese richtung etwas arbeitet, wie gut das den Kindern tut, wie gut das den erwachsenen tut, jedem einfach. weil *, weil das einfach was schönes ist, wenn man positive rückmeldungen bekommt. und die bekommt man nicht so häufig, und dann ist es einfach ganz was angenehmes.

I: ja, und manche müssen es tatsächlich auch wieder lernen. also, nicht nur die kinder, sondern auch die erwachsenen, das anzunehmen, dass man auch mal was positives rückgemeldet bekommt und das nicht so als lappalie abtut. ja, ja, das hat schon gepasst und war gut und nicht der rede wert, sondern man muss tatsächlich ganz oft wieder neu lernen, das anzunehmen und auch als eigene stärke, ja, zu akzeptieren und drauf aufbauen zu können.

B: das stimmt. ja.

I: jetzt hast du schon ganz viel über das erzählt, was perma.teach und das *, diese *, diese auseinandersetzung mit der thematik mit dir gemacht hat. gibt es da noch was, wo du sagst, das war jetzt gerade so in deiner *, in deiner persönlichkeit, in deiner *, lehrkraft und als *, als schulleiter, das dich da noch geprägt hat?

B: * ja, geprägt weiß ich nicht, aber was ich auf alle fälle zurückmelden möchte, ist einfach die *, das werkzeug, das man bekommt. durch das buch zum beispiel, durch die verschiedenen videos, hat jeder so viel werkzeug bekommen, das ganz leicht umzusetzen ist, und in die Klasse zu integrieren ist, weil, wenn ich mir so viel übungen irgendwo im internet suchen muss und irgendwo nachlesen muss, dann geht das natürlich auch, aber es ist sehr viel mehr aufwand. und so habe ich *, bekomme ich das präsentiert und kann das eins zu eins umsetzen.

I: (NICKT)

B: und das ist wirklich etwas ganz, ganz tolles, und es hatte irgendwann mal geheißen, wir sind die pilotschulen, und das wird auf ganz österreich ausgerollt. ich denke mal, ja, das wäre die große Chance, wenn *, wenn jeder dann so ein werkzeug in die hand bekommen würde, jede lehrperson, und das *, ob er es dann umsetzt oder nicht, ist wieder etwas anderes. aber jeder hat die möglichkeiten, das dann viel einfacher umzusetze. und dann würden es auch viel mehr tun, und das hätte in dem gesamten dann einen sehr viel positiveren anklang.

I: (NICKT) ja. und vor allem *, ich habe den eindruck, es macht auch die menge an unterschiedlichen möglichkeiten und methoden und übungen aus, dass man sich vielleicht auch *, auch das raussuchen kann, was mir selber liegt.

B: (NICKT) ja, genau.

I: oder wie würdest du das bewerten?

B: ja, auf alle fälle, und auch das wissen, dass nicht immer was ganz großes sein muss, dass oft auch ganz was kleines genügt, ein paar sätze, einen impuls den kindern geben, dass sie am nachmittag etwas versuchen und dann am nächsten tag erzählen. und durch das erzählen kommen andere drauf, ja, könnte ich vielleicht auch versuchen, die das gar nicht so mitbekommen haben, und *, und die kleinigkeiten, denke ich mir, die sollen wir viel mehr schätzen und werden dadurch auch mehr geschätzt, und *, und viele kleinigkeiten sind dann etwas großes.

I: ja, auf jeden fall. du hast schon gesagt, dass du *, dass dieses angebot mit dem *, mit den modulen und mit der perma.post, also, hast ja schon verschiedene sachen benannt. gibt's irgendwas jetzt von diesen reinen, modul eins und zwei, wo du besonders gut fandest?

I: ja, das war alles gut.

I: was dir besonders hängengeblieben ist, vielleicht?

B: ja. * da bin jetzt momentan wirklich über *, überfragt, weil ich weiß noch, wie wir das erste modul in präsenz gehabt haben, in *, in der volkschule jetzt. das hat sicher mehr wert als das online.

I: hm (zustimmend).

B: weil es einfach direkt ist, weil da gibt's dann die pause dazwischen, wo auch gespräche stattfinden. aber man weiß ja, in der pause sind meistens die besten gespräche. ja (SCHMUNZELT). und *, und das vorher und nachher, und *, und ja, das direkte, das kann jetzt eine videokonferenz nie wettmachen. aber auf der anderen seite wieder, als ich das erzählt habe, auch bei kolleg*innen, dass wir da jetzt die *, das online haben von allen volksschulen, von *, von ganz österreich, die da mitmachen, vom burgenland bis vorarlberg, das wäre sonst nie möglich, und das ist *, hat wieder in einen anderen wert. also *. und *, und da nimmt man auch wieder sehr viel mit, aber andere sachen

wie *, wie beim direkten. ich habe jetzt vorhin gesagt, das direkte ist einfach besser, aber wenn ich jetzt so nachdenke, es ist anders. aber *, aber *, aber *, aber, dass man so verschiedene schulen und von verschiedenen standorten das mitbekommt, das wäre so gar nicht möglich im direkten kontakt. und das ist durch die videokonferenz möglich. und *, und das waren *, die *, die verschiedenen vortragenden, die eva sowieso, aber dann auch, jetzt fallen mir gerade die namen nicht alle ein. ja. jeder hat so seine andere herangehensweise, aber das gesamte, das einheitliche ist bei jedem gleich, und *, und das positive ist ja, das schwingt so mit und *, und das ist einfach mitreißend. wo man dann einfach sagt, ja, wenn wir das hören, das will man gleich umsetzen. jetzt beim modul zwei habe ich nicht die möglichkeit, weil ich momentan keine eigene klasse habe, dass ich das gleich umsetzen kann. aber es ist einfach ganz toll, das *, das zu hören, um das dann auch bei den kollegen nachzufragen: habt ihr das probiert? was habt ihr probiert? ja. aber jetzt ganz konkret, welche sache jetzt am besten war oder am meisten hängengeblieben ist, kann ich dir jetzt leider momentan nicht beantworten.

I: ist auch völlig in ordnung, aber gibt es zum beispiel was, was du dir noch gewünscht hättest, was *, an material oder an input, oder?

B: nein. ein weniger ist mehr, manchmal. bei der perma.post, da war gerade *, da war corona noch recht aktiv, und es waren zu viele perma.posts in zu kurzer zeit.

I: hm (zustimmend).

B: also, das hätte ich mir gewünscht, dass *, dass vielleicht länger zeit für eine perma.post war, um die umzusetzen, weil man hat oft *, ist nicht gleich dazu gekommen, die zu lesen, dann sind ein paar tage vergangen. und dann, ah ja, soll ich mal lesen und dann war schon die nächste da. und *, und das wäre, glaube ich, stressfreier für die *, für die Personen, die da mitmachen, wenn *, wenn weniger perma.posts wären oder *, oder längerer zwischenzeitraum. aber von der art und weise, wie es gemacht worden ist, und auch die cafés und die inputs, da würde ich sagen, ja, das war ideal. das hat sehr gut gepasst.

I: das heißt, du würdest es *, wenn es jetzt dann wieder neue fortbildungsmöglichkeiten gibt, würdest du es empfehlen?

B: auf alle fälle, ja (NICKT). auf alle fälle. und ich habe auch schon *, wir haben so eine kleine direktoren-gruppe, das sind wir acht direktoren. wir treffen uns regelmäßig so alle ein bis eineinhalb monate für einen austausch. der ist sehr wertvoll, weil man da so viel erfährt und *, und einfach miteinander entscheidet. und da habe ich das schon ein paar mal angesprochen, dass wir das machen. und auch die sqm, die andrea weißkopf, die für uns zuständig ist, hat das auch schon wieder mal angesprochen. und du machst ja das, und hat mich auch gebeten, dass ich irgendwann bei einer *, so einer direktoren zusammenkunft, das ist auch keine offizielle konferenz, sondern auch so ein arbeitskreis, der ein bisschen größer ist, dass ich da das mal vorstelle, auch für die anderen,

dass sie sehen, wie *, was wir da machen, weil es wirklich so wertvoll ist und ich es jedem empfehlen kann.

I: sehr schön. wenn ich jetzt als außenstehender zu dir in die schule komme und gar nicht weiß, dass ihr perma.teach macht oder so. woran würde ich es denn erkennen?

B: an der positiven stimmung in der schule.

I: hm (zustimmend).

B: ich hab das jetzt schon so oft zurückgemeldet bekommen von *, von praktikant*innen, die bei uns auch an der schule sind. von einer it-lehrerin, die *, die *, die kolleg*innen ab und zu berät, mit den chrome-books, und in der schule ist. von musikschullehrern, die bei uns an der schule sind, am nachmittag. von verschiedensten leuten, die bei uns hereingehen, die nicht regelmäßig da sind. die sagen, wenn man bei euch *, bei euch in die schule hereinkommt, ist so eine positive, angenehme stimmung. dann denke ich mir, das ist sicher perma.teach mitverantwortlich. ich sag nicht, nur, aber mitverantwortlich, dass eben die positive stimmung bei uns an der schule herrscht, die spürbar ist.

I: sehr schön. was ja so wichtig ist, weil wir brauchen ja eine *, eine anregende und entspannende und aber auch schöne atmosphäre, um wirklich gut auch lernen zu können.

B: ja, genau. ja. und das *, das wird von uns sehr geschätzt. von uns lehrpersonen wird mir auch immer wieder zurückgemeldet, bei * mit *, mitarbeiterinnengesprächen oder mitarbeitergespräche da sind *, dass die kolleginnen und kollegen so schätzen, das wertschätzende klima, das gute miteinander, das positive klima, deswegen sind sie auch so gern an der schule. und auch, wenn zwei, drei personen, die haben recht einen langen schulweg, also für unsere verhältnisse lang, also, die fahren 40 minuten mit dem auto, und *, und die sagen, sie würden aber nie an eine andere schule im moment gehen, weil es einfach bei uns so gut passt. ich denke mal, das ist die zusammenarbeit untereinander. das wird mir auch zurückgemeldet, meine person, das tue ich mich dann auch wieder schwer, das anzunehmen und zu sagen, ich bin der grund, dass es positiv ist. aber vorher hast du auch gesagt, dass man das anwenden kann, aber es wird schon ein mitgrund sein und das wird von allen so empfunden. deswegen ist es nicht nur so dahergesagt.

I: sehr schön. jetzt nur noch so ein bisschen auf meinen fokus kurz eingehen, weil mein fokus ja ist, welche auswirkungen die positive bildung auf die professionsentwicklung hat, und *, also, letzten endes, warum sollten lehrkräfte so den blick auf die positive bildung haben?

B: weil dadurch die kinder viel leistungsfähiger sind.

I: hm (zustimmend).

B: das ist einmal ein haupt *, ein hauptpunkt. ich denke mal, wenn ich jemandem sage, was er gut kann, dann will er das noch besser machen. oder ich kann sagen, was er gut kann und wo er auch

noch gut werden kann, ist immer, wie ich es formuliere. dann, wenn jemand sich bewusst ist, dass er sehr viele stärken in sich trägt, dann will er die stärke weitergeben, will er die ausbauen. das ist ganz ein natürlicher prozess, glaube ich. und wenn ich negativ das ganze betrachte oder auch rückmelde, dann wird er immer kleiner und *, und geht immer weniger. also, es *, es sollte schon unser hauptaugemerk sein in schulen, dass die kinder möglichst viel lernen. und ich denke mal, mit *, mit positiver Energie, mit positiven rückmeldungen kann das sehr viel mehr werden.

I: hm (zustimmend).

B: ja.

I: und wenn man von der ebene kind oder schüler wieder auf die lehrkräfte geht, warum *, warum sollte ich als *, als *, als lehrkraft, ja, mich mit der positiven bildung auseinandersetzen?

B: weil es ja mein *, mein hauptaugenmerk oder meine *, meine aufgabe sein sollte, dass die kinder möglichst viel leisten, und deswegen soll ich ja alles daran setzen, die situation, die lernumgebung so zu gestalten, dass die kinder viel leisten können, und das ist eben mit *, mit *, mit positivem feedback, mit *, mit positiven lernrückmeldungen gegeben und sonst nicht.

B: hm (zustimmend).

B: und nebenbei mache ich *, macht es mich als person ja glücklicher und zufriedener, wenn ich positiv zurückmelde, als wie wenn ich immer auf das negative schau. das macht für mich selber auch sehr viel.

I: das stimmt. und das strahlt man ja wieder aus. also, wenn ich mit begeisterung in den unterricht reingehe, weil ich mich darauf freue, mit den kindern mit zu wachsen und *, und die da begleiten zu dürfen in ihrer entwicklung, dann strahle ich was ganz was anderes aus, wie wenn ich dieses, oh, gott, ich muss schon wieder leistungen und noten und so *, habe.

B: das stimmt. ja.

I: okay. gibt's noch was, was du zum *, zum perma.teach noch gerne sagen würdest, was irgendwie noch gar keinen Raum gefunden hat?

B: * ja, ich hoffe, dass irgendwann einmal das perma.teach *. es steht ja schon im lehrplan, im neuen lehrplan ist, wird es ja schon erwähnt. und ich denke mir, mein wunsch wäre, dass das ein fixer bestandteil in der ausbildung sein muss, den *, * was junge lehrpersonen oder angehende lehrpersonen erfahren, erlernen, aber speziell selber erfahren müssen in der ausbildung, um das dann auch so weitergeben zu können. und das sollte viel wichtiger werden als jetzt note und leistung, so das *, ja, das abprüfen des gelernten. ich denke mir, das ist auch wichtig. aber das kann ein computer, das kann eine maschine auch. aber die menschenbildung, und da würde ich jetzt perma.teach dazu *, auf alle Fälle dazu zählen, die menschenbildung, die kann nur von einem pä* pädagogen, von einem

menschen gemacht werden, und das ist unsere stärke und sollte unsere stärke sein, um auch nicht irgendwann wegrationalisiert zu werden durch maschinen. weil, wenn das nur maschinen machen würden, dann haben wir vielleicht leistungsfähige kinder in *, in *, in den verschiedenen fächern, aber keine menschen. und *, und *, und deswegen würde ich mir wünschen, dass das mehr in der ausbildung verankert werden würde und dann auch wirklich in den schulen umgesetzt wird. da könnten wir sicher *. ja, jeder muss in die schule gehen. deswegen, wenn jeder das durchläuft, und wenn es nur die *, nur die vier schulstufen *, die vier grund *, volksschulklassen sind, dann ist ein grundstock gelegt. wenn das darüber hinausgeht, dann in der mittelschule oder gymnasium, umso besser. aber jeder durchläuft diese ersten vier jahre, und wenn *, wenn er da das öfter hört, dann nicht bei jedem, wissen wir eh, aber bei einigen wird da was hängen bleiben. und da könnte sich wirklich gesellschaftsmäßig etwas drehen.

I: ja, auf jeden fall. okay. dir vielen, vielen dank, das ist ganz, ganz viel tolles dabei, was irgendwie so auch noch neue ideen *. das ist ja auch so mein punkt gewesen. also ich *, ich liebe es auch so zu hören, was machen denn andere, weil man *, man kann eigentlich gerade *. so dieser direkte austausch und *, und ideen kann man eigentlich nie genug haben, und das ist immer nochmal was anderes, wenn man das einfach von jemandem hört, wie wenn man es nur irgendwo liest.

B: ja, genau.

I: und auch ganz, ganz viel von dem, was mir so wichtig war, also dieses, ja, ich muss es selber erfahren, also, ich muss selber positiv bestärkt werden und die haltung in mir haben. nur, wenn ich das selber erlebt habe und verstanden habe, dann kann ich es auch wiedergeben, was ja so der kern von perma.teach auch ist. was ja nicht nur in *, in *, bei euch in der volksschule wichtig ist, sondern jetzt eben letzten endes bei uns in der fachakademie auch, weil die sich ja dann eben mit, entweder in der elementarstufe mit denen auseinandersetzt, beziehungsweise denen das weiter gibt, oder, wenn nicht im schulkontext, zumindestens in der nachmittagsbetreuung, et cetera, da eben auch weiter entsprechend wirken zu können.

B: genau. ja.

I: gut. dann würde ich dich zum schluss, so ganz im Sinne von perma.teach bitten, dass du vielleicht noch ein bis drei sachen sagst, für die du dankbar bist, so mit diesem ganzen fortbildungskonzept.

B: ich bin dankbar, dass wir als schule daran teilnehmen dürfen, dass meine sqm mich ganz richtig eingeschätzt hat, indem sie gemeint hat, das wäre doch was für dich.

I: (LÄCHELT) (NICKT)

B: dankbar bin ich für meine kollegen und kolleginnen, die da mitmachen, die versuchen, das umzusetzen, die da dabei sind. dankbar bin ich für euch alle im *, also im hintergrund, die da mitarbeiten, die das entwickelt haben, die das weitergeben, die voller begeisterung den begeisterungsfunken

übersprühen lassen an allen teilnehmerinnen und teilnehmern, die das dann umsetzen. und von euch, dass es immer, auch wenn's oft kleinigkeiten sind, wo wir uns denken, ja, das war jetzt nicht so großartiges, dass dann sehr positiv zurückgemeldet wird, dass das ja genau so etwas ist. und wir tappen selber in die falle, dass man oft meint, nur was ganz großer *, großes, hat einen Wert. aber es sind kleinigkeiten, und *, und das sollte man sich immer mehr bewusst werden, und das wird von eurer seite eben immer wieder bestärkt, und dafür bin ich auch sehr dankbar.

I: sehr schön. dann dir nochmal vielen, vielen dank. du kriegst du auf jeden fall eine kurze rückmeldung, was aus der ganzen studie geworden ist, was so die erkenntnisse daraus auch sind. und wenn du fragen haben solltest, gerade nach dem nachgang zu unserem interview, kannst du jederzeit gerne schreiben. genau.

B: mich würde *, mich würde jetzt noch interessieren, da geht es jetzt darum, ist das jetzt die gleiche studie, wo die kinder befragt werden und auch die lehrpersonen, oder?

I: genau. also, die ergebnisse von den interviews, die ich jetzt mache, die fließen auf jeden fall bei der frau professor doktor lichtinger und beim herrn doktor wammerl mit dabei *, mit dazu. genau.

B: okay.

I: also, die interviewt auch, oder die interviewen auch nochmal, aber die erkenntnisse fließen da auch mit ein. genau.

B: ja. und das ist auch der fragebogen, den die schüler auswerten oder *, oder beantworten? ist das auch in der studie oder ist das wieder eine separate?

I: nee, das ist in der studie mit drinnen. da bin ich aber gar nicht mehr mit drinnen, sondern ich habe quasi nur einen kleinen Teil gemacht, der quasi mit meiner masterarbeit so eine übereinstimmung hat.

B: okay. okay. ja. ja, finde ich *, finde ich toll. und wenn man eine Studie präsentieren kann, dann ist es vielleicht auch für manche personen dann wirklich die erkenntnis, ja, da ist wirklich etwas dran, weil ohne studie ist gleich einmal etwas gesagt und *, und *, und vielleicht ist dann wirklich das nachhaltig, dass das dann mehr umgesetzt wird.

I: ja, das jeden fall. okay. dann dir noch einen schönen nachmittag, hoffentlich mit nicht mehr ganz so lange im büro, dass du noch ein bisschen was von der sonne genießen kannst.

B: ja. ja. ich sag dir auch ganz vielen dank für das nette interview, war ganz eine feine zeit und ganz sympathisch von dir gemacht. ja.

I: danke (LÄCHELT).

B: und wünsche dir viel erfolg mit deiner masterarbeit und mit allem weiteren, was da bei der studie noch zu tun ist.

I: vielen dank. und dir und deiner *, deinem team letzten endes, auch ganz, ganz viel spaß beim weiteren, positiven blick auf die schule.

B: ja. okay. danke. pfiati, (WINKT).

I: tschüss.

B: ade.

Anhang D: Interview 3

30.05.2023 14 Uhr

I: genau sehr gut, dann vielen dank für das einverständnis der aufnahme. dann ähm bin ich natürlich auch ganz neugierig, wer sie sind so als * als person, als lehrkraft, wen ich denn da gegenübersitzen habe?

B: (LACHT) ähm was jetzt genau die schulort oder ? also volksschule. Ich unterrichte in der volksschule. ähm ja seit 23 jahren.

I: mhm (bejahend).

B: habe mein kind gekriegt, da war ich dreivierteljahr zu hause, und bin aber dann gleich wieder eingestiegen.

I: okay. und wie würden sie sich so als als lehrkraft bezeichnen? also was bedeutet für sie lehrkraft sein?

B: für mich heißt lehrkraft, dass man ähm gerne mit kindern zusammenarbeitet, dass man ähm kindern auf augenhöhe begegnet, dass man aus jedem * dass man bei jedem kind seine stärken sieht. es haben nicht alle kinder die gleichen stärken, dass man die kinder begleiten darf. ich glaube, das ist eine * aufgabe, die man sehr zu schätzen wissen muss. ähm ich staune immer, wenn man so an dem ersten schultag, so die einzelnen klassen, dann hat man das immer ganz fest im kopf, und wenn man dann so die ersten buchstaben lernt und in der vierten, wenn man dann sieht, was sie daraus entwickelt, welche aufsätze die kinder dann schreiben, gell. wenn es teilweise die ganz guten sogar über 350 wörter, teilweise mit einem wortschatz schreiben, wie man das selber oft mal nicht hinkriegen würde, ja dann weiß man einfach, dass man schöne arbeit leisten hat dürfen.

I: sehr schön. ja, das freut mich, das passt ja schon sehr gut zu dieser ähm positiven bildung ähm mit dazu. da schwingt ja schon ganz, ganz viel mit * rein. wie sind sie denn ähm auf die fortbildung aufmerksam geworden?

B: auf perma.teach?

I: mhm (bejahend).

B: das war eigentlich bei uns als schulprojekt gedacht, und auch natürlich ähm für die * für das qualitätsmanagement, * genau.

I: haben sie davor schon * schon kontakt zur positiven psychologie oder positiven bildung gehabt?

B: ähm eher in einem * in einem anderen Projekt * eigentlich eigenständig werden * heißt das.

I: mhm (bejahend).

B: ähm genau, da haben wir mal eine fortbildung gemacht über das, und es waren immer wieder mal so einzelne fortbildungen, wo es um * ja, um das gegangen ist, dass man einfach positiv in das ganze hineingehen soll oder schüler stärken und so weiter. da waren immer wieder mal so fortbildungsangebote bei uns, über jahre. also genau.

I: das heißt, was hat sie denn bei der * bei dem perma.teach besonders angesprochen?

B: ähm bei dem perma.teach besonders angesprochen, hat es mich dann eher im privaten bereich, weil ich ja letztes jahr keine klasse gehabt habe. ähm was ich total gut gefunden habe, war dieses am abend die positiven gedanken aufschreiben. ich habe jetzt einen sohn, der ist viereinhalb jahre alt und auch mit dem nochmal den tag einfach revue passieren lassen, und wirklich einfach das positive herauszuheben, dass er dann gut einschläft. ähm, was ich auch sehr gut gefunden habe im privaten bereich, jetzt kann ich nur sprechen, ja? einfach ähm negative sit* oder situationen, die einem im ersten moment vielleicht negativ erscheinen, dass man die dann einfach in was positives ummünzen kann. und ähm das habe ich einfach ganz super gefunden, weil immer, wenn ich jetzt eine Situation kriege, wo ich mir denke: * wie gibt's denn das ist jetzt? * und auf einmal fällt einem ein, eigentlich ist das was positives, genau.

I: das ist ja das schöne,

B: das sind jetzt die sachen, die mir so einfallen

I: mhm (bejahend). ja auf jeden fall das ist ja das schöne, dass man einfach nochmal eine andere perspektive auch auf die * auf die situationen bekommt.

B: genau, und das hat mir eigentlich auch im privaten bereich sehr weiter geholfen.

I: mhm (bejahend). das ist ja auch letzten endes ein großer bestandteil vom perma.teach, dass man ja auch viel selber erleben ähm darf, um es dann letzten endes auch ähm andere ähm weiterzugeben.

B: ja.

I: * ähm wie * hm (nachdenklich) muss immer so ein bisschen auf meinen * auf meinen Spickzettel schauen, damit ich mich so inhaltlich ein bisschen ähm fokussieren kann. ähm wenn man vielleicht nochmal auf dieses fortbildungskonzept ähm schauen. was ähm * * gibt es was, wo sie sagen, das war besonders gut? sie haben gerade schon ein bisschen die perma.post angesprochen. gibt es noch etwas, was sie von diesem konzept jetzt ähm rein als gut bewerten oder vielleicht * veränderungswürdig?

B: gut habe ich einfach gefunden, diese ähm kleinen arbeitsaufträge, was man selber machen hat müssen. dass man ja dieses * dieses heft eben angelegt hat und gedanken gemacht hat. ähm genau. ähm dann die einzelnen videos habe ich auch sehr gut gefunden. mhm (bejahend). das ist von

knietzsche hat mir einmal gut gefallen. dann wären teilweise andere videos, einer habe ich selber oft kurz angeschaut. ähm man merkt sich leider nicht alles, weil es ziemlich viel ist, muss ich sagen und ich schaue gerne wieder mal ins heft, ab und zu und dann auf einmal, immer wieder *ah ja, jetzt kann ich mich wieder erinnern*, also das ist ganz hilfreich, dass man einfach dieses heft wieder hat zum kurz reinschauen und dass sagt: *ah genau so war das.* also, es bleibt dann schon viel. ähm wo man sich dann erinnert und sich denkt: *ah ja, jetzt weiß ich wieder, was ich umsetzen muss.* oder man erinnert sich: *ah, da war was in dem heft*, man schaut kurz rein. genau.

I: mhm (bejahend).

B: ja.

I: können sie ähm vielleicht irgend so eine situation erzählen, wo sie tatsächlich nochmal auf was zurückgreifen konnten oder was ihnen tatsächlich auch so ähm weitergeholfen hat oder sie neu inspiriert hat?

B: ja muss ich jetzt auch schwindeln, weil ich brauche jetzt das heft, dass ich ein bisschen nachschauen kann.

I: natürlich.

B: weil ich weiß das nicht mehr auswendig.

I: ja, okay, gut. es geht nicht ums abfragen.

B: (LACHT) okay.

I: sondern es geht eigentlich wirklich darum, von ihnen rauszuhören: *mensch, was nehme ich mit, was habe ich mitgenommen? was hat mich begeistert?* also eher so wirklich dieses erzählen, und ähm genau. um das geht es letzten endes.

B: ähm was ich auch gut gefunden habe, war einfach diese * dieses anreize kriegen, dass man selber wieder neue sachen macht, dass man über seine grenzen drüber geht. den anreiz habe ich ganz gut gefunden. ähm dann ähm mit freundschaft, liebe und gefühle auf die Kinder einfach zugehen. ähm was ich auch sehr gut gefunden habe, war das ähm * selber seine stärken einmal aufschreiben * und kennenlernen, ähm, dann war es noch * was ein ganz großer punkt für mich war auch * immer, wenn man dankbarkeit ist. ich habe gemerkt, wenn man für viele sachen dankbar ist, dass man, glaub ich, dann einfach sein leben viel glücklicher gestalten kann. wenn man einfach so kleinigkeiten sieht, wo man einfach dankbar ist, und ähm genau. *3Ü* ja. *3Ü* was mir auch sehr gut gefallen hat, das ist mir gut hängen geblieben war der trommler, der klint pulver (?).

I: mhm (bejahend).

B: weil ich habe so einen ähnlichen schüler in der klasse, und ähm man kann dann viel besser umgehen. also, der hatte mit seinen stiften immer getrommelt, und der alte lehrer hat ihm dann diese

drumsticks gegeben, also das ist so ein video, was bei mir * was mich sehr geprägt hat. also, das video habe ich ganz super gefunden, habe es auch geteilt dann. (LACHT) ja, genau.

I: sehr schön. wollen sie mich in irgend so eine situation noch mal ein bisschen mehr mitnehmen? Jetzt haben sie ja von dem * von dem jungen schon erzählt, wie sie da einfach anreize hernehmen konnten und dann was für sich Positives entwickeln konnten.

B: * ähm positive Situationen aus der schule oder? ich weiß, jetzt * jetzt * oder?

I: völlig egal. also, sie haben ja selber schon geschrieben, dass sie jetzt bei der umsetzung in der schule nur in ansätzen was erzählen können. deswegen also gerne, auch wenn es alltagssachen sind, wo sie sagen: *mensch, da habe ich was umsetzen können, oder das hat mich inspiriert.* das darf es auch sehr gerne sein.

B: ich weiß jetzt nicht, ob das jetzt genau passt. ich habe jetzt gerade mit meiner ersten klasse 20-jähriges treffen gehabt.

I: mhm (bejahend).

B: und das war damals eine sehr große klasse von 29 kinder, ein schwerst behindertes kind und zwei einzelkinder. und das ähm ja, waren ihre eigenen charaktere eigentlich und ähm was da * oder ich habe da meist vom sozialsprengel eine helferin gehabt, und wir haben dann, bevor das treffen mal stattgefunden hat, viel miteinander telefoniert. dann haben wir dann eigentlich ganz viel erinnerungen sind dann wieder aufgetaucht, und auch positive erinnerungen waren am schwierigen kind. und ähm ja, der hat sich für gar nichts interessiert, aber was der super gewusst hat ähm auf dem Bauernhof. wie das läuft, und der hat uns damals eine melkmaschine an die tafel in der ersten klasse aufgezeichnet, und das wissen wir heute noch. also, die gnaze tafel hat er aufgemalen und er hat uns genau erklären können, wo die flüssigkeit rein kommt, was da dazu gemengt, in welcher * also von den mililiter her, was dazu gemengt wird, wie viel druck dann das eingeschalten werden muss, dass das dann weiter wandert in die nächste kammer, und von der nächsten * also der hat uns * ich kann es nicht mehr. er hat es genau gewusst, und von dem tag an, hat man eigentlich gewusst, um das kind braucht man sich keine sorgen machen, der macht seinen weg, und der ist dann auch seinen weg gegangen. und das war eigentlich auch, dass man gewusst hat, das schulische ist das eine, da muss man jetzt einfach durch, aber man weiß, da ist irgendwie so der knopf aufgegangen: *ich muss jetzt nicht stressen, weil der macht das.* und jetzt nach 20 jahren * also bei dem, dem geht es so gut, und der hat seinen weg gemacht, und das war total schön.

I: sehr schön.

B: ich weiß jetzt aber nicht, ob das jetzt so das war. also was sie jetzt brauchen.

I: doch auf jeden Fall, weil das ja letzten endes * es ist zwar jetzt nicht direkt verknüpft, also damals nicht so verknüpft gewesen mit der positiven bildung, aber letzten endes spricht da ja ganz, ganz

viel für diese * dieser blick auf positives und diese ressourcen, um stärken zu erkennen, und da ist ja * da schwingt ja viel mit, auch die positiven emotionen, also auch * auch die * die zuversicht letzten endes, dass ähm der junge seinen Weg machen wird, und das ist ja als * als lehrkraft was ganz, ganz wichtiges, wie sie ja gerade selber schon beschrieben haben.

B: genau. erst dann kann ich sagen

I: (LACHT) ja, ressourcen früh entdeckt und dann der eigenen leidenschaft gefolgt. und es ist ja schön, wenn man dann tatsächlich auch nach 20 jahren ähm das auch nochmal so mitbekommen darf, dass die vermutung oder die * die * die zuversicht, die man damals hatte, dass aus dem was wird, dass man dann auch irgendwo die bestätigung bekommt. und ich glaube, das ist ganz, ganz wichtig, dass man auch immer wieder so die * die * die ergebnisse so ein bisschen auch wieder mitbekommt.

B: mhm (bejahend). ja.

I: sehr schön. ich finde, das ist ein sehr, sehr schönes beispiel. das bedeutet aber, dass ihnen das schon immer ein anliegen war, auf die kinder zu schauen und ressourcenorientiert zu arbeiten, oder?

B: ja. mhm (bejahend).

I: können sie denn da noch beispiele erzählen? die müssen jetzt nicht in den letzten ein bis drei jahren ähm gewesen sein, aber wo sie sagen: *so rückwirkend betrachtet, ist es für mich schon so richtung perma.teach, richtung positive bildung.

B: mhm (nachdenklich). ich glaube, positive bildung ist alles was man oft mal außer, also was im schulischen bereich eh ist, aber was man zusätzlich oftmal macht. also, wenn man mit klassen zum beispiel weihnachten oder theater einstudiert und das vor die eltern aufführt, oder wenn man mit den kindern gewisse sportliche aktivitäten macht, ob man jetzt eislaufen, schwimmen, wenn man solche sachen macht, das prägt einfach eine klasse. und das fügt das ganze sozialgefüge verbessert es einfach auch. oder wenn man eine lesenacht macht und ich glaube, wenn man da sehr engagiert ist und bereit ist, mit den kindern andere sachen zu machen, außerhalb von diesem schulischen bereich, dann bewirkt das ganz viel. oder dass man wirklich sagt, für die kinder mathe und deutsch ist das eine, aber es gibt noch andere wichtige fächer, ähm wie werkerziehung, weil da war sie, ja da waren zwei jungen die waren * also, eher die haben sich sehr schwer getan in diesen sogenannten hauptfächern. wir haben damals in der vierten Klasse einen stromkreis gemacht, und ähm wir haben dann so eine werkbank gehabt, wo die anleitung dabei war, und die zwei jungen haben ohne, dass es von mir erklärt wurde, ofort die beschreibung geschnappt, sind in einer ecke verschwunden und haben das innerhalb von einer dreiviertel stunde alleine zusammen gebaut. also, die haben genau gewusst, wo sie es abseilen müssen, wo das auch blinken muss, wo sie es messen müssen, die haben das alles ohne probleme ausgelesen. und ich glaube, das war * und das haben sie mir auch

dann nochmal gesagt, wie wichtig das war, dass ich gesehen habe, dass eine stärke in dem bereich liegt. ja.

I: mhm (bejahend).

B: und ich glaube, jedes kind, jeder mensch hat seine stärken. auf diese müssen wir wirklich schauen, und ich glaube, dann werden sie halt auch die schullaufbahn, gut durchtragen und ich glaube, auf das können sie dann für das spätere leben einfach aufbauen, die kinder.

I: was meinen sie, braucht es in unserem schulsystem, dass man mehr auf die kinder schauen kann, so stärkenorientiert wie sie es jetzt schon dargestellt haben?

B: ähm 3Ü ich glaube, das muss irgendwas * ist schwierig. Es ist eine ganz schwierige frage jetzt. ich glaube, dass man schon irgendwo schafft, dass eigentlich alle fächer gleichwertig sind. also das nicht immer dieser schwerpunkt, deutsch, mathe, sachunterricht ist. und ich weiß nicht, ob diese ganze entwicklung, was jetzt so die letzten * vor allem die letzten zehn jahre passiert ist, ob das immer zielführend ist, weil von den kindern wird immer mehr verlangt, ähm sie müssten so viele testungen durchmachen. also, ähm wir haben damals so * ähm haben wir so einen lesetest gehabt, ich glaube * ähm ich weiß nicht mehr, pearl (?), ich glaubt hat das geheißen. dann diese bildungs-standards, dann dieses känguru oder mathematik. es ist * es ist ganz viel, was da von den kindern abverlangt wird. und ich weiß nicht, ob das zielführend ist, weil es setzt uns lehrer sehr unter druck, und wir geben den druck automatisch an die kinder weiter.

I: mhm (bejahend).

B: und ich muss sagen, es war die ersten jahre eigentlich leichter, und nachdem man eben weiß, dass wenn jetzt bei kindern wenigstens auf die stärken geschaut wird, es sich super entwickeln kann, dann weiß ich nicht, ob das sinnvoll ist. also ich bin mit der entwicklung, jetzt nicht so begeistert.

I: ja. absolut verständlich, wenn es einfach immer mehr wird und man quasi für alles, was nebenher ähm stattfinden könnte, was ja dann irgendwie wie sie schon gesagt haben, letzten endes die kinder nochmal viel mehr stärkt, wenn man einfach fernab vom reinen sach- und fachunterricht ähm * ähm arbeitet, dass dafür immer weniger platz ist. wie haben sie es *

B: ja. mhm (bejahend).

I: gerne

B: nein, passt.

I: wie haben sie es denn in den letzten jahren trotzdem geschafft, ähm trotz des zusätzlichen ähm leistungsdruck, der ja leider gottes doch immer wieder da ist, so positive momente für die schüler und schülerinnen zu schaffen?

B: ich mein es ist ja ein brutaler spagat, den man dahin legen muss, und jetzt ist ähm * muss jetzt sagen, ähm, man wird innerlich fertig gemacht durch das ganze system. und man muss sich echt selber, die freiheit behalten, und man muss selber ähm sagen, ich geht trotzdem mit freiheit ein und mir sind die kinder ganz wichtig. und das muss man sich jeden tag vor augen halten, ne? weil sonst geht man echt an dem ganzen ein bisschen kaputt, und man muss sagen, ich sehe dass die kinder einfach, der ist in werken super, der ist in turnen super, und das muss man den kindern, glaub ich, weitergeben. ihnen sagen man darf * , man wird also einfach sagen, wie toll du da jetzt rauf geklettert bist, man muss es wirklich verbalisieren, sie meistens wahrnehmen, man müsste wirklich einer in die augen schauen, gleich so nebenbei einer die sagen. ähm es ist ein totaler spagat und ich weiß nicht, ob der spagat in zukunft noch so möglich ist. also, ja.

I: #haben sie den eindruck *

B: also ich kenne lehrer, die bis ins burnout getreten sind. # ist jetzt meine * oder wo man einfach sagt, was da jetzt abverlangt wird, das ist * es ist zu viel.

I: mhm (bejahend). haben sie den eindruck, dass perma.teach das ja jetzt letzten endes als * als ähm pilotprojekt startet, aber schon der gedanke ist, dass man das ähm in allen schulen irgendwann mal etabliert, wenn es auch entsprechend ähm wirkungen hat. dass das eine zusätzliche belastung sein könnte, weil es noch etwas ist, was man macht, oder eher eine entlastung und eine bereicherung?

B: perma.teach?

I: mhm (bejahend).

B: ähm perma.teach ist eine bereicherung, auf jeden fall. ähm aber wie gesagt man merkt halt in den anderen bereiche einfach, glaube ich, wieder ein wenig, ähm weniger machen.

I: mhm (bejahend).

B: also ich würde jetzt nur sagen, damals die bücher, da waren weniger seiten * ähm viel weniger seiten, und ähm ich habe es dann bei jahresplanungen danach gemerkt, und es ist einfach eingestuft, eingestuft und wir haben aber nicht mehr unterrichtszeit, und wir sollen dieses alles durchbringen, weil eben dann diese bildungsstandardstestungen auf das aufgebaut sind, und ich finde früher, hat man einfach ähm durch das, dass man weniger seiten gebraucht hat, viel mehr zeit für spiele * also für lernspiele gehabt, weil die kinder dann zum beispiel total gerne so spiele, wo sie auch bewegung dabei haben. da gibt es zum beispiel ecken rechnen oder raupen rechnen, also da gibt es die verschiedensten mathematikspiele. und jetzt, wo man das zwanzigjährige treffen, gehabt haben, die kinder haben sich an das erinnert, was einfach * und da haben sie * einfach diese spiele haben so viel * oder bingo, und man hat jetzt oft auch, gar nicht mehr die zeit, dass man auf das wert, gell?

I: mhm (bejahend).

B: und ich finde, perma ist da sehr bereichernd, wenn man sich wieder zurück erinnert, was das wesentliche sein sollte, aber es müsste in einem anderen bereich einfach komplett wieder runtergefahren werden, und ich glaube, dass die kinder von damals, also von der klasse, von den 29 ähm, da haben wir dann in der vierten 26 gehabt, weil drei weggezogen sind, von den 26, war einer schwerstbehindert, zwei aso-kinder. also, wenn ich die jetzt wegtue, dann sind wir bei 23 kinder, und 18 haben maturiert, gell. da muss ich einfach sagen * also das ist doch einfach so, wie man früher gearbeitet hat, glaub ich, was nicht schlechter war, man hat aber weniger den druck gehabt. es würde ausreichen, und die kinder sind echt tip top drauf.

I: ja, schade, dass wir an den * an den strukturen hier jetzt leider gar nichts ändern können, also noch nicht. das ist, was, was leider in dem prozess hoffentlich irgendwann wieder eher richtung entschleunigung und qualität statt ähm quantität ähm letzten Endes wieder dahin gehen muss.

B: genau aber, wie gesagt, es hilft dann wieder, dass man das bewusst ist und dass man da einfach wieder: *okay, es war schon mal so, und schau wieder auf das, weil das ist für die Kinder wichtig.*

I: mhm (bejahend). was würden sie sagen, welche wirkung hat * hat perma.teach oder generell die positive bildung auf auch die einzelnen schüler?

B: *3Ü* ja, ich glaube, dass sie einfach dann jeden tag, mit einem guten gefühl in die schule gehen, dass sie gerne in die schule gehen, dass sie ähm * ich glaube, einfach es wirkt auf diese intrinsische motivation einfach von den kindern gar nicht aus, wenn man positiv eingeht. und ähm ja.

I: mhm (bejahend).

I: ja, und ich glaube, die schule ist ein raum, der sich positiv auf ihr leben auswirkt. ja, also deshalb einfach, ja mit der freude reingehen und mit der freude auch wieder nach hause und das ist eigentlich wie es sein sollte, ja?

I: ja, dass schule auch wieder ein ort ist, an dem man wachsen darf, nicht nur muss und ähm von einem test zum anderen, sondern wo man sich entfalten darf und wo man, wo man sich letzten endes auch neu entdecken kann. also, das wäre schön, wenn schule auch wieder zu dem ort werden würden.

B: mhm (bejahend). ja genau.

I: wenn man jetzt die schulfamilie, also * also nicht nur die schüler anschaut, sondern auch die * die * die lehrkräfte mit dazu. was würden sie sagen? welches potenzial steckt aus ihrer sicht in dem fortbildungskonzept perma.teach?

B: ähm, 3Ü ja, ich glaube, es ist einfach auch, dass man ähm sich gegenseitig wieder diese positiven sachen erzählen muss. was passiert ähm im schulalltag, wen man in der pause zusammensteht, dass man mal fragt: *okay, was hat es denn bei dir heute lustiges gegeben oder * oder was habt ihr

heute super gemacht?* und so, dass es sich halt positiv auswirkt, dass man einfach diesen positiven austausch ähm versucht. mehr positives ähm sich gegenseitig zu erzählen, und das hebt einfach die stimmung. ja.

I: mhm (bejahend). ja, das stimmt, weil erfahrungsgemäß ist im lehrerzimmer doch auch immer nochmal dieses thema: *oh gott, was war in der letzten stunde?* oder ähm es werden einzelne schüler nochmal besprochen, eher so richtung, was könnte man noch machen, aber doch auch häufig defizit orientiert beziehungsweise versuchend auszugleichen und weniger auf dieses *mensch, was habe ich eigentlich an schönen erlebnissen?*

B: genau oder was wir jetzt schon mal machen, dass wir uns gegenseitig in die klassen besuchen.

I: mhm (bejahend).

B: also jetzt nicht bei jedem, aber bei einigen, und dass man oft einmal so schaut und sagt: *schau mal, das ist jetzt aber nicht, was du da in der klasse drinnen hast.* oder *na? was habt ihr jetzt gerade gemacht?* und man schaut sich ein wenig um. einfach da wirklich in die Klassenräume, nicht nur unten im konferenzzimmer trifft, sondern wirklich mal die kollegin in der klasse besucht und kurz einen besuch macht, und das ziehen wir eigentlich ganz gut durch.

I: mhm (bejahend). sie sagen, das hat * hat ihnen gut getan, also ihnen selbst und natürlich auch den kolleginnen. was würden sie sagen, was macht denn das mit dem * mit einem team, auch wenn es sich gegenseitig besuchen kann und auch positive rückmeldungen gibt?

B: es stärkt einfach oder man ist öfters mal so ähm * ich glaube, manchmal fühlt man sich so ein bisschen alleine gelassen in dem ganzen system, und dann merkt man einfach mal * oder ich schaue gerne in die klasse, und die arbeit wird nicht so geschätzt

I: mhm (bejahend).

B: man schätzt es einfach, genau. die wertschätzung wird einfach angehoben, und genau. * ja, also man hat das gefühl * und nicht nur ich alleine, ich sehe auch was in meiner klasse passiert, wenn jemand anderes da ist, das bewirkt viel.

I: mhm (bejahend). gibt es da irgend noch eine * eine situation oder irgendein beispiel, wo sie das nochmal konkretisieren können? diese wirkung auf * auf * auf sie selber oder auf das team, auf das kollegium?

B: 3Ü ja, eben, wie gesagt, diese * diese * diese besuche mit der kollegin machen wir jetzt jeden donnerstag, dass man am donnerstag kurz zusammenstehen, weil am freitag bin ich nicht beim unterricht. dann sagen wir uns am donnerstag gegenseitig: *hey, erzählt du mir das positive erzählt du mir.* und entweder trifft man sich bei ihr in der klasse oder bei mir in der klasse.

I: mhm (bejahend).

B: genau. also ist so fix eingeführt worden, würde ich jetzt sagen.

I: sehr schön, das ist dann vielleicht auch was, worauf man sich dann auch schon freuen kann, so zum ende der woche hin.

B: ja (LACHT).

I: sehr schön. was würden sie sagen, ähm wie hat sich die * die * ja dieser blick wieder auch auf die positive bildung oder mit diesem perma.teach, wie hat sich das auf ihre lehrerpersönlichkeit, ausgewirkt? gab es da was * was sie bei sich nochmal entdeckt haben oder wiederentdeckt haben?

B: hm (nachdenklich). *3Ü* na, weil ich einfach immer mit einer freude reingegangen bin. ich hab mir immer das positive, glaube ich bewahren können. zum glück. und ähm ja, es war eigentlich eher dann so, man macht es eh, man * man ist nicht ganz so falsch, wenn man das so macht, ja dass man auf die kinder schaut, ähm und genau.

I: also eher eine * eine bestätigung dessen, dass so wie sie vorher gearbeitet haben, dass das jetzt vielleicht nochmal den anderen namen bekommen hat, und eine bestätigung, dass es * dass es * dass man das vorher vielleicht oft intuitiv gemacht hat, dass das auch * ähm ja auch noch mal einem konzept auch entsprechen kann.

B: genau, ja.

I: sehr schön. das ist ja auch das, was sie eingangs, wo ich dann gefragt habe, wie sie sich als lehrkraft einfach sehen. das war so ein bisschen der hintergedanke der frage, um rauszuhören, was bedeutet denn für sie, als * als lehrerin oder als lehrkraft vorne zu stehen, was macht das für sie aus? und dann haben sie ja schon gesagt, dass dieses kinder ähm entwickeln sehen, entfalten sehen, dass ihnen das ganz, ganz wichtig ist und dass ihnen das, so wie ich jetzt rausgehört habe, immer schon ein anliegen war, nicht auf irgendwelche leistungen hinzutrimmen, sondern einfach diesen prozess auch zu sehen und sich mit den kindern einfach zu freuen, und das fand ich auch ein schönes beispiel, mit diesen aufsätzen vom lernen der ersten buchstaben hin bis zu aufsätzen, die dann einfach wirklich bewundernswert sind. und dass ihnen das wichtig ist, das den kindern auch so auch rückzumelden.

B: mhm (bejahend).

I: kann ich sehr gut nachvollziehen. bei mir sind zwar keine kinder, aber bei mir sind dann die zukünftigen erzieher und erzieherinnen, und ich kriege die bei uns im dritten ausbildungsjahr. da sind sie zwei jahre bei mir im hauptstudium, also überwiegend unterricht, und das letzte jahr ist bei uns ein praxisjahr, und das ist auch immer schön zu sehen, wie die * wie die sich einfach in diesen drei jahren entfalten, auch vielleicht gerade so am anfang noch * noch ganz schüchtern und durch immer wieder rückmeldung und bestätigung nach dem praktika oder so. da hatte ich dann auch schon welche, die am anfang vom letzten jahr noch recht schüchtern waren und die dann am ende wirklich

in einer gruppe drin standen, richtig souverän und gestärkt, wo du wirklich merkst, spürst, die sind um einige zentimeter gewachsen, und ich liebe es, wenn ich dann auch sehe, wie die das leuchten in den augen haben und einfach mit einer absoluten begeisterung dann mit kindern oder jugendlichen arbeiten, wo man dann wirklich merkt, es ist nicht nur ein job, einen beruf, den ich machen muss, weil ich geld verdienen muss, sondern wo wirklich der job ähm als * ja mit herzblut auch erfüllt wird. und das ist das, wo ich mich dann immer freue, wenn ich dann ganz am schluss die praktische prüfung abnehme, wo mir dann auch das herz aufgeht, wenn ich einfach merke, ja, das sind jetzt erzieher und erzieherinnen, die genau, wie sie es jetzt selber geschildert haben, auch ihre kinder dann wieder weiterentwickeln wollen und denen einfach ganz, ganz, ganz viel mitgeben wollen und da mitfiebern und * und mit begeistert sind.

B: ja. ja, verschiedene aufgaben.

I: ja, absolut. die aber auch nur geht, aus meiner sicht, wenn man tatsächlich da eine * eine leidenschaft dafür entwickelt. oder wie sehen sie es?

B: ja schon.

I: und das * das perma *

B: man hat auch das glück, wenn man sich auf die kinder ein wenig einlässt, dass einen die kinder da brutal mitreißen können.

I: ja.

B: oder wenn man die Ideen der kinder zulässt, dann entwickeln sich da ganz tolle sachen draus.

I: ja, was ja auch jetzt mit den ganz vielen kleinen impulsen auch immer wieder möglich ist, und die kinder ja auch ganz viel mitnehmen ähm an * an positivem. also auch sich gegenseitig dann auch mal positives rückmelden, wenn sie das von uns eigentlich vorgelebt bekommen.

B: mhm (bejahend).

I: wie ist ihr eindruck, wie haben die Kinder auch, ja auf dieses perma.teach reagiert? also, ähm sie haben ja dann irgendwann auch angefangen, mehr impulse in die richtung einzubauen. also auch nicht * wenn sie nicht eine eigene klasse haben, aber irgendwo im unterricht gab es da eine veränderung bei den kindern?

B: ich habe es jetzt wenig einbauen können, weil letztes jahr ich ähm fast nur werkstunden gehabt habe und eben vereinzelte stunden mit kindern, so zettelstunden, sagt man da bei uns. da kann ich jetzt nicht so eine detaillierte antwort geben. jetzt nur von der online klasse, glaube ich, ist es auch einfach gut die kinder * ja einige klassen, haben die echt gut durchgearbeitet mit den kindern. ich glaube, da haben wir einfach gemerkt, dass man viel mehr sozial arbeitet, dass das schon die gruppe wieder ganz anders zusammenschweißt. ja.

I: mhm (bejahend). ja, das stimmt. dann brauche ich noch mal meinen zettel. ähm gibt es denn was, wo sie jetzt sagen, zum fortbildungskonzept? da möchte ich unbedingt nochmal was zurückmelden.

B: positiv?

I: positiv, aber auch anregungen für veränderungen. das braucht es ja auch ab und zu.

B: *3Ü* ja, ich habe es gut gefunden alles, aber ich muss sagen, es geht sehr viel zeit darauf, gell. also ich war damals echt froh, dass ich nicht voll beschäftigt bin, ähm und da muss ich sagen, es war schon sehr zeitintensiv, und ich war dann teilweise immer ein bisschen hinten mit dieser perma.post, und das dann wieder aufzuholen. es ist sehr * sehr zeitintensiv, das ganze programm.

I: mhm (bejahend).

B: ja. also, man muss sich genau damit beschäftigen, und es ist ähm * man kriegt so viel * ähm so viele anreize, und das war das gute. also, ich hab es gerne gemacht, muss ich jetzt eigentlich sagen, also, ich habe gerne, in das heft angelegt, man schnauft zwar wenn man mal zehn perma.post hinten ist, also weil jetzt doch * also manche videos haben sehr lang gedauert, ähm aber wie gesagt, es ist dann * wenn man es dann durchgearbeitet hat, denkt man sich schon: *das war es jetzt wert* aber es ist zeitintensiv.

I: mhm (bejahend). mhm (bejahend). aber gerade, weil sie es jetzt gesagt haben, bei der perma.post wäre da vielleicht ein größerer abstand auch noch mal eine option? was meinen sie?

B: ja, wäre fein.

I: dass man auch wirklich die möglichkeit hat, das im alltag einzubauen, und nicht den eindruck hat, man hat jetzt so viel geballt, ähm, was man nachholen muss, weil ja immer schon so ein bisschen sein gewissen mit reinschwingt oder ein schlechtes gewissen, dass man da ja noch was nachholen muss. und das soll ja nicht sein.

B: ja. mhm (bejahend).

I: mhm (bejahend). wenn sie jetzt einer kollegin oder einem kollegen von perma.teach erzählen würden, was wären ihre hauptargumente, an so einem * an so einer fortbildung teilzunehmen?

B: hauptargumente waren einfach wirklich, dass man wieder so positiv gestimmt wird. das heißt also, diese fünf punkte, dann wichtig sind, was dann eben einen anspricht, ähm und eben an diesen fünf Punkten zu arbeiten, glaub ich, das ist ganz wichtig, und man vergisst es oft in seinem alltagsstress und so, und dass man da das wirklich wieder im bewusstsein hat. diese positiven emotionen sind total wichtig, diese freundschaft oder im kollegium, eben diese * also dieses team, was zusammenarbeitet, ist so wichtig, dass man doch wieder die zeit nimmt, dass man sagt: *okay. machen wir das gemeinsam.* ähm ja, was auch ein wichtiger punkt ist, dass man es ja wieder ähm ja, dieses meaning war für mich auch ein punkt wieder herzugehen: *hey, schauen noch mal, was du jetzt bewirkst,

was das positives ist, was du leistest, und ähm dieses ziele setzen, ja das ist ein ganz wichtiger punkt gewesen, den bringt man oft mal so gerne, dass man sagt: *hey, das ist jetzt mein ziel, und das sind kleine schritte, und über die man sich dann auch wirklich freuen kann*

I: mhm (bejahend).

B: eben wie gesagt, es ist einfach gut diese ganzen sachen, sich bewusst zu werden. ja.

I: ein ziel setzen.

B: wieder normen, also man weiß, das gibt es, und wenn man diese fünf punkte in diesem ausmaß zu arbeiten, das war eben so gut, ja.

I: mhm (bejahend). und gerade bei dem ziele setzen, wenn man sie erreicht, sie auch tatsächlich zu feiern, also auch zu würdigen und nicht zum nächsten tagesordnungspunkt überzugehen.

B: ja genau. ja.

I: was ja gerade wenn man so einen vollen lehrplan hat und ganz, ganz viel, was man im unterricht unterbringen muss, dann geht es manchmal unter, auch ähm in der arbeit mit den kindern, dass man wirklich auch sich zeit nimmt, um etwas, was man geschafft hat, auch als solches zu würdigen und sich mit zu freuen und nicht zu sagen: *okay, geschafft*, und das nächste steht schon wieder an.

B: genau, ja.

I: mhm (bejahend). ähm gibt es was, was sie sich noch gewünscht hätten, so im rahmen vom fortbildungskonzept, was ihnen noch geholfen hätte?

B: 3Ü was ich mir gewünscht hätte, dass nicht so viel online ist. ich war mehr oft bei dieser * bei diesem * man arbeiter zwar die perma.post durch, ich finde manchmal wäre es schön gewesen um mehr ähm * noch die einzelnen punkte klar macht, dass man sich eher wirklich noch getroffen hätte und das besprechen hätte können, gell. dass man einfach dann mit der anleitung, glaube ich, oder gewisse sachen reflektieren hätte können, gell. weil das ist ein bisschen abgegangen. man hat es für sich alleine durchgearbeitet und das reflektieren ist oft einmal abgegangen.

I: mhm (bejahend). ja. hat bei ihnen in der Schule noch *

B: wenn man nicht selber aktiv geworden ist, dass man jetzt mit kolleginnen sitzt * zusammen gesetzt hat, da hat mir ein bisschen die anleitung einfach noch gefehlt.

I: mhm (bejahend).

B: ist mir fast ein bisschen wirklich zu kurz ausgefallen.

I: mhm (bejahend).

B: also, da war wirklich eher in Präsenz, dass man öfters zusammenkäme wünschenswert gewesen. ja.

I: ja, verständlich, was ja die positiven effekte nochmal verstärkt, wenn man sich wirklich gezielt noch mal austauschen kann.

B: genau. oder da mir dann auch * ich finde, da ist die rückmeldung einfach zu wenig gewesen dann. also, das man einfach sagt * ja.

I: mhm (bejahend). also letzten endes beispiele vielleicht auch bringen können und auch nochmal ähm sich anzuhören, was die * was die anderen dazu sagen oder welche ideen sich daraus wieder entwickeln könnten.

B: genau, ja.

I: der austausch bei ihnen an der schule, inwiefern konnte der stattfinden, oder haben da noch andere teilgenommen?

B: ähm ja, es haben ja viele schulen teilgenommen.

I: aber aus ihrer schule, aus ihrem direkten kollegium haben sie sich da #mit anderen *

B: das ganze kollegeium.# das ganze kollegium hat teilgenommen. ja. mhm (bejahend).

I: und wie hat da der austausch stattgefunden?

B: *3Ü* ja, mit dem gewissen halt, also mit ähm * mit * wie es halt zeitlich möglich war, ja. genau, weil ich bin jetzt nur an drei tagen, also zum beispiel mit einer kollegin komme ich mich gar nicht zusammen, weil die die anderen zwei, ja, es ist halt so, gell. und dann hat sie es eben ja. hast du jetzt so mit fünf, sechs einen austausch, aber wir sind ein kollegium von über 20 leute, dass man da jetzt mal fast wieder jemanden braucht der sagt: *okay, heute setzen wir uns alle nochmal zusammen und reden über dieses ganze.* also so einer, der vielleicht die Gruppe, ein bisschen anleitet und zusammenhält. genau. das ist mir ein bisschen abgehangen.

I: mhm (bejahend)

B: ja.

I: das heißt, wäre das vielleicht auch was gewesen, was man an so * bei uns heißt es pädagogische tage, weiß nicht, wie es in österreich heißt.

B: ja, genau. mhm (bejahend).

I: dass man da vielleicht nochmal den fokus drauf legt.

B: ja. mhm (bejahend).

I: hat bei ihnen vielleicht nicht so den raum gehabt, wäre aber schön gewesen.

I: ja, da ist wieder jeder in diesem druck drinnen, gell. man hat so viel zu tun, man muss so viel leisten. vielleicht dann einfach dort * ja, dass man dann nicht so bereit ist, wieder zeit in das ganze

* oder ja, es ist neben familie und job einfach nicht so einfach dann. wie gesagt, es ist ein sehr dichtes programm von euch auch, gell.

I: * zusatztermine zu finden, ist immer schwer.

B: ja.

I: und die meisten bestehenden termine sind inhaltlich schon so voll, dass man nicht * nicht wirklich was aufnehmen kann, was auch noch raum braucht, weil ein austausch im kollegium, das kann man ja auch nicht nur in zehn minuten machen, sondern das braucht ja auch wieder eine dreiviertel stunde * eine stunde, wo man wirklich einfach nur mal reden und austauschen kann.

B: mhm (bejahend). ja.

I: mhm (bejahend). gibt es denn bei ihrer schule, ähm rituale oder * andere methoden, die aus dem perma.teach * in den alltag eingeflossen sind? * also nicht nur speziell bei ihnen, sondern wo sie sagen: *mensch, dadurch, dass viele jetzt oder alle teilgenommen haben, hat es eine veränderung gegeben?

B: * ja, wie gesagt, ähm dieses * dass man seich ben die positiven sachen erzählt, gegenseitig am pausenhof.

I: mhm (bejahend).

B: ich glaube, das ist schon mehr so ein ganz wesentlicher punkt. mhm (bejahend).

I: und in der arbeit mit den kindern? gab es da irgendwelche rituale? manche machen ja dann freitags immer nochmal auch so eine * eine reflexion oder starten montags mit * mit was gutem oder * es gibt ja ganz viele verschiedene möglichkeiten.

B: ja, eben dieser erzähl * montagserzählkreis oder jeder schüler soll nochmal zurückschauen, die letzte viertelstunde, dass man einfach sagt: *okay, schauen wir mal zurück, was haben wir denn gelernt? ähm was hat uns befreti?* so sachen macht man dann halt, genau. ähm ja, habt ihr eine idee, was man morgen dazu machen könnte, was euch spaß machen würde. genauso sachen halt, dass man das dann macht.

I: mhm (bejahend). sind es * sind es methoden oder ideen, die neu dazugekommen sind oder die es davor auch schon gab?

B: 3Ü nein, die hat es davor auch gegeben.

I: (LACHT) ist ja letzten endes schön. also es muss ja nicht durch ein fortbildungskonzept alles neu ähm entstehen.

B: ja.

I: aber es ist ja schön, wenn man auch da wieder * da waren wir vorher bei dem punkt schon, wenn man entdeckt, vieles von dem, was sich schon die letzten jahre bewährt hat, ähm würde da als neue methode dazukommen, und man muss es nicht neu etablieren, sondern man macht es schon, man kann darauf aufbauen.

B: ja. mhm (bejahend).

I: okay, dann schaue ich nochmal. *3Ü* mhm (nachdenklich). gibt es denn was, was wenn * wenn sie jetzt die möglichkeit hätten, noch ein * was neues in der schule zu etablieren, umzusetzen? gibt es was, wo sie sagen, das habe ich mir aus dem perma.teach mitgenommen, das würde ich wahnsinnig gerne umsetzen.

B: *3Ü* ich muss jetzt auch nochmal schummeln.

I: ja, gerne.

B: ähm. *3Ü* ja, wir sind eigentlich * ja ich habe es * * mir dieses video, dieses friday, ist einmal vorgekommen, von einer schule in deutschland, ähm und wir haben jetzt eben am schulschluss eigentlich ein projekt vor, wo wir das eine woche dann machen. genau, das steht eigentlich schon an, und da haben wir schon eine konferenz drüber gehabt. ähm und das wir das jetzt eben gemeinsam mit der neuen mittelschule, ähm machen wir da zum schulschluss ein projekt, was dann eine woche andauert. wie es jetzt genau ausschaut, weiß ich noch nicht,weil wir haben jetzt in zwei wochen nochmal eine monatskonferenz, da reden wir nochmal genauer drüber, ähm und da wollen wir mal die Kinder untereinander mischen, also von den sechs - bis zu den 14-jährigen, und da wollen wir dann eben eine ganze woche an sowas arbeiten. genau.

I: sehr schön, was ja auch ein toller abschluss zum * zum jahresende hin ist, wenn man wirklich auch nochmal gemischt ähm an stärken arbeiten kann und nicht noch schulische themen abarbeiten muss.

B: genau und wo * also ähm, das erste mal die kinder nochmal hingesetzt werden, einfach mit denen sie arbeiten wollen und wo wir immer dann die möglichkeit haben, immer biologie - und physiklehre und soweiter, da die kinder wirklich schon in die gruppe einzubringen, wo sie wirklich interessiert sind. ja.

I: mhm (bejahend). sehr schön, das war auch ein * ein sehr schönes beispiel.

B: ja voll.

I: okay. hm (nachdenklich). gibt es irgendwas, was ich jetzt noch gar nicht abgefragt habe, was ihnen aber wichtig ist, wo sie sagen: *mensch, das würde ich gerne noch erzählen, oder das liegt mir noch auf der seele, oder das * war mir wichtig, oder?*

B: *8Ü* ich glaube, ich habe das meiste eigentlich gesagt. ja (LACHT).

I: ist ja völlig in ordnung, aber manchmal ist ja sowas, was man irgendwie, so eine idee, was man irgendwie noch erzählen möchte, und es hat dann nie platz. deswegen ist immer ganz wichtig, am schluss noch mal zu schauen, ob es noch irgendwas gibt, was man ähm unbedingt loswerden möchte. ähm ich fand sehr spannend, wie sie * wie sie erzählt haben, also man merkt ihnen dieses * dieses herzblut lehrkraft wirklich an, dass das einfach auch davor schon da war und dass es so eine bestätigung und bestärkung durch das perma.teach erfahren hat. * und hab mir da ganz, ganz viel mitgenommen, ähm auch so an so kleinen ideen, und ich finde, man merkt auch ganz viel, was * dass es sie in ihrer ähm ja erzieher, entschuldigung, lehrkraft persönlichkeit irgenwie so ein bisschen gestärkt hat. ähm und wie wir vorher schon festgestellt haben, es ist immer ganz schön, wenn die sachen auch nochmal einen neuen namen bekommen. also dieses ich habe schon mal so gemacht, und jetzt kriege ich einen neuen imput und stelle fest *oh ja, das habe ich auch schon gemacht*, oder das sind so sachen, die mir sehr nahe sind, und ähm finde ich auch ganz schön, wenn man da so eine bestärkung von der ähm eigenen Persönlichkeit auch noch mal erfährt.

B: danke schön.

I: haben sie denn irgendwelche fragen, irgendwas, was sie noch wissen wollen?

B: nein, jetzt gar nicht.

I: (LACHT) okay, also es würde jetzt folgendermaßen weitergehen. ähm, entweder von mir oder von der frau professor dr. lichtinger gibt es dann eben nochmal so einen kurzen ähm überblick, was die studie jetzt letzten endes so an ergebnissen geliefert hat. also, die kriegen sie auf jeden fall, was ja auch mal ganz spannend ist, wenn man gut teilnimmt. was ist denn daraus eigentlich entstanden?

B: mhm (bejahend).

I: ähm und wenn sie jetzt zu unserem interview nochmal fragen haben, können sie sich jederzeit an mich nochmal wenden. wenn es die studie allgemein betrifft, dann eben die ähm frau professor dr. lichtinger oder die ansprechpartner, die sie ähm über das perma.teach schon hatten oder kennengelernt haben. ähm ich bedanke mich auf jeden fall, und so zum abschluss würde ich sie bitten, dass sie mir vielleicht noch so drei sachen sagen, für die sie dankbar sind, so im rahmen mit diesem ganzen positiven.

B: (LACHT) ähm aus dem privaten bereich, oder ?

I: das ist völlig ihnen überlassen.

B: das ist völlig mir überlassen. ähm ich glaube, ich bin jedem tag dankbar, an dem ähm wir aufwachen, und begrüße meinen sohn, dann einmal mit einem gebet und wir lachen dann schon im bett für das bin ich einfach * mehr sonnenschein, und ich bin spät mama geworden. ich war schon 40, dann habe ich den bekommen hab, und es ist eine totale freude, dass man so ein kind haben darf, es ist toll wenn man in seiner arbeit aufgeht. ich muss sagen, wenn man mit freude in die arbeit
