# Supplementary material for: PERMA.teach: a study on the effectiveness of a standardized positive education training program in Austria
Source: Front Psychol. 2025 Apr 8;16:1516572. doi: 10.3389/fpsyg.2025.1516572 (PMC12053473; doi:10.3389/fpsyg.2025.1516572)
Supplement: Supplementary file 2 [file Data_Sheet_1.ZIP › Material PERMA.teach/Material LPs/Interviews LPs Teil 4.zip!/Interviews Transkription/Interview 5 - transkribiert.docx]

**Interview 5.m4a**

I: Genau, dass das für sie okay ist, und ja, sie haben eh schon mitbekommen. Es geht darum in diesen Interviews einmal herauszufinden, so grundsätzlich ihre Motivation an der Teilnahme, es geht um ihr Feedback, an den Schulungsbau steinen, die sie da eben erhalten haben, in der Schulung. Es geht in die Umsetzung, ihre Umsetzung im Unterricht, wie leicht, wie schwer ist das gefallen? Und ein bisschen die Veränderungen, die es dann vielleicht mit sich gebracht hat. Und ja, das war eigentlich schon. Und jetzt würde ich gerne anfangen mit der generellen Frage: nämlich einmal ganz zuerst zu ihnen als Expertin: Wie lange sind sie, sind sie denn schon Lehrerin? #00:00:53-4#

B: Das ist jetzt mein fünftes Jahr gewesen, also ich habe selbst Psychologie studiert, also Psychologie/Philosophie auf Lehramt, und deswegen auch natürlich das Interesse. Ja. #00:01:10-5#

I: Okay, und sie arbeiten an ihrer aktuellen Schule PTS 20, das darf ich wissen, sonst weiß ich ja nichts. #00:01:19-3#

B: Genau, das ist jetzt, also das war, da war ich jetzt das vierte Jahr, und das erste Jahr hatte ich eben ein Unterrichtspraktikum an zwei verschiedenen Gymnasien mit Deutsch, dem einen Fach, und Psychologie im zweiten Fach. #00:01:37-9#

I: Okay, und das Unterrichten sie auch an der, an der PTS? #00:01:41-2#

B: Nein, weil wir ja keine Psychologie/Philosophie haben. #00:01:44-9#

I: Okay. #00:01:46-1#

B: Aber ja, ich habe mich dann eben für alle möglichen Schulen beworben und wurde dann eben der FMS zugeteilt. Aber nicht jetzt aufgrund, sondern von den Fächern, sondern aufgrund von, ja Platzmangel für Psychologie-Lehrer innen an der AHS oder an höheren Schulen und aufgrund von Mangel an Lehrerinnen an den Pflichtschulen. #00:02:13-8#

I: Okay, aber das heißt, sie unterrichten welche Klassen an der PTS 20? #00:02:19-8#

B: Ähm, ich bin Klassenvorstand einer Klasse und habe vorwiegend eben Deutsch, und die individuell Kompetenzfördernden Fächer, und Sport habe ich auch. Ja, also, es ist alles so ein bisschen durchgemischt, vor allem, weil wir in der FMS haben wir ja jetzt nicht so diesen regulären, dieser reguläre Stundentafel, die man jetzt so kennt, mit Geschichte und Biologie und solchen Nebengegenständen, sondern eben diese Gegenstände, die einfach auf die Kompetenzorientierung, also Ziel ... Ähm, was jetzt persönliche Entwicklung oder soziale Gemeinschaft, oder Ähm ja, Lebensorientierung einfach betrifft. #00:03:11-2#

I: Okay, möchten sie uns, nur für das Verständnis, die FMS ist was für eine Schule? #00:03:15-7#

B: Eine Fachmittelschule adaptiert ans Polytechnikum. #00:03:21-8#

I: Aha, Okay! #00:03:23-9#

B: Ja, das ist quasi die neunte Schulstufe, ist ja nur ein einjähriger. #00:03:28-6#

I: Okay. Das wollte ich jetzt grad fragen. #00:03:30-7#

B: Genau. genau. #00:03:31-4#

I: Statt dem Polytechnikum? Ist das die 9. Schulstufen Ausbildung? #00:03:34-9#

B: Genau das ist so. Also, es ist eigentlich das gleiche wie die polytechnische Schule. Nur in Wien nennen sie es Fachmittelschule seit einigen Jahren, weil wir da ... #00:03:47-0#

I: Fachmittelschule, damit ich es richtig verstehe? #00:03:50-3#

B: Fachmittelschule. Genau. Das liegt an den, wir haben acht unterschiedliche Fachbereiche, die die Kinder wählen können. Also, sie haben in den ersten paar Monaten die sogenannte Orientierungsphase. Da wählen sie vier dieser acht Fachbereiche und schnuppern da quasi zwei Wochen rein, und nach dieser Orientierungsphase spezialisieren sie sich auf einen Fachbereich, und den haben sie dann das restliche Schuljahr, und es ist natürlich auch schon bezugnehmend auf das, was sie da nach diesem Jahr machen wollen. #00:04:25-3#

I: Alles klar! Das ist eine schöne Vorbereitung! Daher auch die kompetenzorientierten Fächer? #00:04:29-9#

B: Genau! Genau! Genau, genau genau! #00:04:31-5#

I: Ok. Super. Ok. #00:04:34-6#

B: Also, da passt auch diese eure, also eure, eure Themen passen einfach sehr gut auch in unsere Neben-Gegenstände. #00:04:47-1#

I: Genau. #00:04:48-4#

B: Und das eine ist eben die individuelle soziale Bildung, ISB, haben wir zwei Stunden in der Woche, das andere ist IKF, individuelle Kompetenzförderung, also, das passt da thematisch schon sehr gut rein, auch. #00:05:01-0#

I: Ja, jetzt rein für mich als jetzt Verständnisfrage. Das Polytechnikum, das einfach nur anders heisst in anderen Bundesländern, hat aber im Prinzip dann auch dieselben Kompetenzorientierung. Also, ist es auch, ist es auch so aufge, auf äh organisiert, ein Polytechnikum? #00:05:16-9#

B: Ja, ja. Ja. #00:05:16-9#

I: Oder ist da die FMH speziell? #00:05:20-5#

B: Na, ich glaube, dass das schon auch so. Ich muss ehrlich sagen, dass ich, bevor ich in diese Fachmittelschule vor vier Jahren gekommen bin, nichts mit einem Poly oder so zu tun hatte. Also ich bin gleich direkt in die Fachmittelschule rein, dass ich, ich weiß es ehrlich gesagt, nicht. Aber ein Schüler, der ist, - wo ist der hergekommen? Aus Oberösterreich, aus Völklabruck während dem Semester, jetzt im zweiten, und das ist, glaube ich, eine PTS, also polytechnische Schule, und die haben auch diese Fächer gehabt, die haben vielleicht die Fachbereiche ein bisschen anders, aber so von der normalen Stundentafel, von der regulären Stundentafel haben sie schon auch diese kompetenzorientierten Fächer. #00:06:04-7#

I: Das war jetzt rein informativ für mich, denn es ist ja wirklich toll in diesem einen Jahr mit PERMA, da kann man schon, also auf ganz anderem Boden, natürlich vorbereiten für die nächste, nächsten Schritte, für die jungen Menschen. #00:06:21-5#

B: Was auch, was auch eben toll ist, ist dieses "Talent-Loop", eben weil es ja auch bei uns extrem darum geht, ihre Stärken noch einmal zu, also nicht zu finden, aber sie auszuformulieren oder generell auch wieder neu zu entdecken oder zu erweitern, oder ja, finden, trifft eigentlich vielleicht eh nicht so schlecht, weil manche eher noch gar nicht so genau genau wissen, was sie tatsächlich eigentlich für Stärken haben. Und da ist diese diese Internetplattform auch richtig cool, und die, die habe ich am Schulanfang vor allem echt oft verwendet, um eben mit ihnen gemeinsam ihre Fähigkeiten und so weiter, auch zu zeigen. Ja! #00:07:17-5#

I: Kurz noch die letzte Frage zu ihnen als Expertin. Was hat sie denn motiviert, Lehrerin zu werden, wenn ich das fragen darf? Was ist ihre Hauptmotivation? #00:07:26-3#

B: Was hat mich motiviert? Ah. Ich glaube eigentlich meine eigenen Lehrer, aber nicht, weil sie es so toll gemacht haben, sondern weil ich mir gedacht habe, es könnte auch anders gehen. Immer Jugendliche, die vielleicht dann auch mal nicht ganz so in ihrer Höchs-Phase sind, anders motivieren, um vielleicht ihren Weg zu finden. #00:07:54-7#

I: Die Frage zur, die jetzt kommt, die haben sie mir eigentlich schon beantwortet, die Fortbildung, die sie da ausgewählt haben. Hatten sie schon Vorwissen zur positiven Psychologie oder zur positiven Bildung, in dem Fall, da sie es studiert haben? Haben sie im Studium davon erfahren, zumindest? #00:08:17-3#

B: Ja, einbisschen. #00:08:19-0#

I: Auch über die Positive Psychologie? #00:08:21-8#

B: Ahm. War sicher auch ein Thema im Bereich, der mal in einer Vorlesung angeschnitten wurde, so jetzt. Ich glaube, ich hatte mal ein Wahlfach über Positive Psychologie und auch Glücks-Philosophie und so in die Richtung gehend. Also, ich glaube, ich habe eh eine Bekannte, die Sonja Käferböck, so ja. mit der hab ich studiert, gemeinsam, ja, ja! #00:08:52-5#

I: Die kenne ich, weil ich mit ihr auch schon gezoomt habe. #00:08:58-2#

B: Gezoomt ah ja gezoomt. Die hat da ja mit geholfen, und ihre. Eh in , weil ich habe dann mit ihr auch gemeinsam Ethik studiert, den Master. #00:09:13-6#

I: Ja. #00:09:14-2#

B: Und da hat sie, glaube ich, dann ihren Abschluss, also ihre Masterarbeit, eben über diesen Bereich geschrieben oder so. #00:09:22-0#

I: Wüßte ich jetzt gar nicht, so gut kenne ich sie nicht. Ihr Name ist natürlich, merkt man sich, und ich habe auch mit gesprochen in diesen Zoom-Meetings da waren wir gemeinsam. #00:09:33-4#

B: Also ja, vom Studium her kam das wieder thematisch. #00:09:35-3#

I: Das Thema positive Bildung. Was hat sie daran überzeugt, oder was ist das? Was ist da, gibt es da für sie für einen Beweggrund? #00:09:45-4#

B: Na ja, gerade unsere Jugendlichen haben ja recht, meist, also zumeist recht große und schwere Rucksäcke zu tragen, weil die meisten jetzt das nicht ganz so einfachen sozialen Verhältnissen oder Bedingungen auch kommen und viele von ihnen auch gar nicht jetzt von daheim das mitbekommen, dass sie jetzt, weiß ich nicht, optimistisch bleiben, positiv bleiben oder was auch immer. Sie haben da schon so ein gewisses Bild oder eine gewisse Schublade, in der sie stecken, und ich denke mir halt, dass das eben durch positive Psychologie auf jeden Fall gefördert werden kann, dass sie da eben vielleicht einmal hinausschauen aus ihren Schubladen oder weiß ich nicht über ihren Tellerrand irgendwie hinausgehen. Ähm, und ja, ich glaube, es ist extrem wichtig, dass man sie in diese Richtung einfach stärkt und eben auch sagt: "Okay, auch wenn jetzt fünf mal was daneben geht, heißt das nicht, das ist beim sechsten Mal nicht funktionieren kann," und dass einfach eine positive Einstellung das um und auf ist und dass das einfach auch jetzt in Bezug auf irgendwelche Zukunftspläne aufgehen kann. #00:11:23-5#

I: Ja, was würden sie denn sagen? Was ist der größte Unterschied zwischen einem Regelunterricht und einer positiven Bildungs gefärbten Unterricht? #00:11:34-0#

B: Kann man das nicht vereinbaren? Also, ich weiß nicht ich. #00:11:42-4#

I: Also wenn jemand erklären würden, was sie jetzt, was sie jetzt gelernt haben oder was sie tun, gegen, also im Vergleich zu Regelschulwerk. Gibt es einen? #00:11:52-7#

B: Regelschulwerk heißt ähm? #00:11:55-3#

I: Klassisch, also ohne der positiven, ohne diesen positiven Ansatz. #00:11:59-3#

B: Also, ich habe auch in meinem Regelunterricht jetzt vor PERMA und so weiter, versucht möglichst positiv auf die Schülerinnen einzuwirken. Also, das ist eine schwierige Frage, weil ich habe jetzt, ich habe jetzt nie, ich war nie Fan vom Frontalunterricht und irgendwie Angst machenden Vokabelprüfungen oder so, also von dem her ist es. #00:12:27-4#

I: Eine Haltung. #00:12:28-4#

B: Eine Haltung, eher ja, und das, was man halt versucht, glaube ich, von Anfang an mitzugeben, wenn man das irgendwie. #00:12:35-9#

I: Ja, aber das heißt, sie haben jetzt auch keinen großartigen Haltungs-Schwenk hinlegen müssen mit dieser neuen Fortbildung oder mit diesen neuen Information. #00:12:45-4#

B: Mhm, nein, also nein, das ist nicht. Ich habe einfach bewusst dann mit diesem Buch auch gearbeitet und bewusst betont: "Okay, dieses und jenes Projekt gibt es. Wir wollen noch mehr darauf zielen, dass man das vielleicht fördert und das, was euch irgendwie rausholt oder mit euch Ähm erarbeitet", und dann schon immer wieder einfach Übungen aus dem Buch mit ihnen gemeinsam gemacht, besonders halt eben in diesem, in dieser individuellen Kompetenzförderung. #00:13:21-2#

I: Wie haben sie von dieser Ausbildung erfahren? Wie haben sie von dieser PERMAteach Fortbildung erfahren, und warum haben sie daran teilgenommen? #00:13:33-0#

B: Ähm, durch eine Kollegin, die G. E., die das ja uns dann vorgestellt hat, und dann waren, glaube ich, eh auch einmal zwei, also die Eva. #00:13:47-4#

I: und die Ingrid, glaube ich, waren dann auch bei ihnen, oder? #00:13:49-5#

B: und die Ingrid. Genau waren bei uns in der Schule und haben uns das eben vorgestellt, und ja, also mich hatte man schon bei Positiver Psychologie, also mir musste man das nicht großartig erklären. Ich fand das von Anfang an toll, weil eben, wie sie sagen, das ist eine Haltung, und es sollte eine Selbstverständlichkeit sein, die man jetzt eben in den Regelunterricht mit einbringt und mit einbezieht, durch kleine Übungen oder, weiß ich nicht, einfach durch positive Worte. #00:14:26-8#

I: Genau also, die Haltung ist da. Die war vorher schon da, sagen sie, hat sich sozusagen durch die Schulung ihre positiven Erwartungen an diese Schulung bestätigt? Ihre positiven Gedanken haben sich die bestätigt oder wars vielleicht irgendwie ... ja, hat sich was verändert? #00:14:46-8#

B: Ja, ich finde es einfach schön, dass dadurch, das eben ins Bewusstsein gerufen wird, dass man es halt jetzt nicht so nebenbei macht, sondern wirklich bewusst sagt, okay, man nimmt sich zwei Stunden jetzt wirklich Zeit, dass man bestimmte Übungen macht und dass man mit den Jugendlichen gezielt das trainiert. #00:15:06-6#

I: Mhm gut, also ihre Erwartungen für dieses Fortbildungskonzept, um das auch nochmal abzufragen. Sie sagen, man hat sie nicht lange überreden müssen. Was waren denn ihre Erwartungen? #00:15:16-6#

B: Erwartungen? Hatte ich eigentlich jetzt keine großartigen. Vielleicht, dass man mehr Kolleginnen davon überzeugt, da auch ein bisschen bewusster hinzuschauen. Ja, ja, ja! #00:15:41-8#

I: Was heißt, die Erwartungen waren es nicht groß, das heißt, es gibt auch keine Enttäuschungen? #00:15:48-7#

B: Ja, natürlich ist es immer, wenn man, wenn man sich eine Übung irgendwie vornimmt und sich das in Gedanken durchskizziert, wie das verlaufen könnte, und dann funktioniert es nicht so mit den Schülerinnen, weil die jetzt halt nicht so den super optimistischen, positiven Tag haben, dann natürlich ist man ein bisschen enttäuscht und denkt sich ja, warum hat das jetzt nicht funktioniert? Aber gut, dann macht man dasselbe am nächsten Tag mit einer anderen Klasse, und es funktioniert wieder um einiges besser. Also, das ist ja auch immer tagesaktuell. #00:16:27-6#

I: Also, das heißt genau, ihre Enttäuschung war nicht so groß, dass die also, wenn, wenn, wenn es nicht funktioniert hat, dass man gleich gesagt hätte, dann lassen wir es? #00:16:39-9#

B: Ach so, nein, nein, das ist sowieso, das kann man in der Schule sowieso nicht, weil dann müsst man wahrscheinlich eh gleich im ersten Semester aufhören. Oder gar nicht anfangen. #00:16:53-2#

I: Ähm. Jetzt, nachdem sie sagen, sie haben in ihrer, in ihrem, also sie haben, also. Die Frau E. hat das Team sozusagen motiviert. Bei ihnen war die Motivation leicht. Wie war es denn mit den anderen Kolleginnen? Sie sagen, sie hatten die Hoffnung, dass die anderen auch mitziehen? Hat das funktioniert? #00:17:10-5#

B: Ich muss sagen, wir sind ja gerade auf Expositur, das heißt, unser Team ist ein bisschen aufgeteilt an verschiedenen Standorten, und ich weiß jetzt gar nicht, inwiefern die Kolleginnen das jetzt so tatsächlich ausgeübt haben in ihren Stunden. Da hatten wir einmal, glaube ich, ein, eine gemeinsame Fortbildung dieses Jahr, aber es war mitten während des Schuljahres, und genau da sind wir dann schon zusammengekommen und haben uns ausgetauscht, aber sonst habe ich nicht wirklich was mitbekommen, ähm! #00:17:52-1#

I: An ihrem Standort sind wie viele LehrerInnen? #00:17:54-1#

B: Na ja, da waren wir zu dritt und ich war aber die einzige, die eben IKF hatte. Mit den beiden Klassen, das heißt eigentlich habe vorwiegend ich mit dem Buch gearbeitet, Und wir gesagt haben, okay. #00:18:11-9#

I: IKF? #00:18:12-0#

B: Das ist diese individuelle Kompetenzförderung. #00:18:15-2#

I: Okay, na, damit man dann auch ausschreiben können. #00:18:18-0#

B: Ja, ja, ich, meine Frau E. , die G. hat eben auch, ich glaube sie hat immer wieder mal ein paar Sachen draus gemacht. Aber grundlegend haben wir gesagt, okay, ich übernehme PERMA einmal in der Woche für zwei Stunden in jeder Klasse. #00:18:33-2#

I: Genau, das ist nämlich meine nächste Frage. Wie haben sie denn das Wissen, oder diese Fortbildung dann umgesetzt in ihren Alltag? Indem sie einmal in IKF? #00:18:43-0#

B: Eben einmal in der Woche für zwei Stunden genau, und das war meistens also in der einen Klasse war es geblockt, und in der anderen Klasse waren zwei Einzelstunden, und in diesen Stunden, außer es gab jetzt irgendwas anderes Wichtiges zu tun, habe ich mich eben darauf fokussiert und genau und Übungen halt rausgenommen und die vielleicht dann auch noch ein bisschen weiter ausgeführt, noch und detailreicher oder irgendwas, was ich dann selbst aus meinen Vorbereitungen dazu passend fand, habe ich dann auch gleichzeitig dann da anhängt. Und ja. #00:19:27-0#

I: Weil, weil sie das gerade in der Hand haben, haben sie das Buch eigentlich auch gut gebrauchen können? Dieses Buch ist gut in den Einsatz, und sie haben es als Arbeitsgrundlage gut verwenden können? #00:19:40-6#

B: Genau das Buch eben, und die Talent-Loop Plattform. #00:19:46-4#

I: Wie hat das ausgeschaut? Ich springe jetzt ein bisschen vor, weil wir gerade bei dem Thema sind, Das Padlet und diese digitale zur Verfügungsstellung? Haben sie das brauchen können? Wie haben sie damit umgehen können" #00:20:00-6#

B: Welches war das? #00:20:02-3#

I: Da gibt es ein Padlet. Es gibt sogar zwei Padlets, also digitale Plattformen, wo ganz viel, auch Material drauf ist. #00:20:12-9#

B: Okay. Das, das weiß ich jetzt nicht. #00:20:15-0#

I: Okay, sie habens gar nicht? #00:20:16-3#

B: Ähm, nein, also kann sein, dass, das ist sicher mal erwähnt worden, aber das habe ich nicht. Ich war eh, wie gesagt, ganz gut versorgt mit dem Buch und mit. #00:20:26-2#

I: Genau. Also, off the record kann ich ihnen gerne, wenn sie möchten, diese, diesen Link, zu diesen Padlets schicken, dann können sie gerne nochmal reinschauen oder haben sie für die letzten zehn Jahre Material #00:20:39-0#

B: Okay, für die nächsten zehn. Super, sehr gut! #00:20:42-9#

I: Also, sehr umfangreich, aber wenn sie möchten, schick ich ihnen gerne. #00:20:48-9#

B: Nein, ich bin sowieso ich. Ich suche mal gerne immer neue und unterschiedliche Sachen, weil mir sonst eher auch. Ich meine, es kommen zwar jedes Jahr immer neue Schüler, und eigentlich könnte ich es immer gleich machen, aber das ist mir dann halt auch ein bisschen zu zu fad. So für mich selbst, ja! #00:21:06-4#

I: Auch sie müssen auf ihr PERMA, auch ihr PERMA muss gefüttert werden. #00:21:11-4#

B: Wobei es eh dann natürlich immer anders ist, weil das keiner ja gleich macht, und manche mögen das, manche mögen was anderes. #00:21:21-1#

I: Wenn ich grad bei der Abfrage der Materialien bin: wie war denn diese PERMA Post? Haben sie die erhalten? War die für sie hilfreich? #00:21:32-3#

B: Ja, wobei ich ehrlich sagen muss, die habe ich eher, ja oft mal weggeklickt #00:21:43-9#

I: Wars lästig, das sie so oft kam? Oder war´s ok? #00:21:47-3#

B: Es war schon, ahm, es ist dann schon oft ein bisschen viel, und wenn man dann halt gerade so im Tun und Machen ist, und ja, es ist eh ah, wir bekommen recht viele E-Mails, wir bekommen recht viele Infos, und oft mal ist es dann in einer Woche einbisschen too much dann auch noch von extern, also von außerhalb, Input zu bekommen. #00:22:19-9#

I: Also, für sie, für sie war das nicht sehr hilfreich? Sie hättens nicht gebraucht, oder? #00:22:25-8#

B: Ja. Nein. Also natürlich, wenn ich jetzt keine, also wenn ich nicht eh schon genug Material gehabt hätte, wäre es sicher gut gewesen, da nochmal dann vielleicht mehr Informationen zu bekommen. Aber ich habe, ich habe mich ausreichend, ja versorgt gefühlt, mit dem, was ich hatte. #00:22:47-1#

I: Darf ich jetzt ein bisschen zurückspringen auf die Schulungs-Bausteine? Wie bewerten sie für sich diese Schulung-Bausteine: Modul eins und Modul zwei? Das war ja, glaube ich, bei ihnen, ihrem Fall auch, also vor Ort, und einmal digital,. #00:23:03-8#

B: Mh. Mh #00:23:04-7#

I: Hat es da für sie gepasst? Von der Länge her, von von dem Timing? #00:23:08-6#

B: Ja, an sich absolut absolut, Ahm, wobei ich jetzt ehrlich zugeben muss, dass ich gerade das letzte Jahr ahm war. Ich habe ich eben den zweiten Master fertig gemacht in Ethik, und ich war mit meinen Gedanken bei der Schule, und ich war bei meiner, bei meiner Masterarbeit und meiner Masterprüfung. Und toll! Aber ich habe mich jetzt nicht so 100 prozentig da hinein gefühlt, wie ich es vielleicht könnte, wenn ich mich, wenn ich die Kapazitäten noch dafür gehabt hätte. #00:23:51-0#

I: Aber würden sie jetzt sagen, die Präsenztage waren waren für sie besser, oder die virtuelle Geschichte hätten sie präferiert? Einfach nur um für die Zukunft, für die nächsten Ausbildungen ihre Einschätzung? #00:24:02-9#

B: Alles, was alles, was face to face stattfindet, ist, finde ich netter und bleibt, bleibt hängen, weil wenn man das Ganze via Zoom macht, läuft es halt gleich Gefahr, dass man sich sich ablenken lässt, indem man halt, weiß ich nicht, irgendwas anderes nebenbei macht. Und das machen wir alle in Wirklichkeit, weil, ja. #00:24:26-7#

I: So ist es halt. #00:24:28-5#

B: Ja, da muss man so ehrlich sein, das auch, wenn ich meine, natürlich, es ist interessant, und es ist, man fühlt, oder man versucht dann eh, dabei, aktiv zu sein. Aber wenn man jetzt dann eh schon einen recht stressigen Tag hat, und dann geht es am nächsten Tag stressig weiter, und dann muss man aber irgendwie jetzt noch Hausübungen korrigieren. Naja, wann macht man das? Und es ist halt schwierig natürlich. #00:24:54-0#

I: Präsenz heißt halt, ich bin wirklich voll, voll, ganz da. #00:24:56-7#

B: Ich bin da. Ja, und ich bin dabei, und ich, ich fokussiere mich auf das, was passiert. Ich finde, alles Virtuelle ist halt so. Ja, ist zwei, zwei Welten, irgendwie? Oder? Weil? Ich bin da jetzt auf meinem Balkon, und gleichzeitig bin ich aber bei ihnen, und irgendwie ist es halt zerrissen. Also, es fühlt sich zerrissen an. #00:25:16-7#

I: Ist schon merkwürdig, aber die neue, neue Form des Kommunizierens. #00:25:21-5#

B: Ich meine, es ist natürlich. Es ist natürlich super, an zwei Orten gleichzeitig zu sein, aber, ist halt auch irgendwie ein innerlicher Stress. Dann und dann läuft man halt Gefahr, dass man dann noch eine dritte andere Sache macht. #00:25:37-6#

I: Also noch noch an einem Ort, noch einem Extraort. Hat die Vernetzung mit den anderen Teilnehmern für sie stattgefunden? Jetzt unabhängig davon, dass sie sagen, sie haben gar nicht so viel Zeit gehabt. Aber haben sie das Gefühl gehabt, dass die Vernetzung, also auch dieses Sharing, dieses Peergruppen-mäßige mit anderen Teilnehmer? #00:25:58-9#

B: Das fand ich, das finde ich schon interessant, dass wir uns dann ähm, eben durch die Möglichkeit von Zoom wirklich auch mit Vorarlberg, und weiß ich nicht, was für Bundesländer dann noch connecten konnten. Das war schon interessant zu sehen, und das fand ich auch. Da hatten wir eh einmal so ein Zusammenkommen, eben wo wir uns alle bei uns in der Schule getroffen haben und alle Lehrer in der einen Schule und so, und dann hat das Ganze auch noch einmal virtuell stattgefunden. Das fand ich besser als dieses "Jeder sitzt einzeln vorm Computer" weil da ist halt dann trotzdem ein bisschen eine Präsenz da. Aber man kann sich auch mit anderen austauschen, die halt weiter weg sehen, und dass sich alle gleichzeitig irgendwo treffen ist eh unrealistisch, aber. #00:26:49-4#

I: Jetzt ganz kurz, also, das muss ich jetzt einfach abfragen, um auch die Verbesserungsmöglichkeiten abzuklopfen. Wie bewerten sie die fachlichen Impulse der Eva und der Ingrid oder eben anderer, die sozusagen die Schulung gemacht haben? #00:27:09-2#

B: Ähm, den fachlichen Input? Ah, das ist jetzt auch schon wieder lang her. Ahm, ich versuche mich da gerade an unsere erste, an unser erstes Zusammentreffen zu erinnern, wo die zwei in unserer Schule waren, und das war, das war gut, das war gut, weil sie uns da wirklich auch einmal das Ganze vorgestellt und präsentiert haben, und man konnte das, ich glaube, sie haben da auch gewisse, also so ein paar Spielchen mit uns gemacht und so, und wo wir dann auch aufschreiben mussten und halt wirklich selbst auch was tun konnten, und anhand dessen haben sie es uns dann erklärt, eigentlich, wie das ganze sein soll, und das war, das war gut. Aber da war es halt auch wieder, die Präsenz bleibt halt hängen, wenn man was tut oder gemeinsam etwas arbeitet. #00:28:15-5#

I: Genau. Die schriftlichen Handouts? Also ich meine jetzt nicht nicht im speziellen das Stärken Buch, sondern da gab es ja über Internet, glaube ich, hat man ihnen dann auch die Modul eins und Modul zwei geschickt! Da waren auch teilweise Apps drauf. Weitere Inspirationsquellen. Haben sie damit irgendeine positive Assoziation, oder? #00:28:36-6#

B: Das hab ich übersprungen. #00:28:38-4#

I: Ja, gut. #00:28:44-6#

I: So, dann. Ja, dann einfach auch noch zur Verbesserung: die Rahmenbedingungen, also die Einladung zu den Terminen, die Anmeldungen, die Zoom-Räume. Hat das gut funktioniert, haben sie sich professionell aufgehoben gefühlt? #00:29:00-4#

B: Ja, das hat gut funktioniert. Ja, das auf alle Fälle. Also generell ist das ganze ja sehr professionell aufgezogen. Also das, ich glaube, da gibt es ja auch, würde ich jetzt sagen, kaum an Verbesserungs, also wüsste ich jetzt nicht, wie man das verbessern könnte, weil es ist ja wirklich alles bedacht. Es gibt für, glaube ich, also, es gibt die Bücher, es gibt die Post, es gibt diese Online-Plattformen, also ich glaube, man kann es nicht mehr abdecken, als es eh schon abgedeckt ist. Es gibt diese, diese, diese Cafes, diese PERMA Cafes, wo man. Also Stärken-Cafes. #00:29:39-8#

I: Haben sie daran teilgenommen? #00:29:43-3#

B: Nein, Aber ich habe davon gelesen, und ich habe gedacht, ja, das wäre sicher nett. #00:29:47-4#

I: Das wäre eben gedacht als Austausch oder unter den, aber das kann man ja auch sonst. #00:29:55-4#

B: Ja, ja, ja. gut, ich finde das. Ich find so ein Stärken-Cafe fände ich auch für die Schüler eigentlich recht interessant, so etwas mal zu machen, aber da müsste ich mir das von euch anschauen. #00:30:10-0#

I: Es geht ja da auch nur darum, dass sozusagen in den Breakout-Rooms jeder erzählt, was ihm am besten gefallen hat, was bei ihm in der Schule am besten funktioniert hat, und erzählt damit von seinen Stärken oder von den Dingen, die gut geklappt haben, und und. #00:30:25-4#

B: Aber das könnte man mit den Schülern genauso, also. #00:30:27-3#

I: Einen riesen Blumenstrauß an Möglichkeiten, an neuen Interventionen, an auch Erfahrungsschätzen, also keine Ahnung, z.B.: das ist in der Theorie ein bisschen zu lang angeleitet. Ich habe es so gemacht, und die Kinder haben haben viel schneller mitgemacht, zum Beispiel. Also, das sind so diese Erfahrungen, die dort im Stärken-Cafe ausgetauscht werden. Und zu Recht, dass ist genau das, was es dann auch die Ebene geschafft hat, das kann gut sein. #00:30:52-3#

B: Und wenn man das auch, ich finde das eben so nett. Also die Bezeichnung Stärken-Cafe, wo man Stärken und sich so austauscht oder so, reflektiert ja. #00:31:02-3#

I: Genau, jetzt muss ich nochmal ganz kurz zurück, weil das sind wir drüber gesprungen ahm. Würden sie diese Fortbildung jemanden empfehlen? Würden Sie sagen, ja, das kann ich empfehlen? #00:31:15-3#

B: Ja, würde ich allen LehrerInnen empfehlen, weil, wie gesagt, ich finde, dass das etwas ist, was eine Selbstverständlichkeit in den, also eigentlich für jeglichen Regelunterricht sein sollte. #00:31:33-6#

I: Gibt es etwas, was gegen diese Form gesprochen hat oder gegen die Fortbildung spricht? #00:31:39-1#

B: Nein. #00:31:41-7#

I: Sehr schön. #00:31:41-8#

B: Da würde mir jetzt nichts einfallen. #00:31:43-5#

I: Hätten sie sich etwas anderes gewünscht? Etwas, was gefehlt hat? Sie haben vorher gesagt: es hat eh alles gegeben, digital, und #00:31:50-7#

B: Ja, dass ich mehr Zeit gehabt hätte, um mich da mehr hineinfühlen zu können. #00:31:56-0#

I: Mhm, Zeit ist das, was wir nicht vermehren können, leider. Aber sonst gibt es nichts, wo sie sagen, was sie dem Fortbildungskonzept jetzt noch hinzufügen würden? Wo sie eine Empfehlungen hätten für die nächste, für die nächsten Schulen oder LehrerInnen? #00:32:14-4#

B: Mh. Ich schaue gerade mal kurz das Buch durch. Irgendwas? Mh. Eigentlich finde ich´s gut so, wie es ist. #00:32:39-9#

I: Das nehmen wir gerne mit. #00:32:44-4#

B: Ja, ich glaube, ihr habt auch. Ich glaube, ihr habt auch recht viel Arbeit hineingesteckt. #00:32:50-1#

I: Ja, die Eva und die Ingrid sind natürlich Tausensasserinnen und haben wirklich Unmengen an Material, in Buchform, in Digitalform, vielleicht als Erinnerung, jeden Tag oder jeden dritten Tag noch als e Mail, also für alle Typen ist was dabei. #00:33:13-9#

B: Ja, eben, und das habe ich auch vorher gemeint, es ist wirklich egal, wie man arbeiten möchte, man kann sich so viel rausnehmen. Also ja! #00:33:28-9#

I: Hätte ich noch ein paar Fragen zur Umsetzung im Unterricht? Sie sagen, sie haben es ganz spezifisch im IKF, in der individuellen Kompetenzförderung wöchentlich eingesetzt, jeweils zwei Stunden, ahm und da mit dem Buch schön arbeiten können. Gibt es etwas, wo sie gesagt haben, darüber hinaus, über die IKF habe ich auch das eine oder andere angewendet, also im Deutschunterricht? #00:33:59-5#

B: Ja, aber witzigerweise jetzt nicht ich, sondern die Schülerinnen dann selbst. Sie haben dann oft auf das verwiesen, #00:34:07-0#

I: ok? #00:34:09-3#

B: Und irgendwie daran erinnert. So. Wir haben einmal dieses Lach-Yoga gemeinsam gemacht, und das wollten sie dann halt immer machen. "Wann lachen wir wieder mal eine Minute?" #00:34:20-9#

I: Mhm, okay, also, sie haben sie darauf aufmerksam gemacht? #00:34:25-2#

B: Die Schülerinnen also, wenn ihnen irgendwas besonders gut gefallen hat, dann haben sie schon immer wieder darauf verwiesen. Und ja, ich mache generell mit ihnen auch so im Sportunterricht immer wieder Achtsamkeitssachen. Und ist jetzt nicht unbedingt, nehme ich mir jetzt nicht unbedingt aus dem Buch raus, aber ist grundlegend einfach. Oder wenn ich merke: Okay, jetzt in Deutsch, es reicht einfach ahm, weiß ich nicht, Erörterungs-Aufbau, dann sage ich: Okay, passt. Machen wir kurze Pause, und wir stehen auf und machen halt irgendwelche Achtsamkeitsübungen oder irgendwelche Bewegungsgeschichten, wo sie einfach wieder zu sich selbst finden. Und. #00:35:19-1#

I: Weil im Fachunterricht zum Beispiel die Möglichkeiten, auch die Texte anders zu lesen oder anders zu interpretieren. Haben sie sowas, so fachspezifisch etwas verändern können aufgrund dieser neuen PERMA ähm Geschichte, also die positiven Worte, die positiven Emotionen oder solche Dinge? #00:35:43-9#

B: Ahm. Das habe ich nicht gezielt gemacht, aber es ist auf jeden Fall eine Idee, also vor allem dieses Text anders lesen. Ja. ja. Das muss ich mir gleich irgendwo aufschreiben. #00:35:56-7#

I: Die Stärken, das ist ja auch so nett, immer die Stärken des Protagonisten analysieren. #00:36:02-0#

B: Ja, ich meine natürlich, wir arbeiten sehr viel mit, sowohl in also in jedem Fach, eben mit ihren Stärken, und auch wenn es jetzt im Deutschunterricht, weiß ich nicht, um Charakteristik geht, irgendeiner Person, haben sie auch dann die Stärken aus Talent-Loop rausgenommen oder so also, und ich habe es ja auch die Definitionen und so aus dem Talent-Loop dann heraussuchen lassen und dazu passend in einen Text verpacken. Also, so haben wir dann auch gearbeitet. #00:36:43-0#

I: Also, der Fokus wurde verändert? Angeregt. #00:36:48-9#

B: Jaaa. Und man kann es auch richtig gut adaptieren und an den, an die verschiedenen Fächer anpassen. #00:36:57-9#

I: Gibt´s neben den Ritualen oder den Projekten, die sie früher schon hatten, was also irgendwas, was jetzt in dem letzten Jahr, in dieser Zeit, als sie diese Fortbildung gemacht haben, sich verändert hat? Dass es am Morgen andere Rituale gibt, Stärkenduschen? Sie kennen diese Interventionen, haben es da irgendwas eingeführt? Also abgesehen davon, dass die Kinder das Lachyoga? #00:37:20-5#

B: Rituale nicht? Nein, nein, wir haben immer unterschiedliche, unterschiedliche Übungen gemacht. #00:37:27-3#

I: Also Abwechslung. #00:37:28-9#

B: Ähm, was wir schon öfter gemacht haben, waren so kleine Dankbarkeits-Zetteln. Also, wenn jetzt irgendwer, das haben wir uns ausgemacht, wenn irgendwer einem eine Freude gemacht hat oder wenn jetzt gerade irgendwas besonders toll war, von der oder der Person ausgehend, dann haben sie sich immer so kleine Post-its zugesteckt. #00:37:49-7#

I: Okay. #00:37:49-9#

B: Und das haben sie dann auch angefangen, selbst zu machen, ohne dass ich´s jetzt irgendwie ihnen beauftragt habe, sondern einfach so kleine Nettigkeiten, halt zwischendurch. #00:37:59-2#

I: Mhm. mhm. #00:38:00-0#

B: Und das war tatsächlich schön zu sehen, als sie das von sich selbst, es ist nicht oft vorkommen, aber schon, wo sie dann eben erzählt haben, ja, sie haben heute ein Post-it bekommen von der und der Schülerin oder von dem und dem, und dann haben sie sich natürlich gefreut. #00:38:18-8#

I: Mhm. Haben sie, also weil sie es jetzt gerade selber anschneiden, die positiven Veränderungen in der Gruppe, in der Klassengemeinschaft? Gibt es da Dinge, die ihnen aufgefallen wären, dass man sagt, weil halt jetzt andere Rituale oder andere Interventionen gemacht werden, dass es sich verändert in der Klasse? #00:38:39-8#

B: Ich habe schon im Laufe des Jahres, es ist bei uns, sie lernen sich, die meisten kennen sich ja nicht. Sie kommen komplett zusammengewürfelt aus ganz unterschiedlichen Bezirken, aus ganz unterschiedlichen Schulen, auch aus unterschiedlichen Schulstufen, zu uns. Und Ähm, deswegen zielen wir einfach von Tag eins auf Stärkung der Klassengemeinschaft und natürlich am Anfang, sind sie alles so. Nein, sie wollen sich da jetzt nicht großartig irgendwie anfreunden, weil erstens: Ähm, sehen sie ja viel zu cool dafür, jetzt mit 14, 15. Zweitens ist es eh nur ein Jahr, aber trotzdem, das merke ich wirklich jedes Jahr. Innerhalb von den ersten zwei Wochen schwenken die so zusammen, einfach weil es auch so ein intensives Arbeiten mit den Lehrern auch ist, weil wir haben sie ja, ich habe meine Klasse meistens 19 Stunden in der Woche. #00:39:44-9#

I: Das ist viel. Ja. #00:39:46-0#

B: Also, ich habe sie ja in ganz vielen verschiedenen Fächern, und jetzt, wo wir die auf Expositur sind, ist es halt, diese zwei Klassen sind quasi, für die sind wir verantwortlich. Letztes Jahr waren wir zu dritt, also ein Lehrerinnen-Team aus drei, heuer sind wir dann oder nächstes Jahr zu viert. Also, es ist schon ein sehr intensives Arbeiten und sehr auf Beziehungsebene, und deswegen ist so dieses untereinander und miteinander sehr, sehr ausgeprägt. Und ja, demnach besteht diese Klassengemeinschaft dann auch zwischen den Schülern, und ich habe gemerkt, am Anfang waren sie eben noch dieses vorsichtige und dieses Ähähäh, aber es hat sich auch unterm Jahr immer immer mehr verstärkt. Jetzt waren wir dann am Schluss auch auf Projekttagen, und das war, seit dem sind sie unzertrennlich. #00:40:49-2#

I: Ja, aber das würde jetzt bedeuten, dass dieses PERMA, diese fünf Punkte ja eigentlich dann auch erfüllt sind oder? So die positiven Emotionen, #00:41:00-0#

B: mhm, ja. #00:41:00-7#

I: Meistens gepaart mit Relations, mit der Beziehung, die dann aufgebracht wird. #00:41:07-4#

B: mhm. ja. #00:41:07-6#

I: dann Engagement für die Stärken, die wieder ganz wichtig sind, #00:41:12-0#

B: ja.ja. #00:41:12-7#

I: und Meaning und Ziel erreichen. Da werden sie dann auch wissen, wohin die Reise geht. #00:41:20-7#

B: Eben, und das ist eben, worauf wir eigentlich ja auch das ganze Jahr hinarbeiten, dass sie dieses Jahr nutzen, das alles noch einmal zu reflektieren, dass sie Stärken, finden, entdecken, umformen oder Ziele auch umformen, wenn es vielleicht dann doch was anderes in eine andere Richtung geht, und wir sie wirklich noch einmal festigen in dem, was sie wollen, was sie wollen, und nicht, was jetzt unbedingt wer anderer will oder was sie davor geglaubt haben. #00:41:59-9#

I: Genau. genau. Das würde heißen das PERMA. Sie spüren, dass sich das PERMA, also als Kultur, dann auch bei den Schülern entwickelt, in diesem einen Jahr. #00:42:14-8#

B: Unbewusst. Ja. Weil ich meine, sie haben dann schon natürlich immer so dieses PERMA, ja, war dann schon oft einmal, dieses Jahr "schon wieder", weil es ist, sie sind halt auch sehr schnell mal gelangweilt, und wenn man halt dann ein Buch zu oft zückt, dann sind sie auf dieser Schiene. "Äh.Warum wird schon wieder?" Und dann. Ich meine, sie haben dann eh begriffen ok. es sind nicht immer die gleichen Übungen, und wir machen eh immer was anderes und ihnen gefällt es dann eh, aber gerade mit 14, 15. Man muss sie halt sehr, sehr motivieren für alles. Das gelingt manchmal leichter, manchmal gelingt es nicht. #00:42:55-7#

I: Aber sie sehen schon die, also das Sie da mit ihrer PERMA Motivation oder mit ihrer Positiven Psychologie-Motivation natürlich da schon auch bei beitragen können? #00:43:05-9#

B: Das glaube ich schon, aber eben unbewusst. Sie würden jetzt, wenn ich. Sie würden jetzt, das glaube ich, nie so zugeben. Sie würden nie sagen, ja, aufgrund von diesem Buch oder aufgrund von PERMA sind wir jetzt ein bisschen positiver eingestellt. Aber man hat schon im Laufe des Jahres gemerkt, dass sie positiver werden, dass sie eben nicht gleich aufgeben, dass sie nicht gleich komplett schwarz malen, dass sie nicht alles negativ sehen und alles Scheiße ist. #00:43:32-6#

I: Mhm, genau. Schön. Gibt es diese diese Veränderung in ihrem Lehrerteam? Ich meine, sie sind jetzt zu zweit, sagen sie in der Expositur, oder sie waren zu dritt, und ich gehe jetzt mal davon aus, dass sie sowieso in der richtigen Haltung sind. Aber könnten sie vielleicht auch noch versuchen festzumachen, ob das auch sozusagen in ihrem Arbeitsteam Veränderungen mit sich gebracht hat, dass sie sich mit diesen Themen beschäftigen? #00:43:59-2#

B: Ähm, wir haben alle schon so, also, #00:44:07-1#

I: Gute Haltung? #00:44:12-2#

B: Ja, also wir. schon ahm.. Wir drei sind halt sehr, sehr, wie soll ich sagen, Lehrerinnen, die halt pro Schüler arbeiten, und da gehts nur mit einer positiven Einstellung und mit Optimismus und von dem her. Es war immer schon so bei uns, weil vielleicht eben einfach das bewusst, das bewusst darüber sprechen und das bewusst leben, das vielleicht. #00:44:48-3#

I: Oder als Bestätigung vielleicht, dass es, das sie auf dem richtigen Weg. #00:44:52-6#

B: Bewusst. Das bewusste Weitergeben und dass man sich wirklich auch Zeit nimmt und dass das vielleicht auch keine Selbstverständlichkeit ist? Und dass man das vielleicht auch mit den Schülern eben gemeinsam erarbeiten kann, weil eben, wie gesagt, unsere Schüler haben´s meistens wirklich nicht einfach, egal, woher sie jetzt kommen, und wohin sie gehen. Und viele von ihnen, die kennen so was wie LOB oder positive Worte, die kennen das nicht, was erschreckend ist. Aber, eben eh der eine Schüler aus Vöcklabruck der gekommen ist als Ähm im zweiten Semester, der ist mit einem also, der hat ein negatives siebte Klassen-Zeugnis sogar noch gehabt, also der war ein Kooperations-Schüler, der ist als Kooperations Schüler gekommen. Und dann eh die erste Schularbeit mit ihm in Deutsch und so weiter, und ich habe ihm halt ein bisschen geholfen, das zu verstehen, die Angaben und so, und dann sitzt er da und schaut mich und nach der Schularbeit, schaut mich mit großen Augen an und sagt, also er versteht es nicht. Also, ihm hat noch nie irgendjemand geholfen. Und da denke ich mir, der ist 15 Jahre und geht seit weiß ich nicht wie vielen Jahren in die Schule, und sagt mit 15, das ist das erste Mal, dass mir jemand geholfen hat. Man hat noch nie wer geholfen, da denke ich mir was? #00:46:41-8#

I: Ist das das Regelschulwerk, von dem ich vielleicht vorher gesprochen habe? #00:46:47-1#

B: Warscheinlich. Ja. #00:46:47-3#

I: Also ich kenne oder ich weiß jetzt nicht den Staus-Quo an anderen Schulen? Aber er spricht über etwas, was wir alle wahrscheinlich in unseren Schulen schon auch erlebt haben? #00:46:55-6#

B: Ja. Ja. #00:46:57-4#

I: Ja, schön, dass der so ein positives Erlebnis haben konnte. #00:47:03-5#

B: Ja, und das ist halt, und das ist ja auch dann so diese Kette, weil das gibt mir voll viel positive Energie dann wieder, und ich gebe das dann weiter den anderen Schülern, weil das natürlich sehr motiviert als Lehrkraft, wenn du so etwas hörst, und dann, es ist dann halt diese, dieser Kreislauf. #00:47:25-3#

I: Genau. Was mich zu dem Punkt bringt: Was hat PERMA ihnen gebracht? Ihnen ganz persönlich für diese fünf Kriterien? Wenn man eh schon in der richtigen Haltung ist, wahrscheinlich ganz marginal, vielleicht, aber hat es etwas in ihrer Haltung? Gibt es etwas, wo sie sich freuen, wie sie jetzt gerade erzählt haben, vielleicht? #00:47:46-6#

B: Ja, also das. So was sind dann halt natürlich immer die Momente, wo man sich denkt, okay, und genau deswegen mache ich das. Und genau deswegen ist es wichtig, dass man mit Schülern auf einer Beziehungsebene arbeitet, auf Augenhöhe ist und, ähm ja, keineswegs, dieses, weiß ich nicht, dieses Machtverhältnis, das irgendwie ausnutzt, oder weiß ich nicht. Das ist. Ja. Aber was hat mir PERMA persönlich gebracht? Ja, einfach ja, es ist schön, wenn man merkt, dass Schüler, Schülerinnen, die halt so was wie Hoffnung oder so, jetzt nicht wirklich haben oder hatten, sie vielleicht doch finden. Und man merkt, wie sich Beziehungen entwickeln zwischen den Schülern untereinander als auch zu uns. Das sie eine Sinnhaftigkeit sehen in den Dingen, die sie machen, das nicht einfach jetzt nur an ihnen vorbeigeht und sie das machen, weil sie es halt machen müssen, sondern dass sie auch verstehen warum. Das sie wissen: Okay, ja, Leistung ist schon wichtig, aber Leistung ist jetzt nicht alles, und ja, dass sie vielleicht dadurch einfach auch lernen, ein bisschen an sich selbst zu glauben und auch ihre Stärken als Stärken wahrnehmen und das zu schätzen wissen, was sie können, und nicht jetzt unbedingt an dem Festhalten, was sie vielleicht nicht können. #00:49:44-7#

I: Macht ihr, macht ihnen ihr Beruf noch mehr Spaß mit PERMA? #00:49:52-5#

B: Ja. #00:49:56-0#

I: Das war jetzt die Frage, die sich, wenn ich ihnen zuschaue, wie sie da in der Sonne so Strahlen, ja.ja ja. Aber ich nehms wieder raus. Es ist mir jetzt nur so aufgefallen. Es ist schön ihnen zu zu schauen, wie sie gestrahlt haben. Ahm. Also, die Haltung stimmt, aber wenn man dann trotzdem diese, wie sie es beschrieben haben, ich habe es gerade mit ihnen mit gestrahlt, da sind die Augen, diese jugendlichen Augen. Woran würde denn ein Aussenstehender bemerken? Jetzt bin ich wieder bei dieser anfänglichen Frage, das, dass sie positive Psychologie oder positive Bildung in ihrem Unterricht anwenden? Das ist ja nicht nur PERMA, sondern es ist ja ihre ihre Haltung. An was merkt das denn ein Außenstehender, also die Eltern ihrer Kinder. Merken die das? #00:50:46-8#

B: Ich hoffe. Wahrscheinlich am Engagement, vielleicht? Das man sich für die Kinder einsetzt, dass man ähm sie eben versucht, dazu zu bringen, ein bisschen über den Tellerrand zu schauen, dass man ahm sie motiviert, dass man ihnen wirklich das Gefühl gibt: Okay, ich glaube an dich, also glaube auch du an dich selbst, und dann halt einfach am am Umgang miteinander. #00:51:22-1#

I: Mhm. #00:51:23-0#

B: Dass man jetzt, weiß ich nicht, alleine, halt die Art und Weise, wie man in die Klasse geht, oder. #00:51:31-8#

I: Mhm. #00:51:32-1#

B: Das macht es halt auch schon nochmal aus. Also, ich habe das auch bei meinen eigenen Lehrern. Ich fand das, Ich fand nichts schlimmer, als wenn der Lehrer schon mit so einem Gesicht in die Kasse geht und nur angefressen ist, weil er, weil jetzt halt wieder Montag ist. Ich meine, natürlich kann ich jetzt auch nicht sagen, dass ich jeden Montag wie strahlend und im Hopserlauf in die Klasse renne. Das ist auch nicht der Fall. Aber dennoch. #00:52:01-9#

I: Man muss auch authentisch bleiben. #00:52:05-3#

B: Ja, genau, und dass man halt, weiß ich nicht. So viele Lehrer, nehmen, finde ich, haben halt einfach keine Freude an ihrem Beruf, und das ist so schade, oder verlieren die Freude oder verlieren den Blick aufs Wesentliche. Und das Wesentliche sind einfach die Schüler, und auch wenn sie furchtbar anstrengend oft sind und man sie wirklich - das wird auch aufgezeichnet, ich kann jetzt nicht sagen, was man mit denen gerne macht. #00:52:33-7#

I: Ich schneide es raus, ich übersetze es nicht. Ich weiß, ich weiß, was sie meinen. Es ist, es ist wahrscheinlich auch diese, dieses, das ist ja das faszinierende an diesem PERMA, an dieser Positiven Psychologie inklusive diesem Perma teach, das es diese Wechselseitigkeit hätte, diese psychosoziale Gesundheit, Wohlbefinden, ja eigentlich in alle Richtungen dann ausstrahlt. Das ist natürlich die Henne-Ei-Frage, was zuerst da ist, und wahrscheinlich ist das warscheinlich das schwierigste an diesen Interviews, ist, dass diejenigen, die die Haltung haben, so jetzt, wie sie, sich ja auch dann zum Interview also melden und dabei sind, und da gibt es einige, die gesagt, danke, wir wollen das nicht, weil PERMA nicht unsere Haltung entspricht. Da wäre jetzt interessant herauszufinden "warum" . Genau. #00:53:28-8#

B: Also ja, ich verstehe nicht, wie man da jetzt. Ich glaube, wenn, wenn wer sagt, das möchte ich nicht, dann ist es, glaube ich, eher wegen dem, weil das mit Aufwand verbunden ist. Man muss sich damit jetzt auseinandersetzen. Man muss sich das anschauen, und viele haben oder ganz viele Lehrer haben einfach ihr Konzept, dass sie halt von Tag eins fahren, und es ist halt schwierig, da dann irgendwie was Neues aufzunehmen oder einen Zugang zu finden. #00:53:57-9#

I: Was verstört. Weil dieses Wohlbefinden in der Schule für alle Seiten schon ein Thema ist, weil alle immer gefrusteter oder einfach das, das alte System kommt oft nicht weiter? #00:54:09-5#

B: Eben. Eben. Und und das ist ja dann auch da, wo man frustriert wird, oder weil das, wenn man immer eine Schiene fährt, irgendwann funktioniert die Schiene einfach nicht, weil es nicht mit der Zeit geht und weil es nicht die Gegenwart, nicht mehr der Gegenwart entspricht, und genau, und dann reagieren natürlich die SchülerInnen anders, und das frustriert dann den Lehrer, und ein frustrierter Lehrer ist, ja, es geht dann halt weiter, und dann kann das nicht funktionieren. #00:54:42-6#

I: Genau. Ich muss jetzt ganz kurz, dass ich sie nicht zu lange strapaziere, weil wir sind schon bei einer Stunde oder kurz vorher, zehn Minuten, ähm. Schulleitung, in in ihrem Fall hat, die also die Frau E. - ist das die Direktorin? #00:54:59-9#

B: Nein. #00:55:00-1#

I: Dann aber, also die Schulleitung, also die Direktion, hat die mit diesem PERMA teach in irgendeiner Form sich eingebracht? Hat sie ihnen das Empfohlen es zu machen? #00:55:09-6#

B: Unser Schulleiter nicht. Der. Der Schulleiter hat es einfach. Frau E. hat´s ihm präsentiert oder ihm kurz vorgestellt. Und er hat gesagt: "Ja.Ja. Macht´s mal." #00:55:19-5#

I: Okay. Aber kam das auch bei ihm, oder ihr an? Der Frau. Der Schulleiterin? #00:55:24-9#

B: Nein, Das ist. #00:55:37-2#

I: Also, sie haben jetzt kein Gefühl, dass die Schulleitung sie da unterstützt, oder? Es begrüßt? #00:55:43-8#

B: Jaaaa, wir können´s machen, und er, wenn wir jetzt eine, ahm eine Fortbildung haben, dürfen wir die Fortbildung machen. #00:55:44-0#

I: Aber das fließt jetzt nicht wieder zurück in die Schulleitung, diese Themen, die PERMA. #00:55:46-5#

B: Nein, nein, dafür hat er keine Zeit. #00:55:52-3#

I: Okay, also auch keine großartige Unterstützung. Die Kollegen, sie sind ja in der Expositur. #00:55:55-3#

B: Wir machen das, wir machen uns das selbst aus! #00:55:55-3#

I: Machen, machen halt auch, was sie machen. Eine letzte Frage hätte ich noch an sie. Und zwar hätte ich so gerne von ihnen jetzt erfragt; Für was sind sie dankbar?Für was sind sie aus dieser Schulung oder dieser Fortbildung? Alles, was sie in dieser Zeit erlebt haben? Gibt es da etwas, oder können sie drei Dinge nennen, wo sie dankbar sind? #00:56:18-4#

B: Mhhm. Dankbar? Ja für die, das habe ich jetzt schon öfter gesagt, für das Bewusstsein, das dadurch geschaffen wird. Das man Lehrer, Lehrerinnen, die jetzt vielleicht nicht diese automatische Haltung haben, dazu animiert, die Haltung zu bekommen. Und ahm, auch, dass man einfach den Schülern, die Schülerinnen damit positiv verstärken kann. Und darum geht es, glaube ich, dass man ihnen ihnen eben das mitgibt und dass sie das, und das müssen sie tatsächlich lernen. #00:57:18-6#

I: Weil es natürlich zum Wohlbefinden beitragen könnte. #00:57:25-4#

B: Weil es zum Wohlbefinden beiträgt und weil sie ja, glaube ich, oft mal das von zuhause nicht mitbekommen. #00:57:35-1#

I: Genau. #00:57:37-7#

B: Das ist sehr viel wert. #00:57:37-7#

I: Gibt es noch etwas, was ich nicht gefragt habe? Was ich fragen hätte sollen? Was sie mir unbedingt erzählen wollen? #00:57:45-7#

B: Ich glaub, ich glaube, ich habe eh alles erzählt, was mir fehlt, ja, was mir dazu einfällt. ja. Das gemeinsame, ja das gemeinsame Lachen war wirklich. #00:57:58-1#

I: Ja, das habens gesagt. #00:58:01-2#

B: Ja, das war, das ist so ein Moment, an den ich mich echt gut zurück erinnere, das war. #00:58:05-1#

I: Kann man das als das Highlight abbuchen. #00:58:07-3#

B: Ja, weil, da habe ich mir gedacht, am Anfang, nein, das funktioniert nicht. Da war da, war ich so einbisschen, eine Minute, jetzt lachen mit ihnen, und dann dann war das wirklich, und dann hat ein Schüler so angefangen hat, bewusst so ur-blöd zu lachen, und dann haben wir nicht mehr aufhören können, und ich glaube, wir haben dann wirklich drei Minuten durchgelacht. Aber wirklich so, also mit Tränen in den Augen, wenn man sich vorkommt, wo kommt das jetzt her? #00:58:43-0#

I: Das ist faszinierend, was da, wie man dann doch plötzlich ganz echt und aus echten Stücken lachen kann, obwohl es am Anfang eigentlich einen Auftrag war. #00:58:52-7#

B: Ja, das war echt, das war echt toll. Die Dankbarkeit Kette fand ich auch, oder? Oder? Freundlichkeits-Kette? Dankbarkeit, Freundlichkeit Kette? Ja, es war auch schön. Ja. Einfach auch, dass die Schülerinnen gesehen haben, okay, mit so kleinen Worten können sie so viel und so großeseigentlich bewirken bei dem anderen, und dass sie halt einfach dadurch andere Menschen glücklich machen können, und das war ihnen einfach auch vorher, glaube ich, ja, darüber denken sie nicht nach, darüber reflektieren sie nicht. Aber dann, wenn man sie darauf hinweist, und das ist schon schon cool, das sie das dann auch lernen zu sehen. #00:59:44-2#

I: Ja, und wie, mit wie wenig man anderen Menschen eine Freude bereiten kann? #00:59:48-5#

B: Ja, ja, ja. #00:59:49-9#

I: EInfaches Tun, eigentlich nur etwas Aufschreiben oder Danke sagen? #00:59:53-3#

B: Ja, ja, ja. Oder einfach nur ein Lächeln. #00:59:58-3#

I: Lächeln, das ist ja die günstigste Variante, schon mal Emotionen, also positive Emotionen zu erzeugen. #01:00:07-2#

B: Ja, ja. ja. #01:00:09-9#

I: Super, ja cool! Dann sage ich ihnen ganz, ganz herzlichen Dank für ihre Zeit. Nicht nur für ihre Haltungen und auch für ihr Engagement, sondern vor allem jetzt für die Zeit, die sie uns oder dieser Studie jetzt geschenkt haben. Damit wir das noch besser und noch effizienter und jetzt auch evidenzbasiert. Das ist ja das Ziel, um noch mehr Menschen zu motivieren, um alle zu überzeugen. #01:00:34-5#

B: Ja, ich hoffe, es gelingt. Aber ich bin mir sicher. Wir werden es weiter hinaustragen und auf jeden Fall nächstes Jahr auch wieder mit dem Buch arbeiten. Ja. #01:00:46-3#

B: Bekommen wir da. Gibts da neue? Oder sind das die ? #01:00:47-7#

I: Das weiß ich jetzt gar nicht genau. Es gibt. Es gibt jetzt mittlerweile eine neue Auflage. Das weiß ich, weil ich hier in Vorarlberg Volksschulen betreue, und die wollten, denen habe ich auch davon erzählt, und die wollten es schon haben, aber da war es nur digital verfügbar. Ich weiß nicht, ob es mittlerweile nachgedruckt wurde, aber es wäre dann nur, nur eine neue Auflage. Also, ich weiß nicht, ob, da was anderes drin sein wird. Aber wie gesagt, das Padlet, ich glaube, da, das ist so eher das interaktive, da steht immer relativ viel drauf, relativ auch neue Studien, auf neue Interventionen. #01:01:24-4#

B: Mh. Alles klar, dann werde ich mir das auf jeden Fall anschauen, dieses Jahr. #01:01:28-7#

I: Da ist Material für die nächsten zehn Jahre drauf! #01:01:30-5#

B: Ja, ja, ja, super, sehr gut. Ja, dann Danke. #01:01:35-1#

I: Danke für ihre Zeit. Dankeschön. #01:01:37-6#

B: Gerne. Gerne. #01:01:38-2#

I: Alles Liebe und einen wunderbaren Sommer #01:01:40-0#

B: ja ebenfalls. #01:01:40-3#

I: mit ganz vielen positive Emotionen damit man im Herbst wieder motiviert zurück ist. #01:01:44-0#

B: Ja, Danke schön. Ihnen auch. #01:01:46-9#

I: Baba. Wiederschauen. #01:01:49-3#
