# Supplementary material for: PERMA.teach: a study on the effectiveness of a standardized positive education training program in Austria
Source: Front Psychol. 2025 Apr 8;16:1516572. doi: 10.3389/fpsyg.2025.1516572 (PMC12053473; doi:10.3389/fpsyg.2025.1516572)
Supplement: Supplementary file 2 [file Data_Sheet_1.ZIP › Material PERMA.teach/Material LPs/Interviews LPs Teil 4.zip!/Interviews Transkription/Interview 4 - transkribiert.docx]

**Interview 4.m4a**

I: Wunderbar. Geschafft. Auch diese technische Hürde konnten wir nehmen. Und dann die Namensschwierigkeit. Tut mir auch leid. Es ist leider Gottes, in der der Hitze falsch, in die Liste falsch reingerutscht. Aber ich habe es schon an oberster Stelle korrigiert. Vielen Dank, dass sie heute sich Zeit nehmen, Frau Höbartner. Darf ich sie mal kurz fragen? Ich habe gesehen, sie sind sogar Gründerin des Astrid Lindgren Campus. Wie lange sind sie denn schon als Lehrkraft tätig? #00:00:35-9#

B: Ähm. Ähm. 24 Jahre. #00:00:39-9#

I: Und diese 24 Jahre sind jetzt an dieser Schule. #00:00:44-4#

B: 20 sind hier. #00:00:45-9#

I: Okay, Okay. Ja schön. Das heißt, sie sind also, Sie sind eigentlich der Großteil ihres Lehrer-Lebens, sozusagen in ihrer eigenen Schule. #00:00:58-5#

B: Mhm. Genau. #00:01:01-6#

I: Welche Klassen unterrichten sie da? Ich habe gesehen sie sind #00:01:04-0#

B: Erste bis neunte Schulstufe. Also alle. #00:01:07-0#

I: Einmal durch. Und ... aus, ich gelesen habe, reformpädagogisch, dass es auch jahrgangsübergreifende Klasse sind. #00:01:14-1#

B: Mhm. mhm. #00:01:14-7#

I: Okay. #00:01:16-0#

B: ja genau. #00:01:16-5#

B: Genau. Wir sind ganz klein. Wir haben nur 30, maximal 30 Schüler und Schülerinnen insgesamt. #00:01:22-6#

I: Das wäre jetzt genau die nächste Frage gewesen. Weil ich. Sie sind zu viert. Sie sind ein Lehrkörper, der aus vier Personen besteht. #00:01:29-3#

B: Ja, mittlerweile sind wir sechs. #00:01:32-4#

I: Dann habe ich nicht gut, nicht gut genug recherchieren? #00:01:36-5#

B: Also, das steht auch, glaube ich, noch nicht auf der Webseite. #00:01:39-9#

I: Eben. Und die Frage wäre jetzt ganz am Anfang noch, was war ihre Hauptmotivation, überhaupt Lehrerin zu werden? #00:01:48-8#

B: Ich bin ganz, ganz, ganz quer eingestiegen. Ich bin eigentlich Diplomingenieurin für Gartenbau und wollte eigentlich als Sechsjährige immer Lehrerin werden, und als das dann immer näher rückt, nach dem, nach der Matura, war es da in Deutschland so, dass man allen abgeraten hat, auf Lehramt zu studieren, weil man keine Arbeit bekommt, und außerdem hatte mir der Gartenbau mittlerweile auch gut gefallen. Ich habe eine Gärtnerinnenlehre gemacht und dann eben studiert. Dann sind meine Kinder auf die Welt gekommen, und dann habe ich eine Kreativwerkstatt gegründet und Schulgärten gemacht, und so bin ich wieder zu Schulen gekommen, und meine Kinder waren in einer Alternativ Schule, und dann habe ich, war ich neugierig und habe die Montessori Ausbildung gemacht. Und hab dann dort geholfen, und dann habe ich nebenher einen äh, den Bachelor für Na, für für evangelische Religion gemacht, weil das ist der einzige mit dem ich die Genehmigung konnte, von der ersten bis zur neunten Schulstufe zu unterrichten, weil das über alle Schulstufen geht. #00:02:56-8#

I: Sehr clever! Okay. Also sozusagen, der gute, der gute Einfall, dass zu studieren, also das abzuschließen, weil sie damit sozusagen die Erlaubnis gehabt haben. #00:03:09-9#

B: Und genau. #00:03:11-2#

I: Super. #00:03:11-7#

B: Jetzt weiß ich nicht mehr. Habe ich die Frage beantwortet? #00:03:14-0#

I: Ja. Eigentlich schon. Als Quereinsteiger und als jemand, der mit, im Garten die Pflanzen wachsen sieht, war dann der Weg zu den Kindern wahrscheinlich nicht mehr sehr weit. Das Aufblühen. #00:03:33-3#

B: Genau. genau. #00:03:33-6#

I: Eine, ein wunderbares Bild, das für beide Seiten, also den alten Job und den neuen Job gelten. #00:03:35-9#

B: Und zwischendurch habe ich noch mit Arbeitslosen, weil ich eine Coaching Ausbildung gemacht habe, mit arbeitslosen Jugendlichen gearbeitet und so ein Training, und das hat mich sehr erschreckt, wie frustriert die aus der Schule kommen, und das waren noch mal so ein Motivationsschub auch die Schule zu gründen. #00:03:49-0#

I: Haben sie, hatten sie bei all der Ausbildung, die sie genossen haben, und bei allem, was sie gesehen haben, hatten sie Vorkenntnisse in der positiven Psychologie? #00:03:58-0#

B: Nein. #00:03:58-1#

I: Das war für sie eine neue, also eine neue, ein neues Konzept? #00:04:03-0#

B: Ja. #00:04:03-6#

I: Okay und Ähm. Was sozusagen hat sie? Wie sind sie auf diese PERMA teach Ausbildung gestoßen? #00:04:13-9#

B: Also, wir haben von wings das zugeschickt bekommen, also dass das da die Möglichkeit besteht, daran teilzunehmen, und ich finde das eine ganz feine Sache. Also ich fand das damals eine ganz feine Sache, und dann haben wir eben im Team gesprochen, und so sind wir dazu gekommen. #00:04:26-9#

I: Und nachdem sie selbst die Direktorin oder die Leiterin des Campus sind, waren sie dabei. Aber haben sie auch ihr ganzes Team mit begeistern können, oder war nur ein Teil dabei? #00:04:36-4#

B: Also, wir hatten einen kompletten Umbruch jetzt in dem Schuljahr. Das Team, das mitgestartet hat, ist nicht mehr da. Also, da war die Stimmung auch ganz schlecht, und da haben wir auch nicht so wahnsinnig viel PERMA machen können, und seit Jänner ist das Team neu, und jetzt geht es gut. #00:04:52-5#

I: Das war ja schon, aber, Sie haben schon angefangen mit dem alten Team? #00:04:57-9#

B: Wir hatten schon, genau. Wir haben schon angefangen mit dem alten Team, und die wollten dann aber nicht mehr. #00:05:05-9#

I: Lag das, ich nehm an das lag am Alter? Ich nehm jetzt an die sind in Pension gegangen oder? Oder? #00:05:10-0#

B: Nein, nein, die waren auch. Das hat einfach. Sie hatten andere Ideen, wie Schule gemacht wird, eine reformpädagogische Schule und. #00:05:19-1#

I: Ah, Okay, eine Grundsatz. #00:05:20-8#

B: Eine Grundsatzdiskussion war das. Ja. #00:05:23-3#

I: Okay, Okay. Ähm und also der mmh. äh. #00:05:30-6#

B: Aber es ist einfach. Es ist für uns ein aufregendes Jahr gewesen. #00:05:35-8#

I: Das glaube ich. #00:05:36-0#

B: PERMAteach war eigentlich gut für mich dabei, weil ich mir einfach diese Sachen immer wieder herholen könnte: mit einem Lächeln in die Schule kommen, in der Früh, egal was ist und so. #00:05:45-7#

I: Mmh. Ja, die Frage wäre jetzt, die ich hier stehen habe, darum hat es mir jetzt gerade die Rede verschlagen: der Unterschied zwischen Regelschul-Unterricht und positive Bildung wäre jetzt die Standardfrage hier, bei ihnen hätte, hätte ich jetzt gefragt, was ist der Unterschied zwischen ihrer reformpädagogischen und der positiven Bildung? #00:06:03-6#

B: Ähm, es verstärkts eigentlich noch. Also, wir machen das eigentlich sowieso, und das hat mich, das ist auch das, was mich gereizt hat. Das ist eigentlich noch so ein, das Ganze greifbarer gemacht hat, was wir eigentlich tun, und das man noch ein bisschen fokussiert, dass wir nochmal ein bisschen fokussierter sind, auch diese Dinge der positiven Psychologie und des ganzen PERMA teach ich nicht so im Alltag aus den Augen zu verlieren. #00:06:33-8#

I: Mhm, Mhm, und das war ihr, wenn sie sagen, sie haben mit dem alten Team gestartet, mit dem alten mitgemacht. Was war? Was war das? Warum? Warum haben sie angefangen mit ihrem Team? Können sie sich noch erinnern? #00:06:46-4#

B: Also, da war eigentlich noch, also ich habe das im Team vorgestellt, und wir hatten dann die die Schulung, oder nein, da gab es so ein Erstgespräch mit der Eva und der Ingrid, und das hat, da haben sie alle gesagt, ja, das ist eine gute Sache, das ist fein, und dann hat es eben in Sommerferien einfach Bröseln gegeben, und dann war, das ist gar nichtsmehr fein! #00:07:09-3#

I: Okay, und dann aber war das natürlich sozusagen nicht der Auslöser, aber es war halt dann einfach fortwährend nicht. #00:07:17-3#

B: Also, es war eigentlich, ich glaube, es war einfach die Fokussierung, dass dann noch mal klar war, wo wir als Schule eigentlich hin wollen, und dann war klar, oh da will ich ja gar nicht hin. #00:07:26-2#

I: Mhm, Klarmachung! Wahrscheinlich für sie jetzt auch gar nicht so unangenehm, mit dem neuen Team dann wirklich durchstarten zu können. #00:07:34-1#

B: Genau. Genau. #00:07:37-2#

I: Ahm, also, sie haben sich da,s ie haben davon von gehört. Sie haben sich angemeldet. Was war ihre Erwartung, an dieses Fortbildungskonzept? #00:07:47-3#

B: Ähm, die Erwartung war einmal eben dieses, noch mal genauer hinschauen, was läuft bei uns gut und was läuft also, wo können wir besser werden? Ähm, vor allen Dingen, manchmal verliert man oder verliere ich im Alltag so die Achtsamkeit im Umgang mit den Kindern und Jugendlichen, wenn es gerade zu dicht ist. Also, das war mir etwas ganz wichtiges. Und vor allen Dingen, wie kann ich auch die Kinder und wir haben dann hauptsächlich mit den Jugendlichen, also mit der ältesten Gruppe, weil das war meine, habe ich gearbeitet. Wie kann ich sie in dieser Krisenzeit unterstützen, der Pubertät. Und vor allen Dingen nach Corona und mit Ukraine-Krieg und Klima-Dings sind sie, brauchen sie wirklich viel, viel Unterstützung. Und für mich war das dann ein ganz, ganz wichtiges Werkzeug um. Also, sie hatten einfach auch dieses Heft dann in der Hand, zum Beispiel "Jugend stärken" und Ähm, das hat ihnen so ein Stück weit Halt gegeben, dass man immer, okay, wir machen jetzt, zweimal in der Woche arbeiten wir damit, und, ah, ja, jetzt wissen wir, es war zwar, sie waren zwar, es war zwar schwierig mit ihnen, weil sie, weil sie sich auch sie sind dann halt so schüchtern ist vielleicht das falsche Wort, aber ihre eigene Körperwahrnehmung und sowas, das ist ganz schwierig, und sowieso alles ist schwierig. Aber sie haben es dann doch so als Leitfaden und immer wieder, haben wir was gemacht. #00:09:23-5#

I: Ja, hat ja auch was mit Vokabel lernen zu tun, oder? Also dieses Reinspüren lernen und benennen lernen? Aber das heißt, sie haben diese, diese Hefte im Unterricht eingesetzt? #00:09:35-7#

B: Mhm. #00:09:36-3#

I: Und jetzt, ich springe jetzt einfach auf die nächste Frage. Aber würde das heißen, sie haben es nicht jetzt nur in ihrem Fach eingesetzt, sondern in der Persönlichkeitsentwicklung, oder haben sie? #00:09:46-5#

B: Wir haben es in der Persönlichkeitsentwicklung, wir haben, ich muss dazu sagen, wir haben ein ziemlich offenes Lernen, wir haben Lernwerkstätten, die, die dann ein bestimmtes Thema haben, und dann relativ viel Freiarbeit, und ich habe es eigentlich genutzt in der Tutorinnen Stunde. Das heißt immer, am Anfang ist eine ganze Stunde Zeit für eben die Belange der Kinder oder der Jugendlichen in dem Fall, und dann haben wir noch Berufsorientierung gehabt, und da habe ich das Talent-Loop, haben wir da probiert. #00:10:22-6#

I: Super! Das ist nämlich jetzt genau die Frage. Was was im speziellen haben sie eingesetzt? Talent-Loop war ihnen hilfreich? #00:10:30-8#

B: Das haben sie aber nicht angenommen. #00:10:32-5#

I: Okey, #00:10:34-2#

B: Das fand ich ganz spannend, weil ich war so begeistert davon und habe ihnen so davon vorgeschwärmt. Ich habe ihn so vorgeschwärmt, aber Sie mmm. #00:10:43-6#

I: Haben sie was anderes finden können, um diese Stärken zu ähm? #00:10:46-9#

B: Wir haben, wir haben dann selber aus unseren Berufsorientierung-Unterlagen, Sachen rausgesucht, die wir dann mit ihnen gemacht haben. Aber es ist dieses Jahr ganz, ganz schwer. Die brauchen so viel miteinander und und sich aneinander reiben, dass sie das. Sie glauben einfach, sie haben überhaupt keine Stärken, und das war ist wirklich Knochenarbeit im Moment. Das ist wirklich, und das ich habe, jetzt habe ich mit einem Lehrerin gesprochen, wenn sie so sind, dann dann weiß ich überhaupt nicht, wie soll ich mit ihnen tun. Habe ich sagt, sie haben eigentlich ein großes Vertrauen zu uns, dass sie sich so aufführen, dann kann man sie ja auch abholen, weil sie weinen, dann, weil irgendwer nicht mehr verliebt ist und und und oder gemein ist oder sonst irgendwas und rutschen in dieses Kleinkind sein ab, und ich denke, es ist ein großer Vertrauensbeweis, dass sie das hier sich trauen, mit allen Herausforderungen, die wir als Erwachsene damit haben. #00:11:49-6#

I: So ist es.So ist es. Das ist aber interessant, dass sie manche Dinge dann in dieser Form nicht annehmen wollen, die jungen Menschen. Hat es andere Sachen geben, wie z.B. die Apps die da ja waren immer wieder, immer wieder digitale Geschichte? #00:12:04-4#

B: Die wollten überhaupt, wobei sie sonst dauernd vor dem Ding hängen, aber sie wollten überhaupt nicht in diese digitale Welt einsteigen. #00:12:11-0#

I: Ah Interessant, okay. #00:12:12-8#

B: Das fand ich auch, weil wir kämpfen, sonst immer die Handys müssen abgegeben werden. Da hab ich gesagt, ihr dürft eure Handys holen. #00:12:19-0#

I: Interessant, genau die gegenteilige Reaktionen eigentlich. Überraschung. #00:12:24-4#

B: Also, das war wirklich das, dass sie viel mehr das Gespräch gesucht haben, und das Miteinander und und ohne ein Medium dazwischen. Also, das war eine sehr spannende Erfahrung, die ich gemacht habe. #00:12:38-2#

I: Das finde ich jetzt auch ganz spannend. Aber das heißt auch, dass sie das Tablet zum Beispiel oder das Padlet, da war ja, die, da gibt es einen Padlet permanent. Haben sie das verwenden können, für sich. War das hilfreich? #00:12:52-4#

B: Ich habe ab und zu mal reingeschaut, aber wir haben uns wirklich hauptsächlich an diesem "Jugend stärken" entlang gehantelt, weil das war das, wo wir sie auch herholen konnten. #00:13:02-9#

I: Aha. Mit Buch und mit. #00:13:03-3#

B: Ja, irgendwas in der Hand und. #00:13:06-9#

I: Das ist doch nett. Ahm. Würden sie diese Fortbildung anderen empfehlen? #00:13:12-6#

B: Sehr! Ich würde sie ganz wirklich ans Herz legen. #00:13:15-9#

I: Okay. #00:13:16-3#

B: Aber auch sonst auch, wenn man glaubt, man kann das eh schon alles, oder ich kenne das eh schon alles, dann kann ich, es sind so viele Inputs, neue dazu gekommen und dieser Austausch auch mit untereinander mit den anderen Schulen oder die Sachen, die man, die, die ich gehört habe, also ich kann das wirklich jedem nur empfehlen. Das war sehr fein, auch die beiden Moderatorinnen, und manchmal waren ja auch andere noch Mitarbeiter. Das war wirklich bereichernd. #00:13:42-9#

I: Genau, das wären jetzt noch die einzelnen Fragen, noch auf die Schulunsgunterlagen, also das Modul eins und Modul zwei, haben sie wahrscheinlich auch? War das online, oder haben sie das vor Ort gemacht? #00:13:53-9#

B: Modul Eins war das, Moment, das war das erste. Da waren sie in der Schule, da waren die Eva und die Ingrid in der Schule, genau! #00:14:01-9#

I: Mhm und aber das eine Mal, also vor Ort, und das zweite war dann online. #00:14:09-2#

B: Die anderen waren oline. #00:14:09-6#

I: Hat´s da Schwierigkeiten gegeben, oder hat das gepasst? #00:14:12-1#

B: Das hat wirklich alles gut gepasst, und dieser Austausch beim, also wo sie vor Ort waren, war auch gut, und auch zwischendurch immer diese Betreuung, weil wir. Ich habe dann immer wieder geschrieben, ich kann dieses oder jenes nicht machen, weil das Team will nicht, und das war eben also, da habe ich ein offenes Ohr einfach auch gehabt. #00:14:28-8#

I: Also wurden sie auch noch, also unterstützt? #00:14:30-4#

B: Unterstützt und genau, und wenn ich was brauche, soll ich mich melden und so. Das war wirklich fein. #00:14:35-6#

I: Also, ich habe jetzt da nur mal meine Liste: also der fachliche Input hat gepasst, das schriftliche Handout war ihnen sehr hilfreich, und weiteren die weiteren Inspirationsquellen, das Padlet haben sie zwar verwendet, aber jetzt die Kinder nicht. #00:14:50-2#

B: Aber ich fands schon fein. Also, das Padlet ist wirklich ein gutes Hilfsmittel, aber wir haben es halt nicht umgesetzt, weil #00:14:55-9#

I: Man muss ja nicht alles alles gleich. Es ist ja noch länger da. Aber es ist natürlich auch oft der overkill an Informationen, oder? Wenn sie ein Buch haben, ist natürlich oft einfacher, das eins zu eins dann. #00:15:10-6#

B: Ja, ja und und es hat mich wirklich erstaunt, aber sie haben kaum am Computer gearbeitet, dieses Jahr, also, das war witzig. #00:15:17-9#

I: Ja, andere Bedürfnisse, interessant. Kurze Frage zu den Rahmenbedingungen, also zur Einladung, zur Erinnerung, zur Terminisierung. Hat das so gepaßt? #00:15:29-0#

B: Das hat bestens funktioniert, weil ich habe dann immer mal vergessen reinzuschauen in den Kalender, und dann kam das aufgeblinkt, und dann hab ich "oh, zum Glück" und auch rechtzeitig genug die Erinnerung und auch sonst. Ich habe das beim. Wir hatten jetzt noch ein Interview mit der Schülerin, und da war, da, war sie einmal krank, und einmal habe ich dann einen Termin eingeschoben bekommen. Das hat eben eine unserer Jüngsten gemacht, weil die Großen wollten kein Interview machen, und sie hat gesagt, nein, ich mache das, und das war auch ganz problemlos, diesen Termin zu verschieben. Zweimal. Also, da war ich, das war, weil das ist ja nicht einfach. #00:16:09-0#

I: Eben, das ist doch einiges. Aber wenn sie sagen, das hat gut, gut funktioniert. #00:16:13-7#

B: Also, ich kann mich überhaupt über nichts beklagen. #00:16:16-3#

I: Gibt es etwas, was sie sich noch gewünscht hätten, zu den Modulen? Hat etwas gefehlt, wo sie das Gefühl hätten, das hätte ich jetzt gerne noch gehabt? #00:16:25-7#

B: Also, es wäre noch mal eins so vor Ort fein gewesen, wo man so wirklich in der Gruppe sitzt und sich austauscht, weil übers Web/Zoom ist es doch nochmal anders. #00:16:34-1#

I: Mhm. Also #00:16:35-5#

B: Wobei das was was ein guter Input war, dass wir das im Team, also dass wir in der Schule bleiben und das als Team gemeinsam machen und dann auch uns austauschen können. #00:16:45-1#

I: Das man so ein inneres Team hat? #00:16:48-5#

B: Genau. genau. #00:16:49-2#

I: Und das war warscheinlich auch das Stärken-Cafe? Das dann #00:16:54-0#

B: Ja, das war bei den Stärken. Mhm. #00:16:55-4#

I: Ok. Weglassen: Hätte es etwas gegeben, was man weglassen hätte können? War etwas zu viel, wo sie sich gedacht haben? #00:17:02-2#

B: Nein, am Anfang habe ich gedacht, mit dem, am Anfang kamen ja die diese Impulse. Wie heißen die noch schnell? Das, was ich jeden Tag machen kann? #00:17:10-8#

I: Ja. Also die .... PERMA Post. #00:17:11-9#

B: Und am Anfang. Jeden Tag irgendwas, aber dann habe ich angefangen, das wirklich jeden Tag zu machen, und das war fein. Das hat aber nur damit zu tun gehabt, dass ich so viele andere E-Mails bekomme. #00:17:25-1#

I: Ja, und dass man dann die guten von den schlechten Emails ein bisschen trennen muss. #00:17:29-8#

B: Genau, Aber das war dann nach einer Woche, war das erfrischend, weil ich dann eben immer was hatte, wenn ich die anderen Emails hatte, und jetzt kommt etwas zum Aufmuntern. #00:17:40-3#

I: Ja. Eine, eine neue Gewohnheit. #00:17:42-7#

B: Mmh. #00:17:43-5#

I: Weil wir gerade voher beim Stärken-Cafe waren, gab es da etwas, was sie sich als Highlight mitnehmen konnten? Gab es da ein paar, auch Anregungen durch ihr Netzwerk? #00:17:54-0#

B: Ich hab, also das, was ich was, was fein war, weil das mit den, wo alle Schulen etwas vorgestellt haben, und da habe ich viele Inputs mitnehmen können. Da war das eine mit dem, wo man ein Lied und dann dazu was arbeiten. Also, das ist noch im Plan, dann für nächstes Schuljahr. Ich habe mir jetzt aufgeschrieben, also, da waren einige Inputs, die, die richtig gut waren. #00:18:18-3#

I: Die man dann für sich selber aufgreifen kann? #00:18:19-5#

B: Weil, das war gerade ein Zeitpunkt, wo ich überhaupt nichts ein, nicht wirklich was einbringen konnte, und da war das aber auch fein. #00:18:28-8#

I: Wir haben gerade von der PERMA Post gesprochen, oder ich habe von der PERMA Post gesprochen: sie haben täglich etwas bekommen. Das war in diesem Fall für sie anfänglich etwas nervig, weil so viel im Posteingang war, aber dann mit der Zeit eine nette Gewohnheit wurde? #00:18:46-2#

B: Ja. #00:18:46-5#

I: Ja, das hab ich schon gefragt. Und Ähm, eben, da waren wir beim, waren wir schon beim Padlet, da war mir schon. Ähm. Gibt es eine Lieblingsübung? Gibt es etwas, das sie wie ein Ritual bei ihnen in der Schule eingesetzt haben? Oder etwas, wo sie als Lehrerteam haben? Etwas mit dem sie PERMA sichtbar machen? #00:19:10-0#

B: Einmal immer dieses, was wir gemacht haben, auch mit den Jugendlichen, aber auch, was ich auch gerne mit mit allen mache, wenn sie ganz unruhig werden, einfach mal zu sich kommen, atmen und dann so die Mitte spüren und und einfach mal zur Ruhe kommen, dass man ja oft und dass, wenn ich zur Tür reinkomme, Lächeln, und das bringt an manchen Tagen so viel. #00:19:38-4#

I: mmh. Also Lächeln als Ritual. #00:19:45-6#

B: Bitte? #00:19:46-8#

I: Lächeln als Ritual? #00:19:46-8#

B: Genau. Dass, wenn ich ihn zur Tür reinkomme, in einen Raum, dann lächele ich. Und am Anfang "Oh, Wieso müssen wir jetzt lächeln?" "Probiert es einfach mal!" #00:19:56-1#

I: Probieren geht in diesem Falle über Studieren. #00:20:00-4#

B: Also, das ist, das ist ein ganz wertvolles Ritual, was wir, was wir eigentlich das ganze Schuljahr über gemacht haben. #00:20:07-1#

I: Mhm, super. Ahm. In ihrem Fall haben sie das bei allen Klassen angewandt, haben sie gesagt. Also in den älteren Klassen, aber sie sind. #00:20:15-7#

B: Das haben wir auch bei den Jüngeren mit dem Lächeln. #00:20:18-3#

I: Genau, aber ich habe verstanden, dass sie es bei den Älteren, also bei der Jugend, stärker eingesetzt haben. Liegt es daran, dass sie das nicht selber machen, oder liegt es einfach daran, dass? #00:20:32-2#

B: Nein, beim Kinder stärken waren eigentlich die anderen beiden eingesetzt, und nachdem die nichts gemacht haben. Aber ich habe dann, ich habe sie sowieso immer, also wir sind jeden Tag immer in allen Gruppen, und da haben wir einfach auch Sachen, also das auch gemacht, zum Beispiel. #00:20:48-9#

I: Also, das heißt, sie haben, haben gesagt, sie haben das in den Tutorial-Klassen eingebaut. Gibt es so was wie die Chance, dieses PERMA Denken oder PERMA Handeln in den Unterricht per se einzubauen? #00:21:03-6#

B: Da tüfteln wir jetzt im Sommer dran, wie wir das jetzt umsetzen können, weil, weil es uns also, die jetzt da sind, allen gut gefällt, und, ähm, es gibt eben einfach ich denke mal, ähm, also, wo es, wo es sicher gut ist nächstes Jahr, um, um die Jugendlichen zu stärken, ist in der Berufsorientierung, also da wollen wir es viel mehr einsetzen, nämlich wie kann ich die Dinge positiv sehen, wie kann ich auf etwas zugehen, und wie kann ich, wie verändert sich auch etwas, ob ich das positiv oder negativ sehe, und vor allen Dingen mich selber. Ja, wenn ich immer sage, ich kann nicht dann so herauskitzeln, was kann ich denn gut, nämlich zu schauen, was, das haben wir auch gemacht, was kann ich den anderen zum Beispiel? Wir haben manche Mehrsprachigen. #00:21:53-5#

I: Hm! #00:21:54-2#

B: Und dann sagen wir, ja, du kannst ja, könntest uns ja rumänisch oder serbokroatisch oder sonst was jetzt beibringen oder so, also diese Sachen einfach, an die sie gar nicht denken, herauszuholen, und ähm, da war dann auch der Wunsch von den Jüngeren, dann lasst uns das doch bitte nicht mehr Stundenplan, sondern Werkstattplan nennen und auch nicht mehr Lernbüro, sondern Begeisterung-Werkstätten, und dann fühlen wir uns schon viel besser! #00:22:22-8#

I: Okay, also ein, ein, ein, ein, ein Umbenennen! #00:22:27-4#

B: Ja, das hat schon, also, ich hätte das nicht gedacht, weil bei den Jüngeren ist es eigentlich gar nicht so schwierig gewesen. Aber es hat gerade die Anna, die dann auch das Interview gemacht hat, die hat da sehr darauf bestanden, und es hat was verändert, einfach durch die anderen Namen, mein Stundenplan, das hört sich so streng an, und Werkstatt Plan ist wieder was ganz anderes, hat sie gemeint. #00:22:54-4#

I: Mhm also, das heißt, sie spüren diese positiven Veränderungen in der ganzen Schule, im ganzen Campus, mit unterschiedlichen Wortmeldungen, unterschiedlichen Bezeichnungen. #00:23:05-2#

B: Und wir haben auch jeden Tag, jeden Tag nicht, jede Woche einen Montags- Parlament, da können sie, da hat sie das dann eben für alle eingebracht, und dann haben wir einen Stundenplan-Team und so was gemacht. Also das, und das hat, da habe ich gesehen, das hat richtig Freude gemacht durch diese, einfach eine Wort Veränderungen, und das habe ich mir auch damit genommen, dass man eben mit diesem positiven Wörter finden für Sachen. #00:23:29-1#

I: dass das auch Begeisterung schafft? #00:23:32-4#

B: Auch auf diesen, den Schulalltag betrifft, und dass wir an Dinge gar nicht denken, die eigentlich den Kindern oder Jugendlichen wichtig wären, und das ist ja leicht, einen Begriff am Computer auszutauschen und dann neu auszudrucken. #00:23:47-0#

I: Wie schön das, dass sie so offene Gemeinschaft leben, dass da sozusagen die Kinder sich wirklich auch unmittelbar so einbringen können im Montags-Parlament und und ihre #00:23:57-4#

B: Ja. #00:23:57-7#

I: Ahm. Wünsche platzieren können. #00:23:59-4#

B: Genau also, wir sind eigentlich bis zu dem Relationships gekommen, und das andere machen wir jetzt wirklich verstärkt nächstes Jahr noch. #00:24:11-3#

I: Mhm. #00:24:12-6#

B: Bei dem PERMA, das andere so ein bisschen angerissen, aber nächstes Jahr, glaube ich, sind sie auch so weit, dass sie dann also gerade die Jugendlichen, dass sie da aufnahmefähiger mmh, dann ist auch, die Schulzeit ist langsam zu Ende. Dann muss ich mal überlegen, wo soll es hingehen, und dann brauche ich das, dass ich positiv bin und auch von mir überzeugt bin und dass ich dem ganzen einen Sinn geben kann. #00:24:36-9#

I: Da startet man besonders besonders besser durch, wenn man an sich glaubt oder ziemlich weiß, was sind meine Stärken, und wo möchte ich die einsetzen? Was wäre, würden sie sagen, war einer der schönsten Veränderungen, die sie im Klassensystem, in der Wahrnehmung mit den Kindern durch PERMA erlebt haben? #00:24:55-8#

B: Ähm, wir hatten jetzt Projektwoche, und da fahren wir alle zusammen weg auf eine Burg, wo wir uns selbst versorgen, fünf Tage lang, und die dieses, eben dieses, ich gehe durch eine Tür und Sag: "Lächeln" war sehr fein, und dann einfach so ein besonderes Miteinander, nämlich auch, dass ich mich für jemand anders engagiere, dass ich mich, wenn es Streit gibt, auch nicht, also auf dieser Beziehungsebene, wenn es Streit gibt, arbeiten kann. Also z.B. aber wieso müssen wir jetzt kochen und jetzt machen, da auch die Sinnhaftigkeit hinter dem allen zu verstehen, warum muss ich das machen? Also, da sind ganz viele Sachen für uns so eingeflossen, und wir hatten uns eben mit dem neuen Team auch nochmal zusammengesetzt. Das war eigentlich so ein riesen, ein großes PERMA Projekt für uns diese ganze Woche, weil da alles mit und ineinander einfließt, und eben auch die jüngsten bis zu den ältesten einbezogen sind. Ja. #00:26:08-6#

I: Ja, das klingt nach einer Herzens-Woche. #00:26:13-5#

B: Und dann ist ja auch das, wie heißt es gleich, dass ich Erfolg habe und dafür aber auch eine Leistung erbringe? Das ist das A, glaube ich, bei PERMA #00:26:24-5#

I: Ja, Accomphlisment. #00:26:24-6#

B: Wir sind sechs, also wir sind zu Fuß von der Schule aus losgegangen, und wir sind sechs Stunden dahingewandert zur Burg. Also wir hatten nicht das Gepäck, das Gepäck haben die Eltern voher gebracht, also wir hatten nur die Rucksäcke, und ähm, es war dann ein Gejammer zwischendurch und das. Als wir aber dann auf der Burg waren, waren alles gut, und das ist eigentlich auch das. Da haben sie das auch, dass ich, dass ich eine Leistung bringen muss, um dorthin zu kommen und dann eine gute Zeit zu haben. #00:26:53-3#

I: Und das, mit einer Freude erfüllt, wenn man´s erreicht. #00:26:56-5#

B: Genau. genau. Weil, da waren sie so glücklich. #00:27:00-3#

I: Ach wie schön! #00:27:01-6#

B: Und es gab ein gutes Mittagessen und ja! #00:27:04-9#

I: Was ist die schönste Veränderung die sie, die sie an sich selbst wahrgenommen haben? Gibt es da etwas?. #00:27:11-5#

B: Die schönste, das ist auch dieses Lächeln und einfach dieses ähm, ähm, dieses wirklich das Wahrnehmen des Positiven. Was ist auch, wenn, wenn gerade alles schief läuft, was kann ich mir positives rausholen? Wo kann ich den Fokus drauf geben? Und und und dann wieder einfach auch den Kindern und Jugendlichen gegenüber positiv begegnen, einfach zu schauen, nicht auf das schlechte, sondern auf das, was gut läuft. #00:27:41-0#

I: Und was ist das besondere am Umgang mit ihren, mit ihren Arbeitskollegen im neuen Team? Hat sich da was für sie herauskristallisiert, dass sie auf PERMA zuschreiben würden? #00:27:54-2#

B: Ähm, ja, weil ich hab, ich hab mehr Achtsamkeit, dann, weil das hat sich ja, die sind alle neu zusammen, und und wir waren eigentlich, es war so mehr Achtsamkeit auch drauf, dass ich vorsichtiger bin. Ähm, um zuzuhören, wo stehen die anderen?was wollen sie, und wie geht das gemeinsam? Und das hat eigentlich wunderbar funktioniert. Also, diese Achtsamkeit habe ich mir mitgenommen, und auch dieses, bei allen zu schauen oder miteinander zu schauen, wenn Herausforderungen sind, was können wir, was? Wie können wir´s einen? Jedes positive hat auch, jedes negative hat auch irgend etwas Gutes an sich, und was können wir daraus? Wie können wir das rausnehmen? Und das war zum Beispiel das, wo wir, wo sie alle gesagt haben: "Oh, mit diesen Jugendlichen, dass ist so herausfordernd, wir mögen sie, aber ..." und da hab ich gesagt, seht es einfach als Vertrauen in uns. #00:28:58-3#

I: Und dass ist dann ja schon wieder positiv. #00:29:02-3#

B: genau. genau. #00:29:03-2#

I: Wenn sie jetzt ihre Veränderungen, die sie bemerken oder die sie mir da jetzt beschreiben, in diese Kategorien von PERMA packen würden? Was hat sich denn bei ihnen, oder wie schaffen sie es, bei ihren Mitarbeitern dieses P wie positive Emotion ähm verstärkt zu aktivieren? #00:29:25-5#

B: Ähm, einfach, ich habe eigentlich, ich schaue, dass ich immer, dass wir morgens irgendwie immer eine Plauder-Zeit haben, also wo wir einfach mal kurz alles bereden oder oder einfach nur auch: "wie geht es dir?" "und was macht ihr?" Dann bei den Teamsitzungen haben wir eingeführt, einen Punkt: Wohlbefinden, dass das erstmal ganz am Anfang steht. Wir nehmen uns dann 20 Minuten Zeit. Wie geht es dir und was macht dein Wohlbefinden aus? Ähm, und wir haben auch Supervision eingeführt, einfach, also, die hatten wir vorher auch schon, aber jetzt einfach in einem dichteren Takt. #00:30:15-6#

I: Mhm. #00:30:16-6#

B: Wo wir auch da den Fokus haben, wo sind unsere Stärken? Wie, wie, wie können wir also positiv auf die Dinge zugehen? #00:30:24-4#

I: Und auch das, E im Engagement stärken, wenn man die die richtigen Stärken einsetzen kann? #00:30:30-9#

B: Genau wie kann ich dann meine Stärken? Weil wir ganz bunt gemischt sind, und wir, manche haben ein Lehramt, manche nicht. Wie können wir uns gegenseitig auch inspirieren mit dem, was, was unsere Stärken sind? Und auch die Beziehungen untereinander sind auch wichtig, und da aber auch zu schauen, wir sind nicht verheiratet miteinander, wir sind auch nicht eine Familie, sondern wir sind Arbeitskolleginnen und -kollegen, und wenn wir uns gut verstehen, ist das sehr fein. Aber man muss immer schauen, wir sind trotzdem ein berufliches Team und nicht ein Familienteam, oder #00:31:09-9#

I: Die Rolle, die Rollen definieren! #00:31:12-6#

B: Genau! #00:31:13-5#

I: Aber das heißt trotzdem, ich habe jetzt ganz auch viel rausgehört, was natürlich in das R für Relation einzahlt. Die Plauder-Zeit. Oder einfach diese, positiven Emotionen und Beziehungen gehen ja Hand in Hand. Gibt es etwas, was, was sich beim Meaning, also beim Sinn ihres Tuns, mmmh verändert hat? #00:31:32-8#

B: Ähm, Also ich muss dazu sagen, ich werde in vier Jahren aufhören, weil ich dann einfach alt genug bin und was sich da für mich geändert hat im Sinne ist, nochmal zu reflektieren, wie möchte ich das Ganze übergeben. #00:31:51-9#

I: Mmh. #00:31:51-9#

B: Also wie möchte ich diesen Weg gestalten, dass es gut ist und dass das, was mir wichtig ist, auch rüberkommt und sich aber auch verändern kann gleichzeitig, und das ist eigentlich der Sinn, den ich in dieser Arbeit sehe, und das hat sich einfach noch mal. #00:32:08-1#

I: Ja, das ist doch schön! Schöner Sinn für sie. Das sie sozusagen ihr legacy, also ihre Verlassenschaft sozusagen. Das ist einfach, dass das dann zurückbleibt, ordentlich geordnet, mit gutem Spirit. #00:32:22-1#

B: Ja genau. #00:32:25-0#

I: Und A wie Accomplishment. Also die kleinen Ziele. Können sie sehen oder können sie ihre Schüler sehen oder ihre Mitarbeiter? #00:32:33-4#

B: Eigentlich sehe ich uns alle bei den kleinen Zielen, also wir, also auch auch unsere herausfordernden Jugendlichen, in kleinen Schritten schaffen sie dann doch so viel, und auch als Team haben wir unglaublich, weil das hätte ja auch in die Hosen gehen können, wenn man zu fünft auf einmal neu zusammen ist, ähm ja, zu sechst, und das, da können ja auch Persönlichkeiten aufeinander treffen, die überhaupt nicht miteinander können. Und und in Zeiten wie diesen gute Lehrkräfte zu finden, ist nicht so einfach, und da haben wir einfach ein Riesenglück gehabt. Und: Ähm fürs Team, die bringen eine ganz große Leistung, dass sie das sich so ins kalte Wasser stürzen. Und ja, es ist auch immer das jeden, das tägliche, es geht immer einen Schritt weiter. Es wird immer, es kommt von den Kindern ein neuer Impuls, oder wir haben auch viel Elternarbeit. Also von den Eltern kommen ganz viele positive Rückmeldungen, und das ist einfach also die Leistungen, die wir hier erbringen, weil das ist ja, Lehrerinnen sein, ist ja nicht nicht einfach, und die werden auch gesehen, und das tun ich auch immer wieder bei Eltern an, und so #00:33:57-2#

I: sage ich das auch immer wieder, und das ist mir auch wichtig, dass das auch gesehen wird. #00:34:01-9#

I: Okay, wenn wir gerade bei den Eltern sind. Oder Eltern sind vielleicht gar nicht so außenstehend. Wenn jetzt jemand Außenstehenden kommen würde, woran würde er denn bemerken, dass sie und ihr Team anders Unterrichten, anders Lehrkraft sind, seit sie PERMA erlebt haben? #00:34:21-4#

B: Ähm, seit wir PERMA erlebt haben? #00:34:24-6#

I: Gibt es da einen nennenswerten Unterschied? #00:34:28-6#

B: Also, wir haben immer wieder Hospitationen, und und was mir dann mich dann immer sehr verblüfft, weil wir unser Aufnahmeverfahren so ist oder auch manchmal Praktikantinnen kommen, ist, ähm, dann denke ich immer, dieser Tag heute war echt so in die Hosen gegangen, und von den Eltern mit den bei den Nachgesprächen, da kommt eigentlich fast immer die Rückmeldung, dass sie am Anfang ein bisschen chaotisch gefunden haben. Aber sie sind verblüfft, wie wohlwollend und wie fein alle miteinander umgehen. Das haben wir, glaube ich, nochmal durch PERMA nochmal verstärkt. Also, das ist immer so, dass dass sie das überhaupt nicht gewohnt sind, dass man die Kinder untereinander, ihre Konflikte auf so eine gute Art lösen, aber dass auch die Beziehung zu den Pädagoginnen einfach so auf eine Art und Weise ist, die, die sie verblüffend finden. #00:35:28-4#

I: Überraschend, weil sie es einfach so nicht kennen? #00:35:32-9#

B: Und ja sonst? PERMA von außen? Wir haben ein paar Sachen geändert in den Räumen, die wichtig waren, damit man das PERMA besser #00:35:43-8#

I: sichtbar machen kann, oder? #00:35:46-7#

B: Ja und auch besser leben kann. #00:35:48-6#

I: Okay, was waren das für Veränderungen? #00:35:50-0#

B: Das waren manchmal die Tischordnung, einfach zu schauen, wie das, also auch, dass die Kinder oder Jugendlichen sich das selber machen, wie sie gerne sitzen wollen. Und wir haben auch so flexible Tische, also sie können die zusammenklappen, dann kann nur einer da sitzen oder aufklappen, dann können bis zu vier dran sitzen, und das war bei den Jugendlichen ganz wichtig, das war ganz spannend. #00:36:16-2#

I: Sich´s selber richten konnten, so wie sie es brauchen. #00:36:19-2#

B: Ja. Und dann auch mal wieder verändern, weil es dann doch nicht gepasst, weil das ist jetzt nicht mehr meine Freundin, und auch ausprobieren konnten, dass ein sechser Tisch vielleicht nicht das beste ist, dass das von den Räumlichkeiten her, konnte man schon sehen. #00:36:37-5#

I: Jetzt hätte ich hier einen ganzen Block für die Fragen über die Schulleitung. Aber nachdem sie die Schulleitung sind: Ähm, es ist ja oft oft so, dass, wenn ein Lehrer Theam in einer größeren Schule etwas macht, dann ist es ganz oft schwierig, ohne dem Leiter die nötige Unterstützung dafür zubekommen. Jetzt in ihrem Fall sind, sind wir direkt bei der Direktion, bei der Leitung. Können sie sich? Könnten sie sich vorstellen, was sie ihren Direktor, Direktor-Kolleginnnen raten würden oder empfehlen würden? Oder was würden sie sich wünschen, was die tun bei anderen Teams? #00:37:20-4#

B: Ähm, dem Team vertrauen. #00:37:26-3#

I: Mhm. #00:37:27-8#

I: Dass das gut ist, was sie machen, und einfach auch mal arbeiten lassen, und aber nicht so nach dem Motto ja ist egal, was ihr macht, sondern Wertschätzend auch dann vielleicht mal auch bei einem Projekt oder bei einem, bei einer Stunde oder bei mehreren mit mitschauen und schauen, wie es geht, und eine Rückmeldung geben, aber grundsätzlich mal den Team vertrauen, dass sie es gut, dass sie es gut machen. #00:37:52-3#

I: Mhm. #00:37:53-2#

B: Und Hilfestellung geben, wenn irgendwas nicht so klappt, wo man dann einfach so als Moderatorin vielleicht dabei ist. #00:38:01-5#

I: Mhm, Mhm, #00:38:03-2#

I: Okay, ja, und dann bleibt eigentlich nur noch meine Frage, eine PERMA-Frage an sie. Nennen sie doch bitte drei Gefühle, die sie mit PERMA jetzt als, als Ausgebildete sozusagen, in Verbindung bringen. Und was ist da wichtig? #00:38:21-7#

B: Fröhlich sein. #00:38:23-9#

I: Mhm. #00:38:25-0#

B: Ähm, Mut haben und auch entspannt sein! #00:38:33-3#

I: Mhm, sensationell! Ich danke ihnen herzlich. Gibt es noch etwas, was unbedingt gesagt werden muss? Und was sie unbedingt noch loswerden wollen zu diesem Ausbildungskonzept? #00:38:50-0#

B: Ich habe es vorhin schon gesagt, aber ich möchte einfach sagen, ein großes Danke sagen, und das war wirklich ein etwas feines und hat mich auch durch diese schwierigen Zeiten getragen, weil ich auch immer wieder zur Eva und zur Ingrid Kontakt haben konnte, und ich wünsche einfach möglichst vielen, dass sie das auch machen. #00:39:10-6#

I: Sensationell. Danke ihnen vielmals von Herzen für ihre Zeit, fürs Mitmachen, für ihre Begeisterung, fürs Verbreiten, und ich danke ihnen, dass sie mir dabei helfen, dass wir die Studie füttern dürfen mit guten Inputs und Ideen, wie man die Dinge noch noch besser machen könnte. #00:39:36-4#

B: Das ist ganz, ganz wichtig, Und da danke ich ihnen auch für ihre Arbeit, weil dadurch verbreitet sich das ganze, und dadurch wird es fundierter, für manche, die zweifeln. Das ist nämlich ganz fein. #00:39:48-3#

I: Nein, das ist wunderbar, und ich finde auch, also, wenn man mit dem mit dem Gedanken von PERMA in Berührung kommt, dann ist es egal, ob das PERMA teach. Es gibt ja in der, ich komme jetzt eher aus der Business-Welt, das heißt das, PERMA lead. Da machen wir dasselbe mit Mitarbeitern, was sie jetzt mit Schülern machen. Und ich, und das ist eigentlich mein Weg gewesen, weil ich dort so erstaunt war, dass manche Menschen so viele Dinge nicht können, habe ich mir gedacht, da muss man doch in die Schulen schauen, weil dort könnt man es doch viel besser lernen, als später als 30ig jähriger und dann bei Burnout Prophylaxe zu landen. Also, es wäre doch schön, wenn man das vorher, so als Rüstzeug mitbekommen könnte fürs Leben, und darum danke ich ihnen, dass sie das tun. #00:40:30-3#

B: Sehr, sehr gerne. Es macht viel Freude. #00:40:33-2#

I: Danke Ihnen vielmals. #00:40:33-2#

B: Okay, danke für das schöne Interview. #00:40:34-8#

I: Ich danke ganz herzlich, für ihre offene, offenen Antworten, und wir sehen uns sicher wieder einmal oder hören uns. #00:40:42-9#

I: Danke, wiederschauen, baba! #00:40:47-1#
