# Supplementary material for: PERMA.teach: a study on the effectiveness of a standardized positive education training program in Austria
Source: Front Psychol. 2025 Apr 8;16:1516572. doi: 10.3389/fpsyg.2025.1516572 (PMC12053473; doi:10.3389/fpsyg.2025.1516572)
Supplement: Supplementary file 2 [file Data_Sheet_1.ZIP › Material PERMA.teach/Material LPs/Interviews LPs Teil 4.zip!/Interviews Transkription/Interview 2 - transkribiert.docx]

**Interview 2.m4a**

I: Wunderbar! Ich darf nämlich im Zusammenhang mit der Uli Lichtinger, die PERMAteach Ausbildung evaluieren und da sind wir mehrere Menschen, die in dieser Studie mitarbeiten dürfen. Und ich darf dies wiederum für meine Masterarbeit verwenden. Also, es ist eine Win.Win-Situation. Und ich freue mich sehr, sie in diesem Zusammenhang kennenlernen zu dürfen. Und da hätte ich gleich eben ein paar Fragen. Mal rein, wie gesagt statistisch, nämlich wie lange sie schon Lehrperson sind, sie als Lehrkraft an dieser Schule, an der sie jetzt gearbeitet haben - Sie sind jetzt ja im Mutterschutz, aber da sind sie ja trotzdem noch angestellt - und welche Klassen sie unterrichten? #00:00:45-1#

B: Ich bin seit sechs Jahren Lehrerin und unterrichte Klassen, ja ich wandere da halt so mit, so, wie es ist. Jetzt, das letzte Jahr habe ich eben das PERMA Projekt in einer ersten Klasse begonnen, die mittlerweile eine zweite ist, und: Ähm, hab auch in einer dritten und vierten unterrichtet. Also bei uns in der Schule zumindest ist das System immer so, dass man, dass man, wenn man eine erste hat, hat man auch eine dritte, und dann springt man immer mit zweiten Klassen, oder man hat eine zweite und eine vierte. Ja, aber. #00:01:18-9#

I: Schön, und die Frage wäre jetzt, was hat sie motiviert, Lehrer, Lehrperson zu werden? Was ist, was war ihre Grundannahme, damals zu sagen, ich möchte gerne Lehrerin werden? #00:01:31-9#

B: Also, ich bin eigentlich Quereinsteigerin. Ich habe internationale Betriebswirtschaft studiert und hatte eher in Projektleitung, mit Forschungsförderung zu tun, und das waren gerade Projekte, eigentlich, die mit Schulen abgeschlossen wurden. Und während ich der Projektleiterin war und alles sehr administrativ war, habe ich mir damals immer gedacht, ich sitze am falschen Ende der Arbeit, wenn ich über Projektberichte lese und die Arbeit mit Kindern sehe. Das war eigentlich der der zündende Funke, dass ich mir gedacht habe, ich bin falsch in dem rein administrativen und bin über das Programm "teach for Austria" - ich weiß nicht, ob ihnen das was sagt - bin dann in die Schule gekommen. Habe dann auf der PH das Lehramt nachgeholt, und ja, liebe bei allen Anstrengungen auch den Job immer noch. #00:02:29-1#

I: Sind sie am richtigen Ende angekommen? #00:02:32-0#

B: Am richtigen Ende, ja. Zumindest in den letzten Jahren. #00:02:34-6#

I: Seit sechs Jahren sagen sie, unterrichten sie. #00:02:38-7#

B: Ja genau #00:02:39-2#

I: Sehr schön. Super! Hatten sie vor dieser Vorbildung zu PERMAteach ein Vorwissen zur positiven Psychologie oder zur positiven Bildung? Ist ihnen das schon mal untergekommen auf ihrem auch vielleicht beruflichen Weg? #00:02:52-6#

B: Nein, konkret mit diesen PERMA Begriffen nicht! #00:02:57-8#

I: Mhm, mhm, und was hat sie überzeugt, da mit zu machen? Also, wie war das für sie, dass sie da gesagt haben, da mache ich mit, da will ich mich weiterbilden. #00:03:08-9#

B: Ähm, der erste Anstoß ist von unserer Direktorin gekommen, die man auch gerne bereit ist, neue Sachen auszuprobieren. Ich glaube, das ist auch immer ganz wichtig, dass man eine Leitung hat, die sagt, ja, da sind wir dabei, und speziell was in der Klasse, in unserer Klasse, die damals eine erste war, dass ich mir gedacht habe, und mein Kollege, das ist eine Klasse, die das sehr gut brauchen kann, weil sie ja verhaltensauffällig im positiven und im negativen Sinn immer immer waren und nicht sehr gut zur Ruhe gekommen sind. Sehr, sehr, ja eine sehr aufgeweckte Klasse, und wir haben gedacht, vielleicht ist das gerade für so eine Klasse ein guter Zugang. Man macht, was man tun kann, um Problemchen zu bezwingen. Und da haben wir uns gedacht, das könnte gut passen. #00:04:03-9#

I: Jetzt, die nächste Frage passt jetzt sehr gut darauf. Haben sie einen Unterschied zwischen Regelunterricht und dieser positiven Bildung jetzt schon mal erlebt? Können sie da ganz grob, wenn sie jemanden erklären würden, was ist denn da der Unterschied? #00:04:21-5#

B: Ja, absolut. Also, ich glaube, es ist für jede Klasse gut, aber für unsere speziell, weil sie sehr, sehr schnell springen können in dem, was sie machen oder auch springen müssen, in dem, was sie machen und sich nicht lange auf eine Sache fokussieren können, äh, da fand ich viele Methoden aus der positiven Psychologie immer sehr gut, um einfach sie kurz rauszuholen oder eine andere Aktivität reinzubringen. So einen Schnitt zu setzen, um ja, wieder vielleicht Bewegung reinzubringen oder neue Gedanken oder weg von von schwerer Materien im Sinne jetzt etwas, wo sie denken, oh das müssen sie lernen, weil Schule kommt, ein Test, kommt eine Schularbeit, sondern etwas, was unabhängig von "Ich muss jetzt performen" oder "ich muss mir das merken" oder "ich muss das lernen" machen. #00:05:17-5#

I: Das wäre für sie der unmittelbare Unterschied zwischen klassischem Regelunterricht und eben dieser positiven Bildung? #00:05:26-3#

B: Genau im Idealfall macht man das sowieso in seiner Stundenplanung, dass man vielleicht irgendwas im Petto hat, wo man ein bisschen reaktiviert. Aber bei der positiven Psychologie und zumindest auch diesem Toolkit oder die ganzen Bücher, die man hat, finde ich, ist einfach sehr viel, was man sich rausholen kann und immer mal reingeworfen werden kann. #00:05:48-5#

I: Genau das heißt, sie haben den Unterschied schon festmachen können. Jetzt nochmal zurück an den Anfang. Sie haben sich überlegt, damit zu machen. Bei dieser Fortbildung, das ist ja auch immer mit Zeit und Aufwand verbunden ist, hat sich die Erwartung, die sie am Anfang hatten, erfüllt? #00:06:09-1#

B: Ähm, teils teils, also es kommt drauf an, was es war. Ich habe von dem rein theoretischen PERMA, dann waren für mich viele Wiederholungen, weil ich das Jahr davor hatte ich einmal einen, da waren sie bei uns an der Schule, und dann hatten wir über diese PERMA Fortbildung - wir wurden als Pilotklasse, als Lehrerteam dann ja intensiver begeleitet, hatten intensivere Fortbildungen, und mir ist zumindest der theoretische Teil immer sehr ähnlich vorgekommen. Wo ich mir gedacht habe, das habe ich schon gehört, ah jetzt wieder die fünf Finger. Ich verstehe, warum sie es so machen, weil jedes mal war wieder wer neuer dabei, für die das sinnvoll ist, das zu hören. Aber wenn man das fünfmal hört, dann dann denkt man sich ja, okay, dann auch mit dem Online macht es noch schwieriger, man schweift einfach ab. Mir würde das wahrscheinlich weniger passieren, wenn ich persönlich da sitzen würde und mir denken würde, ah, ja, das P steht für das, sodass eher ist für mich eine, auch eine Auffrischung. Aber gerade online habe ich mir das sehr schwer getan, das dann als zeiteffizient oder als Mehrwert zu erkennen. Ähm, Aber das ist nur zum theoretischen Teil. Profitiert habe ich immer von den Praxisteilen. Das heißt, wenn andere Schulen ist, wenn immer andere Schulen oder Vertreter da, die dann halt darüber reden, was, was funktioniert bei ihnen gut, oder wie arbeiten sie mit PERMA anders als wir mit PERMA arbeiten und ja auch Ergebnisse zeigen, von Videos bis Bildern, bis was auch immer gemacht wird. Also praktischen Input. #00:07:44-9#

I: Praktischen Input oder ? Im Prinzip auch auf Augenhöhe lernen von irgendwie "wie haben dies eingesetzt!" "Ach so, da müsste man aufpassen", "das wäre eine Intervention, die hat dort gut funktioniert" und so, also so der Austausch zwischen den Lehrern: Ok? #00:08:01-8#

B: Ja, genau. #00:08:02-7#

I: Gab es etwas, wo sie von vornherein gesagt haben, da bin ich skeptisch? Schauen wir mal, ob das überhaupt so ... würde ich sagen, sie haben, wie sie sagen alles sehr oft gehört. Ahm, waren sie immer davon überzeugt, dass das auch eine gute Idee ist? Oder gab es auch Skepsis, wo sie gesagt haben, da schauen wir mal? #00:08:19-9#

B: Ähm, Skepsis? Nein, zu Beginn nicht. Keine Skepsis. Eher dann eben schon ein bisschen hatte ich eine Erwartungshaltung, immer schon, jetzt haben wir dann wieder ein Treffen, dann dann kommen wieder ein paar Dinge, die wir schon gehört haben. Ähm, und ich muss sagen, gefühlt habe ich das ein bisschen meinem Team in der Schule aufs, also was heißt aufs Auge gedrückt? Ja, aber wir waren ein Schulteam, wir waren eine Pilotklasse, und natürlich will man alle im Boot haben. Aber ich habe mich dann immer schlecht gefühlt, alle einzuladen zu diesen Meetings, und dann müssen sie sich wieder dasselbe anhören. Das ist nicht unbedingt immer einfach zu verkaufen, dann. So, dass ich auch am Ende dann manchmal gesagt habe "ich bin auf jeden Fall dabei. Wer Zeit hat, kommt noch dazu. #00:09:06-0#

I: Mhm, mhm. Aber wir reden jetzt ganz konkret, weil sie jetzt sagen, sie haben einfach, weil ich ja nicht weiß, dass sie jetzt im Vorfeld damit meinen, sie sind schon als, also früher eingestiegen in das PERMAteach. Sie haben sozusagen immer wieder die selben Module gehört? #00:09:25-0#

B: Ja, ja, eben diesen Theorieanteil. Worum es geht bei PERMA teach, und das hat dann schon immer bei diesen, also ich rede jetzt diese von konkreten online Terminen, die Teil dieser Ausbildung sind, so wie ich das verstehe. Die habe ich nicht so sinnvoll bekommen, weil, wie gesagt, das war eine Wiederholung, und es war dann auch für das gesamte Team eine Wiederholung, und ich glaube, dass, wenn man es einmal hört oder zweimal und dann das ganze Material hat, dann ist man schon sehr, sehr gut dabei. Da braucht man nicht sehr viel Anleitung, weil es ist alles sehr gut, es ist alles erklärt. Ich könnte auch mit dem Material arbeiten, ohne jemals eine PERMA Fortbildung besucht zu haben, muss ich sagen, Deswegen meine ich. Was, was aber positiv trotzdem an den Meetings immer war natürlich eben dieser Austausch mit anderen Schulen, der Praxis-Input und für uns als Team, das würde ich nochmal unterstreichen, ganz, ganz, ganz wertvoll, dass wir uns ein, also wir gezwungen waren, uns so zusammenzusetzen. Etwas, was im Lehreralltag ganz viel fehlt, wovon wir viel mehr bräuchten, ist in Teams, wenn man schon das Privileg hat, Klassenteams zu haben, regelmäßig sich hinzusetzen und zu besprechen, "wie geht es dir in der Klasse?" "was machst du?" "was funktioniert?", "wo hast du Probleme? "mit welchen einzelnen Schülern geht es gut oder geht es nicht so gut?" Und diese Treffen waren immer wieder auch ein Ankerpunkt, wo wir das gemacht haben, wo wir uns da tatsächlich ... #00:10:56-8#

I: Ja, Der Mehrwert eigentlich aufgrund der PERMA Schulung? Eigentlich dann dieses Netzwerken, auch im Team untereinander, oder? #00:11:04-2#

B: Genau, weil sonst im Schulalltag kommt man gar nicht so, wie gesagt. Das sollte verankert sein. Es sollte in unserem Wochenplan verankert sind, dass wir eine Stunde uns zusammensetzen, alle sechs oder sieben, wie groß das Team halt ist, und am besten jedes Kind durchbesprechen. Ich meine, das ist eh utopisch, aber die, die Highlights und besprechen der Woche zum Beispiel, und das fand ich durch die die PERMA Meetings schon sehr gut, also auch dann, wo die anderen Lehrer gesagt haben, "Ah, ach so, bei dir ist es auch so?" Und ja, es ist auch ... #00:11:39-2#

I: Auch schön, wenn man, wenn man spürt, man ist nicht alleine, oder ? Es gibt also sozusagen, man kann sich austauschen. Da sind noch mehr vom selben Muster, die wollen und nicht immer können und manchmal scheitern. Das ist wahrscheinlich auch insofern ein Mehrwert, dass man eben nicht alleine kämpft. #00:11:57-5#

B: Genau. #00:11:59-2#

I: Mhm, mhm! Jetzt, wenn sie gerade vom Team sprechen, hupf ich jetzt gleich auf die Teamfragen: Wie ist es ihnen denn gelungen, diesen Spirit im Team dennoch - in diesem Falle - aufrechtzuerhalten? Sind die noch dabei? Sind sie ähm. wurden sie zur Einzelkämpferin, oder konnten sie eine breite Begeisterung auslösen? #00:12:20-8#

B: Ähm, ja, sicher eher eher breite Begeisterung. Also wir sind, sind wir sechs im Team, weil eh alle von, würde ich sagen, jetzt von ihrer Grundeinstellung, offen für neues sind und gerne Dinge ausprobieren und alles im Team, recht junge Lehrer, also neue Lehrer sind, die jetzt noch nicht mehr als drei Jahre Berufserfahrung haben und die in meinem Empfinden so was auch dankbar dann annehmen, wenn man ihnen dann noch, weiß ich nicht, ein Heft in die Hand gibt oder sagt: Ah, probier mal das, oder machen wir das! Und Ähm ja, das war auch der Struktur unseres Teams geschuldet, dass das positiv Anklang gefunden hat, und es gibt ein paar Dinge, die wir konsequent durchgezogen haben, also wo ich weiß, dass das jeder Lehrer in unserem Team gemacht hat. #00:13:20-0#

I: Das wäre jetzt auch eine Frage. Was sind denn die Dinge, die sie, also wie ein Ritual oder Dinge, die sie jetzt für sich als Lehrerteam beibehalten haben oder für sich als Lehrkraft in die Schulen, in die Klassen bringen? #00:13:34-7#

B: Ähm, also, was wir sehr viel gemacht haben, ist dieses, weil es einfach so kurz und simple ist, das ALI Prinzip. Ja, das Lächeln und Innehalten, weil, weil oft sind sie so gepuls getrieben, und wir haben einen Ali in der Klasse. Das ist irgendwie so gut angekommen in der Klasse, das er immer der Aufhänger war für für das ALI Prinzip. Also, das weiß ich, das war immer so ein. Das wurde eigentlich quer durch die Bank gezogen, dass man sich da immer wieder mal erinnert, dass man innehält und die Emotion vielleicht die überschäumend aufhält und in andere Richtung lenkt. Das war sehr konsequent, aber mit das Größte war wahrscheinlich, wir hatten so ein Spiel zur Disziplinierung. Hört sich vielleicht nicht so positiv an, aber weil so eine ähm, verhaltensauffällige Klasse ist, haben wir ein Spiel gehabt, wo sie zusammen gruppiert wurden, und als, bevor wir PERMA gemacht haben, war das eigentlich sehr negativ behaftet. Das Spiel in dem Sinne, dass man für Fehlverhalten, wir haben uns also ein Fowl-Spiel haben wir es genannt. Wir haben uns mit der Klasse drei Fowls ausgemacht, sowas wie, ich rufe raus, ich laufe durch die Klasse, ungefragt, sowas, das sie selber als störend empfunden haben, und für jedes Fowl, dass jemand begangen hat, hat die Gruppe einen Strich gesammelt. #00:15:00-3#

I: Mhm. #00:15:01-2#

B: Da gab es eine Verlierer und eine Gewinner Gruppe. Die Gewinner Gruppe haben dann natürlich Preise bekommen, wie mit auf einen Ausflug gehen oder Hausübungs-Gutschein, sowas. Und mit PERMA haben wir das ein bisschen geändert, weil da haben wir den Fokus nicht auf das negative Verhalten gelenkt, sondern haben begonnen, das positive Verhalten zu visualisieren auf der Tafel, und das haben wir in, das haben alle Lehrer in allen Stunden gemacht. Das war ja ein Spiel, das ist die ganze Zeit im Hintergrund gelaufen mit Namen an der Tafel, die wir herum geschoben haben. Ja. #00:15:33-7#

I: Also, gerade eine neue Perspektive auf ein bestehendes Ritual eigentlich. Und sie sagen, daß haben alle Lehrer in ihren Teams mitgenommen? #00:15:44-8#

B: Ja, in dem Großteil der Stunden, vielleicht nicht in allen Nebenfächern, dann. Es ist oft leichter, das zu spielen, wenn man zwei Lehrer hat, weil einer kann richtig beobachten und der andere visualisiert. Aber ja, es hat uns eigentlich immer sehr begleitet, und den Schülern hat es sehr viel Spaß gemacht, weil die auch sehr wettbewerbsgetrieben sind. Und ähm, ja, das hatte einen positiven Effekt. Wir haben da immer zwischendurch Pausen gemacht, haben gesagt, jetzt spielen wir nicht mehr, weil wir haben realisiert, wenn, wenn sie sich immer, wenn wir es jeden Tag das ganze Schuljahr machen würden, dann wird es seine Schubkraft verlieren. #00:16:22-2#

I: Zur Gewohnheit, genau! #00:16:25-9#

B: Und deswegen haben wir dann immer Gewinner gekürt, dann gab es ein paar Wochen Pause, und dann haben sie eh schon gefragt, "Wann spielen wir das Spiel?" "Wieso spielen wir nicht wieder das Spiel?" Auch wenn sie selber realisieren, dass sie nicht mehr funktionieren, muss man wirklich sagen, das realisieren sie dann auch. Es wird zu laut. Die Klasse wird zu schlimm #00:16:45-0#

I: Also diese, also auch den Fokus auf, wie soll ich sagen, die Selbstwirksamkeit, wird eigentlich auch aktiviert damit. Also um besser auf sich aufzupassen, zu wissen, jetzt ist nicht mehr gut, jetzt müssen wir mal wieder spielen, damit wir wieder in Ruhe haben oder in die Ruhe kommen. Spannend! Das heißt also, ALI als fixer Bestandteil in der Klasse mit Tier, also mit mit einem, einem personifizierten Geschichte. Gibt es da auch im Lehrerteam etwas, was sie sagen das hat sich verändert, seit sie da bei PERMAteach dabei sind oder dabei waren. #00:17:17-6#

I: Ähm, ja, eben sicher, dass man näher zusammen kommt und persönlicher zu reden beginnt. Gerade für junge Lehrer, glaube ich, ist es gar nicht so einfach, dann zu sagen, wenn ihnen was, wenn es nicht gut läuft. Man kommt irgendwie in diesem Beruf, und man ist vielleicht idealisiert, und man hat einen hohen Anspruch an sich selber, und ich glaube, es ist dann schwierig zu sagen, ich bin überfordert oder ich komme nicht zurecht mit der Klasse, ich habe sie nicht im Griff. Das ist, ähm, das ist total schwierig, glaube ich, sich das einzugestehen, gerade am Anfang, und das wird, umso länger man Lehrer ist und so, also sollte normaler werden, oder dass man sieht, man hat die, die, die Peer Group, man hat seine Kohorte, die, die dieselben Probleme hat oder die dir ja, auch wenn ich sechs Jahre Lehrerin bin, kann ich straucheln in einer Klasse oder 20 Jahre, kann ich auch mich plagen in einer Klasse, ähm! #00:18:18-9#

I: Und diesen Austausch auf einer ehrlichen Ebene hat es gefördert, sagen sie? #00:18:24-1#

B: Ja, genau! #00:18:25-2#

I: Und auch der, vielleicht der Austausch in den positiven Momenten? Weil ich glaube, dass ihr Lehrer ganz oft in der Situation seid, dass man natürlich die 17 guten Dinge, die am Tag passieren, ahm, aber der eine, der ....und dann redet man über das. Gibt es da einen Wechsel oder einen....? #00:18:43-3#

B: Ja, ja, eh durch das, durch diese Meetings mit PERMA total angeleitet, einfach auch, dass man sagt, was war, was ist heute gut gelungen. Dass man immer mit dem einsteigt, und ja, das hat da sicher geholfen, hätten wir aber wahrscheinlich in unserem Alltag noch mehr machen können, dass man, wenn man im Lehrerzimmer zusammensteht, dass man nicht jetzt den Kopf wieder über den Händen zusammenschlägt, sondern sich überlegt. "Ah, aber heute, ich habe zehn Minuten unterrichtet, das waren zehn Minuten", ich habe die haben, "sie haben eine neue Sache gelernt" oder so. Ja, also, das könnte sicher intensiver sein, aber das ist, glaube ich, das ist schwer. Bei aller, bei aller Fortbildung und so. Ich glaube, das muss man wirklich, muss man .... konzentriert angehen! #00:19:33-4#

I: Ja, also, dass wäre ja das schöne, dass dieses Psychosoziale, ja nicht sozusagen als Instrument, also PERMA, als Instrument für die Schüler, und dieser psychosoziale Aspekt, das auch für die Lehrer Gesundheit rückwirkend, also rückwirkt. PERMA, diese Hand, die man nach vorne versucht zu leben, auch für sich selber natürlich lebt. #00:19:57-9#

B: Ja. #00:19:59-4#

I: Aber die Zeit ist immer ein Faktor, wahrscheinlich? #00:20:02-3#

B: Ja, und wahrscheinlich braucht man da auch immer schon ein bisschen einen Anstoß. Also natürlich jetzt, wenn sie das so sagen. So retroperspektivisch könnte ich mir denken, gerade wenn ich das initiiert habe und ein bisschen die Leitung von dem Projekt in unserem Team übernommen habe, dass ich jedes mal, wenn ich mit wem aus unserem Team rede, die als erste Frage stelle. "Und was ist da heute gut gelungen in der Klasse oder so?" Also retroperspektivisch wäre das sicher gut, wenn ich so ein Ankerpunkt bin, für für so was im Team #00:20:32-1#

I: Oder eben auch inzwischen die Frage nach, wenn man, wenn man zumindest einmal nach der Stärke, also mit dieser positiven Brille, oder diesem Stärken- Spotting in die Klassen geht. Ich glaube, es tut dem Lehrer gut, weil es ein Haltungs Change ist, gerade in verhaltensauffälligen Klassen zu sagen: "Mensch, der hat hier eine Stärke, der ist ein Unterhalter," um jetzt ein Beispiel zu nennen. Dann hat man schon eine andere Perspektive, er muss trotzdem hinsitzen und das machen. Aber man hat, man bekommt so einen Wertschätzung-Blick für Verhaltensoriginelles. Das passt vielleicht nicht in die Situation passt, aber rein menschlich für das spätere Leben vielleicht. Ja. #00:21:13-5#

B: Ja, das haben wir sicher auch. Also in Tendenzen war das sicher immer vorhanden bei uns im Team, weil eben, wie gesagt, ein engagiertes, engagierte Lehrer, die da auch noch noch positiver an alles rangehen und das auch sehen können und ja optimistisch sind in ihrer Grundeinstellung. #00:21:35-9#

I: Toll! #00:21:36-3#

B: Aber ja, man könnte es definitiv noch mehr ritualisieren für sich selber. #00:21:41-2#

I: Eben. Also, das war jetzt die Frage nach nach innen und nach außen. PERMA wirkt ja, oder sollte ja, nicht nur für die anderen, sondern auch für einen selbst eine Möglichkeit sein, in diese gesunde, in dieses Aufblühen, in dieses Flourishing zu kommen. Aber es klingt so, als wenn ihr Team eh schon sehr engagiert ist und da dieser Optimismus, der ja auch eine große Stärke ist, leben kann. Jetzt ganz kurz noch mal zu diesen Bausteinen, die sie bekommen haben. Sie haben das Schulungs-Modul eins und Modul zwei, das ist glaube ich ein Buch gewesen oder ein Heft mit Seiten. Wie haben sie damit arbeiten können? War das brauchbar für sie? Jetzt einfach nochmal auf die Frage, theoretischer Input, praktischer Input und Material. Was war da etwas, das ihnen positiv aufgefallen ist? Was kann man da verändern? Oder was sollte man verändern? #00:22:40-5#

B: Ähm, ja, das Material ist sehr gut aufgearbeitet, würde ich sagen. Es ist eben nach Heftchen, die pro, eigentlich nicht nur, aber so verwendet werden können, pro Schulstufe also vier Bände, erste, zweite, dritte, vierte Klasse und dann auch thematische Schwerpunkte, was mir positiv aufgefallen ist für neue Fächer, die dann kommen, zum Beispiel in der dritten Klasse, das ist das orange Heft: ist dann ein großer Schwerpunkt auf Berufsorientierung und Dinge, die in dem Zusammenhang wichtig sind, und das ist in den Bänden davon nicht, weil es logischerweise auch noch keine Berufsorientierung gibt als Unterrichtsfach. Also von dem her ist es sehr sinnvoll aufgebaut. Aber ich könnte auch nur den ersten Band nehmen und ihnen vier Jahre lang verwenden. #00:23:31-9#

I: Mhm. #00:23:32-6#

B: Ja. #00:23:32-9#

I: Also viel Material? #00:23:35-1#

B: Es ist sehr viel Material, und das ist auch einbisschen ein, also Kritikpunkt ist falsch, weil Material ist immer super. Aber man tut sich ein bisschen schwer, das richtige zu finden, was man gerade brauchen könnte oder was in diesem Moment passt. Ich müsste eigentlich jedes Heft, so habe ich das Gefühl, stundenlang schon mal mich damit beschäftigt habe und durchgelesen haben und mir viele Pickerl gemacht haben mit daneben vielleicht Stichpunkten, dass ich weiß, das passt jetzt perfekt für diesen Moment, weil dann, wenn ich, wenn ich vielleicht irgendwas brauche, was in PERMA drin ist, dann finde ich es in dem Moment nicht, und dann ist der Moment schon verstrichen, wieder und dann finde ich es Wochen später und denke mir, "Ah, na, das hätt so gut dazu gepasst", und da habe ich mich immer ein bisschen geplagt, und das war auch eine Rückmeldung von Kollegen, Kolleginnen, das viel, sehr, sehr, sehr viel Material ist. Ich meine, sowas ist ja nicht negativ, aber dass man - ich weiß, nicht, man bräuchte eine Search Engine, Online, dass das alles eingefüttert ist, und ich gebe ein Stichwort ein, sag Impulskontrolle oder irgendwas, und es schlägt mir Übungen vor, die zum Thema Impulskontrolle passen. #00:24:49-3#

I: Das ist jetzt ein wunderbarer, kreativer Beitrag, wie wir, den ich gerne weiterleite, wie man diese Flut oder diese Mengen an wirklich tollen Inputs userfriendly gestalten kann. Und da haben sie schon recht. Viel Material ist natürlich da. Das die ist die Idee, möglichst vielen Menschen unterschiedliche Sachen anzubieten, aber es kann natürlich dann auch ja schwierig werden, das richtige zu finden. Das wäre jetzt das Stichwort für die Newsletter. Sie bekommen ja wöchentlich oder haben wöchentliche PERMA Newsletter bekommen. War das hilfreich? Weil das waren dann ja kleinere Portionen. Als reminder, unter Umständen. Hat man das praktisch gleich umsetzen können? War das hilfreich? #00:25:37-9#

B: Ähm, ich, ich glaube, es kann hilfreich sein. Ich habe es persönlich wenig genutzt, weil ich dann, ich komme nicht in die Schule und setze mich gleich zu meinen E-Mails, sondern ich habe einfach einen anderen Arbeitsflow und Ähm, habe dann öfter das gelesen, aber vielleicht hat es halt gerade.... Ich finde die Idee gut. Es war, es war auch immer so, uns kommuniziert, das sind so ein Häppchen. Dann lass heute, mach einfach diese Übung, die dir da gerade vorgeschlagen wird. Ich hab das selten gemacht. Manchmal war es gerade passend, und ich habe es im richtigen Zeitpunkt gesehen und habe dann im richtigen Zeitpunkt dann gedacht, ach, das war jetzt ein guter Lückenfüller, das würde passen. Aber in meinem so Arbeitsalltag ist es ein bisschen untergegangen. #00:26:30-0#

I: Also andere, also andere, auch Herangehensweise an e-Mail und Newsletter. Aber störend war es nicht? War es ein overkill oder unangenehm, weil lästig? #00:26:39-7#

B: Ähm, nein, habe ich nicht so empfunden, also aber das ist, ich empfinde generell Emails nicht selbst lästig, und äh! #00:26:48-5#

I: Man kann es ja weg, man kann´s ja wieder löschen, wenn man es nicht braucht. #00:26:50-5#

B: Ja, ich überlege nur. Generell habe ich auch mit anderen Lehrern die Erfahrung gemacht, das sind so oft nicht so e-Mail.Menschen. Also, mir ist es dann selber aufgefallen, dass ich, wenn ich jetzt Meetings kommuniziert habe in meinem Team, oder wir haben da wieder was von PERMA, wenn ich das nur als e-Mail schreibe, das geht unter. Also das ist, dann vergisst man einen Termin oder hat noch nicht mal die e-Mail gelesen. Also, in meiner Erfahrung sind Lehrer nicht so die e-Mail-Menschen. #00:27:21-5#

I: Jetzt frag ich gleich weiter. Das gehört jetzt nicht zum Interview, aber sie interessiert mich, weil man daraus was lernen kann. Wie erreicht man denn Lehrer am besten? Mit Plakaten? Postkarten? #00:27:35-7#

B: Ja, Anrufe im Lehrerzimmer ist auch schwierig, je nachdem, wozu man sie erreichen will. Ja: Ähm! #00:27:42-0#

I: Mit diesem Newsletter, mit dieser Inspiration ALI - um die nicht zu vergessen zum Beispiel, einfach so ganz leicht Inputs. Sind es Karten? Sind das Karten, die man vielleicht auflegt? Und. #00:27:56-7#

B: Ja, es muss gut, sichtlich sein, irgendwo, ja, vielleicht haptisch, visuell, etwas, was ich gleich, wenn also das mit dem Ali oder sonst irgendeine Methode ist, sei es jetzt den den Puls zählen oder irgendwas, wenn ein Kollege im Lehrerzimmer neben mir steht und irgendwas in der Hand hat und sagt, ich habe das gerade in der Klasse gemacht, hat nur fünf Minuten gedauert, aber das hat Wunder gewirkt, ich sag dir, ja, - hier, dann nehme ich das, ich gehe eine Klasse, und ich mache es. #00:28:28-0#

I: Mhm, Okay. Also visuell, haptisch und und Häppchen. #00:28:34-1#

B: Und jemand, dem man gerade sagt, hey, ich habe das gemacht. Brauchst du noch was? Auch wenn du einen anderen Stundenplan hast? Egal, dauert nur fünf Minuten, machs irgendwann, weil irgendwann sind sie eh gelangweilt, oder ja? #00:28:47-2#

I: Ja, okay, Ich nehme das gerne mit als Anregung, weil diese Gestaltung, das ist ja genauso. Man kann ja einiges auf Karten machen, und wenn man dann sagen würde, ihr habt alle gute Erfahrungen damit und damit gemacht, sucht euch was aus und teilt das so unter den Lehrern. #00:29:02-6#

I: Ja, #00:29:03-5#

I: Ich bin in meinem Original-Leben Kommunikations-Expertin für Werbung und Marketing -darum höre ich natürlich gleich die Möglichkeiten, wie hier Kommunikation noch ein bisschen besser funktionieren kann. #00:29:17-2#

B: Ja, also, wie gesagt, e-Mail ist, glaube ich, nicht the way to go mit Lehrern, so wie ich sie kennengelernt habe, auch im Vergleich, weil ich auch in der Privatwirtschaft gearbeitet habe oder halt in Firmen oder Institutionen. Da liest jeder seine e Mail, und es gibt es nicht, dass man sich nicht einlockt und eine e-Mail verpasst. Aber Lehrer verpassen E-Mails, wochenlang. #00:29:37-1#

I: Super interessanter Input, weil, wenn, ich muss mich auf die Bedürfnisse meiner Zielgruppe einlassen, und wenn die nicht e-Mail lesen, ist das e Mail der falsche Weg zum Kommunizieren. #00:29:48-1#

B: Ja, also, mir ist es ja mit ihnen auch passiert, obwohl ich normalerweise natürlich im Lehreralltag schon einmal zumindest am Tag in meinen e-Mail Account schaue. #00:29:59-4#

I: Naja, das ist in diesem Fall nicht die oberste Priorität. Man funktioniert vor Ort, man ist konzentriert, man geht da rein. Und jetzt wäre nur die Frage, die mich jetzt bewegt hat, wie komme ich denn mit einer Info an den Lehrer heran? Aber danke für die Info, das werde ich weiterleiten. Das finde ich super. Jetzt nochmal ganz kurz zu diesen Schulungsunterlagen. Also, sie haben gesagt, die Bücher sind super, die haben eine Fülle. Ähm. War ihnen Präsenz oder virtuelle lieber? Sie haben gesagt, sie waren, sie hatten ja beides. Die Vernetzung war ihnen da vordergründig das wichtigste, habe ich herausgehört, und die Ausbildung hat sich wiederholt. Virtuell genauso wie in den Präsenztagen? #00:30:43-8#

B: Ja, obwohl also ich profitiere, das ist meine persönliche Einstellung, ich profitiere von Präsenz, mehr, auch was theoretische Inputs angeht, weil dann bin ich da, und dann dann muss ich quasi da sein, dann bin ich körperlich, da, bin ich das gelingt bei Computer, andere Dinge zu beginnen, was ich dann in einem so Meeting schon mache. Wenn wieder derselbe Imput kommt, der ich schon gehört habe, dann beginne ich auf einmal abzuschweifen und verpass vielleicht doch die Dinge, die, die für mich doch neu wären, oder ein guter, eine gute Auffrischung. #00:31:20-3#

I: Den fachlichen Impuls haben sie schon, also beurteilt, im Sinne von, dass, dass sie das großartig fanden, was an Informationen da war, aufgearbeitet. Schriftliche Handout haben wir auch schon besprochen, und ah, die weiterführenden Inspirationsquellen. Da gab es ja immer wieder so Apps und Dinge, die man also, die den Alltag vereinfachen würden. Haben sie da was nutzen können? #00:31:46-6#

B: Also, wir haben probiert. Huh, Ich habe jetzt schon diesen den Namen vergessen. Wie das heißt, irgendwas mit Talent?. #00:31:53-9#

I: Mhm. #00:31:54-0#

B: Mhm, das auch über Perma. Durften wir das gerade verwenden? Haben wir gerade das Lizenzen bekommen? Ich habe jetzt schon den Namen vergessen, muss ich sagen, weil. #00:32:02-6#

I: Ja. #00:32:03-0#

B: Irgendwas mit talent? talent tool? loop? #00:32:09-6#

I: Ja,sie haben das raufgeladen, weil es gratis war? #00:32:15-0#

B: Genau, das wollten wir probieren, weil das war gratis, und normalerweise kostet das recht viele so Schullizenzen, und das hat in meiner Klasse gar nicht funktioniert, muss ich sagen. Wir haben das zwei, dreimal probiert. Das war nicht sehr erfolgsversprechend. Aber das ist, muss ich sagen, auch nicht nur diesem Tool geschuldet, sondern einfach der schulischen Infrastruktur. Wir haben immer ein Problem mit Internet und Dinge, wo es darum geht, sich wo einzuloggen und Online was zu arbeiten. Ähm, es ist vielleicht ein anderes Thema, aber wir sind auch eine iPad Schule und haben iPad Klassen, was toll ist. Aber wir können Apps und Sachen Online kaum verwenden, weil das das System einfach nicht mitspielt, weil das Internet nicht mitspielt, und dann kann man mit Dingen nicht mehr arbeiten, weil, wenn dann vier eingelockt sind, zehn warten, und die anderen fünf können sich nicht mal einloggen. #00:33:09-1#

I: Mhm, dann ja, das sind die Voraussetzungen nicht optimal. #00:33:13-0#

B: Ja. Und das ging uns mit diesem Tool so. #00:33:15-7#

I: Ja, ja, genau, und dann hört man auch damit weiter zu experimentieren. Okay. Schade! Noch ganz kurz zu diesen Rahmenbedingungen. Wie wurden sie eingeladen? Hat das geklappt mit Erinnerungen, mit Terminen? So dieses organisatorische, Orga? Gibt es da etwas, was, was gut gepasst hat? Oder etwas, wo sie, wo sie Verbesserungsvorschläge hätten, damit wir auch das noch gehört haben? #00:33:42-1#

B: Nein, das war gut gemacht. Man hat von Anfang an die Termine gekannt, es gab dann immer wieder eine Erinnerung, und ... das hat alles für mich geklappt. Ich war da dahinter. Ich habe dann auch immer meine Kollegen Ähm erinnert. #00:33:58-2#

I: Mhm. #00:33:59-1#

B: Ja. so hat das gut ... #00:34:02-3#

I: Weil ich das gerade noch sehe und ich vorher vergessen habe: das Padlet, das angeboten wird? Haben sie da mal reingeschaut? Oder war das für sie eine Inspirationsquelle, oder haben sie sich aus dem ...? #00:34:15-0#

B: Ja, ich habe mal reingeschaut, aber ich habe nicht wirklich damit gearbeitet. Meine Hauptquelle waren wirklich die Bücher. #00:34:22-5#

I: Bei ihren Kollegen? Wie ist es bei ihren Kollegen? Hat jeder von ihnen Bücher bekommen? Oder war das einfach für die Schule oder für das Team? #00:34:31-7#

B: Also bei uns in der Klasse hat jedes Kind ein Exemplar Buch gehabt, also wir haben in dem Buch direkt gearbeitet gehabt. #00:34:39-9#

I: Als Unterrichtsmaterial. #00:34:43-5#

B: Genau, und so haben auch die meisten Lehrer bei uns an der Schule, also ich habe das dann, wer sich dafür interessiert hat, habe Bücher in meinen Klassenstärke bestellt. Alle ersten Klassen haben bei uns mit diesem ersten Band gestartet, und, ähm ja, unsere Direktorin, die auch ein großer Fan von dem Material ist, die ich glaube, es war nicht wirklich verpflichtend, ob sie möchte, das kann eigentlich verpflichtet machen, dass man sagt, einmal die Woche arbeitet jeder mit diesem Buch, weil wenn man eh schon eins hat. #00:35:11-1#

I: Genau okay, also, das heißt, mit dem Buch, höre ich jetzt raus, hatten sie die größte Freude. Mit dem konnte man am meisten rausziehen, und der größte Nutzen eigentlich aus diesen Geschichten. Hätten sie sich noch was gewünscht an Material, an Input, dass sie nicht bekommen haben? Gab es etwas, wo sie ganz konkret sagen, das wäre noch toll gewesen? #00:35:35-4#

B: Nein, also, ich finde, es ist, ich kann mich nur wiederholen, es ist sehr viel eigentlich. Ich glaube, man kann sich vier Jahre Schule damit füllen, auch weil es neben diesen Jugend stärken gibt es ja noch dann das mit der Aufmerksamkeit. Ein eigener Band, ein eigener Band, ganzheitlich lernen. Also, mir würde nichts einfallen, es ist sehr, sehr, sehr fächendeckend! #00:36:01-2#

I: Das ist schon genug da, höre ich raus. Man muss es jetzt einfach nur tun, man braucht auch Zeit, das umzusetzen. Was mich zu der Frage bringt: wann genau haben sie denn dieses Buch eingesetzt? Ist es sozusagen dann wirklich, wie die Direktorin sich wünscht, einmal in der Woche war das in allen Unterrichtsfächern? Gab's ein eigenes Fach oder gab es eigene zehn Minuten dafür? Wie haben sie das integriert? #00:36:27-7#

B: Ähm, also, die Bucharbeit an sich, haben wir integriert in einer eigenen Stunde, die bei uns LIM heißt, Lernen und Informationsmanagement. Da geht es eher um Lernen lernen oder soziales Lernen. In jeder Schule heißt dieses Fach anders. Da haben wir es genutzt, und da haben wir auch die meisten Dinge zu PERMA gemacht, weil wir da immer als Doppelgespann in der Klasse sind, als KV und Co KV. Was dann auch die die größte Wirksamkeit hat, weil es, weil es mit uns verankert ist, irgendwie und was mit uns verankert ist, ist auch immer dann. #00:37:08-3#

I: Genau, authentisch! #00:37:10-0#

B: Ein bisschen ja und ein bisschen tonangebender, würde ich mal sagen, wo man sagt, das sind Sachen, die wir machen in der Klasse, das ist unser, unser Spirit. #00:37:18-0#

I: Mhm, also, auch die Haltung hat's hat's eigentlich demonstriert. #00:37:22-0#

B: Ja. #00:37:23-6#

I: Mhm also, das heißt, da haben sie auch in eine andere Energie im Raum. #00:37:30-1#

B: Definitiv, ja, definitiv! Also, ich kenne es nur aus so einer Rückmeldung auch von Lehrern. Also wir haben, muss ich sagen, sowieso zu zweit ist das anders in der Klasse. Aber wenn man dann alles auch noch KV-Team in der Klasse ist, da hat man natürlich ein ganz anderes Arbeiten mit den Kindern. Da haben wir auch, muss man sagen, keine disziplinären Probleme, oder da kann man anders damit umgehen, auch weil wir schon unsere Methoden haben und weil wir die Kinder halt dann noch mal viel besser kennen, als vielleicht Lehrer, die dann nur ein oder zwei Stunden in der Klasse stehen. Das ist halt dann Unterschied. Ich bin elf Stunden in der Klasse gestanden und unser KV, mein Kollege 13 Stunden, also das sind so so viele Stunden, die man verbringt. #00:38:14-9#

I: Da lernt man sich wirklich gut kennen, und dann kann ich auch diese Frage stellen, nämlich diese Veränderungen, die sie festgestellt haben. Können sie das an diesen fünf Fingern, also festmachen, im Sinne von positiven Emotionen: wird mehr gelacht? Sind die Menschen freundlicher zueinander oder kann man das gar nicht so zuordnen, sondern einfach nur fühlen? Wissen sie mehr über ihre Stärken? Die Menschen, die jetzt mit diesem, mit euch und dem PERMAteach gearbeitet haben, bessere Beziehungen? #00:38:57-3#

B: Beziehungen auf jeden Fall, allein durch, weil so viele auch persönliche Fragen gestellt werden, weil auch PERMA darauf aufbaut. Natürlich, dass man sich kennenlernen, dass man es wird, ein Glückstagebuch geschrieben oder so Sachen, die halt nicht im Schulalltag passieren, die auch unter anderem sehr persönlich sind, was, was aber auch nicht immer einfach zu teilen ist und natürlich auch immer auf einer Freiwilligkeit passiert, aber die Kinder das eh meistens gerne teilen wollen. #00:39:30-9#

I: Mhm. #00:39:31-4#

B: Vielleicht nicht am Anfang mit jedem, aber irgendwann dann auch vor der Klasse, also bessere Beziehungen, auf jeden Fall. Auch zu, also vielleicht noch mehr zu den Lehrern als also unter nach und aber auch, aber sicher eine bessere Schüler-Lehrer Beziehung, weil es mit sehr viel Interesse eingeht, etwas, was die Schüler viel, viel mehr wollen, eigentlich, glaube ich auch, als sie, als ihnen bewusst ist, oder viel wichtiger ist, für Lernen und für den Lernprozess, eine positive Beziehung zu haben zu dem Lehrkörper, der sich ernsthaft und authentisch für sie interessiert, abseits davon, was sie für eine Note auf die Schularbeit schreiben. #00:40:14-9#

I: Mhm, Mhm, Okay, also in diese Richtung hat, würden sie jetzt den Unterschied fest machen können? Ich frage jetzt bewusst in die andere Richtung. Hat sich ihre Beziehung zu einzelnen Schülern verändert, weil man mehr über jemanden weiß? #00:40:32-0#

B: Ja. So ist es sicher so immer im Moment, wo man sich denkt, das hätte ich jetzt gar nicht gedacht, oder wenn man sieht, was sie, was sie auch für eine Reflexionsfähigkeit haben, manche Kinder, denen man das so vielleicht gar nicht zutrauen würde in in ihrem Verhalten, dass sie sonst zeigen, und die dann halt mit anderen Methoden, vielleicht auf einmal. #00:40:57-7#

I: Mhm also, man lernt sich besser kennen, also das lustige an PERMA ist ja, dass sich alles, das ist ja nicht wirklich trennbar, sondern es ist ja wirklich das eine bedingt dann das andere, egal wie man es jetzt nennen. Aber sie merken einen Unterschied. Ich wollte jetzt nur noch wissen, ob das irgendwie zuordenbar wäre? #00:41:15-0#

B: Ja, für die Beziehung auf jeden Fall, aber also jetzt, unabhängig vom PERMA, ist es immer wichtig und, glaube ich, wenn man sowieso eine Person ist, eine Lehrkraft, die sich für seine Schüler interessiert, dann wird sich die Beziehung immer verbessern. Und Perma gibt halt nochmal so Tools, die das wirklich auch in eine Richtung lenken. Wo du sagst, auch, du gibst mit Übungen in den Kindern auch tatsächlich das Gefühl, dass dass dich das interessiert. #00:41:44-3#

I: Wahres, echtes Interesse an ihnen, an ihren Themen außerhalb der Noten. Gibt es eine Veränderung, wo sie sagen würden, da ist mein Herz aufgegangen? Das war das schönste, ein richtig schöner Moment, ein bisschen ein "Wow-Effekt" ? Bei Lehrern, bei Kollegen oder bei Schülern oder bei sich selber? #00:42:09-4#

B: Ähm, ja, sicher, viele. Mir würde jetzt gar nichts konkret einfallen, also viele Momente, wo man einfach sehr stolz auf sie sein kann als Klasse. Ähm ja, zum Beispiel eben, wenn ich an dieses Spiel denke, dass es sich dann eingebürgert hat, dass sie das selber einfordern, weil sie, weil sie merken, dass es ihnen gut tut oder dass es gut tut, zu sehen, wenn, wenn es richtig funktioniert, dass sie den Ansporn haben, dass sie dann eine Belohnung oder was dafür bekommen. Also diese, die die persönliche Weiterentwicklung, auch was was Selbstreflexion angeht, hat mich oft begeistert. Auch Kinder, die noch in der ersten Klasse, das weiß ich nicht, ob das mit PERMA zu tun hat oder ob es dann einfach eine, auch eine Altersfrage ist, aber die unsere ersten Klasse noch vor lauter Wutausbrüchen Tische umgehaut haben oder in Tränen ausgebrochen sind, die so sehr viel mehr schon ein Jahr später selbstregulierend waren. Ähm, also, das habe ich bei einigen unserer Schüler, die sehr, sehr unter Impulskontrolle, also nicht nicht viel Impulskontrolle hatten, dass sie in der zweiten Klasse schon ganz andere Menschen waren. Aber das kann ich nicht unbedingt PERMA zuschreiben, das weiß ich nicht. #00:43:44-0#

I: Nein, das muss ja nicht. Ein Zusammenhang möge bestehen, egal in welcher Zahl, aber man hat Freude. Das sind die schönen Momente des Lehrersein, möge PERMA was beigetragen haben oder die Haltung oder die Entwicklung. Was sagen denn andere, die jetzt ihre Klasse kannten und kennen, bemerken andere, Außenstehende auch eine Veränderung? Die Eltern oder andere Lehrerkollegen? #00:44:15-6#

B: Ähm, andere Lehrerkollegen, die nicht. Das ist eher schwierig, weil wir immer dieselben Leute eigentlich im Team haben, das kann ich schwierig sagen, weil sonst sind nur Leute, die nicht in der Klasse stehen, die supplieren halt mal, und das ist nicht, finde ich, kein besonders aussagekräftiges, weil die Klasse funktioniert über Beziehung, wie die meisten Klassen, und wenn dann ein Fremder drin steht, dann sind sie einfach eine andere Klasse und eine andere Dynamik, und dann ist vielleicht alles, was wir uns mit ihnen arbeitet haben, als Team vergessen, und sie sind, sie sind eine andere Klasse. Also, das ist schon funktioniert, schon kausal irgendwie mit uns. #00:45:00-6#

I: Mhm, ja, natürlich ist das eine Beziehungsthematik. Also ein Außenstehender, würde jetzt nicht erkennen können ... Aber ein Außenstehender könnte vielleicht erkennen, dass ihre Klasse positive Bildung hat, oder ? #00:45:17-0#

B: Und von den Eltern tue ich mir auch schwer, ob sie das so sagen würden, weil die kennen ihre Kinder ja auch noch mal anders. Die Kinder sind ja zu Hause auch noch mal, oder die Eltern nehmen ihre Kinder ganz anders wahr, als wir sie wahrnehmen, und fallen dann auch manchmal aus den Wolken, wenn wir irgendwas erzählen, wie wir die Kinder wahrnehmen. Deswegen, glaube ich, war von unserer Seite auch mehr die Aufgabe, das zu kommunizieren, was wir Positives an Veränderung sehen. Also ... #00:45:46-8#

I: Das haben sie. Hatten sie die Gelegenheit, dass den Eltern auch so zurückzumelden ? #00:45:52-4#

B: ja, ja. sicher. #00:45:54-9#

I: Also besteht ein Unterschied, vielleicht bei anderen Lehrpersonen also, dass die Eltern so diese positiven Rückmeldungen bekommen, was sich auch verändert hat, von der ersten Klasse, sage ich jetzt bis zur zweiten oder dritten. #00:46:07-7#

B: Ja, das ist ganz wichtig, finde ich, eine der wichtigsten Faktoren, dass man dann auch, dass man den Vergleich zieht und ihnen sagt, "ach, letztes Jahr, da hat er noch", ich weiß nicht, "mit dem Federal vor Wut durch die Klasse geschmissen", wenn was weiß ich, was war, und jetzt, das kann ihnen auch sagen, es ist so viel ruhiger geworden, und er kann anders damit umgehen, wenn er gestresst ist, oder also das, das finde ich ganz wichtig, dass die Eltern das zu sagen, das haben wir auch immer gemacht. #00:46:39-2#

I: Was ja eh schon positive Bildungs-Haltung ist, also da kommt dann als als neue vielleicht Methode oder als neues Buch dazu. Aber die Haltung ist diese positive Bildung, die ich da raus höre, und die bemerkt man offensichtlich auch in diesen Methoden, die sie da anwenden, in dem man mit den Eltern positive Gespräche führt. #00:46:58-8#

B: Ja, ja, absolut ganz wichtig für die Eltern, weil meistens werden sie eh nur angerufen, wenn irgendwas passiert ist, und auch in so ein Gespräch zumindest ist das unser unser Usus von jetzt als KV werden auch immer positive Entwicklungen verpackt. #00:47:16-4#

I: Super, super! Ja, ja, also die Fragen, die ich jetzt danach habe, sind die: Wie erleben sie die Haltung der Schulleitung? Das haben sie mir schon erzählt, dass die Direktorin - Gott sei Dank - in ihrem Fall sehr unterstützend ist und sie sogar auch mehr unterstützend ist, das ja wirklich auch noch proaktiv gerne hätte. Gibt es Lehrer, die sie noch gerne mitnehmen würden, also die jetzt nicht in ihrem ersten roll out dabei waren? Gibt es dann noch welche, wo sie sagen, die würde ich noch gerne anstecken, oder sind die ...? #00:47:49-1#

B: Ja, also, ich glaube, es muss, ich fand es, wie wir es gemacht haben, ist ja sinnvoll, so als Team. Ich glaube, es ist am wirkvollsten, wenn man sagt, alle kennen das, die in einem Team arbeiten, und alle machen das. Ich glaube, es bringt weniger, wenn ich jetzt Lehrerkollegen, die eigentlich einen ganz anderen Klassen sind, und ich kann denen immer mal zu Schmankerl in die Hand drücken, wie ich gesagt habe. Aber ich glaube, der Große, die große Wirksamkeit kommt eher durch ein flächendeckendes Team praktizieren von von Sachen, die immer wieder durchgezogen werden, ob es ein Zeichnen oder in Mathe oder in Turnen ist, dass die Kinder auch den Wiedererkennungswert haben von Dingen, die man macht, oder die Methoden, die man einsetzt, oder ob es ALI ist oder eine kurze, eine kurze Atem-Meditation oder irgendwas, was sie kennen, weil dann können sie sich schneller drauf einlassen. Also ist uns beim Meditieren, vor allem das haben wir auch öfter gemacht, aufgefallen, dass das ganz ganz, wenn man das beginnt mit ihnen noch in der ersten Klasse ganz schwierig so liegen und einfach nur atmen und in den Körper spüren, das können, da können sie nichts damit anfangen, und dann, umso öfter man es macht, ist es, dass sie halt, dass man merkt, dass sie das können, bis vielleicht genießen, sie sehen, dass sie das jetzt brauchen, dass sie da jetzt liegen und einfach nur atmen. #00:49:15-4#

I: Aber auch das ist ein Lernen, auch eine Vertrauenssache natürlich, und und ähm ja, also ich habe da noch die Frage, welche Veränderungen können sie in der gesamten Schule feststellen? Gibt es Projekte, Rituale, neue Projekte, wo man sagt, die sind irgendwie initiert worden? Das kann man an der ganzen Schule schon merken oder spüren? #00:49:45-1#

B: Jetzt mit PERMA konkret könnte ich nicht sagen, weil wir waren eigentlich nur eine Pilotklasse. Ich weiß, in anderen Schulen, die sind ja mal Pilotschulen, haben die fogiert, und wir haben gesagt, wir sind ein Pilotklasse, weil wir das tatsächlich nur so, als auch die die Fortbildungen, nur das Team von von unserer Klasse besucht haben und aber auch andere Klassen das Material verwendet haben. Aber da habe ich halt nicht so den Austausch gehabt, oder ja. #00:50:13-8#

I: Das Team in ihrer Klasse das sind zwei Leute, also sie und ihr Kollege? #00:50:18-0#

B: Ja, wir sind sechs, oder sind wir sieben? Also Lehrer, die in der Klasse stehen und alles unterrichten, was es zu unterrichten gibt. #00:50:24-6#

I: Okay, aber nur in ihrer ersten oder dritten Klasse: Okay, jetzt habe ich es verstanden. #00:50:27-7#

B: Genau, wir haben das nur in der Klasse, wo ich Co-KV bin, und mein Kollege KV in der jetzigen, 2b haben wir das so. #00:50:34-9#

I: Okay, und das war so ihre Initiative für die anderen, die sie mitgenommen haben. #00:50:40-0#

B: Genau! #00:50:40-7#

I: Okay, aber gibt es also. Würden sie sagen, aufgrund ihrer Erfahrungen - Ja, es war so gut, oder es war auch die Ausbildung so interessant? Würden sie sie anderen empfehlen? #00:50:53-1#

B: Ähm, ja, eben, aber auch mit dem Hinweis, dass ich das theoretische, das theoretische ein bisschen abgespeckter wäre oder sich nicht so oft wiederholen würde, dann durchaus, aber alleine durch diesen Austausch mit anderen Schulen, mit anderen Lehrern oder mit den eigenen Lehrern. Und eben auch die Zeit haben, in so eine Selbstreflexion zu gehen oder vielleicht selber aus einer negativen Spirale zu kommen. Für sowas sehe ich, dass es immer sinnvoll, würde ich auf jeden Fall empfehlen. #00:51:34-0#

I: Das ist doch schön, das hören wir, das hören wir gerne. Wir nehmen die Kritikpunkte, auch gerne mit, weil man möchte ja so viele wie möglich von der guten Sache inspirieren, und manchmal scheitert man halt an der Form oder an diesem und das, und deswegen sind diese Interviews für uns jetzt so wirklich wertvoll. Da bleibt mir nur noch zu fragen, in alter PERMA Manier: wofür sind sie dankbar? Gibt es drei Gefühle, die sie mit dieser Ausbildung, mit diesem Jahr, das jetzt hinter sich haben, verbinden würden? Was war so richtig gut, und wofür sind sie dankbar in dieser, in diesem Zusammenhang? #00:52:13-8#

B: Ähm, ja, ich bin sicher dankbar dafür, dass ich in unserem, das wir als Team so näher gekommen sind, dass wir, wie ich schon gesagt habe, in einen sehr authentischen Austausch gekommen sind durch PERMA, mehr als wir es sonst gemacht hätten. Ähm, ich bin auch dankbar für die Fülle einfach an Methoden, die, die ich gar nicht, die ich gar nicht wüsste, wie ich mir zusammensuchen müsste, oder, ich weiß nicht, aber die sehr komprimiert da verpackt sind und mit dem ich immer, auch wenn ich wieder zurückkomme aus der Karenz, sich immer wieder arbeiten werde. Ja! #00:53:02-9#

I: Und die letzte, allerletzte Frage ist einfach noch: Gibt es noch etwas, was ich nicht gefragt habe? Was sie unbedingt noch ergänzen möchten? Etwas das noch gesagt werden muss? #00:53:13-1#

B: Zu PERMA und der Fortbildung? Das Team hinter PERMA ist natürlich großartig. Die, die Eva und die Ingrid, die Tragen, sehr viel, muss man wirklich sagen, die tragen dieses Programm schon sehr gut mit, auch in ihrer Persönlichkeit. Das sind, würde ich sagen, Frauen, die das ganze Programm ausstrahlen und denen man auch abnimmt, dass sie es leben, so wie man sich das wahrscheinlich wünschen würde, dass man selber so leben kann, jeden Tag in der Schule mit diesem positiven Fokus und diesen ja. Ingrid eine Meisterin der Stärken-Lupe und so. Ja, also, das Programm ist sicher auch durch die Personen gut, gut verkauft und gut getragen. Ja, das ist so! #00:54:07-0#

I: Ja, das wäre es schon gewesen. Ich danke ihnen wahnsinnig für ihre Zeit. Ich freue mich sehr für sie, dass sie ein neues Leben unter ihrem Herzen tragen und sozusagen dann in der Mutter-Zeit auch PERMAZeit, hoffentlich haben. Und das ist ja das zweite, denn das erste ist ja jetzt im Kindergarten? #00:54:34-1#

B: Ja, ich freue mich, ist schon sehr. Darf ich, darf ich ihnen auch noch eine Frage stellen? Für welchen Master ist das? #00:54:41-7#

I: Ich mache einen Master in positiver Psychologie in Ismaning. also das ist durch die Deutsche Gesellschaft für positive Psychologie organisiert, an der DHGS. Das ist die erste, also wir sind jetzt erst der dritte Jahrgang, aber das ist die erste akademische, sozusagen Ausbildung, und ich mache das drei Semester nebenberuflich und bin schon. Also, es ist, es braucht, es ist intensiver, als ich mir das gedacht hätte. Aber ich komme aus diesem PERMA lead Bereich, als ich nach Trainings und Workshops für Führungskräfte und kommen über diese, über diese dieselbe 5 Finger gibt es logischerweise für Führungskräfte, und das ist auch nicht immer einfach. Weil da sind mal ganz viele der Sozialromantik, muss ich dafür sorgen, dass es denen gut geht, muss ich Beziehungen zu den aufbauen, muss ich wissen, wie die heißen. Ja, das wäre hilfreich, dass immer bei den Lehrern ja schon viel leichter dort, weil das ist ja schon eure Lebensaufgabe oder mit Kindern, ist auch schon wichtig. Also, Führungskräfte sind da oft ein bisschen unsensibel für diese Themen, und dann bin ich sozusagen über das zu burnout prophylaxe, Seminare, die einfach aus dieser nicht perma kann. Man hat Krankheit und Erschöpfung, und dann werden wir doch ein ich in der Prophylaxe gut aufgehoben, bevor es dann kracht, und dann bin ich sozusagen in die Schule. Jetzt brenne ich gerade für Lehrer und für für Kinder und hätte so gern das PERMA lead.Also, ihr seid ja auch alle Mitarbeiter, und ihr habt ja auch alle einen, und das wäre doch toll, wenn man sozusagen euch führen würde wie ihr, eure Kinder, und dann wäre für diese psychosoziale Gesundheit schon viel getan, wobei es ist, jeder Schritt finde ich großartig, wenn man auf sich aufpasst, wenn man lernt, auf andere aufzupassen, geht in beide Richtungen, und darum mache diesen Master sehr enthusiasmiert. Er, weil ich gerne in Richtung Lehre, Ausbildung gehen würde oder eben Direktoren Schulungen machen würde. #00:57:04-3#

B: Und Begleitung, ja, sicher wäre ganz, ganz wichtig. #00:57:06-8#

I: Mhm und mach eben auch hier in die Schule im Aufbruch! Wir dürfen Schulen im Aufbruch unterstützen auf freiwilliger Basis. Wir haben jetzt einfach, ich komme aus Voralberg, sechs Direktoren meinen Teams Volksschule, aber es wäre auch gemischte interessant. Die haben einfach drei Jahre Zeit, so ein neues Multi Schulentwicklungsprogramm mit unserer Führung. Aber wir müssen eher für den Prozess als für die Inhalte. Aber da passiert auch vernetzen, da passiert auch. Die sind nicht alleine, jeder hat eine tolle Idee. Keiner kann das Rad Neuerfinden bei der ganzen Belastung, die auf euch liegt und warum nicht Copy passt, schauen wir mal, was machen andere gut? Da kommt wieder das Perma rein, was machen andere gut, und was können wir davon einfach nachmachen für uns copy paste und übersetzen. #00:57:56-4#

B: Ja. #00:57:57-5#

I: Und da und da führend drum habe ich mich sehr gefreut, dass sie sich, weil ich meine ich auch nicht selbstverständlich, dass man sich darüber hinaus noch eine Stunde Zeit nimmt, um das Programm zu verbessern, damit die nächsten, die wieder durch diese Schulung durch dürfen, nicht vielleicht Dinge doppelt hören oder einfach einfach um sie nicht zu verlieren. #00:58:17-5#

B: Ja, nein, für mich war das auch sehr nett, und jetzt habe ich auch viel Zeit, muss ich sagen, immer am Vormittag, alles passt, schon eine eine nette Abwechslung. #00:58:26-1#

I: Ja, das ist schön. Ich danke ihnen ganz herzlich, wünsche ihnen alles, alles gute, und Wer weiss vielleicht, kreuzen sich unsere Wege wieder, aber in der Schule. #00:58:35-0#

B: Bei der positiven Psychologie. #00:58:36-8#

I: Okay, gut, vielen dank. #00:58:39-6#

B: Danke. #00:58:41-4#

I: Wiederschauen und wiederhören . #00:58:47-3#
