# Supplementary material for: PERMA.teach: a study on the effectiveness of a standardized positive education training program in Austria
Source: Front Psychol. 2025 Apr 8;16:1516572. doi: 10.3389/fpsyg.2025.1516572 (PMC12053473; doi:10.3389/fpsyg.2025.1516572)
Supplement: Supplementary file 2 [file Data_Sheet_1.ZIP › Material PERMA.teach/Material LPs/Interviews LPs Teil 4.zip!/Interviews Transkription/Interview 3 - transkribiert.docx]

**Interview 3.m4a**

I: Genau, Frau O.! Ganz kurz zu den Fragen: Es geht wirklich nur darum, sie haben an diesem PERMAteach Programm teilgenommen. Können sie mir jetzt für die Statistik sagen, wie lange sind sie schon Lehrperson und in welcher Schule? #00:00:17-4#

B: Also, ich bin jetzt in meinem 26. Dienstjahr. Sie bin seit 1997 an der Mittelschule Tux. #00:00:27-5#

I: Okay. #00:00:28-7#

B: Und das war meine erste Schule und meine einzige. #00:00:33-9#

I: Wie schön. Dann sind sie verwachsen! Haben sie alleine an diesem PERMAteach Programm teilgenommen, oder haben sie Kollegen aus ihrer Schule mitgenommen? #00:00:42-7#

B: Na, da ist die ganze Schule beteiligt. Wir sind ganz eine kleine Schule mit, ich weiß jetzt gar nicht genau, zehn oder zwölf Lehrer. Ich glaub zehn Lehrer, so grob. Eben. Und ungefähr 70 Schüler. Also klein und fein. Und da sind jetzt alle dabei. Ja! #00:01:00-9#

I: Das ist schön, dass sie nicht alleine gelassen wurden. Und ich habe gehört, sie waren gerade, also die Tuxner, haben eine Sonderurlaub im Mai. Wie gibt es denn das? #00:01:08-0#

B: Diesen Sonderurlaub, also diese Sonderferien, hat es in Tux eigentlich von Anbeginn an, also seit ich dort drin war, immer gegeben. Wir haben eine reine Tourismusgemeinde da drin mit dem Gletscher, und jetzt hat dieses Thema Herbstferien das ist für die Tuxner im Endeffekt ein rotes Tuch, weil da hat ja niemand Zeit, da ist Hochsaison in Tux. Und was tun jetzt die Kinder Ende Oktober Anfang November ? Das war eben uninteressant, und dann hat sich eben diese Idee entwickelt mit diesen Sonderferien. Wir haben jetzt bis vor kurzem einfach im Herbst imme eine Woche früher gestartet. Und nachher haben wir die Möglichkeit gehabt, so zwischen Christihimmelfahrt und Pfingsten, je nachdem, wie das jetzt vom Datum her gefallen ist, diese Woche dafür investiert und da sind sich manchmal wirklich zwei schulfreie Wochen ausgegangen, und das waren die speziellen Tuxer-Ferien. Und dann ist diese Verordnung dahergekommen, so quasi alle dasselbe, jetzt mit den Ferien. Dann haben wir das ein Jahr lang ausgehalten und dann hat sich wieder so ein Hintertürchen aufgetan. Also, wir starten jetzt normal wie alle anderen Schulen auch ein Herbst, das gleiche Schulbeginn, aber wir arbeiten drei Tage in der Karwoche, Montag, Dienstag, Mittwoch ist Unterricht, und jetzt haben wir heuer zum Beispiel jetzt vor Christi Himmelfahrt, diese drei Tage Montag, Dienstag, Mittwoch haben wir schon hereingearbeitet, und deswegen hatten wir jetzt da wieder ein paar Tage frei. Da war Tux wie ausgestorben! #00:02:37-6#

I: Aber man muss eben der geographischen Gegebenheit einfach nachgehen. Man kann natürlich nicht, wie alle tun. Das ist schwierig in einer Tourismusgemeinde, wenn man wirklich ... Ferien hat, wenn keiner sie braucht. #00:02:51-4#

B: Na eben. Mhm, ja. Sehr wichtig. Deswegen geniessen wir das alle, denn das ist recht herrlich. Recht fein. Ein idealer Sommerurlaub. #00:02:59-0#

I: Kurz bevor einem die Luft ausgeht. Mein. #00:03:04-5#

I: Sehr schön. Das war jetzt nur wegen meiner eigenen Neugier. Ich dachte, da muss ich nachfragen. #00:03:09-9#

I: Also, nur zur Frage, die klassische, was hat sie dazu bewogen, Lehrkraft oder Lehrerin zu werden? Können sie sich noch erinnern? Was war ihre Vision, ihre Mission? #00:03:21-2#

B: Ähm, also irgendwie war für mich, also diese pädagogische Akademie, eigentlich eine Notlösung. Ich hätte irgendwie gern etwas und zwar in künstlerische Richtung eben gemacht, und nach der Matura hat sich da nicht wirklich so ganz klar was aufgetan, es hat sich nicht wirklich was ergeben. Und dann habe ich mir gedacht, ja, was könnte für, könnte den machen, wo bisschen eben Kunst auch vorhanden ist, das tu ich nämlich gern. Und so bin ich eigentlich auf diese Pädak geraten und wollte eigentlich nach dem ersten Jahr wieder aussteigen, und da es mir aber irgend wo Spaß gemacht hat. Und, ähm, noch ein paar neue Kontakte irgendwo geknüpft. Und dann, damals war eben die Ausbildung noch absehbar, also, das hat ja nicht lang gedauert. Das war nur diese drei Jahre damals. Und dann habe ich mir gedacht, das mach ich jetzt mal fertig. Ja, und dann bin ich da drin in Tux gelandet, und da war ein ganz ein junger Lehrkörper. Unser ältester Lehrer, als ich angefangen habe, war 40, das war der älteste. Jetzt war das so eine coole junge Truppe, und da war das einfach so klass, da hab ich mich total total aufgehoben gefühlt, und das hat dann absolut für mich gepasst. #00:04:34-6#

I: Mhm. #00:04:34-9#

B: Ja. #00:04:35-6#

I: Da sieht man, wie wichtig die Beziehungen im Leben sind. #00:04:38-0#

B: Ja, ja. Natürlich. Und das Plätzchen, wo man ist und dass man sich einfach wohlfühlt. Das ist sicher total wichtig. Jetzt, was die Arbeit anbelangt. #00:04:47-5#

I: Ja, genau. Das ist ja auch ein Bestandteil des PERMAS. #00:04:51-6#

B: Genau. #00:04:52-4#

I: Freut mich das zu hören! Ja! Kurze Frage zu ihrem Vorwissen. Haben sie von positiver Psychologie in ihrer Ausbildung schon gehört? Haben sie PERMA schon in irgendeiner Form gekannt oder positive Education, positive Bildung? #00:05:07-5#

B: Ähm, also, jetzt von der Ausbildung her könnte ich mich nicht entsinnen, dass da so groß über dieses Thema irgendwo gesprochen worden ist. Da kann ich mich nicht entsinnen. Mmh. Was wir jetzt schon haben, jetzt gerade in Tux drinnen, also ich rede jetzt von mir. Ahm. Wir haben äh, also wir waren früher eine sogenannte Land-Hauptschule mit verkürzter Stundentafel, und jetzt waren die Pflichtstunden für die Schüler total reduziert, und durch diese verkürzte Stundentafel hat es jetzt Stundenkontingente gegeben, die wir für ganz spezielle Dinge haben nutzen können. Und da war, da kann ich mich jetzt zum Beispiel erinnern, da hab ich immer wieder einmal ah, eine Gruppe gehabt, Schüler, da habe ich dann am Nachmittag - und das hat sich über ein paar Wochen erstreckt, da haben wir zum Beispiel autogenes Training für Schüler gemacht. Da habe mal eine keine Ausbildung genossen und habe dann dieses Projekt zum Beispiel durchgeführt. Und da hat es ganz viele, ich kann mich da nur mal erinnern, als unser alter Direktor in Pension gegangen ich, haben wir mal Projekte zusammen geschrieben, die gemacht worden sind in den 10 bis 15 Jahren und da waren, über 300 verschiedene Projekte. Und jetzt im nachhinein betrachtet, könnte man da ganz viele zuordnen, die in diesen PERMA Bereich wunderbar hineinpassen. #00:06:29-9#

I: Auf diese 5 Finger. #00:06:33-1#

B: Ja, genau. Ohne dass man jetzt diesen Begriff gekannt hat. Aber es ist so im Laufe der Jahre ganz viel eigentlich gemacht worden, nachhaltige Sachen, die jetzt wirklich bei den jungen Leuten hängen geblieben sind, also die jetzt weg von diesem schulischen. Das ist jetzt nur ein Beispiel, das fällt mir jetzt zum Beispiel grad ein, wenn ich jetzt angeboten bekomme, ich kann jetzt mit Gleichgesinnten am Samstag eine Skitour machen, nur wir fünf 5 mit einem Lehrer irgendwohin. Das kann man alles wieder in Verbindung bringen, wieder mit PERMA! Da ist jetzt kein Druck, kein Stress, da wird auf den Schwächeren Rücksicht genommen, man hat ein gemeinsames Gipfel Erlebnis, man ist in der Natur, man spürt sich, man bewegt sich, und so weiter. Also, allein solche Dinge zum Beispiel ist, gehört alles zu PERMA. Also, bewusst jetzt kennengelernt, also in der Theorie dazu, habe ich PERMA erst jetzt wirklich mit dem Projekt. #00:07:34-5#

I: Okay, aber es scheint so, dass sie schon viel davor investiert haben. Das ist ja das schöne, dass die positive Psychologie und PERMA, das sozusagen nur ein Teil aus dieser, oder ein Akronym, dass es leichter zuordenbar macht und übersetzbar macht. Aber das gute ist ja, dass man bei den Dingen forscht, die gut funktionieren. Und man sieht: sie beschreiben es ja auch so wunderbar. Instinktiv macht man die Dinge ja richtig mit seinen Freunden, mit seinen, die guten Momente, also, wo man nicht drüber nachdenkt, macht man es eh. Wenn man es dann als Intervention dazu bekommt, oder also keine Ahnung, man hat drei Stunden man geht, und wenn man jetzt im nachhinein es zuordnen möchten, den fünf Fingern, geht es eben immer, weil genau das tun ja gesunde Menschen, gesunde Beziehungen anders. Wenn man es Instinktiv richtig machen, sie immer dort, und die positiven, die jetzt eigentlich nur bei denen beobachtet, wo es gut läuft. Was machen denn die also können, nehmen also es andere, die es nicht wissen, wie es geht. Also, Lernen von den Besten, und in dem Fall gehört die Tuxer Schule schon dazu, dass man da einfach Instinktiv ganz gut in das PERMA schon investiert hat. Also, sie sagen aber, sie haben von PERMA, also so als Konstrukt erst durch die Ausbildung jetzt gehört? #00:08:49-8#

B: Ja! #00:08:50-6#

I: Wie kam es zu dieser Ausbildung? Wie sind sie ausgewählt worden? Haben sie sich da selber engagiert? Oder? #00:08:57-2#

B: Das war jetzt der Vorschlag von unserem Direktor. Also, wir haben wieder nach neuen Dingen Ausschau gehalten, die uns als Schule jetzt weiterbringen. Wir haben Schwerpunkte versucht zu finden, die jetzt eben uns im Tuxertal irgendwo entsprechen und was soll so auffällig ist, was wir jetzt wirklich machen könnten, und das sind eben dieses PERMA aufgetaucht. Dieses "Stop Mobbing", kann ich mich noch erinnern als Schlagwort bei der Konferenz wirklich angesprochen worden. Und unsere "bewegte Schule", das waren eine zeitlang unsere Schwerpunkte. #00:09:39-1#

I: Da hat es ganz gut reingepasst, diese Fortbildung? #00:09:41-9#

B: Ja, dort hat das wunderbar reingepasst, und wir haben auch einfach beobachtet, dass dass das auch etwas sein könnte, was wirklich eben für unsere Schule - das wird warscheinlich in jeder Schule passen - aber jetzt für uns sehr gepasst hat. Es hat hatten immer wieder mal gröbere Konflikte gegeben, und es haben oft so die Kollegen gejammert "da grüßt keiner mehr", ähm, die Stimmung ist schlecht. Oder da gibts wieder da einen "Mobbingfall", unter Anführungszeichen und so. Und da haben wir einbisschen Ausschau gehalten: "was könnte man machen?" Und dann hat es vor allem eine Kollegin gegeben, ah, die hat jetzt privat eine Ausbildung gemacht, eben in Richtung Journaling. Also persönlich diese Schreiberfahrungen eben für sich selber, sie ist eine Literatin im Grunde und hat das dann immer wieder mal mit den Kindern ausprobiert. Und dann hat eben die Claudia oft gesagt: "Mei, das wäre was. Das wäre was. das bringt so viel. Das bringt so viel." Und so sind wir dann in unserem kleinen Lehrkörper, da ist alles so übersichtlich und klein, da kann man sich ganz gut austauschen, und das so in der Pause, da kriegt man ganz viel mit von allen möglichen Kollegen. Und dann haben wir uns eben für diesen Schwerpunkt entschieden. Ja.... #00:10:56-8#

I: Super, und wie würden sie den Unterschied jetzt ganz schnell festmachen zum Regelschulwerk oder Regelschul-Unterricht und ihren neuen Erkenntnissen, dieser positiven Bildung. Kann man das in ein paar Worten ...? #00:11:08-8#

B: Das. Das. Also. Man kann die Veränderung, die sich jetzt ergeben hat oder, Frau Thurnher, ob man das was feststellen kann ? Ja. Also, das bin ich überzeugt, da bin ich überzeugt, dass sich da was getan hat. Wir besprechen das auch jedesmal, bei jeder Konferenz wird das zum Thema irgendwo gemacht: "Tut sich was?" "Merkt man was?" Und ich muss ganz ehrlich sagen, also ich bin von PERMA total überzeugt, und ich will eigentlich - jetzt zu ihnen gesagt - nur noch so in diese Richtung arbeiten. Also ich habe jetzt auch - sicherlich das Fachliche gehört dazu, das muss ich natürlich machen, das ist auch wichtig, Aber in erster Linie will ich, das ist mein oberstes Ziel, dass sich meine Schüler einfach wohlfühlen, dass es denen gut geht. Und ich glaube, da hat sich dadurch das wir wirklich festgelegte PERMA Einheiten haben. Also, wir haben uns wirklich für einen Plan entschieden. Der hängt im Konferenzzimmer: verpflichtende PERMA-Einheiten pro Woche mindestens drei Einheiten. Das kann oft nur ganz kurz sein oder auch länger sein. Und dadurch fühlt sich jetzt jeder jetzt wirklich verpflichtet, auch in seinen Stunden einfach auch was zu tun. Und wenn man da so lange konsequent dabei bleibt, dann, dann verändert das einfach die, die Gruppe zum Positiven. #00:12:34-8#

I: Das heißt, sie haben eigentlich die Erwartungen ... Hatten sie eine Erwartung, bevor sie in diese Ausbildung rein sind? Haben sie da irgendwie - weil sie sind ja jetzt so überzeugt - heißt das, dass sie wussten, was sie tun, oder hat sie das einfach auf dem Weg dann begeistert? #00:12:48-2#

B: Also, das hat sich so irgendwo entwickelt. Natürlich, zuerst im ersten Moment. Dann ist die Literatur daherkommen und so weiter, und die Fortbildungen. Und da waren schon auch natürlich der Gedanke "Mei, jetzt wieder was neues!" "Ma, was kommt jetzt da wieder daher?" Aber sich einfach darauf eingelassen und fest gestellt. Ahm. Da waren noch so nette Erlebnisse, einfach zu Beginn dabei, wo wir wirklich jetzt aus dem "Jugend stärken,"-Heft etwas heraus genommen haben, einfach probiert haben. Was da so nett war, zum Beobachten war einfach, wie sie tun die Kinder und wie das jetzt, was das jetzt für einen Spaß gemacht hat. Ich kann mich einmal so wunderbar daran erinnern, ich hab alles müssen fotografieren und ausstellen, es war herrlich! Eine Mandarine haben wir mal gegessen, eine ganze Stunde lang. Mandarinen-Meditation. Ja, das war einfach so herrlich. Das hängt heute alles noch in Tux drinnen. Also, das war, das war einfach herrlich! Und da waren so Episoden, einfach, wo man denkt, das macht so einen Spaß oder das bringt so viel! Das ist jetzt so nett gewesen, dass sich da die Begeisterung irgendwo so entwickelt hat. #00:13:48-8#

I: Mhm, mhm, Das klingt nämlich so, also das Schöne an PERMA ist ja, dass es nicht nur sozusagen ein PERMAteach ist, im Sinne, oder ein PERMAlearn ist, sondern auch für einen selber ist, oder ? Wechselwirkung? #00:14:00-0#

B: Ja, natürlich! #00:14:00-1#

I: Sie machen ja jetzt den Kindern eine Freude, oder versuchen ihnen eines dieser Ps E R M A zu entringen und haben selber die Freude. Den Sinn. ..... Also, sehr schön, diese Wechselwirkung. Ich sehe es ihnen gerade an. #00:14:17-7#

B: Ja #00:14:18-1#

I: Ja großartig. Das heißt, sie haben also .... Für sie ist der Unterschied zwischen Regel-Schulwerk und positiver Bildung völlig klar? #00:14:27-0#

B: Klar! Ja, ist für mich klar. Ich sehe nämlich, ich sehe nämlich jetzt durch meinen Sohn, der ist 13, der ist in einer Mittelschule. Ah. Und ich ertappe mich ganz oft dabei, dass ich mir denke "Mei, ich würde mir das so für´n Leo wünschen. Ich würde mir das so wünscnhen. Der würde das so brauchen. Für den wär das so super. Ja, da denke ich mir einfach "schade, dass er das nicht so genießen kann." Jetzt einfach weil, weil die Schule hat, andere Schwerpunkte. Die hat natürlich ihre Qualitäten, aber die, diese positive Psychologie, die wäre jetzt gerade für alle Kinder wunderbar. Jetzt gerade, für die Kinden die sich so suchen und und sich nicht so leicht tun mit sich finden, so, diese Pubertierenden. Da sehe ich gerade das allergrößte Potenzial! #00:15:13-7#

I: Damit habe ich die nächste Frage. Die kann ich mir schon schenken, aber dennoch: Wie würden sie ihre aktuelle Motivation beschreiben, PERMAteach in der Schule umzusetzen? #00:15:25-0#

B: Also, ich ertappe mich immer wieder einfach auch bei meinen spontanen Ideen. Gestern war wieder, gestern hab ich zwei Supplierstunden, zum Beispiel gehabt: Mathe, hmm. Was tu ich jetzt in zwei Stunden Mathe? Und dann habe ich mir schon wieder gedacht, so, Stärkenmappe raus und dann nochmal haben wir mit der Stärkenmappe gearbeitet, und am Schluss hab ich ihnen nich zwei kleine Kurzfilme anschauen lassen. Ja, so nett. Das ist ja auch wieder Richtung PERMA gegangen. Und da filtern wir uns jetzt einfach was heraus für unseren Tag heute. Eine Aktion, so dieses täglich, diese Pfadfinder Regel da, eine "gute Tat pro Tag" also das, oder einen guten Gedanken. Soetwas versuche ich ganz oft. Das sie das einfach am Schluss von meiner Stunde oder aber auch schon zu Beginn. "Wie fangen wir denn heute an?" "Was wünschen wir uns denn heute?" das wir einfach mit dem auch so starten. Oder so spontane Ideen. Ich schicke sie also auch so in meinem Umfeld weiter. Jetzt hat auch mal unlängst mal eine Freundin - es ist herrlich gewesen - , in der Früh einmal von Max Raabe "Guten Tag liebes Glück", das Lied, geschickt. Ich hab das gleich in die Schule getragen und wir haben es alle dann gleich in der Schule angehört. Ich finde es herrlich: "Komm herein, bleib da, setzt dich nieder!" Ja, einfach so spontane Ideen und so Kleinigkeiten sind´s oft einfach. #00:16:40-4#

I: Es baut sich auf, oder? Es wächst ja! Man sieht dann immer mit der Taschenlampe, sie leuchtet auf diese Dinge, und dann kann man das PERMA für sich selber ja weiter aufbauen oder Material weiterfinden. #00:16:49-9#

B: Ja. Ja, auf jeden Fall. #00:16:51-8#

I: Die Frage zu: Fortbildung für andere empfehlen können? #00:16:56-7#

B: Absolut ja. Also das, das würde ich mir für alle Schulen irgendwo wünschen, dass das überall Platz findet. Und ja! #00:17:06-8#

I: so rentiert sich der Zusatzaufwand, den man dann doch machen muss. Jetzt, vielleicht helfen sie uns, dass wir unsere Ausbildungen verbessern können: Diese Schulungs-Bausteine, die Theorie, die Praxis? Gab es da etwas, was besonders für sie wichtig war? Gibt es etwas, was sie sagen "Das war vielleicht nicht so prickelnd?" Könnte man weniger, kürzer? #00:17:27-9#

B: Mhm also, ich muss sagen, das war jetzt, ich bin jetzt wirklich 25 Jahre im Dienst, also diese Fortbildungen mit den Damen, also, das waren für mich die allerbesten Fortbildungen, die ich je besucht habe. Das muss sie wirklich suchen, weil jetzt auch so der Aufwand für den Lehrer nachher in der Umsetzung hat sich absolut einfach in Grenzen gehalten, weil so viel, so unbeschreiblich viel fertig ist, total bäriges Zeug einfach da war, also alleine diese PERMA Post, und auch so, also dieser Aufwand und da ist schon so viel im Hintergrund gearbeitet worden. Also, das war für mich phänomenal. Und man kann das Buch auch aufschlagen, das Buch mit Stärken, kann ich aufschlagen, egal auf welcher Seite. Das kann man einfach alles eins zu eins umsetzen. Und das finde ich, immer das ist so so wichtig, dass du einfach so viel schon für die Praxis einfach da ist. #00:18:19-5#

I: dass der Aufwand nicht mehr so groß! Für die, die .. Umsetzung. #00:18:22-0#

B: Na eben! Dann tut man es nämlich auch. Da ist es auch für die Lehrer auch einfach viel leichter in der Umsetzung. Wenn du jetzt wieder irgendwo überlegen musst, was könnte ich denn jetzt machen? Ah. Ja, da fallen dann schon wieder ganz viele weg, die diesen Aufwand einfach nicht haben will oder nicht verkraften, irgendwo. Und so ist einfach so total viel da. Das muss ich sagen. Das war jetzt wirklich toll! #00:18:43-3#

I: Also, da waren sie begeistert! ich habe jetzt hier noch die einzelnen Punkte: Die Präsenz war ... sie waren in Präsenz oder virtuell bei der Schulung? #00:18:53-4#

B: Beides. #00:18:58-7#

I: Was war für sie angenehmer? #00:18:58-7#

B: Kann jetzt gar nicht wirklich sagen. Das war beides, also durch das, dass man auch jetzt, wenn wir jetzt online waren, hat es auch genauso Arbeitsaufträge gegeben, und man hat sich in der kleinen Gruppe dann getroffen. Also, das war irgendwo ähnlich. #00:19:13-9#

I: Und die Vernetzung mit den anderen Teilnehmern ? War das für sie ein Faktor, der sie begeistert hat? #00:19:22-4#

B: Das ist jetzt für mich immer so ein bisschen ungut. Das tue ich jetzt nicht so furchtbar gern. Wenn es jetzt heißt, ich kann mich ans letzte, also die letzte Fortbildung erinnern, im Chatroom Nr 8, so mit 3 Teilnehmer.Jetzt war die die zweite Frau gar nicht da, so jetzt war ich mit so einem Herren alleine. Ahm. Ja, das war mir jetzt irgendwie, da hab ich mich so gefühlt, so quasi jetzt ein Date. Na, das war für mich ungut. Also, wenn das jetzt die größere Gruppe, wenn jetzt fünf, sechs oder so sind. Ok. Dann sagt der was, dann sagt der. Aber so jetzt in diesem Zweiergespräch, war mir das irgendwo ein bisschen unangenehm, muss ich sagen. Also ich persönlich tue das nicht so gern. Da bin jetzt lieber, wirklich, da wäre ich jetzt lieber präsent, als wie jetzt da so über den Bildschirm. Da fühle ich mich jetzt also, wenn jetzt wirklich in der kleinen Gruppe, dann ist mir das ein bisschen unangenehm. Das tu ich jetzt nicht so gern! #00:20:20-6#

I: Da schauen wir mal, das wir die Gruppengrößen beachten. Ahm. Die schriftlichen, Handouts - das haben sie schon gesagt - die haben ihnen, haben sie begeistert. Weil die Informationen für sie wirklich sofort umsetzbar waren, oder? #00:20:33-0#

B: Ja, absolut ! #00:20:34-8#

I: Die Inspirationsquellen, die dann da drauf waren, wie z.B. die Apps, oder die Youtube VIdeos - haben sie diese verwendet ? Sind sie da, kann man die ... ? #00:20:45-0#

B: Ahhhm. Die App selber. Also, da muss ich jetzt dazu sagen, also weniger weniger. Weil ich bin irgendwo noch so einbisschen vom alten Schlag. Also, ich habe nach wie vor lieber. Ich tu mir auch - das muss ich jetzt ganz ehrlich sagen - auch mit diesem IPad in der Schule, also, das kommt bei mir jetzt eher selten vor, oder nur für einbisschen so Recherchezwecke. Ich tue das nicht gern. Ich habe lieber irgendetwas in der Hand. Tu lieber blättern oder wirklich mit meinen Händen was machen. Mir sind jetzt die Bücher lieber! Ja, die sind mir lieber. #00:21:19-4#

I: Die nächste Frage hängt zusammen mit dem IPad, da gibt es auch noch das Padlet Haben sie das verwendet? #00:21:31-1#

B: Nicht. Nein. Gar nicht. #00:21:33-7#

I: Okay, aber nur damit sie es wissen: Man kann ja auch später noch einmal darauf zugreifen, falls ihnen die Inspirationsquellen ausgehen. #00:21:40-8#

B: Aha. #00:21:41-2#

I: Ahm. Jetzt ganz noch ganz kurz zu diesen Rahmenbedingungen wie E-Mails, Einladungen, zeitliche Terminisierung. Wie war das für sie? Hat das gut funktioniert? #00:21:51-4#

B: Das hat sehr gut funktioniert, also, das war total problemlos. Rechtzeitig. Also, das hat perfekt gepaßt. #00:22:03-7#

I: Und die Frage jetzt noch, ob es etwas gegeben hätte, dass sie vermisst haben oder das mal weglassen hätte können? #00:22:12-3#

B: Na, da kann ich mich nicht entsinnen. Also, es es war immer sehr kurzweilig, interessant, viel aus der Praxis. Das hat alles Hand und Fuß gehabt. #00:22:26-4#

I: Sehr cool! Dann habe ich noch eine konkrete Frage zum Stärken-Cafe: das hat ja dann auch immer regelmäßig stattgefunden. Jetzt nehme ich an, diese eine Geschichte, diese Episode mit dem Herrn war dort beim Stärken-Cafe. #00:22:43-1#

B: Genau. Ja. #00:22:43-8#

I: Aha... Also dann frage ich jetzt: gibt es sowas wie ein Ritual, das sie in der Schule machen, das vielleicht als ähnliches Wiederholungs Meeting bezeichnen könnte, dass ihr euch intern, also ihr euch in der Schule, austauschen könnt? Gibt's sowas, wo sie so ein Ritual eingesetzt haben? #00:23:02-4#

B: Also nicht offiziell, nicht wirklich. Natürlich sind spontan Ideen entstanden in den Fachgruppen. Das muss ich jetzt schon sagen, weil es einfach alles so klein und fein ist bei uns. In Deutsch, also im Deutsch-Unterricht zum Beispiel gibt es nur drei Lehrerinne. Da muss ich schon sagen, das ist jetzt so eine kleine Gruppe, und da sind jetzt alle drei wirklich von dem PERMA so begeistert, dass man uns wirklich ganz oft einfach austauschen und sagen: "Mei, da habe ich jetzt was probiert oder das hab ich jetzt gesehen, das musst du machen, das ist total nett!" Das schon. Jetzt in der Fachgruppe. Aber im Grunde eigentlich durch das, dass man noch im Team-Teaching viel miteinander unterwegs sind. So passiert das oft auch wirklich im regulären Unterricht das wir fünf Minuten haben, wo wir sagen, so "Mei, das habe ich gesehen!" oder "Das müssen wie bestellen für die Bücherei!" oder dies und jenOder am Gang. Oder einmal bei einer Aufsicht oder so. Da wird eigentlich in der Fachgruppe viel darüber geredet, dass sind jetzt die Deutsch- Damen sozusagen. #00:24:14-4#

I: Da braucht es sozusagen gar keinen Jour Fix, weil ihr sowieso im Austausch seid? #00:24:19-5#

B: Ja, genau. #00:24:20-4#

I: und weil sie jetzt die per PERMA Post vorher, die haben sie zwar voher schon erwähnt, ich habe jetzt nicht extra noch einmal abgefragt: diese wöchentlichen Emails, die kommen, als Erinnerung teilweise. Natürlich. Sie sind jetzt so begeistert, dass sie diese wahrscheinlich gar nicht brauchen. Aber viele ziehen dort Erinnerungen raus beziehungsweise wieder neue Inspiration. Ähm, können sie das brauchen die PERMA Post ? #00:24:43-9#

B: Die PERMA Post finde ich toll. Die ist super. Also, da habe ich mir sehr viel herausgenommen, und durch das, dass - so glaube ich, sind die Menschen nämlich oft wirklich gestrickt, wenn da regelmäßig wöchentlich einfach so was daher flattert, wird man das halt so gewöhnt, dass, wenn man nicht so wäre, würdede das schon wieder vermisst. Das ist ja wie das regelmäßige Training. Finde ich jetzt einfach ganz wichtig. Dass also ich habe das genossen, ich habe das herrlich gefunden. Also, PERMA Post habe ich mir alles fein säuberlich sortiert, dass ich da ganz schnell wieder zugreifen kann. #00:25:26-7#

I: Ich muss ganz kurz, der Postbote will mich an der Türe sehen. #00:25:34-5#

B: Ja. #00:25:34-7#

I: Entschuldigen Sie. Der war jetzt hartnäckig und ist einfach vor der Türe stehen geblieben. #00:25:42-6#

B: Alles gut. #00:25:43-4#

I: Sie haben fein säuberlich alles abgespeichert von der PERMA Post und können es jederzeit wieder verwenden? #00:25:53-7#

B: Das verwende ich aufjeden Fall. #00:25:55-1#

I: Schön! Das ist schön, wenn das Material auch geschätzt wird. Gibt es etwas, aus dem sie weniger Nutzen ziehen konnten? Gibt es etwas, wo sie sagen, das hätte ich jetzt nicht gebraucht? Zuviel des Guten? #00:26:09-6#

B: Nein, da wüsste ich jetzt nichts. Da fällt mir jetzt nichts ein. #00:26:25-9#

I: So. Und jetzt die Frage: spüren sie eine Veränderung in ihrem Team ? Also nicht nur in der Klasse. Dort, wo sie sozusagen das auch anwenden oder bewusste Intervention setzen. Gibt es nach hinten, sprich ins eigene Team, offensichtliche Veränderungen, die PERMA vielleicht begünstigt hat. #00:26:45-7#

B: Da würde ich mir jetzt schwer tun. Da irgendwie. Also große Unterschiede zu vorher, könnte ich jetzt nicht ausmachen. Nein. Im Grunde hat es im Team selber, hat das eigentlich immer gut funktioniert, und dass sind jetzt irgendwo die Fachgruppen selber, diese kleinen Grüppchen, die da bei uns so einbisschen- wie soll ich da sagen "existieren oder so ein bisschen die älteren Lehrer ein bisschen untereinander und die Jüngeren aber, dass ich jetzt irgendwie eine Veränderung, groß feststellen könnte oder im Lehrkörper selber kann ich nicht sagen #00:27:39-3#

I: Ich meine damit so Beispiele wie, dass Konferenzen anders beginnen, oder dass man mehr vielleicht mit der Stärken-Brille auf, in diesen Konferenzen spricht, oder auf Kollegen schaut? #00:27:42-2#

B: Nein, könnte ich nicht sagen! #00:27:55-6#

I: Die Direktion, sie haben gesagt die Direktion war da eigentlich sehr initiativ, und wollte das sie das machen. Hat der oder die Direktorin auch daran teilgenommen, ist sie/er dabei, oder? #00:28:08-0#

B: Unser Direktor ist da voll dabei, muss ich, muss ich sagen, hat da wirklich, war da offen für alle möglichen Ideen. Ah auch die Gemeinde ist involviert worden. Die Eltern haben eine Fortbildung genossen. Das war zum Beispiel ganz wichtig und eine ganz coole Sache, dass es auch, das ist auch von unserem Direktor ausgegangen, dass es auch für die Eltern eine Fortbildung geben hat zum Thema PERMA . Das betrifft ja nicht nur die Schule, sondern es geht ja daheim irgendwo weiter. Das ist jetzt zum Beispiel auch vom Andi ausgegangen. Und jetzt haben wir, wenn man bei uns jetzt im Schulhaus wirklich schaut, da ist PERMA vom Keller bis zum Dach. Ich hab mir jetzt gedacht "Wo finde ich noch einen Platz um noch etwas aufzuhängen. Wir haben jetzt auch große Stelltafeln bekommen von der Gemeinde, so Rahmen, das sind so große Holzrahmen, die haben wir mit Fäden bespannt hinten, da sind jetzt überall große Buchstaben drauf, weil uns einfach der Platz ausgegangen ist, um die Sache ein bisschen zu präsentieren. Das glaube ich nämlich auch, das finde ich irgendwo sehr wichtig. Ich habe heuer so viel getan wie überhaupt noch nie. Das hat der Andi, das ist von der Direktion da wirklich gefördert worden. Einfach Schülerarbeiten präsentieren, das Wertschätzen, was die Kinder machen und was die so leisten. Also da sind wir jetzt auf der Homepage, da hat es eine neue Rubrik gegeben, mit diesen wirklich Schülerarbeiten, und es ist das ganze Schulhaus, einfach voll. Einfach diese Wertschätzung den Kindern und deren Arbeiten einfach gegenüber. Das ist jetzt wirklich total präsentiert, muss ich sagen, und das ist viel von der Direktion ausgegangen. #00:29:54-5#

I: Wie schön, das das A aus PERMA seine Wirkung zeigt im Haus. Überall Leistungen, kleine Zwischenziele. #00:30:02-1#

B: Ja, absolut, also das auf jeden Fall. Es ist kunterbunt. #00:30:06-9#

I: Kunterbunt. Das ist schön, wenn sie sagen, dass sie nicht einmal mehr einen Platz zum mehr aufhängen. Genau. Sie haben viel erzählt, dass sie viel aus dem Buch rausnehmen können, dass es für sie eine Erleichterung ist. Ich höre heraus dass es eine Haltung ist, die sie auch einnehmen, oder wahrscheinlich früher auch schon eingenommen haben, und jetzt nur auch wunderbares Werkzeug dazu haben. Wo genau im Unterricht setzen sie das ein? Können sie das auch in ihrem Fach einsetzen, oder ist es ...? #00:30:41-2#

B: Doch, das kann ich in meinem Fach wunderbar einsetzen. Also, ich unterrichte jetzt Deutsch und bildnerische Erziehung und Werken, Ähm. Und ich ich, ich bin, ich habe natürlich, also ich muss jetzt sagen, ehrlich sagen, dass das Leben, so wie mein Leben jetzt war. Das hat jetzt Stolpersteine gehabt, gravierende, und dieses Leben lehrt einen dann, irgendwo auf die Dinge anders zu schauen. Also diese Achtsamkeit zum Beispiel, die habe ich inzwischen viel mehr noch, wie wie früher, einfach, und ich bin da auch drauf kommen, dass die, wenn man bewusst hinschaut, die Kinder bewusst betrachtet, dann dann dann hat man irgendwo das Gespür, was braucht denn der jetzt genau von mir? Wie kann ich dem denn jetzt so helfen? Und jetzt auch zum bei Beispiel dieses, dieses Lob, das gezielte Lob, einfach wirklich, alleine das, wenn ich jetzt wirklich auch zu einem schwachen Schüler hingehen kann, und sag, also z.B. "dieser Satz, der ist Bombe, der gefällt mir jetzt total" einfach diese Kleinigkeiten oft so zwischendurch, das zum Beispiel tue ich jetzt inzwischen viel mehr wieder, wie ich es schon dazwischen einmal getan habe. Auch jetzt im Fach, oder generell, diesen Stress, den möchte ich heraussen haben aus meinem Unterricht, also den Druck reduzieren, den Stress irgendwo reduzieren, Ähm, das kann ich wunderbar machen in den Hausübungen total reduziert. Reduziert. Es ist oft nicht mehr so sinnvoll für mich, muss ich sagen, also, die Schwerpunkte haben sich irgendwo ein bisschen verlagert. #00:32:22-6#

I: Aber auch in den inhaltlichen - keine Ahnung - in Deutsch z.B. Ich sage jetzt mal, kann man sowas wie eine Inhaltsanalyse nach Stärken machen? #00:32:35-3#

B: Ja, auch. Auch. In Deutsch zum Beispiel durch das, dass wir da wirklich viel im Team Teaching unterwegs sind und die Claudia jetzt diese Ausbildung hat, mit dem Journaling tun wir oft wirklich Gruppen teilen. Die kleinen Gruppen sind da von großem Vorteil. Also, wenn man wirklich nur sieben, acht, neun Kinder hat in der Gruppe, kann man auch ganz anders arbeiten. Das muss ich jetzt auch ganz ehrlich sagen. Wir haben eine Wellness-Oase eingerichtet in der Bücherei, da ist dann "nicht zu stören", da holen wir uns eine Luftmatratze herunter bei der Glastür da ist dann"Wellness" dahinter und diese Einheiten, die machen wir einfach vermehrt. Dieses persönliche Schreiben jetzt mit Gedichten, Arbeiten mit Gefühlen. Einfach jetzt die inhaltlichen Themen. Wenn jetzt irgendetwas aus der Lyrik einmal ansteht. bewusster auswählen, was nehme ich für einen Text, was sollen die Kinder selber produzieren. Das kann man jetzt in Deutsch zum Beispiel wunderbar machen, das muss ich schon sagen. #00:33:37-7#

I: Eben. Da fließt das dann in den Inhalt ein und nicht in den Rahmen. #00:33:44-7#

B: Na, es ist ich auch im Inhalt zu finden. #00:33:47-7#

I: Beim Werken? #00:33:53-7#

B: In Werken, was ich da vermehrt mache, immer wieder. Wenn jetzt Arbeiten abgeschlossen sind, dass man wirklich eine Werkbetrachtung machen. Eine ausführlichere, wo es nicht darum geht, welcher Name steht da hinten oben. Das ich sagen kann, die Sarah hat das super gemacht und das, vom Tobias gefällt mir nicht. Das möchte ich da gar nicht haben. Sondern einfach das bewusste hinschauen und das besondere an dem Bild heraus finden und das auch formulieren. Derjenige, der weiss dann schon, wenn das zweite Bild oben rechts, da weiß derjenige, der es gezeichnet hat schon, das ist jetzt mein Bild. Und was sagen denn die jetzt? Da kommt schon viel. In erster Linie, was hat es für Besonderheiten, was macht es denn einzigartig? Und alleine durch die Fragestellungen wird ihnen da Wertschätzung entgegen gebracht. #00:34:41-8#

I: Höre ich da auch eine neue Feedback Kultur, ein bisschen heraus? Das auch positiv geben zu können. #00:34:51-3#

B: Natürlich! Sicher darf ich schon sagen, das gefällt mir nicht so gut. Aber dann, dann muss ich das begründen. Einfach nur so, und das finde ich nicht gut, das ist nicht schön, dass ist mir dann zu wenig, Weil ... dann darf er das natürlich sagen, Und dann dürfte ich, könnte ich auch sagen, dieses Kind darf vielleicht beim nächsten Man das noch ein bisschen besser machen, der Künstler da. Und so weiter. Das darf man ja sagen. Also, das mache ich jetzt zum Beispiel in Zeichnen und Werken. Und da kann ich natürlich auch in der Themenstellung einbisschen beeinflussen. Natürlich! #00:35:24-8#

I: Ja, PERMA kann in ihrem Fall wirklich überall reinfließen. Dann sehen sie diese Chancen auch. Das ist toll. Gibt es Rituale in ihrer Klasse oder neue Projekte, die sowie als Gewohnheit neu entstanden sind? #00:35:49-7#

B: Ja, jetzt, vielleicht könnte man ein kleines Ritual wird vielleicht zu erwähnen geben, mir haben da was. Es geht jetzt nämlich über schon einige Zeit. Ich würde jetzt sagen, wann haben wir den gestartet, im März haben wir gestartet. Mit einer Sache. Und zwar, das habe ich mir jetzt da hergerichtet. Da ist mir einmal ein Kinderbuch untergekommen. Von meiner Kleinen. Und darf ich das Foto ganz kurz zeigen, jetzt weiss ich nicht: "Loni lacht" Da ist ein lustiges Monster drauf, und der nennt sich, das ist ein sogenannter Bumpf, und der ist so unscheinbar. Aber immer, wenn es ihm gelingt, jemanden eine Freude zu machen, dann kriegt er einen farbigen Punkt. Da wirds einen Bumpf-Punkt, Punkt, und diesen diesen Bumpf zum Beispiel, den hat jetzt jede Klasse in Großformat auf Papier. Also du ich jetzt, die ganze Tür, die Vorderseite, also die Außenseite von der Tür ist jetzt beklebt mit so einem riesigen Bumpf. Da hat jede Klasse ihre eigenen Bumpf gemalt, großformatig, und jetzt haben wir wirkliche Klekse, ausgestanzt aus bunten Klebefolien, und die sind in der Klasse deponiert. In jeder Klasse gibts eine Schachtel mit Bumpf-Punkten, und das hat sich jetzt schon oft entwickelt, dass man - natürlich spontan - wenn im Unterricht was ist, oder in der Pausenzeit, wo man sagt: "Meil Valentina, jetzt hast du mir so nett die Tasche raufgetragen, weißt du was? du bekommst heute einen Bumpf-Punkt" Also, immer so wenn die kleinen Freuden da sind, dann wird geklebt. Und da ist dann oft noch so, wenn man jetzt irgendwo am Ende der Stunde oder am Ende von deinem Vormittag merkt, jetzt heute haben wir noch gar nicht gelobt oder was war denn heute schon, man sieht die Dinge, oft viel zu wenig, was war denn, was ist denn heute gelungen oder was hat denn Spaß gemacht? Oder wo ist denn heute jemanden gut gegangen und soweiter. Wenn man da einbisschen mit den Kinder darüber redet, dann fällt dem einen oder anderen noch was ein und dann kriegen wir wieder unseren Bumpf-Punkt. Vielleicht könnte man das, das geht jetzt wirklich über zwei Monate, wenn man jetzt noch so bis Mitte juni, diese Bümpfe bekleben lassen. Das könnte man jetzt vielleicht als Ritual bezeichnen, sich sich bewusst machen, was hat denn heute Freude gemacht oder was ist denn gelungen, und wofür haben wir denn heute ein Pumpf-Punkt verdient? #00:38:22-5#

I: Mhm, wie schön, dass sie da eine eigene, eigene Bezeichnung dafür sogar gefunden haben und und man wird, Sie trainieren sie darauf, man schaut auf das positive. Wenn es dann so Spiele oder so ein Ritual, zur Gewohnheit wird, dann ist es auch für die Kinder lustiger. Sensationel. Das heißt, sie würden sagen oder Haben sie ihr persönliches Perma verbessert durch diese Ausbildung? #00:38:49-9#

B: Ja, absolut! Das glaube ich schon, weil man einfach jetzt viel mehr auch theoretischen Hintergrund einfach hat und und und die Dinge gezielt und bewusst einsetzen kann. Früher, hat man da gar nicht darüber nachgedacht. Da haben wir auch allerhand sicherlich gemacht. Aber, jetzt nie so so bewusst. Also, ich greife die Inhalte heute wirklich einfach viel, viel bewusster auf und denke immer wieder mal so, und das möchte jetzt heute machen, und das ist einfach aus dem PERMA Bereich einfach heraus, wirklich gezielter wird es eingesetzt, mit mehr Hand und Fuß! #00:39:27-0#

I: Und würden sie sagen, dass ihre positiven Emotionen am Tag sich erhöht haben in der Schule? #00:39:35-9#

B: Ja. Ahhhh und vor allem eine eine Sache, und ich bin so froh, dass ich das wieder zurück habe, ich bin im Grunde so gestrickt, dass mir ahm vieles, ahm, so ein bisschen eine Wurstigkeit habe ich immer gehabt, vieles war mir so insgeheim egal, und das ist zwischenzeitlich so einbisschen verloren gegangen. Da war es so Struktur und ein bisschen so verkrampft oft und so böse und und eher nicht so prickelnd. Und inzwischen bin ich da jetzt einfach wieder zurück, und das ist mein allerschönstes Gefühl. Einfach so ein bisschen die Leichtigkeit einfach wiederhaben, den Humor, drüber lachen, über viele Dinge einfach einbisschen hinweglachen ah, und einbisschen, ah, ich nenne es jetzt einfach die Wurstigkeit. Das ist für mir ein herrliches Gefühl! #00:40:26-6#

I: Das heißt, sie haben wieder zurück zu ihren Stärken gefunden, vielleicht ? #00:40:32-6#

B: Ja! Schon. #00:40:32-9#

I: Leichtigkeit, Humor, ahm. auch Drüberstehen im Sinne von ... #00:40:38-7#

B: Genauso! Auch auch auch nicht so, da ist so wenig, was eigentlich persönlich ist, das Meinen ist ja oft ja, wenn es so Konflikte und so weiter gibt. Es wollen ja oft nicht Schüler nur dir zum fleiß tun du. Einfach dieses! Es ist nicht vieles so persönlich gemeint, und nicht so ernst gemeint. Und auch einfach ahm einbisschen drüber hinwegsehen, über manche Dinge. Einfach leichter leben. #00:41:07-0#

I: Leichter leben. #00:41:08-0#

B: Ja. #00:41:08-1#

I: Das würde heißen, sie hätten also durch durch die Beschäftigung mit PERMA gelernt, aber auch das Gefühl, sie hätten eigentlich für sich selber ihre Stärken wieder gefunden. #00:41:16-9#

B: Ja schon ! und da irgendwo auch, irgendwo bemerkt, dass ich das für mich auch haben will. Nicht nur jetzt für die, für die Kinder, einfach, für die für die Schüler, sondern ich will das ja auch, das das es einfach mir gut geht! #00:41:34-0#

I: Ja, darum heißt dieses Projekt ja auch Wohlbefinden in der Schule und psychosoziale Entwicklung für Lehrer. Wenn wir jetzt gerade beim PERMA durchdeklinieren sind. R für Relation ! Haben sich ihre Beziehungen in der Schule verändert, verbessert? #00:41:54-3#

B: Ja schon, die Beziehungen in der Schule, muss ich sagen, die habe ich schon vor vier Jahren schon neu sortiert, das muss ich jetzt ganz ehrlich sagen. Da bin ich eben schwer erkrankt, ahm, und dann war ich zweieinhalb Jahre im Krankenstand, und und da habe ich mit den Beziehungen gerade in der Schule massiv zu kämpfen gehabt. Einfach, jetzt, unter meinen Kollegen muss ich ganz ehrlich sagen, also, ich war der Meinung, ursprünglich, aber das ist meine Meinung gewesen, meine Einstellung, das sind meine Freunde irgendwo durch das, dass der Lehrkörper so klein ist. Ich bin mit manchen wirklich 25 Jahre beieinander gewesen da drinnen, und nachher, aber das kürze ich jetzt einfach ab. Da haben sich während meiner Erkrankung einfach, ähm, zwei, drei vielleicht heraus kristallisiert, die sich interessiert haben für mich und meine Geschichte, und es hat Kollegen gegeben, da hab ich in meine zweieinhalb Jahren Abwesenheit nicht einmal "Muh" oder "Mäh" gehört. Und da habe ich für mich gedacht, ich muss diese Leute meiden, ich will da nicht mehr. Also ich habe auch Ausschau gehalten nach einer neuen Schule, wo ich hingehen könnte, und bin aber dann doch irgendwo zum Schluss kommen. Es war noch drinnen, eben durch das, dass die Schule so klein ist, hat mir mein Direktor einen wunderbaren Stundenplan entgegen gekommen. Er hat gesagt, "Bitte Verena komm wieder. Komm wieder. Sag du was du möchtest. Und mit wem möchtest du ?" und so weiter. Und das war einfach so verlockend, dass ich für mich einfach beschlossen haben, ich gehe jetzt arbeiten, ich umgebe mich nur noch mit den Kollegen, die mir entsprechen, mir gut tun, und die Anderen sind mir egal. Und das ist jetzt inzwischen für mich so jetzt. Aber das ist jetzt meine Geschichte, und ist einfach so gewesen. Es ist jetzt einfach so, und ich lebe jetzt einfach leicht. Also ich komme mit jedem aus, aber das sind jetzt in dem Sinne meine Kollegen und das sind nicht meine Freunde, bis auf die ein, zwei. Und dann hab ich für mich ist quasi diese Beziehungen neu sortiert, und jetzt passt es für mich wunderbar. Ich mach zum Beispiel, was ich nicht mehr tue, das hab ich früher auch geliebt, Lehrer-Ausflüge. Da haben wir früher herrliche gehabt, da haben wir viel davon gehabt, Gardasee und so weiter. Das möchte ich alles nicht mehr. Also das tue ich nicht mehr. Das möchte ich nicht mehr. Aber ich sehe das, was für mich der ganz große Vorteil war in dieser ganzen Misere, ist einfach gewesen, dass diese ganzen Grauabstufungen jetzt weg sind. Ich sehe nur noch schwarz und weiß, und ich weiß ganz genau, wer tut mir gut, und mit dem umgebe ich mich. Und es sind neue Menschen in mein Leben getreten und so, so ist es für mich in Ordnung. #00:44:33-9#

I: Genau, und das ist natürlich das, was bei PERMA schon auch das ist, also diese, relations, diese Beziehungen auch zu bewerten, also nicht alle, nicht alle zu pflegen und nicht überall Energie zu verbrauchen, sondern zu schauen, was tut mir gut, und andere eher meiden. Und wenn sie das vorher schon geschafft haben: Bravo! Manchmal muss man leider Gottes da durch um zu lernen. ähm, Jetzt, um PERMA weiter zu deklinieren, M wie Meaning: Haben sie mehr Freude in ihrem Sinn, für ihre Schüler da zu sein. Gibt es da vielleicht eine andere Haltung zum Lehrerberuf, zu ihrem Job? #00:45:12-4#

B: Auch auch, und zwar ..... das hat sich auch wieder erst entwickeln müssen, ich investieren sehr gerne viel von meiner Energie in schulische Projekte, die ich jetzt einfach für die, für die Schüler herrlich finde, weil sie da Dinge zum Tragen kommen, die in der Schule nie zutage kommen. Da war heuer jetzt zum Beispiel, da haben wir so ein Musical aufgeführt, Theater mit Musical und so weiter. Und das war für mich so herrlich zum Sehen und auch wie Eltern, da war kurz danach dann Elternsprechtag, wie manche Mütter auch gesagt haben, "ich hätte gar nie geglaubt, dass meine Tochter das kann." Und solche Sachen mach ich jetzt sschon jetzt, wo die Kinder so ohne Druck, ohne also, da geht es nicht um die Schule und da geht es nicht um die Noten, wo sie so aufblühen können und und so Talente zeigen können. Das tue ich zum Beispiel liebend gern! #00:46:13-5#

I: Schön. Ja. #00:46:15-0#

B: Also, so Schulprojekte, da bin ich einfach der Überzeugung, wenn sie so ein Auftritt irgendwo haben in der Gruppe, sich so lang vorbereiten, und jeder ist irgendwo wichtig. Gute Schüler, schwache Schüler. Das spielt keine Rolle, das tue ich jetzt zum Beispiel total gerne wieder. Das ist mit viel Energie verbunden, mit viel Aufwand verbunden, das hab ich jetzt im letzten Jahr zum Beispiel, war das für mich nicht möglich, einfach weil ich das selber nicht geschafft hätte, und inzwischen denke ich mir, ich will eigentlich nur noch sowas machen. Ja! #00:46:44-8#

I: Ja. An ihre Stärken angedockt können sie jetzt die Stärken der anderen aktivieren. #00:46:50-8#

B: Mhm. #00:46:50-9#

I: Und Aufblühen, wie gesagt, geht immer in beide Richtungen. Also nur Geben und selber dabei verkümmern geht nicht. Drum ist es so schön, dass bei PERMA diese Wechselwirkung sichtbar ist. Und das wäre jetzt auch die nächste Frage: was ähm wie würde ein Außenstehender sehen, sie sagen zwar, dass die Eltern eine Schulung bekommen haben, Aber an was erkennen die, dass ihr Unterricht anders ist? Wenn es ein Mars-Menschlein käme und vergleichen würde, an was glauben, sie würde er erkennen, dass sie positive Bildung praktizieren? #00:47:24-0#

B: Ahm. Einmal sicher jetzt, wenn er jetzt in Hefte von den Schülern schauen würde, dass da wirklich vermehrt ahm. Ich habe auch die Farbe gewechselt. Ich mag gar nicht mehr mit rot korrigieren, das tue ich auch nicht mehr. Es ist grün. Jetzt ist alles grün. Und dann sind einfach die Kommentare anders geworden. Früher hat man oft so eben da ist es so gegangen, zehn Fehler hast du gemacht, das kannst du noch nicht, und das kannst du noch nicht. Das einfach die Feedback Kultur, eine andere geworden ist., dass man da wirklich versucht auch, natürlich muss ich sagen, dass war leider jetzt nicht so gut, muss ich natürlich auch sagen. Aber kommt natürlich darauf, jetzt, das hätte ich früher auch nicht getan, aber ich hab jetzt einem Schüler wirklich in ein "nicht genügend" drunter schreiben müssen. Ich hasse das. Aber es ist nicht anders gegangen. Das habe ich eben tun müssen. So jetzt ist der Bub in der 3. Klasse, 13 Jahre alt. Jetzt weint er jämmerlich dahinten. Zuerst habe ich mir gedacht ich weine gleich mit. Also bin ich hin und hab gefragt: "Warum er weint?" "Ob er selber so enttäuscht ist?" oder "was jetzt ist?" Ja, er weint wegen der Mama eigentlich. Die Mama ist aum jetzt ein, ich war selber so in deutsch ist oder was sie ist, ja wegen der Mama eigentlich, weil die Mama, die schimpft jetzt, wenn er nach Hause kommt mit einem 5er. Und dann hab ich sagen müssen, zu dem Bub. "Weiß du was Jakob? Ich ruf jetzt deine Mama an. Und ich mach das jetzt für dich. Ich erkläre das jetzt der Mama. Und die Mama, das garantiere ich dir, schimpft heute nicht mit dir. Sie schimpft nicht mit dir. Ich erkläre ihr jetzt was das Problem war und so weiter. Und danach werde ich der Mama sagen, sie soll dir heute ganz was gutes kochen und vielleicht auch noch eine Nachspeise, weil traurig bist du ja auch und so weiter. Und da darf man nicht noch einmal eine auf den Deckel bekommen. Solche Dinge zum Beispiel, das hätte ich jetzt früher, sie hätten mich auch getroffen, ich hätte mir auch gedacht "ahhh, blöd oder was. Aber alleine, dass ich danoch initiativ werde offen hätten oder was nicht, aber dass sie doch noch initiativ werde, und mir denke, so, das nehme ich jetzt dem Kind ab. Oder das jetzt mit der Lisa, wo ich einfach merke, dass sie in der Klasse, so einbisschen eine Außenseiterin ist. Sie klammert sich sehr an mich, die bringt mir oft so Zeug von zuhause mit und zeigt mir das und so weiter. Und der einfach das Gefühl geben und das Zeug herzeigen lassen und sagen "Lisa, ahm, total nett, dass kann ich zum Beispiel gar nicht. Wie hast du das gemacht?" Also, sich da einfach viel mehr persönlich einbisschen eingelassen auf die Schüler, das tue ich jetzt mehr als früher, ist, das muss ich schon sagen! #00:49:45-5#

I: Und an dem würde man den Unterschied sehen? #00:49:49-8#

B: Das ist sicher ein Unterschied, einfach ja, zu früher, eben: die Korrekturfarbe, das positive Feedback irgendwo, ja, und einfach so, dass die Menschen einfach mehr sehen, ja. #00:50:07-4#

I: Schön, ihnen zuzuhören, Frau O. Ja, sie sind. Ja, da ist jetzt warscheinlich genau das Samenkorn auf den richtigen Boden gefallen, zur richtigen Zeit. Ja, jetzt habe ich noch zwei Fragen an sie, nämlich, ich hab schon gefragt: wem, ob sie es empfehlen würden. Sie haben gesagt, ja. Gibt es jemanden ganz speziellen? Aber bei ihnen ist ja die ganze Schule schon dabei. Dann vielleicht noch die letzte sachliche Frage: was ist der Hauptnutzen von PERMAteach für sie? #00:50:48-6#

B: Der Hauptnutzen, ahm, ist jetzt für mich einfach, dass, dass sich das Wohlbefinden steigert, von von allen, die da in den Genuss kommen, mit PERMA in Kontakt zu sein. Also, das muss ich sagen, dass einfach, ahm, dass es den Leuten einfach gut geht, dass die sich wohlfühlen. Das würde ich mal sagen. Und und das, was mir so wichtig ist,einfach ist, dass man, dass man den jungen Leuten jetzt einfach vermitteln kann, dass sie alle, so wie sie sind, alle absolut in Ordnung einfach sind. Und das man ihnen, dass man ihnen einfach für ihre Persönlichkeitsentwicklung so einbisschen ein Rüstzeug mitgeben kann. Das, glaube ich, ist das wichtigste. #00:51:51-1#

I: Wofür sind sie dankbar? Was hat ihnen an der PERMA teach, ähm, Wofür sind sie dankbar in der PERMA teach Ausbildung? Was hat ihnen ...? Eine klassische PERMA Frage? Wofür bin ich dankbar? Sie dürfen auch drei Nennungen haben, nicht nur eine. #00:52:11-4#

B: Okay, ich bin dankbar für die für die vermehrt aufgetretenen, jetzt so freudigen Momente. Einfach, das mehr, mehr zum Lachen ist, das mehr gelacht wird, dass der Spaß irgendwo mehr geworden ist, muss ich sagen. Dafür bin ich dankbar, Ahm, dass man merkt einfach, dass die Konflikte weniger geworden sind und wenn sie jetzt auftreten und man direkt beim "Krawattl packt", dass man die schneller auch wieder los wird. Das würde ich sagen, das ist ähm. schön. Ähm, und wofür denn vielleicht noch? mmh. Ja, das könnte vielleicht auch herausgreifen: das es jetzt grad in einer Gesellschaft, wo es oft um Leistung geht und wo der Einzelne sich so wichtig vorkommt, dass man mit dem PERMA wieder das Augenmerk einbisschen auf die Gruppe und auf die Gemeinschaft und auf sein ganzes Umfeld legen kann, dass man das in den jungen Leuten wieder mehr bewusst macht. Es geht nicht immer nur um dich, sondern es geht auch nicht immer um Leistung, und ich muss mich auch nicht dauernd mit einem Anderen vergleichen, sondern ich kann mein Umfeld auch einfach sein lassen und es genießen, dass jeder seinen Platz in der Gruppe, einfach wieder mehr spürt. Das würde ich auch noch sagen. Ja! #00:53:59-6#

I: Ja, danke, vielmals. Gibt es noch etwas, was sie ergänzen möchten? Etwas, das ich nicht gefragt habe? Was unbedingt gesagt werden muss? #00:54:09-0#

B: Ich hab da ein paar kleine Notizen aber jetzt also, das wäre vielleicht jetzt einfach Da, das habe ich mir mit Großbuchstaben da vorne noch notiert. Das würde ich einfach so wichtig finden, das möchte ich auch so meinen, meinen Schützlingen so immer wieder mitgeben dieses: das JETZT. Das Jetzt. Schieb nicht dauernd Dinge vor dir her, sondern Jetzt, einfach das DA SEIN. Und wenn ich jetzt merk, das brauche ich jetzt, dann schau ich, kümmere ich mich darum, dass ichdas jetzt vielleicht bekommen kann, einfach dieses Leben im Moment. Das wäre vielleicht nur so ein Schlagwort, das ich ganz wichtig finde. Nicht später und warten wir und tun wir dann schon, sondern einfach: MACHEN und JETZT LEBEN. Ok? #00:54:57-4#

I: Super, herzlichen Dank für ihre Zeit!, Herzlichen Dank für ihre Begeisterung! Danke, dass sie uns helfen, das Programm besser zu machen, es noch breiter in die Öffentlichkeit zu bringen. Ich selber arbeite mit Perma lead. Es geht nämlich dasselbe gilt, nämlich für die Arbeit, für Führungskräfte in der Wirtschaft. Und hab leider Gottes nicht ein solches Buch um den Führungskräften zu zeigen, wie sie mit ihren Mitarbeitern umgehen sollten. Aber im Prinzip ist es genau das. Ich habe dann in Klein- und Mittelbetrieben, die Mitarbeiter dort oder die Führungskräfte. Und sie haben sie als junge Menschen. Und ich finde, das ist eine Profilaxe für alle, für all das, was später kommt. Und ich freue mich so darüber, als persönliche Anmerkung, dass es in den Schulen nun eine Form gibt, wo sie mal was ganz anderes sehen und lernen dürfen, weil wenn sie dann 30 sind und mich hören, denken sie sich, sie hören richtig. Aber ich erzähle dasselbe, was man ihnen in der Schule erzählen könnt, und ich glaube, man hat schon verhärtet, und man ist schon Leistung, und man ist schon Wettbewerb und "gutes, was gutes sehen, der hat nichts gutes"Ja, da wird es eng und wenn wir früher anfangen, umso besser, macht die Welt ein bisschen besser. #00:56:21-1#

B: Genau. Dankeschön, Frau Thurnher. #00:56:28-0#

I: Alles Liebe! Ich wünsche ihnen nicht nur ein Gutes Ende des Schuljahres, sondern auch einen erholsamen Sommer und viel Freude im neuen Schuljahr. #00:56:37-6#

B: Danke, alles Gute. Schönen Dank! Wiederschauen. #00:56:41-5#
