# Supplementary material for: PERMA.teach: a study on the effectiveness of a standardized positive education training program in Austria
Source: Front Psychol. 2025 Apr 8;16:1516572. doi: 10.3389/fpsyg.2025.1516572 (PMC12053473; doi:10.3389/fpsyg.2025.1516572)
Supplement: Supplementary file 2 [file Data_Sheet_1.ZIP › Material PERMA.teach/Material LPs/Interviews LPs Teil 4.zip!/Interviews Transkription/Interview 1 - transkribiert/Interview 1, 1.Teil transkribiert.docx]

**Interview 1. 1.Teil.m4a**

B: Sehr spannend, jawohl. #00:00:04-2#

I: Und ja, also die, das Interview geht eigentlich nur darum, darum habe ich ihnen auch im Vorfeld keine großartigen Fragen schicken wollen oder müssen, weil sie hier nicht richtig und falsch gibt, sondern wirklich wir sie gerne befragen würden, wie´s ihnen ergangen ist? Ihnen persönlich, beim Erfahren der neuen Methoden, der Interventionen, der Möglichkeiten? Wie gut oder wie leicht es war, es umzusetzen in ihrem Alltag in der Schule, mit ihren Kollegen ? Und wie es ihnen denn dabei gegangen ist? #00:00:39-1#

B: Ok. #00:00:40-4#

I: Das wäre jetzt eigentlich schon der, die Einleitung. Vielleicht ganz kurz zu ihnen noch, dass wir, dass wir das auch statistisch auf der Spur haben: Wie lange sind sie bereits Lehrkraft? #00:00:52-9#

B: Ich bin schon seit über 30 Jahren im Dienst? #00:00:55-8#

I: Also, sie haben viel Erfahrung, Wahrscheinlich, auch schon viel, ausprobiert. sehr schön. Und in ihrer aktuellen Schule, ich habe gelesen, das ist die PTS 20 #00:01:06-6#

B: PTS 20. Ja. Und ich bin aber derzeit mit einem kleinen Team am Campus Christine Nöstlinger. Wir sind eine Expositur von der PTS 20. Wir gehören aber zur PTS 20. #00:01:17-9#

I: Okay, und das ergibt das kleine Team und betreut dort äh #00:01:22-3#

B: zwei Klassen betreuen wir. Wir sind drei Kern-Lehrerinnen in dem Team. Es gibt noch zwei andere, die dabei sind, aber das ist, und eigentlich ist unsere von von, von der Grundeinstellung, das sind zwei junge Kolleginnen, sind Anfang 30, da bin ich schon quasi ein alter Hase dazu. Und irgendwie, ich glaube, das, was PERMA teach gibt, oder die positive Psychologie, ist auch von Grund auf unsere Lebenseinstellung. #00:01:47-4#

I: Genau, das haben sie in dem Fall nicht neu als Haltung kennengelernt, sondern war schon da. #00:01:53-4#

B: So nicht. Aber mit den vielen Übungen, und die bin ja neugierig für das Ganze und offen, und haben wir dann sehr gefreut, also das vielleicht zu Einleitung, wie ich bei meinen vielen Fortbildungen, die ich besuche, über Wings, über das Wiener Netzwerk gesundheitsfördernder Schulen Ingrid und Eva kennengelernt. Und Ingrid ist ja für auch ältere Lehrerinnen vor allem ein bekanntes ja! #00:02:18-7#

I: Ein Urgestein? #00:02:18-7#

B: Ja. Ja. #00:02:19-4#

I: Aha, aha! #00:02:20-7#

B: Ja, in meinem Alter kennt man sie auch. #00:02:23-3#

I: Aha, sehr schön! #00:02:24-7#

B: Und das war da natürlich naheliegend, weil ich gehe zu diesen gewissen Veranstaltungen auch nur noch, um schauen, was gibt es neues, wo kann man sich vernetzen und Ingrid zu treffen und dann auch die Eva kennenzulernen, die ja Sprühen für das. Ja. Da bin ich natürlich sofort aufgesprungen. #00:02:41-8#

I: Schön, wie schön, dass dieser Kontakt entstanden ist. Also sie haben da in ihrer, ihrer eigenen Geschichte, also Klausur haben sie es genannt? Positur? Wie viele Klassen sind das? #00:02:56-2#

B: In dieser, in der Expositur? #00:02:58-9#

I: Expositur, war der Ausdruck. #00:03:00-0#

B: In der Expositur sind wir zwei Klassen. #00:03:02-6#

I: Mhm, Okay. #00:03:03-7#

B: Mhm, ja. . #00:03:06-0#

I: Und da haben sie #00:03:06-7#

B: Na, na, in dem Haupthaus, in der Stromstraße 44 gibt es auch noch andere Kollegen, die, die auch aufgesprungen sind, und da sind wir jetzt zum Beispiel, kann jetzt gleich einhaken bei einem Punkt, bei der Schwierigkeit, des Umsetzens. Anfänglich waren ja alles sehr schwer begeistert, und die Schwierigkeit hier war zum Beispiel also im Laufe des Jahres zu sehen: Ähm, die Überforderungen der Lehrerinnen und Lehrer. Ähm, das dann auch wirklich konsequent umzusetzen oder einzusetzen. #00:03:41-0#

I: Mhm. mhm #00:03:42-6#

B: Bei ein paar hat es ganz gut geklappt, die aber eh auch vom Grundgedanken her, die ähnliche Philosophie haben, oder die Einstellung, Kinder positiv zu bestärken oder dort anzusetzen, was sie können und nicht mehr was sie nicht können? Da gab es dann halt auch dann bei den Lehrerinnen. Es kommt natürlich auch immer aufs Team an und auch auf die, das Rundherum, das Umfeld. Da habe ich für mich als Initiatorin fürs Poly gemerkt, wui, ja es, es ist fein, wenn man für was brennt, aber man muss selber dafür brennen und es an andere weiterzugeben. Das ist oft die Schwierigkeit. Sind zuerst zwar Feuer und Flamme. Ingrid und Eva waren auch bei uns an der Schule, also im Stammhaus Stromstraße, haben sich ganz viele Kollegen gemeldet. Im Herbst war dann der Fakt schon, oder die Tatsache schon wieder anders. "Ich weiß nicht, wann ich das soll?" und "ich habe so viel um die Ohren!" Eh immer das gleiche. #00:04:35-4#

I: Klar, die zeitliche Komponente. Kommt da immer dazwischen. #00:04:41-2#

B: Es war trotzdem, glaube ich, für einige und vor allem für die Schüler, weil wir haben ja dann auch mitgemacht bei diesen "Mutmach-Videos". #00:04:48-4#

I: Mutmach? Mhm. #00:04:48-7#

B: Die Mutmacherei. #00:04:52-3#

I: Mhm. Genau #00:04:54-2#

B: Auch ein Teil von PERMA teach, oder zumindst wurde ein, ich glaub, das ist die Ira Mollay oder wie sie heißt. Da haben wir auch gemerkt, wa die Kinder, so auf Youtube und so, oh, das ist ein bisschen schwierig, haben aber dann doch statt fünf immerhin acht Schülerin davon überzeugen können im Internet. #00:05:10-0#

I: Und das hat, das war eine schwierige Aufgabe, vom Gefühl her, sie zu überzeugen, oder da irgendwie sie zu motivieren? #00:05:16-3#

B: Also in unserem, jetzt bleibe ich bei unserem Team an der, am Campus war´s nicht schwierig, da waren sie so: "Frau Enzi, ein Interview und dann auf Youtube. Na, so." und dann hab ich gesagt: "Du bist so gut. Du hast so tolle Sichtweisen und so. Machs halt für mich quasi." Und das ist da zum Teil der persönliche Erfolg #00:05:37-8#

I: Klar, der persön, die persönliche Beziehung hat natürlich schon auch beigetragen, oder? Das Vertrauen. #00:05:42-4#

B: Das ist dann für uns war es eigentlich ganz toll und die Videos zu sehen, was die Kinder gesagt, und das Feedback, und das war dann eigentlich eine tolle Geschichte. #00:05:52-0#

I: Schöne. das ist auch. Das spiegelt ihn dann auch wieder schön zurück, dass es eigentlich angekommen ist. #00:05:56-7#

B: Ja ist angekommen. Ja. #00:05:57-9#

I: Das ist auch für uns jetzt schön zu hören, weil es ist immer die Frage, wem bringt es Wohlbefinden? Wohlbefinden in der Schule ist natürlich ein riesengroßer Bereich, und da ist eben jetzt die Frage für uns: Wie konnten sie es umsetzen? Und wie haben sie gespürt, dass die Kollegen miteinander, im Gespräch, in der Art und Weise, eine Verbesserung oder eine Veränderung passiert ist? #00:06:25-8#

B: Ich kann jetzt nicht wirklich sagen, ob es eine spürbare oder sichtbare, beziehungsweise was man so eine so messbare Veränderung ist. Aber wenn wir das dann angesprochen haben mit den Schülern und dann auch bei den Interviews, war es eigentlich die Übungen, die vorwiegend meine Kollegin, die Frau Rautner, gemacht hat, also ich auch, aber sie noch mehr haben dann schon durch, ja auch das viel mit der Dankbarkeit oder die tägliche Zufriedenheit. Oder wie kann ich, wenn es mir schlecht geht, das ändern, dass es mir besser geht, oder wie kann ich jemanden helfen? Das kam definitiv durch. #00:07:05-0#

I: Okay, und das haben sie jetzt auch formuliert. #00:07:08-4#

B: Ja auch formuliert, weil wir haben dann auch gesagt, natürlich gibt es in der Schule, und das darf man auch nicht untern Tisch kehren, es kommt zu Schwierigkeiten, es kommt zu Spannungen bei den Kindern, es gibt schlechte oder gute Tage auch bei uns, Lehrer natürlich, #00:07:24-5#

I: Klar. Man ist ja auch nur Mensch. #00:07:28-1#

B: und denke mal doch dran, die eine Übung, die Achtsamkeitsübung, halten wir kurz inne, schnaufen wir tief durch, zählen wir bis zehn, ich mein das in der Form habe ich schon vorher gemacht, ich selber! #00:07:40-0#

I: Ja, als Potenzialentfaltung für sich selber. #00:07:43-9#

B: Ja, also, man muss sich dann selber auch bei der Nase nehmen. Manchmal, wenn man halt schlecht drauf ist und so okay, Entschuldigung, ist es jetzt gemeint, ich geh zurück, wir sind jetzt achtsam, bisschen nett zueinander,mit dem Bewusstsein das PERMA teach einzusetzen. Also, das war meine persönliche Veränderung, wenn man sich doch bewusst mit dem auseinan, BEWUSSTER. #00:08:02-3#

I: Weil es einen Namen hat, und weil es ein Konzept ist, sozusagen dann "ah ja, genau das setzt man jetzt da ein. #00:08:10-6#

B: Und genau natürlich mit den Kindern sozusagen: "Kinder, da gibt es eine wissenschaftliche Psychologie. Das ist erwiesen, dass, wenn man sich mit positiven Dingen umgibt oder wenn es einem schlechter geht, wenn man sich einmal, wenn man nur lächelt, da fallt mir die Birkenbihl ein, Methode, dann geht es einem besser, dann dann schaltet im Hirn was um. Und das ist schon angekommen bei den Kindern. #00:08:33-9#

I: Und sie glauben, Weil es auch, weil es natürlich schöner oder glaubwürdiger ist, da mal eine Studie auch zitieren zu können, oder? #00:08:38-9#

B: Ja, das ist natürlich #00:08:40-1#

I: und nicht nur die Lebenserfahrung, oder ihre keine Ahnung. #00:08:43-2#

B: Genau, genau und wenn man auch sagen kann, "Passts auf, da gibt es jemand" da hab ich das Büchlein, habe ich direkt hier vorbereitet, und arbeite noch mit dem, und das ist erforscht. Das ist ja eine Tatsache. Wir müssen bei alle, ich, du, wir alle gemeinsam daran arbeiten, und so kommen wir leichter durchs Leben, auch in schwierigen Situationen, und das ist schon angekommen und auch im Team. Also jetzt im Kernteam, wir sind noch der Einstellung so, wenn manchmal hat man halt so seine Tage also okay, "schnaufen wir einmal kurz durch!" #00:09:17-9#

I: Okay, ich meine, das ist immer die Frage. Wann haben sie begonnen, diese Erkenntnisse umzusetzen? Ich meine, sie haben, sie haben mir das eh schon erzählt. Sie machen das schon länger. Aber wenn sie jetzt, das ist jetzt ne konkrete Frage, wenn sie jemandem erklären würden, also aus dieser Fortbildung jetzt heraus, einem Außenstehenden, Was ist der Unterschied zwischen Regelschule und diesem positiven Bildungsansatz, dieses PERMA teach Ansatz? Was wäre der Unterschied, würden sie jemandem erklären? #00:09:53-4#

B: Ich würde erklären, dass es in Zeiten bewusst, in diesen auch schwierigen Zeiten, aktuell, weil es gibt immer schwierige Zeiten, setzt diese Methode ein. Versuche es mit diesem Büchlein, das es bereits gibt, mit tollen, mit tollen, bereits fix und fertigen, quasi Unterrichtsvorbereitungen daran zu arbeiten. Lass dich drauf ein, und du wirst eine Veränderung sehen im Laufe des Schuljahres oder schon auch im Laufe von Monaten. Probier es, steigt ein und nimm dir Zeit dafür! Wir haben die Gegenstände jetzt in unserem Fachbereich, in unserem Bereich polytechnische Schule. Es gibt individuelle kreative Förderungen und individuelle soziale Berufsorientierung, so in etwa heißt das,, was ja auch das anspricht, das Team Teaching, das Team Miteinander arbeiten? Probier, die Kinder darauf aufmerksam zu machen, und du wirst sehen, es geht auch dir besser in schwierigen Zeiten. #00:10:57-6#

I: Okay, also, diese Wechselwirkung auch, das ist nicht nur an, sondern. #00:11:02-0#

B: Nicht nur für die Kinder, sondern auch für dich selbst. So würde ich, oder so erzähle ich es auch Kollegen, oder wenn ich jetzt wieder woanders bin: "Habt´s ihr schon gehört, das gibts!" "Versuch, ist, weil es macht einen Unterschied mit dir. #00:11:16-8#

I: Mhm. #00:11:17-4#

B: Mit dem Unterricht und auch für das Gegenüber, in dem Fall unserem Klientel, die Schüler. #00:11:23-0#

I: Mhm, mhm! Okay. Super! #00:11:24-0#

B: So würde ich es weiter transportieren oder so werbe ich es auch an. Sagen wir einmal so. #00:11:28-9#

I: Mhm, weil das ist ja, das führt jetzt zur nächst, zur nächsten für uns wichtigen Frage, Also, diese Erwartungen, bevor sie jetzt in diese, in diese Fortbildung eingetaucht sind. Sie waren da natürlich ein bisschen positiv, sagen wir vorbelastet, weil sie die Ingrid Teufl mit großer Freude wieder gesehen haben und gekannt haben. Aber also, wenn sie jetzt die Erwartung nehmen, bei Beginn der Fortbildung und jetzt dieses Gefühl, jetzt, wo es fertig ist. Sind die Erwartungen erfüllt worden? Hätte es #00:12:01-1#

B: Das ist jetzt eine gute Frage, da muss ich kurz. Ich glaube, ahm alle meine Erwartungen sind nicht erfüllt worden. Aber das bezüglich auf das Team in der Schule, das war, zum Teil die Schwierigkeiten zu erkennen. Aber das ist nicht das erste Mal, das mir sowas passiert. Aber das macht nichts. Wenn ich zwei bis drei erreiche im Team, im Erwachsenen-Team, finde ich das schon als Erfolg. #00:12:24-5#

I: Okay. Okay. #00:12:26-2#

I: Ich äh der Ansteckung, Ansteckungs-Moment hat, war vielleicht einbisschen enttäuschend? So zu sagen. #00:12:33-0#

B: Ja, weil, das war auch, wir waren ja getrennt in den Häusern, und die Enttäusch. Meine Erwartung war halt, weil es viele junge Kollegen waren, das die doch mehr sich mit dem Auseinandersetzen oder mehr drauf Einsteigen, oder. Das war meine Erwartung persönlich an das Team. #00:12:54-0#

I: Mhm. #00:12:54-4#

B: Aber das positive an dem Ganzen war dann doch zu sehen und, mir ist schon klar, dass ich eben nicht alle erreichen kann. Ich bin so, so jemand, für alle der "Wa, das ist so was tolles!" #00:13:06-5#

I: Schneller, schneller, besser! #00:13:09-9#

B: Ja, leider. Aber das war zum Beispiel, wo ich sage, okay, das müsste ich jetzt vielleicht anders herangehen. Ich habe aber auch. #00:13:17-9#

I: Darf ich sie fragen, wie würden sie das anders angehen, wenn sie jetzt das noch mal machen müssten? #00:13:24-0#

B: Ähm, ich glaube, ich würde es weiterhin einfach so verbreiten und habe auch zufällig in diesem, in dieser Expositur, wo ja auch eine Mittelschule ist, und ich habe viele Mittelschullehrer um mich herum. Da kam eine zum Beispiel von einer Fortbildung und sagt, bringt das ganze Paket von PERMA teach mit. "Ma, ich möchte euch etwas zeigen!" sag ich: "Ja super. Das kenn ich schon, wir arbeiten damit, wir haben das im Programm". Und so im Gespräch bin ich darauf gekommen, ist es am einfachsten, wenn Leute kommen oder Kolleginnen kommen, die sagen, "da ist, was toll." Da sag ich: "Versuche es einmal im Monat, einen Tag." #00:14:01-6#

I: Mhm. #00:14:02-5#

B: Also, ich habe die Erwartung insofern runtergesetzt. Ich empfehle jetzt, mach einmal im Monat einen Tag, wo du PERMA teach machst, wo du positive Psychologie bewusst einsetzt, nicht in dem, wie wir machen. Wir machen es ja wöchentlich. #00:14:19-6#

I: Mhm, mhm, ja gut, da muss man wirklich auch reinwachsen, oder, wie sie sagen. #00:14:24-0#

B: Ich glaube, da muss man reinwachsen. #00:14:25-4#

B: Ja, so habe ich das jetzt für mich runtergebrochen, also das jetzt für mich runtergeht. Also, meine Art war "Okay, Gertraud! geh´s kleiner an!" #00:14:31-5#

I: Mhm Okay, ja, aber ist ja auch ein gutes Learning, nicht gleich das Kind mit dem Bade dann auszuschütten, sondern das gute einfach langsam wachsen zu lassen. #00:14:43-5#

B: Genau ja, das ist jetzt von meiner Erfahrung. Meine Erwartung war zu hoch. #00:14:48-0#

I: Mhm. #00:14:48-9#

B: Aber weil auch so viele Kolleginnen aufgesprungen sind und dann einfach eigentlich wieder abgespru, also nicht ganz abgesprungen, aber nicht in der Intensität, wie wir ich. Aber ich habe jetzt für mich auch gelernt, "Okay, Gertraud, das haben wir auch schon durch," Das kenne ich schon von anderen Sachen. Es wird wachsen. #00:15:07-4#

I: Ja, genau. Sie würden, wenn ich das richtig höre, die Fortbildung anderen empfehlen? Also nicht jetzt, also Ja. Empfehlen. Als Inspiration. Wäre, würden sie das aus ihre Überzeugung heraus? #00:15:22-5#

B: Ja, und ich würd´s auch wenn ich jetzt sagen würde "Ihr müsst ja keine Fortbildung machen, nehmt jetzt mal so ein paar Übungen raus, und wie gesagt, machts einmal im Monat, probiert es einmal aus! #00:15:33-2#

I: Mhm, Mhm, Okay! Also das heißt. Was hat ihnen denn besonders gut gefallen an der PERMAteach Ausbildung? #00:15:41-3#

B: Die Ausbildung, die Ausbildung selbst, also unsere Seminare, unsere Fortbildungen für uns Lehrerinnen, war das Zusammenkommen oder auch das Untereinander, der Austausch. #00:15:57-7#

I: Ähm, das Vernetzten der eigenen Kollegen. #00:16:01-5#

B: Ja, genau das Vernetzten der eigenen Kollegen und das Treffen, und einmal hatten wir wirklich ein gutes Miteinander. Da waren mehrere Kollegen bei einem, von dem ich glaube, das war Stärke-Cafe, oder war das, ich weiß jetzt gar nicht mal, und da waren dann wirklich, es war ein hardcore Geschichte. Wir hatten vorher Konferenz, und im Anschluss gab es dann das Zoom-Meeting. Also da ging´s halt, und das hängt aber jetzt wirklich von der Leitung ab. Wenn es nicht ermöglicht wird, dass an dem Tag keine Konferenz ist, sondern macht es einfach im Anschluss, weil die Wichtigkeit nicht so gegeben ist, da ist es, da ist der Haken. #00:16:35-5#

I: Mhm. Beim Setting, das Setting einfach. #00:16:39-2#

B: Wenn die Leitung nicht dahinter steht, gleich, und die Schwierigkeiten gibt es zum Beispiel Kollegen vormittags Unterrichten, eine kurze Pause haben, dann drei Stunden Konferenz haben und dann noch zwei Stunden PERMAteach Fortbildung. Da geht schon jeder am Zahnfleisch. #00:16:58-3#

I: Ja . ja, #00:17:00-3#

B: Es ist aber nicht in jeder Schule so. #00:17:00-3#

I: Mhm. mhm. okay. #00:17:01-4#

B: Aber die, mir hat es halt, also bei der letzten Fortbildung, da hab ich einfach, ich war zuhause, ich habe es einfach vergessen, weil ich es nicht aufgeschrieben habe, und es ist mir einfach passiert, ist passiert, aber ich für mich selber. Also, ich freue mich, als nächstes Jahr bin ich auf Sabbatical, aber ich finde, ich freue mich also. Meine Kollegin zum Beispiel, die das sehr intensiv wirklich gemacht hat, in beiden Klassen hat mich schon "Du, kannst du mir diese Talent-Loop Lizenzen verlängern?" sag ich "ja, gerne" sag ich "und die Bücher bestellen fürs nächste Jahr?" "sehr gerne" Ich hab´s nicht angepriesen, jetzt gewartet, bis sie an mich herantritt, und also eine ist schon die jetzt unmittelbar in meinem Umfeld ist schon an mich herangetreten, und im Haupthaus habe ich es an eine Kollegin mit abgegeben, sie dort quasi vor Ort die Ansprechpartnerin ist. Ich bin natürlich erreichbar. #00:17:58-4#

I: Mhm, aber dann ist es Übergeben, ist offensichtlich. #00:18:02-5#

B: Aber ich glaube, bei den Fortbildungen, was mir sehr gut gefallen hat, war eben das eine, speziell, wo wir Schulen eine kurze Präsentation machen sollten, gemacht haben, wer was gemacht hat und wie PERMA in den Schulen umgesetzt wird, und das hat mir sehr gut gefallen und mir sehr viel gebracht zu sehen, wie toll die anderen das wirklich im Schulhaus umgesetzt haben, also sichtbar gemacht haben. #00:18:27-9#

I: Okay also, also nach außen hin sogar sichtbar. #00:18:31-3#

B: Nach außen sichtbar, das ist, was was mir heuer oder uns im Team nicht so gelungen ist. Aber wir sind auch gesiedelt, wir haben ein neues neues Haus, wir haben alles active, also für uns war sehr vieles neu. #00:18:44-4#

I: Aber das heißt, dieser Austausch hat wohlgetan, wenn man einfach auch neue Ideen bekommen hat, das man vielleicht sogar selber nächstes Jahr oder dann in der nächsten Zeit Dinge tun könnte? #00:18:54-5#

B: In der nächsten Zeit. Genau. Also, das war jetzt für mich persönlich von der gesamten Fortbildung eins der Highlights. #00:19:05-9#

I: Dieser Austausch? #00:19:06-5#

B: Dieser Austausch, ja. Zu hören, was tun die anderen, und ich glaube, so könnte das Schneeballsystem dann auch funktioniert. #00:19:13-1#

I: Mhm, Mhm, eben also immer. Ich gehe jetzt davon aus. Also der fachliche Input, der war neu, also teilweise neu? Haben sie da das Gefühl gehabt, sie haben, waren gut unterstützt mit Workbook oder mit, ich glaube, es gab was zum Ausdrucken? #00:19:31-0#

B: Ja. Also, wir waren ja, wir waren hervorragend unterstützt. Wir kriegen jetzt auch wieder die PERMA Post. Großartig! Das kannst du wirklich! Das kriegst du in der Früh, oder in der Nacht, und das hast du entweder am Bildschirm und du druckst es aus, und du hast eine Stunde. Du kannst quasi das Durchlesen was Sache ist und du machst das. Das ist super! Und die Kinder? Also, ich habe das jetzt gerade einmal wieder mal ausprobiert. Die Kinder springen drauf auf, sag ich "Wir machen jetzt einmal eine Viertestunde kein Mathe. Wir machen, jetzt das." Das is, "Das probieren wir jetzt aus, und ihr müsst uns das Feedback geben, wie das ist". Wir fragen unsere Kinder, "wie seht ihr das?" "Was ist das positive? Was ist das schlechte?" Oder? Also, wir holen uns sehr viel von den kids, generell. #00:20:16-1#

I: Also gemeinsames Lernen, eigentlich? Oder? #00:20:18-3#

B: Gemeinsames Lernen: was sollen wir ändern oder was auch immer, und ja, und unser Spiegel oder unser Feedb, unser Lernen sind auch die Kinder. Aber es ist großartig zu haben, diese bereits ausgearbeiteten Materialien, die zur Verfügung gestellt bekommen, also jetzt in Papierform oder digital, eben diese PERMA Post, die wir da kriegen. Es ist. Es macht eigentlich, es ist eine große Erleichterung. #00:20:47-2#

I: Okay, super! #00:20:48-4#

B: Und die Materialen sind sehr gut durchdacht, weil es gibt ja eben alles für die Kleinen, es gibt das für die Großen. #00:20:53-9#

I: Mhm. mhm. #00:20:54-4#

B: Es ist eine große Hilfe. #00:20:56-4#

I: Ja. Und es ist aber nicht zu viel, sondern ist wirklich so, dass man, dass sie als Lehrer selber gestalten können, was sie rausnehmen, was zu ihnen passt, welche Interventionen auch immer in ihren Unterricht reinpasst? #00:21:07-1#

B: Genau, genau, und es war anfänglich vielleicht, das ist auch ein Punkt, ganz interessant, das habe ich damals auch Eva und Ingrid eh, so haben wir´s eben eh mitgeteilt, anfänglich, wenn die Kinder so waren "äh, jetzt nicht schon wieder das Buch" aber das sind Kinder generell. Also bei den Jugendlichen in der Pubertät, ist ja das, über Gefühle sprechen, über sich ist ja nicht einfach, manchmal. #00:21:29-7#

I: Und das haben sie an dem Buch natürlich festgemacht, oder? Kaum hat man das Buch in der Hand? #00:21:32-9#

B: Ja an dem Buch, "ja na, haben´s gesagt" das Buch, dann hab ich gesagt "was fehlt was fehlt denn an dem Buch?" "Was ist denn da so schlimm?" "Ja, dann müssen wir immer" da sag ich "na ja, dann machst du die eine Übung, und wenn sie dir nicht gefällt, dann machst du halt die nächste." Es soll ja Spaß machen, oder "du tauscht dich aus und redest halt einmal mit deiner Nachbarin." Jo das. Wir müssen das jetzt ja nicht so strikt anwenden! #00:21:52-7#

I: Genau. man muss ja nicht. Man muss ja nicht. #00:21:55-1#

B: Kindern muss ja jetzt auch erst erklären, dass es das gibt. Es gibt eine Freiheit. #00:21:58-5#

I: Ja, es ist halt eine neue, wirklich eine neue Haltung, und dementsprechend muss man auch den Kindern erklären, "man darf auch nicht". Nicht geht auch. #00:22:06-9#

B: Genau. Man darf auch nicht. Genau, Und das war eigentlich schon sehr, also für uns eine große Erleichterung auch, das weiß ich von den Kolleginnen, mit den vorhandenen Materialien. #00:22:18-9#

I: Super, okay, das nehmen wir, das nehmen wir mal ganz gerne mit! #00:22:21-4#

B: Das ist ganz ein wichtiger Punkt bei dem Ganzen, glaube ich. #00:22:24-8#

I: Also, das da wirklich schon so gut vorgearbeitet wurde! #00:22:27-0#

B: Ja, und das weiß die Ingrid auch ganz sicher bestimmt, auch die Eva sicher, aber, weil es einfach, weil die Ingrid hat ja da lang dran und wirklich in allen drum und dann. Und es ist fein, wenn du was hernimmst und sagst "okay, ich kann jetzt weiterarbeiten", und ich muss nicht mehr Gedanken machen, zu was weiß ich was. #00:22:44-5#

I: Das passt ja auch einbisschen in den Lehreralltag auch, also es erleichtert den Lehreralltag oder ? Man hat keine Zeit und neue Sachen fressen eh schon Zeit, und dann ist es natürlich, wahrscheinlich sehr erleichternd, wenn man das wirklich eins zu eins verwenden kann. #00:22:58-1#

B: Ja, und wie haben hier bei uns, Oder weiter? #00:23:01-9#

I: Ich wollte in diesem Zusammenhang nur noch, es gibt da ja auch Apps. Es gab ja auch. Es gab ja auch einige technische Apps, die angeboten wurden. Werden, die genutzt? Werden, die von ihnen, oder den Kindern? #00:23:14-9#

B: Ja sicher. Das Talent. Talent-Loop. Wir haben das Talent-Loop #00:23:16-3#

I: Okay, okay, genau. #00:23:18-7#

B: Und wie gesagt, dass wie vorher schon erwähnt, die Kollegin, Frau R., die hat mich, sie ist jetzt eben an mich heran und hat gesagt "Du, Getraud, weil du bist nächstes Jahr nicht, kannst du mir nicht die Lizenzen geben?" weil da sagt sie, "das ist so großartig" Und die Kinder, wir haben das auch gemerkt, dass war vielleicht, noch einmal die Frage vorhin, ähm die Veränderungen und die Auswirkungen im Laufe des Schuljahres. Wir machen jetzt Präsentationen, oder wir machen ein neues Thema, und wir holen immerFeedback ein von den Kindern, und dann war ein junger Kollege oder ein Kollege bei uns und hat das Feedback eingeholt. Und er hat gesagt: "Bist du deppert. Was haben die für eine Feedback-Kultur?" #00:23:58-4#

I: Aha, okay, Also hat sich das sichtbar. #00:24:01-4#

B: Hier, ja ganz. Wir haben wirklich, also, wir machen das wirklich mit plus /minus, Augenkontakt oder was auch immer. Wir haben so Punkte auf, und sie müssen dann wirklich nicht nur sagen, dass ist super, das ist schlecht, sondern wirklich ganz konkret sagen, was war gut, war die Sprache gut, war´s gut erklärt, wirklich bewusst Zuzuhören! #00:24:19-4#

I: Schön, dass man das Feedback von einem außenstehenden Kollegen, weil selber merkt man ja gar nicht, dass das schon eine verbesserte Kultur ist, weil man ja mit mitwachst und mitlebt. #00:24:29-2#

B: Ja, und das haben wir jetzt dann auch bewusst aufgenommen in unserem kleinen Team, natürlich auch einbisschen durch PERMA teach. #00:24:36-4#

I: Mhm. #00:24:37-1#

B: Wir haben gesagt, wir müssen, wenn wir das wirklich einbringen, sollen, wir müssen einfach, wir müssen selber überlegen, dass man für uns auch "Was lernen wir draus?" Wir. #00:24:46-0#

I: Genau. Als Lehrer. #00:24:47-0#

B: Als Lehrer, und was geben wir den Kindern, und was sollen die Kinder draus lernen? Und da war eben das Feedback, von einem, der sonst nicht bei uns in der Klasse ist "Super. was habts ihr da für Kinder?" Und wirklich. Auch die ruhigsten Kinder haben aufgezeigt und wollten Feedback geben. #00:25:02-6#

I: Aha. Ja und schön, dass man das kultiviert und lernt und auch die Vokabeln dazu lernt, zu finden. Gut und schlecht, oder? Das haben wir. Aber wenn man weiterdenken darf und sollte und angeregt wird, dann kann man eben auch die Stärken, dann, ich finde, man muss ja das auch trainieren, ähm. Die Stärken-Brille ist ja nicht so leicht, wenn einem die Vokabeln dazu fehlen? #00:25:28-2#

B: Ja. Und das mit diesen Stärken, das haben wir wirklich durch dieses Talent-Loop #00:25:32-6#

I: Mhm. #00:25:33-4#

B: Und wir haben ja dann auch so Bewerbungsgesprächs-Training, und das machen wir mit den Schülern, und wir haben das im Vorfeld Stärken/Schwächen, was auch immer alles da drin war, wirklich bewusst. Die Kinder mussten erst einmal herausfinden, was sie können. #00:25:48-2#

I: Ja genau. #00:25:49-4#

B: Man muss ja selber. Das ist ja. #00:25:51-2#

I: Das weiß man ja, weiß man ja oft gar nicht, oder? #00:25:53-8#

B: Wie? Wie? Wie heißt das Wort dazu? Zum Beispiel. #00:25:59-3#

I: Genaus. genaus. Also das ist wirklich ein Vokabeltraining. #00:25:59-3#

B: Ja, und das ist wirklich gewachsen. #00:26:02-1#

I: Mhm. Mhm. Okay. Sehr schön! #00:26:04-2#

B: Das ist definitiv, definitiv das PERMA teach. Das bewusste, der bewusste Einsatz. Talent-Loop, PERMA teach, das Gesamtpaket, was da mitkam. #00:26:11-8#

I: Mhm, mhm, das ist toll! Sie haben eigentlich, ich schau da grad meine meine Fragen zum Feedback. Ich glaube, die einzige Feedback-Frage, die ich jetzt einfach noch brauche, ist. So die Rahmenbedingungen, das Eingeladen werden, das Terminisieren, das Zoom-Room. MMMh. Hat das alles gut geklappt? Sind sie da zufrieden? Hätte es da Verbesserungsmöglichkeiten gegeben ? Oder weniger ist auch manchmal mehr? #00:26:38-7#

B: Also, da muss ich kurz nachdenken, was da alles war. Also die Ahm. #00:26:47-5#

I: dass Eingeladen werden, das Kontaktiert werden? Die Termine #00:26:47-6#

B: Also vom technischen, von dem ganzen Termine, die gab es ja schon wirklich ganz lang voraus. Also das war, perfekt. #00:26:58-2#

B: Es war planbar, und es war von Ingrid und Eva hervorragend, bis ins Detail. Ich sage, den letzten habe ich selber verschwitzt, wurscht, passiert, das finde ich großartig. Was jetzt für mich das negative ist, ist nicht Seiten von PERMA teach, sondern, das hab ich vorher schon erwähnt, das Seiten von Organisation im Team. #00:27:23-0#

I: Mhm, Okay. #00:27:24-9#

B: Im Team an der Schule. Also mein Team, natürlich da bin ich ja vor Ort. Das ist ganz anders. Da sag ich "Bitte nehmt´s euch die Stunde Zeit? Setzt euch dazu, sag ich " das letzte Mal war ich alleine. Das ist mir peinlich." Außerdem ist es gut, wenn, haben sich die Ladies das verdient. #00:27:42-3#

I: Ja. #00:27:43-4#

B: Aber nicht nur von mir, und ahm da ist es aber was abhängig von den Rahmenbedingungen. Diese Termine, die wir bereits vorgegeben ist, manchmal kollidiert mit Konferenzen, mit ahm anderen, für manche, ahm dann eben bei uns im Haupthaus, war einfach wirklich da, dass die Leitung zwar alles super findet. "Mach nur, Mach nur," aber eigentlich das nicht wirklich mit Herz unterstützt, zum Beispiel bewusst diese Konferenz dort hinsetzt, obwohl wir den Termin bereits ein Jahr haben. Eine Konferenz, die ein Tag drauf oder zwei, also da hackt es, und da ist es so, wie ich sage, da steht und fällt mit der Leitung. #00:28:35-7#

I: Mhm. #00:28:35-9#

B: Gar nicht, dass das bewusst und bösartig und absichtlich war, und wenn ich das dann hinterher erklärt, sage ich "du, das war jetzt" #00:28:43-1#

I: Die Priorisierung, die Priorisierung oder die Wahrnehmung, die Wertschätzung ist nicht. Das ist schwierig. #00:28:49-1#

B: Also, das war das, wo ich sage, das war nicht in Balance, manchmal. #00:28:53-5#

I: Jetzt, weil die gerade übersehen habe, die die, die sie haben, jetzt gerade die, die die Bücher und die Kinder und die jugendlichen Ausführungen gelobt, die Padlets, als dieses digitale zur Verfügung stellen, war das auch hilfreich? Haben sie das nützen können? #00:29:10-0#

B: Das habe ich, ich zum Beispiel, ich persönlich wenig genutzt, nur weil ich einfach eh schon so viel Material gehabt habe. #00:29:15-9#

I: Ok. Also. #00:29:18-0#

B: Ich war, ich selber, war. Ich zum Beispiel muss, da bei mir steht das auf meiner Liste, weil wir haben das so ein bisschen Protokoll geführt für uns. Muss ich mich auch wieder ein bisschen ein. Auf das habe ich nicht so viel Rücksicht genommen, zum Beispiel. Aber es war hilfreich, weil es war da. #00:29:36-5#

I: Ja, okay, und nicht. Man kann ja jederzeit darauf zu. #00:29:40-6#

B: Von jederzeit zugreifen, genau also, das ist schon eine tolle Sache, nur eben durch das, dass ich nicht wirklich aus dem digitalen Zeitalter komme. #00:29:47-9#

I: Da ist man analoger unterwegs, symphatischer! #00:29:52-4#

B: Oder dann, dass diese tägliche oder diese wöchentliche PERMA Post, die da kommt, das war dann leichter für mich. Das ist mir dann gar nicht eingefallen. Aber ich weiß von einer Kollegin aus dem anderen Haus, die eben auch mehr im IT-Bereich ist, die hat das sehr wohl genutzt. #00:30:10-5#

I: Mhm. Okay. Das ist dann in ganz anderer Zugang zur Materie. Regelmäßig. Sie haben vorher kurz erzählt von ihrem, ihrem Umsetzen von PERMA oder äh den Interventionen im Unterricht. Wie genau ist es in den gelungenen es umzusetzen? Also ist es jetzt sozusagen einmal in der Woche eine Stunde, oder ist es am Morgen zehn Minuten? Oder geht es auch in die Richtung, dass man sagt, man macht mathematische, neue Aufgaben oder in Deutsch irgendwie eine andere Literaturanalyse? #00:30:47-4#

B: Ja, wir haben es sehr zielbewusst. Wir haben eben einmal in der Woche, das nennt sich eine Stunde, und da wird PERMA teach gemacht, definitiv. Meistens war das eine. Die Kollegin hat das mit Talent-Loop gemacht. Also, meistens. hat das die eine Kollegin gemacht. Diese Stunde in jeder Klasse ist einmal in der Woche, und da wird nichts anderes gemacht. Es wird keine Klassenvorstand-Stunde gemacht, es wird kein was weiß ich gemacht. Diese Stunde ist für mich, und das wurde so fast, ich würde sagen zu 99% auch durchgeführt. Also wirklich bewusst, und wenn wir dann, ich kann mich erinnern, wir hatten einen Weihnachtsmarkt-Vorbereitung, an dem Tag hatten wir wirklich nur Weihnachtsmarkt vorbereitet. Aber dann haben wir gebastelt und so, und da haben wir während der, dessen Vorbereitungen, haben halt auch über das gesprochen. Bei den Kindern natürlich, "wie siehst du das?" "Jetzt haben wir das nicht gemacht." "Wie war ist das letzte Mal?" Da haben wir das halt so eingebaut. #00:31:44-3#

I: Mhm. Okey. #00:31:45-6#

B: Dann gibt es noch einen Gegenstand, wo wir dann auch, zum Beispiel dann die Mutmach-Videos, wie die wir vorher dann mit den Kindern bewusst, da haben wir gesagt "Passt´s auf, das kommt. Wir müssen jetzt bewusst reflektieren, welche Übung hat euch angesprochen? Jetzt üben wir noch andere?" Ahm. Also wir haben es, glaube ich, schon sehr, wir in diesem kleinen Team, wir in diesem zweier, zwei Klassenteam haben es sehr bewusst eingesetzt. #00:32:12-7#

I: Dann höre ich es, höre ich es bei der Feedbackkultur raus. Und in jeder Klassenstunde! #00:32:19-0#

B: Genau, und wir haben genau, wir haben dann schon. Also wenn ich jetzt zum Beispiel persönlich von mir ausgehe, wenn etwas zu unruhig war in der Klasse, sehe ich das, das war mein langjähriger Zugang, nur mir war das immer wie man das Punkt auf Tafel immer, oder quasi kurz so gemacht: wir halten, wir stehen auf, und wir sind jetzt kurz bei uns und halten inne. Und Silencio, Silence und und dieses Wort in allen Sprachen aufgeschrieben und dann hab ich gesagt: "Ihr erinnert euch?" Ich hab so eine kleine Klingel in der Klasse "Steht´s auf, kurz fünf Sekunden Innehalten, wieder zu sich finden und wieder bitte mit Ruhe weiterarbeiten. "Denkst dran, was wir gemacht haben!" Und das, war so das, was wir für mich wirklich bewusst eingeführte. #00:33:04-5#

I: Aha, Okay! Und das eigentlich immer, auch während des Unterrichts, #00:33:08-0#

B: Ja, auch während des Unterrichts. #00:33:10-3#

I: Egal in welcher Stunde. #00:33:10-9#

B: Eine Intervention, weil, die geht sich aus. #00:33:15-2#

I: Mhm. mhm. #00:33:16-7#

B: Die ist schnell machbar und einsetzbar. #00:33:20-7#

I: Ja, Yes. Was habe ich noch für Fragen an sie? Sie haben haben mir eh schon so viel erzählt. Ahm. Über die Haltung haben wir schon gesprochen. Mmh. Über die Rituale haben wir jetzt eigentlich auch schon gesprochen. Welche Rituale haben sie denn eingeführt aufgrund dieses neuen, dieser Fortbildung? Aber da haben wir jetzt eigentlich auch schon. Gibt's noch was? #00:33:45-0#

B: Diese Rituale, ich mein, wir haben, wirklich bewusst, dieses in sich-ruhig-sein, kurz. #00:33:52-7#

I: Bei sich sein. #00:33:53-9#

B: Bei sich sein, ja. Und das war eigentlich so das bewusste Ritual. Ahm, was wir wirklich bewusst eingeführt, das haben wir auch aufgrund auch von PERMA, meine Kollegin kennt den Klassenrat, und wir haben auch gesagt, "Oay, wir wollen eine Partizipation von den Jugendlichen haben, eine wirkliche Gestaltung, auch mit, also eine kleine Form von Mitgestaltung. Aber wichtig. Und das, da gab es dann auch die Gesprächskultur, das miteinander umgehen, und da ist ja, sind ja auch Teile bei PERMA teach drinnen, das haben wir halt dann auch bewusst gesagt: "Könnt´s ihr euch , erinnern, was da war? Das führen wir jetzt ein." Du bist, du schaust, du bist der Zeitmanager, du führst Protokoll, und das haben sie dann, wenn man zum Beispiel den Klassenrat, da hatten wir nicht immer so viel tun können "Wir haben schon so lange kein Klassenrat mehr gemacht! Wir wollen was besprechen" da hab ich gesagt: "Okay, passt. Machen wir!" Dann haben wir halt einmal eine Mathe-Stunde dafür geopfert. #00:34:52-3#

I: Ja, aber das war sozusagen ein unmittelbares neues Ritual auf Basis des der Fortbildung. #00:35:00-2#

B: Der Fortbildung und im Zuge des PERMA teachs und des Einführen der positiven Psychologie. Und auch wenn es Schwierigkeiten gab, und die gibt es natürlich in der Schule, unter Jugendlichen speziell, oder Reibereien, wo du vielleicht auch an dein Limit kommst, und so, "Okay, was machen wir jetzt? Wie gehen wir das jetzt an?" Da gabs einen ziemlichen Konflikt. Wir müssen das besprechen, und die Pausen sind zu unruhig. Da hab ich gesagt "okay," wir machen jetzt, zum Beispiel, wir haben dann eingeführt "Okay, Kinder, wenn es nicht geht. Wir machen jetzt dann eine meditative Klassen-Pause." Wir wollen keine kollektive," da hab ich gesagt "Das ist keine Strafe". Wir sitzen gemeinsam, oder stehen in der Klasse, sitzen, ihr spielt Karten, ihr redet miteinander, ihr esst eure Jause, ihr könnt mit mir reden, wir gehen nicht raus, und wir laufen nicht. Ihr seid jetzt aufgebracht, sag ich, und wenn es solche aufregende Tage gibt, dann sitzen wir einfach oder verbringen die Pause gemeinsam ruhig und machen eine Meditations-Pause, hab ich immer dazu gesagt. #00:35:56-9#

I: Ja schön. Okay. Ja. Das ist positiv formuliert. #00:35:59-2#

B: Nicht als Strafe. Natürlich haben, wir gesagt, müssen da draußen ein bisschen entspannen, diese Spannungen. Und sie sind halt dann zwischendurch Pause, nicht mit schreiben oder was, sondern einfach eine ruhige Pause, und das war zum Beispiel ein neues Ritual, das wir auch zwangsweise eingeführt haben. Wir haben gesagt, wir müssen das, wir dürfen das nicht, wir wollen das nicht als Strafe rüberkommen lassen, sondern auch erklären, warum aus den Situationen heraus. Und das ist eigentlich dann angenommen worden, und gut. Ich sag, das ist ja nicht der Dauereinrichtung, sondern einmal zwischendurch. #00:36:35-2#

I: Frau E.! Mein Computer sagt mir gerade, dass er gleich mal den Zoom-Raum schließt, aber ich habe ihnen einen zweiten Link geschickt. Darf ich ihnen, der haut mich jetzt raus. Ich habe es leider nicht geschafft, das zu verlängern heute. Er hats nicht wollen. Aber ich habe ihnen eine e-Mail schon gerichtet und schicke ihnen jetzt einfachden neuen Link, und dann steigen wir, wenn er uns jetzt da raus haut, einfach neu wieder ein. #00:37:03-2#

B: Okay. Gerne. #00:37:05-2#

I: Ich habe nämlich noch ein paar Fragen, aber sie haben mir ja jetzt eigentlich eh schon wunderbar, das ist ja das schöne an offenen Interviews, dass ihr Redefluss eigentlich alles beinhaltet, was ich sonst einzeln abfragen hätte müssen. Aber das ist jetzt richtig, das fließt aus ihnen heraus, Frau E. #00:37:19-6#
