# Supplementary material for: PERMA.teach: a study on the effectiveness of a standardized positive education training program in Austria
Source: Front Psychol. 2025 Apr 8;16:1516572. doi: 10.3389/fpsyg.2025.1516572 (PMC12053473; doi:10.3389/fpsyg.2025.1516572)
Supplement: Supplementary file 2 [file Data_Sheet_1.ZIP › Material PERMA.teach/Material LPs/Interviews LPs Teil 4.zip!/Interviews Transkription/Interview 1 - transkribiert/Interview 1, 2.Teil transkribert.docx]

**Interview 1, 2.Teil.m4a**

I: Dankeschön! Sie haben ja jetzt alles wirklich selber so wunderbar beantwortet. Die Fragen stehen da, aber ich muss sie nicht stellen, weil es ja aus ihrer Leidenschaft, das ist schön. Ihre Motivation haben sie uns auch erklärt, also, man spürt sie ihnen in jeder Phase ihres Körpers an. Das ist toll! Die einzige, der Fragenblock, den ich jetzt noch ein bisschen auslasten habe, weil sie am Anfang gesagt haben, das mit den Lehrpersonen Unterschiede. Wir haben uns jetzt auf die Kinder und auf die Auswirkungen konzentriert. Würden sie vielleicht jetzt, darf ihre Aufmerksamkeit jetzt noch auf ihre Expositur mit ihrer Kollegin? Gibt's da Veränderungen menschlicher Art, oder können sie dort auch, wie sie manchmal sagen, dieses PERMA oder dieses positive Psychologie anwenden? Wenn man mal einen schlechten Tag hat? #00:00:50-5#

B: I, ich glaub schon, also aber wie gesagt, wir sagen immer, wir sind dreamteam. #00:00:58-5#

I: Schön! #00:01:02-8#

B: Oja. Es gibt natürlich #00:01:03-2#

I: Gibt es etwas im Team das noch besser wirkt? #00:01:07-9#

B: Es stärkt. Ja, ich glaube schon. #00:01:09-6#

I: Es stärkt warscheinlich die Beziehung. #00:01:12-8#

B: Ja, das stärkt. Und wenn man auch bewusst damit umgeht und wenn man, wenn man ein Team hat, das auch generell positiv zu Kindern eingestellt ist und das gerne unterrichtet, und mit allen auf und Abs im Beruf, die es überall gibt, und natürlich, wenn es zu schwierigen Tagen gekommen ist, dann haben wir schon gesagt:" Ach komm, Heut ist Montag. Das vergeht wieder!" #00:01:33-4#

I: Genau. Man stützt sich gegenseitig. Im Alltag, der manchmal ja auch schwer ist und nicht nur positiv. No na ned? #00:01:45-7#

B: Ja, und wir haben, da fällt mir eine Übung dazu ein, die die Kollegin mit ihnen gemacht hat. Das ist die Lach-Übung, und die Kinder haben zuerst gesagt "Das ist ja ein Blödsinn!", und die hatten, ich war in der Nebenklasse, die haben dort so gelacht, und dann hab ich gesagt "So, jetzt fangen wir auch einfach zum Lachen an" wenn wir einen schlechten Tag haben und das geht dann einfach über. #00:02:01-4#

I: Genau. Ja, eben. Das sind dann schon auch, wie soll ich sagen, wenn man gemeinsam an einer Fortbildung teilnimmt und sagt "naja, jetzt lachen wir halt" dann lacht man ja schon schon aus dieser Fortbildung heraus. Wenn man weiß, "die haben gesagt, das macht lustig", dann fangen wir halt mal an zu zweit, und dann wird es plötzlich auch lustig. #00:02:24-5#

B: Ja, also, das hat, ich glaube schon. Ich würde also, ich spreche jetzt für mich. Ich habe schon das Gefühl, dass das Spuren hinterlassen hat. #00:02:36-3#

I: Und ich und ich strapaziere jetzt sogar alle fünf Buchstaben, oder, und sag jetzt, die positiven Gefühle höre ich ihnen an. Ich hör auch diese Stärken an. Man schätzt den anderen, was der kann, und man darf seine eigenen Stärken einbringen und wird dafür gesehen, geschätzt und und eigentlich auch gemocht. Ähm, ich hör das relation, also die Beziehung raus, die sich, die vorher schon gut war. Aber. #00:03:01-9#

B: verstärkt. #00:03:02-7#

I: Und das gemeinsame, also dieses Meaning, der Sinn, oder? Man ist in diesem Positiven gemeinsam unterwegs, und das A als Accomplishment, als Ziel, wenn man dann die Kinder lachen hört oder das Feedback bekommt, "was habt ihr für tolle Feedbackkultur?" Das wäre dann so ein gemeinsames Ziel erreichen? #00:03:25-3#

B: Ja, ja, na auf alle Fälle. Ich glaube, das ist schon angekommen, und mir kommt vor, es wächst auch weiter. #00:03:31-8#

I: Mhm, das ist doch schön! Ihre Expositur mit ihrer Kollegin! Sie haben gesagt, das Schwierige ist natürlich die große andere Schule, also das Haupthaus. Ahm. Wenn man jetzt ganz genau hinschauen würde mit der Lupe, mit der Stärken-Lupe, mit der positiven. Gibt es dort Veränderungen? Vielleicht sogar in die Führung hinein, in die Direktion? #00:03:54-1#

B: Nein, das glaube ich nicht. #00:03:55-9#

I: Okay. Klar und deutlich. Okay, gut, also, weil es noch zu wenig gelebt wird oder noch noch zu wenig. #00:04:05-5#

B: Ersten bin ich zu wenig vor Ort. #00:04:06-9#

I: Mhm. #00:04:07-5#

B: Wenn ich vor Ort, vor Ort bin? Ich bin am Montag dort. Ich werd´s schauen, wie das. #00:04:15-7#

I: Ja. Das ist, es ist ja einfach auch oft man, sie haben es am Anfang so schön gesagt, wir tendieren dazu, dass man es ganz, ganz schnell und viel wollen und einfach auch oft unzufrieden ist. Aber sie kennen jetzt ihre Lehrerkollegen wahrscheinlich am besten. Vielleicht sieht man kleine Veränderungen? #00:04:34-4#

B: Aber wie gesagt, ich, vom Haupthaus kann ich jetzt nicht so viel mitreden, weil ich einfach so gut wie nie dort bin. Ahm. Da habe ich wirklich zu wenig. #00:04:42-5#

I: Und ich weiß auch nicht genau also, wobei es ja auch nicht relevant ist, jetzt aber, wie viele Lehrer vom Haupthaus bei der Ausbildung mitgemacht haben? #00:04:48-2#

B: Am Anfang waren es fast, wieviel waren denn das? Es waren eigentlich, ich sage mal, 15 haben sich angemeldet, zehn waren es, und ich glaube, Fazit: sinds´s jetzt vielleicht jetzt vier, fünf! #00:05:05-8#

I: Mhm Okay, ja, also es ist dann wenig. Es wird dann wenig, um diese kritische Größe zu haben. #00:05:15-4#

B: Aber ich glaube, es ist trotzdem vor Ort, also von zwei Kolleginnen weiß ich es, die das bewusst einsetzen, auch daran arbeiten, einen weiß ich der, der das auch lebt und der sowieso auch ein positiver Mensch ist. #00:05:31-5#

I: Mhm. #00:05:32-2#

B: Und von einer Kollegin weiß ich, die findet das ganz toll. Aber die kommt, aber es ist rundherum so viel. Die setzen das ein, und wie viel ist aber jetzt im Lehrerzimmer oder im Haus? Es ist eine besondere Situation, muss man auch dazu sagen. Es ist, wir sind quasi fusioniert worden. Es ist im ersten Stock, und im Erdgeschoss ist das Poly. Dann hat man das umgebaut, zwei Horte reingesetzt. Dann hat man im im zweiten Stock, wo unser Stock auch ist, eine Volksschule reingesetzt. Es ist auf engsten Raum sehr viel los. #00:06:09-2#

I: Ja, Eigene Gruppierungen oder ? das hat das hat. #00:06:12-4#

B: Eigene Gruppierung. Es hat sich jetzt, ich bin nicht vor Ort wie gesagt. Es wäre, es hätte sich vielleicht auch anders entwickelt. Wobei, ich glaube schon, ich weiß von der einen Kollegin, die sehr viel macht und sie sagt "Die machen nix und haben alle aufgezeigt", und ich sag "Judith, das ist wurscht. Du machst was. Es ist gut. Es ist für dich gut." Also ich bestärkt halt die, die was tun. #00:06:32-4#

I: Ja, ja, klar, im Moment kann man mehr wahrscheinlich auch nicht tun. Die Frage wäre, können wir? Haben sie das Gefühl, man könnte da was tun? Was? Was würden sie ja den Initiatoren dieses, diese dieser Fortbildung vielleicht gerade in so einer Situation für einen Tipp mitgeben können? Noch einmal anschreiben, dranbleiben? #00:06:53-9#

B: Die Eva macht das es eh sehr nett, wenn sie ihre Dinge schickt, und die Judith, die Kollegin drüben, ist eh auch dabei. "Danke fürs Mitmachen und toll, dass sie bei der Pilotschule sind." Die Kollegen machen auch bei den Umfragen mit, was ich mitbekommen haben. Ja, Anschreiben vielleicht, dass man "Danke fürs Mitmachen" und wollen sie weiter was weiß ich denn?" Also, ich glaube, das ist sowieso, es hört, wird nicht aufhören! #00:07:26-2#

I: Mhm. #00:07:26-9#

B: Also, das glaube ich nicht und ist auch, wie gesagt, was ihr vorher gesagt habe, wenn zwei jetzt nächstes Jahr das weitermachen in der Hauptschule und vielleicht doch noch jemanden dazu ins Boot holen, ist es gut. #00:07:40-4#

I: Ja, na genau, man muss ja mit denen dankbar sein. #00:07:43-8#

B: Das ist jetzt meine Erwartung, weil ich einfach dieses "Bumm, bumm, bumm" #00:07:47-8#

I: Man hätte so gerne, dass es sich schneller, schneller und in double speed verbreitet. #00:07:52-2#

B: Aber ich glaube, es ist gutes Potenzial vorhanden, es gibt Junge, und es könnte durch, es wird auch wachsen. Also, das war gut. Aber Ich bin prinzipiell positiv, also ich bleibe sicher dran. Wir sehen uns ja auch. Ich werde auch am Montag zum Beispiel, das nochmal, weil ich bin da bei der Konferenz auch dabei. Das werde ich noch einmal erwähnen beziehungsweise nur haben, auch noch mal in Erinnerung rufen, nachfragen, "wer möchte weiter, wer möchte weitermachen nächstes Jahr?" Ahm. Das ist mein Plan. #00:08:28-9#

I: Also das ist so interessant, dass dieses, also dieses Wohlbefinden, diese psychosoziale Gesundheit, dieses Mental Health, Mental Load Themen, die gerade für Lehrer ja nicht weniger geworden sind über all die Zeit, und ich finde, das ist immer so ein guter Aufhänger, weil ihr Lehrer, sage ich immer, müsst immer für die anderen euch fortbilden und wie schön wäre es, den eigenen Vorteil daraus zu sehen. Also, das macht man ja nicht nur, sondern auch für die anderen., Und das finde ich jetzt eine Möglichkeit oder den Vorteil für sich selber, weil weil die Jacke manchmal, gerade in der Not der Lehrer, Jacke manchmal näher als Hose. Und dann ist es natürlich diese Gesundheit für mich und für die anderen. #00:09:19-3#

B: Ja, aber ich glaube, wenn das jetzt weitergeht oder wie auch immer, weil ich glaube, im Herbst haben wir dann noch, haben wir eh noch ein Stärken-Cafe, glaube ich. #00:09:28-2#

I: Mhm. #00:09:28-7#

B: Und dann gibt es noch Befragung. #00:09:31-3#

I: Genau, und darum haben wir sagt, wir machen die Interviews vorher, bevor diese Befragung jetzt nachher kommt, damit man die qualitative Information jetzt so mal haben. #00:09:40-5#

B: Ich muss jetzt wirklich sagen, bewusst, ich meine, ich habs ja an Land gezogen für die Schule, und es gibt dort, und es wird ja auch von, also von den SQMs unterstützt, weil die haben jetzt nicht Zeit, das sie sich um das jetzt kümmern können, für das sind sie auch nicht da, das stimmt, ne, ähm, aber ich denke einfach, wenn das Stärken-Cafe, muss man überlegen, diese für Eva und Ingrid, ich wollte denen sowieso noch sagen: Ein großes Danke für mich, und das wird sowieso weitergetragen. #00:10:12-6#

I: Mhm.mhm #00:10:13-2#

B: Und die zwei Damen ist ja so aktiv, also das hört eh nicht auf. #00:10:21-1#

I: Gott sei dank, haben wir sie. #00:10:21-7#

B: Ich denk mir, sie haben doch schon einiges erreicht, und sie haben bei den Pilotschulen, und ich bin sicher, auch ein guter Schneeball. #00:10:28-5#

I: Ja, das ist großartig, Frau E. Jetzt habe ich noch eine Frage an sie, und zwar würde ich gern wissen, wofür sie in in dieser Fortbildung oder für diese PERMA teach Geschichte dankbar sind? Ich hätte ihnen gerne drei Gefühle abgerungen, um sie mit einem guten Gefühl, aus diesem Interview zu entlassen. Wofür sind sie dankbar? #00:10:49-0#

B: Also, ich bin dankbar, dass Ingrid und Eva getroffen habe damals, bei der bei der ersten, bei der ersten Präsentation, wo sie das vorher gestellt haben. Dann bin ich dankbar, dass sich die Kollegen am Anfang so begeistern konnte, also viele, und die Geschichte stimmt, hat gestimmt haben, dass sie das auch wirklich hören wollen und ich, das wir das umsetzen wollen, und ich bin dankbar, dass ich das umgesetzt habe, weil ich lerne auch daraus. #00:11:20-9#

I: Wie schön. Super. #00:11:24-4#

B: Mhm. #00:11:24-5#

I: Genau. Man holt ja für sich auch noch was, was raus. Das wäre es jetzt schon gewesen, Frau E.. Ich danke ihnen für ihre Zeit. Wenn sie mir noch sagen wollen, ob sie, ob ich irgendwas vergessen habe zu fragen oder ob sie noch irgendetwas anbringen wollen, dann wäre jetzt noch drei Minuten Zeit dazu? #00:11:43-6#

B: Ja, ich glaube, das wichtigste schon gesagt: es ist großartig, dieses, die ganzen Materialien, sie zwei, Ingrid und Eva sind hervorragende, aktive Frauen, die diese Kraft in die Welt bringen #00:12:00-4#

I: Ja, vielleicht in die Welt, und alle unterstützen dabei. #00:12:03-3#

B: Und ich glaube, solche Menschen braucht´s. #00:12:08-5#

I: Und solche wie sie. Liebe Frau E! Ich danke ihnen ganz, ganz herzlich, dass sie überhaupt die Zeit genommen haben, weil wir wissen das wirklich zu schätzen, in all dem Trubel auch noch befragt dazu werden. Aber es hilft natürlich, um die Ausbildung besser zu machen, zu optimieren, Schwerpunkte anders vielleicht zu setzen, einfach um zu lernen. #00:12:26-2#

B: Ja. #00:12:27-3#

I: Und das haben wir jetzt von ihnen gelernt. #00:12:29-3#

B: Ja, das ist super. Ich bin ja deswegen auch dabei. Deswegen mache ich das auch gern. Ich will ja, dass das weitergeht. #00:12:35-1#

I: Danke für ihren Beitrag. Und wünsche ihnen jetzt ein ganz schönes Wochenende, hoffentlich mit ganz viel guten Emotionen! #00:12:44-4#

B: Danke vielmals. Ihnen auch ein schönes Wochenende! #00:12:47-2#

I: Wiederschauen. Baba. #00:12:48-6#
